# Ebselen derivatives inhibit SARS-CoV-2 replication by inhibition of its essential proteins: PL^pro^ and M^pro^ proteases, and nsp14 guanine N7-methyltransferase

**DOI:** 10.1038/s41598-023-35907-w

**Published:** 2023-06-06

**Authors:** Mikolaj Zmudzinski, Wioletta Rut, Kamila Olech, Jarosław Granda, Mirosław Giurg, Małgorzata Burda-Grabowska, Rafał Kaleta, Michala Zgarbova, Renata Kasprzyk, Linlin Zhang, Xinyuanyuan Sun, Zongyang Lv, Digant Nayak, Malgorzata Kesik-Brodacka, Shaun K. Olsen, Jan Weber, Rolf Hilgenfeld, Jacek Jemielity, Marcin Drag

**Affiliations:** 1 Department of Chemical Biology and Bioimaging, Wroclaw University of Science and Technology, Wyb. Wyspianskiego 27, 50-370 Wroclaw, Poland; 2 Department of Organic and Medicinal Chemistry, Faculty of Chemistry, Wroclaw University of Science and Technology, Wyb. Wyspianskiego 27, 50-370 Wroclaw, Poland; 3 Institute of Organic Chemistry and Biochemistry, Czech Academy of Sciences, Flemingovo Nám. 2, 16610 Prague, Czech Republic; 4 Centre of New Technologies, University of Warsaw, Banacha 2C, 02-097 Warsaw, Poland; 5 College of Inter-Faculty Individual Studies in Mathematics and Natural Sciences, University of Warsaw, Banacha 2C, 02-097 Warsaw, Poland; 6 Institute of Molecular Medicine, University of Lübeck, Ratzeburger Allee 160, 23562 Lübeck, Germany; 7 Department of Biochemistry and Structural Biology, University of Texas Health Science Center at San Antonio, San Antonio, TX 78229 USA; 8 National Medicines Institute, Ul. Chełmska 30/34, 00-725 Warsaw, Poland; 9 German Center for Infection Research (DZIF), Hamburg-Lübeck-Borstel-Riems Site, University of Lübeck, 23562 Lübeck, Germany

**Keywords:** Proteases, Transferases, Drug screening, Target validation, Virology

## Abstract

Proteases encoded by SARS-CoV-2 constitute a promising target for new therapies against COVID-19. SARS-CoV-2 main protease (M^pro^, 3CL^pro^) and papain-like protease (PL^pro^) are responsible for viral polyprotein cleavage—a process crucial for viral survival and replication. Recently it was shown that 2-phenylbenzisoselenazol-3(2*H*)-one (ebselen), an organoselenium anti-inflammatory small-molecule drug, is a potent, covalent inhibitor of both the proteases and its potency was evaluated in enzymatic and antiviral assays. In this study, we screened a collection of 34 ebselen and ebselen diselenide derivatives for SARS-CoV-2 PL^pro^ and M^pro^ inhibitors. Our studies revealed that ebselen derivatives are potent inhibitors of both the proteases. We identified three PL^pro^ and four M^pro^ inhibitors superior to ebselen. Independently, ebselen was shown to inhibit the N7-methyltransferase activity of SARS-CoV-2 nsp14 protein involved in viral RNA cap modification. Hence, selected compounds were also evaluated as nsp14 inhibitors. In the second part of our work, we employed 11 ebselen analogues—bis(2-carbamoylaryl)phenyl diselenides—in biological assays to evaluate their anti-SARS-CoV-2 activity in Vero E6 cells. We present their antiviral and cytoprotective activity and also low cytotoxicity. Our work shows that ebselen, its derivatives, and diselenide analogues constitute a promising platform for development of new antivirals targeting the SARS-CoV-2 virus.

## Introduction

In the winter of 2019, an outbreak of pneumonia with flu-like symptoms emerged in Wuhan, China^1,2^. Shortly thereafter, the disease-causing pathogen was isolated and analyzed, leading to identification of the novel, highly contagious human beta*-*coronavirus SARS-CoV-2 (formerly known as 2019-nCoV)^3^. By February 2023, with over 674 million people diagnosed with Coronavirus Disease 2019 (COVID-19), the death toll exceeded 6.86 million patients worldwide^4^. Newly developed COVID-19 vaccines rely on the immunogenicity of viral spike protein (S), however emergence of novel SARS-CoV-2 Variants of Concerns (VOCs) highlight the need of new antivirals targeting the more conserved non-structural proteins (nsps) of the virus^5,6^. Various strategies have been employed to accelerate finding an effective therapy to fight the pathogen^7^. One of these strategies is drug repurposing—establishing therapeutic properties for already approved substances for new medical applications. This strategy can be supported by computational analysis, which can lower the costs, speed up the process in comparison with de novo development of new therapeutics and serve as a first stage in screening vast libraries of active compounds^8–12^. Drug repositioning has already been used in fighting COVID-19^13^. An example here is remdesivir, an antiviral agent targeting the viral RNA-dependent RNA polymerase (RdRp) that was designated to treat Ebola but has shown efficacy in shortening recovery time and reducing mortality as well as serious adverse effects in COVID-19 patients in initial studies^14^. However, after extended clinical trials, the WHO Solidarity Trial Consortium concluded that treatment with remdesivir does not prevent, or prevents only a small fraction of, deaths in hospitalized COVID-19 patients. In the study, researchers evaluated also the efficacy of other repurposed drugs—hydroxychloroquine, lopinavir, and interferon beta-1a. As a result, the drugs provided no or little benefit for hospitalized patients with no reduction in hospitalization time, mortality, and initiation of ventilation^15^. Recently, two orally administrated drugs have been introduced into the market. Pfizer’s PF-07321332 (nirmatrelvir) is a SARS-CoV-2 M^pro^ inhibitor and it is being marketed in combination with Ritonavir under the name paxlovid (https://clinicaltrials.gov/ct2/show/NCT04960202; [accessed on 21st Sep 2021]). The second drug, molnupiravir developed by Merck and Ridgeback Biotherapeutics, is a viral RNA-dependent RNA polymerase (RdRp) inhibitor (https://www.clinicaltrials.gov/ct2/show/NCT04939428; [accessed on 21st Sep 2021]). Despite this, current treatment options are critically limited and finding new therapeutics for COVID-19 patients constitutes a leading challenge for the scientific community.

To address the problem, scientists identified druggable targets among viral non-structural proteins, two of them being proteases. The SARS-CoV-2 main protease (M^pro^, 3CL^pro^, nsp5) and the papain-like protease (PL^pro^, nsp3 papain-like protease domain) enable viral replication in host cells by processing the viral polyprotein and generating 16 nsps, crucial for virus replication. SARS-CoV-2 M^pro^ generates 13 viral nsps, making it a key player in the process of virus replication and maturation^16–18^. M^pro^ is a cysteine protease with a structure highly conserved among human coronaviruses. In solution, the enzyme exists as both monomers and homodimers, but only the homodimeric form of the protease possesses the full catalytical activity^18–21^. An unusual preference for a glutamine residue at the P1 position of the substrate cleavage site sets M^pro^ apart from known human proteases. This feature can be beneficial for design and synthesis of effective, broad-spectrum antiviral agents with minimum side effects^9,18,19,21–23^. SARS-CoV-2 PL^pro^ is a viral cysteine protease proposed as an excellent target for COVID-19 treatment due to its pathophysiological roles. PL^pro^ processes viral polyproteins to generate nsp1–3 proteins. Moreover, the protease also alters the host immune response by deubiquitinating and deISGylating proteins within infected cells^24–27^. Thus, PL^pro^ inhibition would not only block the replication of the virus, but would also limit the dysregulation of cellular signaling mediated by ISG15 and ubiquitin.

2-Phenylbenzisoselenazol-3(2*H*)-one (ebselen), firstly prepared by Lesser and Weiß in 1924^28^, is a small-molecule drug with a pleiotropic mode of action in cells^29^. Ebselen is an excellent scavenger of ROS that acts as a mimic of the selenoenzyme glutathione peroxidase (GPx) and interacts with the thioredoxin (Trx) system by oxidation of reduced TrxR^30–32^. During the GPx-like activity, ebselen undergoes a series of reactions arranged in catalytic cycles. Data suggest that the mode of reactions is dependent on the cellular concentrations of thiols and hydrogen peroxide^30,33–37^. Recently, it was shown that ebselen inhibits both the SARS-CoV-2 proteases. Weglarz-Tomczak et al. evaluated ebselen and a collection of its derivatives as inhibitors of the PL^pro^, leading to identification of inhibitors with IC_50_ values in the nanomolar range^38^. Ebselen and its derivatives have also been employed in a study by Amporndanai et al., who investigated the inhibitory effectiveness of these compounds against SARS-CoV-2 M^pro^ and proposed a mechanism of the enzyme’s catalytic Cys145 selenation^39^. Tested compounds exhibited sub-micromolar IC_50_ values in recombinant enzyme assays and anti-SARS-CoV-2 activity with EC_50_ in the low-micromolar range in cellular assays. Moreover, in antiviral assays, ebselen derivatives were superior to ebselen. In another study, a library of approximately 10,000 drugs and drug candidates was screened for M^pro^ inhibitors. As a result, ebselen displayed the lowest IC_50_ among the substances tested (0.67 µM), furthermore it also displayed an antiviral effect in SARS-CoV-2-infected Vero cells^9^. In a study by Cao et al. researchers performed HTS of the NIH Clinical Collection compound library. Ebselen was amongst the most potent anticoronaviral agents in cell assays^40^. In a study conducted by Mangiavacchi et al., it was shown that introduction of a selenium atom into the structure of a quercetin derivative increases its antiviral potency nearly 24 times in comparison with quercetin, indicating the significance of organoselenium compounds in the discovery of novel antivirals^41^.

**Figure 1:**
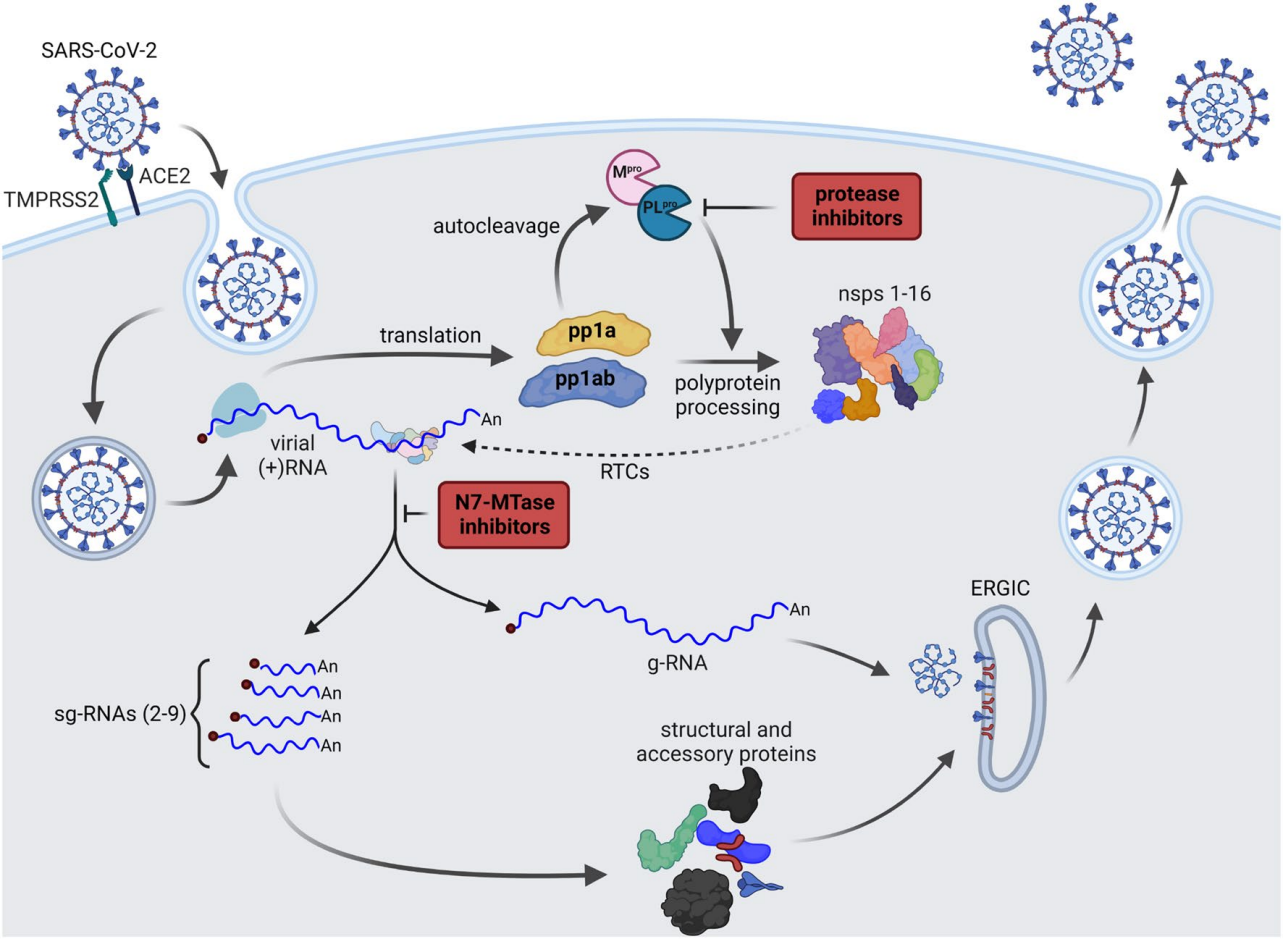
Simplified life cycle of SARS-CoV-2. The virus enters a host’s cell and releases its genome in cytoplasm. Viral RNA is translated into two polyproteins: pp1a and pp1ab. Then, due to autocleavage, two viral proteases (PL^pro^ and M^pro^) are liberated. Their main role is to further process polyproteins, what results in a release of other nsps. Next, a group of nsps form replication and transcription complexes (RTCs). RTCs are further involved in a generation of copies of viral genomic RNA (g-RNA), as well as a set of sub-genomic RNAs (sg-RNA) responsible for synthesis of viral structural and accessory proteins. Virions are assembled in endoplasmic reticulum-Golgi intermediate compartments (ERGICs). g-RNA is coated with structural N-protein and enters the ERGIC containing M, E, and S glycoproteins. Then, the virions are released by exocytosis^47,48^. Inhibition of M^pro^, PL^pro^, and N7-MTase may lead to suppression of virus replication. Protease inhibition stops the generation of nsps, while N7-MTase inhibition prevents the synthesis of stable transcripts of the viral RNA. Created with BioRender.com.

Recent studies revealed that ebselen also inhibits the RNA cap guanine N7-methyltransferase^42^ and exonuclease^43^ nsp14 activities from SARS-CoV-2. Nsp14 is a bifunctional enzyme with an independently functioning N7-methyltransferase (N7-MTase) domain and an nsp10-dependent exonuclease domain^44^. The enzyme is involved in 5′-end capping of newly synthesized viral mRNAs, crucial for viral transcript stability and protein biosynthesis. The role of nsp14 N7-MTase is to catalyze the reaction of methyl group transfer from *S*-adenosyl-l-methionine (SAM) onto the N7-position of guanosine 5′-triphosphate located at the 5′ RNA end (Gppp–RNA), resulting in cap-0 formation^43^. Inhibition of viral N7-MTases has already been shown to suppress viral replication^45^, including that of SARS-CoV^44^. Thus, the nsp14 enzyme is considered a good target for antiviral drug development (Fig. 1)^46^.

**Figure 2:**
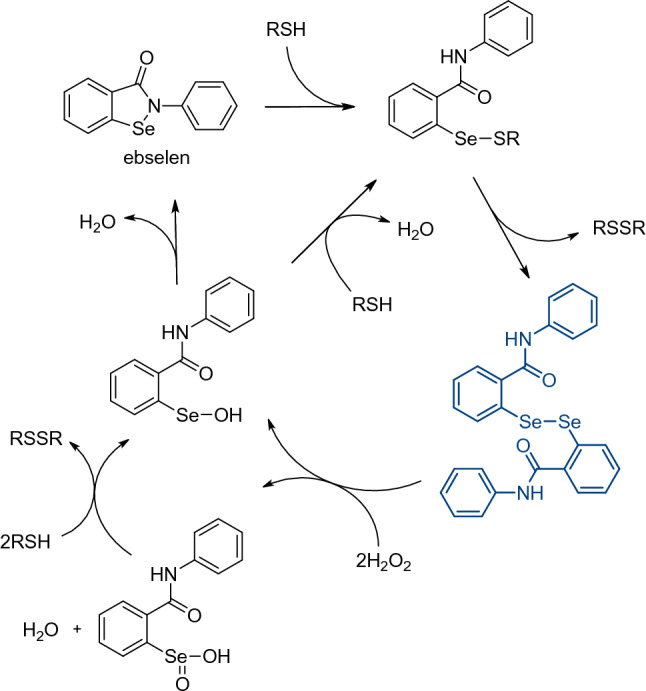
Plausible catalytic cycle of ebselen involving hydrogen peroxide reduction, including formation of the ebselen open form (dark blue color)^33,37^.

The efficacy of ebselen and other organoselenium compounds has been previously evaluated for HIV^49,50^, HSV2^51^, HCV^52^, and Zika virus^53^ infections. Moreover, a recent report presents ebselen and its derivatives as potent inhibitors of SARS-CoV-2 PL^pro^^38^. Currently, ebselen is being evaluated in a phase 2 clinical trials as an oral therapeutic in moderate and severe COVID-19 patients (https://clinicaltrials.gov/ct2/show/NCT04484025; https://clinicaltrials.gov/ct2/show/NCT04483973; [accessed on 10th Aug 2021]). In this work we investigated ebselen derivatives and analogues as potential anti-SARS-CoV-2 agents. Ebselen’s low toxicity and ongoing clinical trials make it attractive as a lead compound. First, we screened a collection of 23 ebselen and 11 ebselen diselenide derivatives and determined the half-maximum inhibitory concentration (IC_50_) values for the most promising 2-phenylbenzisoselenazol-3(2*H*)-ones to evaluate their properties as SARS-CoV-2 PL^pro^ and M^pro^ inhibitors. Next, employing a Py-FLINT fluorescence assay, we evaluated nsp14 N7-MTase inhibitory properties for selected ebselen derivatives against the recombinant enzyme. Ebselen’s ‘open form’—bis[2-(*N*-phenylcarbamoyl)phenyl]diselenide—is proposed as one of the intermediates during ebselen catalytic cycles in living organisms (see Fig. 2) and in cellular environment the compound could act as reservoir of corresponding benzisoselenazolones that inhibit viral enzymes and participate in protection against H_2_O_2_ and other ROS^54^. Hence, we determined anti-SARS-CoV-2 activity in an RNA-reduction-based assay and cytopathic effect-based assays in Vero E6 cells for 11 bis[2-(*N*-arylcarbamoyl)phenyl]diselenides. Lastly, we show that ebselen may constitute a potential lead compound for development of novel antiviral agents with minimal cytotoxic action in vivo. The results can be useful in the design of new active compounds targeting the proteases encoded by SARS-CoV-2, to be applied in COVID-19 treatment.

## Results and discussion

### Compound library preparation

**Figure 3:**
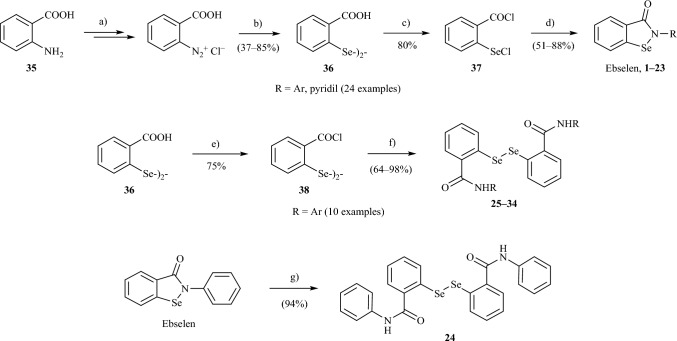
Preparation of ebselen, its derivatives and their ‘dimeric’ form analogues **1–33**. Reagents and conditions: (a) (i) aq. HCl, (ii) NaNO_2_, − 7 to + 7 °C, (b) (i) NaSeSeNa, MeOH, NaOH or LiSeSeLi, THF, HMPTA, − 7 to + 5 °C, (ii) aq. HCl, (c) 7 equiv SOCl_2_, cat. (DMF), benzene, reflux, (d) RNH_2_, Et_3_N, MeCN or DCM, (e) 3.5 equiv SOCl_2_, cat. (DMF), benzene, reflux, (f) RNH_2_, Na_2_CO_3_, DCM, (g) H_2_N-NH_2_∙H_2_O, MeOH, reflux. (Carried out in accordance with Refs.^60,62–64^.

The synthesis of biologically active organoselenium compounds is in the scope of many research teams around the world^35,55,56^. Ebselen and other benzisoselenazol-3(2*H*)-ones have been previously prepared by several ways^57,58^. Structures of compounds included in the collection are presented in Table 1. The general procedure for preparation of ebselen, its derivatives (**1**–**23**), and their analogues (**24**–**34**) is presented in Fig. 3. 2,2′-dicarboxydiphenyl diselenide (**36**) was obtained as a result of consecutive protonation, diazotation and disodium or dilithium diselenide selenenylation of anthranilic acid (**35**). In the next steps, the reactions of diselenide **36** with thionyl chloride in benzene in the presence of DMF at solvent reflux produced 2-(chloroseleno)benzoyl chloride (**37**) or bis[(2-chlorocarbonyl)phenyl] diselenide (**38**) depending on the amounts of thionyl chloride used. Tandem selenenylation/acylation reaction^59^ of aniline or its phenyl ring substituted derivatives with 2-(chloroseleno)benzoyl chloride (**37**) in anhydrous MeCN, or DCM, in the presence of dry Et_3_N base gave ebselen and its derivatives **1–23**. The acylation reaction of phenyl ring substituted anilines with chloride **38** in anhydrous DCM in the presence of anhydrous Na_2_CO_3_ as a base gave ebselen ‘dimeric’ form analogues **25–34**^60^. In particular, carbamoylphenyl diselenide **24** was prepared by reduction of ebselen with hydrazine monohydrate in methanol as a solvent^61^. The purity of the compounds was > 95% as confirmed by LC–MS analysis (see Supplementary Information).

**Table 1:** Composition of the compound library employed in the study.

benzisoselenazol-3(2*H*)-ones				bis(2-carbamoylaryl)-phenyl diselenides	
**R:**		**R:**		**R:**	
**Ebselen**		**12**		**24**	
**1**		**13**		**25**	
**2**		**14**		**26**	
**3**		**15**		**27**	
**4**		**16**		**28**	
**5**		**17**		**29**	
**6**		**18**		**30**	
**7**		**19**		**31**	
**8**		**20**		**32**	
**9**		**21**		**33**	
**10**		**22**		**34**	
**11**		**23**

FT-IR spectra were measured in the crystal lattice or in KBr and the ^1^H-, ^13^C-, ^77^Se-, and ^19^F-NMR spectra were generally measured in DMSO-*d*_6_. For ebselen and its analogues **1–23**, the wavenumbers corresponding to the stretching vibration of carbonyl (C=O) groups were around 1583–1649 cm^−1^, nitrogen carbon single bonds with substituent (C-N) bands were at 1305–1363 cm^−1^, and the C–Se at 727–747 cm^−1^, moreover, vibration of the diselenides **24–34** are in agreement with data reported previously^60^. In the benzisoselenazol-3(2*H*)-one region of the NMR spectra measured in DMSO-*d*_6_, the proton H-4, H-5, H-6, and H-7 resonances of compound **1–23** were observed at: 7.85–7.94, 7.45–7.51, 7.52–7.78, and 8.05–8.12 ppm, respectively, and the carbon C=O, C-3′, C-4, C-5, C-6, C-7 and C–Se resonance generally were observed at 164.87–166.08, 126.43–128.53, 127.73–128.22, 126.06–126.52, 132.07–133.01, 125.71–126.07 and 138.50–140.59 ppm, respectively, while the carbon atom of a phenyl substituent linked with heteroaromatic (PhC-1) resonance were observed at 119.7–146.3 ppm, dependent on the substituents used. The ^77^Se-NMR resonance of benzisoselenazolones (**1–33** observed at 914.33–974.90 ppm) and diselenides (**25–34** observed at 443.48–447.93 ppm) are in agreement with previously published data^64–66^. For benzisoselenazolones **1**, **14**, **15**, **17**, and **5**, we found selenium fluoride spin–spin coupling constants ^4^*J*(^77^Se–^19^F) and ^5^*J*(^77^Se–^19^F) values of 15.1–25.5 Hz, and 10.7 Hz, respectively. No ^6–7^*J*(^77^Se–^19^F) spin–spin constants were measured.

### Compound library screening and IC_50_ determination for SARS-CoV-2 PL^pro^ and M^pro^

First, we evaluated the inhibitory properties for ebselen and the compounds **1**–**23** at 1 µM inhibitor concentration with 100 nM SARS-CoV-2 PL^pro^ and at 100 nM inhibitor concentration with 100 nM SARS-CoV-2 M^pro^. For the PL^pro^, we used an Ac-LRGG-ACC fluorogenic substrate with a structure based on the C-terminal epitope of Ub and ISG15 proteins as well as on the nsp1/2, nsp2/3, and nsp3/4 cleavage sites in the coronaviral polyprotein. For the M^pro^, we used a novel tetrapeptide fluorogenic substrate, QS1 (Ac-Abu-Tle-Leu-Gln-ACC; K_M_ = 207.3 ± 12 µM, k_cat_/K_M_ = 859 ± 57 M^−1^ s^−1^)^22^. PL^pro^ screening resulted in identification of only benzisoselenazolone **7** with higher potency than ebselen. However, compound **7** differed from the other compounds in the collection as it was the only investigated ebselen derivative with a 3-substituted pyridinyl moiety instead of a substituted phenyl ring. For the M^pro^, the best hits were compounds **10** and **17**. Compound **10** represents monosubstituted derivatives with a nitro group at the *para* position, while **17** is a derivative with 2-fluoro and 5-chloro substitutions in the aromatic ring. We observed that 2,4-dimethoxy derivative **16** displays potency towards both of the proteases close to that of ebselen, however, in comparison with ebselen, its toxicity evaluated in the A549 human cell line was 10 times lower^60^. In general, substitutions within the phenyl ring of ebselen boost inhibition of M^pro^ as we identified only 3 compounds (**6**, **7** and **12**) with a potency lower than for ebselen. See Table S2 in supplementary information for screening results^39^.

Based on the screening results, we selected ebselen and seven of its derivatives for further inhibitory property evaluation in IC_50_ assays. We chose compounds: (a), exhibiting the highest potency towards M^pro^ (**10**, **17**) or PL^pro^ (**7**) in the screening; or (b), displaying relatively high inhibition towards both the investigated proteases (**3**, **16**, **20**, **21**). During the assays, IC_50_ values for PL^pro^ were in the micromolar range while for M^pro^, they were in the low nanomolar range. The results are presented in Table 2. For the reference inhibitor, ebselen, IC_50_ values were 1.12 ± 0.06 µM for PL^pro^ and 30.91 ± 2.67 nM for M^pro^. Compound **7**, which was the best hit in the PL^pro^ inhibitor screening assay, indeed had the lowest IC_50_ value (0.58 ± 0.04 µM) among the tested compounds. Despite ebselen being the second best PL^pro^ inhibitor in the screening assay, we found that two other compounds (**17**, **21**) displayed slightly lower IC_50_ values. Compounds **10** and **17**, which were selected for the analysis as the best M^pro^ inhibitors, displayed lower IC_50_ values than ebselen. The most potent M^pro^ inhibitor with IC_50_ = 15.24 nM was compound **17**, the second best hit from the screening experiment. Interestingly, the best hit **10** displayed an IC_50_ value similar to values determined for **3** and **21** (27.95 nM, 25.69 nM, and 27.37 nM respectively). For **16** and **20**, we observed that despite a higher potency in the screening assay, the IC_50_ values determined for these compounds were higher than for ebselen.

**Table 2:** Inhibitory activity of ebselen and its selected derivatives against SARS-CoV-2 PL^pro^ and M^pro^. For IC_50_ graphs see Supplementary Information Fig. S1.

		SARS-CoV-2 PL^pro^	SARS-CoV-2 M^pro^
R:		IC_50_ (µM)	IC_50_ (nM)
**Ebselen**		1.12 ± 0.06	30.91 ± 2.67
**3**		1.255 ± 0.10	25.69 ± 2.64
**7**		**0.578 ± 0.04**	49.55 ± 2.95
**10**		1.885 ± 0.10	27.95 ± 5.10
**16**		0.990 ± 0.06	52.50 ± 4.51
**17**		2.067 ± 0.08	**15.24 ± 4.58**
**20**		1.038 ± 0.08	37.81 ± 3.28
**21**		1.288 ± 0.05	27.37 ± 2.35

Significant values are in bold.

IC_50_ is an assay-dependent measure. Low IC_50_ values obtained for the compounds are probably due to the used substrates’ properties. SARS-CoV-2 PL^pro^ possesses deubiquitinating activity and used Ac-LRGG-ACC substrate has relatively low k_cat_/K_M_ value (k_cat_/K_M_ ≈ 900 s^−1^ M^−1^). Optimal substrates for this enzyme are based on ubiquitin or ISG15 molecules^26^. For SARS-CoV-2 M^pro^ we used our novel tetrapeptide QS1 fluorogenic substrate, that has a lower k_cat_/K_M_ value (k_cat_/K_M_ = 859 s^−1^ M^−1^) in comparison to FRET substrates^22^.

### Inhibition of nsp14 N7-MTase by 2-phenylbenzisoselenazol-3(2H)-ones

Selected ebselen analogues—**3**, **7**, **10**, **16**, and **17** were further tested for their inhibitory properties towards nsp14 N7-MTase. To determine their IC_50_ values, we used the previously described fluorescence assay Py-FLINT^42,67^. To this end, the Py-FLINT probe (1 μM) was incubated with SAM cosubstrate (20 μM), nsp14 enzyme (40 nM), and half-log inhibitor dilutions in 50 mM Tris–HCl pH 7.5 buffer at 30 °C. The reaction progress was monitored in 96-well cells by registering the fluorescence intensity signal with a 1-min time interval. Using the initial reaction course, the values of initial rates *V* were calculated. To calculate IC_50_ values from the obtained dependences of initial rates versus inhibitor concentration, we fitted a four-parameter dose–response equation, assuming a variable Hill slope *p* (Table 3, Fig. S2).

**Table 3:** IC_50_ and Hill coefficient (*p*) values determined for SARS-CoV-2 nsp14 N7-MTase using the Py-FLINT assay. For inhibition curves see Supplementary Information Fig. S2.

		SARS-CoV-2 nsp14	
**R:**		IC_50_ (µM)	*p*
**Ebselen**		**0.35 ± 0.03**	**2.74 ± 0.47**
**3**		**0.35 ± 0.02**	**3.12 ± 0.47**
**7**		**0.35 ± 0.02**	**4.08 ± 1.33**
**10**		3.08 ± 0.46	1.73 ± 0.35
**16**		0.42 ± 0.04	2.36 ± 0.32
**17**		3.83 ± 0.35	2.15 ± 0.28

Significant values are in bold.

High values of the hill slope were observed for the most potent inhibitors (compounds **3**, **7** and ebselen control) and we assume that they result from limitations of applied fluorescent method. The range of inhibition that can be studied in competition experiment is limited by the fluorescent probe affinity to the protein. We believe that a better fluorescent substrate with lower Michelis-Menten constant value would enable better distinguishment between strong nsp14 inhibitors visible in p ~ 1. Compounds **3**, **7** and **16** had IC_50_ values comparable to ebselen (0.35–0.42 μM), indicating they are potent inhibitors of nsp14. Compounds carrying 4-nitrophenyl (**10**) and 5-chloro-2-fluorophenyl (**17**) substitutions had one order of magnitude higher IC_50_ values (3.08 ± 0.46 μM and 3.83 ± 0.35 μM, respectively). The same substitutions caused an increase in inhibitory potency against M^pro^, which implies that phenyl ring substitutions are a possible route towards tailoring selectivity of the compounds. Overall, the results indicate that ebselen and its derivatives may act as multi-target inhibitors of SARS-CoV-2 protein activity.

### Ebselen diselenide derivatives as SARS-CoV-2 M^pro^, PL^pro^, and nsp14 inhibitors

To get a deeper insight into the biological activity of organoselenium compounds, we included a collection of 11 diselenides—open forms of various ebselen derivatives in our study. First, we performed a screening for PL^pro^ and M^pro^ inhibitors according to a protocol employed for benzisoselenazol-3(2*H*)-ones. Screening revealed that diselenides inhibit both of the proteases. Diselenides generally inhibited M^pro^ more poorly than ebselen; however, contrary to benzisoselenazolones, for PL^pro^, diselenides displayed a higher potency than the reference compound (for full screening results see Supplementary Information Table S3). The IC_50_ parameter for protease inhibition could not be assessed due to the high hydrophobicity of the compounds and their precipitation in the assay buffer. Next, we tested diselenides for anti-N7-MTase activity employing the Py-FLINT assay. All of the tested compounds displayed IC_50_ values in the high nanomolar or low micromolar range (Table 4). We observed that for compound **25** with a 2-fluorosubstituted phenyl ring, the IC_50_ is 3 times lower (0.12 ± 0.01 µM) than for ebselen. The ‘dimeric form’ of ebselen **24** and its 3-fluorosubstituted derivative **27** also displayed an IC_50_ around two times lower than ebselen. Results for these compounds correlate with their low EC_50_ values in CPE and RNA-based assays (Table 5).

**Table 4:** Ebselen diselenide derivatives as SARS-CoV-2 nsp14 inhibitors. IC_50_ was determined in the Py-FLINT assay. Conditions: probe [S_1_] = 1 μM, SAM cosubstrate [S_2_] = 20 μM, nsp14 [E] = 40 nM. For inhibition curves see Supplementary Information Fig. S3.


IC_50_ (µM)			IC_50_ (µM)		
R:		SARS-CoV-2 nsp14	R:		SARS-CoV-2 nsp14
**Ebselen**		0.35 ± 0.03	**29**		4.5 ± 1.3
**24**		0.21 ± 0.02	**30**		11.5 ± 1.4
**25**		**0.12 ± 0.01**	**31**		0.91 ± 0.10
**26**		1.82 ± 0.32	**32**		12.1 ± 1.1
**27**		0.18 ± 0.04	**33**		1.06 ± 0.05
**28**		0.58 ± 0.04	**34**		2.10 ± 0.58

Significant values are in bold.

**Table 5:** Anti-SARS-CoV-2 activities (EC_50_) and cytotoxicity in Vero E6 cells (CC_50_) for investigated diselenides determined by RNA reduction- and CPE-based assays. Remdesivir was used as a positive control. For inhibition curves see Supplementary Information Figs. S4–S6.

		EC_50_ (µM) CPE	EC_50_ (µM) RNA	Titer reduction at EC_90_ (log_10_PFU/mL)	CC_50_ (µM)
R:					
**Ebselen**		34 ± 7.9	n.d.	n.d.	~ 30
**24**		1.5 ± 0.13	**1.0 ± 0.14**	1.5	> 50
**25**		3.1 ± 0.36	1.9 ± 0.15	3.3	~ 60
**26**		37 ± 2.4	n.d.	n.d.	> 50
**27**		**0.7 ± 0.13**	1.5 ± 0.15	0.4	> 50
**28**		7.6 ± 0.71	2.7 ± 0.23	0.5	> 50
**29**		29 ± 3.5	n.d.	n.d.	> 50
**30**		1.1 ± 0.15	1.9 ± 0.1	1.1	> 50
**31**		7.1 ± 0.84	2.8 ± 0.31	0.3	> 50
**32**		22 ± 2.5	n.d.	n.d.	> 50
**33**		20 ± 1.4	1.7 ± 0.08	0.3	> 50
**34**		9.3 ± 0.92	3.4 ± 0.2	1.3	> 50
**Remdesivir**		0.1 ± 0.01	0.66 ± 0.06	3.1	> 50

*n.d.* not determined, *CPE* cytopathic effect-based assay, *RNA* RNA reduction-based assay, *PFU* plaque forming units.

Significant values are in bold.

### Evaluation of ebselen diselenide derivatives: anti-SARS-CoV-2 activity in Vero E6 cells

In vitro assays with recombinant enzymes showed that ebselen, its derivatives, and analogues possess inhibitory activity towards SARS-CoV-2 M^pro^, PL^pro^, and nsp14. Knowing that ebselen diselenide takes part in ebselen’s catalytic cycles and that oxidative stress plays an important role in SARS-CoV-2 infection^68^, we assumed that ebselen analogues—bis(2-carbamoylaryl)phenyl diselenides—could also possess antiviral activity in cells. Our next step was the evaluation of antiviral properties and cytotoxicity of selected ebselen analogues in cellulo in the Vero E6 cell line^69^. For this experiment, we included the available dimeric forms of ebselen derivatives (structures presented in Table 5). To get a deeper insight into the activity of tested compounds, we performed four tests: cytopathic effect-based assay, RNA reduction-based assay, virus titer reduction assay, and cytotoxicity assay. Compounds with an EC_50_ higher than 20 µM in the CPE-based assay were excluded from the RNA reduction-based and virus titer reduction assays. We used remdesivir as a positive control anti-SARS-CoV-2 agent. Besides ebselen, the CC_50_ of all tested compounds exceeded 50 µM, indicating their generally low cytotoxicity. Moreover, ebselen displayed the second highest EC_50_ in the CPE-based assay. However, for ebselen diselenide (bis(2-carbamoyl)diphenyl diselenide, **24**), we observed the strongest antiviral response (EC_50_ = 1.0 ± 0.14 µM in the RNA reduction-based assay) and the third strongest cytoprotective effect (EC_50_ = 1.5 ± 0.13 µM in the CPE-based assay). The highest cytoprotective effect was observed for bis[2-(3-fluorophenylcarbamoyl)]phenyl diselenide (**27**), which was the only diselenide with EC_50_ in the nanomolar range (EC_50_ = 0.7 ± 0.13 µM). The compound also displayed high antiviral activity with the second lowest EC_50_ (1.5 ± 0.15 µM) in the RNA reduction-based assay. Another potent compound was **30**, with a 4-chloro and 2-fluoro substituted phenyl ring, as it displayed the second lowest EC_50_ in the CPE-based assay. In most cases, we observed that for diselenides with methyl (**32** and **33**, with chlorine counterparts) or larger substituents (**28**, **31**—methoxy, and **26**, **29**—trifluoromethyl groups), the cytoprotective effect was decreased, but the antiviral activity did not change significantly compared to compounds with only halide substituents in the phenyl ring. However, compound **34** with a 5-chloro-2-fluoro substituted phenyl ring displayed the highest EC_50_ in the RNA reduction-based assay and had considerably higher EC_50_ in the CPE-based assay compared to other halide-substituted ebselen diselenide derivatives (**25**, **27**, **30**). We observed that especially trifluoromethyl groups hampered the activity of the compounds, resulting in the highest EC_50_ values in the CPE-based assay. The anti-SARS-CoV-2 potency of diselenide derivatives was further confirmed by the determination of maximum virus titer reduction at EC_90_ concentration. The virus titer reduction ranged from 0.3 to 3.3 log_10_PFU/mL with the compound **25** displaying the best virus titer reduction followed by compound **24**, **34**, and **30**.

## Conclusions

With over 6.86 million deaths caused by COVID-19, the need for a widely accessible, effective and safe therapy against coronaviral diseases is crucial for public health. A promising strategy involves M^pro^ inhibition and this approach can lead to novel, broad spectrum anticoronaviral drugs^18^. Recently, repurposing efforts enabled identification of ebselen as a potential drug against COVID-19, probably due to its action as an inhibitor of the SARS-CoV-2 main protease^9^. This concept was later supported by Amporndanai et al., who proposed a mechanism for selenation of M^pro^’s catalytic Cys145 residue by ebselen^39^. Furthermore, using molecular dynamics simulations, Menéndez et al. explored noncovalent interactions between the M^pro^ and the ebselen molecule. Researchers found two possible interaction sites: one located within the active site and the second in the region involved in dimer formation^70^. Promising results coming from studies of ebselen as anti-SARS-CoV-2 agent inspired researchers to explore also it’s derivatives as potential antivirals. Węglarz-Tomczak et al. investigated ebselen and its analogues as PL^pro^ protease inhibitors^38^. Qiao et al. designed and synthesized a series of ebselen derivatives to improve the inhibition of the SARS-CoV-2 M^pro^. Researchers identified three compounds, that were superior to ebselen in cellular antiviral activity assays performed in HPAepiC cells^71^. Sun et al. investigated ebselen, ebsulfur and their derivatives as M^pro^ inhibitors. In non-reducing conditions all of the compounds displayed inhibitory properties. However, the inhibition was diminished in DTT-dependent screening assay, what led to the conclusion, that ebselen is a promiscous cysteine protease inhibitor and its antiviral activity in cells may not be a result of direct protease inhibition. The role of ebselen as a protease inhibitor has been also questioned by Ma et al., who showed, that ebselen binds nonspecifically to active sites of various cysteine proteases in the lack of reducing agent^72^. In another study by the Wang’s group ebselen displayed no M^pro^ inhibition in cells in Protease-Glo luciferase and Flip-GFP assays. Authors performed in vitro FRET substrate assay using recombinant enzyme and showed, that in presence of reducing agent ebselen does not inhibit the protease^73^. Additionally, employing Flip-GFP assay, researchers observed no SARS-CoV-2 PL^pro^ inhibition by ebselen in 293T-ACE2 cells^74^. No cellular M^pro^ inhibition was reported also by Heilmann and colleagues, who developed a novel, VSV-based assay for evaluation of protease inhibitors in cells^75^.

Ebselen has a pleiotropic mode of action that is a result of its reactivity towards cysteine residues affecting many biological targets^29,31,76^. One of the plausible explanations for high antiviral activity of ebselen and its derivatives might be their function as mimetics of GPx selenoenzymes, that are crucial in maintaining low levels of ROS (Fig. 2). The oxidative stress plays a major role in viral infections^77^, thus, antioxidants such as organoselenium compounds may provide a beneficial outcome. What is more, the GPx1 isoform was proposed as a possible SARS-CoV-2 M^pro^ cellular substrate, thus presence of ebselen in cells would counteract the depletion of the enzyme^78^. Additionally, Du et al. have shown, that SARS-CoV-2 M^pro^ activity might be promoted by the oxidative stress^79^. Thus, antioxidant activity of ebselen would indirectly decrease the activity of SARS-CoV-2 M^pro^, however, this concept needs further investigation and experimental confirmation.

Organoselenium compounds may display toxicity at higher concentrations due to their pan-reactivity toward thiol groups^76^. However, the clinical safety of ebselen has been proven in multiple studies what makes this substance a good candidate for further structure optimization. Ebselen belongs to the NIH Clinical Collection compound library, that gathers substances that are not currently clinically used, but with history of usage in clinical trials in humans. Its efficacy and safety in humans have been evaluated in various studies. In a phase I clinical trial of ebselen as a potential treatment for hearing loss in humans, patients tolerated a single dose of 1600 mg^80^. In a phase IIa double-blind placebo study of ebselen in treatment of mania or hypomania, the drug was administrated for three weeks in 600 mg doses. As an outcome, adverse effects in both groups were comparable^81^. Administration of ebselen for two weeks in doses of 300 mg a day also was proven to be safe for patients suffering from acute ischemic stroke^82^. Eventually, due to its favorable pharmacokinetics and safety profile, ebselen is being investigated as a safer alternative to lithium salts in bipolar disorder^83^.

In our study, we utilized a collection of ebselen derivatives and analogues to evaluate their SARS-CoV-2 PL^pro^ and M^pro^ inhibitory properties. As ebselen has been identified as a potent nsp14 N7-MTase inhibitor, we also evaluated our series of ebselen analogues against SARS-CoV-2 nsp14. First, we screened a library of organoselenium compounds. Next, we determined IC_50_ values for selected compounds. The most potent PL^pro^ inhibitor, 2-(3-hydroxypyridin-2-yl)-1,2-benzisoselenazol-3(2*H*)-one, displayed the highest potency in the screening assay and the lowest IC_50_ value (0.58 µM). IC_50_ determination enabled identification of two more compounds with inhibitory properties similar to ebselen. These compounds were 2,4- and 2,5- disubstituted derivatives of ebselen that displayed lower potency during screening, but also slightly lower IC_50_ parameters than for the reference inhibitor. A similar analysis for the M^pro^ enabled identification of four compounds displaying higher potency during screening and a lower IC_50_ parameter. Two of them had a monosubstituted phenyl ring at the *para* and *ortho* positions, and two were disubstituted ebselen derivatives. The best inhibitor with an IC_50_ value approximately 2 times lower than for ebselen was 2-(5-chloro-2-fluorophenyl)-1,2-benzisoselenazol-3(2*H*)-one. Among the ebselen derivatives tested, we found compounds with 2-bromophenyl and 2-(3-hydroxypyridin-2-yl) modifications having inhibitory properties towards nsp14 similar to ebselen.

Another part of the research was assessment of antiviral and cytoprotective activity of ebselen and its diselenide analogues. We tested 12 selenoorganic compounds and remdesivir (positive control) in Vero E6 cells. CPE and RNA reduction-based assays revealed that this class of compounds could be a source of promising candidates for new antiviral agents. Cytotoxicity of the diselenides was generally low (CC_50_ > 50 µM). We observed that for ebselen diselenide and 3 out of 4 diselenides with halogen substitutions in the phenyl ring, EC_50_ in the CPE reduction-based assays was the lowest. Ebselen diselenide also displayed the highest antiviral activity in the RNA reduction-based assay. Substitution with bigger groups resulted in lower cytoprotective activity (higher EC_50_ in the CPE reduction-based assay), but a similar effect was not observed for antiviral activity.

In this work, we showed that in non-reducing conditions ebselen derivatives with substitutions and other modifications within the phenyl ring generally possess good inhibitory properties against both of the proteases and the N7-guanine methyltransferase encoded by the novel coronavirus in in vitro assays. Moreover, ebselen diselenide derivatives possess high antiviral and cytoprotective activity. The results constitute a promising platform for novel therapeutics and we believe that the data can be used to facilitate efforts towards new anticoronaviral drugs to be used for the treatment of COVID-19.

## Materials and methods

### Compound library preparation

All solvents were distilled before use. Commercially available reagents were used without further purification. Selenium powder (100 mesh) (Sigma-Aldrich, Saint Louis, MO, USA) used for Na_2_Se_2_ and Li_2_Se_2_ preparation had a purity of ≥ 99.5%. Freshly distilled MeCN was redistilled twice over P_2_O_5_ before preparation of ebselen and its derivatives. MeOH was distilled slowly over a mixture of LAH and CaH_2_ before hydrogenation of ebselen. CH_2_Cl_2_ (DCM) was distilled over P_2_O_5_ before preparation of ebselen analogues **25–34**. Triethyl amine (Et_3_N) (POCh, Gliwice, Poland), distilled over NaOH, was stored over NaOH pellets. Anhydrous sodium carbonate (Na_2_CO_3_) (POCh, Gliwice, Poland) was ground in a mortar before use. The intermediates, 2-(chloroseleno)benzoyl chloride and bis[(2-chlorocarbony)phenyl] diselenide were prepared from anthranilic acid and elemental selenium via the formation of 2,2′-dicarboxydiphenyl diselenide—a key intermediate, according to the literature procedure^60,61,63,64^. Preparative column chromatography was performed on Merck Si60 silica gel (63–200 µm). Analytical TLC was performed on PET foils precoated with silica gel (Merck silica gel, 60 F254) (Sigma-Aldrich, Saint Louis, MO, USA), and were visualized with light (λ_max_ = 254 nm), or by staining with iodine steam. Melting points were determined on an Electrothermal IA 91100 digital melting-point apparatus using the standard open capillary method. IR spectra (4000–400 cm^−1^) were recorded in KBr plates on a Perkin-Elmer 2000 FT-IR spectrometer or on a Fourier transform, Bruker VERTEX 70V spectrometer using diamond ATR accessory. Absorption maxima are reported in wavenumbers (cm^−1^). ^1^H-NMR and ^13^C-NMR spectra (300.1, 399.8, 600.6 MHz and 75.48, 100.5, 151.0 MHz, respectively) were recorded on a Bruker DRX 300 (Bruker, Rheinstetten, Germany), Jeol 400yh (Jeol, Tokyo, Japan) and Bruker Avance II 600 (Bruker, Poznań, Poland) instruments. NMR spectra recorded in CHCl_3_-*d*_1_ and DMSO-*d*_6_ were referenced to the respective residual ^1^H or ^13^C signals of the solvents, and chemical shifts (δ) are given in parts per million (ppm), and coupling constants (*J*) are in Hz. ^19^F-NMR and ^77^Se-NMR (376.2 and 76.24 MHz, respectively) were collected on Jeol 400yh instrument. High-resolution mass spectra were collected using electrospray ionization on a Waters LCT Premier XE TOF instrument.

The literature procedure was adapted for the preparation of dilithium diselenide^62^, diaryl diselenides **24**^84^, **28**^60,63^, **31**^60^, **32–33**^63^, **36**^60,64^, **38**^60^, and 2-(chloroseleno)benzoyl chloride (**37**)^60^. Purity and homogeneity of known compounds were confirmed by measuring their m.p. for ebselen^60^, **1–2**^58^, **3**^57^, **7–8**^60^, **9**^85^, **11**^86^, **16**^60^, **19–22**^63^, **23**^60^, **24**^87^, **26**^87^, **28**^63^, **29**^87^, **31**^60^, **32–33**^63^, **36**^86^, **37**^88^, and **38**^86^, or FT-IR spectra for ebselen^57^, **3**^57^, **10**^66^, **11**^86^, **16**^60^, **31**^60^, ^1^H- and/or ^13^C-NMR spectra for **1–2**^58^, **7**^59,60^, **8**^63^, **10**^86^, **11**^86^, **16**^60^, **19–22**^63^, **23**^60^, **24**^84^, **28**^63^, **31**^60^, **32**^63^, and ^77^Se-NMR spectrum for **10**^66^, and HRMS for **16**^60^, and comparing them with literature data. All new **13**, **30**, **34**, uncharacterized, **4**^89^, **5**^87^, **6**^90^,** 9**^85^, **12**^91^, **14**^91^, **15**^92^, **17–18**^93^, **25**^92^, **27**^92^, and spectroscopically uncharacterized **26**, **29**^87^ selenium species were fully characterized. The hydrogen and carbon atom positions in the ^1^H-NMR and ^13^C-NMR spectra were supported by the dept-135 or COSY experiments and by 2D-NMR map analysis of the Heteronuclear Multiple-Quantum Correlation (HMQC) and Heteronuclear Multiple Bond Correlation (HMBC), Nuclear Overhauser Enhancement Spectroscopy (NOESY) if measured. See Supporting Information for detailed synthesis protocols and compounds spectroscopic characterization.

### SARS-CoV-2 PL^pro^ preparation

SARS-CoV-2 PL^pro^ was prepared as described^26^. In brief, *pGEX6P-1-SARS-CoV-2PLpro* was transformed into BL21 (DE3) codon-plus *E. coli* cells and induced with 0.1 mM IPTG and 0.1 mM ZnSO_4_ at 18 °C overnight. GST-fusion SARS-CoV-2 PL^pro^ was purified using a standard protocol. The fusion protein was cleaved using GST-PreScission protease at 4 °C overnight followed with desalting and passing through fresh glutathione beads to remove cleaved GST and GST-PreScission protease. The sample was further purified using Superdex 200 pg size-exclusion columns (GE) equilibrated with 20 mM Tris–Cl pH 8.0, 40 mM NaCl and 2 mM DTT. The peak fractions were pooled and concentrated to ~ 10 mg/mL and snap frozen in liquid nitrogen for later use.

### SARS-CoV-2 M^pro^ preparation

SARS-CoV-2 M^pro^ was recombinantly produced as described^18^. Briefly, the gene of the M^pro^ was cloned into the PGEX-6p-1 vector, which has a Nsp4-Nsp5 and a PreScission cleavage site at the N- and C-termini, respectively, to generate the authentic target protein. The gene of the target protein was expressed in the *E. coli* of the BL21-Gold (DE3) (Novagen) strain. The recombinantly produced M^pro^ was purified by employing HisTrap FF (GE Healthcare) and ion-exchange chromatography (Q FF, GE Healthcare). Finally, the high-purity target protein was subjected to a buffer exchange (20 mM Tris, 150 mM NaCl, 1 mM EDTA, 1 mM DTT, pH 7.8) for further experiments.

### SARS-CoV-2 nsp14 preparation

SARS-CoV-2 mRNA cap guanine N7-methyltransferase nsp14 was prepared as described previously^42^. Briefly, the nsp14 gene was cloned into the pET28 SUMO expression vector. Nsp14 was overexpressed in BL21 (DE3) RIL *E. coli* (Invitrogen), as a fusion protein with His-tagged SUMO. The fusion protein was purified using HisTrap FFTM column (Cytiva), followed by loading on HiTrap 26/10 Desalting column (Cytiva). To remove the N-terminal tag (6 ×His-Sumo) Sumo protease (MCLAB) was added and then the nsp14 protein again purified on HisTrap FFTM column. Flow-through fractions containing nsp14 were collected and separated from N-terminal tag (6 ×His-Sumo), and His-tagged Sumo protease. The flow-through fraction was further finally purified on a Superdex 75 pg HiLoad 26/600 gel filtration column (Cytiva). Fractions containing nsp14 were concentrated to 30 μM, flash frozen and stored at − 80 °C in a buffer containing 50 mM HEPES (pH 8.0), 100 mM NaCl, 1 mM DTT, 10% glycerol.

### Inhibitor screening

Evaluation of the compound library for inhibitors of SARS-CoV-2 PL^pro^ and SARS-CoV-2 M^pro^ was carried out in Corning 96-wells plates. For PL^pro^, 1 µL of each compound in DMSO solution was added to the wells. Next, 79 µL of enzyme preincubated for 10 min at 37 °C in assay buffer (50 mM Tris, 5 mM NaCl, 0.075% BSA, pH 7.5) was added to each well. The enzyme was incubated with the compounds at 37 °C for 30 min. Next, 20 µL Ac-LRGG-ACC substrate in assay buffer was added to the wells. Final concentrations were: 100 nM enzyme, 10 µM substrate and 1 µM tested compounds. In the assay for M^pro^, 1 µL of each compound in DMSO solution was added to the wells. Next, 79 µL of enzyme in assay buffer (50 mM Tris, 1 mM EDTA, pH 7.3)^94^ was added to each well and the plate was incubated at room temperature for 2 min. Next, 20 µL of QS1 substrate in assay buffer was added to the wells. Final concentrations were: 100 nM enzyme, 50 µM substrate, and 100 nM or 1 µM tested compounds. Measurements were carried out at 37 °C using a Molecular Devices Spectramax Gemini XPS spectrofluorometer. ACC fluorophore release was monitored for 30 min (λ_ex_ = 355 nm, λ_em_ = 460 nm). For the further analysis, the linear range of the progress curves was used. Measurements were performed at least in duplicate. Results were presented as mean values of relative enzyme inhibition (%, compared to the control measurement without inhibitor) with standard deviations. During the assays, the final DMSO concentration in the wells was < 2%.

### IC_50_ determination

To determine IC_50_, the relative activity of the investigated proteases was assessed in at least 11 different concentrations of selected inhibitors. Initial compound concentrations were found experimentally. Serial dilutions of inhibitors in assay buffers (described above) were prepared in 96-well plates (20 µL of each dilution in wells). For SARS-CoV-2 PL^pro^, 60 µL enzyme preincubated for 10 min at 37 °C in assay buffer was added to the wells. The enzyme was incubated with inhibitors for 30 min at 37 °C. Next, 20 µL substrate (Ac-LRGG-ACC) in assay buffer was added to the wells. Final concentrations were 100 nM enzyme and 10 µM substrate. For SARS-CoV-2 M^pro^, 60 µL enzyme was added with no preincubation. The enzyme was incubated with inhibitor for 2 min at room temperature. Next, 20 µL of substrate (QS1) in the assay buffer was added to the wells. Final concentrations were 100 nM for the enzyme and 50 µM for the substrate. Measurements were carried out at 37 °C using a Molecular Devices Spectramax Gemini XPS spectrofluorometer. ACC fluorophore release was monitored for 30 min (λ_ex_ = 355 nm, λ_em_ = 460 nm). IC_50_ values were determined with GraphPad Prism software using non-linear regression (dose–response—Inhibition equation) and presented as relative enzyme activity vs. inhibitor concentration. Measurements were performed at least in triplicate. Results are presented as mean values with standard deviations. During the assays, the DMSO concentration in wells was < 2%. See Supplementary Information for IC_50_ graphs.

### IC_50_ determination with nsp14 N7-MTase

To determine IC_50_ parameters of ebselen analogs towards the nsp14 enzyme, we used the previously described Py-FLINT assay designed for N7-MTase activity studies^42,67^. The Py-FLINT probe (1 μM) was incubated with SAM cosubstrate (20 μM), nsp14 (40 nM), and an inhibitor (half-log dilutions log*C*_*inh*_ < − 2.5; 2 >). Point fluorescence measurements (λ_ex_ = 345 nm, λ_em_ = 378 nm) were carried out in 96-well black, non-binding assay plates at 30 °C. Initial rates *V* were calculated by fitting a linear curve to the first 10 points (10 min). To the obtained dependences *V*(*C*_*inh*_) the following four-parameter dose–response equation was fitted:

1 $$\frac{V}{{V}_{0}}=A1+\frac{A2-A1}{1+{\left(\frac{{C}_{inh}}{{IC}_{50}}\right)}^{p}},$$

where A1 and A2 are the bottom and top asymptotes, respectively; *C*_*inh*_ the inhibitor concentration; *p* is the Hill coefficient, and *V*/*V*_0_ is the ratio of the initial reaction rate with the inhibitor to that without the inhibitor. For curve fitting and IC_50_ calculations we used GraphPad Prism software.

### Anti-SARS-CoV-2 and cytotoxicity assays in Vero E6 cells

The anti-SARS-CoV-2 activity was measured by determining the extent to which the compounds inhibited the virus-induced cytopathic effect (CPE) and reduced SARS-CoV-2 RNA in Vero E6 cells (**ECACC 85020206**). For the CPE-based assay, two-fold fold serial dilutions of compounds were added in triplicate in a 384-well plate with 5000 Vero E6 cells in DMEM medium with 2% FBS, 100 U of penicillin/mL, and 100 µg of streptomycin/mL (all Merck). After 1 h incubation, SARS-CoV-2 (strain hCoV-19/Czech Republic/NRL_6632_2/2020 was isolated in a biosafety level 3 laboratory from nasopharyngeal swab by inoculating Vero CCL81 cells [**ECACC 84113001**]) was added at multiplicity of infection 0.05 IU/mL. Following three days incubation at 37 °C in 5% CO_2_, the cell viability was determined by addition of XTT solution (Sigma-Aldrich) for 4 h and the absorbance was measured using EnVision plate reader (Perkin Elmer). Drug concentrations required to reduce viral cytopathic effect by 50% (EC_50_) were calculated using nonlinear regression from plots of percentage cell viability versus log_10_ drug concentration using GraphPad Prism v.9.0.0 Software. For RNA reduction-based assay, two-fold fold serial dilutions of compounds were added in triplicate in 96-well plate with 20,000 Vero cells plated day before in the same medium as above. After 1 h incubation, SARS-CoV-2 was added at multiplicity of infection 0.05 IU/cell. After 2 h, virus was removed and new compound was added to the cells. Cells were incubated for two days, then the medium was used as a template in RT-qPCR (Multiplex RT-PCR for COVID-19, Diana Biotechnologies, Czech Republic). Compound concentrations required to reduce SARS-CoV-2 RNA copy number by 50% (EC_50_) were calculated from plots of percentage of RNA copy number versus log_10_ drug concentration as above.

Maximal titer reduction for selected compounds was determined at EC90 concentration that was computed from EC50 determinations using equation EC90 = 9^√H^ × EC50, where H is Hill slope. Briefly, EC90 concentration of selected compounds were added in triplicate to 20,000 Vero E6 cells seeded 24 h before, incubated for 1 h and SARS-CoV-2 at MOI = 0.05 IU/mL was added. After 1 h incubation at 37 °C, 5% CO_2_ medium was removed and fresh compounds at EC90 were added and incubated for 48 h at 37 °C, 5% CO_2_. Remdesivir was included as control. Titer reduction was determined by plaque assay in Vero E6 cells. Briefly, virus supernatant was removed, tenfold serially diluted in 24-well plate followed by addition of 300,000 Vero E6 cells and 4 h incubation at 37 °C, 5% CO_2_. Then, the suspension was overlaid with 1.5% (w/v) carboxymethylcellulose in DMEM and incubated for 5 days at 37 °C, 5% CO_2_. After incubation, the cells were washed once with 1 × PBS, stained with naphthalene black for 45 min, washed with ddH_2_O and air-dried. Plaques were counted and difference between no drug control and compound virus titer was expressed as log_10_ plaque forming units (PFU) per mL.

Cytotoxicity was evaluated by incubating two-fold serial dilutions of each compound with Vero E6 cells. Following three days incubation at 37 °C in 5% CO_2_, the cell viability was determined by addition of XTT solution as above. The compound concentrations resulting in 50% reduction of absorbance (CC_50_) were calculated from plots of percentage of absorbance versus log_10_ drug concentration as above.

## Supplementary Information

Supplementary Information.

## Data Availability

The datasets used and/or analysed during the current study available from the corresponding author on reasonable request.

## Abbreviations

Abu: α-Aminobutyric acid
ACC: 7-Amino-4-carbamoylmethylcoumarin
DCM: Dichloromethane
DMF: Dimethylformamide
Et_3_N: Triethylamine
HMPTA: Hexamethylphosphoramide
MeCN: Acetonitrile
Py-FLINT: Pyrene-based fluorescence intensity
SAM: *S*-Adenosyl-l-methionine
THF: Tetrahydrofuran
Tle: *tert*-Leucine
