# Supplementary material for: Ebselen derivatives inhibit SARS-CoV-2 replication by inhibition of its essential proteins: PLpro and Mpro proteases, and nsp14 guanine N7-methyltransferase
Source: Sci Rep. 2023 Jun 6;13:9161. doi: 10.1038/s41598-023-35907-w (PMC10242237; doi:10.1038/s41598-023-35907-w)
Supplement: Supplementary file 1 — Supplementary Information. [file 41598_2023_35907_MOESM1_ESM.pdf]

Supplementary Information

Ebselen derivatives inhibit SARS-CoV-2 replication by inhibition of its essential proteins: PL^{pro} and M^{pro} proteases, and nsp14 guanine N7-methyltransferase

Mikolaj Zmudzinski[1,*] , Wioletta Rut[1], Kamila Olech[2], Jarosław Granda[2], Mirosław Giurg[2], Małgorzata Burda-Grabowska[2], Rafał Kaleta[2], Michala Zgarbova[3], Renata Kasprzyk[4,5], Linlin Zhang[6], Xinyuanyuan Sun[6], Zongyang Lv[7], Digant Nayak[7], Malgorzata Kesik-Brodacka[8], Shaun K. Olsen[7], Jan Weber[3], Rolf Hilgenfeld[6,9], Jacek Jemielity[4], Marcin Drag[1,*]

[1]Department of Chemical Biology and Bioimaging, Wroclaw University of Science and Technology, Wyb. Wyspianskiego 27, 50-370 Wroclaw, Poland

[2]Department of Organic and Medicinal Chemistry, Faculty of Chemistry, Wroclaw University of Science and Technology, Wyb. Wyspianskiego 27, 50-370 Wroclaw, Poland

[3]Institute of Organic Chemistry and Biochemistry of the Czech Academy of Sciences, Flemingovo nám. 2, 16610 Prague, Czech Republic

[4]Centre of New Technologies, University of Warsaw, Banacha 2c, 02-097 Warsaw, Poland

[5]College of Inter-Faculty Individual Studies in Mathematics and Natural Sciences, University of Warsaw, Banacha 2c, 02-097 Warsaw, Poland

[6]Institute of Molecular Medicine, University of Lübeck, Ratzeburger Allee 160, 23562 Lübeck, Germany

[7]Department of Biochemistry & Structural Biology University of Texas Health Science Center at San Antonio, San Antonio, TX, 78229 USA

[8]Research Network Łukasiewicz - Institute of Biotechnology and Antibiotics, Starościńska 5, 02-516, Warsaw, Poland

[9]German Center for Infection Research (DZIF), Hamburg-Lübeck-Borstel-Riems Site, University of Lübeck, 23562 Lübeck, Germany

[*]Corresponding author: e-mail address mikolaj.zmudzinski@pwr.edu.pl; marcin.drag@pwr.edu.pl

Content of supporting information:

1. Table S1. Characteristics of ebselen and compounds **1**–**34** S3-S5
2. Table S2. Screening of 2-phenylbenzisoselenazol-3(2*H*)-ones S6
3. Table S3. Screening of diselenides S7
4. IC_{50} graphs S8
5. Inhibition curves for anti-SARS-CoV-2 nsp14 N7-MTase activity S9-S10
6. CPE-based dose-response curves S11
7. RNA-reduction based assay dose-response curves S12
8. Cytotoxicity assay dose-response curves S13
9. Synthesis of the compounds S14-S37
10. LC-MS analysis of synthesized compounds S38-S73
11. ^{1}H, ^{13}C NMR, selected ^{19}F NMR spectra, and NMR experiments of ebselen derivatives **1–23** S74-180
12. NMR spectra of diselenides **24–34** S181-S242
13. References S243

Table S1. Characteristics of ebselen and compounds **1**–**34**.

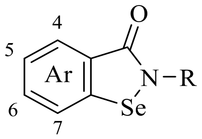

Ebselen, **1**–**6**, **8**–**23** ;

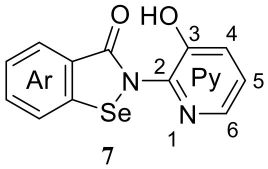

7 ;

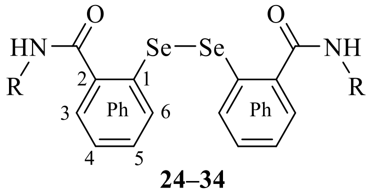

24–**34**

Compd	R	Rx time	Yield (%)	Mp (°C)	Mp lit. (°C)	Lit
**Ebs**[a]	C_6H_5	2 h	72	180–182	281.0–282.5	[1]
**1**	2-FC_6H_4	3 h	77	166–167	160–161	[2]
**2**	2-ClC_6H_4	3 h	75	200–203	194	[2]
**3**	2-BrC_6H_4	16 h	65	214–216	174–176	[3]
**4**	2-MeC_6H_4	16 h	78	191–193	–	[4]
**5**	2-$CF_3C_6H_4$	1.25 h	78	205–206	–	[5]
**6**	2-$NO_2C_6H_4$	10 days	69	242–243	–	[6]
**7**	3-HOpyridin-2-yl	2 h	66	228–229[b]	229[b]	[1]
**8**	3-$MeOC_6H_4$	1 h	66	168–169	166–168	[1]
**9**	4-$CF_3C_6H_4$	1 h	65	241–242	246–248	[7]
**10**	4-$NO_2C_6H_4$	2 h	51	286–288	280–282	[8]
**11**	4-IC_6H_4	1.5 h	88	203–204	201–202	[9]
**12**	4-AcC_6H_4	16 h	67	185–186	–	[10]
**13**	4-$AcNHC_6H_4$	4 days	67	282–283[b]	–	–

**14**	2,4-$F_2C_6H_3$	8 h	75	178–179	–	[10]
**15**	2-F-4-ClC_6H_3	3.5 h	85	220–221	–	[11]
**16**	2,4-$(MeO)_2C_6H_3$	5 h	76	238–240	239–240	[1]
**17**	2-F-5-ClC_6H_3	6 days	51	197–198	–	[4]
**18**	2,5-$Cl_2C_6H_3$	5 h	85	188–190	–	–
**19**	3-Cl-5-MeC_6H_3	1 day	63	183–184	184–185	[12]
**20**	2-Me-5-ClC_6H_3	18 h	83	190–191	189–190	[12]
**21**	2-Me-3-ClC_6H_3	3 days	63	282–283	282–283	[12]
**22**	3-Me-4-ClC_6H_3	2 h	74	208–209	209–210	[12]
**23**	3,4-$(MeO)_2C_6H_3$	2 days	73	157–159	159–161	[1]
**24**	C_6H_5	7 days	94	262–264	263–265	[5]
**25**	2-FC_6H_4	15 days	64	233–236	–	[11]
**26**	2-$CF_3C_6H_4$	19 days	78	228–229	228–229	[5]
**27**	3-FC_6H_4	7 days	92	238–240	–	[11]
**28**	3-$MeOC_6H_4$	3 days	98	221–223	221–223	[12]
**29**	4-$CF_3C_6H_4$	2 days	94	261–264	273	[5]
**30**	2-F-4-ClC_6H_3	6 days	75	232–233	–	–
**31**	2,4-$(MeO)_2C_6H_3$	7 days	98	211–214	211.0–213.5	[1]
**32**	2-Me-5-ClC_6H_3	6 days	86	248–249	247–248	[12]

**33**	3-Me-4-ClC_6H_3	6 days	85	246–248	239–240	[12]
**34**	2-F-5-ClC_6H_3	7 days	84	218–219	–	–

[a] Ebselen. [b] With decomposition.

Table S2. 2-phenylbenzisoselenazol-3(2*H*)-ones screening results for SARS-CoV-2 proteases. Assay conditions for SARS-CoV-2 PL^{pro}: [E] = 100 nM, [I] = 1 μM, [S] = 10 μM; for SARS-CoV-2 M^{pro}: [E] = 100 nM, [I] = 100 nM, [S] = 50 μM. The results show that electron-withdrawing groups (EWGs) at the *ortho* position of the phenyl ring hamper inhibition of the PL^{pro}. M^{pro} screening at [I] = 1 μM resulted with 100% inhibition of the protease by all of the compounds.

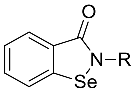

R:		inhibition [%] SARS-CoV-2 PL^{pro}	inhibition [%] SARS-CoV-2 M^{pro}	R:		inhibition [%] SARS-CoV-2 PL^{pro}	inhibition [%] SARS-CoV-2 M^{pro}
**ebselen**	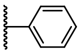	65.4 ± 2.4	57.6 ± 1.3	**12**	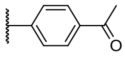	59.0 ± 3.0	55.1 ± 2.7
**1**	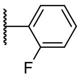	54.6 ± 2.0	72.3 ± 2.3	**13**	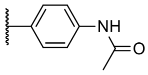	57.3 ± 1.8	62.0 ± 1.0
**2**	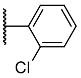	56.7 ± 4.1	66.5 ± 3.4	**14**	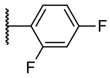	58.1 ± 3.9	64.8 ± 0.0
**3**	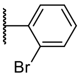	59.2 ± 4.5	73.3 ± 4.5	**15**	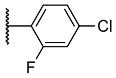	55.7 ± 3.1	77.1 ± 2.8
**4**	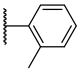	55.8 ± 5.2	63.4 ± 2.7	**16**	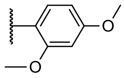	64.3 ± 3.8	59.0 ± 1.5
**5**	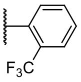	44.4 ± 2.5	62.8 ± 0.7	**17**	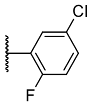	47.2 ± 1.0	**82.3 ± 2.1**
**6**	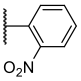	46.6 ± 2.3	38.5 ± 2.3	**18**	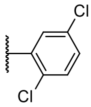	47.8 ± 0.9	72.3 ± 3.1
**7**	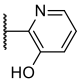	**84.4 ± 2.1**	55.9 ± 4.1	**19**	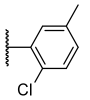	57.6 ± 3.0	61.8 ± 0.5
**8**	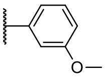	52.6 ± 1.4	59.9 ± 4.8	**20**	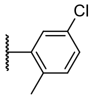	62.6 ± 1.7	69.0 ± 3.5
**9**	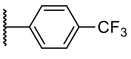	48.0 ± 3.6	71.0 ± 9.3	**21**	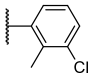	58.3 ± 2.4	70.6 ± 1.4
**10**	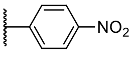	56.2 ± 4.2	**82.9 ± 1.7**	**22**	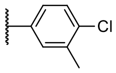	43.1 ± 2.1	72.2 ± 0.8
**11**	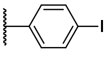	48.0 ± 2.1	71.6 ± 5.6	**23**	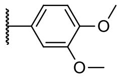	58.0 ± 1.9	60.6 ± 0.4

Table S3. Ebselen analogues diselenide derivatives screening results for SARS-CoV-2 proteases. Assay conditions for SARS-CoV-2 PL^{pro}: [E] = 100 nM, [I] = 1 μM, [S] = 10 μM; for SARS-CoV-2 M^{pro}: [E] = 100 nM, [S] = 50 μM, [I] = 100 nM and [I] = 1 μM.

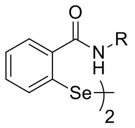

	R:	inhibition [%] SARS-CoV-2 PL^{pro}	SARS-CoV-2 M^{pro}, [I] = 100 nM	SARS-CoV-2 M^{pro}, [I] = 1 μM
	**ebselen**	65.4 ± 2.4	57.6 ± 1.3	100 ± 0.0
**24**	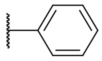	**87.4 ± 0.7**	44.7 ± 3.1	100 ± 0.0
**25**	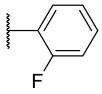	82.1 ± 2.7	33.0 ± 5.5	100 ± 0.0
**26**	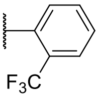	61.3 ± 2.2	34.5 ± 4.4	78.1 ± 2.4
**27**	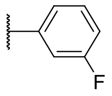	**90.1 ± 5.4**	44.0 ± 4.1	100 ± 0.0
**28**	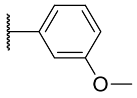	81.9 ± 0.8	37.0 ± 4.3	100 ± 0.0
**29**	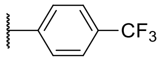	61.3 ± 3.4	14.0 ± 2.4	100 ± 0.0
**30**	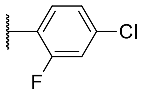	75.1 ± 0.4	27.4 ± 0.0	49.6 ± 2.7
**31**	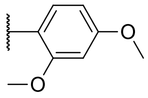	76.6 ± 2.4	50.0 ± 1.0	100 ± 0.0
**32**	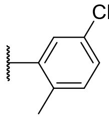	72.4 ± 1.7	55.4 ± 3.8	100 ± 0.0
**33**	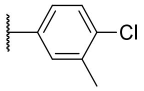	72.1 ± 3.2	48.7 ± 5.4	92.0 ± 0.3
**34**	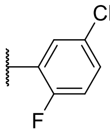	60.2 ± 1.7	32.0 ± 2.1	43.1 ± 3.0

SARS-CoV-2 PL^{pro}

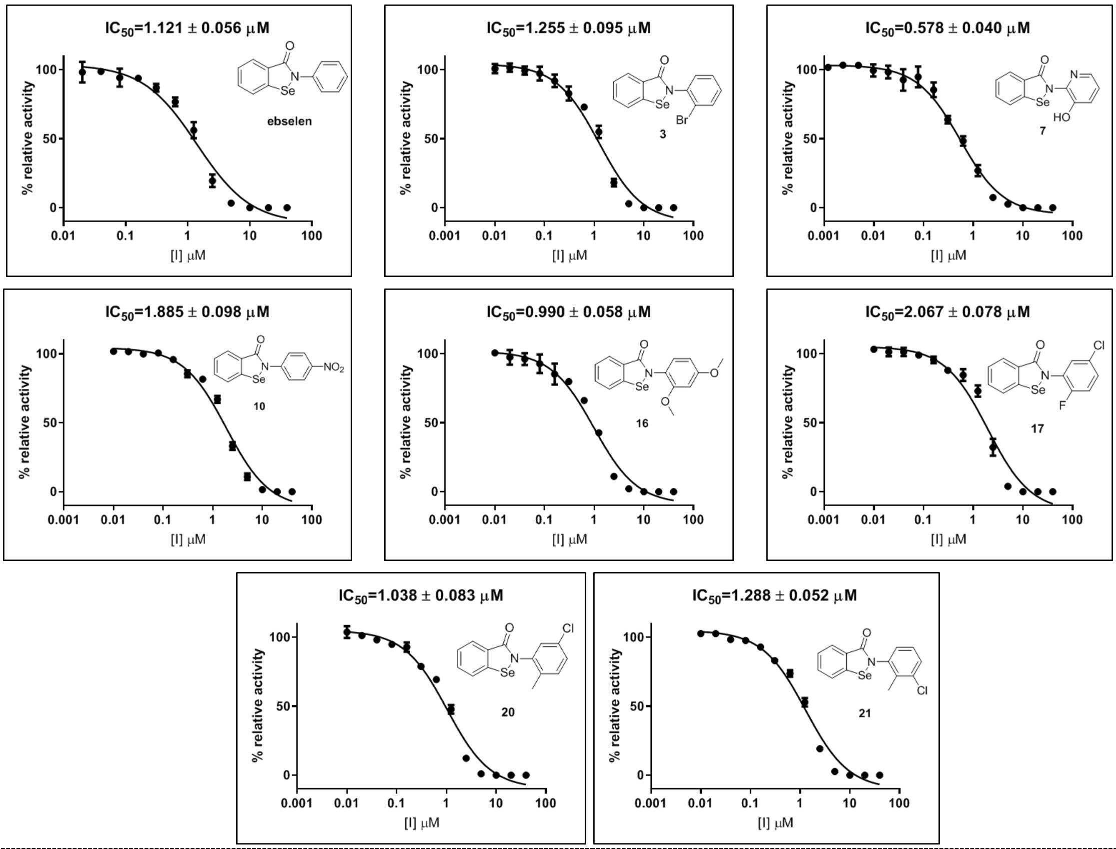

SARS-CoV-2 M^{pro}

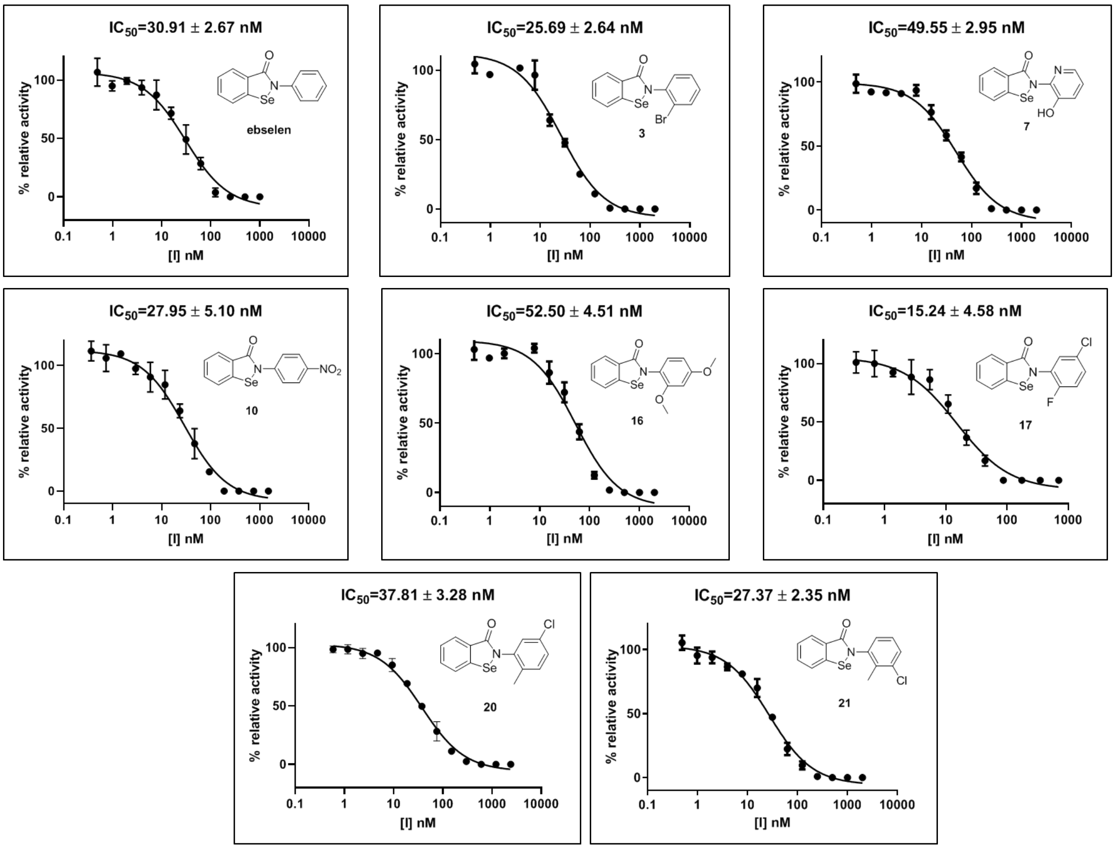

Figure S1. IC_{50} values of SARS-CoV-2 M^{pro} and PL^{pro} inhibitors.

SARS-CoV-2 nsp14

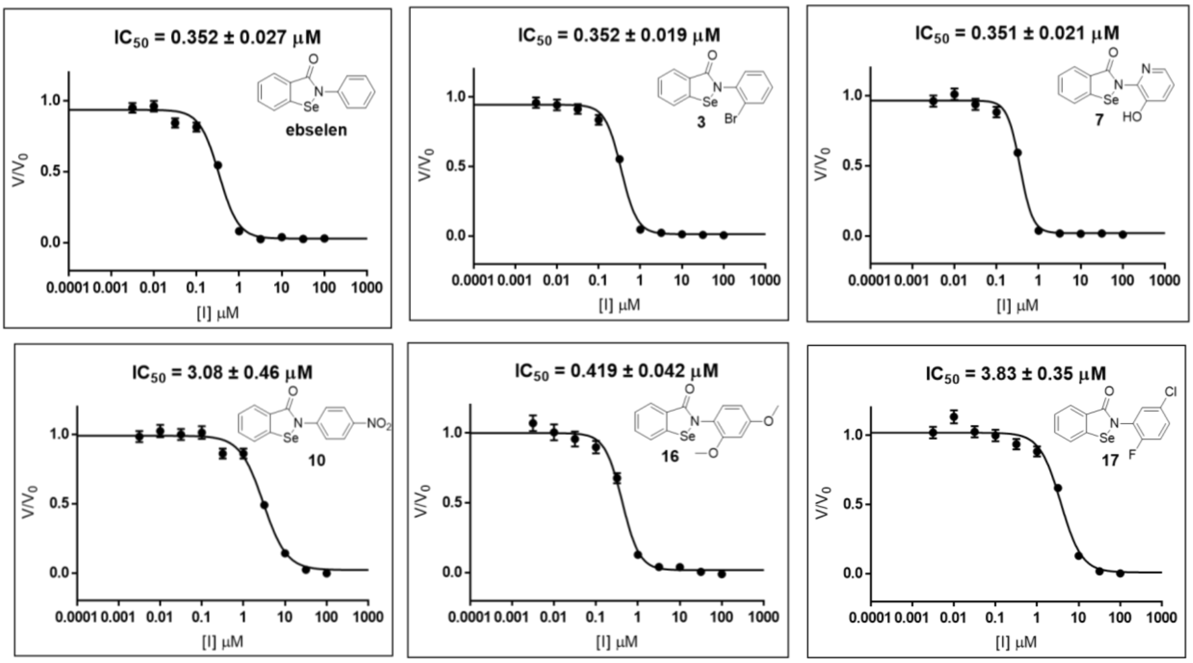

Figure S2. Inhibition curves of ebselen analogs against SARS-CoV-2 nsp14 N7-MTase.

SARS-CoV-2 nsp14

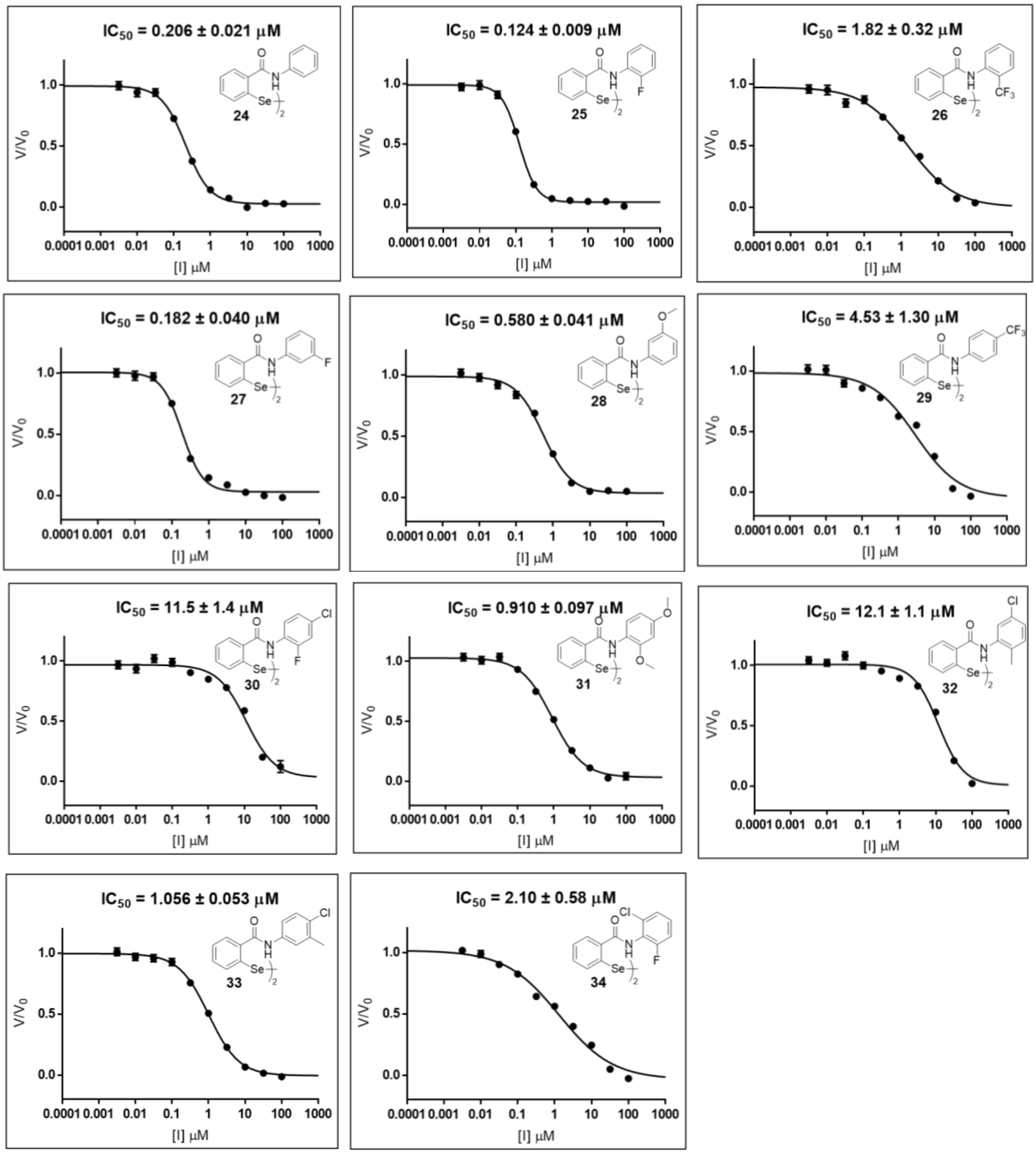

Figure S3. Inhibition curves of ebselen diselenides against SARS-CoV-2 nsp14 N7-MTase.

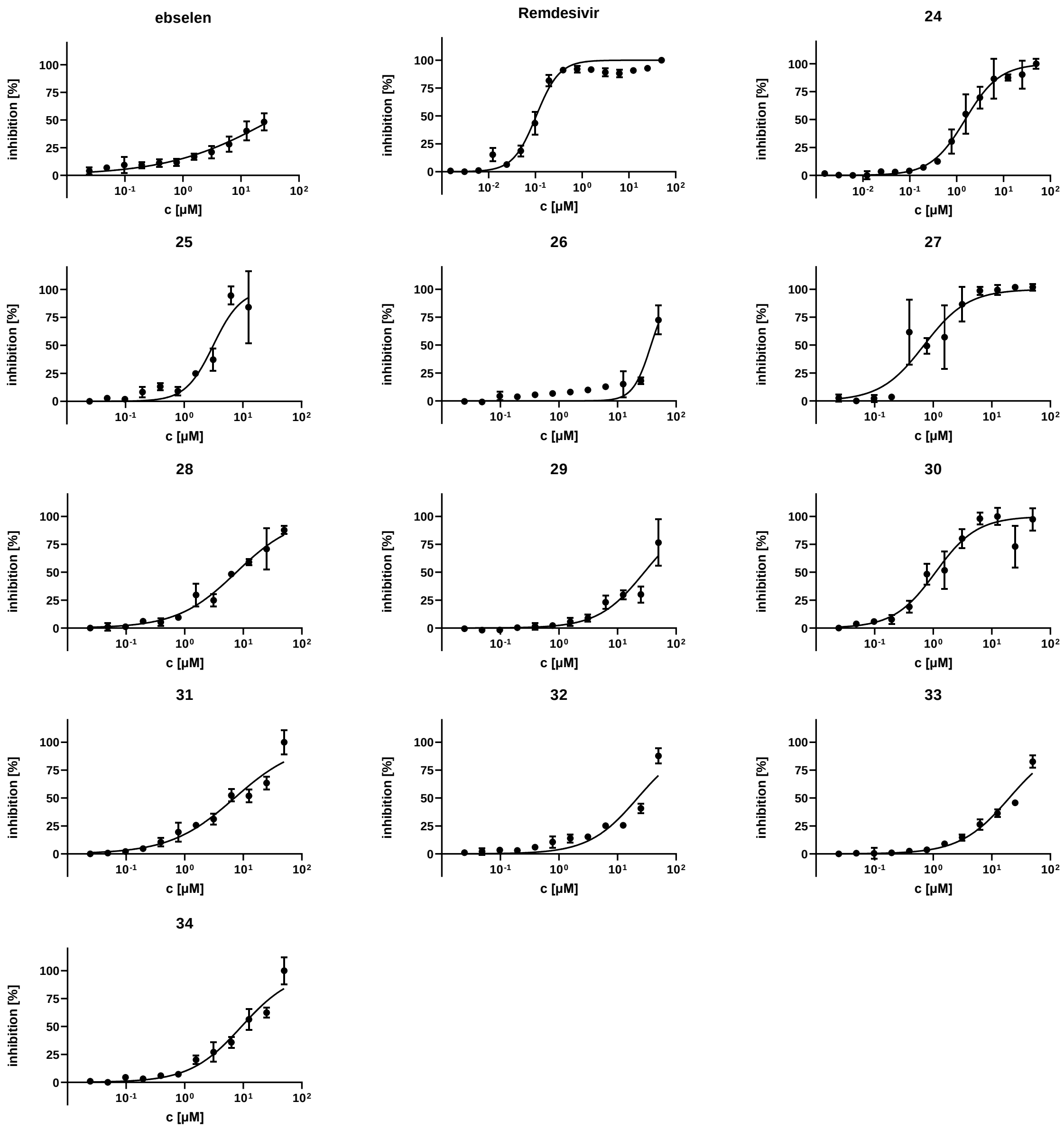

Figure S4. Dose response curve analysis of anti-SARS-CoV-2 activity of diselenides from cytopathic effect-based experiment.

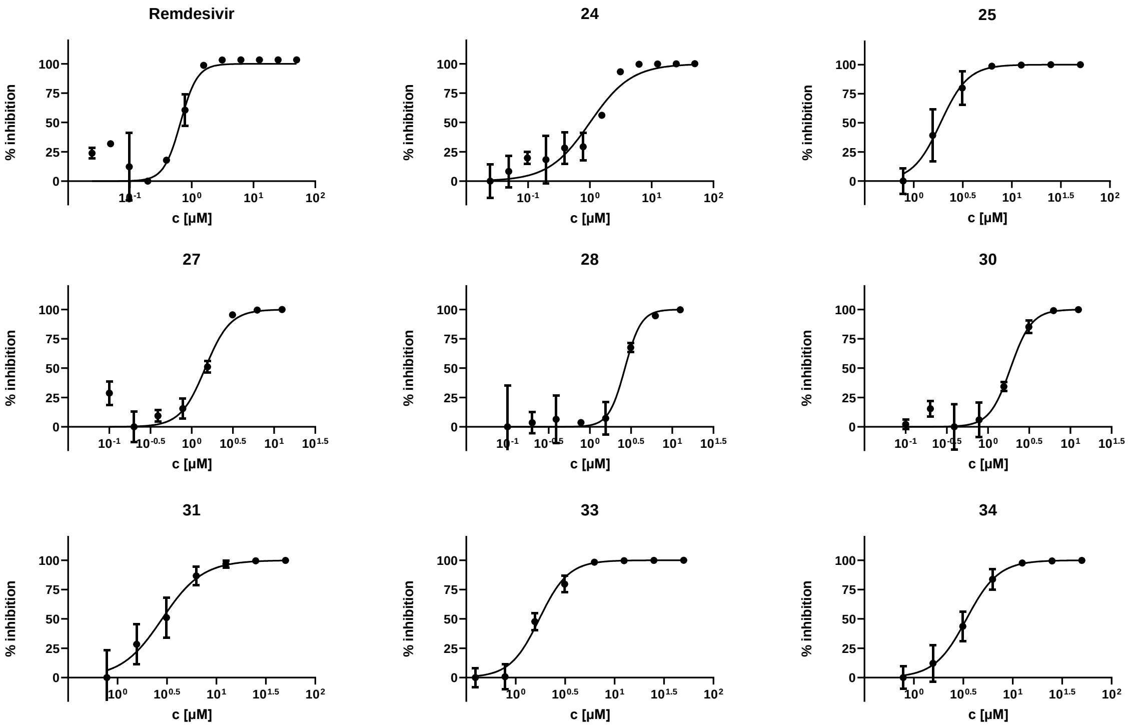

Figure S5. Dose response curve analysis of anti-SARS-CoV-2 activity of selected diselenides from RNA reduction-based experiment.

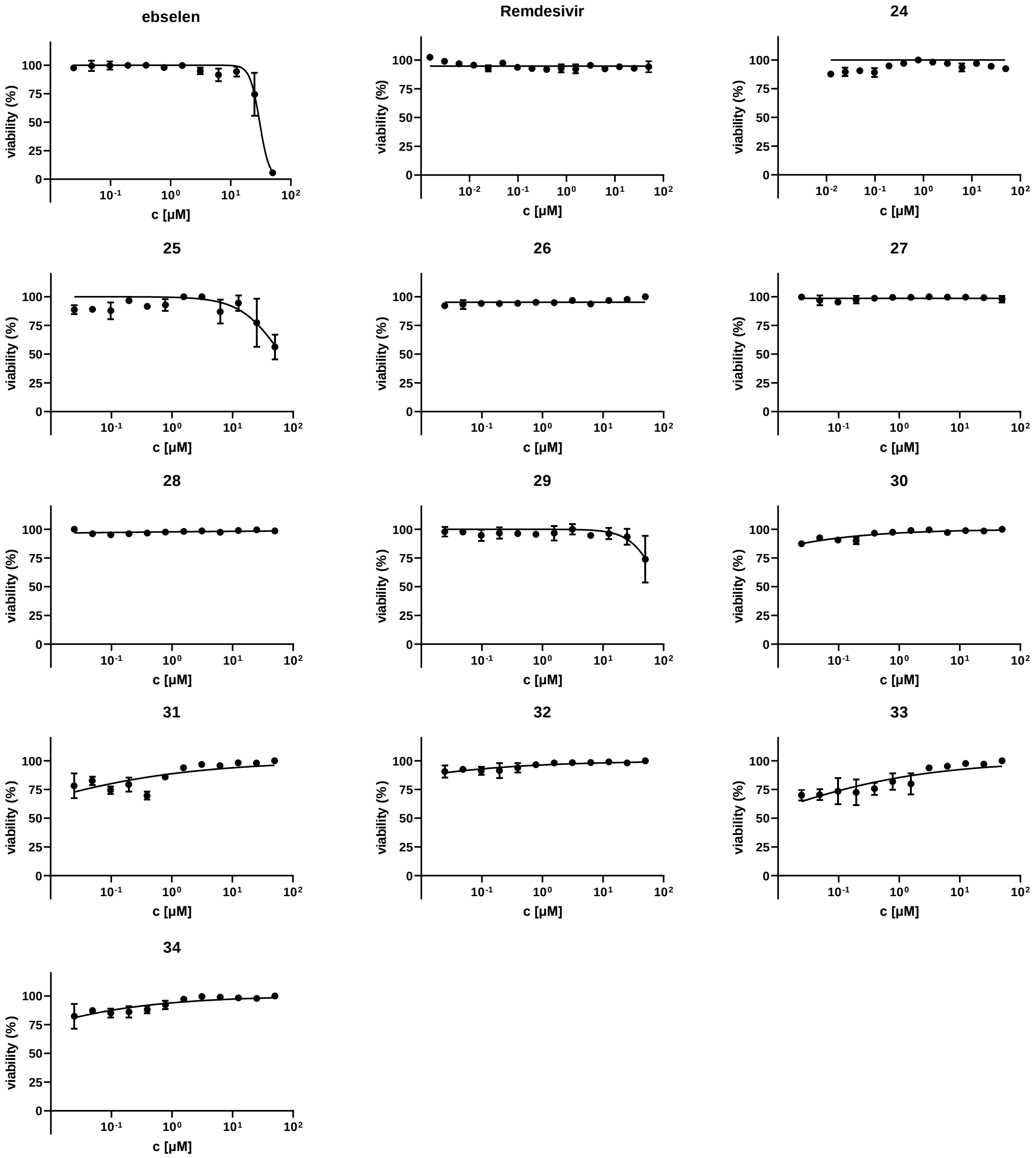

Figure S6. Dose response curve analysis of cytotoxicity of diselenides.

Synthesis of bezisolenazol-3(2H)-ones: ebselen and compounds 1–23

2-Phenyl-1,2-benzisoselenazol-3(2*H*)-one (**ebselen**) [13]

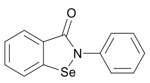

The general procedure starting from aniline (0.455g, 5.0 mmol) was employed with a 2-h reaction time and 2-h standing before filtration to obtain ebselen which was recrystallized from EtOAc to afford 2-phenyl-1,2-benzisoselenazol-3(2*H*)-one (1.00g, 3.65 mmol) as dark orange prisms with 72% yield, which melted at 180–182°C (from EtOAc) (m.p. 181.0–181.5°C [1]). Selected FT-IR (ATR): ν_{max} 3091 (C_{Ar}-H), 3001 (C_{Ar}-H), 1585 (C=O), 1561, 1486, 1444, 1310 (C_{Ar}-N), 1263, 1135, 1028, 946, 755, 735, 684, 600, 509, 479 cm^{-1}; ^{1}H-NMR (300.1 MHz, $CDCl_3$): δ 8.12 (ddd, ^{3}J = 7.8 Hz, ^{4}J = 1.3 Hz, ^{5}J = 0.7 Hz, 1H), 7.60–7.69 (m, 4H), 7.40–8.50 (m, 3H), 7.28 (tt, ^{3}J = 7.4 Hz, ^{4}J = 1.2 Hz, 1H) ppm; HRMS (TOF MS ESI): *m/z* for $C_{13}H_9NOSe + Na^+$ calculated: 297.9742; found: 297.9745. ^{1}H-NMR spectrum is in agreement with the literature data [14, 15].

2-(2-Fluorophenyl)-1,2-benzisoselenazol-3(2*H*)-one (**1**)

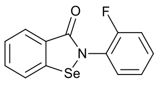

The general procedure starting from 2-fluoroaniline (0.556g, 5.0 mmol) was employed with a 3 h reaction time to obtain 2-(2-fluorophenyl)-1,2-benzisoselenazol-3(2*H*)-one (**1**) (1.13g, 3.87 mmol) as yellow crystals with 77% yield. The analytically pure sample was obtained by recrystallization from EtOAc to obtain **1** (0.949 g, 3.25 mmol) as a yellow solid with 65% yield which melted at 163–164°C (from EtOAc) (m.p. 160–161°C [2]). Selected FT-IR (KBr): ν_{max} 3086 (C_{Ar}-H), 3063 (C_{Ar}-H), 1593 (C=O), 1578, 1561, 1497, 1443, 1338 (C_{Ar}-N), 1227, 1210, 1141, 1025, 933, 791, 754, 739 (C-Se), 677, 555, 463 cm^{-1}; ^{1}H-NMR (399.8 MHz, DMSO-d_6): δ 8.10 (d, ^{3}J = 8.0 Hz, 1H, ArH-7), 7.91 (dd, ^{3}J = 7.7 Hz, ^{4}J = 1.5 Hz, 1H, ArH-4), 7.70 (ddd, $^{4}J_{HF}$ = 8.0 Hz, ^{3}J = 7.2 Hz, ^{4}J = 1.5 Hz, 1H, ArH-6), 7.51 (ddd, ^{3}J = 7.7 Hz, ^{3}J = 7.2 Hz, ^{4}J = 1.7 Hz, 1H, ArH-5), 7.49 (ddd, $^{4}J_{HF}$ = 8.0 Hz, ^{3}J = 7.2 Hz, ^{4}J = 1.4 Hz, 1H, PhH-6), 7.45 (dddd, ^{3}J = 9.3 Hz, ^{3}J = 7.9 Hz, $^{4}J_{HF}$ = 5.3 Hz, ^{4}J = 1.6 Hz, 1H, PhH-4), 7.38 (ddd, $^{3}J_{HF}$ = 10.2 Hz, ^{3}J = 7.3 Hz, ^{4}J = 1.5 Hz, 1H, PhH-3), 7.30 (ddd, ^{3}J = 7.9 Hz, ^{3}J = 7.2 Hz, ^{4}J = 1.5 Hz, 1H, PhH-5) ppm; ^{13}C-NMR (75.48 MHz, DMSO-d_6): δ 165.56 (C=O), 157.60 (d, $^{1}J_{CF}$ = 250.0 Hz, CF), 140.27 (C), 132.24 (CH), 130.17 (CH), 129.43 (d, $^{3}J_{CF}$ = 7.9 Hz, CH), 127.92 (CH), 126.83 (C), 126.27 (d, $^{2}J_{CF}$ = 17.0 Hz, PhC-1), 126.16 (CH), 126.00 (CH), 124.86 (d, $^{4}J_{CF}$ = 3.5 Hz, CH), 116.47 (d, $^{2}J_{CF}$ = 19.8 Hz, PhC-3) ppm; ^{13}C-NMR (100.5 MHz, DMSO-d_6): δ 165.57 (C=O), 157.61 (d, $^{1}J_{CF}$ = 250.0 Hz, CF), 140.28 (CSe),

132.27 (ArC-6), 130.18 (PhC-6), 129.47 (d, $^{3}J_{CF}$ = 7.8 Hz, PhC-4), 127.93 (ArC-4), 126.83 (ArC-3'), 126.28 (d, $^{2}J_{CF}$ = 17.3 Hz, PhC-1), 126.19 (ArC-5), 126.01 (ArC-7), 124.89 (d, $^{4}J_{CF}$ = 3.8 Hz, PhC-5), 116.50 (d, $^{2}J_{CF}$ = 19.8 Hz, PhC-3) ppm; ^{19}F-NMR (376.2 MHz, DMSO-d_6): δ –118.63 (ddd, $^{3}J_{FH}$ = 10.2 Hz, $^{4}J_{FH}$ = 8.0 Hz, $^{4}J_{FH}$ = 5.3 Hz) ppm; ^{77}Se-NMR (76.24 MHz, DMSO-d_6): δ 940.51 (d, $^{4}J_{SeF}$ = 18.2 Hz) ppm; HRMS (TOF MS ESI): *m/z* for $C_{13}H_8FNOSe$ + H^+ calculated: 293.9828; found: 293.9838. ^{1}H-NMR and ^{13}C-NMR spectra are in agreement with the literature data [2].

2-(2-Chlorophenyl)-1,2-benzisoselenazol-3(2*H*)-one (**2**)

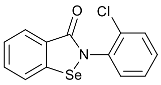

The general procedure starting from 2-chloroaniline (0.638 g, 5.0 mmol) was employed with a 3-h reaction time to obtain 2-(2-chlorophenyl)-1,2-benzisoselenazol-3(2*H*)-one (**2**) (1.16g, 3.76 mmol) as a pale yellow crystals with 75% yield, which melted at 200–203°C (m.p. 194°C [2]). Selected FT-IR (ATR): ν_{max} 3078 (C_{Ar}-H), 3051 (C_{Ar}-H), 1602, 1584 (C=O), 1473, 1439, 1342 (C_{Ar}-N), 1307, 1139, 1062, 1019, 760, 730 (C-Se), 672, 606, 504, 456 cm^{-1}; ^{1}H-NMR (600.6 MHz, DMSO-d_6): δ 8.10 (d, ^{3}J = 8.0 Hz, 1H, ArH-7), 7.90 (dd, ^{3}J = 7.7 Hz, ^{4}J = 0.9 Hz, 1H, ArH-4), 7.70 (ddd, ^{3}J = 8.0 Hz, ^{3}J = 7.3 Hz, ^{4}J = 1.3 Hz, 1H, ArH-6), 7.60–7.64 (m, 1H, PhH), 7.49–7.53 (m, 1H, PhH), 7.49 (ddd, ^{3}J = 7.7 Hz, ^{3}J = 7.3 Hz, ^{4}J = 0.9 Hz, 1H, ArH-5), 7.43–7.47 (m, 2H, PhH) ppm; ^{13}C-NMR (100.5 MHz, DMSO-d_6): δ 165.59 (C=O), 140.35 (CSe), 136.15 (PhC-1), 132.53 (CCl), 132.21 (ArC-6), 131.12 (PhCH), 130.12 (PhCH), 129.75 (PhCH), 128.05 (PhCH), 127.94 (ArC-4), 126.80 (ArC-3'), 126.12 (ArC-5), 125.99 (ArC-7) ppm; ^{77}Se-NMR (76.24 MHz, DMSO-d_6): δ 936.61 ppm; HRMS (TOF MS ESI): *m/z* for $C_{13}H_8ClNOSe$ + Na^+ calculated: 331.9352; found: 331.9363. ^{1}H-NMR and ^{13}C-NMR spectra are in agreement with the literature data [2].

2-(2-Bromophenyl)-1,2-benzisoselenazol-3(2*H*)-one (3)

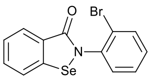

The general procedure starting from 2-bromoaniline (0.860 g, 5.0 mmol) was employed with a 16 h to obtain 2-(2-bromophenyl)-1,2-benzisoselenazol-3(2*H*)-one (**3**) (1.15g, 3.26 mmol) as pale yellow prisms with 65% yield, which melted at 214–216°C (m.p. 174–176°C [3]). Selected FT-IR (ATR): ν_{max} 3063 (C_{Ar}-H), 3049 (C_{Ar}-H), 1601 (C=O), 1587, 1563, 1467, 1437, 1342 (C_{Ar}-N), 1306, 1138, 1044, 1020, 964, 850, 783, 761, 730 (CSe), 671, 605, 507, 494, 450, 411 cm^{-1}; ^{1}H-NMR[56] (600.6 MHz, DMSO-d_6): δ 8.10 (d, ^{3}J = 8.0 Hz, 1H, ArH-7), 7.91 (ddd, ^{3}J = 7.7 Hz, ^{4}J = 1.4 Hz, ^{5}J = 0.6 Hz, 1H, ArH-4), 7.77 (dd, ^{3}J = 8.1 Hz,

^4J = 1.0 Hz, 1H, PhH-3), 7.69 (ddd, ^3J = 8.1 Hz, ^3J = 7.2 Hz, ^4J = 1.4 Hz, 1H, ArH-6), 7.47–7.51 (m, 3H, PhH-5, PhH-6, ArH-5), 7.37 (ddd, ^3J = 8.1 Hz, ^3J = 6.3 Hz, ^4J = 2.8 Hz, 1H, PhH-4) ppm; ^{13}C-NMR (100.5 MHz, DMSO-d_6): δ 165.50 (C=O), 140.31 (CSe), 137.75 (PhC-1), 133.22 (PhC-3), 132.18 (ArC-6), 131.22 (PhC-6), 130.02 (PhC-4), 128.64 (PhC-5), 127.95 (ArC-4), 126.88 (ArC-3'), 126.08 (ArC-5), 125.96 (ArC-7), 123.23 (C-Br) ppm; ^{77}Se-NMR (76.24 MHz, DMSO-d_6): δ 935.53 ppm; HRMS (TOF MS ESI): *m/z* for $C_{13}H_8BrNOSe + H^+$ calculated: 353.9027; found: 353.9026. The FT-IR spectrum is in agreement with the literature data [3].

2-(2-Methylphenyl)-1,2-benzisoselenazol-3(2*H*)-one (**4**) [14]

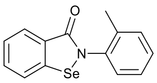

The general procedure starting from 2-methylaniline (0.536 g, 5.0 mmol) was employed with a 16-h reaction time to obtain compound **4** (1.12 g, 3.89 mmol) as yellow prisms with 78% yield. The analytically pure sample was obtained by recrystallization from MeCN:H_2O (2:1, *v*/*v*, 55 mL/g), before decolorization with charcoal, to obtain 2-(2-metylphenyl)-1,2-benzisoselenazol-3(2*H*)-one (**4**) (0.894g, 3.10 mmol) as pale yellow prisms with 62% yield, which melted at 189–191°C. Selected FT-IR (KBr): ν_{max} 3061 (C_{Ar}-H), 3050 (C_{Ar}-H), 2941 (CH_3), 2914 (CH_3), 1587 (C=O), 1563, 1486, 1440, 1344, 1306, 1108, 1018, 762, 731 (C-Se), 673, 611, 509, 487, 453, 413 cm^{-1}; ^{1}H-NMR (600.6 MHz, $CDCl_3$): δ 8.13 (d, ^3J = 7.8 Hz, 1H, ArH-4), 7.71 (d, ^3J = 8.0 Hz, 1H, ArH-7), 7.63 (ddd, ^3J = 8.0 Hz, ^3J = 7.2 Hz, ^4J = 1.3 Hz, 1H, ArH-6), 7.47 (ddd, ^3J = 7.8 Hz, ^3J = 7.2 Hz, ^4J = 0.9 Hz, 1H, ArH-5), 7.31 (d, ^3J = 7.5 Hz, 1H, PhH-3), 7.28–7.30 (m, 2H, PhH-4, PhH-6), 7.22–7.27 (m, 1H, PhH-5), 2.24 (s, 3H, CH_3) ppm; ^{1}H-NMR (399.8 MHz, DMSO-d_6): δ 8.10 (d, ^3J = 8.1 Hz, 1H, ArH-7), 7.90 (dd, ^3J = 7.8 Hz, ^4J = 1.2 Hz, 1H, ArH-4), 7.68 (ddd, ^3J = 8.1 Hz, ^3J = 7.2 Hz, ^4J = 1.2 Hz, 1H, ArH-6), 7.48 (ddd, ^3J = 7.8 Hz, ^3J = 7.2 Hz, ^4J = 1.0 Hz, 1H, ArH-5), 7.30–7.27 (m, 4H, PhH-3–6), 2.11 (s, 3H, CH_3) ppm; ^{13}C-NMR (100.5 MHz, $CDCl_3$): δ 166.08 (C=O), 139.02 (CSe), 137.54 (PhC-1), 136.68 (PhC-2), 132.27 (ArC-6), 131.14 (PhC-3), 129.29 (ArC-4), 129.04 (PhC-4 or PhC-6), 128.98 (PhC-4 or PhC-6), 126.78 (PhC-5), 126.43 (ArC-3'), 126.32 (ArC-5), 124.08 (ArC-7), 18.13 (CH_3) ppm; ^{13}C-NMR (100.5 MHz, DMSO-d_6): δ 164.04 (C=O), 140.12 (CSe), 137.37 (PhC-2), 136.55 (PhC-1), 131.93 (ArC-6), 130.73 (PhC-3), 128.73 (PhC-4 or PhC-6), 128.13 (PhC-4 or PhC-6), 127.87 (ArC-4), 127.27 (ArC-3'), 126.67 (PhC-5), 126.08 (ArC-5), 125.92 (ArC-7), 17.79 (CH_3) ppm; ^{77}Se-NMR (76.24 MHz, $CDCl_3$): δ 963.01 ppm; ^{77}Se-NMR (76.24 MHz, DMSO-d_6): δ 917.69 ppm. In the case of the interpretation of the proton spectrum and carbon chemical shifts, discrepancies in the spectroscopic data measured at $CDCl_3$ were

found [16]. In particular, the triplets (t) recorded at 7.74 ppm, 7.70 ppm and 7.53 ppm and the second signal at 139.1 ppm (C) were absent in our spectra in ^{1}H NMR and ^{13}C NMR spectroscopy, while we observed a doublet (d) or twice, doublet doublet of doublets (ddd) and resonance from ArC-5 (CH) at 126.32 ppm, respectively, at both cases measured in $CDCl_3$. HRMS (TOF, MS, ESI) m/z for $C_{14}H_{11}NOSe + H^+$ calculated: 290.0079; found: 290.0094. Anal. Calcd. for $C_{14}H_{11}NOSe$ (288.22): C, 58.34; H, 3.85; N, 4.86. Found: C, 58.42; H, 3.86; N, 4.93.

2-(2-Trifluoromethylphenyl)-1,2-benzisoselenazol-3(2*H*)-one (**5**) [5]

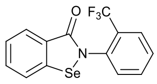

The general procedure starting from 2-trifluoromethylaniline (0.806 g, 5.0 mmol) was employed with a 1.25-h reaction time to obtain product **5** (1.33 g, 3.89 mmol) as a yellow solid with 78% yield. The analytically pure sample was obtained by recrystallization from EtOAc (12 mL/g) to obtain 2-(2-trifluoromethylphenyl)-1,2-benzisoselenazol-3(2*H*)-one (**5**) (1.03g, 3.01 mmol) as pale-yellow crystals with 60% yield, which melted at 205–206°C (from EtOAc). Selected FT-IR (KBr): ν_{max} 3095 (C_{Ar}-H), 3058 (C_{Ar}-H), 3016 (C_{Ar}-H), 1615 (br, C=O), 1457, 1443, 1345, 1316 (C-N), 1258, 1167, 1134, 1057, 1034, 772, 733 (C-Se), 676, 649, 510 cm^{-1}; ^{1}H-NMR (600.6 MHz, DMSO-d_6): δ 8.10 (dd, ^{3}J = 8.1 Hz, ^{4}J = 0.9, 1H, ArH-7), 7.89 (dd, ^{3}J = 7.7 Hz, ^{4}J = 1.1 Hz, 1H, ArH-4), 7.86 (dd, ^{3}J = 7.9 Hz, ^{4}J = 0.8 Hz, 1H, PhH-3), 7.79 (ddd, ^{3}J = 7.8 Hz, ^{3}J = 7.5 Hz, ^{4}J = 0.8 Hz, 1H, PhH-5), 7.70 (ddd, ^{3}J = 8.1 Hz, ^{3}J = 7.2 Hz, ^{4}J = 1.1 Hz, 1H, ArH-6), 7.67 (dd, ^{3}J = 7.0 Hz, ^{3}J = 7.5 Hz, 1H, PhH-4), 7.53 (d, ^{3}J = 7.8 Hz, 1H, PhH-6), 7.49 (ddd, ^{3}J = 7.7 Hz, ^{3}J = 7.2 Hz, ^{4}J = 0.9 Hz, 1H, ArH-5) ppm; ^{13}C-NMR (151.0 MHz, DMSO-d_6): δ 166.78 (C=O), 140.36 (CSe), 136.91 (k, $^{3}J_{CF}$ = 1.7 Hz, PhC-1), 133.56 (PhC-5), 132.40 (PhC-6), 132.15 (ArC-6), 128.92 (PhC-4), 128.33 (k, $^{2}J_{CF}$ = 30.2 Hz, PhC-2), 127.80 (ArC-4), 127.04 (k, $^{3}J_{CF}$ = 4.9 Hz, PhC-3), 126.26 (ArC-3'), 126.09 (ArC-5), 125.74 (ArC-7), 123.20 (k, $^{1}J_{CF}$ = 273.8 Hz, CF_3) ppm; ^{77}Se-NMR (76.24 MHz, DMSO-d_6): δ 974.13 (k, $^{5}J_{SeF}$ = 10.7 Hz) ppm; ^{19}F-NMR (376.2 MHz, DMSO-d_6): δ –59.23 ppm; HRMS (TOF, MS, ESI) m/z for $C_{14}H_8F_3NOSe + H^+$ calculated: 343.9796; found: 343.9797; Anal. Calcd. for $C_{14}H_8F_3NOSe$ (342.19): C, 49.14; H, 2.36; N, 4.09. Found: C, 49.00; H, 2.41; N, 4.16.

2-(2-Nitrophenyl)-1,2-benzisoselenazol-3(2*H*)-one (**6**) [6]

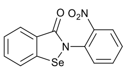

The general procedure starting from 2-nitroaniline (0.691 g, 5.0 mmol) was employed with a 10-days reaction time to obtain a dark red product. The mixture was further acidified with HCl to pH 1 with stirring overnight at room temperature to obtain 2-(2-nitrophenyl)-1,2-benzisoselenazol-3(2*H*)-one (**6**) (1.10 g, 3.45 mmol) as a pale wool with 69% yield, which melted at 239–242°C. The analytically pure sample was obtained by column chromatography with silica gel (70–230 mesh) using chloroform as an eluent, and with wash with chloroform to obtain **6** (0.321g, 1.01 mmol) as a pale orange solid with 20% yield, which melted at 242–243°C (from $CHCl_3$). Selected FT-IR (ATR): ν_{max} 3085 (C_{Ar}-H), 3062 (C_{Ar}-H), 1649 (C=O), 1591, 1526 (NO_2), 1476, 1446, 1359 (NO_2), 1332 (C-N), 1298, 1121, 955, 835, 778, 737 (C-Se), 669, 607, 506, 484 cm^{-1}; ^{1}H-NMR (399.8 MHz, DMSO-d_6): δ 8.12 (d, ^{3}J = 8.1 Hz, 1H, ArH-7), 8.04 (dd, ^{3}J = 8.1 Hz, ^{4}J = 1.4 Hz, 1H, PhH-3), 7.87 (dd, ^{3}J = 7.7 Hz, ^{4}J = 1.2 Hz, 1H, ArH-4), 7.82 (ddd, ^{3}J = 8.0 Hz, ^{3}J = 7.5 Hz, ^{4}J = 1.4 Hz, 1H, PhH-5), 7.72 (ddd, ^{3}J = 8.1 Hz, ^{3}J = 7.2 Hz, ^{4}J = 1.2 Hz, 1H, ArH-6), 7.64 (dd, ^{3}J = 8.0 Hz, ^{4}J = 1.3 Hz, 1H, PhH-6), 7.59 (ddd, ^{3}J = 8.1 Hz, ^{3}J = 7.5 Hz, ^{4}J = 1.3 Hz, 1H, PhH-4), 7.49 (ddd, ^{3}J = 7.7 Hz, ^{3}J = 7.2 Hz, ^{4}J = 0.9 Hz, 1H, ArH-5) ppm; ^{13}C-NMR (100.5 MHz, DMSO-d_6): δ 165.36 (C=O), 146.21 (PhC-2), 140.25 (CSe), 134.23 (PhC-5), 132.58 (ArC-6), 131.59 (PhC-1), 129.71 (PhC-6), 128.04 (PhC-4 or ArC-4), 127.97 (PhC-4 or ArC-4), 126.76 (ArC-3'), 126.31 (ArC-5), 126.07 (ArC-7), 125.22 (PhC-3) ppm; ^{77}Se-NMR (76.24 MHz, DMSO-d_6): δ 947.90 ppm; HRMS (TOF, MS, ESI) m/z for $C_{13}H_8N_2O_3Se + H^+$ calculated: 320.9773; found: 320.9776.

2-(3-Hydroxypyridin-2-yl)-1,2-benzisoselenazol-3(2*H*)-one (**7**)

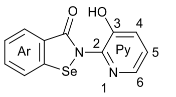

The general procedure starting from 2-amino-3-hydroxypyridine (0.551 g, 5.0 mmol) was employed with a 2-h reaction time, without acidification step, to obtain 2-(3-hydroxypyridin-2-yl)-1,2-benzisoselenazol-3(2*H*)-one (**7**) (0.960 g, 3.30 mmol) as a pale yellow powder with 66% yield, which melted at 228–229°C with decomposition (m.p. 229°C with decomposition [1]). Selected FT-IR (ATR): ν_{max} 3077 (C_{Ar}-H), 3058 (C_{Ar}-H), 2200–2650 (br., OH), 1607, 1583 (C=O), 1539, 1444, 1398, 1349 (C-N), 1294, 1245, 1107, 1021, 955, 892, 871, 842, 797, 786, 735 (C-Se), 672, 620, 505, 422 cm^{-1}; ^{1}H-NMR (399.8 MHz, DMSO-d_6): δ 12.20 (s, 1H, OH), 8.09 (d, ^{3}J = 8.1 Hz, 1H, ArH-7), 7.97 (dd, ^{3}J = 4.6 Hz, ^{4}J = 1.4 Hz, 1H, PyH-6), 7.94 (d, ^{3}J = 7.8 Hz, 1H, ArH-4), 7.71 (ddd, ^{3}J = 8.1 Hz, ^{3}J = 7.2 Hz, ^{4}J = 1.3 Hz, 1H, ArH-6), 7.48 (ddd, ^{3}J = 7.8 Hz, ^{3}J = 7.2 Hz, ^{4}J = 1.0 Hz, 1H, ArH-5), 7.37 (dd, ^{3}J = 8.0 Hz, ^{4}J = 1.4 Hz, 1H, PyH-4), 7.24 (dd, ^{3}J = 8.0 Hz, ^{3}J = 4.6 Hz, 1H, PyH-5) ppm; ^{13}C-NMR (100.5 MHz, DMSO-d_6): δ 165.07 (C=O), 144.58 (C-O), 142.32 (CSe), 140.33 (PyC-2),

138.37 (PyC-6), 132.95 (ArC-6), 129.24 (ArC-3’), 128.03 (ArC-4), 127.09 (PyC-4), 126.24 (ArC-5), 125.71 (ArC-7), 123.22 (PyC-5) ppm; ^{77}Se-NMR (76.24 MHz, DMSO-d_6): δ 963.13 ppm; HRMS (TOF, MS, ESI) m/z for C12H8N2O2Se + H+ calculated: 292.9824; found: 292.9825. ^{1}H-NMR and ^{13}C-NMR spectra agree with the literature data [1, 17].

2-(3-Methoxyphenyl)-1,2-benzisoselenazol-3(2*H*)-one (**8**)

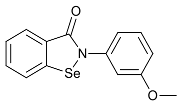

The general procedure starting from 3-methoxyaniline (0.616 g, 5.0 mmol) was employed with a 1-h reaction time with crystallization in the refrigerator without solvent removed, with washing the product with MeCN to obtain 2-(3-methoxyphenyl)-1,2-benzisoselenazol-3(2*H*)-one (**8**) (0.998 g, 3.28 mmol) as yellow crystals with 66% yield, which melted at 167–168°C (from a mixture of MeCN/H_2O, 1/1, *v/v*) (m.p. 166–168°C [1]). Selected FT-IR (ATR): ν_{max} 3097 (C_{Ar}-H), 3056 (C_{Ar}-H), 3003 (C_{Ar}-H), 2938 (CH_3), 2838 (CH_3), 1586 (C=O), 1479, 1444, 1433, 1344 (C-N), 1314, 1300, 1285, 1272, 1231 (C-O), 1171, 1049 (C-O), 871, 762, 738, 731 (C-Se), 674, 516, 555 cm^{-1}; ^{1}H-NMR (600.6 MHz, DMSO-d_6): δ 8.08 (d, ^3J = 8.0 Hz, 1H, ArH-7), 7.91 (dd, ^3J = 7.7 Hz, ^4J = 0.9 Hz, 1H, ArH-4), 7.68 (ddd, ^3J = 8.0 Hz, ^3J = 7.2 Hz, ^4J = 1.4 Hz, 1H, ArH-6), 7. 48 (ddd, ^3J = 7.7 Hz, ^3J = 7.2 Hz, ^4J =0.9 Hz, 1H, ArH-5), 7.36 (dd, ^3J = 7.7 Hz, ^3J = 7.2 Hz, 1H, PhH-5), 7.32 (dd, ^4J = 2.5 Hz, ^4J = 2.0 Hz, 1H, PhH-2), 7.16 (ddd, ^3J = 7.9 Hz, ^4J = 2.0 Hz, ^4J = 0.7 Hz, 1H, PhH-6), 6.68 (ddd, ^3J = 8.3 Hz, ^4J = 2.5 Hz, ^4J = 0.7 Hz, 1H, PhH-4), 3.79 (s, 3H, OCH_3) ppm; ^{13}C-NMR (151.0 MHz, DMSO-d_6): δ 164.93 (C=O), 159.58 (PhC-3), 140.78 (PhC-1), 138.79 (CSe), 132.20 (ArC-6), 129.91 (PhC-5), 128.53 (ArC-3’), 127.86 (ArC-4), 126.18 (ArC-5), 125.71 (ArC-7), 116.58 (PhC-6), 111.27 (PhC-4), 110.29 (PhC-2), 55.17 (OCH_3) ppm; ^{77}Se-NMR (76.24 MHz, DMSO-d_6): δ 916.40 ppm; MS: *m/z* for $C_{14}H_{11}NO_2Se$ + H^+ calculated: 306.0028; found: 305.85. ^{1}H-NMR and ^{13}C-NMR spectra agree with the literature data [12].

2-(4-Trifluoromethylphenyl)-1,2-benzisoselenazol-3(2*H*)-one (**9**)

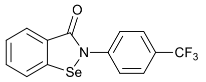

The general procedure starting from 4-trifluoromethylaniline (0.806 g, 5.0 mmol) was employed with a 1-h reaction time to obtain 2-(4-trifluoromethylphenyl)-1,2-benzisoselenazol-3(2*H*)-one (**9**) (1.11 g, 3.25 mmol) as a yellow solid with 65% yield. The analytically pure sample was obtained by recrystallization from EtOAc (40 mL/g) to obtain **9** (0.930 g, 2.72 mmol) as bright yellow flakes with 54% yield, which melted at 241–242°C (from EtOAc) (m.p. 246–248°C [7]). Selected FT-IR (KBr): ν_{max}

3061 (C_{Ar}-H), 1625, 1600 (C=O), 1446, 1329 (C_{Ar}-N), 1307, 1194, 1117, 1073, 840, 740 (C-Se), 673, 525, 408 cm^{-1}; ^{1}H-NMR (600.6 MHz, DMSO-d_6): δ 8.10 (dd, ^{3}J = 8.0 Hz, ^{4}J = 0.9 Hz, 1H, ArH-7), 7.944 (d, ^{3}J = 8.5 Hz, 2H, PhH-2,6), 7.936 (dd, ^{3}J = 7.7 Hz, ^{4}J = 1.4 Hz, 1H, ArH-4), 7.80 (d, ^{3}J = 8.5 Hz, 2H, PhH-3,5), 7.71 (ddd, ^{3}J = 8.0 Hz, ^{3}J = 7.5 Hz, ^{4}J = 1.4 Hz, 1H, ArH-6), 7.50 (ddd, ^{3}J = 7.7 Hz, ^{3}J = 7.5 Hz, ^{4}J = 0.9 Hz, 1H, ArH-5) ppm; ^{13}C-NMR (100.5 MHz, DMSO-d_6): δ 165.37 (C=O), 143.59 (PhC-1), 138.63 (CSe), 132.64 (ArC-6), 128.37 (ArC-3'), 128.07 (ArC-4), 126.37 (ArC-5), 126.25 (k, $^{3}J_{CF}$ = 3.7 Hz, PhC-3,5), 125.84 (ArC-7), 125.24 (k, $^{2}J_{CF}$ = 32.3 Hz, PhC-4), 125.15 (PhC-2,6), 124.08 (k, $^{1}J_{CF}$ = 271.8 Hz, CF_3) ppm; ^{77}Se-NMR (76.24 MHz, DMSO-d_6): δ 919.59 ppm; ^{19}F-NMR (376.2 MHz, DMSO-d_6): δ –60.56 ppm HRMS (TOF, MS, ESI) m/z for $C_{14}H_8F_3NOSe + H^+$ calculated: 343.9796; found: 343.9794.

2-(4-Nitrophenyl)-1,2-benzisoselenazol-3(2*H*)-one (**10**)

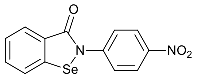

The general procedure starting from 4-nitroaniline (0.691 g, 5.0 mmol) was employed with a 2-h reaction time to obtain of 2-(4-nitrophenyl)-1,2-benzisoselenazol-3(2*H*)-one (**10**) (0.818 g, 2.56 mmol) as orange prisms with 51% yield. The analytically pure sample was obtained by recrystallization from DMSO (4 mL/g) to obtain **10** (0.721g, 2.26 mmol) as yellow prisms with 45% yield, which melted at 286–288°C (from DMSO) with decomposition (m.p. 280–282°C [8]). Selected FT-IR (KBr): ν_{max} 3061 (C_{Ar}-H), 1606 (C=O), 1584 (NO_2), 1494, 1328 (NO_2), 1319, 1305 (C_{Ar}-N), 1267, 1112, 844, 735 (C-Se), 672, 524, 473, 445 cm^{-1}; ^{1}H-NMR (399.8 MHz, DMSO-d_6): δ 8.29 (d, ^{3}J = 9.2 Hz, 2H, PhH-3,5), 8.09 (dd, ^{3}J = 8.1 Hz, ^{4}J = 0.9 Hz, 1H, ArH-7), 8.04 (d, ^{3}J = 9.2 Hz, 2H, PhH-2,6), 7.94 (dd, ^{3}J = 7.8 Hz, ^{4}J = 1.3 Hz, ArH-4), 7.72 (ddd, ^{3}J = 8.1 Hz, ^{3}J = 7.2 Hz, ^{4}J = 1.3 Hz, 1H, ArH-6), 7.49 (ddd, ^{3}J = 7.8 Hz, ^{3}J = 7.2 Hz, ^{4}J = 0.9 Hz, ArH-5) ppm; ^{13}C-NMR (100.5 MHz, DMSO-d_6): δ 165.68 (C=O), 146.27 (PhC-1), 143.33 (PhC-4), 138.50 (CSe), 133.01 (ArC-6), 128.37 (ArC-3'), 128.22 (ArC-4), 126.52 (ArC-5), 125.85 (ArC-7), 124.83 (PhC-3,5), 123.39 (PhC-2,6) ppm; ^{77}Se-NMR (76.24 MHz, DMSO-d_6): δ 923.30 ppm; HRMS (TOF, MS, ESI) m/z for $C_{13}H_8N_2O_3Se + H^+$ calculated: 320.9773; found: 320.9776. The spectral data are consistent with the literature [8].

2-(4-Iodophenyl)-1,2-benzisoselenazol-3(2*H*)-one (**11**)

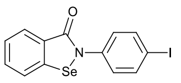

The general procedure starting from 4-iodoaniline (1.10 g, 5.0 mmol) was employed with a 1.5-h reaction time to obtain 2-(4-iodophenyl)-1,2-benzisoselenazol-3(2*H*)-one (**11**) (1.76 g, 4.40 mmol) as a heavy flesh-color solid with 88% yield. The analytically pure sample was obtained by recrystallization from EtOAc (100 mL/g) to obtain **11** (1.48 g, 3.70 mmol) as light yellow needles with 74% yield, which melted at 203–204°C (from EtOAc) (m.p. 198–200°C [8], m.p. 201–202°C [9]). Selected FT-IR (KBr): ν_{max} 3094 (C_{Ar}-H), 3064 (C_{Ar}-H), 1595 (C=O), 1568, 1483, 1330 (C_{Ar}-N), 1304, 1265, 1137, 999, 820, 795, 731 (C-Se), 671, 524, 506, 474 cm^{-1}; ^{1}H-NMR (399.8 MHz, DMSO-d_6): δ 8.08 (dd, ^3J = 8.1 Hz, ^4J = 0.5 Hz, 1H, ArH-7), 7.90 (dd, ^3J = 7.9 Hz, ^4J = 1.3 Hz, 1H, ArH-4), 7.78 (d, ^3J = 8.6 Hz, 2H, PhH-3,5), 7.68 (ddd, ^3J = 8.1 Hz, ^3J = 7.2 Hz, ^4J = 1.3 Hz, 1H, ArH-6), 7.50 (d, ^3J = 8.6 Hz, 2H, PhH-2,6), 7.47 (ddd, ^3J = 7.9 Hz, ^3J = 7.2 Hz, ^4J = 0.5 Hz, ArH-5) ppm; ^{13}C-NMR (100.5 MHz, DMSO-d_6): δ 165.00 (C=O), 139.60 (PhC-1), 138.60 (CSe), 137.79 (PhC-3,5), 132.37 (ArC-6), 128.35 (ArC-3’), 127.94 (ArC-4), 126.40 (PhC-2.6), 126.27 (ArC-5), 125.78 (ArC-7), 90.21 (C-I) ppm; ^{77}Se-NMR (76.24 MHz, DMSO-d_6): δ 916.59 ppm; HRMS (TOF, MS, ESI) m/z for C13H8INOSe + H^+ calculated: 401.8889; found: 401.8896. IR and ^{1}H-NMR spectra are consistent with the literature value [9].

2-(4-Acetylphenyl)-1,2-benzisoselenazol-3(2*H*)-one (**12**) [10]

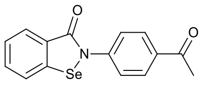

The general procedure starting from 4-aminoacetophenone (0.676 g, 5.0 mmol), and (2-chloroseleno)benzoyl chloride (1.90 g, 7.5 mmol) was employed with a 16-h reaction time and the product was isolated by extraction with EtOAc (250 mL), and purification by column chromatography on silica gel (70–230 mesh) using EtOAc as an eluent, to obtain 2-(4-acetylphenyl)-1,2-benzisoselenazol-3(2*H*)-one (**12**) (1.06 g, 3.35 mmol) with 67% yield calculated on used 4-aminoacetophenone. Analytically pure sample was obtained by repetitive crystallization from EtOAc to obtain **12** (0.760 g, 2.40 mmol) as pale yellow prisms with 48% yield, which melted at 185–186°C. Selected FT-IR (ATR): ν_{max} 3063 (C_{Ar}-H), 2998 (CH_3), 1672 (C=O), 1607, 1584 (C_{Ar}=O), 1561, 1504, 1415, 1323, 1307, 1261 (C-N), 1184, 1139, 959, 831, 738 (C-Se), 672, 586, 530, 574, 416 cm^{-1}; ^{1}H-NMR (399.8 MHz, DMSO-d_6): δ 8.10 (d, ^3J = 8.1 Hz, 1H, ArH-7), 8.03 (d, ^3J = 8.6 Hz, 2H, PhH-3,5), 7.93 (dd, ^3J = 7.7 Hz, ^4J = 0.9 Hz, 1H, ArH-4), 7.88 (d, ^3J = 8.6 Hz, 2H, PhH-2,6), 7.71 (ddd, ^3J =8.1 Hz, ^3J = 7.2 Hz, ^4J = 0.9 Hz, 1H, ArH-6), 7.50 (dd, ^3J =7.7 Hz, ^3J = 7.2 Hz, 1H, ArH-5), 2.59 (s, 3H, Ac) ppm; ^{13}C-NMR (100.5 MHz, DMSO-d_6): δ 196.66 (C=O), 165.32 (C_{Ar}=O), 144.30 (PhC-1), 138.58 (CSe), 133.25 (PhC-4), 132.65 (ArC-6), 129.43 (PhC-3,5), 128.53 (ArC-3’),

128.06 (ArC-4), 126.38 (ArC-5), 125.81 (ArC-7), 123.24 (PhC-2,6), 26.57 (CH_3) ppm; ^{77}Se-NMR (76.24 MHz, DMSO-d_6): δ 916.79 ppm; HRMS (TOF, MS, ESI) m/z for $C_{15}H_{11}NO_2Se + H^+$ calculated: 318.0028; found: 318.0044. Anal. Calcd. for $C_{15}H_{11}NO_2Se$ (316.23): C, 56.97; H, 3.51; N, 4.43. Found: C, 56.93; H, 3.43; N, 4.50.

***N*-(4-(1,2-benzisoselenazol-3(2*H*)-one-2-yl)phenyl]acetamide** (**13**)

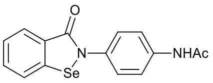

The general procedure starting from 4-acetaminoaniline (0.751 g, 5.0 mmol), in anhydrous CH_2Cl_2 (50 mL), was employed with a 4-day reaction time with washing with 5% aq. HCl, water, brine, MeOH, and $CHCl_3$ to obtain the *N*-(4-(1,2-benzisoselenazol-3(2*H*)-one-2-yl)phenyl]acetamide (**13**) (1.11 g, 3.35 mmol) as a pale-yellow solid with 67% yield. Analytically pure sample was obtained by recrystallization from DMSO to obtain **13** (0.892 g, 2.69 mmol) as bright shiny flakes with 54% yield, which melted at 282–283°C with decomposition. Selected FT-IR (ATR): ν_{max} 3301 (N-H), 3063 (C_{Ar}-H), 2923 (CH_3), 1664, 1621 (C=O), 1599 (C=O), 1538 (CONH), 1509, 1440, 1409, 1329 (C_{Ar}-N), 1305 (C_{Ar}-N), 1260, 1133, 1017, 996, 950, 833, 823, 728 (C-Se), 705, 670, 598, 530, 517, 483 cm^{-1}; ^{1}H-NMR (600.6 MHz, DMSO-d_6): δ 10.05 (s, 1H, NH), 8.08 (d, ^3J = 8.0 Hz, 1H, ArH-7), 7.89 (d, ^3J = 7.7 Hz, ^4J = 1.4 Hz, 1H, ArH-4), 7.67 (ddd, ^3J = 8.0 Hz, ^3J =7.2 Hz, ^4J = 1.4 Hz, 1H, ArH-6), 7.64 (d, ^3J = 8.8 Hz, 2H, PhH-3,5), 7.54 (d, ^3J = 8.8 Hz, 2H, PhH-2,6), 7.47 (ddd, ^3J = 7.7 Hz, ^3J =7.2 Hz, ^4J = 0.8 Hz, 1H, ArH-5), 2.06 (s, 3H, CH_3) ppm; ^{13}C-NMR (100.5 MHz, DMSO-d_6): δ 168.26 (C=O), 164.87 (C_{Ar}=O), 138.82 (CSe), 137.11 (PhC-4), 134.38 (PhC-1), 132.08 (ArC-6), 128.35 (ArC-3’), 127.83 (ArC-4), 126.17 (ArC-5), 125.75 (ArC-7), 125.20 (PhC-2,6), 119.43 (PhC-3,5), 23.92 (CH_3) ppm; ^{77}Se-NMR (76.24 MHz, DMSO-d_6): δ 914.33 ppm; HRMS (TOF, MS, ESI) m/z for $C_{15}H_{12}N_2O_2Se + H^+$ calculated: 333.0137; found: 333.0148.

2-(2,4-difluorophenyl)-1,2-benzisoselenazol-3(2*H*)-one (**14**) [10]

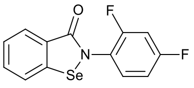

The general procedure starting from 2,4-difluoroaniline (0.646 g, 5.0 mmol) was employed with an 8-h reaction time, and product extraction with EtOAc to obtain 2-(2,4-difluorophenyl-1,2-benzisoselenazol-3(2*H*)-one (1**4**) (1.16 g, 3.74 mmol) as pale yellow crystals with 75% yield, after crystallization from EtOAc, which melted at 175–176°C (from EtOAc). Selected FT-IR (KBr): ν_{max} 3097 (C_{Ar}-H), 3051 (C_{Ar}-H), 1603 (C=O), 1507, 1443, 1436, 1351 (C_{Ar}-N), 1283, 1265, 1145, 1095, 956, 881, 809, 737 (C-Se), 676, 609,

586, 547, 515, 471 cm^{-1}; ^{1}H-NMR (600.6 MHz, DMSO-d_6): δ 8.10 (d, ^{3}J = 8.0 Hz, 1H, ArH-7), 7.91 (dd, ^{3}J = 7.7 Hz, ^{4}J = 1.4 Hz, 1H, ArH-4), 7.70 (ddd, ^{3}J = 8.0 Hz, ^{3}J = 7.2 Hz, ^{4}J = 1.4 Hz, 1H, ArH-6), 7.56 (ddd, $^{3}J_{HF}$ = 8.8 Hz, ^{3}J = 8.8 Hz, $^{4}J_{HF}$ = 6.1 Hz, 1H, PhH-6), 7.49 (ddd, ^{3}J = 7.7 Hz, ^{3}J = 7.2 Hz, ^{4}J = 0.9 Hz, 1H, ArH-5), 7.45 (ddd, $^{3}J_{HF}$ = 10.3 Hz, $^{3}J_{HF}$ = 9.2 Hz, ^{4}J = 2.9 Hz, 1H, PhH-3), 7.20 (dddd, ^{3}J = 8.8 Hz, $^{3}J_{HF}$ = 8.2 Hz, ^{4}J = 2.9 Hz, $^{5}J_{HF}$ = 1.2 Hz, 1H, PhH-5) ppm; ^{13}C-NMR (100.5 MHz, DMSO-d_6): δ 165.78 (C=O), 161.16 (dd, $^{1}J_{CF}$ = 247.4 Hz, $^{3}J_{CF}$ = 11.5 Hz, PhC-4), 157.90 (dd, $^{1}J_{CF}$ = 252.5 Hz, $^{3}J_{CF}$ = 13.0 Hz, PhC-2), 140.36 (CSe), 132.34 (ArC-6), 131.47 (d, $^{3}J_{CF}$ = 9.3 Hz, PhC-6), 127.94 (ArC-4), 126.65 (ArC-3’), 126.22 (ArC-5), 126.04 (ArC-7), 122.87 (dd, $^{2}J_{CF}$ = 13.2 Hz, $^{4}J_{CF}$ = 3.5 Hz, PhC-1), 111.94 (dd, $^{2}J_{CF}$ = 22.4 Hz, $^{4}J_{CF}$ = 3.2 Hz, PhC-5), 105.06 (dd, $^{2}J_{CF}$ = 25.9 Hz, $^{2}J_{CF}$ = 25.2 Hz, PhC-3) ppm; ^{19}F-NMR (376.2 MHz, DMSO-d_6): δ −109.71 (dddd, $^{3}J_{FH}$ = 10.3 Hz, $^{3}J_{FH}$ = 8.2 Hz, $^{4}J_{FF}$ = 7.7 Hz, $^{4}J_{FH}$ = 6.1 Hz, 1F, PhF-4), −113.86 (dddd, $^{3}J_{FH}$ = 9.2 Hz, $^{3}J_{FH}$ = 8.8 Hz, $^{4}J_{FF}$ = 7.7 Hz, $^{5}J_{FH}$ = 1.2 Hz, 1F, PhF-2) ppm; ^{77}Se-NMR (76.24 MHz, DMSO-d_6): δ 942.50 (d, $^{4}J_{SeF}$ = 15.1 Hz) ppm; HRMS (TOF, MS, ESI) m/z for $C_{13}H_7F_2NOSe + H^+$ calculated: 311.9734; found: 311.9739.

2-(4-chloro-2-fluorophenyl)-1,2-benzisoselenazol-3(2*H*)-one (**15**) [11]

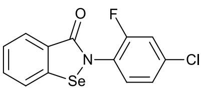

The general procedure starting from 4-chloro-2-fluoroaniline (0.728 g, 5.0 mmol) was employed with a 3.5-h reaction time with product separation by extraction with EtOAc to obtain 2-(4-chloro-2-fluorophenyl)-1,2-benzisoselenazol-3(2*H*)-one (**15**) (1.39 g, 4.26 mmol) as a pale yellow solid with 85% yield. Recrystallization from EtOAc afforded the analytically pure sample of **15** (0.867 g, 2.65 mmol) as a colorless wool with 53% yield, which melted at 220–221°C (from EtOAc). Selected FT-IR (KBr): $ν_{max}$ 3090 (C_{Ar}-H), 3065 (C_{Ar}-H), 1585 (C=O), 1574, 1561, 1494, 1445, 1363 (C-N), 1232, 899, 861, 810, 737 (C-Se), 678, 528, 495, 462, 442 cm^{-1}; ^{1}H-NMR (600.6 MHz, DMSO-d_6): δ 8.09 (dd, ^{3}J = 8.1 Hz, ^{4}J = 0.9 Hz, 1H, ArH-7), 7.91 (dd, ^{3}J = 7.7 Hz, ^{4}J = 1.2 Hz, 1H, ArH-4), 7.70 (ddd, ^{3}J = 8.1 Hz, ^{3}J = 7.2 Hz, ^{4}J = 1.2 Hz, 1H, ArH-6), 7.63 (dd, $^{3}J_{HF}$ = 10.1 Hz, ^{4}J = 2.3 Hz, 1H, PhH-3), 7.55 (dd, ^{3}J = 8.5 Hz, $^{4}J_{HF}$ = 8.3 Hz, 1H, PhH-6), 7.49 (ddd, ^{3}J = 7.7 Hz, ^{3}J = 7.2 Hz, ^{4}J = 0.9 Hz, 1H, ArH-5), 7.39 (ddd, ^{3}J = 8.5 Hz, ^{4}J = 2.3 Hz, $^{5}J_{HF}$ = 0.8 Hz, 1H, PhH-5) ppm; ^{13}C-NMR (100.5 MHz, DMSO-d_6): δ 165.65 (C=O), 157.47 (d, $^{1}J_{CF}$ = 253.6 Hz, CF), 140.34 (CSe), 132.61 (d, $^{2}J_{CF}$ = 9.9 Hz, PhC-1), 132.35 (ArC-6), 131.31 (PhC-6), 127.93 (ArC-4), 126.60 (ArC-3’), 126.20 (ArC-5), 126.02 (ArC-7), 125.59 (d, $^{3}J_{CF}$ = 13.1 Hz, CCl), 125.09 (d, $^{4}J_{CF}$ = 3.3 Hz, PhC-5), 117.12 (dd, $^{2}J_{CF}$ = 23.7 Hz, PhC-3) ppm; ^{77}Se-NMR (76.24 MHz, DMSO-d_6):

δ 946.74 (d, $^4J_{SeF}$ = 19.8 Hz) ppm; ^{19}F-NMR (376.2 MHz, DMSO-d_6): δ −115.16 (dd, $^3J_{FH}$ = 10.1 Hz, $^4J_{FH}$ = 8.3 Hz) ppm; HRMS (TOF, MS, ESI) m/z for $C_{13}H_7ClFNOSe$ + H^+ calculated: 327.9438; found: 327.9446.

2-(2,4-Dimethoxyphenyl)-1,2-benzisoselenazol-3(2*H*)-one (**16**)

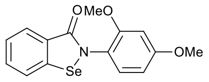

The general procedure starting from 2,4-dimethoxyaniline (0.766 g, 5.0 mmol) was employed with a 5-h reaction time and product isolation by column chromatography on silica gel (70–230 mesh) using chloroform as an eluent to obtain 2-(2,4-dimethoxyphenyl)-1,2-benzisoselenazol-3(2*H*)-one (**16**) (1.27 g, 3.8 mmol) as a pale solid with 76% yield. The analytically pure sample was obtained by recrystallization from chloroform (75 mL/g) to obtain **16** (1.10 g, 3.29 mmol) as a colorless prisms with 66% yield, which melted at 238–240°C (from $CHCl_3$) (m.p. 239–240°C [1]). Selected FT-IR (KBr): ν_{max} 3050 (C_{Ar}-H), 1601 (C=O), 1588, 1510, 1442, 1208, 1162 (C-O), 1044 (C-O), 747 (C-Se) cm^{-1}; ^{1}H-NMR (399.8 MHz, DMSO-d_6): δ 8.05 (ddd, ^3J = 8.0 Hz, ^4J = 1.0 Hz, ^5J = 0.5 Hz, 1H, ArH-7), 7.85 (ddd, ^3J = 7.8 Hz, ^4J = 1.4 Hz, ^5J = 0.5 Hz, 1H, ArH-4), 7.65 (ddd, ^3J = 8.0 Hz, ^3J = 7.2 Hz, ^4J = 1.4 Hz, 1H, ArH-6), 7.45 (ddd, ^3J = 7.8 Hz, ^3J = 7.2 Hz, ^4J = 1.0 Hz, 1H, ArH-5), 7.23 (d, ^3J = 8.6 Hz, 1H, PhH-6), 6.70 (d, ^4J = 2.7 Hz, 1H, PhH-3), 6.58 (dd, ^3J = 8.6 Hz, ^4J = 2.7 Hz, 1H, PhH-5), 3.81 (s, 3H, 4-OCH_3), 3.75 (s, 3H, 2-OCH_3) ppm; ^{13}C-NMR (100.5 MHz, DMSO-d_6): δ 165.76 (C=O), 160.08 (PhC-4), 156.29 (PhC-2), 140.29 (CSe), 131.77 (PhC-6), 130.33 (ArC-6), 127.73 (ArC-4), 127.30 (ArC-3'), 125.77 (ArC-5), 125.74 (ArC-7), 119.75 (PhC-1), 104.86 (PhC-5), 99.39 (PhC-3), 55.68 (2-OCH_3), 55.45 (4-OCH_3) ppm; ^{77}Se-NMR (76.24 MHz, DMSO-d_6): δ 927.78 ppm; The FT-IR, ^{1}H-NMR, ^{13}C-NMR spectra and HRMS analysis are consistent with the literature value [1].

2-(5-Chloro-2-fluorophenyl)-1,2-benzisoselenazol-3(2*H*)-one (**17**) [4]

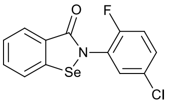

The general procedure starting from 5-chloro-2-fluoroaniline (0.728 g, 5.0 mmol) was employed with a 6-day reaction time, and washing the solid with MeCN/H_2O (2:3, *v/v*) (3 × 2 mL), to obtain 2-(5-chloro-2-fluorophenyl)-1,2-benzisoselenazol-3(2*H*)-one (**17**) (0.830 g, 2.54 mmol) as colorless needles with 51% yield. The analytically pure sample was obtained by recrystallization from EtOAc (25 mL/g) to obtain product **17** (0.590 g, 1.81 mmol) as a colorless wool with 36% yield, which melted at 197–198°C (from $CHCl_3$). Selected FT-IR (KBr): ν_{max} 3065 (C_{Ar}-H), 1600 (C=O), 1495, 1443, 1406, 1329 (C-N), 1232,

1211, 1137, 1106, 967, 866, 809, 736 (C-Se), 675, 654, 515, 467 cm^{-1}; ^{1}H-NMR (399.8 MHz, DMSO-d_6): δ 8.10 (d, ^{3}J = 8.1 Hz, 1H, ArH-7), 7.91 (dd, ^{3}J = 7.8 Hz, ^{4}J = 1.3 Hz, 1H, ArH-4), 7.70 (ddd, ^{3}J = 8.1 Hz, ^{3}J = 7.6 Hz, ^{4}J = 1.3 Hz, 1H, ArH-6), 7.65 (dd, $^{4}J_{HF}$ = 6.5 Hz, ^{4}J = 2.6 Hz, 1H, PhH-6), 7.50 (ddd, ^{3}J = 8.7 Hz, $^{4}J_{HF}$ = 4.4 Hz, ^{4}J = 2.6 Hz, 1H, PhH-4), 7.49 (ddd, ^{3}J = 7.8 Hz, ^{3}J =7.6 Hz, ^{4}J = 0.9 Hz, 1H, ArH-5), 7.44 (dd, $^{3}J_{HF}$ = 10.0 Hz, ^{3}J = 8.7 Hz, 1H, PhH-3) ppm; ^{13}C-NMR (100.5 MHz, DMSO-d_6): δ 165.75 (C=O), 156.42 (d, $^{1}J_{CF}$ = 250.2 Hz, CF), 140.42 (CSe), 132.42 (ArC-6), 129.78 (PhC-6), 129.06 (d, $^{3}J_{CF}$ = 7.9 Hz, PhC-4), 128.12 (d, $^{4}J_{CF}$ = 3.1 Hz, CCl), 127.96 (ArC-4), 127.79 (d, $^{2}J_{CF}$ = 14.6 Hz, PhC-1), 126.54 (ArC-3'), 126.20 (ArC-5), 126.02 (ArC-7), 118.09 (d, $^{2}J_{CF}$ = 22.0 Hz, PhC-3) ppm; ^{77}Se-NMR (76.24 MHz, DMSO-d_6): δ 952.86 (d, $^{4}J_{SeF}$ = 25.5 Hz) ppm; ^{19}F-NMR (376.2 MHz, DMSO-d_6): δ −120.34 (ddd, $^{3}J_{FH}$ = 10.0 Hz, $^{4}J_{FH}$ = 6.5 Hz, $^{4}J_{FH}$ = 4.4 Hz) ppm; HRMS (TOF, MS, ESI) m/z for $C_{13}H_7ClFNOSe + H^+$ calculated: 327.9438; found: 327.9446.

2-(2,5-Dichlorophenyl)-1,2-benzisoselenazol-3(2*H*)-one (**18**) [4]

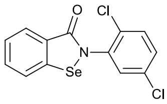

The general procedure starting from 2,4-dichloroaniline (0.810 g, 5.0 mmol) was employed with a 5-h reaction time and product extraction with EtOAc to obtain 2-(2,5-dichlorophenyl)-1,2-benzisoselenazol-3(2*H*)-one (**18**) (1.46 g, 4.25 mmol) as a pale yellow solid with 85%. The analytically pure sample was obtained by recrystallization from EtOAc to obtain **18** (0.910 g, 2.65 mmol) as pale yellow solid with 53% yield, which melted at 188–189°C (from EtOAc). Selected FT-IR (ATR): ν_{max} 3064 (C_{Ar}-H), 1605 (C=O), 1590, 1578, 1566, 1466, 1443, 1388, 1321 (C-N), 1256, 1097, 1050, 966, 899, 875, 810, 783, 734 (C-Se), 673, 582, 458 cm^{-1}; ^{1}H-NMR (399.8 MHz, DMSO-d_6): δ 8.10 (d, ^{3}J = 8.1 Hz, 1H, ArH-7), 7.90 (d, ^{3}J = 7.8 Hz, 1H, ArH-4), 7.70 (dd, ^{3}J = 8.1 Hz, ^{3}J =7.2 Hz, 1H, ArH-6), 7.66 (d, ^{3}J = 8.6 Hz, 1H, PhH-3), 7.66 (d, ^{4}J = 2.5 Hz, 1H, PhH-6), 7.53 (dd, ^{3}J = 8.6 Hz, ^{4}J = 2.5 Hz, 1H, PhH-4), 7.49 (dd, ^{3}J = 7.8 Hz, ^{3}J =7.2 Hz, 1H, ArH-5) ppm; ^{13}C-NMR (75.48 MHz, DMSO-d_6): δ 165.70 (C=O), 140.49 (C), 137.56 (C), 132.19 (CH), 131.74 (C), 131.48 (C), 131.29 (CH), 130.82 (CH), 129.45 (CH), 127.88 (CH), 126.50 (C), 125.98 (CH), 125.95 (CH) ppm; ^{13}C-NMR (100.5 MHz, DMSO-d_6): δ 165.79 (C=O), 140.59 (CSe), 137.65 (PhC-1), 132.36 (ArC-6), 131.82 (CCl), 131.57 (CCl), 131.40 (PhC-3 or PhC-6), 130.96 (PhC-3 or PhC-6), 129.60 (PhC-4), 127.98 (ArC-4), 126.58 (ArC-3'), 126.14 (ArC-5), 126.05 (ArC-7) ppm; ^{77}Se-NMR (76.24 MHz, DMSO-d_6): δ 947.54 ppm; HRMS (TOF, MS, ESI) m/z for $C_{13}H_7NOSe + H^+$ calculated: 343.9143; found: 343.9140.

2-(2-Chloro-5-methylphenyl)-1,2-benzisoselenazol-3(2*H*)-one (19)

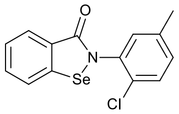

The general procedure starting from 2-chloro-5-metylaniline (0.708 g, 5.0 mmol) was employed with a 24-h reaction time, product extraction with DCM, and product isolation by column chromatography (silica gel, 70–230 mesh) using $CHCl_3$:EtOAc in gradient as an eluent to obtain 2-(2-chloro-5-methylphenyl)-1,2-benzisoselenazol-3(2*H*)-one (**19**) (1.01 g, 3.13 mmol) as a white powder with 63% yield, which melted at 183–184°C (from CH_2Cl_2) (m.p. 184–185°C [12]). Selected FT-IR (ATR): ν_{max} 3059 (C_{Ar}-H), 2920 (CH_3), 1604 or 1592 (C=O), 1479, 1441, 1340 (C-N), 1060, 1020, 817, 735 (C-Se), 675, 617, 563, 525, 450 cm^{-1}; ^{1}H-NMR (399.8 MHz, DMSO-d_6): δ 8.09 (d, ^3J = 8.0 Hz, 1H, ArH-7), 7.89 (dd, ^3J = 7.8 Hz, ^4J = 1.2 Hz, 1H, ArH-4), 7.69 (ddd, ^3J = 8.0 Hz, ^3J = 7.2 Hz, ^4J = 1.2 Hz, 1H, ArH-6), 7.48 (ddd, ^3J = 7.8 Hz, ^3J = 7.2 Hz, ^4J = 1.0 Hz, 1H, ArH-5), 7.48 (d, ^3J = 8.2 Hz, 1H, PhH-3), 7.32 (d, ^4J = 1.7 Hz, 1H, PhH-6), 7.26 (dd, ^3J = 8.2 Hz, ^4J = 1.7 Hz, 1H, PhH-4), 2.33 (s, 3H, CH_3) ppm; ^{13}C-NMR (100.5 MHz, DMSO-d_6): δ 165.60 (C=O), 140.34 (CSe), 137.84 (PhC-5), 135.77 (PhC-1), 132.16 (ArC-6), 131.39 (PhC-6), 130.33 (PhC-4), 129.68 (PhC-3), 129.35 (CCl), 127.92 (ArC-4), 126.84 (ArC-3’), 126.08 (ArC-5), 125.98 (ArC-7), 20.12 (CH_3) ppm; ^{77}Se-NMR (76.24 MHz, DMSO-d_6): δ 936.44 ppm; The ^{1}H-NMR and ^{13}C-NMR spectra and HRMS analysis are consistent with the literature value [12].

2-(5-Chloro-2-methylphenyl)-1,2-benzisoselenazol-3(2*H*)-one (20)

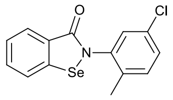

The general procedure starting from 5-chloro-2-metylaniline (0.708 g, 5.0 mmol) was employed with an 18-h reaction time, product extraction with $CHCl_3$, and the organic layer was washed with 3.3% aq. HCl and water to obtain 2-(5-chloro-2-methylphenyl)-1,2-benzisoselenazol-3(2*H*)-one (**20**) (1.34 g, 4.15 mmol) with 83% yield. The analytically pure sample was obtained by recrystallization from $CHCl_3$, before decolorization with charcoal, in an open vessel to obtain **20** (0.829 g, 2.57 mmol) as a white powder with 51% yield, which melted at 190–191°C (from $CHCl_3$) (m.p. 189–190°C [12]). Selected FT-IR (ATR): ν_{max} 3062 (C_{Ar}-H), 2921 (CH_3), 1589 (C=O), 1561, 1483, 1442, 1336 (C-N), 1120, 962, 815, 740 (C-Se), 679, 617, 518, 453 cm^{-1}; ^{1}H-NMR (399.8 MHz, DMSO-d_6): δ 8.10 (d, ^3J = 8.1 Hz, 1H, ArH-7), 7.90 (d, ^3J = 7.8 Hz, 1H, ArH-4), 7.69 (ddd, ^3J = 8.1 Hz, ^3J = 7.2 Hz, ^4J = 0.9 Hz, 1H, ArH-6), 7.49 (dd, ^3J = 7.8 Hz, ^3J = 7.2 Hz, 1H, ArH-5), 7.36–7.42 (m, 3H, PhH-3,4,6), 2.08 (s, 3H, CH_3) ppm; ^{13}C-NMR (100.5 MHz, DMSO-d_6): δ 165.23 (C=O), 140.26 (C-Se), 138.83 (PhC-2), 135.77 (PhC-1), 132.19 (PhC-3), 132.07 (ArC-6),

130.25 (CCl), 128.57 (PhC-4 or PhC-6), 127.99 (PhC-4 or PhC-6), 127.89 (ArC-4), 126.99 (ArC-3'), 126.10 (ArC-5), 125.99 (ArC-7), 17.28 (CH_3) ppm; ^{77}Se-NMR (76.24 MHz, DMSO-*d*$_6$): δ 927.66 ppm; The ^{1}H-NMR and ^{13}C-NMR spectra and HRMS analysis are consistent with the literature value [12].

2-(3-Chloro-2-methylphenyl)-1,2-benzisoselenazol-3(2*H*)-one (21)

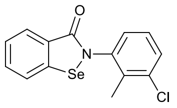

The general procedure starting from 3-chloro-2-metylaniline (0.708 g, 5.0 mmol) was employed with a 72 h reaction time, and recrystallization of crude product from $ClCH_2CH_2Cl$ to obtain 2-(3-chloro-2-methylphenyl)-1,2-benzisoselenazol-3(2*H*)-one (**21**) (1.02 g, 3.15 mmol) as yellow crystals with 63% yield, which melted at 282–283°C (from $ClCH_2CH_2Cl$) (m.p.[11] 282–283°C). Selected FT-IR (ATR): ν_{max} 3097 (C_{Ar}-H), 3062 (C_{Ar}-H), 2923 (CH_3), 1598 or 1589 (C=O), 1563, 1441, 1345 (C-N), 1188, 1015, 869, 779, 733 (C-Se), 699, 677, 643, 525, 498, 477, 420 cm^{-1}; ^{1}H-NMR (600.6 MHz, DMSO-*d*$_6$): δ 8.10 (d, ^3J = 8.0 Hz, 1H, ArH-7), 7.90 (dd, ^3J = 7.7 Hz, ^4J = 1.1 Hz, 1H, ArH-4), 7.70 (ddd, ^3J = 8.0 Hz, ^3J = 7.0 Hz, ^4J = 1.1 Hz, 1H, ArH-6), 7.52 (dd, ^3J = 7.8 Hz, ^4J = 1.5 Hz, 1H, PhH-4 or PhH-6), 7.50 (ddd, ^3J = 7.7 Hz, ^3J = 7.0 Hz, ^4J = 0.8 Hz, 1H, ArH-5), 7.33 (dd, ^3J = 7.8 Hz, ^3J = 7.8 Hz, 1H, ArH-5), 7.31 (dd, ^3J = 7.8 Hz, ^4J = 1.5 Hz, 1H, PhH-4 or PhH-6), 2.11 (s, 3H, CH_3) ppm; ^{13}C-NMR (75.48 MHz, DMSO-*d*$_6$): δ 165.27 (C=O), 140.16 (CSe), 138.96 (CCl or CN), 134.79 (PhC-2), 134.10 (CCl or CN), 132.11 (ArC-6), 128.74 (PhC-4 or PhC-6), 127.98 (PhC-5), 127.89 (ArC-4), 127.48 (PhC-4 or PhC-6), 126.88 (ArC-3'), 126.14 (ArC-5), 125.96 (ArC-7), 15.27 (CH_3) ppm; ^{77}Se-NMR (76.24 MHz, DMSO-*d*$_6$): δ 928.10 ppm; The ^{1}H-NMR and ^{13}C-NMR spectra and HRMS analysis are consistent with the literature value [12].

2-(4-Chloro-3-methylphenyl)-1,2-benzisoselenazol-3(2*H*)-one (22)

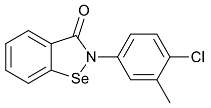

The general procedure starting from 4-chloro-3-metylaniline (0.708 g, 5.0 mmol) was employed with a 2-h reaction time, and recrystallization of crude product from water to obtain 2-(4-chloro-3-methylphenyl)-1,2-benzisoselenazol-3(2*H*)-one (**22**) (1.19 g, 3.69 mmol) as light beige shiny flakes with 74% yield, which melted at 208–209°C (from H_2O) (m.p. 209–210°C [12]). Selected FT-IR (ATR): ν_{max} 3088 (C_{Ar}-H), 3025 (C_{Ar}-H), 2918 (CH_3), 1586 (C=O), 1559, 1477, 1442, 1406, 1326 (C-N), 1308, 1260, 1135, 1044, 1021, 836, 812, 785, 727 (C-Se), 672, 561, 521, 482, 437 cm^{-1}; ^{1}H-NMR (600.6 MHz, DMSO-*d*$_6$): δ 8.09 (d, ^3J = 8.0 Hz, 1H, ArH-7), 7.90 (dd, ^3J = 7.7 Hz, ^4J = 1.1 Hz, 1H, ArH-4), 7.69 (ddd, *J*

= 8.0 Hz, ^3J = 7.2 Hz, ^4J = 1.1 Hz, 1H, ArH-6), 7.64 (d, ^4J = 2.4 Hz, 1H, PhH-2), 7.50 (dd, ^3J = 8.6 Hz, ^4J = 2.4 Hz, 1H, PhH-6), 7.48 (ddd, ^3J = 7.7 Hz, ^3J = 7.2 Hz, ^4J = 0.9 Hz, 1H, ArH-5), 7.47 (d, ^3J = 8.6 Hz, 1H, PhH-5), 2.37 (s, 3H, CH_3) ppm; ^{13}C-NMR (100.5 MHz, DMSO-d_6): δ 165.04 (C=O), 138.79 (CSe), 138.49 (PhC-1), 136.27 (PhC-3), 132.29 (ArC-6), 130.04 (CCl), 129.31 (PhC-5), 128.25 (ArC-3'), 127.90 (ArC-4), 126.98 (PhC-2), 126.23 (ArC-5), 125.79 (ArC-7), 123.66 (PhC-6), 19.62 (CH_3) ppm; ^{77}Se-NMR (76.24 MHz, DMSO-d_6): δ 919.71 ppm; The ^{1}H-NMR and ^{13}C-NMR spectra and HRMS analysis are consistent with the literature value [12].

2-(3,4-Dimethoxyphenyl)-1,2-benzisoselenazol-3(2*H*)-one (23)

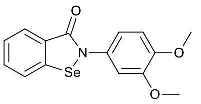

The general procedure starting from 3,4-dimethoxyaniline (0.766 g, 5.0 mmol) was employed with a 2-day reaction time, to obtain 2-(3,4-dimethoxyphenyl)-1,2-benzisoselenazol-3(2*H*)-one (**23**) (1.22 g, 3.65 mmol) as a pale powder with 73% yield. The analytically pure sample was obtained by rapid column chromatography on silica gel (70–230 mesh), using $CHCl_3$:EtOAc with gradient as an eluent, to product **23** (0.840 g, 2.51 mmol) as colorless crystals with 50% yield, which melted at 157–159°C (from EtOAc) (m.p. 159–161°C [1]). Selected FT-IR (ATR): ν_{max} 3053 (C_{Ar}-H), 2933 (CH_3), 2842 (CH_3), 1597 or 1585 (C=O), 1510, 1457, 1442, 1317 (C_{Ar}-N), 1233 (C-O), 1177, 1141, 1120, 1022 (C-O), 958, 822, 809, 766, 728 (C-Se), 613, 519, 483 cm^{-1}; ^{1}H-NMR (399.8 MHz, DMSO-d_6): δ 8.09 (dd, ^3J = 7.9 Hz, ^4J = 0.9 Hz, 1H, ArH-7), 7.89 (dd, ^3J = 7.7 Hz, ^4J = 1.1 Hz, 1H, ArH-4), 7.67 (ddd, ^3J = 7.9 Hz, ^3J = 7.3 Hz, ^4J = 1.1 Hz, 1H, ArH-6), 7.47 (ddd, ^3J = 7.7 Hz, ^3J = 7.3 Hz, ^4J = 0.9 Hz, 1H, ArH-5), 7.29 (d, ^4J = 2.1 Hz, 1H, PhH-2), 7.03 (dd, ^3J = 8.6 Hz, ^4J = 2.1 Hz, 1H, PhH-6), 7.00 (d, ^3J = 8.6 Hz, 1H, PhH-5), 3.78 (s, 3H, OCH_3), 3.78 (s, 3H, OCH_3) ppm; ^{13}C-NMR (100.5 MHz, DMSO-d_6): δ 164.91 (C=O), 148.66 (PhC-3), 147.07 (PhC-4), 139.04 (CSe), 132.44 (PhC-1), 132.00 (ArC-6), 128.37 (ArC-3'), 127.79 (ArC-4), 126.14 (ArC-5), 125.74 (ArC-7), 117.22 (PhC-6), 111.78 (PhC-5), 109.51 (PhC-2), 55.65 (OCH_3), 55.54 (OCH_3) ppm; ^{77}Se-NMR (76.24 MHz, DMSO-d_6): δ 917.51 ppm; The ^{1}H-NMR and ^{13}C-NMR spectra and HRMS analysis are consistent with the literature value [1].

Synthesis of ebselen open chain and their derivatives 24–34

Source of bis[2-(*N*-phenylcarbamoyl)phenyl]diselenide (**24**) was prepared from ebselen with hydrazine monohydrate ring opening in methanol solvent [13]. Derivatives of **24** – bis[2-(*N*-arylcarbamoyl)phenyl]diselenides **25–34** were synthesized from anthranilic acid and selenium

by 2,2’-dicarboxydiphenyl diselenide followed by *N*-acylation of two molecules of aniline derivatives with bis[(2-(chlorocarbonyl)phenyl] diselenide [1, 12, 18, 19]. The acylation reaction of appropriate anilines used in stoichiometric amounts in the presence of anhydrous sodium carbonate (Na_2CO_3) in anhydrous DCM (CH_2Cl_2) provided final products **25–34**. Mono- and disubstituted anilines were used as key aniline reagents. The aniline substituents used in different positions of the benzene ring were Me, CF_3, OMe, Cl and F. Compounds **24–34**, of which **30**, **34** are new, **25**, **27** are uncharacterized, and **26**, **29** are spectroscopically uncharacterized, were obtained. Purity of all compounds was determined and all new compounds were fully characterized.

Bis[2-(*N*-phenylcarbamoyl)phenyl]diselenide (24) [13]

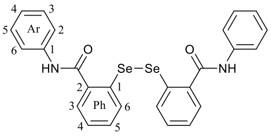

To the solution of 2-phenyl-1,2-benzisoselenazol-3(2*H*)-one (ebselen) (0.274 g, 1.0 mmol) in distilled methanol (4 mL) hydrazine monohydrate (0.05 mL, 1.0 mmol) in methanol (1 mL) was added while stirred, and the reaction was continued at gentle reflux for 30 min, then the reaction mixture was cooled to room temperature. The formed precipitate was filtered off, washed with methanol and dried to obtain colorless powder of **24** (0.272 g, 0.495 mmol) with 99% yield, which melted at 262–264°C (m.p. 263–265°C [5]). ^{1}H-NMR (399.8 MHz, DMSO-d_6): δ 10.57 (s, 2H, NH), 7.97 (dd, ^3J = 7.5 Hz, ^4J = 1.5 Hz, 2H, PhH-3), 7.80 (dd, ^3J = 7.0 Hz, ^4J = 1.2 Hz, 2H, PhH-6), 7.79 (d, ^3J = 8.0 Hz, 4H, ArH-2,6), 7.46 (ddd, ^3J = 8.0 Hz, ^3J = 7.0 Hz, ^4J = 1.5 Hz, 2H, PhH-5), 7.42 (ddd, ^3J = 8.0 Hz, ^3J = 7.5 Hz, ^4J = 1.2 Hz, 2H, PhH-4), 7.40 (dd, ^3J = 8.0 Hz, ^3J = 7.4 Hz, 4H, ArH-3,5), 7.16 (t, ^3J = 7.4 Hz, 2H, ArH-4) ppm; ^{13}C-NMR (100.5 MHz, DMSO-d_6): δ 166.28 (2 × C=O), 138.59 (2 × C – ArC-1), 133.74 (2 × C – PhC-2), 131.97 (2 × CH – PhC-5), 131.95 (2 × CSe), 130.13 (2 × CH – PhC-6), 128.66 (4 × CH – ArC-3,5), 128.59 (2 × CH – PhC-3), 126.36 (2 × CH – PhC-4), 124.08 (2 × CH – ArC-4), 120.51 (4 × CH – ArC-2,6) ppm; ^{77}Se-NMR (76.24 MHz, DMSO-d_6): δ 443.58 ppm. MS: *m/z* for $C_{26}H_{20}N_2O_2Se_2$ + H^+ calculated: 552.9928; found: 552.81; HRMS (TOF, MS, ESI) m/z for $C_{26}H_{20}N_2O_2Se_2$ + Na^+ calculated: 574.9747; found: 574.9760. The ^{1}H-NMR spectrum is in agreement with the literature data [13].

General procedure for synthesis of bis[2-(N-arylcarbamoyl)phenyl]diselenides 25–27, 29–30, 33–34 [1]

The solution of bis[(2-chlorocarbonyl)phenyl]diselenide (0.219 g, 0.50 mmol) in DCM (20 mL) was dropped to the mixture of substituted aniline (1.05 mmol) and powdered sodium carbonate (0.265 g, 2.5 mmol) in DCM (5 mL) during stirring for 30 min at room temperature, and the reaction was continued with TLC control. Every other 2–4 days, chloride (11–22 mg, 25–50 mmol) was added. When the reaction was completed, the product was filtered off, washed with DCM, 2.5% $NaHCO_3$ at brine, 3.5% aq. HCl, water and dried on the air to obtain diselenide product **25–34**. The product **26** was isolated by extraction with DCM and recrystallized from the same solvent. Crude products **25**, **30**, and **33–34** were recrystallized from dichloroethane ($ClCH_2CH_2Cl$).

Bis[2-(2-fluorophenylcarbamoyl)phenyl]diselenide (25) [11]

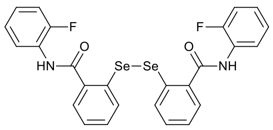

The general procedure starting from 2-fluoroaniline (0.117 g, 1.05 mmol) was employed with a 15-day reaction time, to obtain crude **25** as a pale powder which was recrystallized from $ClCH_2CH_2Cl$ (1400 mL/g) with slow concentration to obtain white powder of pure bis[2-(2-fluorophenylcarbamoyl)phenyl]diselenide (**25**) (0.198 g, 0.338 mmol) with 64% yield (calculated on 2-fluoroaniline used), which melted at 233–236°C (from $ClCH_2CH_2Cl$). Selected IR (KBr): ν 3265 (N-H), 3057 (C-H), 3012 (C-H), 1644 (C=O), 1522, 1505, 1438, 1317 (C-N), 1280, 1213, 1107, 1027, 914, 785, 758, 737, 689, 597, 455 cm^{-1}; ^{1}H-NMR (300 MHz, DMSO-d_6): δ 10.45 (s, 2H, NH), 8.03 (dd, ^3J = 7.5 Hz, ^4J = 1.4 Hz, 2H, PhH-3), 7.78 (dd, ^3J = 7.8 Hz, ^4J = 1.1 Hz, 2H, PhH-6), 7.62 (dd, J = 8.1 Hz, J = 6.9 Hz, 2H, ArH-6), 7.49 (ddd, ^3J = 7.8 Hz, ^3J = 7.3 Hz, ^4J = 1.4 Hz, 2H, PhH-5), 7.43 (ddd, ^3J = 7.5 Hz, ^3J = 7.3 Hz, ^4J = 1.1 Hz, 2H, PhH-4), 7.21–7.39 (m, 6H, ArH-3,4,5) ppm.^{1}H-NMR (600.6 MHz, DMSO-d_6): δ 10.46 (s, 2H, NH), 8.03 (d, ^3J = 7.5 Hz, 2H, PhH-3), 7.79 (d, ^3J = 8.0 Hz, 2H, PhH-6), 7.62 (dd, $^4J_{HF}$ = 7.8 Hz, ^3J = 7.6 Hz, 2H, ArH-6), 7.49 (ddd, ^3J = 8.0 Hz, ^3J = 7.3 Hz, 2H, PhH-5), 7.42 (dd, ^3J = 7.5 Hz, ^3J = 7.3 Hz, 2H, PhH-4), 7.34 (dd, $^3J_{HF}$ = 9.1 Hz, ^3J = 6.4 Hz, 2H, ArH-3), 7.33 (ddd, ^3J = 6.4 Hz, ^3J = 6.4 Hz, $^4J_{HF}$ = 6.4 Hz, 2H, ArH-4), 7.26 (ddd, ^3J = 7.6 Hz, ^3J = 6.4 Hz, ^4J = 2.7 Hz or $^5J_{HF}$ = 2.7 Hz, 2H, ArH) ppm; ^{13}C-NMR (100.5 MHz, DMSO-d_6): δ 166.47 (2 × C=O), 155.82 (d, $^1J_{CF}$ = 247.5 Hz, 2 × CF), 132.45 (2 × CSe or PhC-2), 132.43 (2 × CSe or PhC-2), 132.36 (2 × CH – PhC-5), 130.16 (2 × CH – PhC-6), 128.89 (2 × CH – PhC-3), 127.42 (d, $^3J_{CF}$ = 7.6 Hz, 2 × CH – ArC-6), 127.20 (2 × CH – ArC-4), 126.48 (2 × CH – PhC-4), 125.20 (d, $^2J_{CF}$ = 12.3 Hz, 2 × C – ArC-1), 124.43 (d, $^4J_{CF}$ = 3.3 Hz, 2 × CH – ArC-5), 115.96 (d, $^2J_{CF}$ = 19.8 Hz, 2 × CH – ArC-3) ppm; ^{19}F-NMR (376.2 MHz, DMSO-d_6): δ −120.518 (ddd, $^3J_{FH}$ = 9.1 Hz, $^4J_{FH}$

= 8.8 Hz, $^4J_{FH}$ = 6.4 Hz, 2F) ppm; ^{77}Se-NMR (76.24 MHz, DMSO-d_6): δ 447.22 ppm. ; HRMS (TOF, MS, ESI) m/z for $C_{26}H_{18}F_2N_2O_2Se_2$ + Na+ calculated: 610.9559; found: 610.9569.

Bis[2-(2-trifluoromethylphenylcarbamoyl)phenyl]diselenide (26) [5]

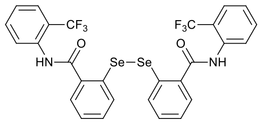

The general procedure starting from 2-trifluoromethylaniline (0.169 g, 1.05 mmol) was employed with a 19-day reaction time, with extraction with DCM, washing with 3.5% aq. HCl, water, brine and drying with anhydrous Na_2CO_3 to obtain crude diselenide product **26** (0.280 g, 0.408 mmol, 78%) which was repetitively crystallized from DCM to obtain white powder of pure bis[2-(2-fluoromethylphenylcarbamoyl)phenyl]diselenide (**26**) (0.235 g, 0.342 mmol) with 64% yield (calculated on 2-trifluoromethylaniline used), which melted at 228–229°C (from CH_2Cl_2) (m.p. 228–229°C [5]). Selected IR (KBr): ν 3282 (N-H), 3048 (C-H), 3009 (C-H), 1644 (C=O), 1589, 1527, 1456, 1320 (C-N), 1288, 1176, 1111, 1036, 763, 733, 654, 593, 468, 407 cm^{-1}; ^{1}H-NMR (399.8 MHz, DMSO-d_6): δ 10.65 (s, 2H, NH), 8.00 (dd, ^3J = 7.4 Hz, ^4J = 1.6 Hz, 2H, PhH-3), 7.85 (d, ^3J = 7.4 Hz, 2H, ArH), 7.75–7.83 (m, 4H, PhH), 7.55–7.65 (m, 4H, PhH), 7.51 (ddd, ^3J = 7.4 Hz, ^3J = 7.4 Hz, ^4J = 1.6 Hz, 2H, PhH), 7.43 (ddd, ^3J = 7.4 Hz, ^3J = 7.4 Hz, ^4J = 1.3 Hz, 2H, PhH) ppm; ^{1}H-NMR (399.8 MHz, DMSO-d_6): δ 10.52 (s, 2H, NH), 8.00 (d, ^3J = 7.3 Hz, 2H, PhH-3), 7.84 (d, ^3J = 7.8 Hz, 2H, ArH-3), 7.79 (d, ^3J = 7.8 Hz, 2H, PhH-6), 7.79 (dd, ^3J = 7.7 Hz, ^3J = 6.7 Hz, 2H, ArH-5), 7.60 (d, ^3J = 7.7 Hz, 2H, ArH-6), 7.59 (dd, ^3J = 7.3 Hz, ^3J = 6.7 Hz, 2H, ArH-4), 7.49 (ddd, ^3J = 7.8 Hz, ^3J = 7.4 Hz, ^4J = 1.6 Hz, 2H, PhH-5), 7.45 (ddd, ^3J = 7.4 Hz, ^3J = 7.3 Hz, ^4J = 1.3 Hz, 2H, PhH-4) ppm; ^{13}C-NMR (100.5 Hz, DMSO-d_6): δ 167.43 (2 × C=O), 135.17 (2 × C – ArC-1), 133.30 (2 × CH – ArC-5), 132.50 (2 × CSe), 132.39 (2 × C – PhC-2), 132.29 (2 × CH – PhC-5), 131.32 (2 × CH – ArC-6), 130.14 (2 × CH – PhC-6), 128.55 (2 × CH – PhC-3), 127.86 (2 × CH – ArC-4), 126.59 (k, $^3J_{CF}$ = 4.9 Hz, 2 × CH – ArC-3), 126.51 (k, $^2J_{CF}$ = 29.4 Hz, 2 × C – ArC-2), 126.48 (2 × CH – PhC-4), 123.56 (k, $^1J_{CF}$ = 273.5 Hz, 2 × CF_3) ppm; ^{19}F-NMR (376.2 MHz, DMSO-d_6): δ −59.170 (2 × CF_3) ppm; ^{77}Se-NMR (76.24 MHz, DMSO-d_6): δ 445.64 ppm. HRMS (TOF, MS, ESI) m/z for $C_{28}H_{18}F_6N_2O_2Se_2$ + Na^+ calculated: 710.9495; found: 710.9492.

Bis[2-(3-fluorophenylcarbamoyl)phenyl]diselenide (27) [11]

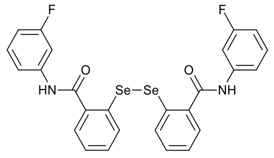

The general procedure starting from 3-fluoroaniline (0.117 g, 1.05 mmol) was employed with a 7 day reaction time, to obtain pure bis[2-(3-fluorophenylcarbamoyl)phenyl]diselenide (**27**) (0.283 g, 0.483 mmol) as a white powder with 92% yield (calculated on 3-fluoroaniline used), which melted at 239–240°C. Selected IR (KBr): ν 3291 (N-H), 3055 (C-H), 1652 (C=O), 1638, 1603, 1490, 1438, 1317 (C-N), 1261, 1150, 1027, 858, 775, 734, 695, 677, 597, 521, 460 cm^{-1}; ^{1}H-NMR (399.8 MHz, DMSO-d_6): δ 10.75 (s, 2H, NH), 7.97 (dd, ^{3}J = 7.5 Hz, ^{4}J = 1.3 Hz, 2H, PhH-3), 7.81 (d, ^{3}J = 7.8 Hz, 2H, PhH-6), 7.74 (ddd, $^{3}J_{HF}$ = 11.6 Hz, ^{4}J = 2.4 Hz, ^{4}J = 1.7 Hz, 2H, ArH-2), 7.59 (d, ^{3}J = 8.6 Hz, 2H, ArH-6), 7.48 (ddd, ^{3}J = 7.8 Hz, ^{3}J = 7.1 Hz, ^{4}J = 1.3 Hz, 2H, PhH-5), 7.40–7.47 (m, 4H, PhH-4, ArH-5), 6.99 (ddd, $^{3}J_{HF}$ = 8.5 Hz, ^{3}J = 8.5 Hz, ^{4}J = 2.4 Hz, 2H, ArH-4) ppm; ^{13}C-NMR (100.5 MHz, DMSO-d_6): δ 166.51 (2 × C=O), 161.99 (d, $^{1}J_{CF}$ = 241.6 Hz, 2 × CF), 140.35 (d, $^{3}J_{CF}$ = 10.96 Hz, 2 × C – ArC-1), 133.43 (2 × C – PhC-2), 132.20 (2 × CH – PhC-5), 132.00 (2 × CSe), 130.29 (d, $^{3}J_{CF}$ = 14.9 Hz, 2 × CH – ArC-5), 130.27 (2 × CH – PhC-6), 128.71 (2 × CH – PhC-3), 126.43 (2 × CH – PhC-4), 116.16 (2 × CH – ArC-6), 110.55 (d, $^{2}J_{CF}$ = 21.0 Hz, 2 × CH – ArC-4), 107.15 (d, $^{2}J_{CF}$ = 26.2 Hz, 2 × CH – ArC-2) ppm; ^{19}F-NMR (376.2 MHz, DMSO-d_6): δ −111.909 (ddd, $^{3}J_{FH}$ = 11.6 Hz, $^{3}J_{FH}$ = 8.5 Hz, $^{4}J_{FH}$ = 7.1 Hz, 2F) ppm; ^{77}Se-NMR (76.24 MHz, DMSO-d$_6$): δ 444.64 ppm. HRMS (TOF, MS, ESI) m/z for $C_{26}H_{18}F_2N_2O_2Se_2 + Na^+$ calculated: 610.9559; found: 610.9594.

Bis[2-(3-methoxyphenylcarbamoyl)phenyl]diselenide (28) [1, 12]

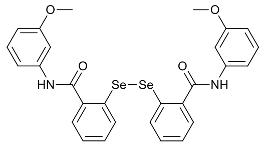

Compound was prepared according to the references [1, 12], bis[2-(3-methoxyphenylcarbamoyl)phenyl]diselenide (**28**) was obtained with 98% yield as colorless powder of which melted at 220–222°C (from $CHCl_3$/EtOAc, 1/2, *v/v*) (m.p. 221–223°C [12]). ^{1}H-NMR (600.6 MHz, DMSO-d_6): δ 10.54 (s, 2H, NH), 7.95 (dd, ^{3}J = 7.6 Hz, ^{4}J = 1.4 Hz, 2H, PhH-3), 7.81 (dd, ^{3}J = 8.0 Hz, ^{4}J = 1.1 Hz, 2H, PhH-6), 7.46 (ddd, ^{3}J = 8.0 Hz, ^{3}J = 7.2 Hz, ^{4}J = 1.4 Hz, 2H, PhH-5), 7.45 (s, 2H, ArH-2), 7.42 (ddd, ^{3}J = 7.6 Hz, ^{3}J = 7.2 Hz, ^{4}J = 1.1 Hz, 2H, PhH-4), 7.39 (dd, ^{3}J = 8.0 Hz, ^{4}J = 1.0 Hz, 2H, ArH-6), 7.30 (dd, ^{3}J = 8.0 Hz, ^{3}J = 8.0 Hz, 2H, ArH-5), 6.74 (d, ^{3}J = 8.0 Hz, 2H, ArH-4), 3.78 (s, 6H, OCH_3) ppm; ^{13}C-NMR (151.0 Hz, DMSO-d_6): δ 166.30 (C=O), 159.40 (2 × C – ArC-3), 139.77 (2 × C – ArC-1), 133.75 (2 × C – PhC-2), 131.97 (2 × CH – PhC-5), 131.92 (2 × CSe), 130.13 (2 × CH – PhC-6), 129.45 (2 × CH – ArC-5), 128.57 (2 × CH – PhC-3), 126.34 (2 × CH – PhC-4), 112.70 (2 × CH – ArC-6), 109.51 (2 × CH – ArC-4), 106.22 (2 × CH – ArC-2), 55.00 (OCH_3) ppm; ^{77}Se-NMR (76.24 MHz, DMSO-d_6): δ 443.48 ppm; MS: *m/z* for

$C_{28}H_{24}N_2O_2Se_2 + H^+$ calculated: 613.0139; found: 612.89. The ^{1}H-NMR, and ^{13}C-NMR spectra are in agreement with the literature data [12].

Bis[2-(4-trifluoromethylphenylcarbamoyl)phenyl]diselenide (29) [5]

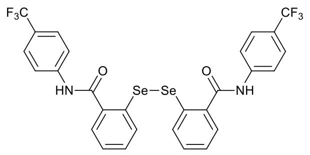

The general procedure starting from 4-trifluoromethylaniline (0.169 g, 1.05 mmol) was employed with a 14-day reaction time, to obtain bis[2-(4-trifluoromethylphenylcarbamoyl)phenyl]diselenide (**29**) (0.326 g, 0.475 mmol) as white powder with 90% yield (calculated on 4-trifluoromethylaniline used), which melted at 261–264°C (m.p. 273°C [5]). Selected IR (KBr): ν 3316 (N-H), 3052 (C-H), 1650 (C=O), 1600, 1524, 1516, 1410, 1323 (C-N), 1159, 1121, 1068, 839, 744, 696, 592, 510 cm^{-1}; ^{1}H-NMR (399.8 MHz, DMSO-*d*$_6$): δ 10.91 (s, 2H, NH), 8.02 (d, ^3J = 7.4 Hz, 2H, PhH-3), 8.02 (d, ^3J = 8.7 Hz, 4H, ArH-3,5), 7.82 (d, ^3J = 7.9 Hz, 2H, PhH-6), 7.77 (d, ^3J = 8.7 Hz, 4H, ArH-2,6), 7.49 (ddd, ^3J = 7.9 Hz, ^3J = 7.3 Hz, ^4J = 1.4 Hz, 2H, PhH-5), 7.44 (ddd, ^3J = 7.4 Hz, ^3J = 7.3 Hz, ^4J = 1.4 Hz, 2H, PhH-4) ppm; ^{13}C-NMR (100.5 MHz, DMSO-*d*$_6$): δ 166.74 (2 × C=O), 142.28 (2 × C – ArC-1), 133.27 (2 × C – PhC-2), 132.34 (2 × CH – PhC-5), 132.15 (2 × CSe), 130.26 (2 × CH – PhC-6), 128.92 (2 × CH – PhC-3), 126.46 (2 × CH – PhC-4), 125.62 (k, $^3J_{CF}$ = 2.7 Hz, 2 × CH – ArC-3,5), 124.28 (k, $^1J_{CF}$ = 271.4 Hz, 2 × CF_3), 124.03 (k, $^2J_{CF}$ = 32.2 Hz, 2 × C – ArC-4), 120.36 (2 × CH – ArC-2,6) ppm; ^{19}F-NMR (376.2 MHz, DMSO-*d*$_6$): δ −60.290 ppm; ^{77}Se-NMR (76.24 MHz, DMSO-*d*$_6$): δ 445.89 ppm; HRMS (TOF, MS, ESI) m/z for $C_{28}H_{18}F_6N_2O_2Se_2 + H^+$ calculated: 688.9676; found: 688.9683.

Bis[2-(4-chloro-2-fluorophenylcarbamoyl)phenyl]diselenide (30)

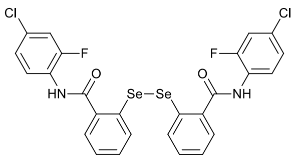

The general procedure starting from 4-chloro-2-fluoroaniline (0.153 g, 1.05 mmol) was employed with a 6-day reaction time, during which a violet color formation was observed. Crude **30** (0.259 g, 0.395 mmol) was obtained as a pale violet solid with 75% yield (calculated on 4-chloro-2-fluoroaniline used) and was recrystallized from $ClCH_2CH_2Cl$ (175 mL/g), before decolorization with charcoal, with slow concentration to obtain pale powder of pure bis[2-(4-chloro-2-fluorophenylcarbamoyl)phenyl]diselenide (**30**) (0.242 g, 0.369 mmol) with 70% yield, which melted at 232–233°C (from $ClCH_2CH_2Cl$). Selected FT-IR (KBr): ν 3253 (N-H), 3052 (C-H), 3017 (C-H), 1647 (C=O), 1595, 1513, 1405, 1316 (C-N), 1254, 1218, 1119, 1027, 895, 830, 739, 699, 660, 421 cm^{-1}; ^{1}H-NMR (300.1 MHz, DMSO-*d*$_6$): δ 10.61 (s, 2H, NH), 8.02 (dd, ^3J

= 7.5 Hz, ^4J = 1.4 Hz, 2H, PhH-3), 7.78 (dd, ^3J = 7.8 Hz, ^4J = 1.1 Hz, 2H, PhH-6), 7.68 (dd, ^3J = 8.6 Hz, $^4J_{HF}$ = 8.4 Hz, 2H, ArH-6), 7.58 (dd, $^3J_{HF}$ = 10.3 Hz, ^4J = 2.3 Hz, 2H, ArH-3), 7.49 (ddd, ^3J = 7.8 Hz, ^3J = 7.3 Hz, ^4J = 1.4 Hz, 2H, PhH-5), 7.43 (ddd, ^3J = 7.5 Hz, ^3J = 7.3 Hz, ^4J = 1.1 Hz, 2H, PhH-4), 7.37 (ddd, ^3J = 8.6 Hz, ^4J = 2.3 Hz, $^5J_{HF}$ = 1.1 Hz, 2H, ArH-5) ppm; ^{13}C-NMR (75.48 MHz, DMSO-d_6): δ 166.40 (2 × C=O), 155.50 (2 × CF), 132.44 (2 × CH, 2 × C), 132.14 (2 × C), 130.34 (d, ^3J = 9.9 Hz, 2 × C), 130.13 (2 × CH), 128.94 (2 × CH), 128.01 (d, ^3J = 1.8 Hz, 2 × CH), 126.43 (2 × CH), 124.60 (d, ^4J = 3.6 Hz, 2 × CH), 124.49 (d, $^2J_{CF}$ = 13.7 Hz, 2 × C), 116.56 (d, ^2J = 23.7 Hz, 2 × CH) ppm; ^{1}H-NMR (399.8 MHz, DMSO-d_6): δ 10.54 (s, 2H, NH), 8.02 (dd, ^3J = 7.6 Hz, ^4J = 1.4 Hz, 2H, PhH-3), 7.78 (dd, ^3J = 7.9 Hz, ^4J = 1.0 Hz, 2H, PhH-6), 7.68 (dd, ^3J = 8.6 Hz, $^4J_{HF}$ = 8.3 Hz, 2H, ArH-6), 7.58 (dd, $^3J_{HF}$ = 10.3 Hz, ^4J = 2.3 Hz, 2H, ArH-3), 7.49 (ddd, ^3J = 7.9 Hz, ^3J = 7.3 Hz, ^4J = 1.4 Hz, 2H, PhH-5), 7.43 (ddd, ^3J = 7.6 Hz, ^3J = 7.3 Hz, ^4J = 1.0 Hz, 2H, PhH-4), 7.36 (ddd, ^3J = 8.6 Hz, ^4J = 2.3 Hz, $^5J_{HF}$ = 1.1 Hz, 2H, ArH-5) ppm; ^{13}C-NMR (100.5 MHz, DMSO-d_6): δ 166.43 (2 × C=O), 155.52 (d, $^1J_{CF}$ = 251.1 Hz, 2 × CF), 132.45 (2 × CH – PhC-5), 132.44 (2 × CSe), 132.17 (2 × C – PhC-2), 130.36 (d, $^3J_{CF}$ = 9.9 Hz, 2 × CCl), 130.16 (2 × CH – PhC-6), 128.95 (2 × CH – PhC-3), 128.02 (d, $^3J_{CF}$ = 1.8 Hz, 2 × CH – ArC-6), 126.45 (2 × CH – PhC-4), 124.62 (d, $^4J_{CF}$ = 3.2 Hz, 2 × CH – ArC-5), 124.49 (d, $^2J_{CF}$ = 12.4 Hz, 2 × C – ArC-1), 116.58 (d, ^2J = 23.7 Hz, 2 × CH – ArC-3) ppm; δ −117.034 (dd, $^3J_{FH}$ = 10.3 Hz, $^4J_{FH}$ = 8.3 Hz, 2F) ppm; ^{77}Se-NMR (76.24 MHz, DMSO-d_6): δ 447.93 ppm; HRMS (TOF, MS, ESI) m/z for $C_{26}H_{16}Cl_2F_2N_2O_2Se_2$ + Na^+ calculated: 678.8780; found: 678.8778.

Bis[2-(2,4-dimethoxyphenylcarbamoyl)phenyl]diselenide (31) [1]

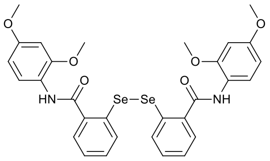

Preparation according to the reference [1], bis[2-(2,4-dimethoxyphenylcarbamoyl)phenyl]diselenide (**31**) was obtained as a white powder with 98% yield, which melted at 211–214°C (from CH_2Cl_2) (m.p. 211.0–213.5°C [1]). ^{1}H-NMR (399.8 MHz, DMSO-d_6): δ 9.75 (s, 2H, NH), 8.01 (d, ^3J = 7.4 Hz, 2H, PhH-3), 7.76 (d, ^3J = 7.9 Hz, 2H, PhH-6), 7.48 (d, ^3J = 8.7 Hz, 2H, ArH-6), 7.44 (dd, ^3J = 7.9 Hz, ^3J = 7.1 Hz, 2H, PhH-5), 7.38 (dd, ^3J = 7.4 Hz, ^3J = 7.1 Hz, 2H, PhH-4), 6.69 (d, ^4J = 2.6 Hz, 2H, ArH-3), 6.58 (dd, ^3J = 8.7 Hz, ^4J = 2.6 Hz, 2H, ArH-5), 3.82 (s, 6H, 2-OCH_3), 3.79 (s, 6H, 4-OCH_3) ppm; ^{13}C-NMR (100.5 MHz, DMSO-d_6): δ 166.20 (2 × C=O), 158.28 (2 × C – ArC-4), 153.73 (2 × C – ArC-2), 132.96 (2 × C – PhC-2), 132.30 (2 × CSe), 131.87 (2 × CH – PhC-5), 129.99 (2 × CH – PhC-6), 128.38 (2 × CH – PhC-3), 126.75 (2 × CH – ArC-6), 126.28 (2 × CH – PhC-4), 119.08 (2 × C – ArC-1), 104.26 (2 × CH – ArC-

5), 98.95 (2 × CH – ArC-3), 55.69 (2 × 2-OCH_3), 55.33 (2 × 4-OCH_3) ppm; ^{77}Se-NMR (76.24 MHz, DMSO-d_6): δ 446.62 ppm; MS: *m/z* for $C_{30}H_{28}N_2O_6Se_2$ + H^+ calculated: 673.0351; found: 672.93. FT-IR, ^{1}H-NMR and ^{13}C-NMR spectra are in agreement with the literature data [1].

Bis[2-(5-chloro-2-methylphenylcarbamoyl)phenyl]diselenide (32) [12]

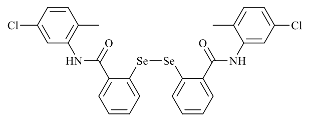

Preparation according to the reference [12] with a 6-day reaction time. Bis[2-(5-chloro-2-methylphenylcarbamoyl)phenyl]diselenide (**32**) was obtained as a white powder with 86% yield, which melted at 248–249°C (from $ClCH_2CH_2Cl$) (m.p. 247–248°C [12]). Selected FT-IR (KBr): ν 3250 (NH), 3047 (C_{Ar}-H), 2969 (CH_3), 1631 (C=O), 1520, 1500, 1482, 1436, 1306, 1280, 1253, 1026, 858, 810, 739, 653, 597, 456, 447 cm^{-1}; ^{1}H-NMR (600.6 MHz, DMSO-d_6): δ 10.28 (s, 2H, NH), 7.80 (dd, ^3J = 7.1 Hz, ^4J = 1.4 Hz, 2H, PhH-3), 7.80 (dd, ^3J = 8.0 Hz, ^4J = 1.0 Hz, 2H, PhH-6), 7.53 (d, ^4J = 2.1 Hz, 2H, ArH-6), 7.48 (ddd, ^3J = 8.0 Hz, ^3J = 7.5 Hz, ^4J = 1.4 Hz, 2H, PhH-5), 7.43 (ddd, ^3J = 7.5 Hz, ^3J = 7.1 Hz, ^4J = 1.0 Hz, 2H, PhH-4), 7.34 (d, ^4J = 8.3 Hz, 2H, ArH-3), 7.28 (dd, ^3J = 8.3 Hz, ^4J = 2.1 Hz, 2H, ArH-4), 2.28 (s, 6H, CH_3) ppm; ^{13}C-NMR (100.5 MHz, DMSO-d_6): δ 166.47 (2 × C=O), 137.23 (2 × C – ArC-1), 132.93 (2 × C – PhC-2), 132.51 (2 × CH – PhC-5), 132.27 (2 × CSe), 132.12 (2 × C – ArC-2), 131.89 (2 × CH – ArC-3), 130.13 (2 × CH – PhC-6), 129.84 (2 × CCl), 128.63 (2 × CH – PhC-3), 126.44 (2 × CH – PhC-4), 125.96 (2 × CH – ArC-4), 125.86 (2 × CH – ArC-6), 17.39 (2 × CH_3) ppm; ^{77}Se-NMR (76.24 MHz, DMSO-d_6): δ 445.56 ppm; MS: *m/z* for $C_{28}H_{22}Cl_2N_2O_2Se_2$ + H^+ calculated: 648.9462; found: 648.81. ^{1}H-NMR and ^{13}C-NMR spectra are in agreement with the literature data [12].

Bis[2-(4-chloro-3-methylphenylcarbamoyl)phenyl]diselenide (33) [12]

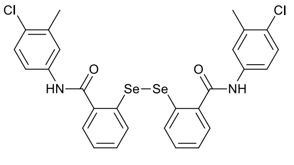

The general procedure starting from 4-chloro-3-methylaniline (0.149 g, 1.05 mmol) was employed with a 6-day reaction time, to obtain bis[2-(4-chloro-3-methylphenylcarbamoyl)phenyl]diselenide (**33**) (0.290 g, 0.448 mmol) with 85% yield (calculated on 4-chloro-3-methylaniline used) as a white powder which melted at 242–243°C. Analytical sample was obtained by recrystallization from $ClCH_2CH_2Cl$ (600 mL/g), with slow concentration to 50 mL volume, to obtain **33** (0.238 g, 0.368 mmol) as a pale powder with 70% yield, which melted at 246–248°C (m.p. 239–240°C [12]). Selected FT-IR (KBr): ν 3278 (NH), 3058 (C_{Ar}-H), 2983 (CH_3), 1636 (C=O), 1520, 1483, 1402, 1313 (C-N), 1254, 1046, 868, 812, 741, 691, 571, 445 cm^{-1}; ^{1}H-NMR (399.8 MHz,

DMSO-d_6): δ 10.25 (s, 2H, NH), 8.01 (d, ^3J = 7.1 Hz, 2H, PhH-3), 7.79 (dd, ^3J = 7.9 Hz, ^4J = 1.1 Hz, 2H, PhH-6), 7.47 (ddd, ^3J = 7.9 Hz, ^3J = 7.3 Hz, ^4J = 1.5 Hz, 2H, PhH-5), 7.42 (ddd, ^3J = 7.3 Hz, ^3J = 7.1 Hz, ^4J = 1.1 Hz, 2H, PhH-4), 7.42 (d, ^4J = 8.4 Hz, 2H, ArH-5), 7.41 (d, ^4J = 2.5 Hz, 2H, ArH-2), 7.32 (dd, ^3J = 8.4 Hz, ^4J = 2.5 Hz, 2H, ArH-6), 2.28 (s, 6H, CH_3) ppm; ^{13}C-NMR (100.5 MHz, DMSO-d_6): δ 166.48 (2 × C=O), 136.18 (2 × C – ArC-1 or ArC-4), 134.90 (2 × C – ArC-1 or ArC-4), 132.97 (2 × C – PhC-2), 132.27 (2 × C – CSe), 132.08 (2 × CH – PhC-5), 130.26 (2 × C – ArC-3), 130.12 (2 × CH – PhC-6), 129.95 (2 × CH – ArC-2), 128.56 (2 × CH – PhC-3), 128.15 (2 × CH – ArC-5), 126.43 (2 × CH – PhC-4), 125.98 (2 × CH – ArC-6), 17.72 (2 × CH_3) ppm; ^{77}Se-NMR (76.24 MHz, DMSO-d_6): δ 445.63 ppm; MS: *m/z* for $C_{28}H_{22}Cl_2N_2O_2Se_2$ + H^+ calculated: 648.9462; found: 648.83.

Bis[2-(5-chloro-2-fluorophenylcarbamoyl)phenyl]diselenide (34)

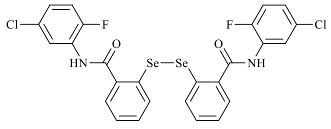

The general procedure starting from 5-chloro-2-fluoroaniline (0.153 g, 1.05 mmol) was employed with a 7-day reaction time, to obtain white powder of crude **34** (0.290 g, 0.448 mmol) almost quantitatively by recrystallization from $ClCH_2CH_2Cl$ (250 mL/g) in open vessel, to obtain a pale powder of bis[2-(5-chloro-2-fluorophenylcarbamoyl)phenyl]diselenide (**34**) (0.290 g, 0.44 mmol) with 84% yield (calculated on 5-chloro-2-fluoroaniline used), which melted at 218–219°C (by repetitive crystallization from $ClCH_2CH_2Cl$). Selected FT-IR (KBr): ν 3299 (N-H), 3081 (C-H), 3050 (C-H), 1645 (C=O), 1609, 1529, 1482, 1417, 1313 (C-N), 1252, 1198, 862, 810, 736, 654, 454 cm^{-1}; ^{1}H-NMR (600.6 MHz, DMSO-d_6): δ 10.58 (s, 2H, NH), 8.02 (dd, ^3J = 7.7 Hz, ^4J = 1.3 Hz, 2H, PhH-3), 7.78 (d, ^3J = 8.1 Hz, 2H, PhH-6), 7.7 (d, $^4J_{HF}$ = 8.9 Hz, 2H, ArH-6), 7.50 (ddd, ^3J = 8.1 Hz, ^3J = 7.2 Hz, ^4J = 1.3 Hz, 2H, PhH-5), 7.43 (ddd, ^3J = 7.7 Hz, ^3J = 7.2 Hz, ^3J = 1.1 Hz, 2H, PhH-4), 7.41 (dd, $^3J_{HF}$ = 9.6 Hz, ^3J = 8.9 Hz, 2H, ArH-3), 7.38 (ddd, ^3J = 8.9 Hz, $^4J_{HF}$ = 4.4 Hz, ^4J = 2.5 Hz, 2H, ArH-4) ppm; ^{1}H-NMR (300.1 MHz, DMSO-d_6): δ 10.59 (s, 2H, NH), 8.01 (dd, J = 7.5 Hz, J = 1.3 Hz, 2H, PhH), 7.75–7.85 (m, 4H, ArH, PhH), 7.50 (ddd, J = 7.6 Hz, J = 7.6 Hz, J = 1.3 Hz, 2H, PhH), 7.38–7.47 (m, 6H, ArH and PhH) ppm; ^{13}C-NMR (75.48 MHz, DMSO-d_6): δ 166.48 (2 × C=O), 154.20 (d, J_{CF} = 247.9 Hz, 2 × CF), 132.47 (2 × CH), 132.44 (2 × C), 132.15 (2 × C), 130.16 (2 × CH), 129.03 (2 × CH), 127.77 (d, J_{CF} = 3.1 Hz, 2 × CCl), 126.71 (d, J_{CF} = 7.8 Hz, 2 × CH), 126.69 (d, J_{CF} = 13.3 Hz, 2 × C), 126.45 (2 × CH), 126.10 (2 × CH), 117.54 (d, $^2J_{CF}$ = 21.8 Hz, 2 × CH) ppm; ^{13}C-NMR (151.0 MHz, DMSO-d_6): δ 166.50 (2 × C=O), 154.22 (d, $^1J_{CF}$ = 247.8 Hz, 2 × CF), 132.48 (2 × CH – PhC-5), 132.42 (2 × CSe or PhC-2), 132.18 (2 × CSe or PhC-2), 130.18 (2 × CH – PhC-6),

129.02 (2 × CH – PhC-3), 127.78 (d, $^{4}J_{CF}$ = 3.2 Hz, 2 × CCl), 126.73 (d, $^{3}J_{CF}$ = 7.7 Hz, 2 × CH – ArC-4), 126.69 (d, $^{2}J_{CF}$ = 13.9 Hz, 2 × C – ArC-1), 126.48 (2 × CH – PhC-4), 126.11 (d, $^{3}J_{CF}$ = 1.4 Hz, 2 × CH – PhC-6), 117.56 (d, $^{2}J_{CF}$ = 21.7 Hz, 2 × CH – ArC-3) ppm; ^{19}F-NMR (376.2 MHz, DMSO-d_6): δ −122.398 (ddd, $^{3}J_{FH}$ = 9.6 Hz, $^{4}J_{FH}$ = 8.9 Hz, $^{4}J_{FH}$ = 4.4 Hz, 2F) ppm; ^{77}Se-NMR (76.24 MHz, DMSO-d_6): δ 447.56 ppm; HRMS (TOF, MS, ESI) m/z for $C_{26}H_{16}Cl_2F_2N_2O_2Se_2$ + Na^+ calculated: 678.8780; found: 678.8787.

Mass spectra and analytical chromatograms of the compounds

The purity and molecular weight of each compound was confirmed with LC-MS system (Waters e2695 Separations Module, 2489 UV/Vis Detector, Acquity QDa MS Detector). Analytical HPLC column: Jupiter 10 mm C4 300 Å (250x4.6 mm). Solvent composition: phase A (water/0.1% HCOOH) and phase B (MeCN/0.1% HCOOH); gradient from 5% B to 95% B over a period of 15 min.

2-phenylbenzisoselenazol-3(2*H*)-one (ebselen)

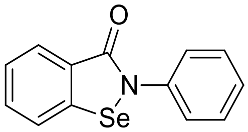

(MS: for $C_{13}H_9NOSe$ + H+ m/z $_{calcd}$ = 275.9923; m/z $_{found}$ = 275.87)

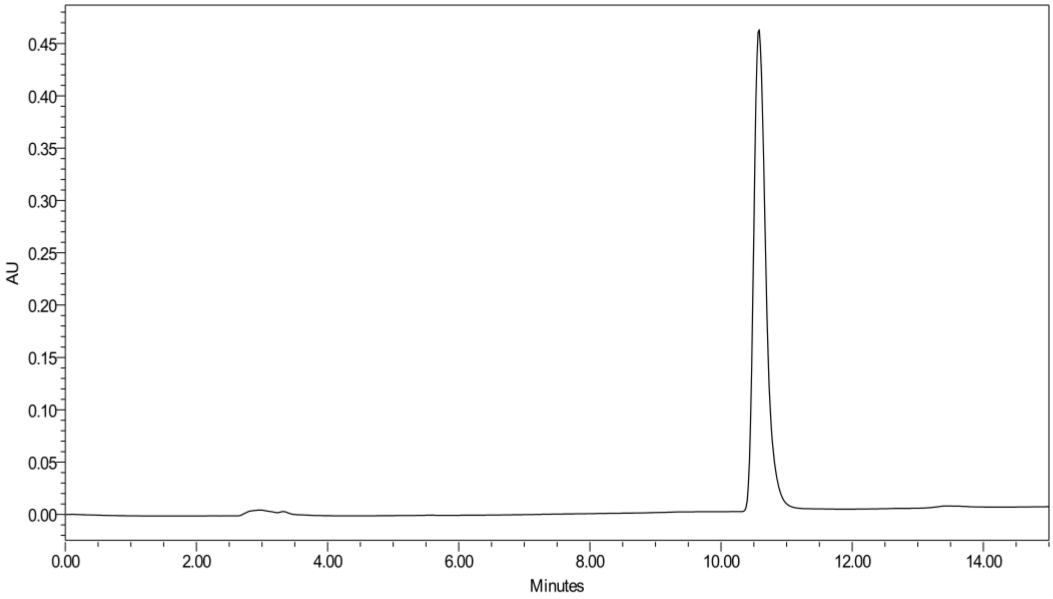

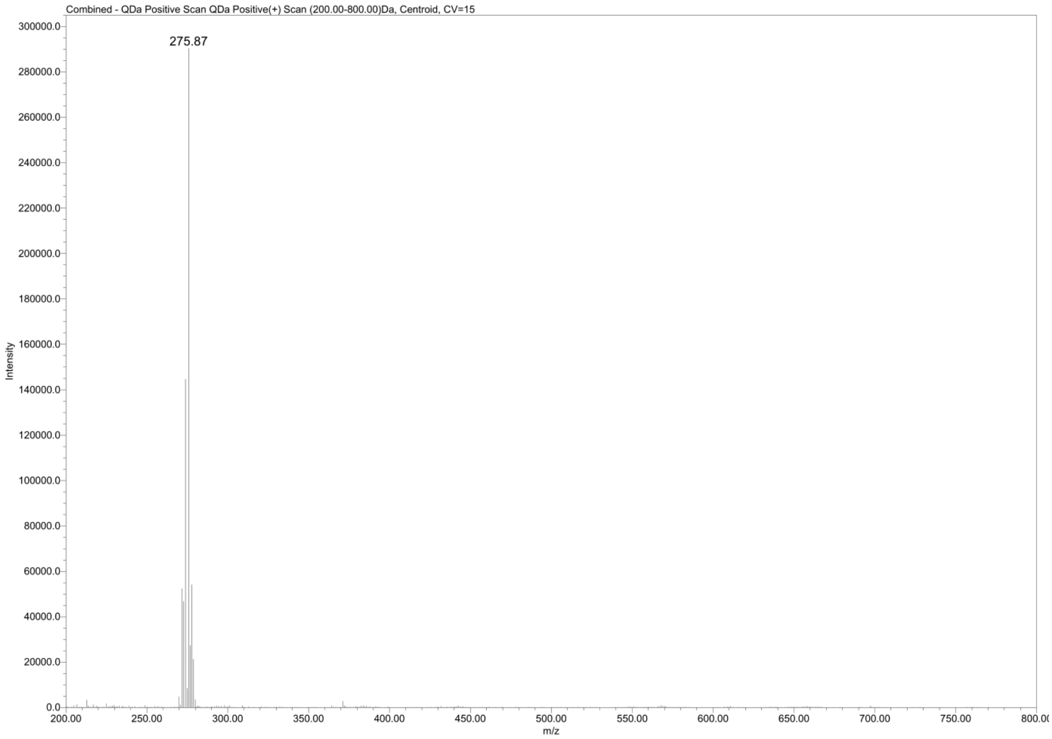

Compound 1

2-(2-fluorophenyl)-benzisoselenazol-3(2*H*)-one

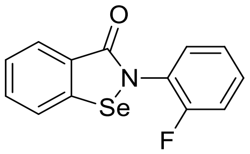

(MS: for $C_{13}H_8FNOSe + H^+$ m/z $_{calcd}$ = 293.9828; m/z $_{found}$ = 293.81)

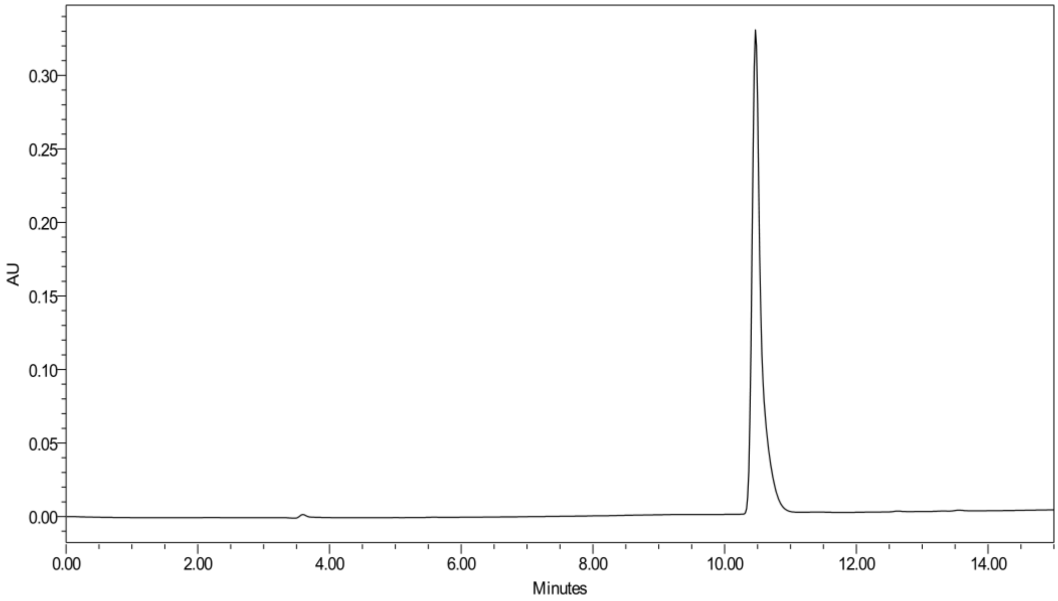

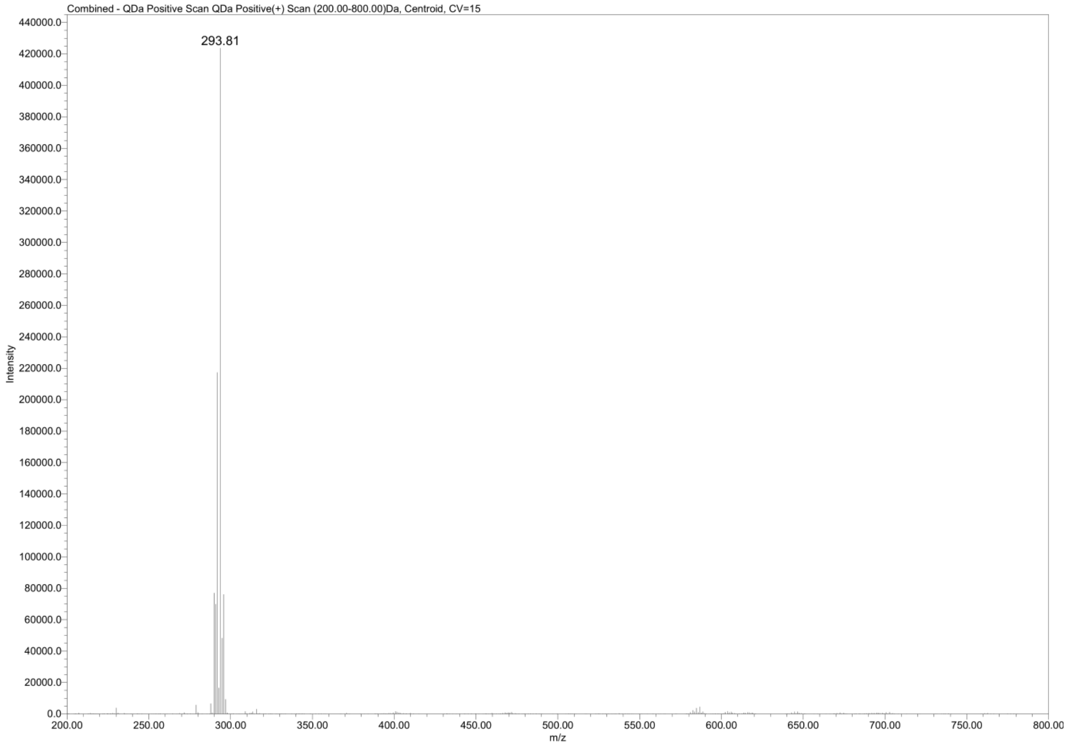

Compound 2

2-(2-chlorophenyl)-benzisoselenazol-3(2*H*)-one

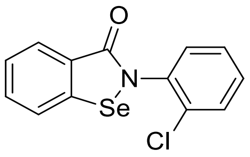

(MS: for $C_{13}H_8ClNOSe + H^+$ m/z $_{calcd}$ = 309.9533; m/z $_{found}$ = 309.79)

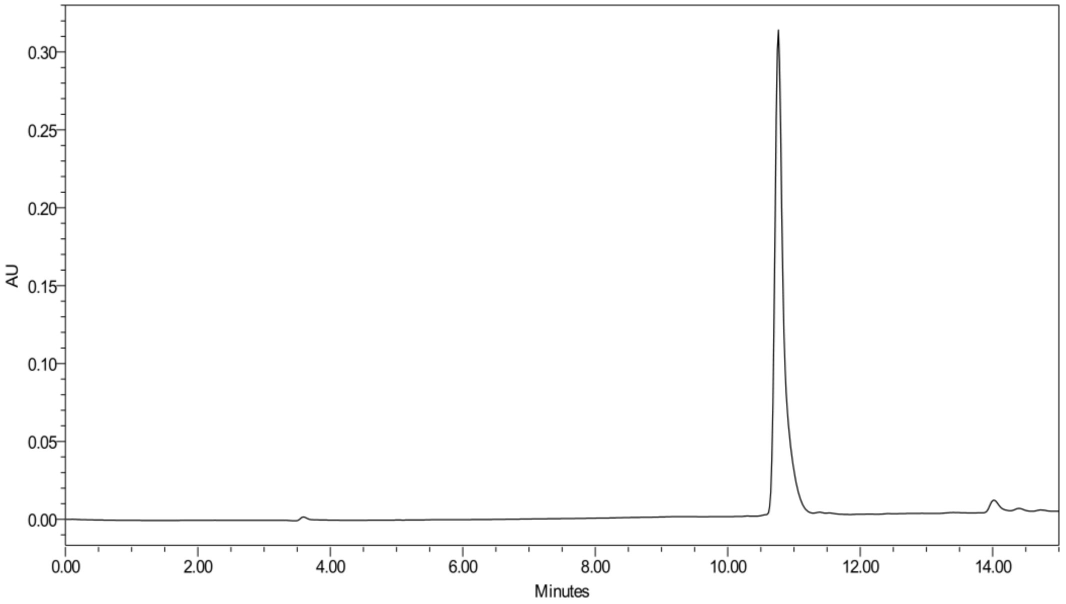

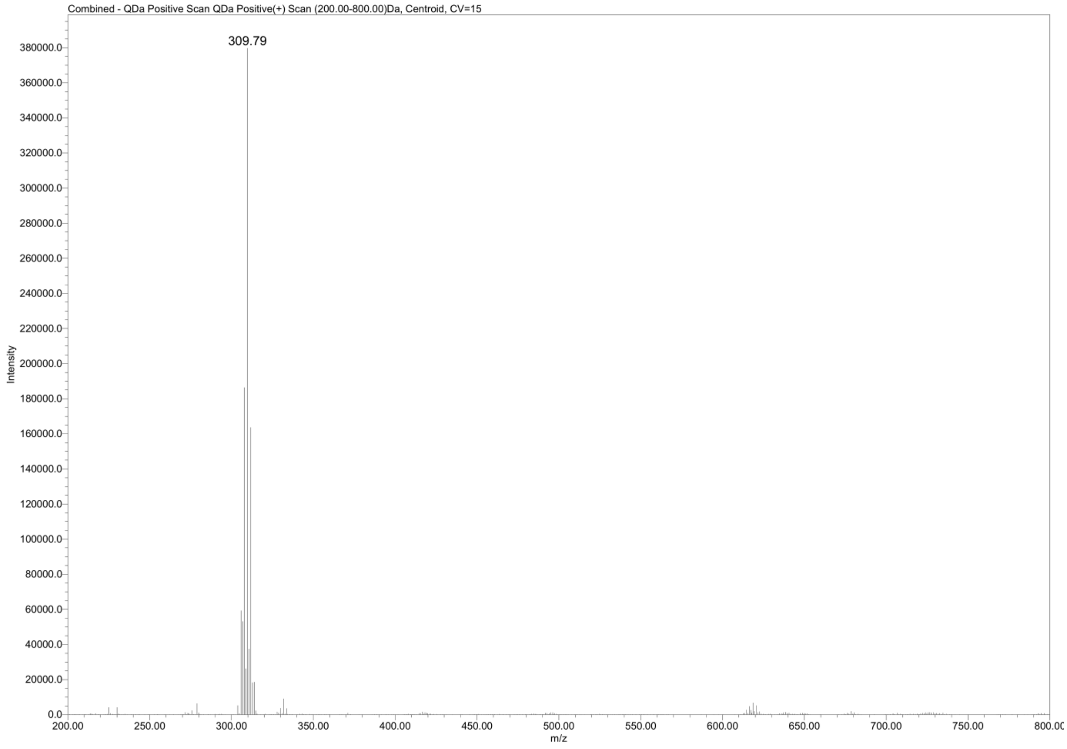

Compound 3

2-(2-bromophenyl)-benzisoselenazol-3(2*H*)-one

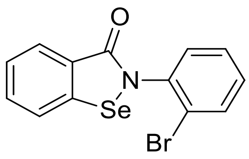

(MS: for $C_{13}H_8BrNOSe + H^+$ m/z $_{calcd}$ = 353.9028; m/z $_{found}$ = 353.74)

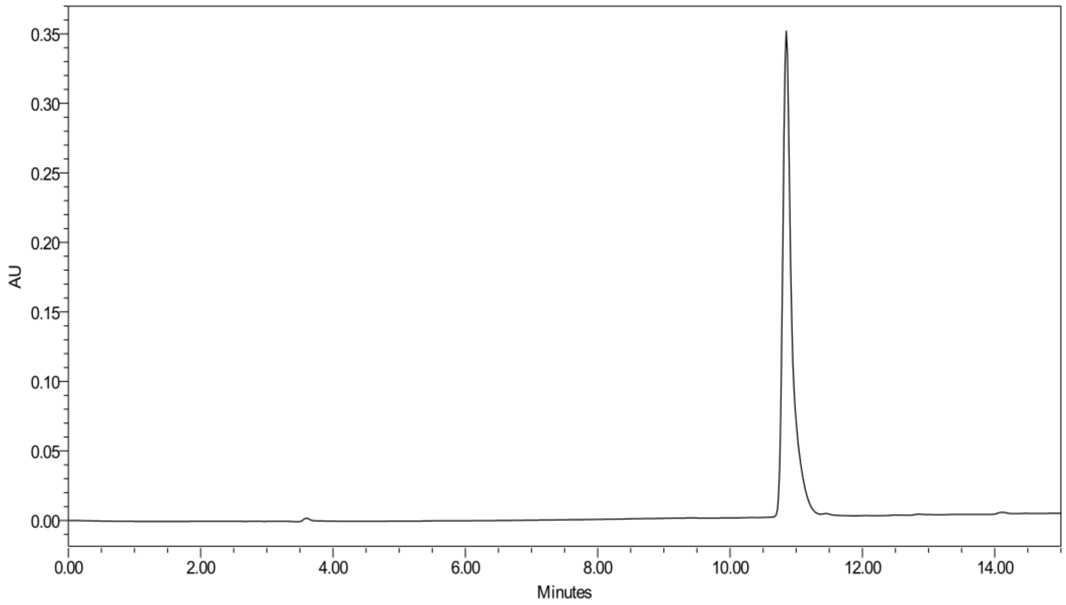

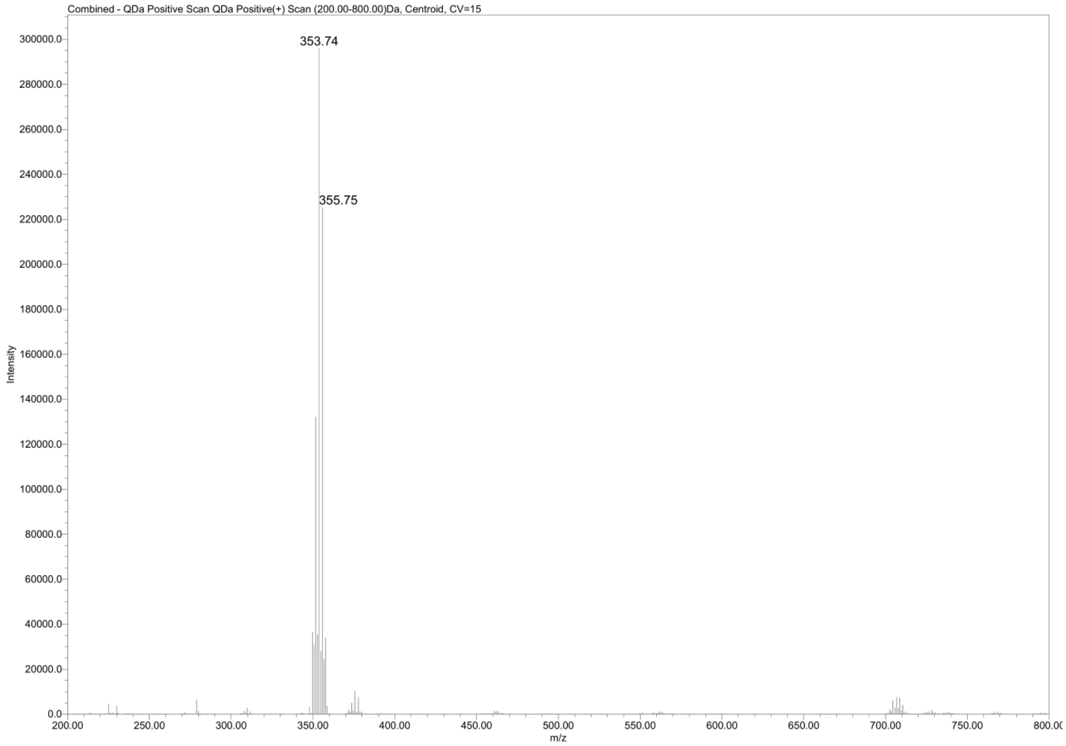

Compound 4

2-(2-methylphenyl)-benzisoselenazol-3(2*H*)-one

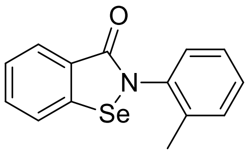

(MS: for $C_{14}H_{11}NOSe + H^+$ m/z $_{calcd}$ = 290.0079; m/z $_{found}$ = 289.85)

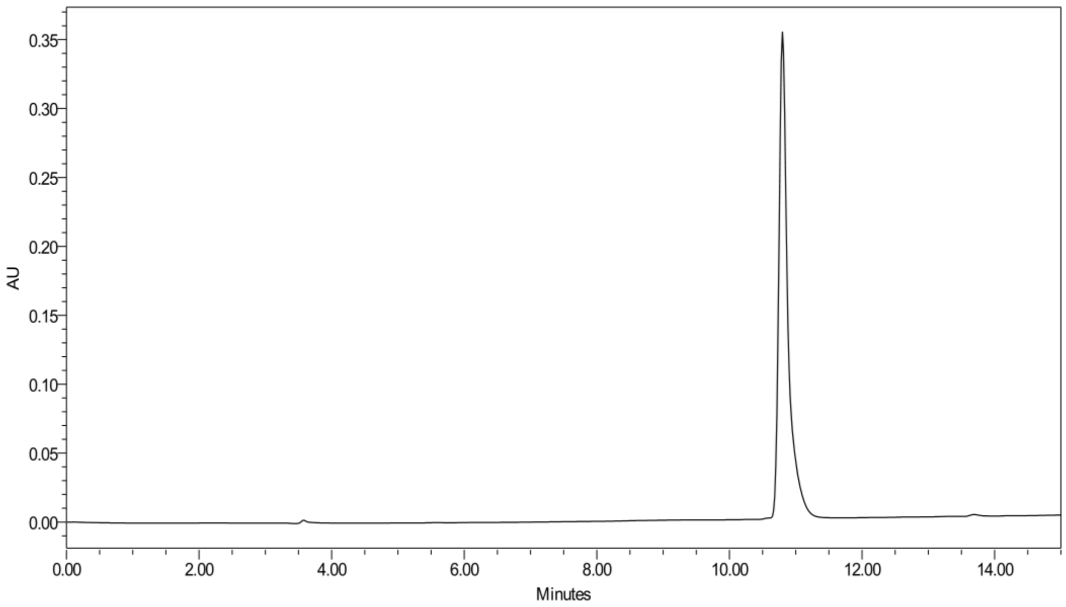

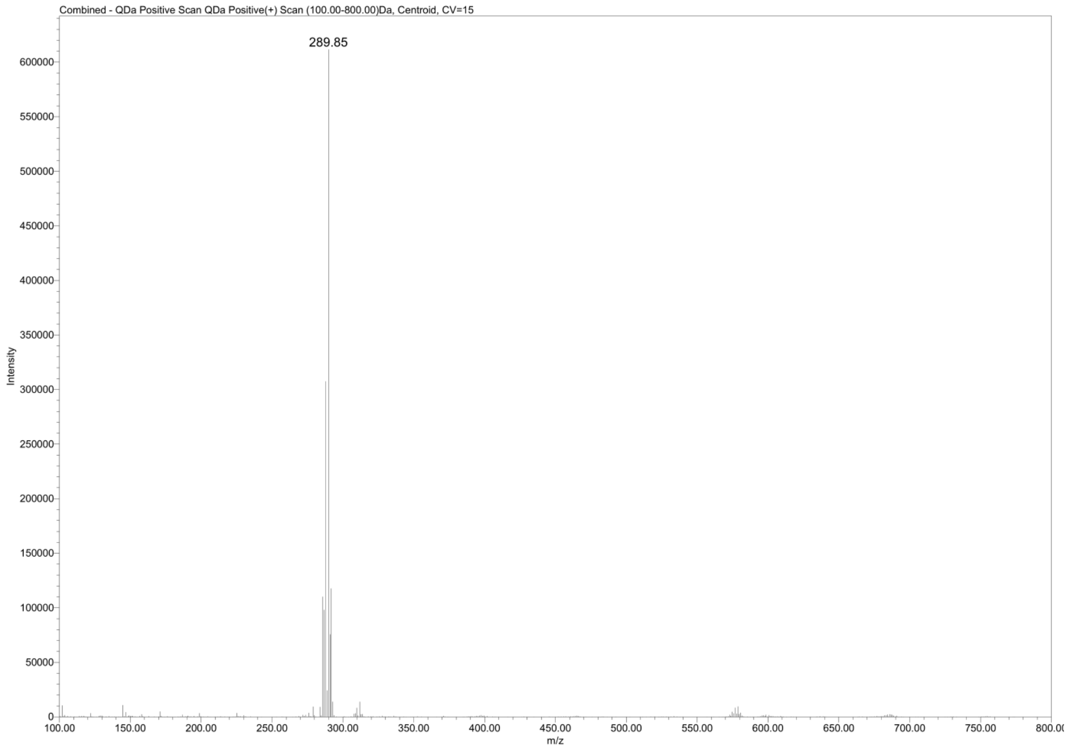

Compound 5

2-(2-(trifluoromethyl)phenyl)-benzisoselenazol-3(2*H*)-one

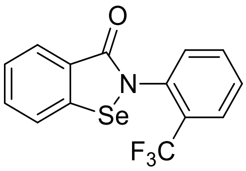

(MS: for $C_{14}H_8F_3NOSe + H^+$ m/z $_{calcd}$ = 343.9796; m/z $_{found}$ = 343.84)

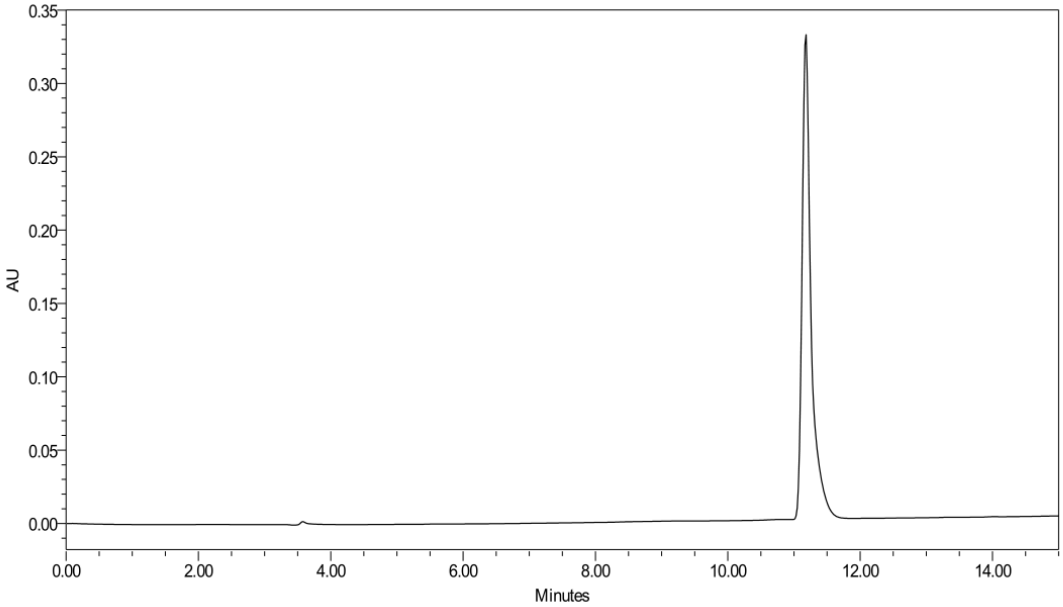

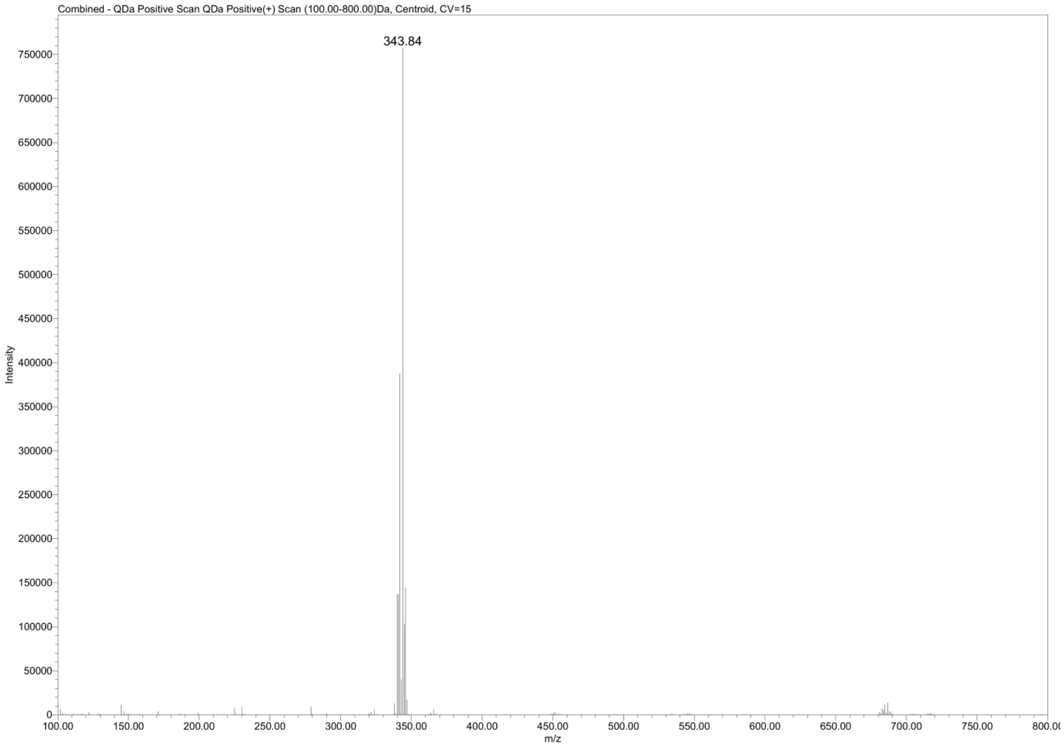

Compound 6

2-(2-nitrophenyl)-benzisoselenazol-3(2*H*)-one

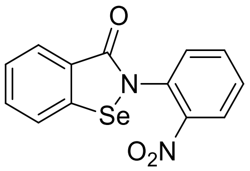

(MS: for $C_{13}H_8N_2O_3Se + H^+$ m/z $_{calcd}$ = 320.9773; m/z $_{found}$ = 320.83)

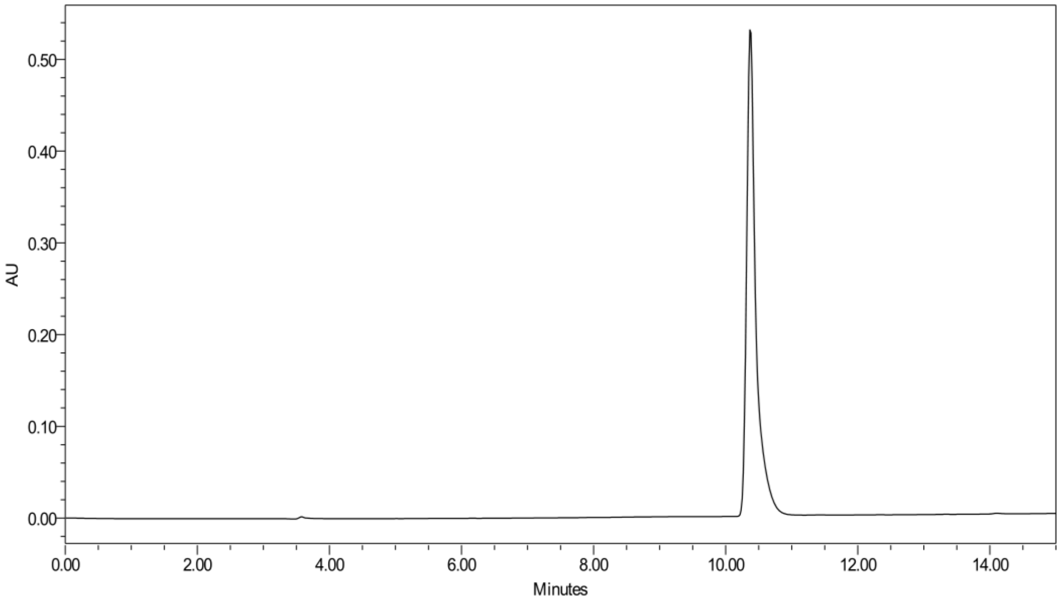

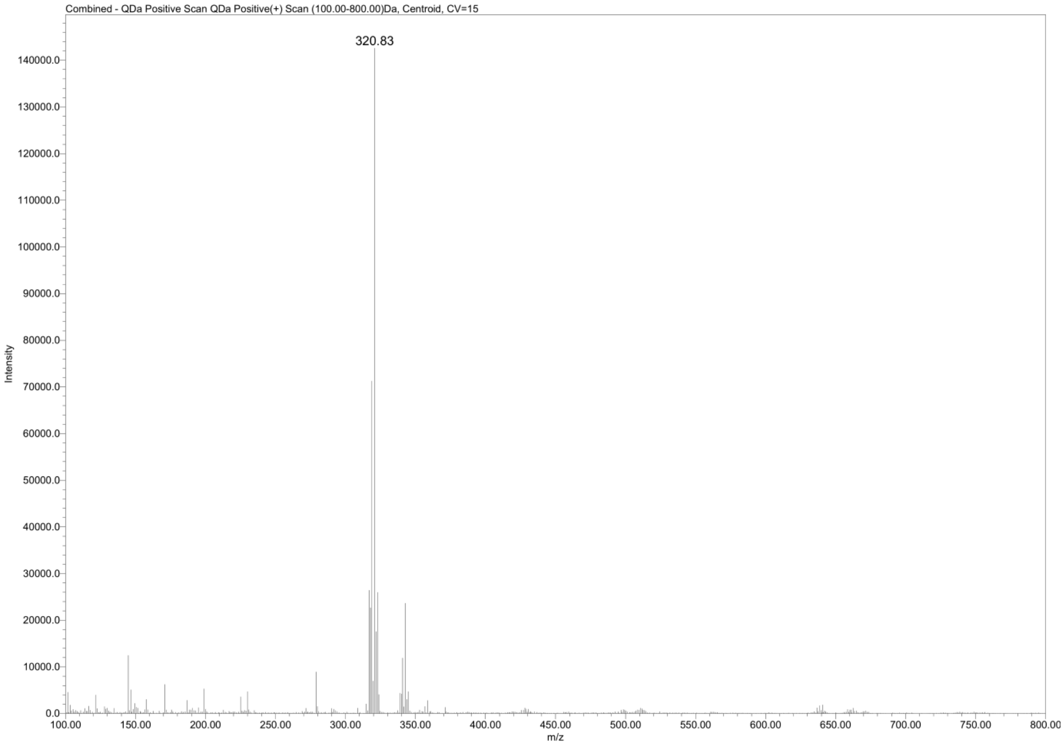

Compound 7

2-(3-hydroxypyridin-2-yl)-1,2-benzisoselenazol-3(2*H*)-one

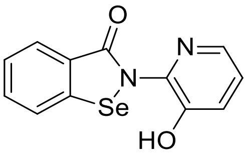

(MS: for $C_{12}H_8N_2O_2Se + H^+$ m/z $_{calcd}$ = 292.9824; m/z $_{found}$ = 292.80)

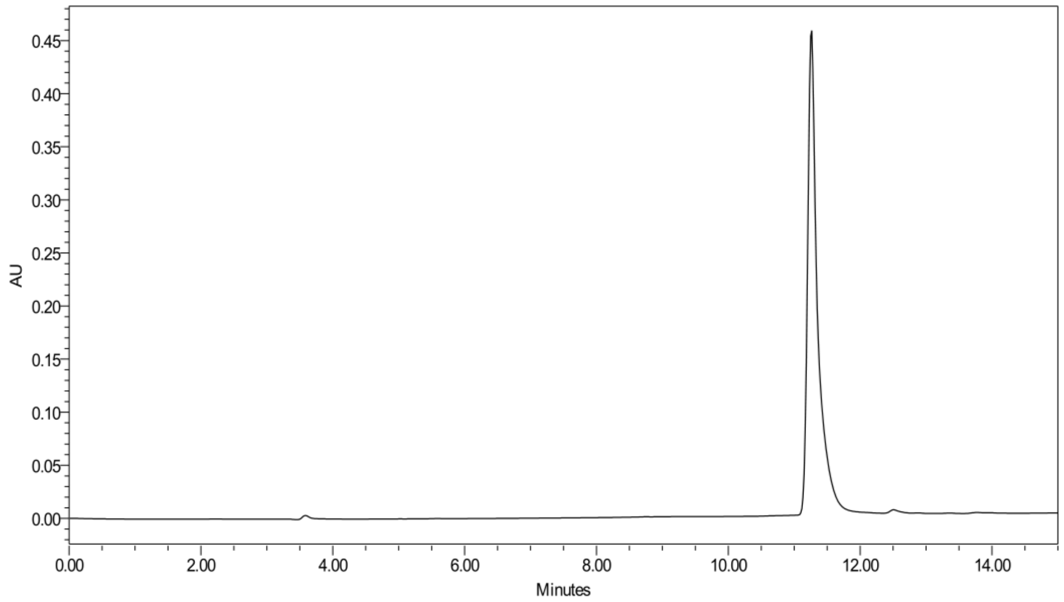

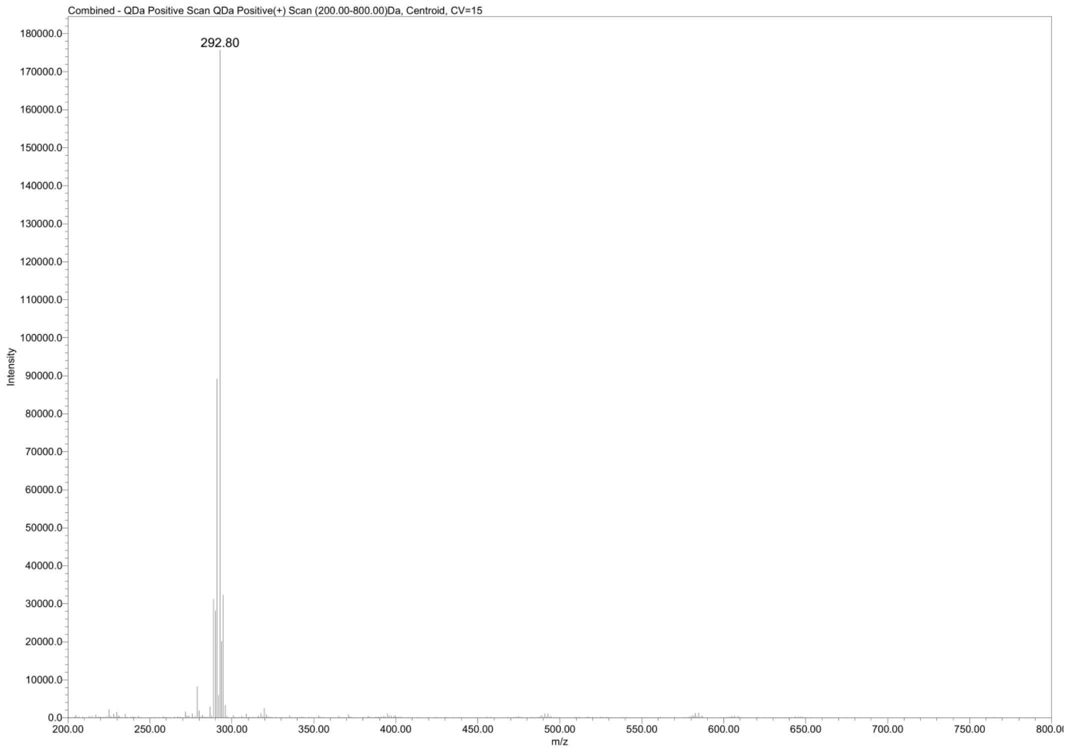

Compound 8

2-(3-methoxyphenyl)-benzisoselenazol-3(2*H*)-one

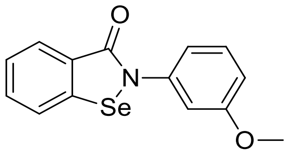

(MS: for $C_{14}H_{11}NO_2Se + H^+$ m/z $_{calcd}$ = 306.0028; m/z $_{found}$ = 305.85)

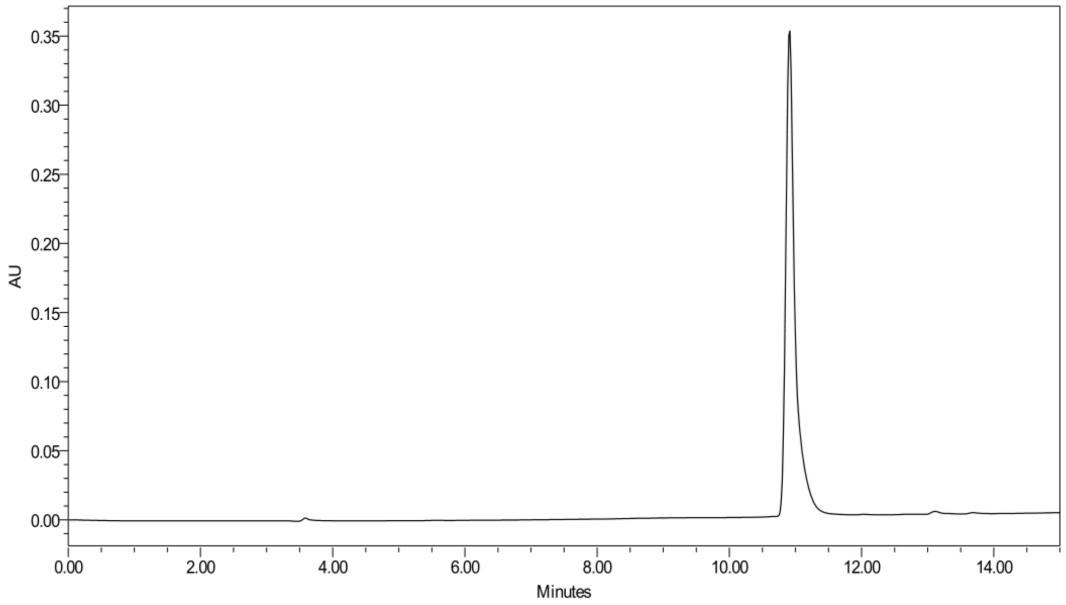

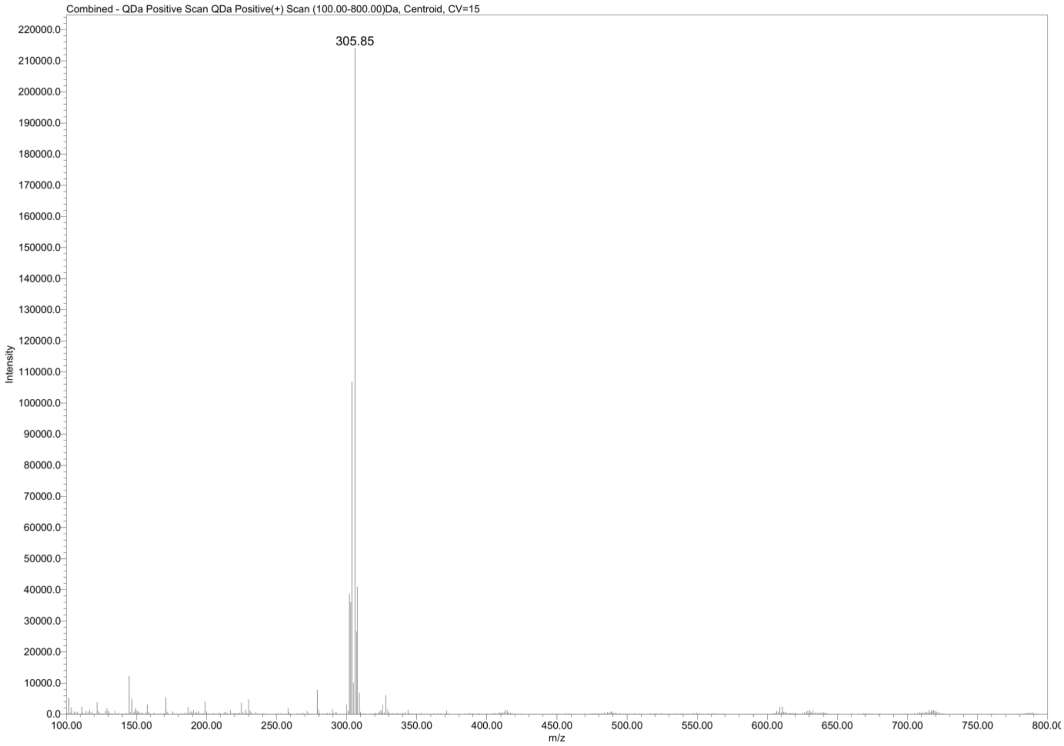

Compound 9

2-(4-(trifluoromethyl)phenyl)-benzisoselenazol-3(2*H*)-one

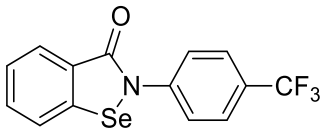

(MS: for $C_{14}H_8F_3NOSe + H^+$ m/z $_{calcd}$ = 343.9796; m/z $_{found}$ = 343.88)

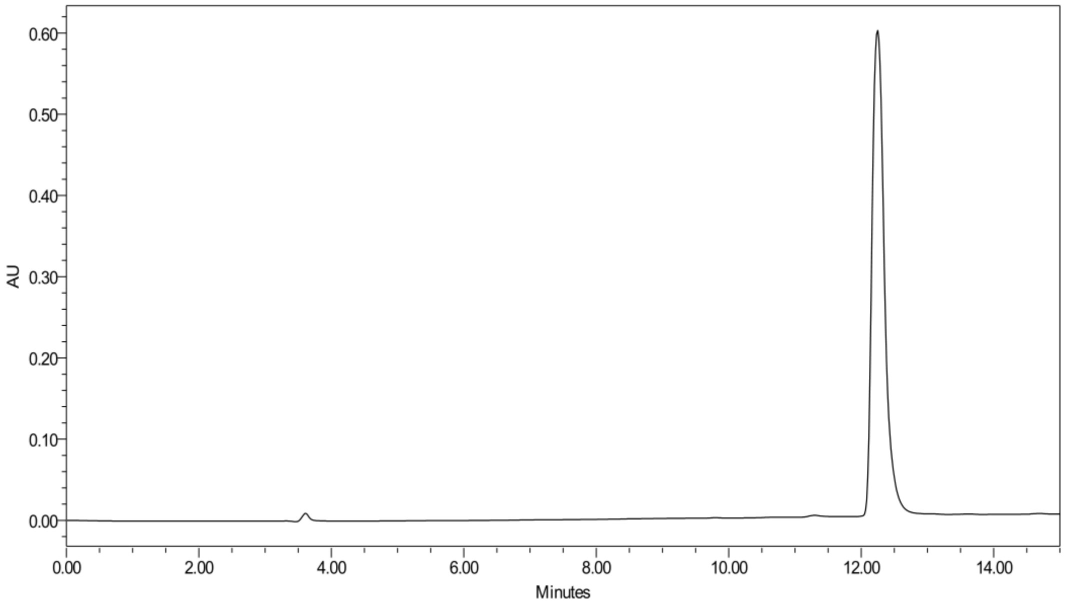

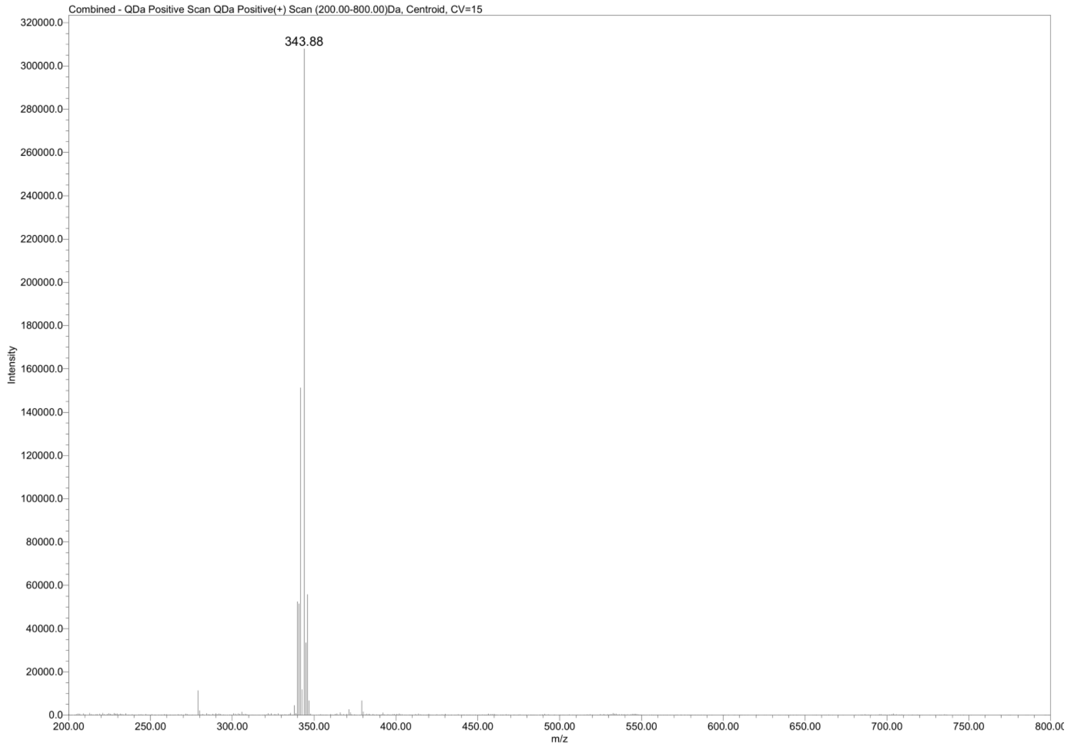

Compound 10

2-(4-nitrophenyl)-benzisoselenazol-3(2*H*)-one

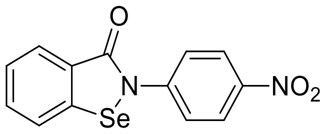

(MS: for $C_{13}H_8N_2O_3Se + H^+$ m/z $_{calcd}$ = 320.9773; m/z $_{found}$ = 320.82)

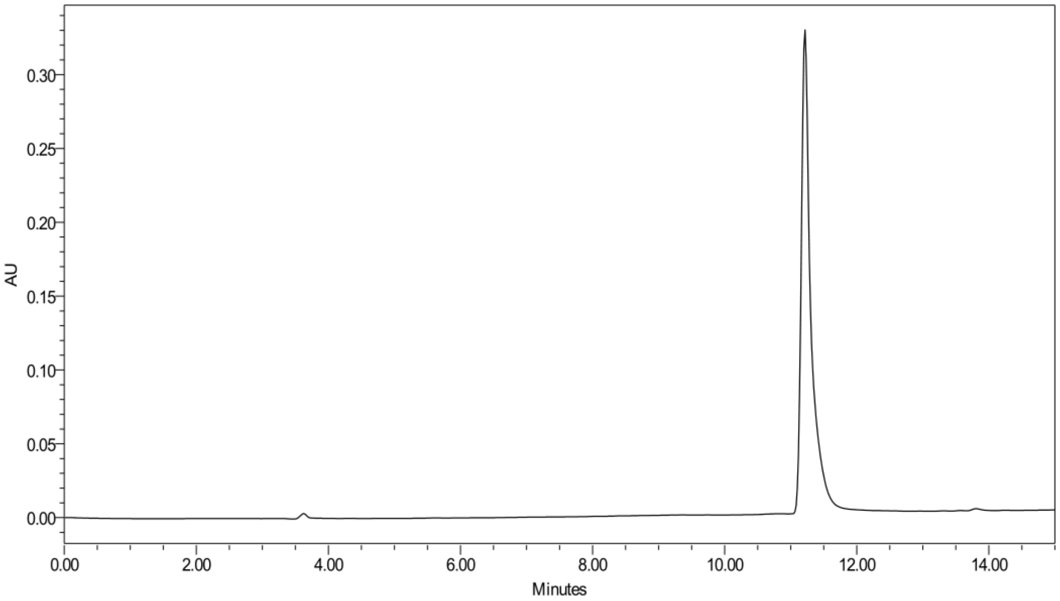

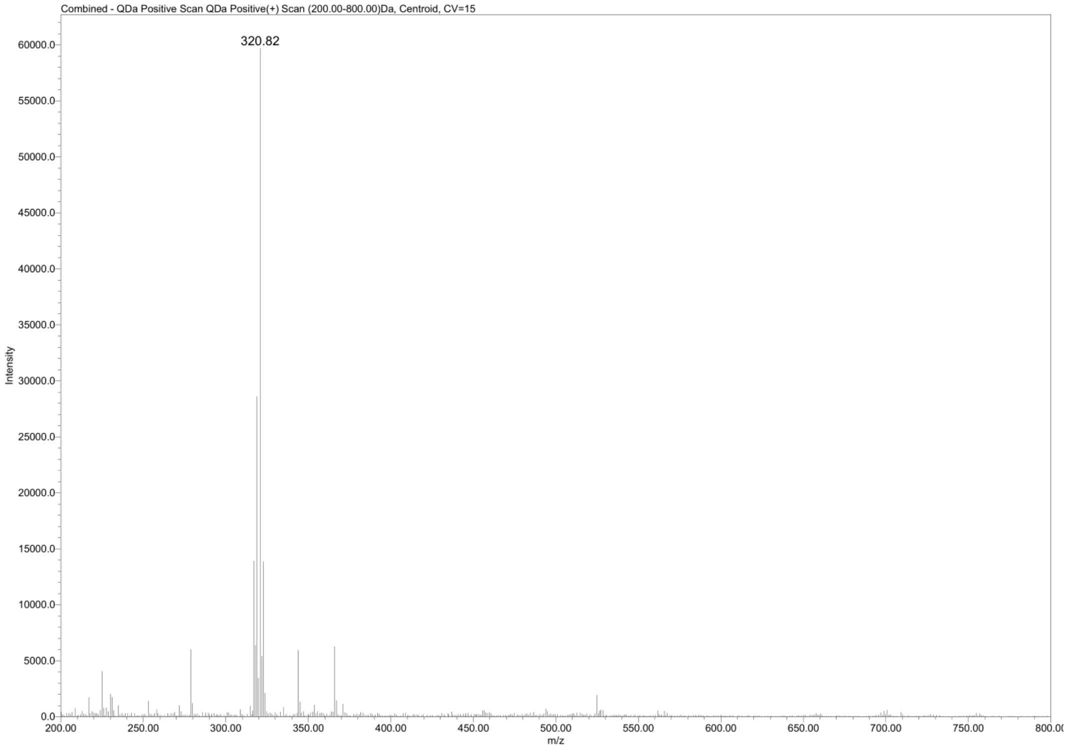

Compound 11

2-(4-iodophenyl)-benzisoselenazol-3(2*H*)-one

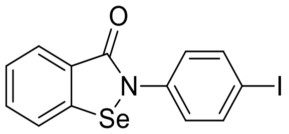

(MS: for $C_{13}H_8INOSe + H^+$ m/z $_{calcd}$ = 401.8889; m/z $_{found}$ = 401.73)

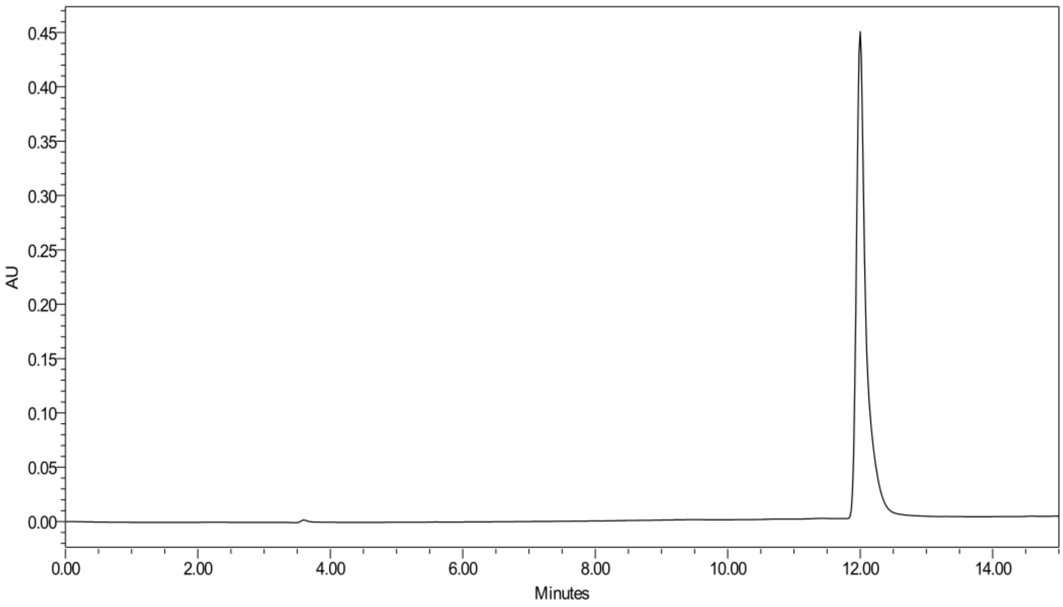

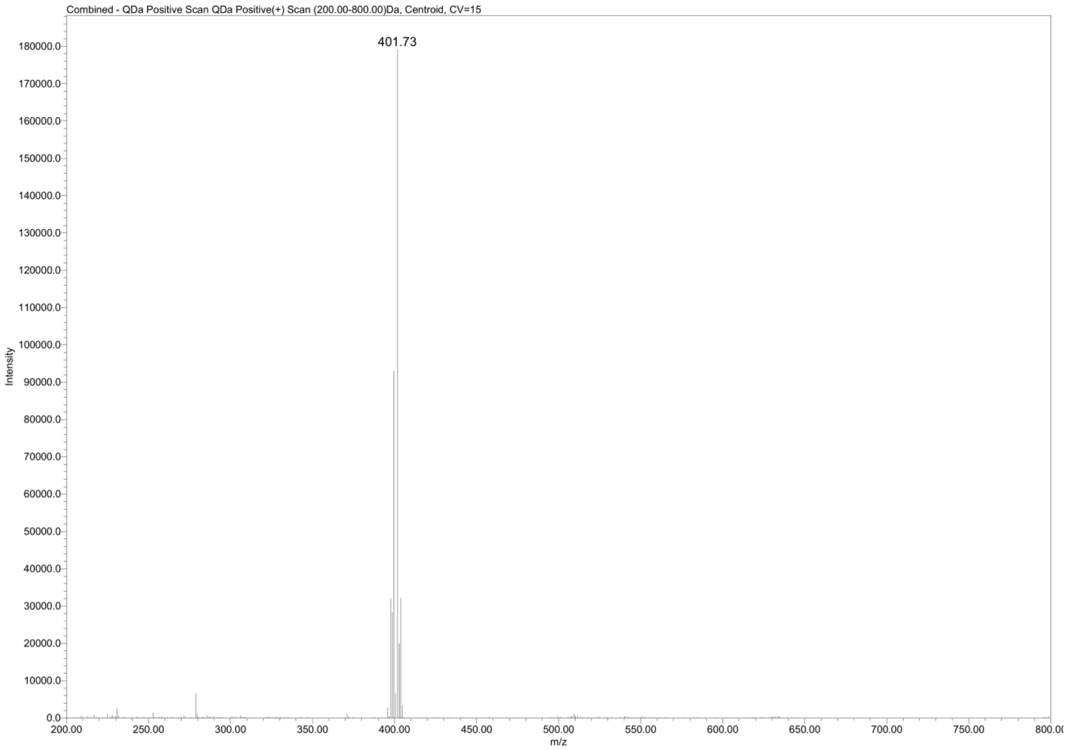

Compound 12

2-(4-acetylphenyl)-benzisoselenazol-3(2*H*)-one

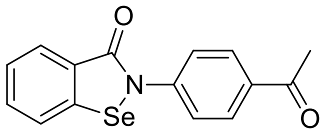

(MS: for $C_{15}H_{11}NO_2Se + H^+$ m/z $_{calcd}$ = 318.0028; m/z $_{found}$ = 317.85)

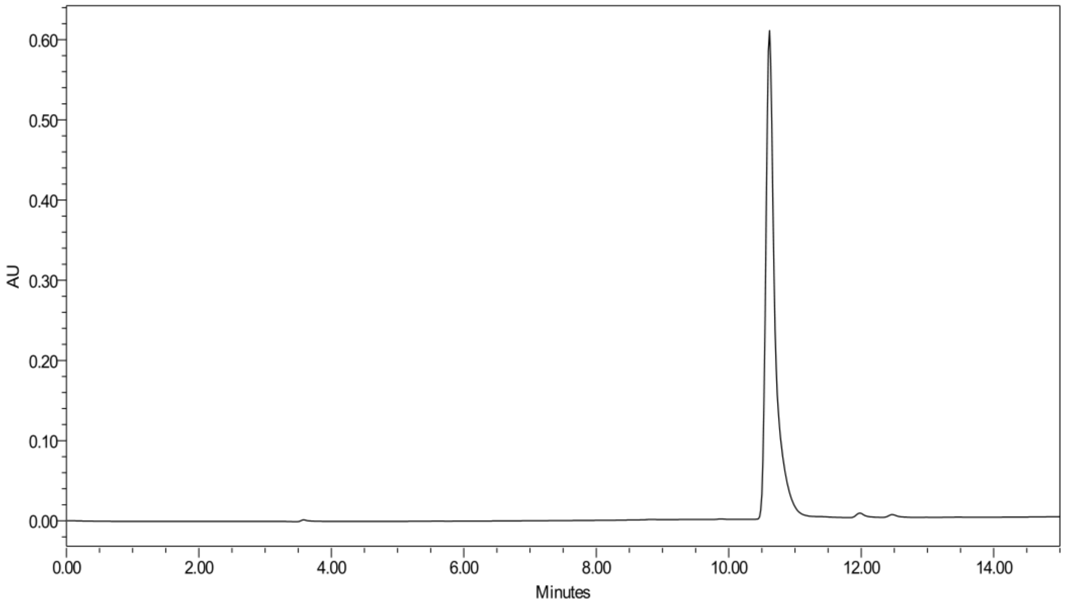

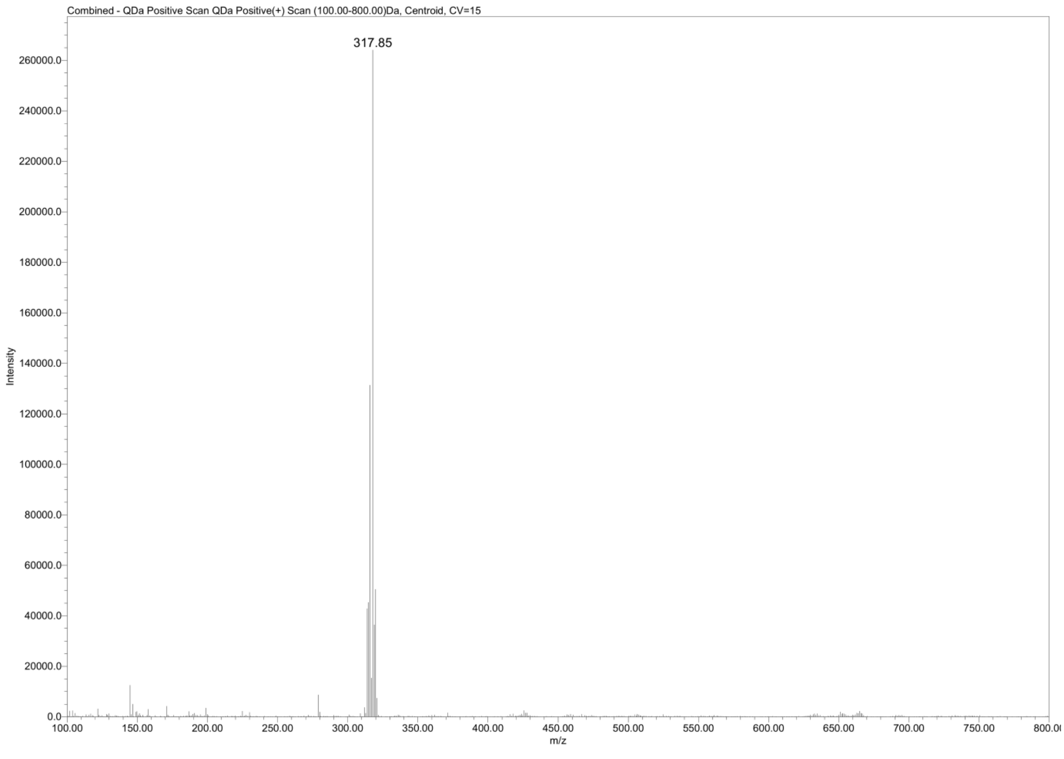

Compound 13

2-(4-acetamidophenyl)-benzisoselenazol-3(2*H*)-one

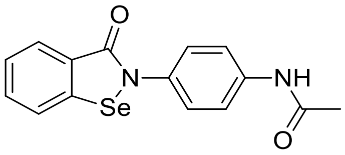

(MS: for $C_{15}H_{12}N_2O_2Se + H^+$ m/z $_{calcd}$ = 333.0137; m/z $_{found}$ = 332.86)

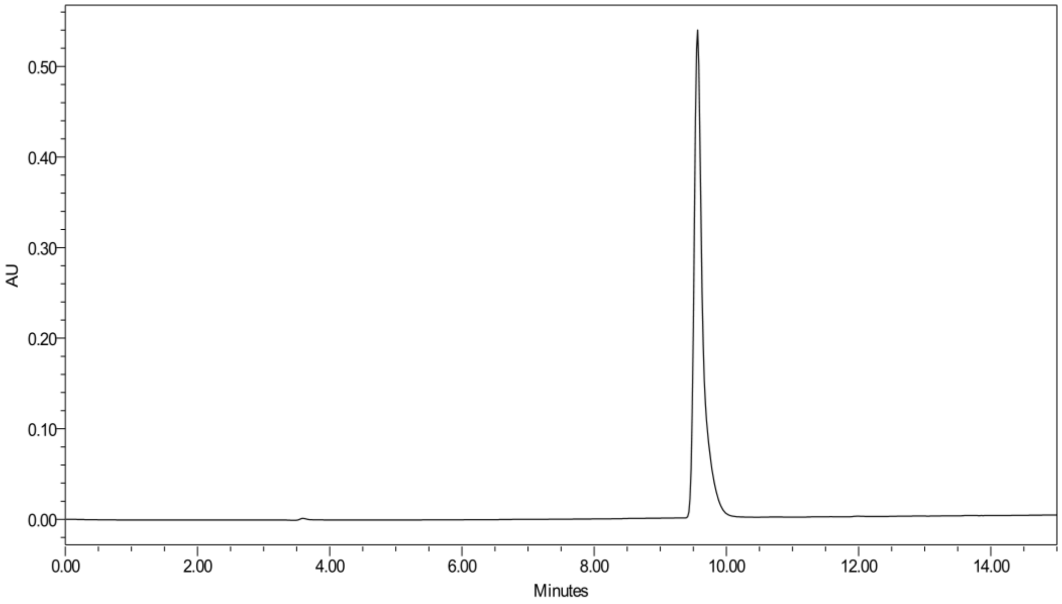

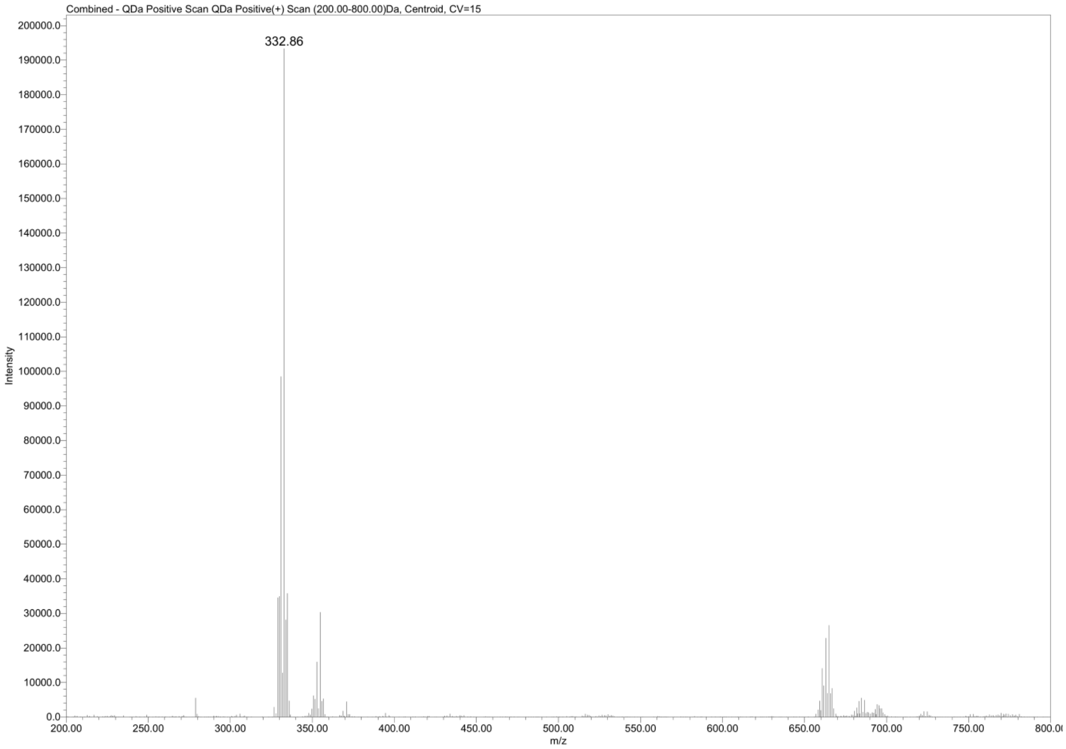

Compound 14

2-(2,4-difluorophenyl)-benzisoselenazol-3(2*H*)-one

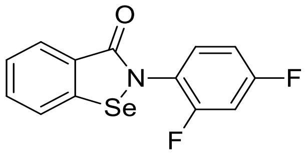

(MS: for $C_{13}H_7F_2NOSe + H^+$ m/z $_{calcd}$ = 311.9734; m/z $_{found}$ = 311.82)

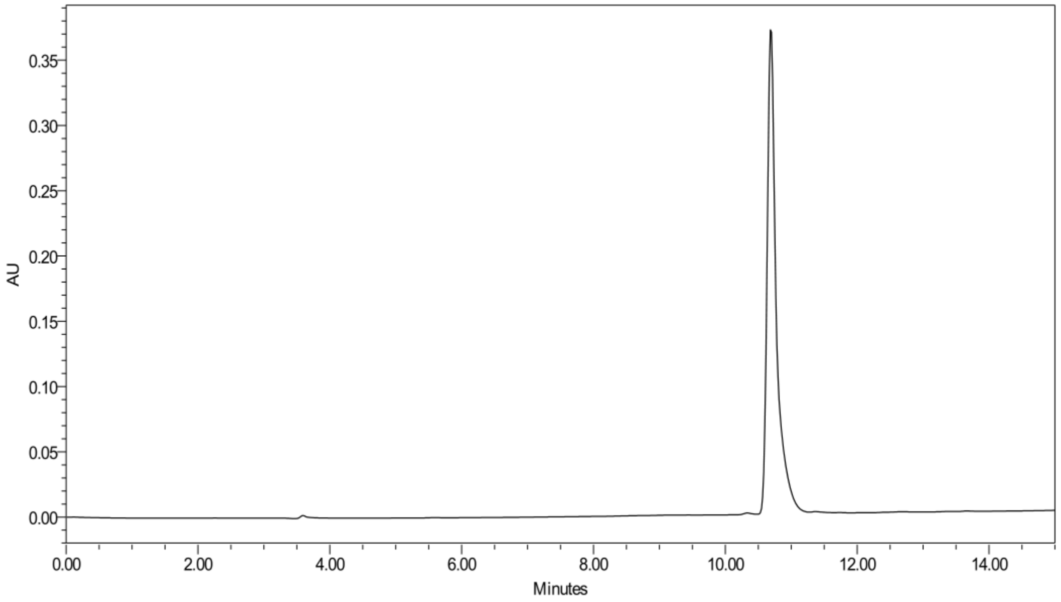

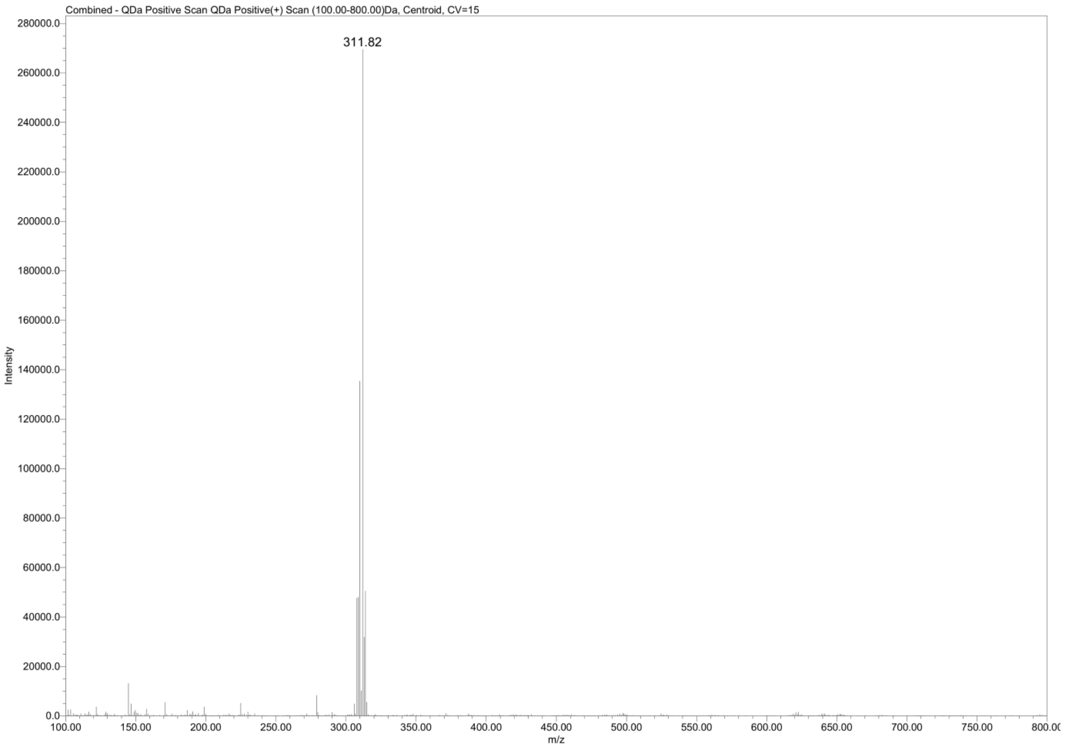

Compound 15

2-(4-chloro-2-fluorophenyl)-benzisoselenazol-3(2*H*)-one

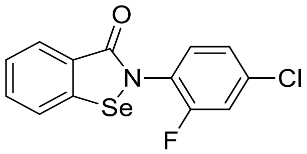

(MS: for $C_{13}H_7ClFNOSe + H^+$ m/z $_{calcd}$ = 327.9439; m/z $_{found}$ = 327.78)

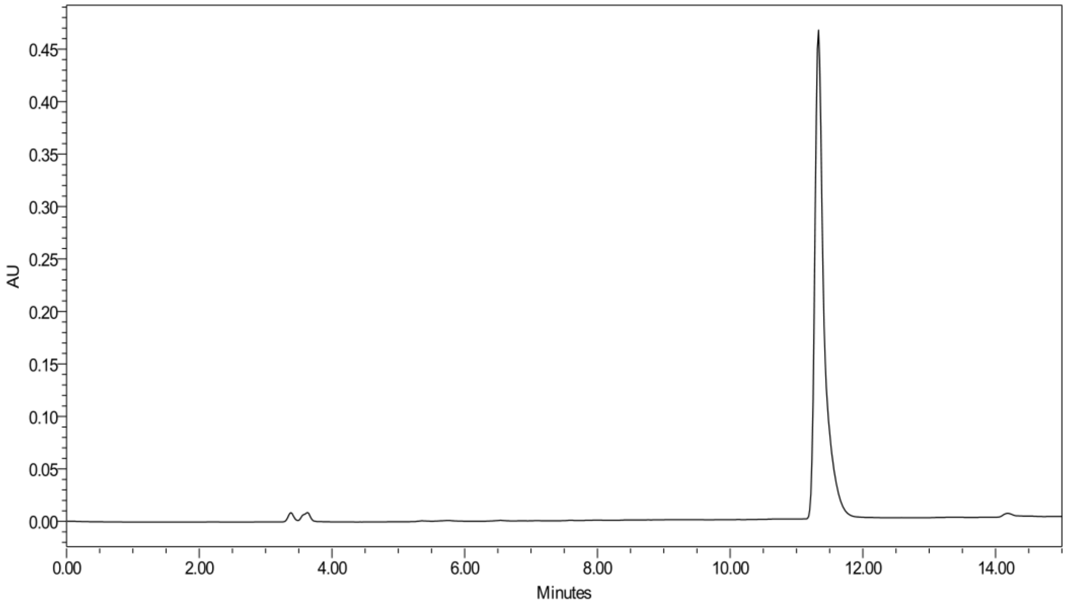

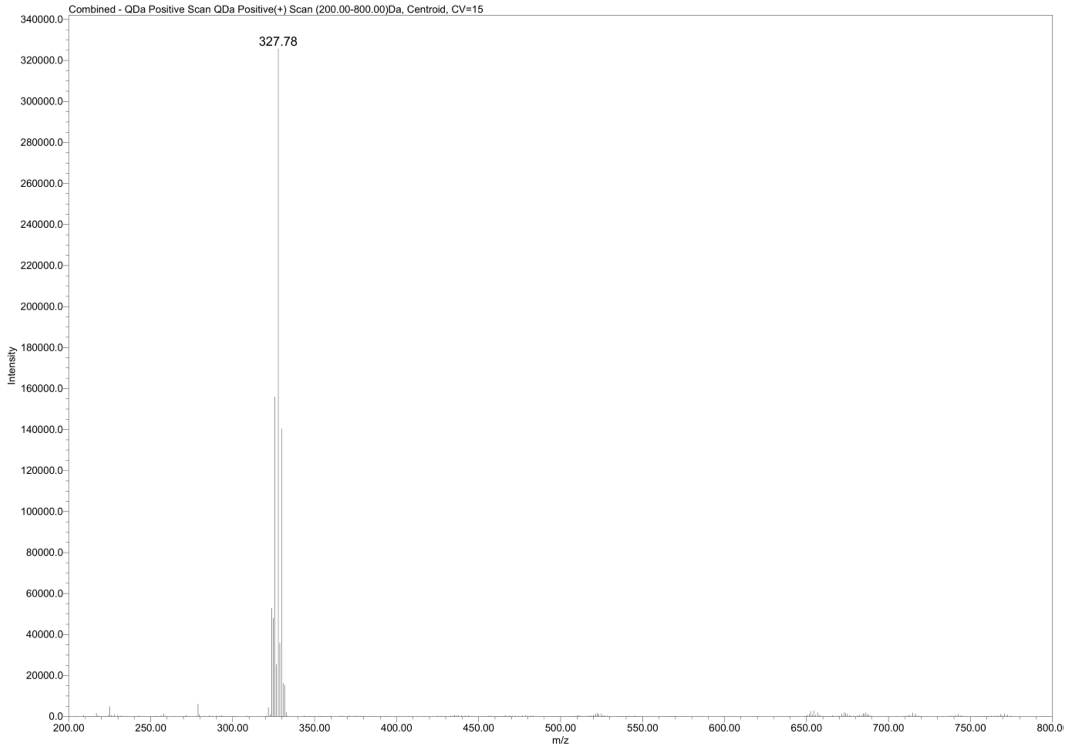

Compound 16

2-(2,4-dimethoxyphenyl)-benzisoselenazol-3(2*H*)-one

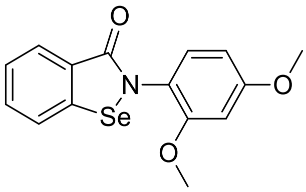

(MS: for $C_{15}H_{13}NO_3Se + H^+$ m/z $_{calcd}$ = 336.0134; m/z $_{found}$ = 335.88)

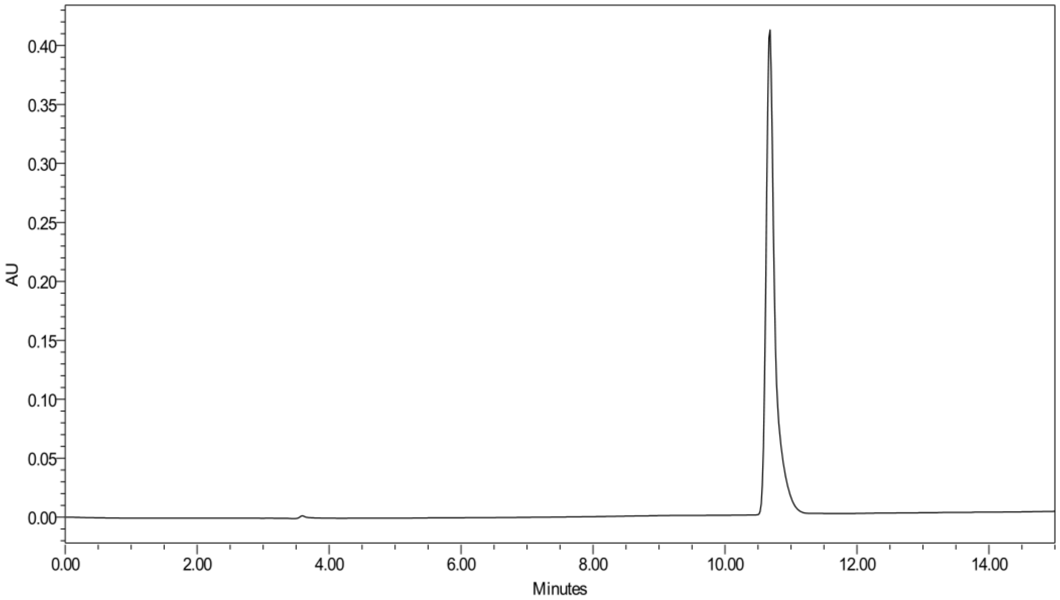

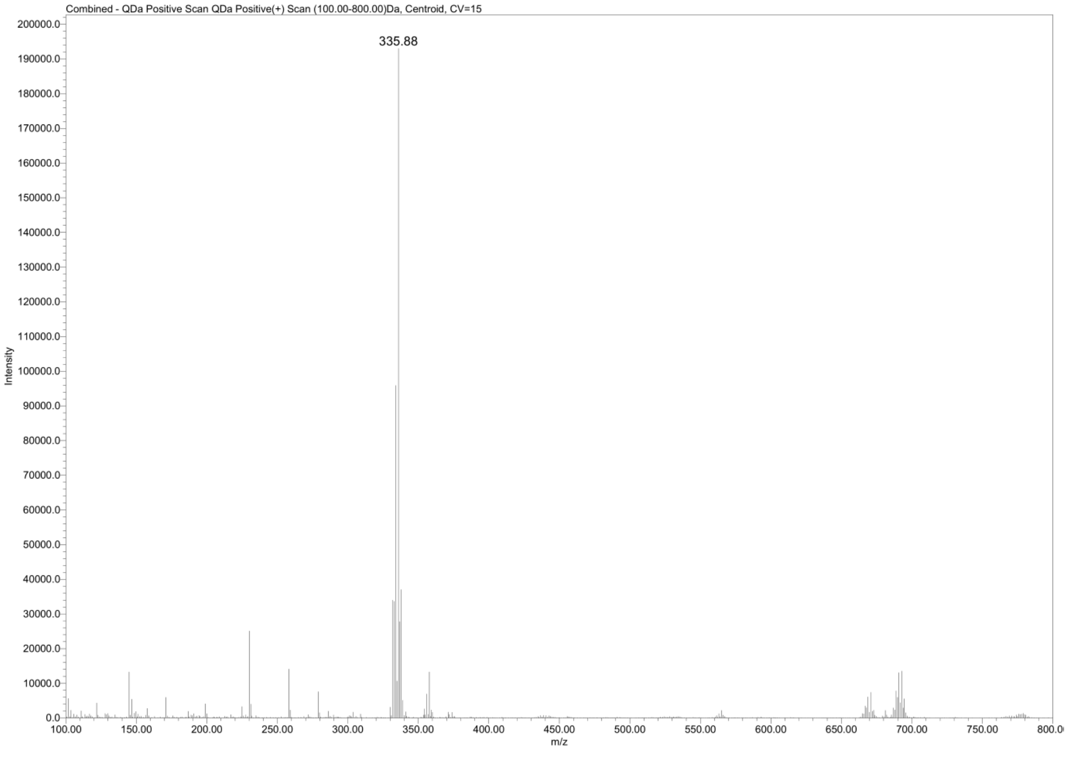

Compound 17

2-(5-chloro-2-fluorophenyl)-benzisoselenazol-3(2*H*)-one

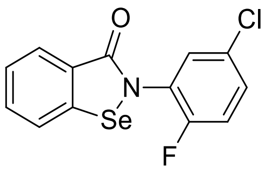

(MS: for $C_{13}H_7ClFNOSe + H^+$ m/z $_{calcd}$ = 327.9439; m/z $_{found}$ = 327.80)

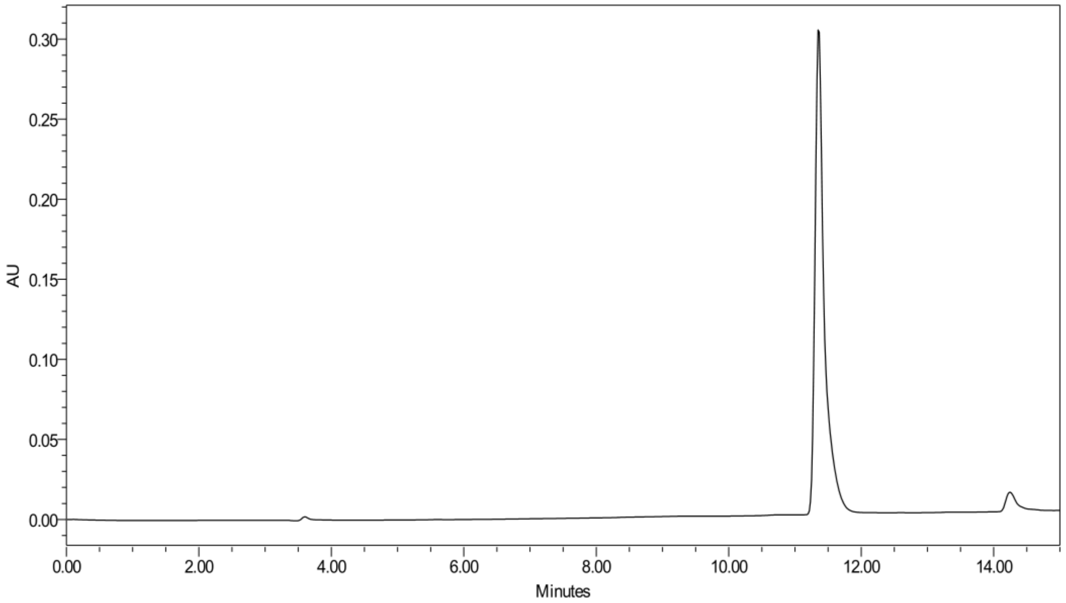

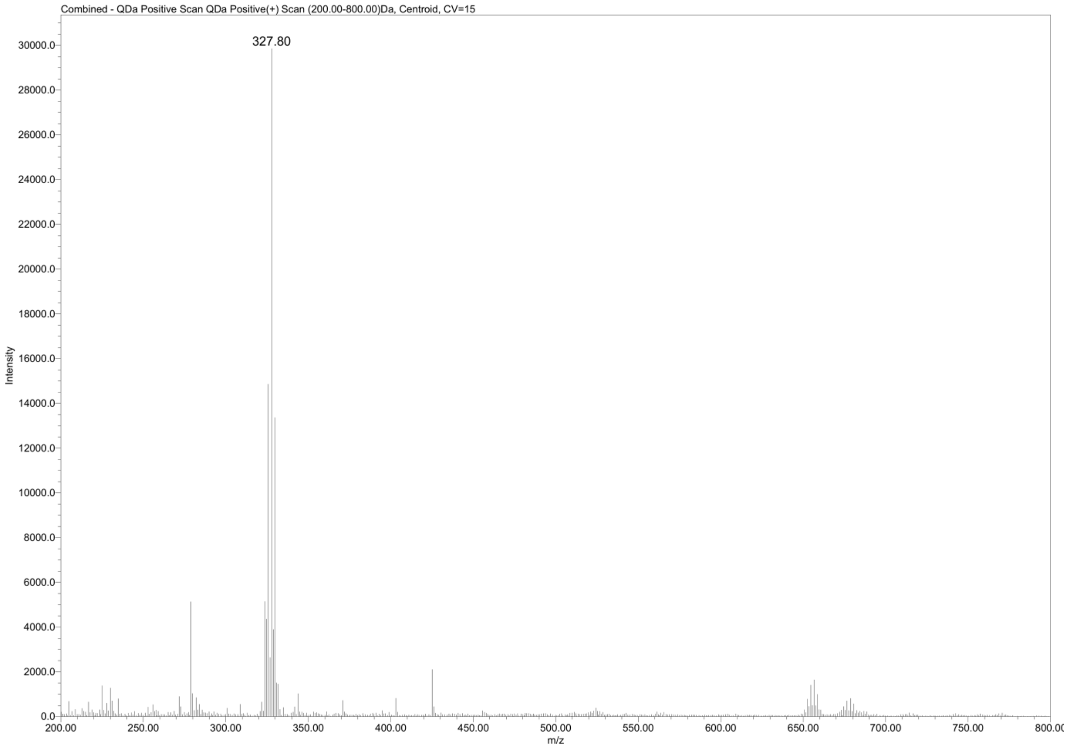

Compound 18

2-(2,5-dichlorophenyl)-benzisoselenazol-3(2*H*)-one

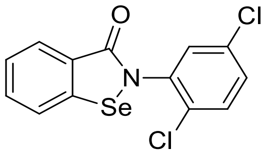

(MS: for $C_{13}H_7Cl_2NOSe + H^+$ m/z $_{calcd}$ = 343.9143; m/z $_{found}$ = 343.75)

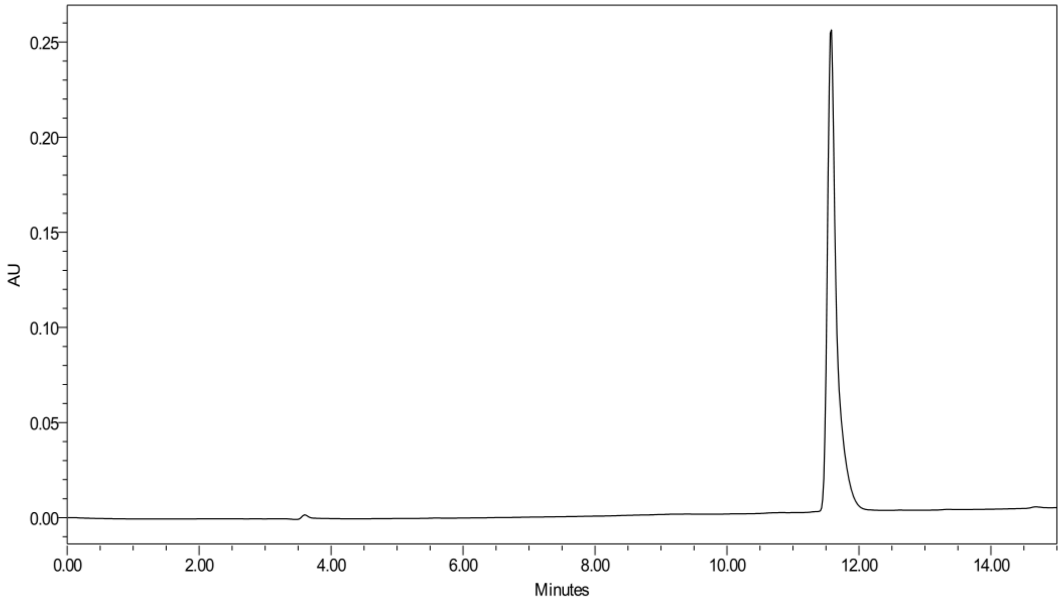

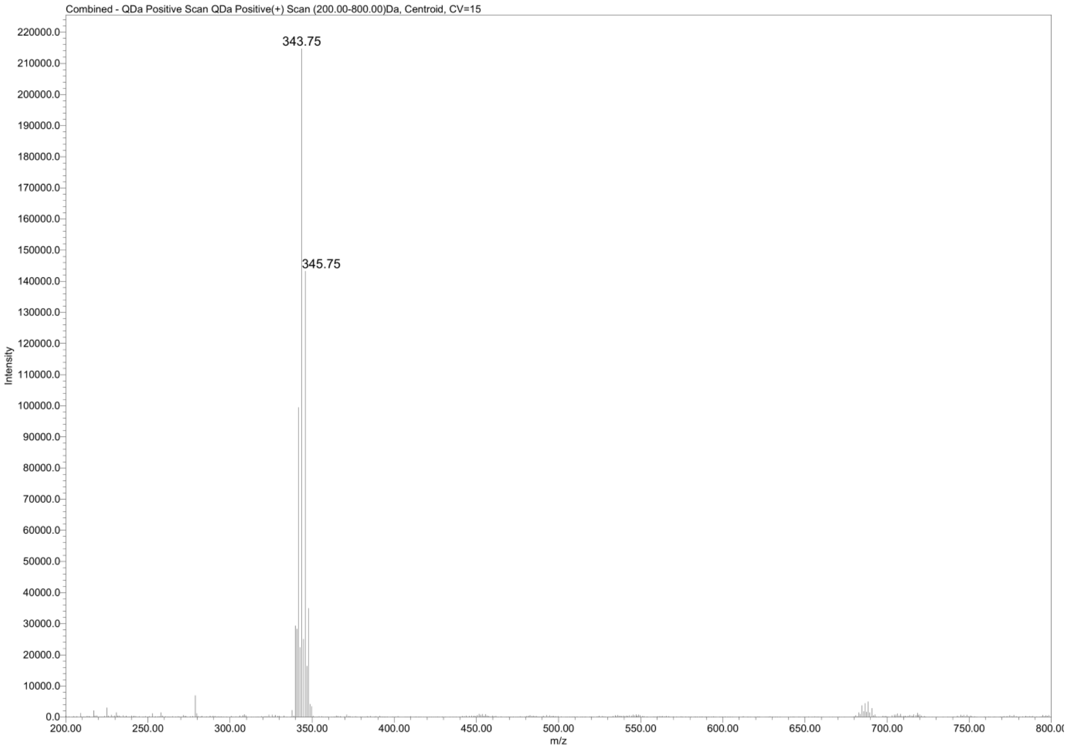

Compound 19

2-(2-chloro-5-methylphenyl)-benzisoselenazol-3(2*H*)-one

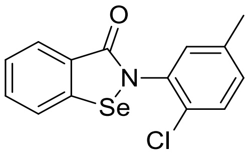

(MS: for $C_{14}H_{10}ClNOSe + H^+$ m/z $_{calcd}$ = 323.9689; m/z $_{found}$ = 323.87)

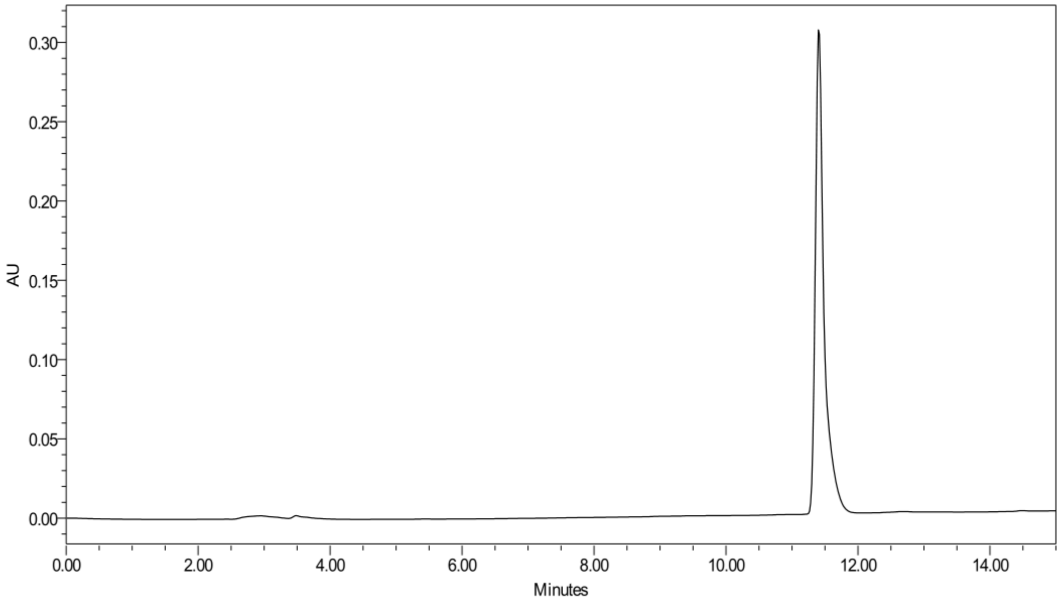

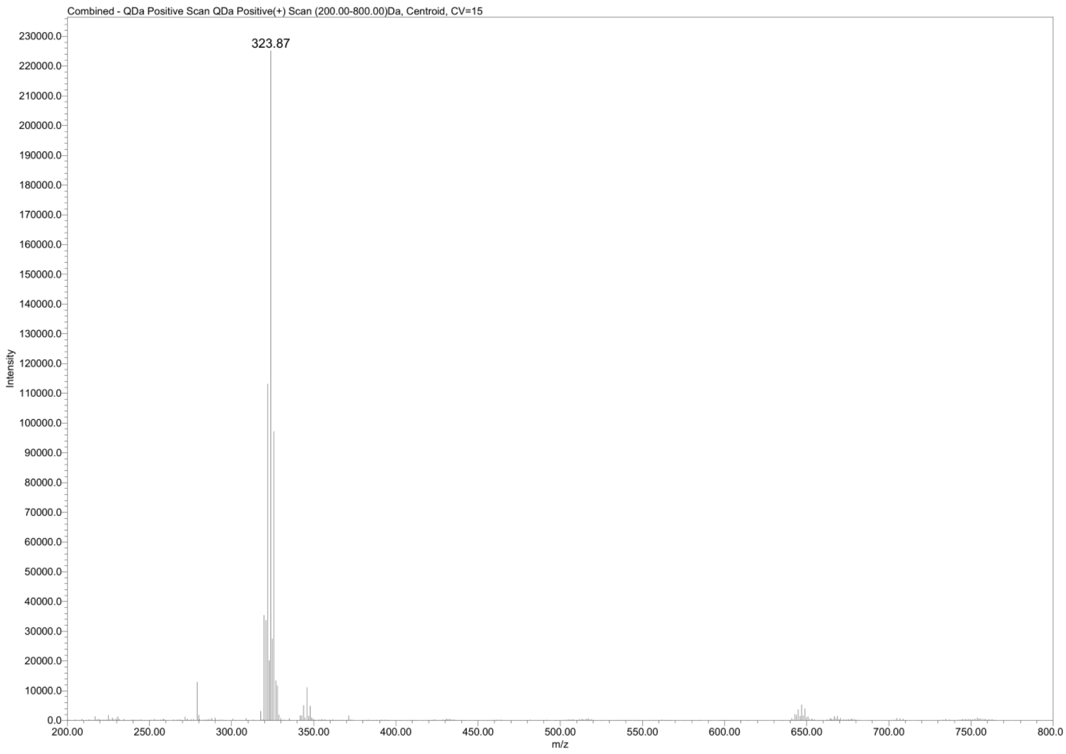

Compound 20

2-(5-chloro-2-methylphenyl)-benzisoselenazol-3(2*H*)-one

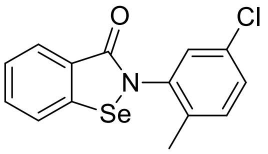

(MS: for for $C_{14}H_{10}ClNOSe + H^+$ m/z $_{calcd}$ =323.9689; m/z $_{found}$ =323.88)

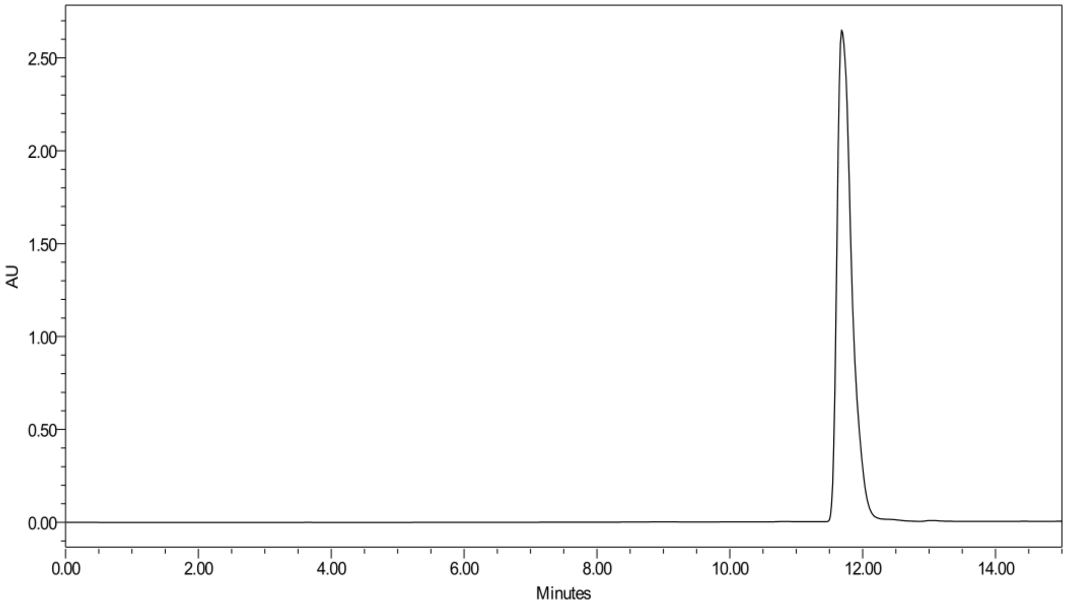

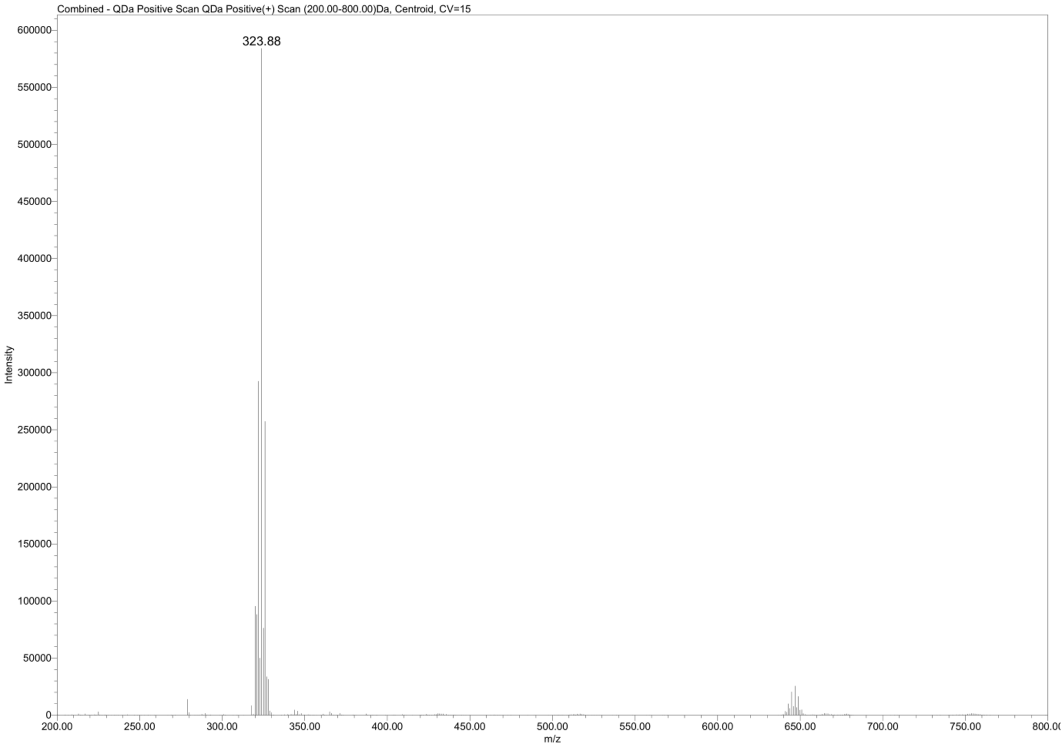

Compound 21

2-(3-chloro-2-methylphenyl)-benzisoselenazol-3(2*H*)-one

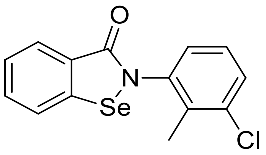

(MS: for $C_{14}H_{10}ClNOSe + H^+$ m/z $_{calcd}$ = 323.9689; m/z $_{found}$ = 323.83)

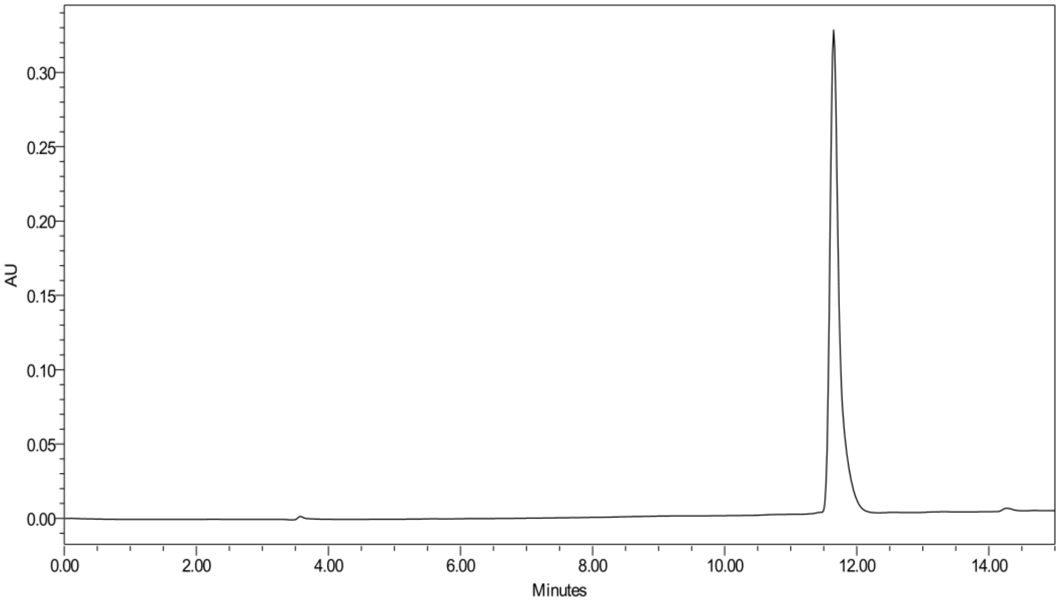

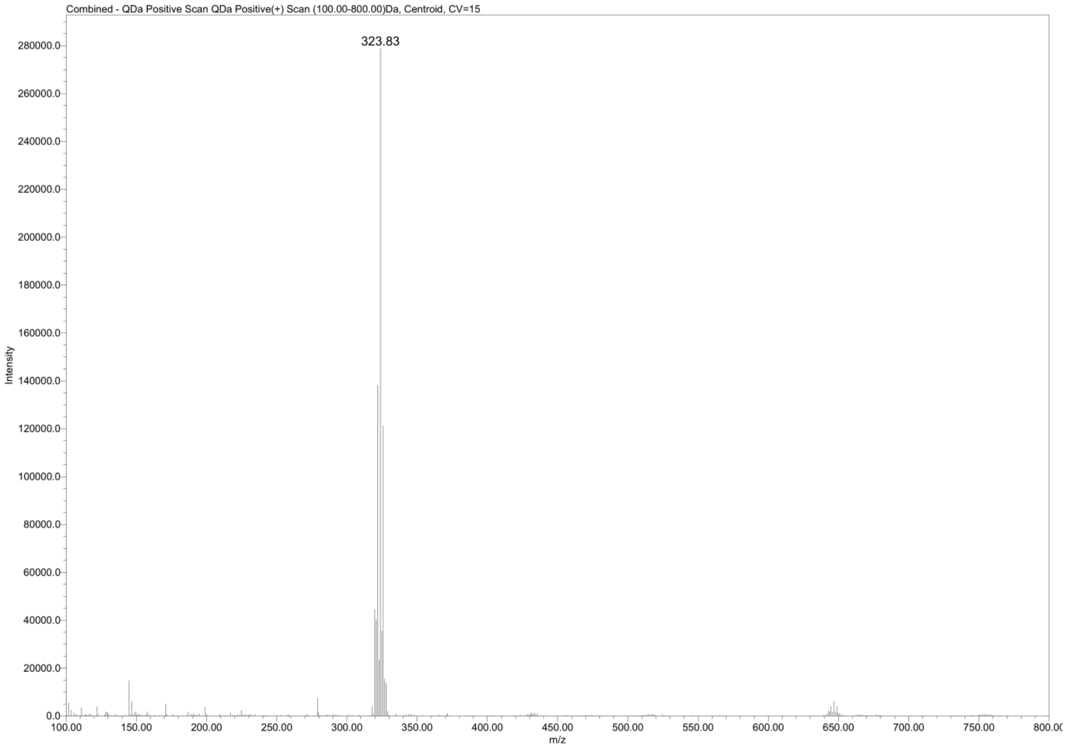

Compound 22

2-(4-chloro-3-methylphenyl)-benzisoselenazol-3(2*H*)-one

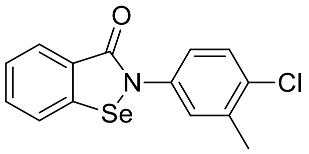

(MS: for $C_{14}H_{10}ClNOSe + H^+$ m/z $_{calcd}$ = 323.9689; m/z $_{found}$ = 323.83)

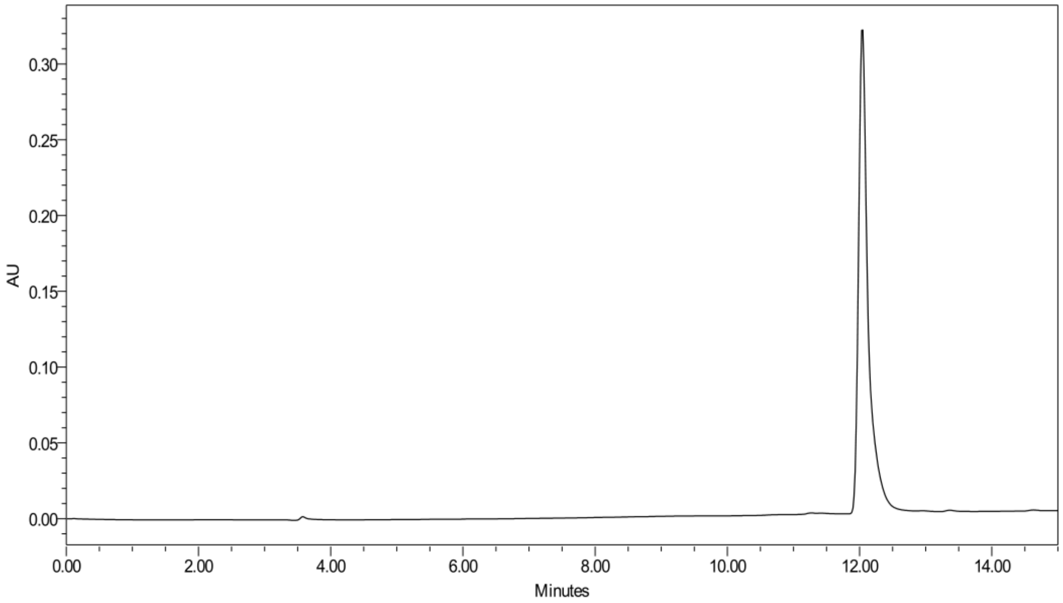

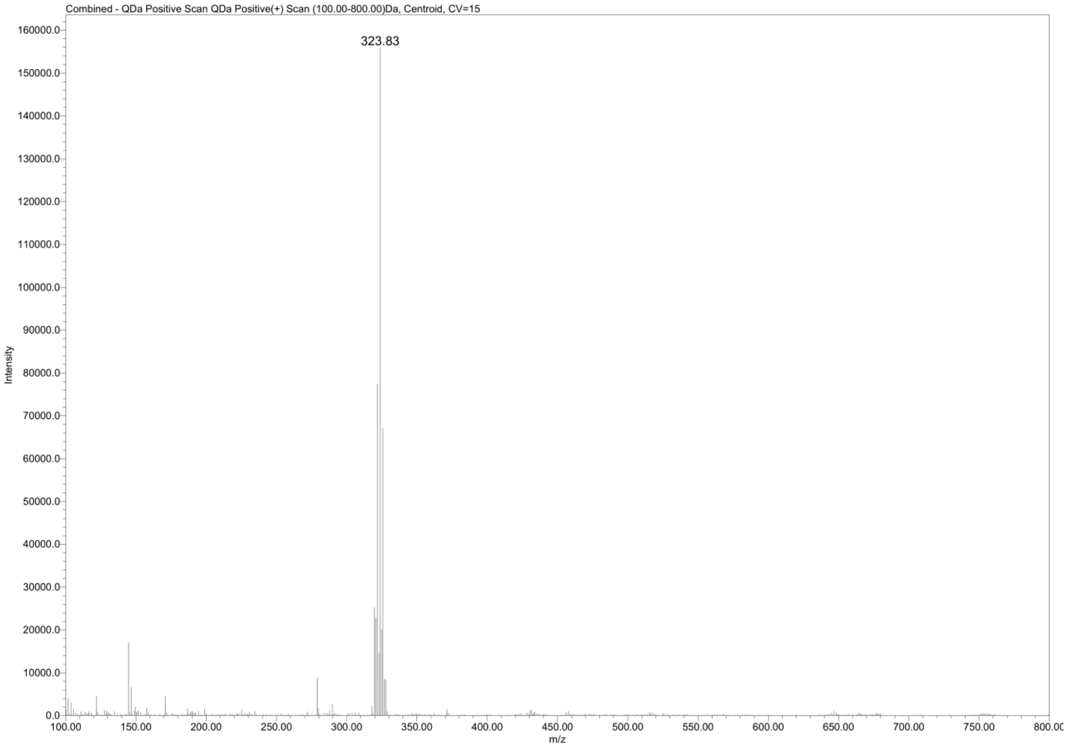

Compound 23

2-(3,4-dimethoxyphenyl)-benzisoselenazol-3(2*H*)-one

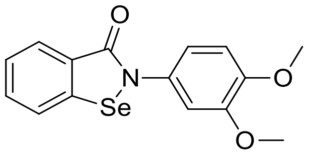

(MS: for $C_{15}H_{13}NO_3Se + H^+$ m/z $_{calcd}$ = 336.0134; m/z $_{found}$ = 335.87)

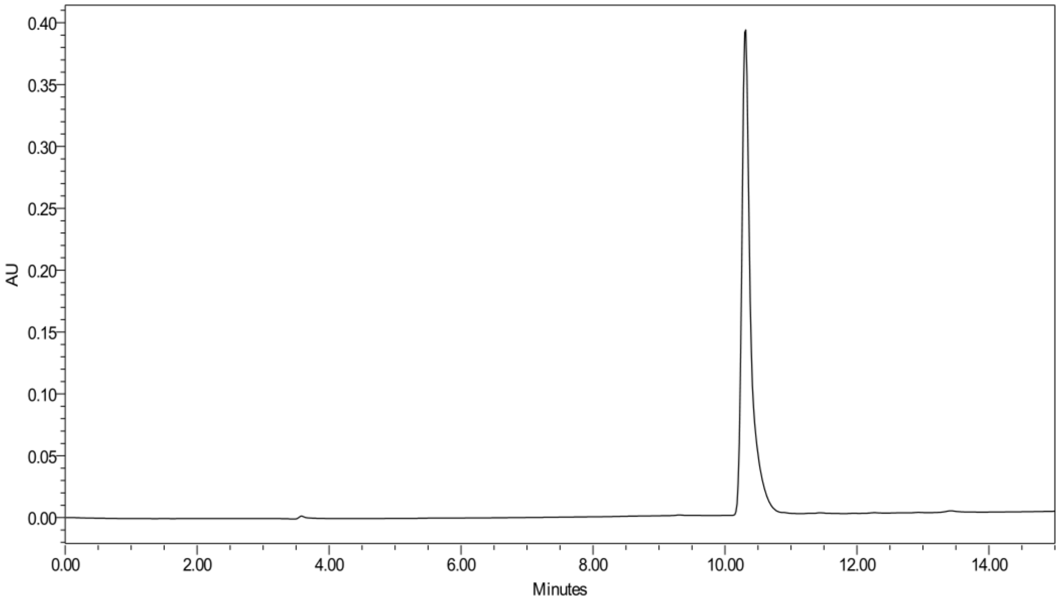

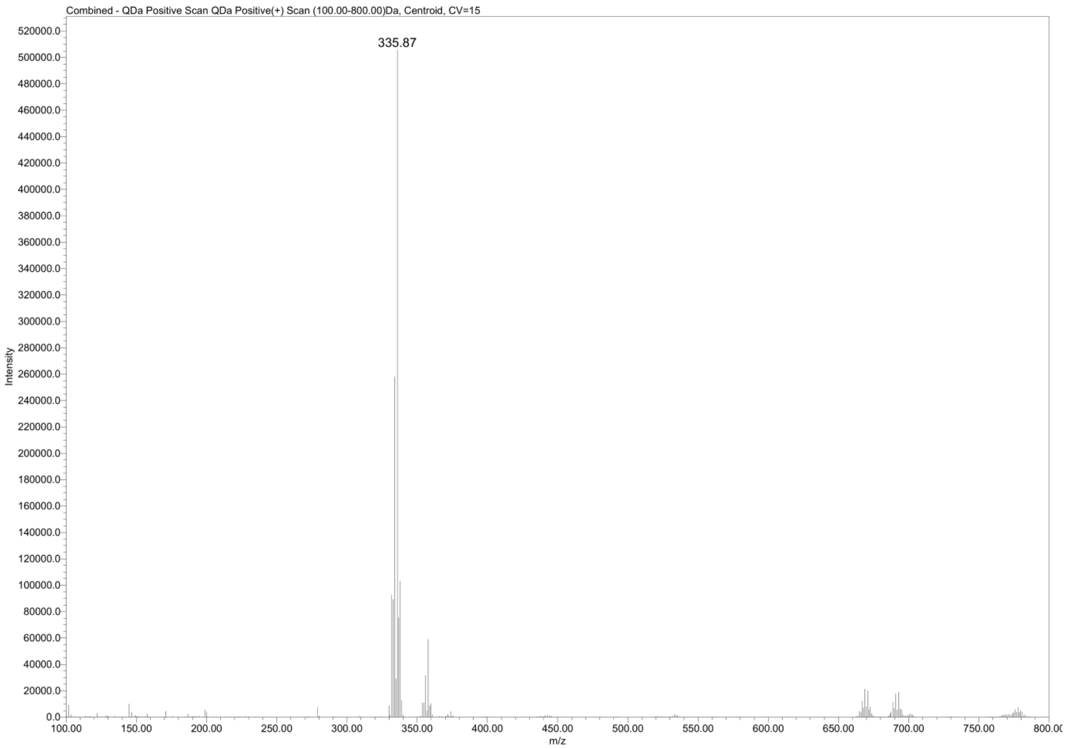

Compound 24

bis[2-(*N*-phenylcarbamoyl)phenyl] diselenide

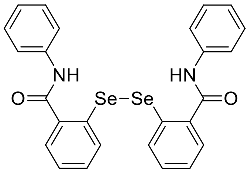

(MS: for $C_{26}H_{20}N_2O_2Se_2 + H^+$ m/z $_{calcd}$ = 552.9928; m/z $_{found}$ = 552.81)

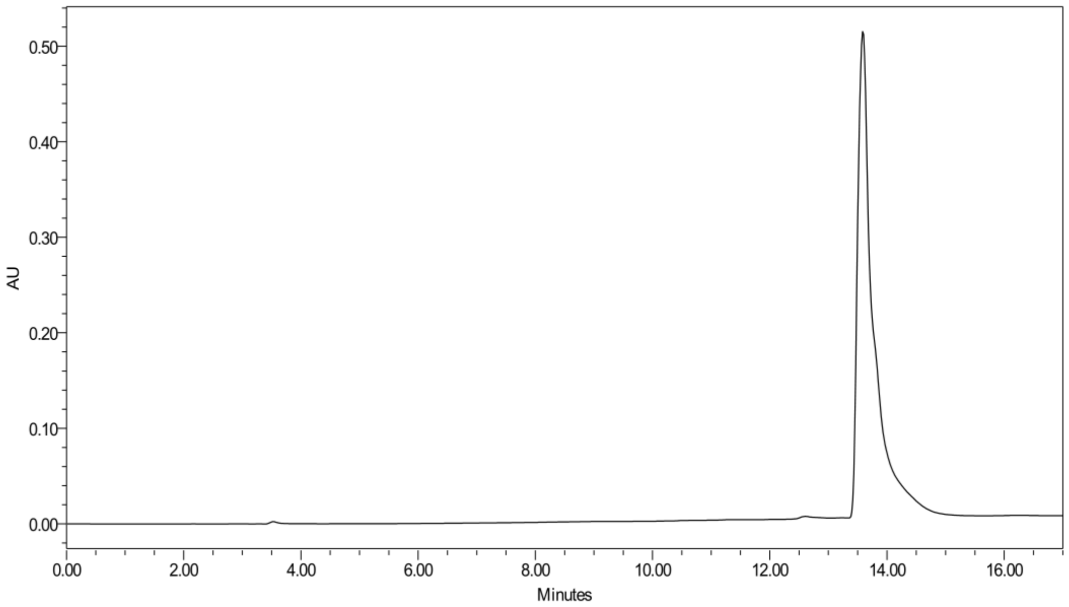

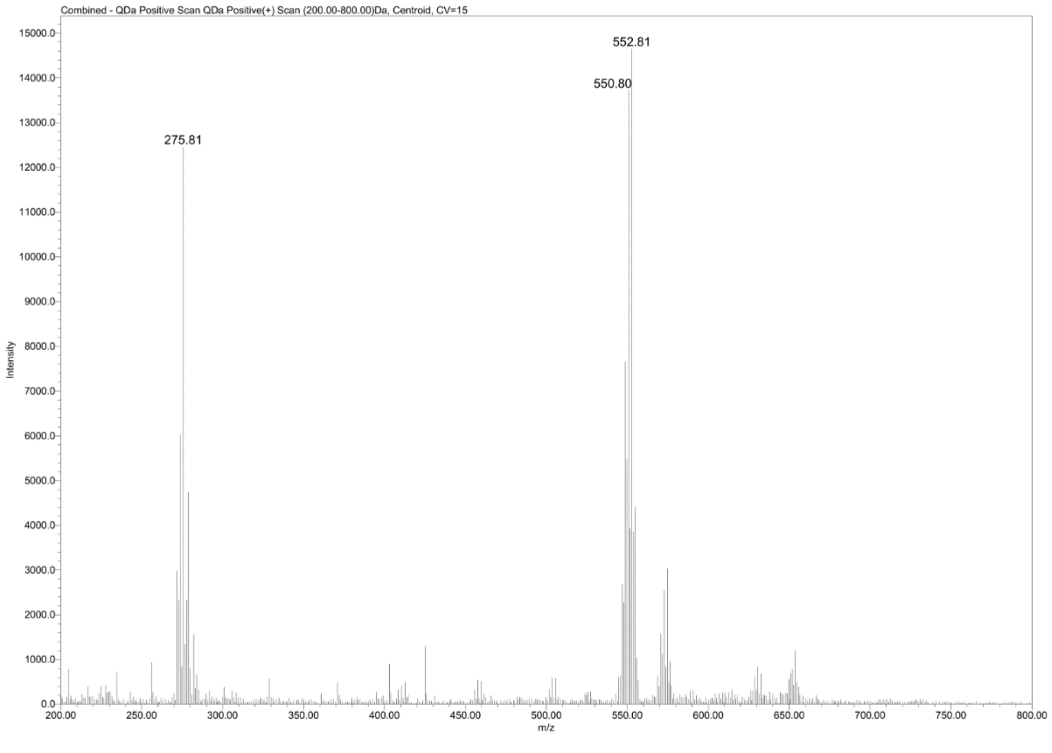

Compound 25

bis[2-(2-fluorophenylcarbamoyl)phenyl] diselenide

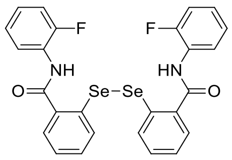

(MS: for $C_{26}H_{18}F_2N_2O_2Se_2 + H^+$ m/z $_{calcd}$ = 588.9740; m/z $_{found}$ = 588.83)

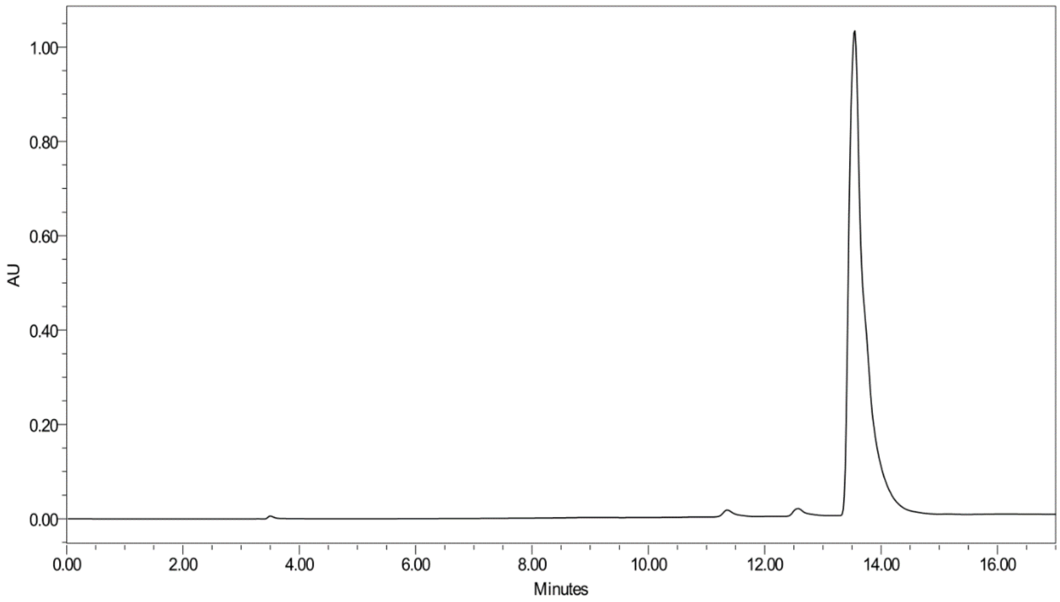

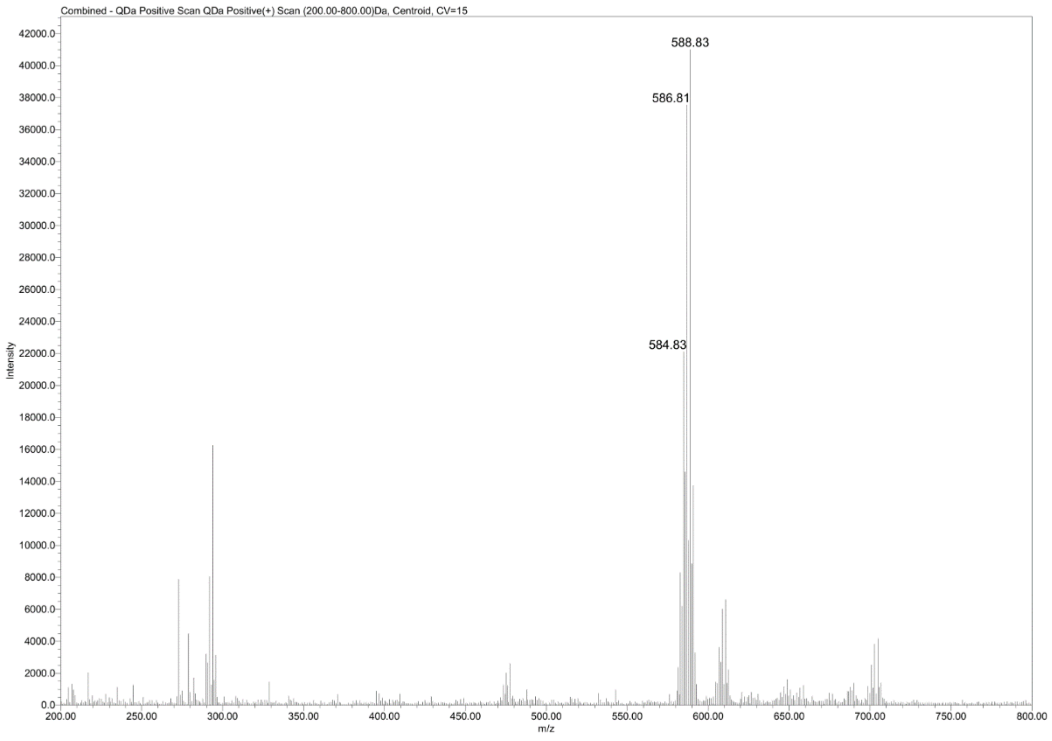

Compound 26

bis[2-(2-trifluoromethylphenylcarbamoyl)phenyl] diselenide

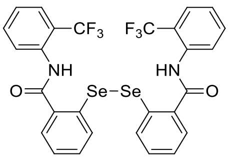

(MS: for $C_{28}H_{18}F_6N_2O_2Se_2 + H^+$ m/z $_{calcd}$ = 688.9676; m/z $_{found}$ = 688.86)

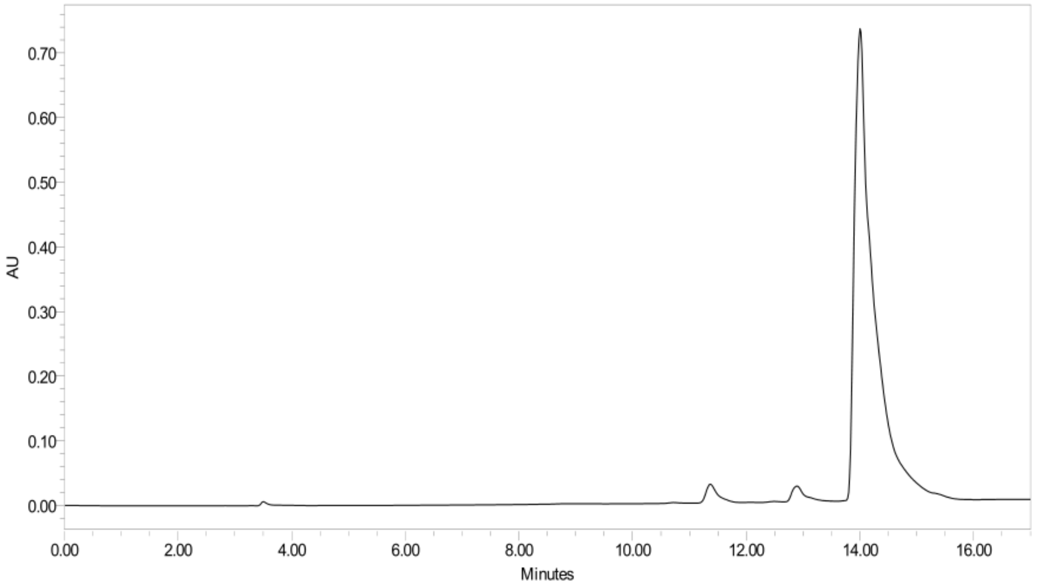

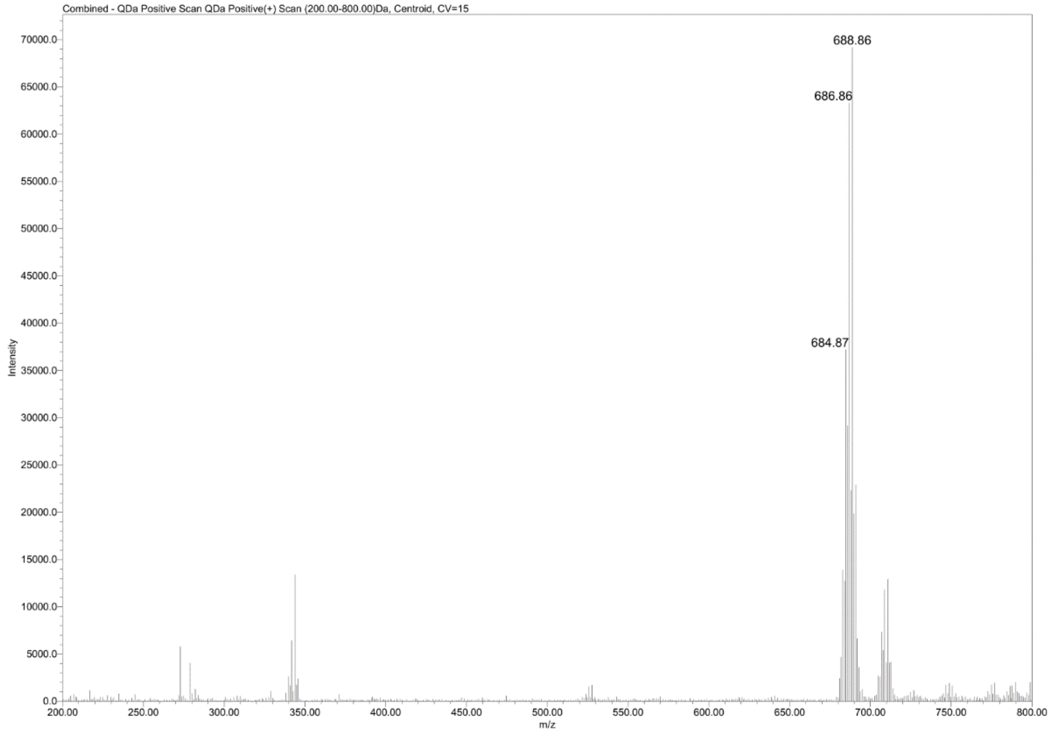

Compound 27

bis[2-(3-fluorophenylcarbamoyl)phenyl] diselenide

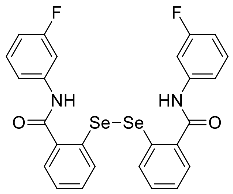

(MS: for $C_{26}H_{18}F_2N_2O_2Se_2 + H^+$ m/z $_{calcd}$ = 588.9740; m/z $_{found}$ = 588.83)

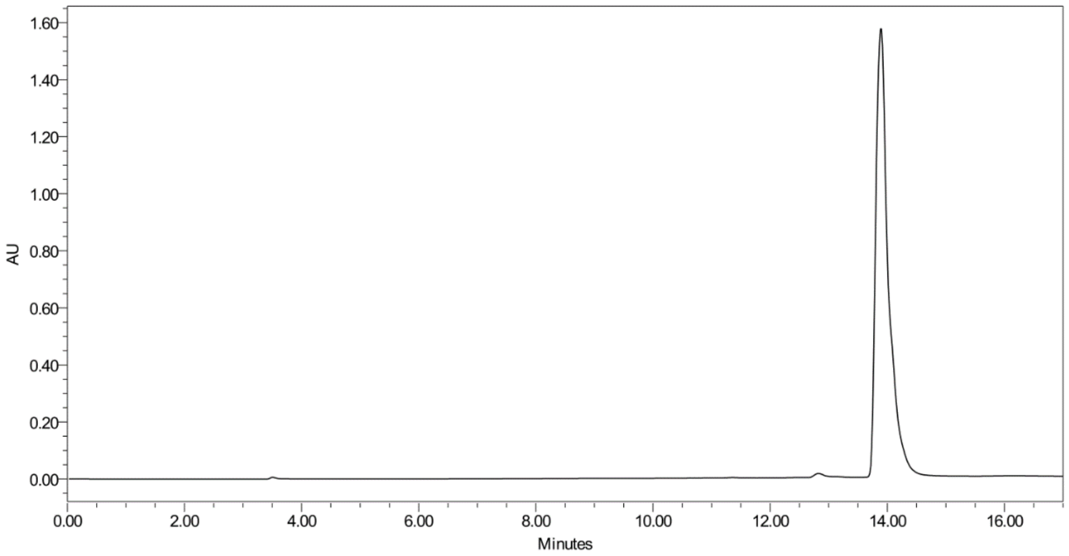

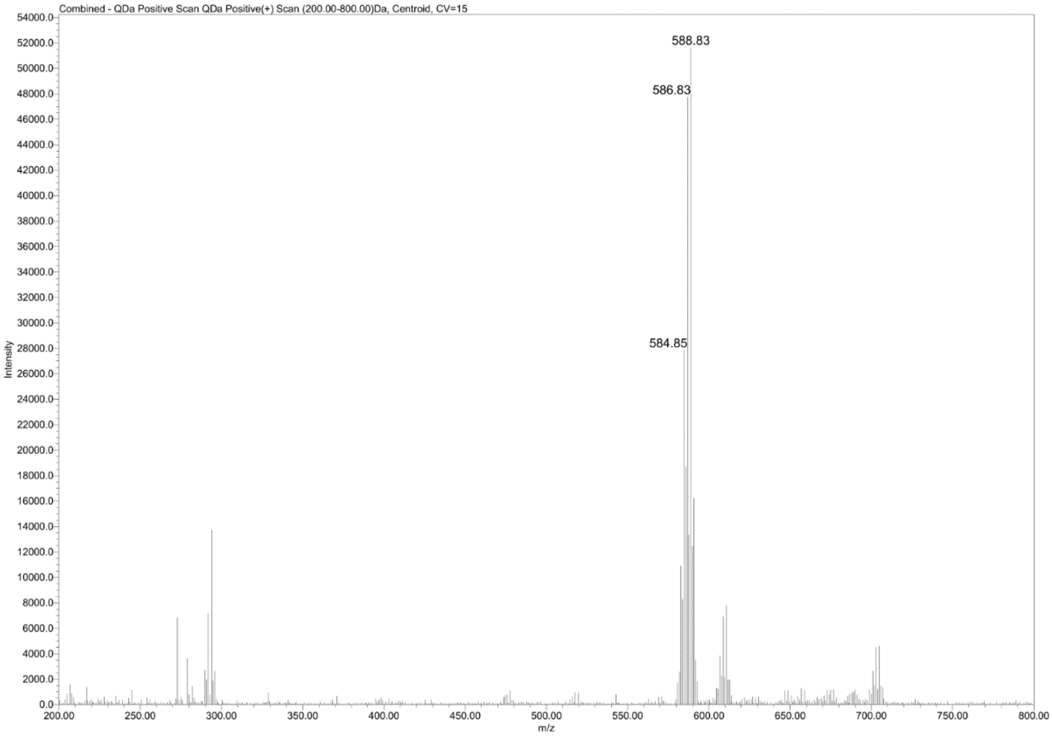

Compound 28

bis[2-(3-methoxyphenylcarbamoyl)phenyl] diselenide

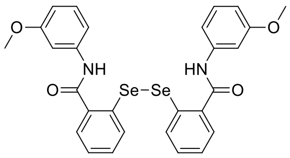

(MS: for $C_{28}H_{24}N_2O_2Se_2 + H^+$ m/z $_{calcd}$ = 613.0140; m/z $_{found}$ = 612.89)

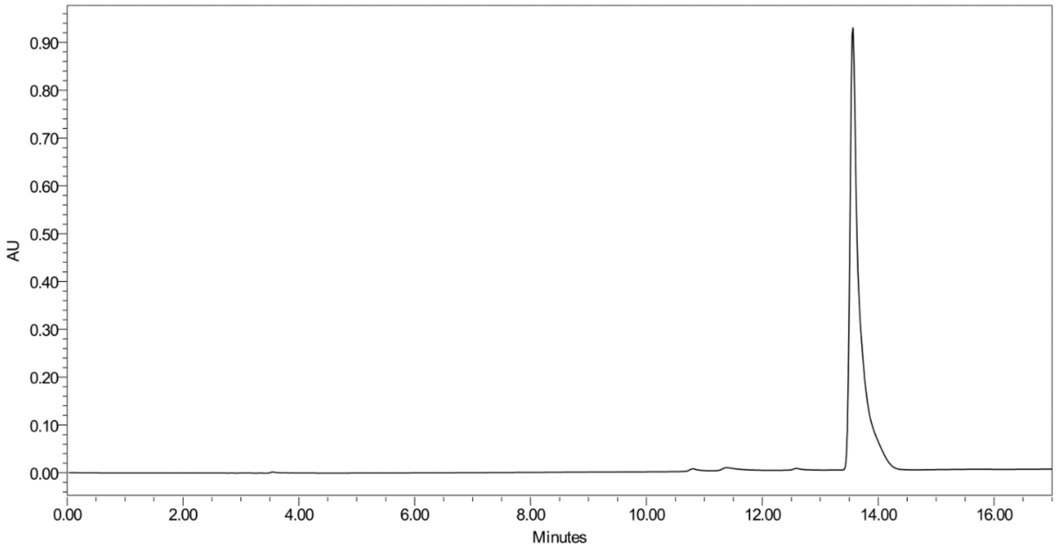

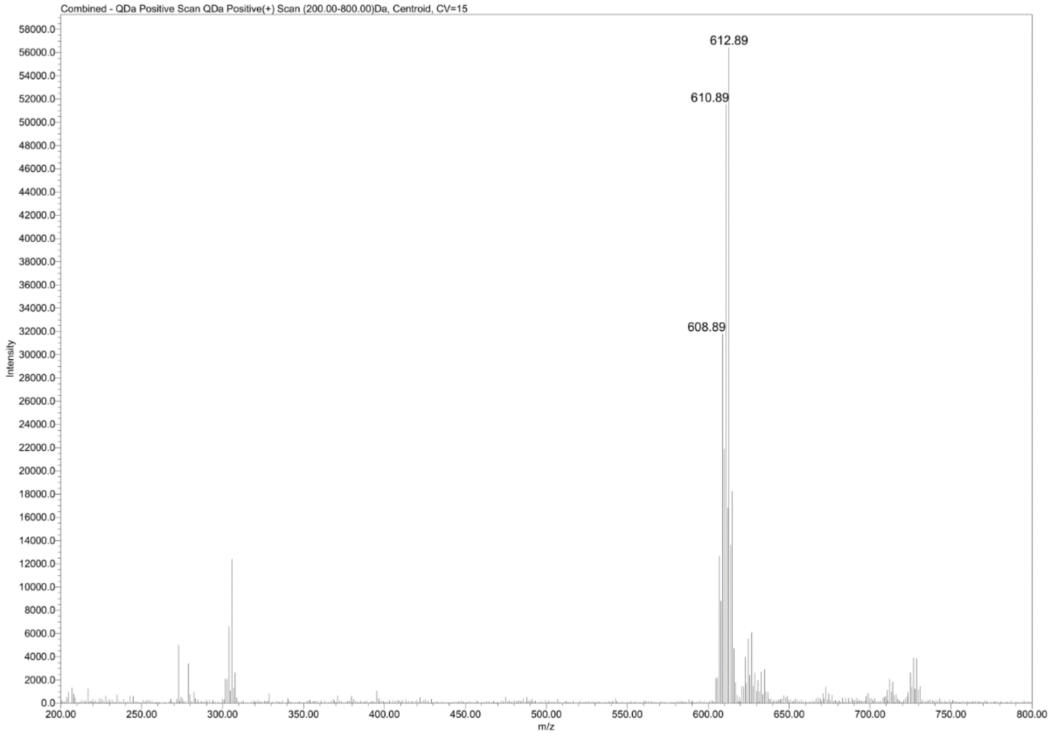

Compound 29

bis[2-(4-trifluoromethylphenylcarbamoyl)phenyl] diselenide

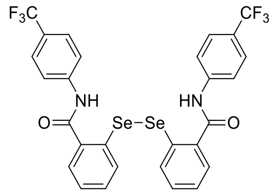

(MS: for $C_{28}H_{18}F_6N_2O_2Se_2 + H^+$ m/z $_{calcd}$ = 688.9676; m/z $_{found}$ = 688.87)

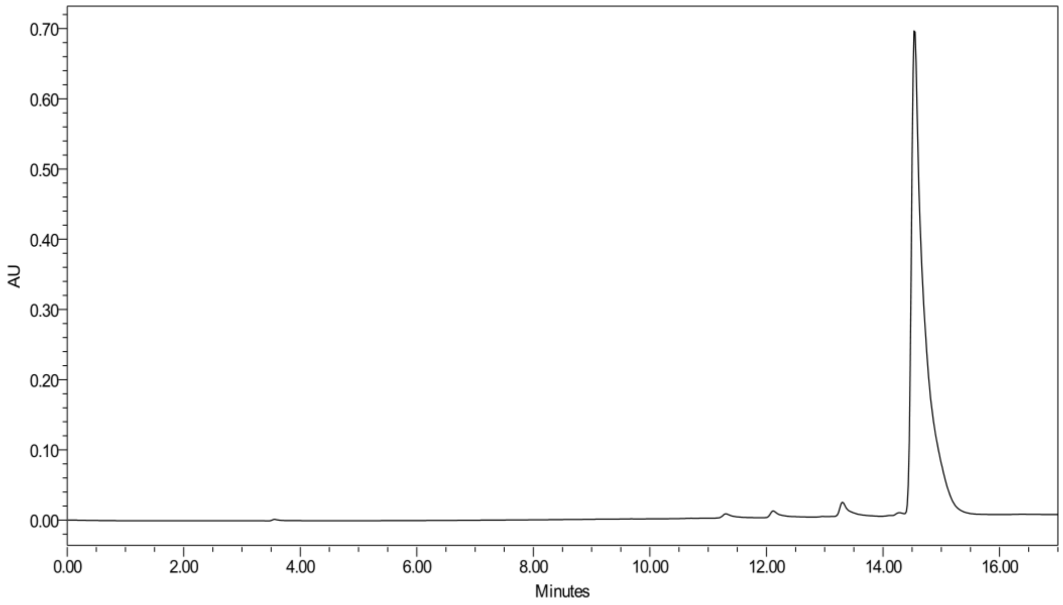

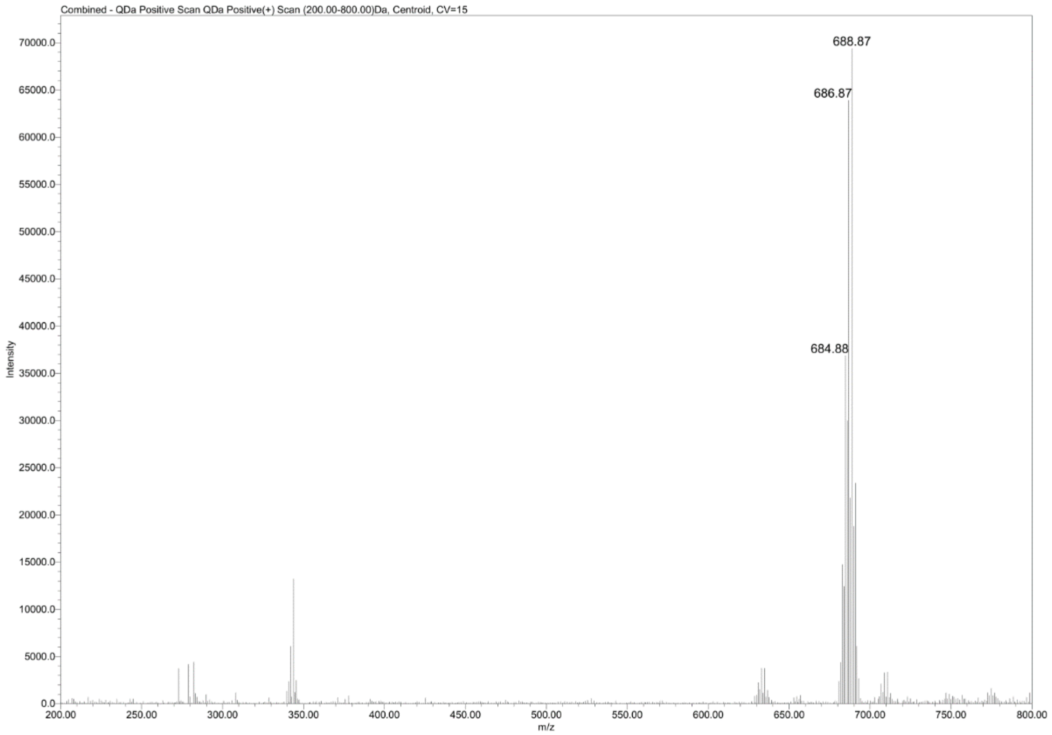

Compound 30

bis[2-(4-chloro-2-fluorophenylcarbamoyl)phenyl] diselenide

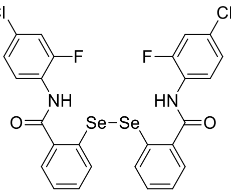

(MS: for $C_{26}H_{16}Cl_2F_2N_2O_2Se_2 + H^+$ m/z $_{calcd}$ = 613.0140; m/z $_{found}$ = 612.89)

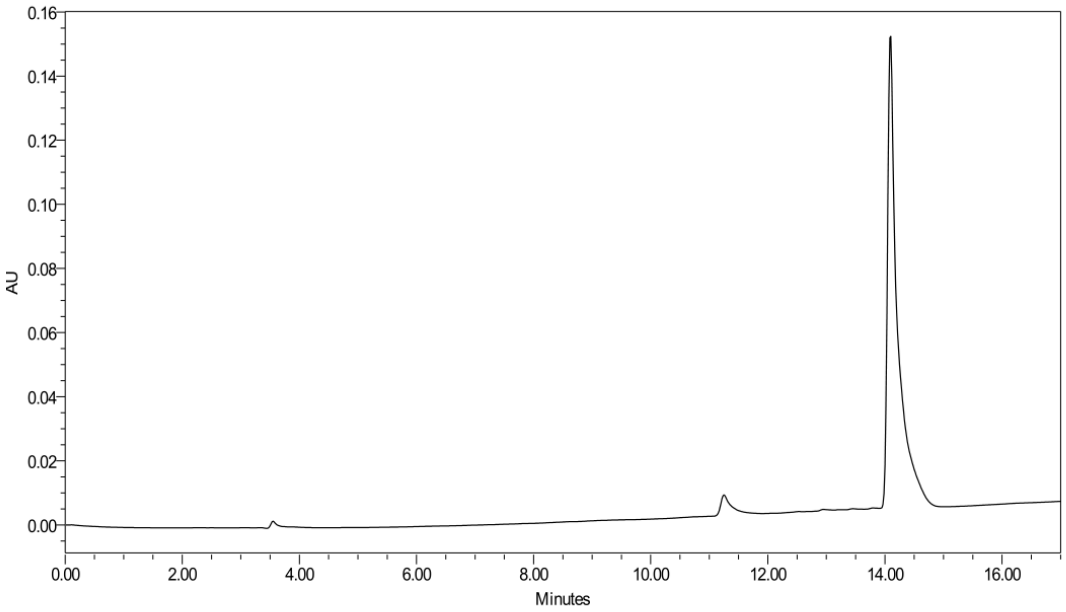

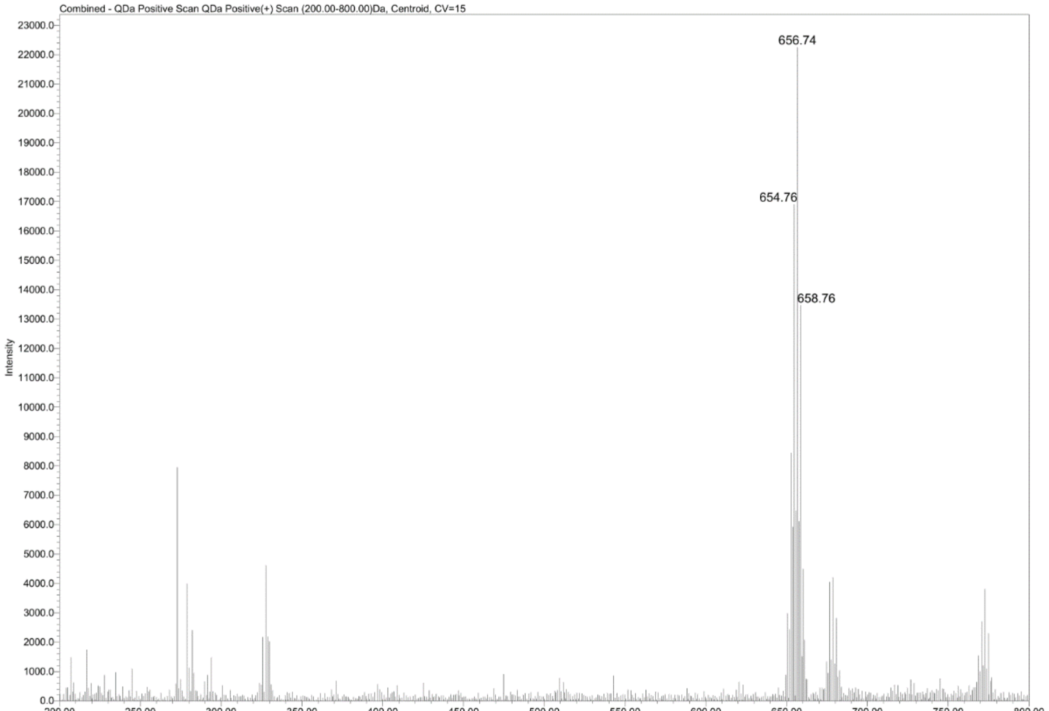

Compound 31

bis[2-(2,4-dimethoxyphenylcarbamoyl)phenyl] diselenide

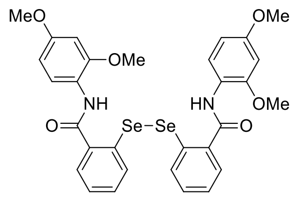

(MS: for $C_{30}H_{28}N_2O_6Se_2 + H^+$ m/z $_{calcd}$ = 673.0351; m/z $_{found}$ = 672.93)

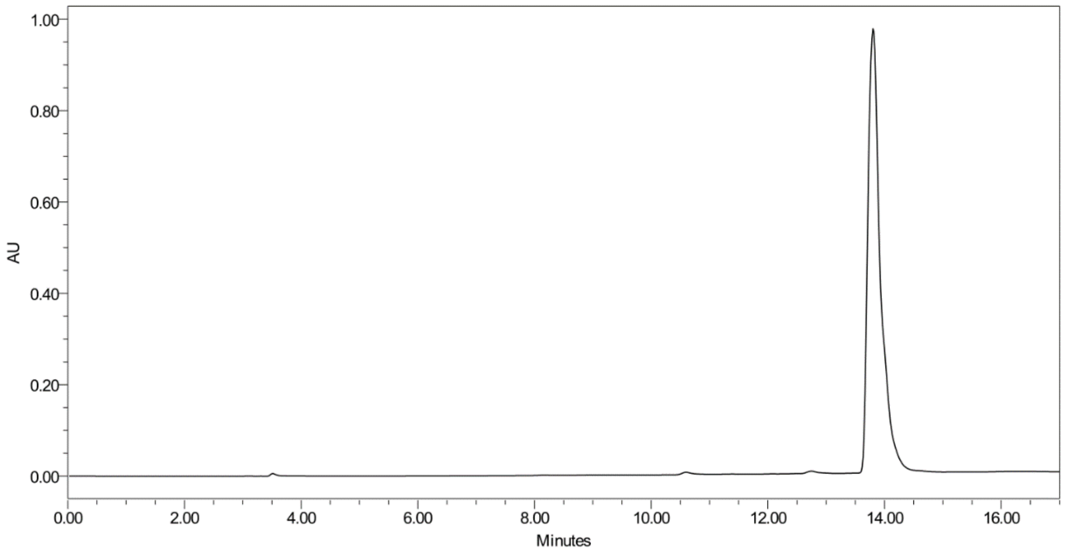

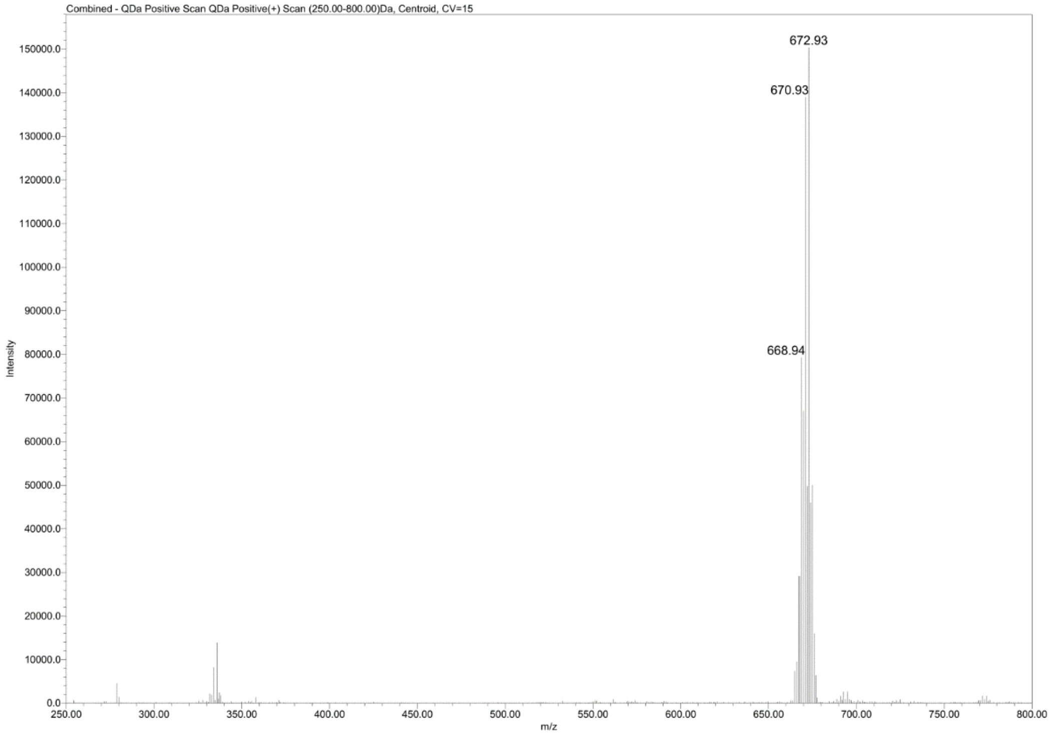

Compound 32

bis[2-(5-chloro-2-methylphenylcarbamoyl)phenyl] diselenide

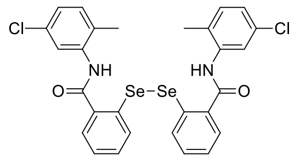

(MS: for $C_{28}H_{22}Cl_2N_2O_2Se_2 + H^+$ m/z $_{calcd}$ = 648.9462; m/z $_{found}$ = 648.81)

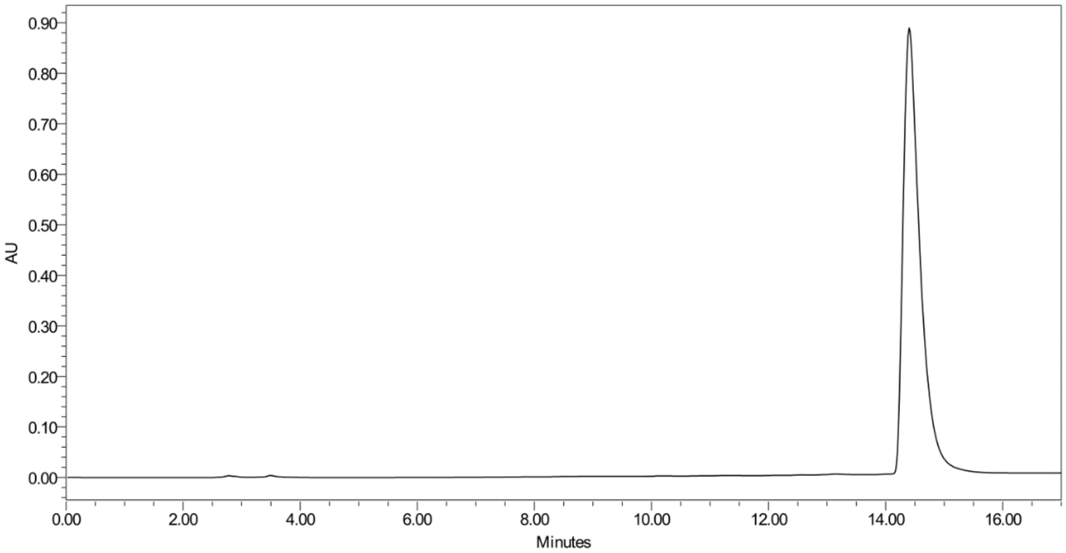

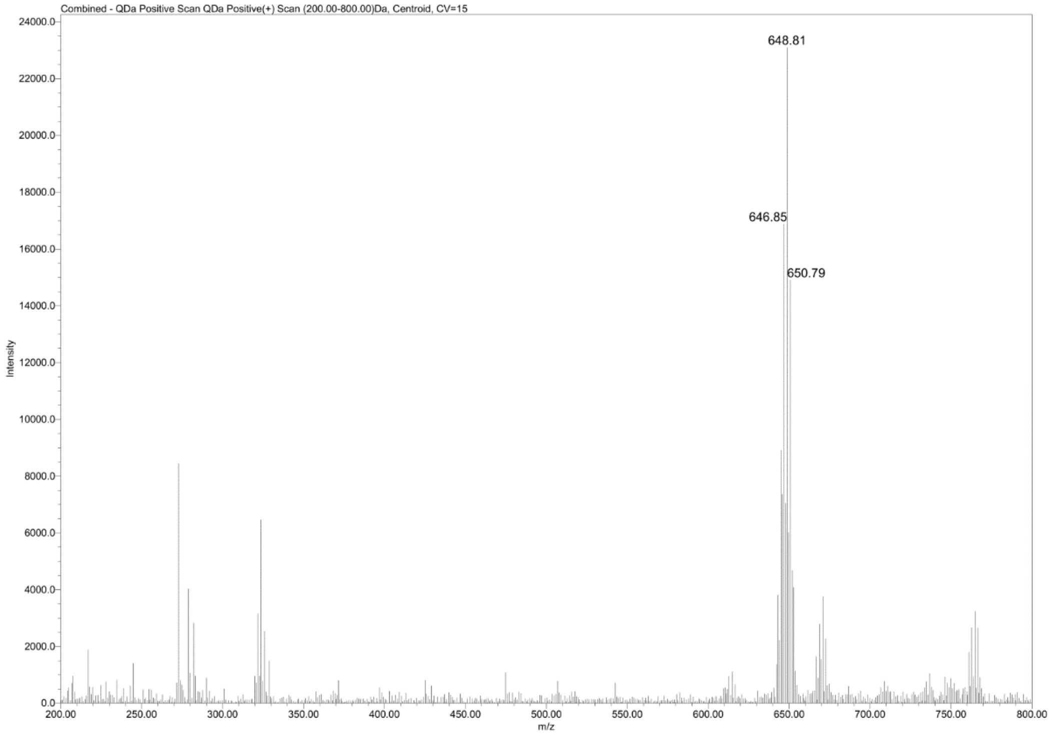

Compound 33

bis[2-(4-chloro-3-methylphenylcarbamoyl)phenyl] diselenide

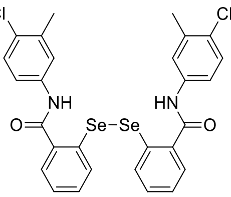

(MS: for $C_{28}H_{22}Cl_2N_2O_2Se_2 + H^+$ m/z $_{calcd}$ = 648.9462; m/z $_{found}$ = 648.83)

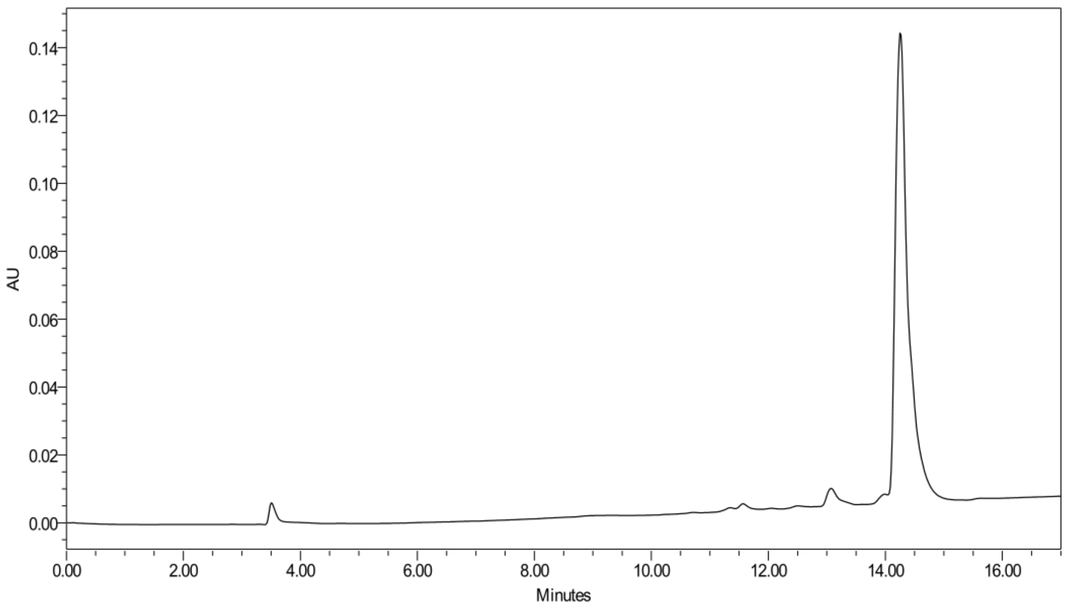

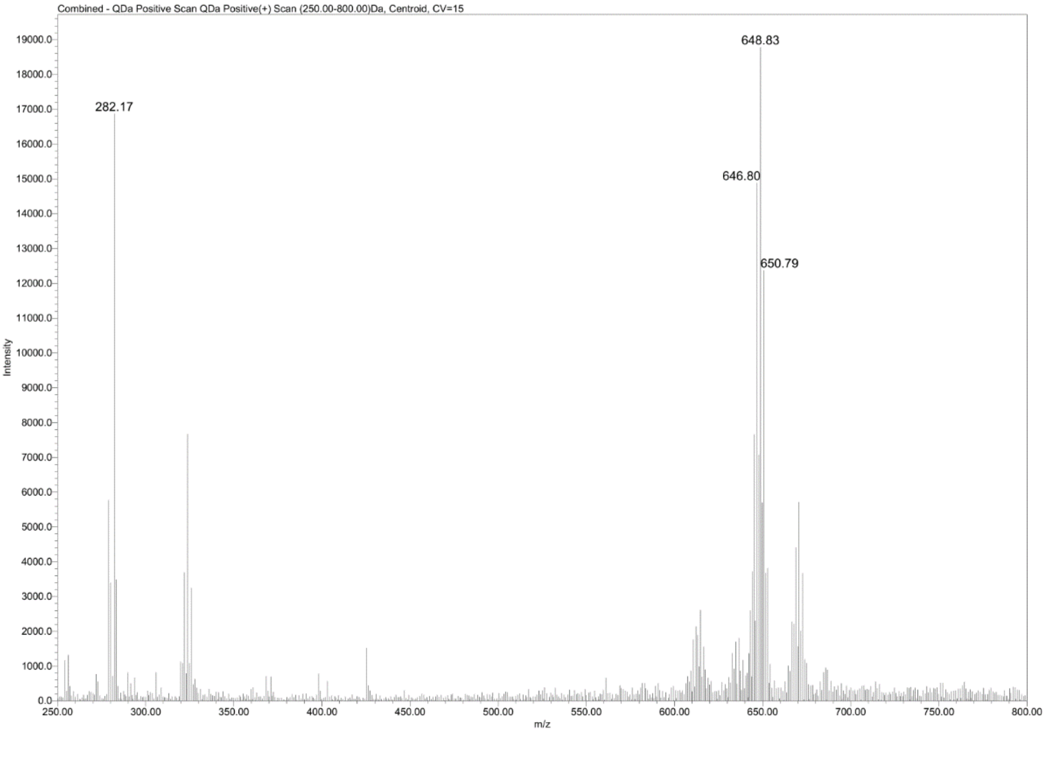

Compound 34

bis[2-(2-chloro-6-fluorophenylcarbamoyl)phenyl] diselenide

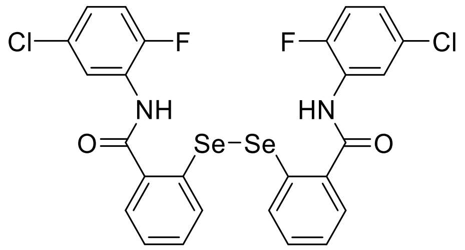

(MS: for $C_{26}H_{16}Cl_2F_2N_2O_2Se_2 + H^+$ m/z $_{calcd}$ = 656.8961; m/z $_{found}$ = 656.78)

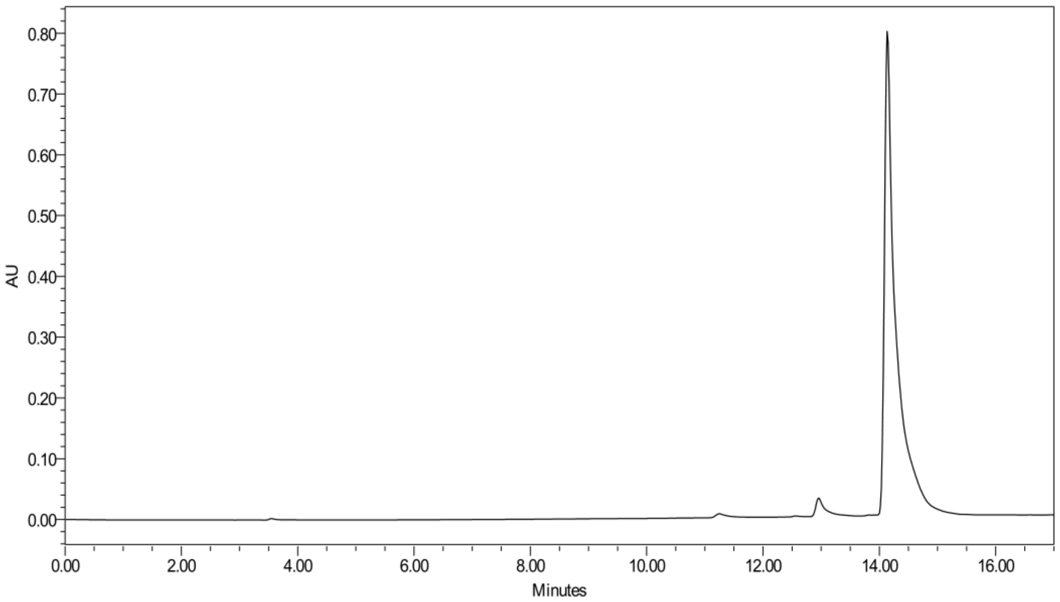

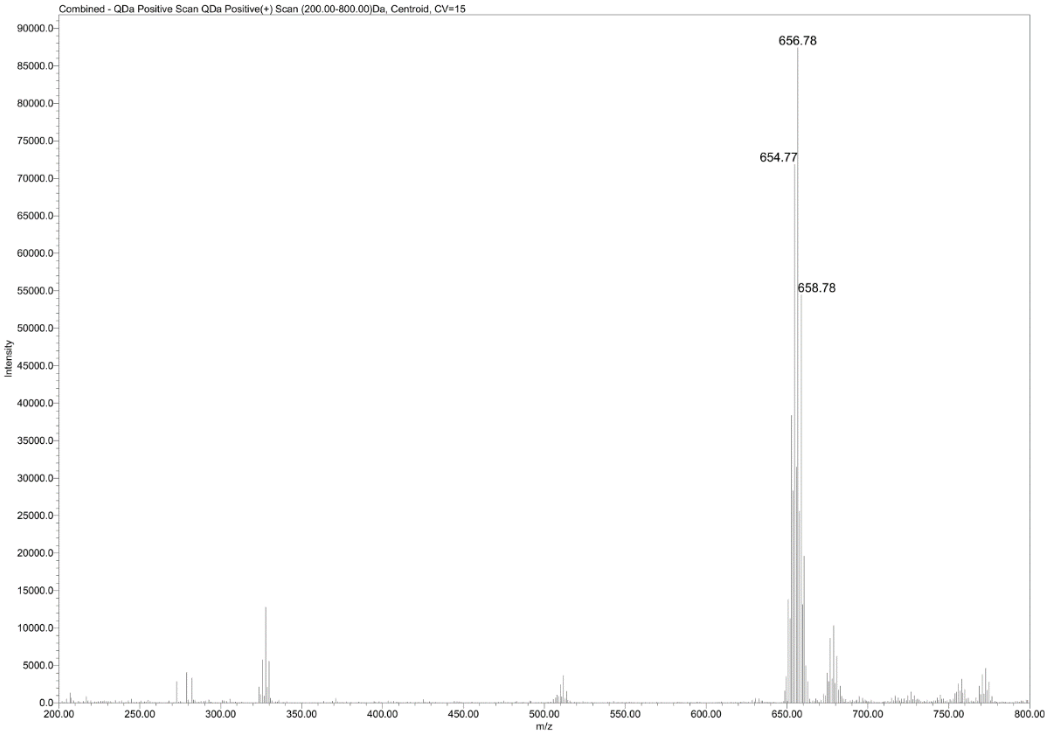

NMR spectra of the compounds

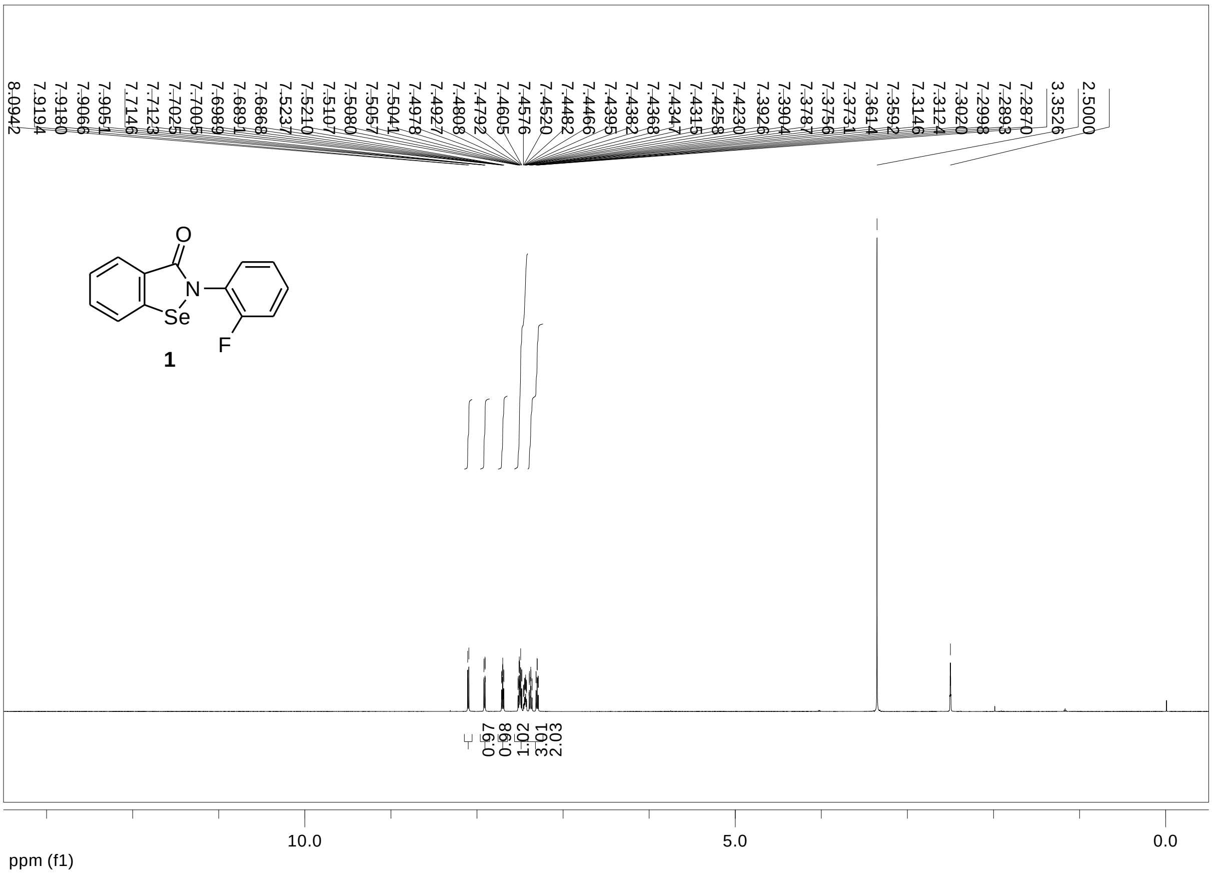

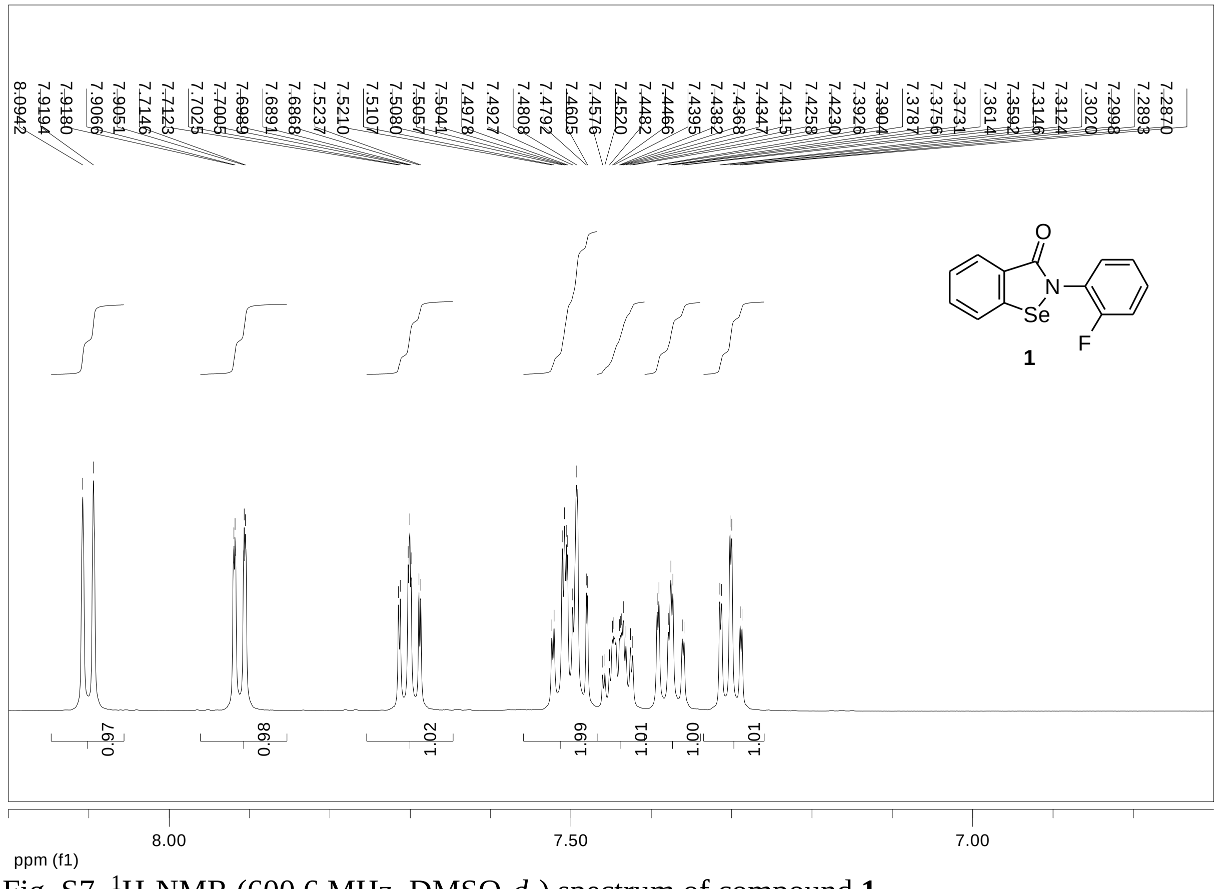

Fig. S7. ^{1}H-NMR (600.6 MHz, DMSO-d_6) spectrum of compound **1**

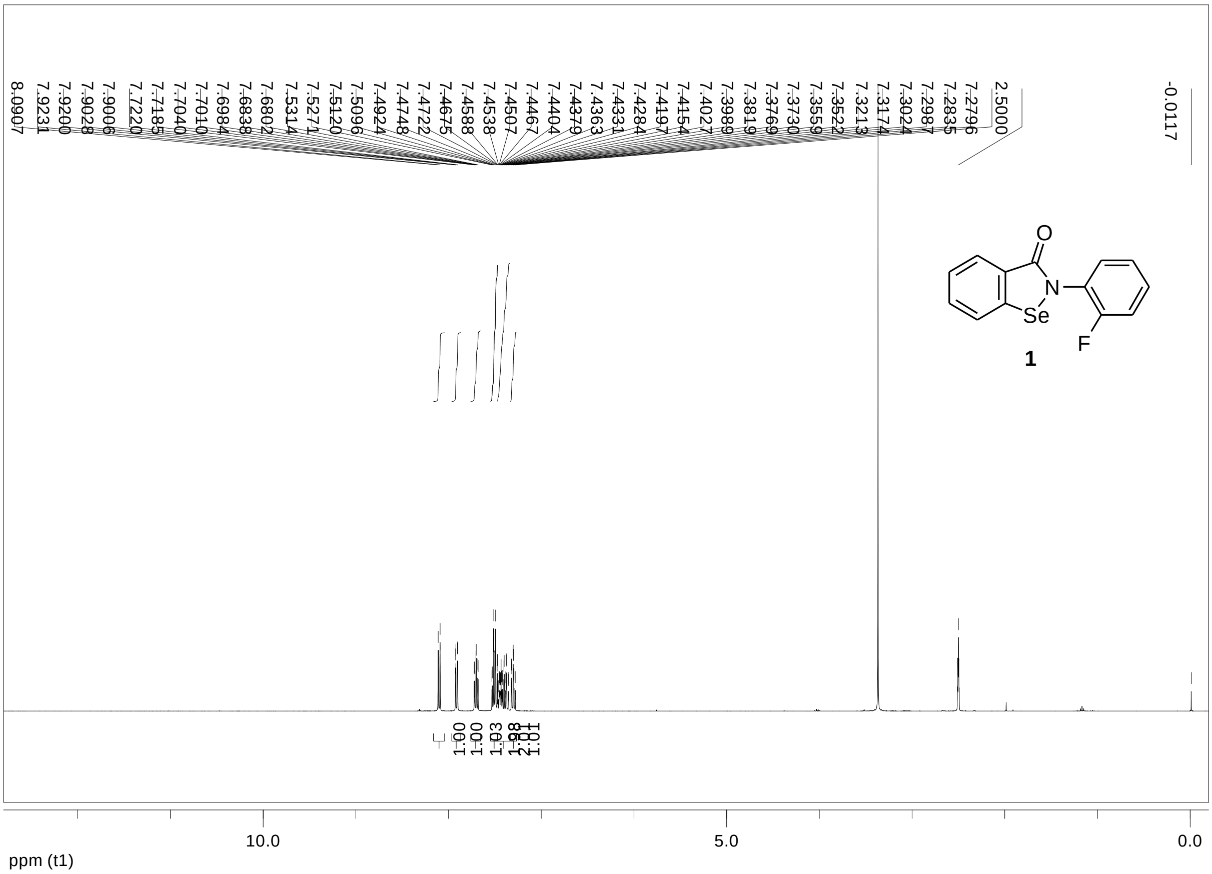

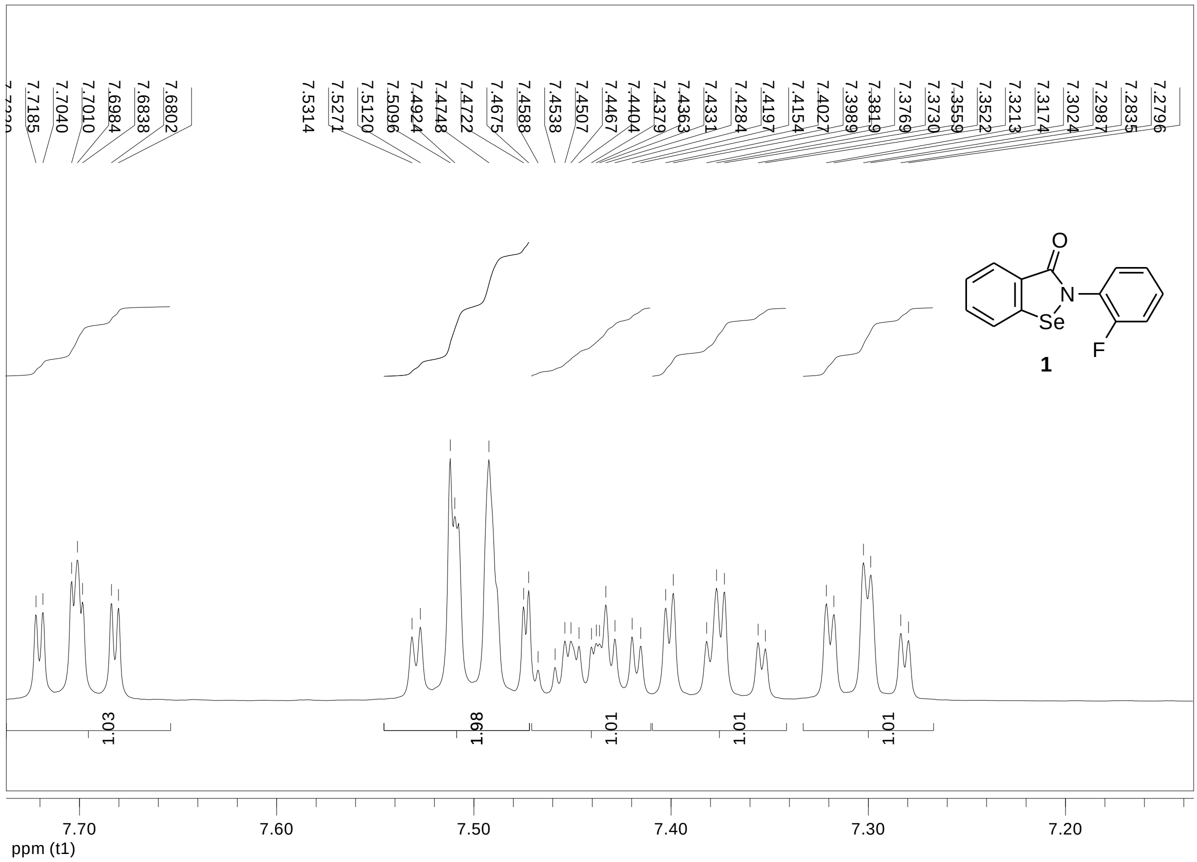

Fig. S8. ^{1}H-NMR (399.8 MHz, DMSO-d_6) spectrum of compound **1**

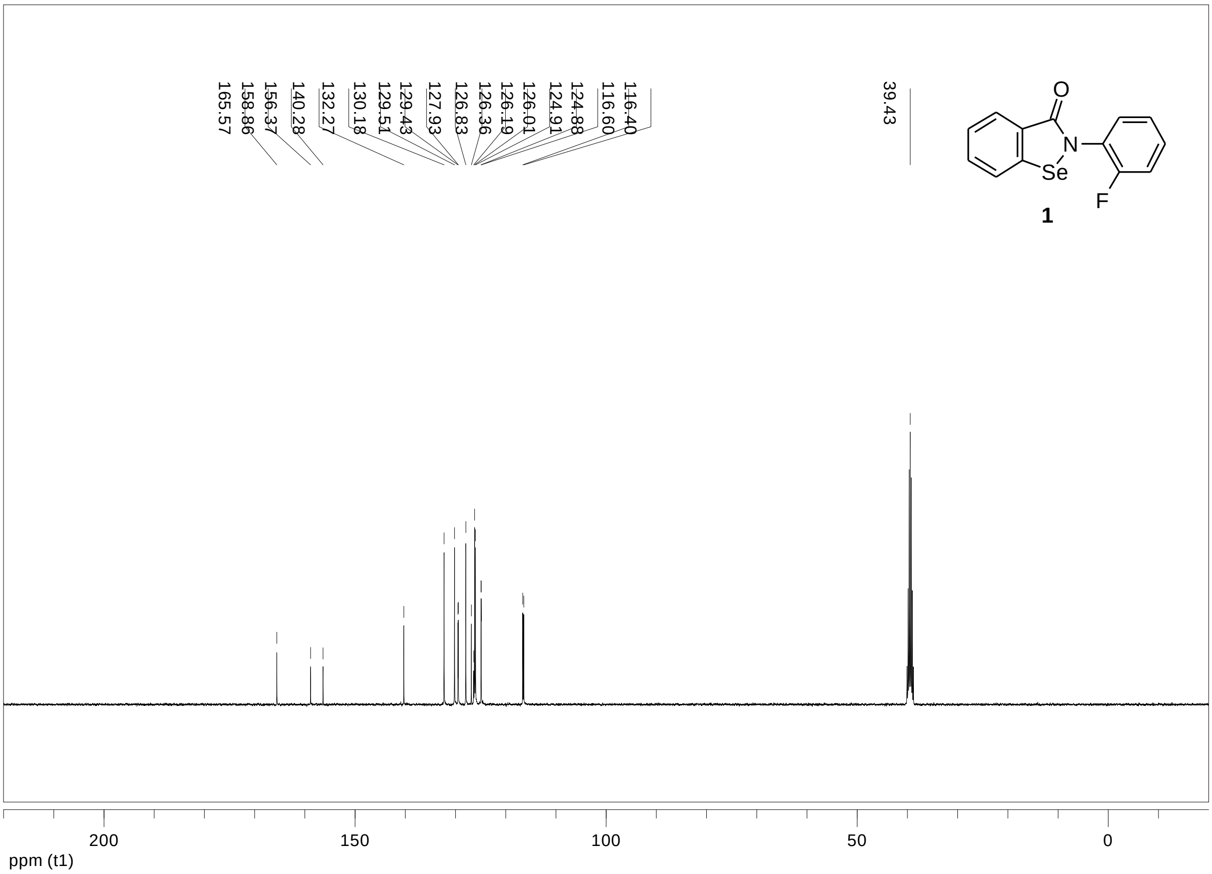
165.57
158.86
156.37
140.28
132.27
130.18
129.51
129.43
127.93
126.83
126.36
126.19
126.01
124.91
124.88
116.60
116.40
39.43
O
N
Se
F
1
200
150
100
50
0
ppm (t1)

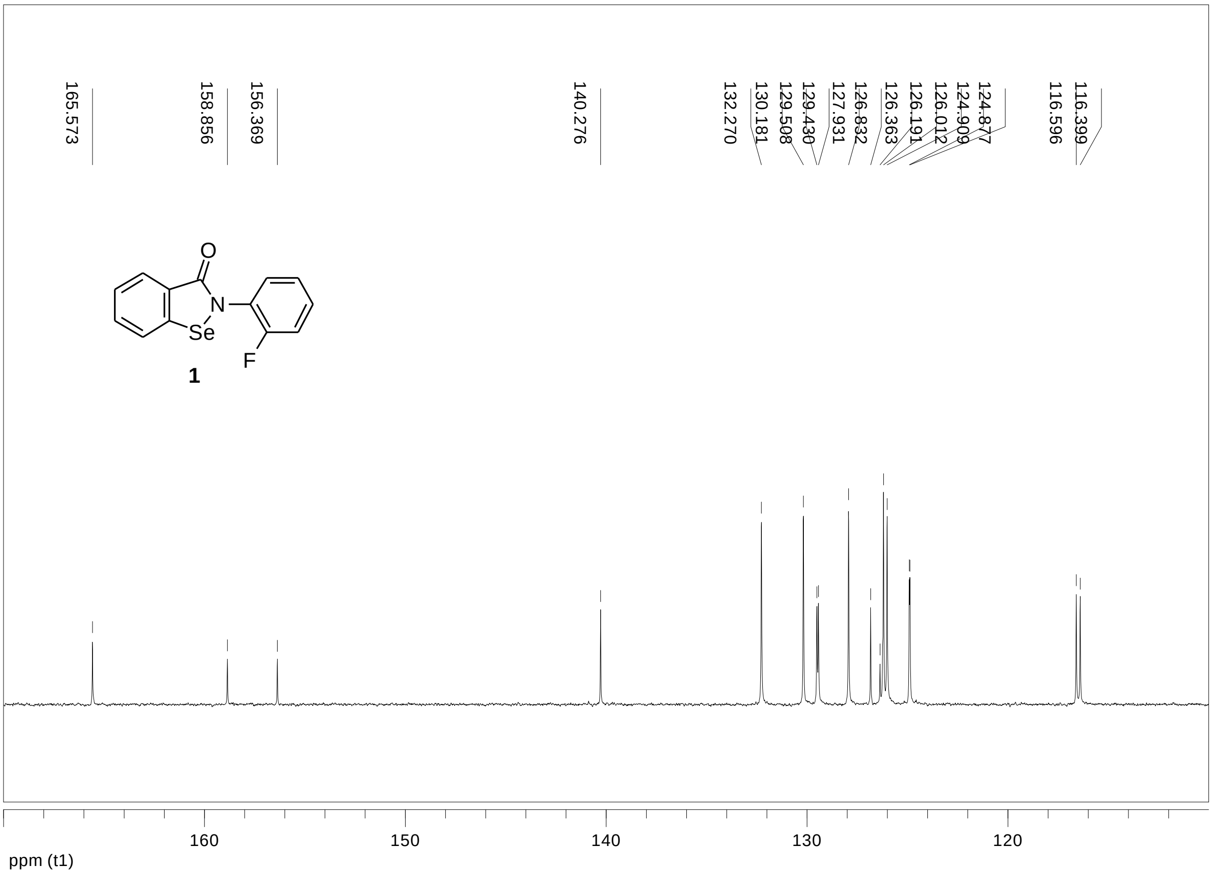
165.573
158.856
156.369
140.276
132.270
130.181
129.508
129.430
127.931
126.832
126.363
126.191
126.012
124.909
124.877
116.596
116.399
O
N
Se
F
1
160
150
140
130
120
ppm (t1)

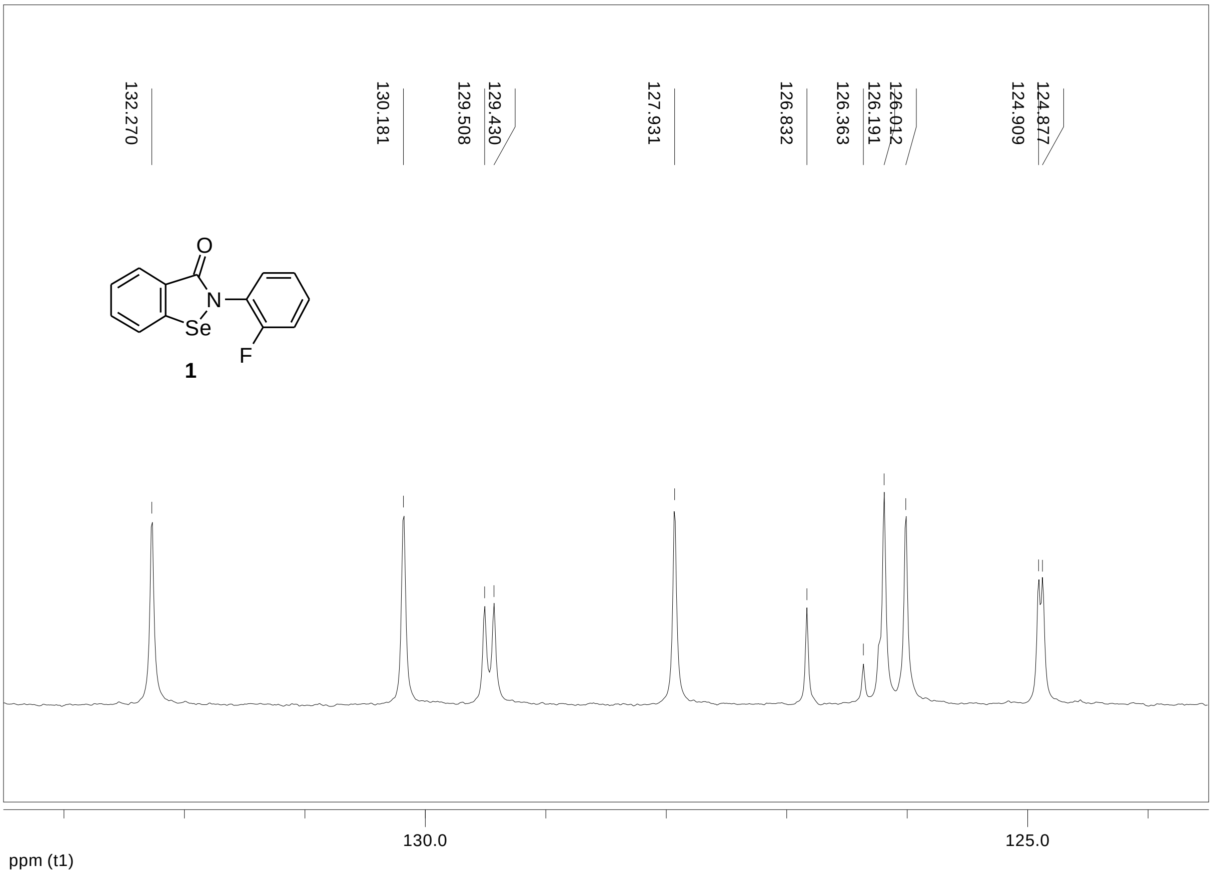

Fig. S9. ^{13}C-NMR (100.52 MHz, DMSO-d_6) spectrum of compound **1**

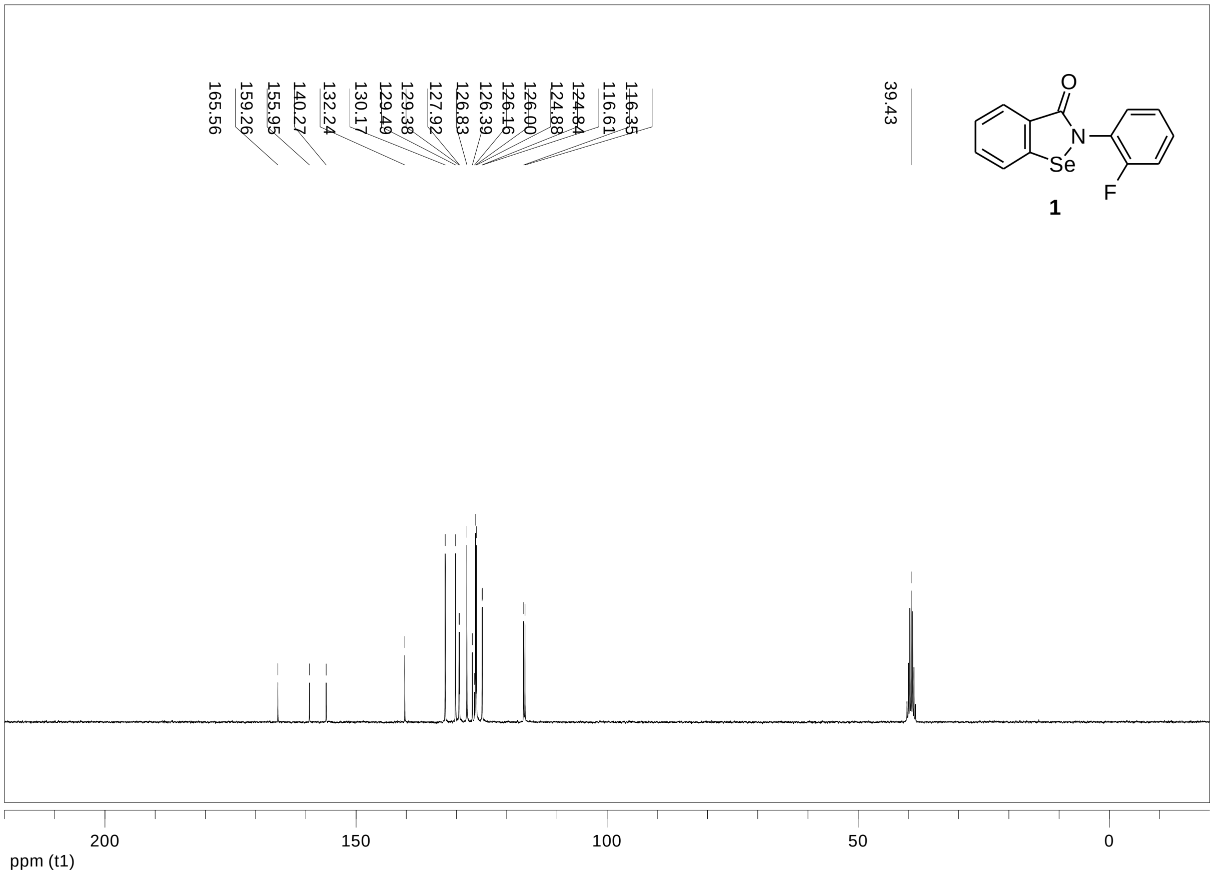

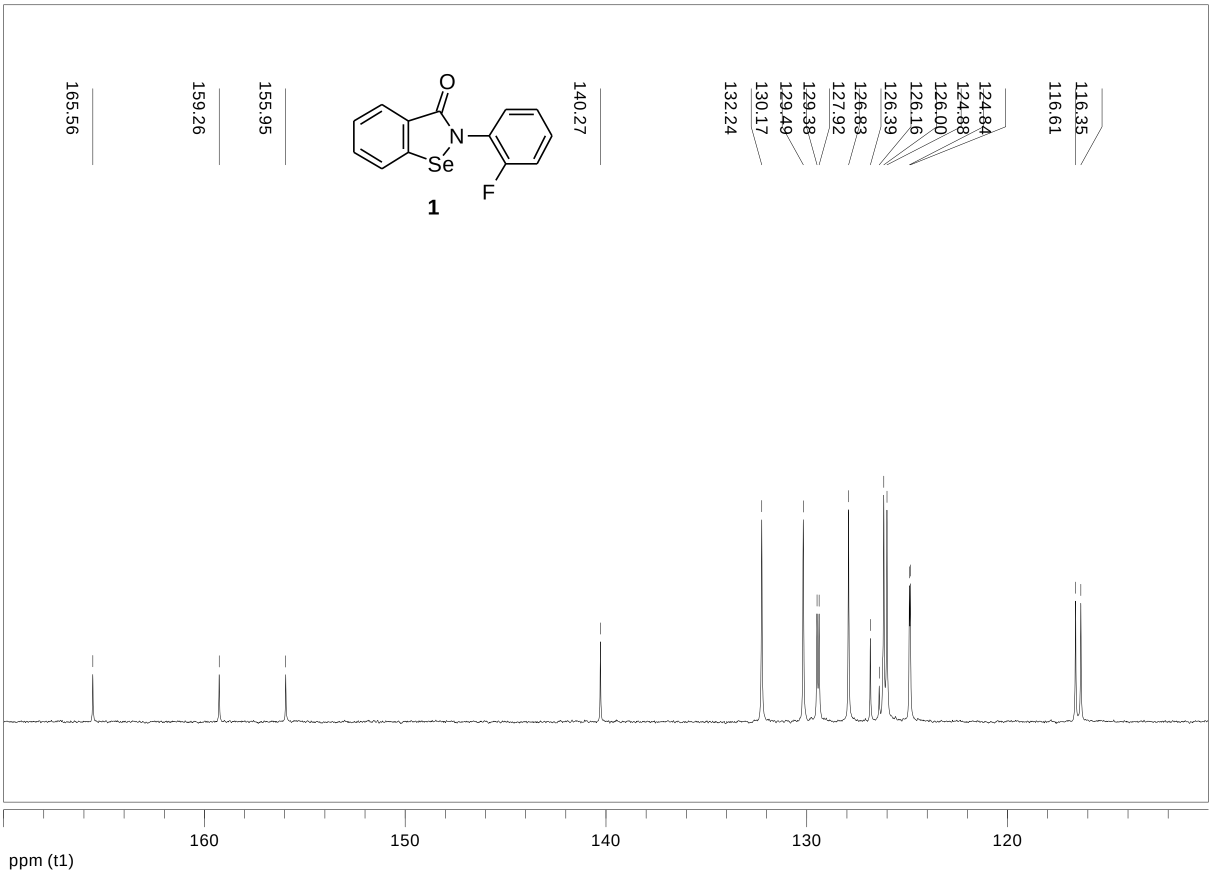

Fig. S10. ^{13}C-NMR (75.45 MHz, DMSO-d_6) spectrum of compound **1**

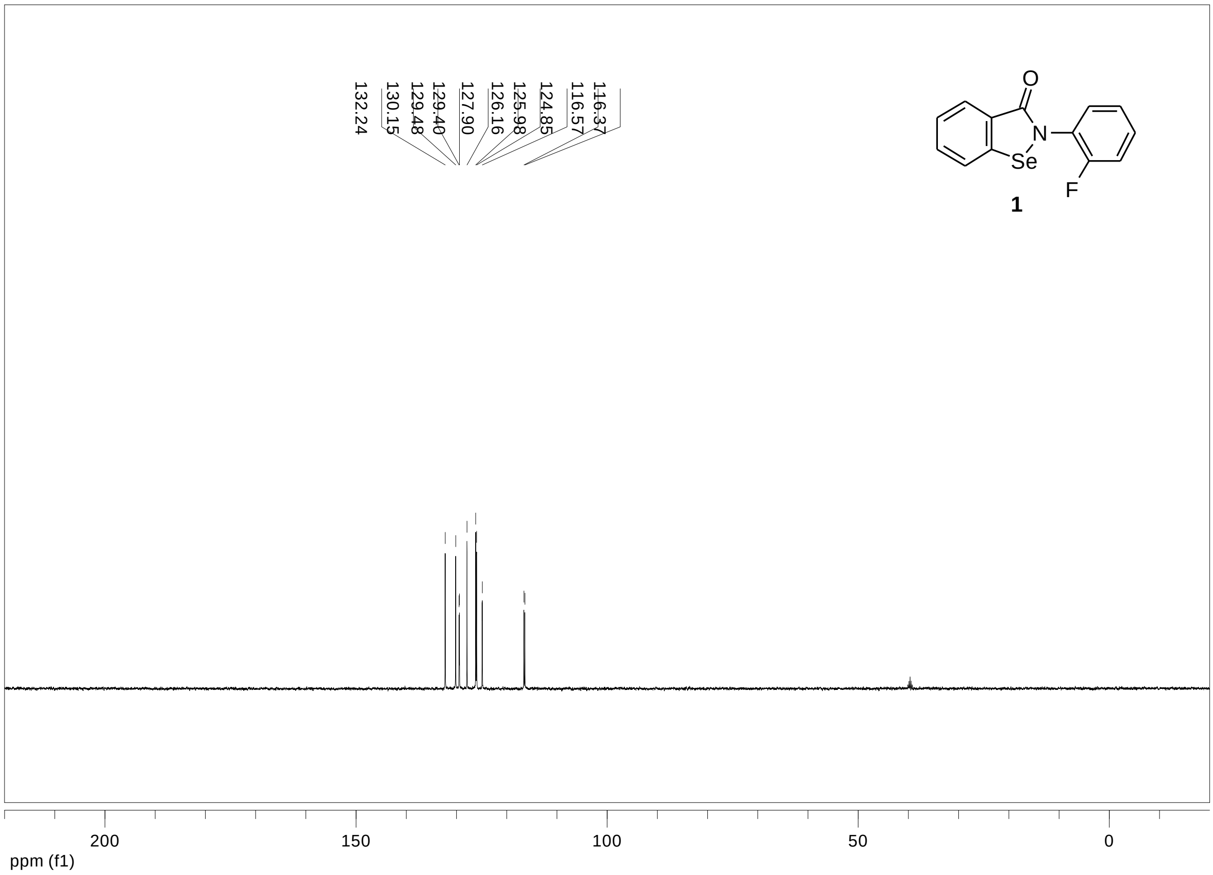

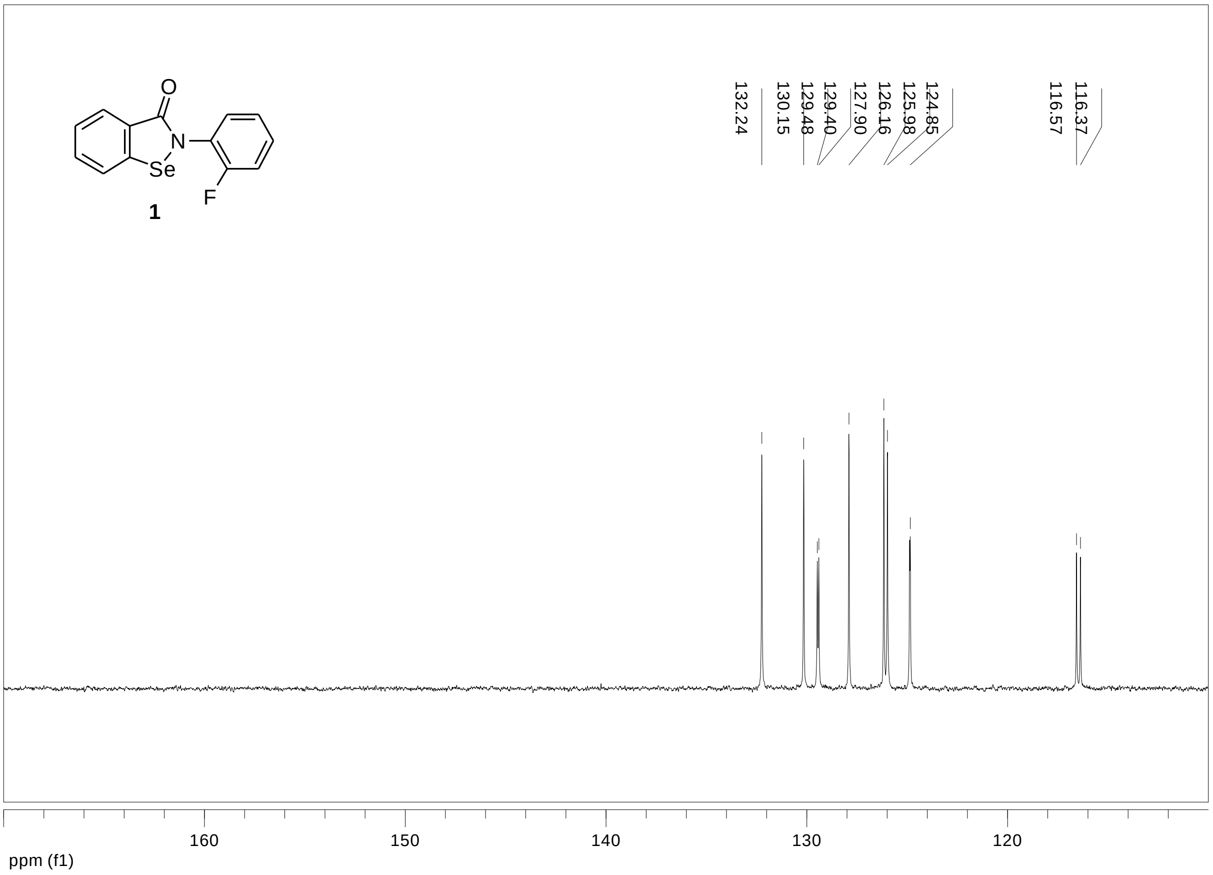

Fig. S11. ^{13}C-NMR (100.52 MHz, DMSO-d_6) dept-135 experiment of compound **1**

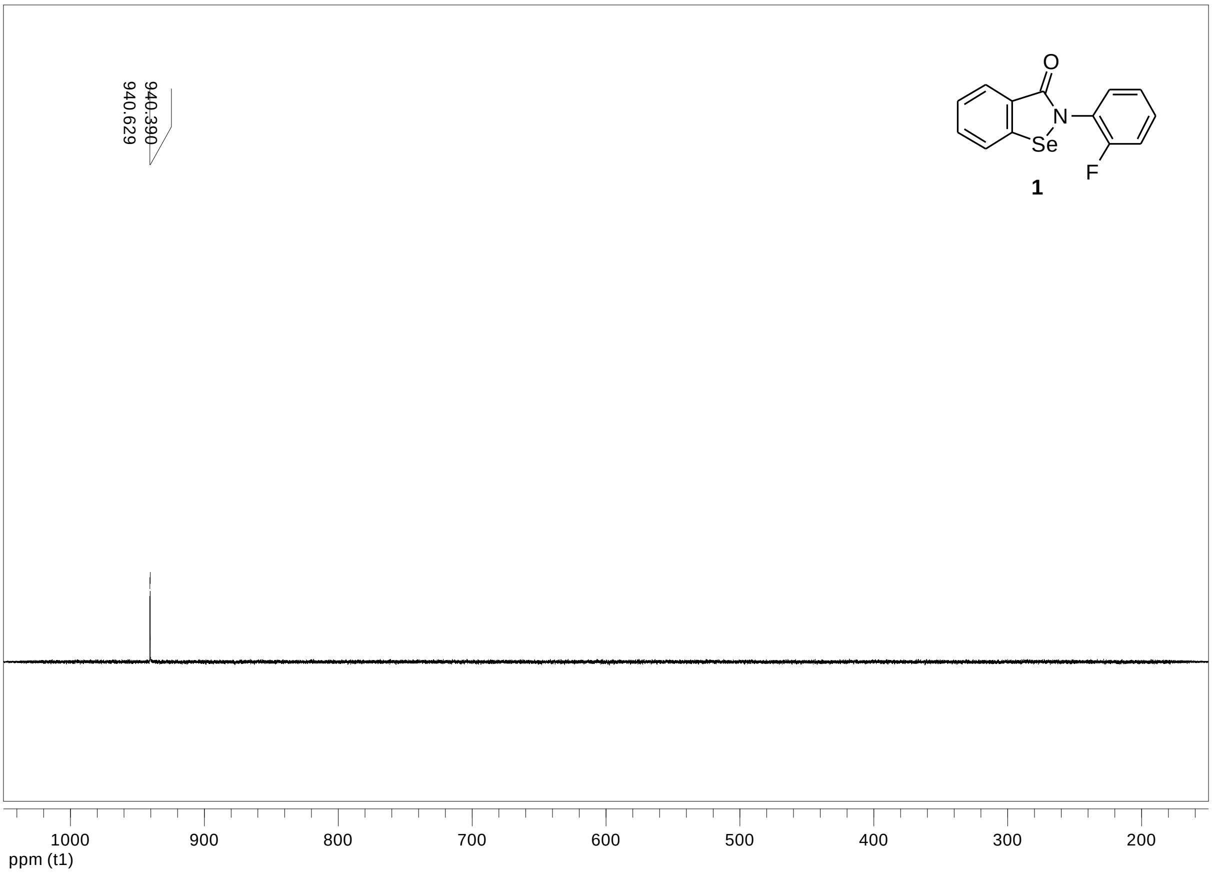

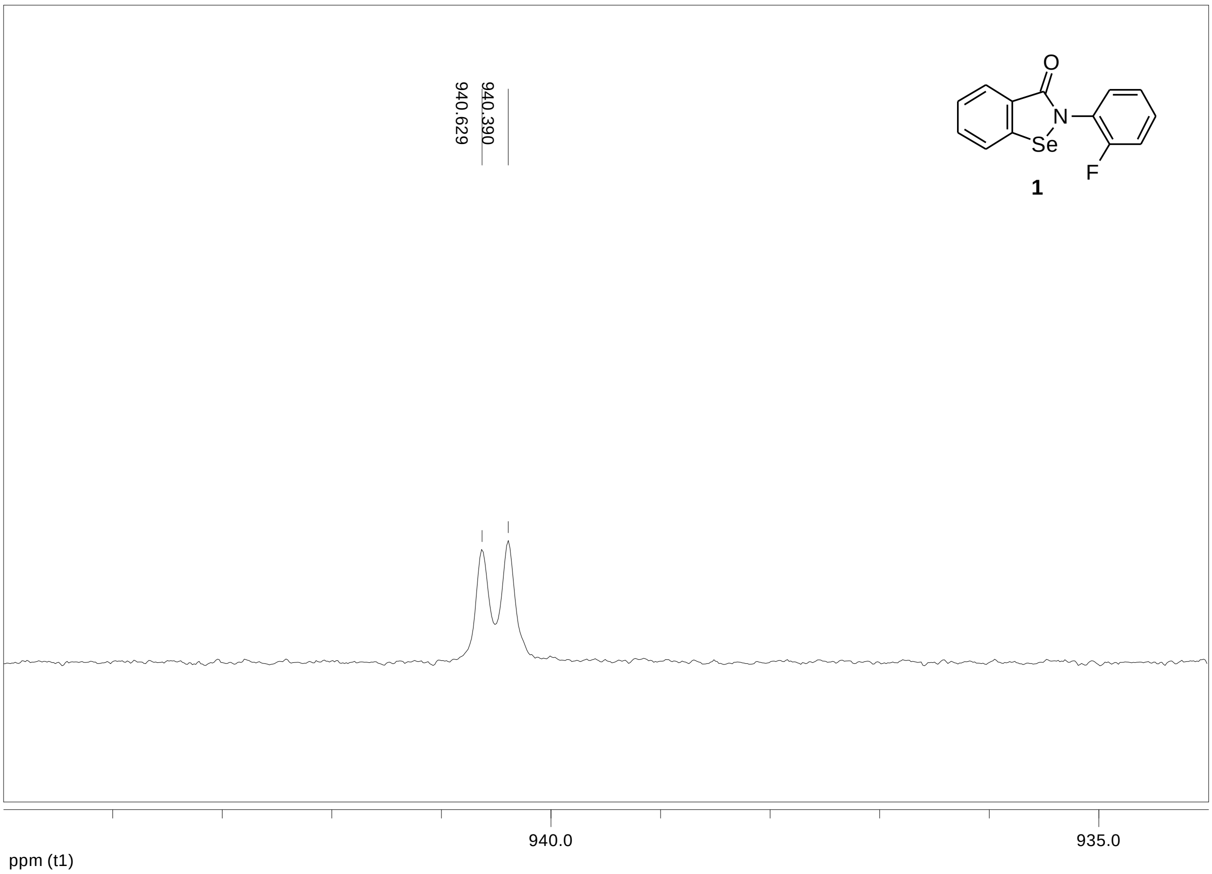

Fig. S12. ^{77}Se-NMR (76.24 MHz, DMSO-d_6) spectrum of compound **1**

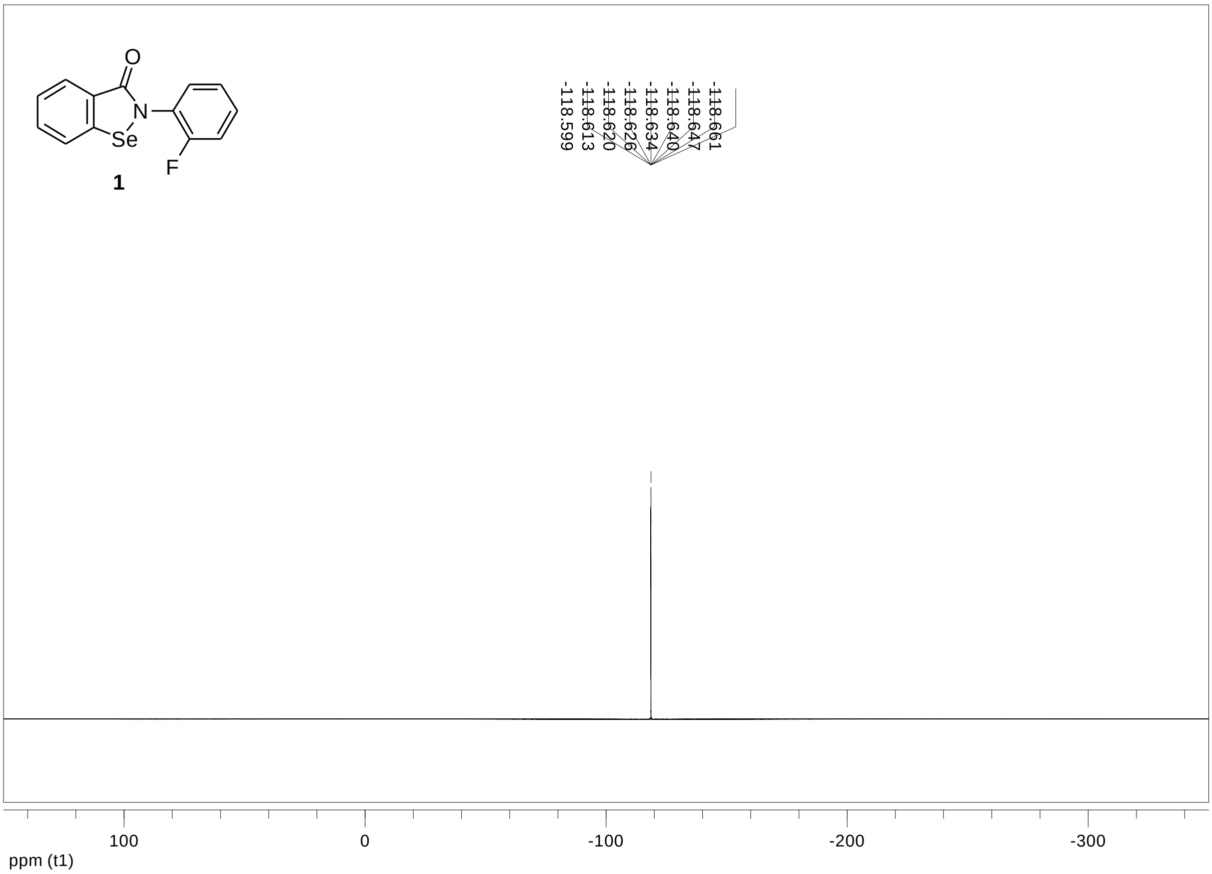

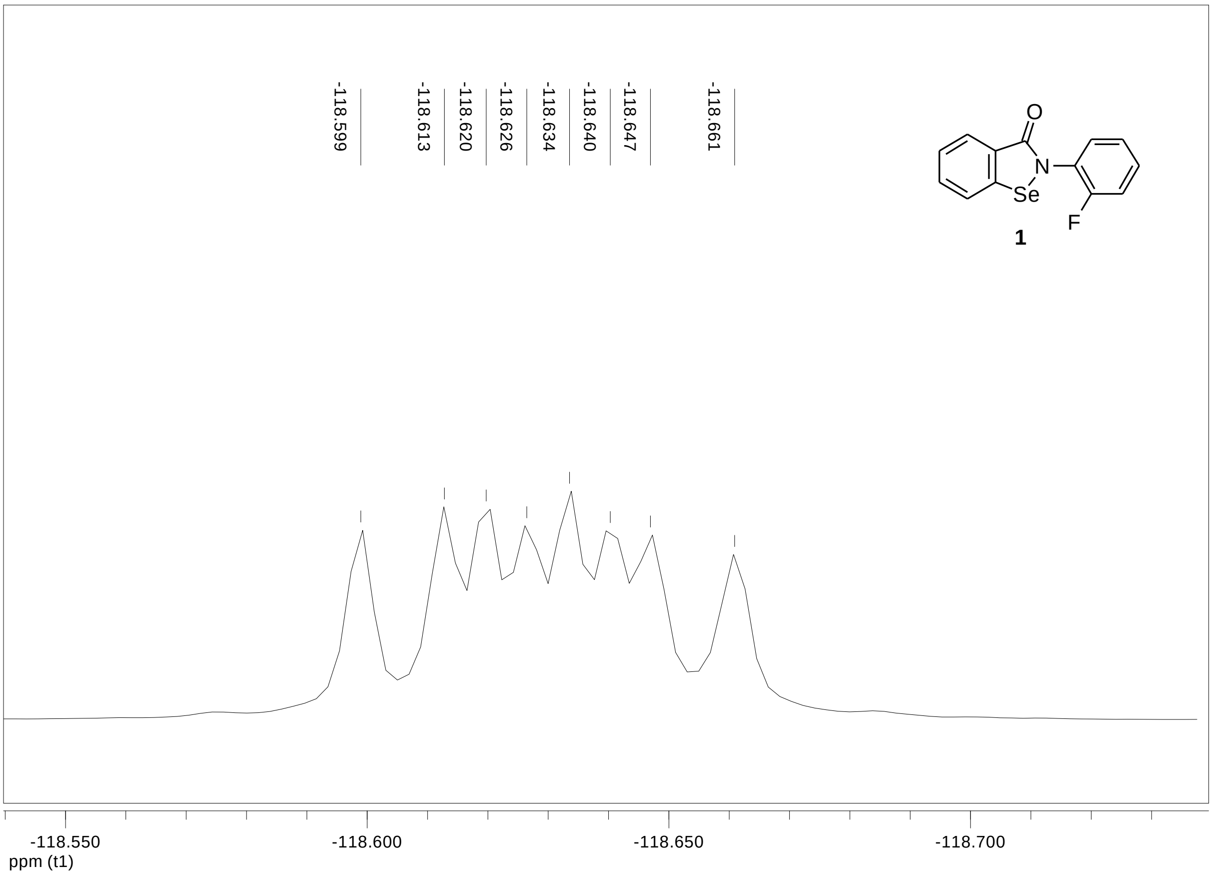

Fig. S13. ^{19}F-NMR (376.17 MHz, DMSO-d_6) spectrum of compound **1**

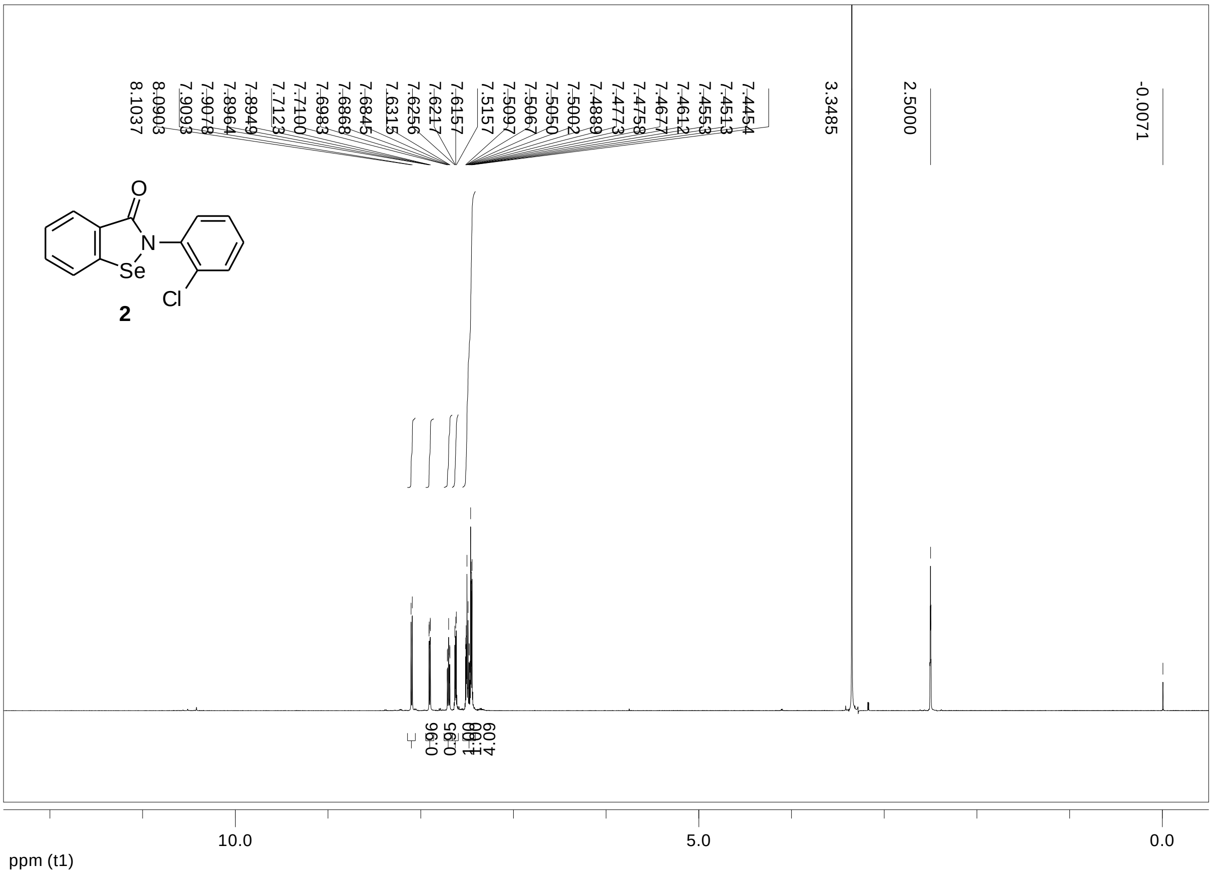

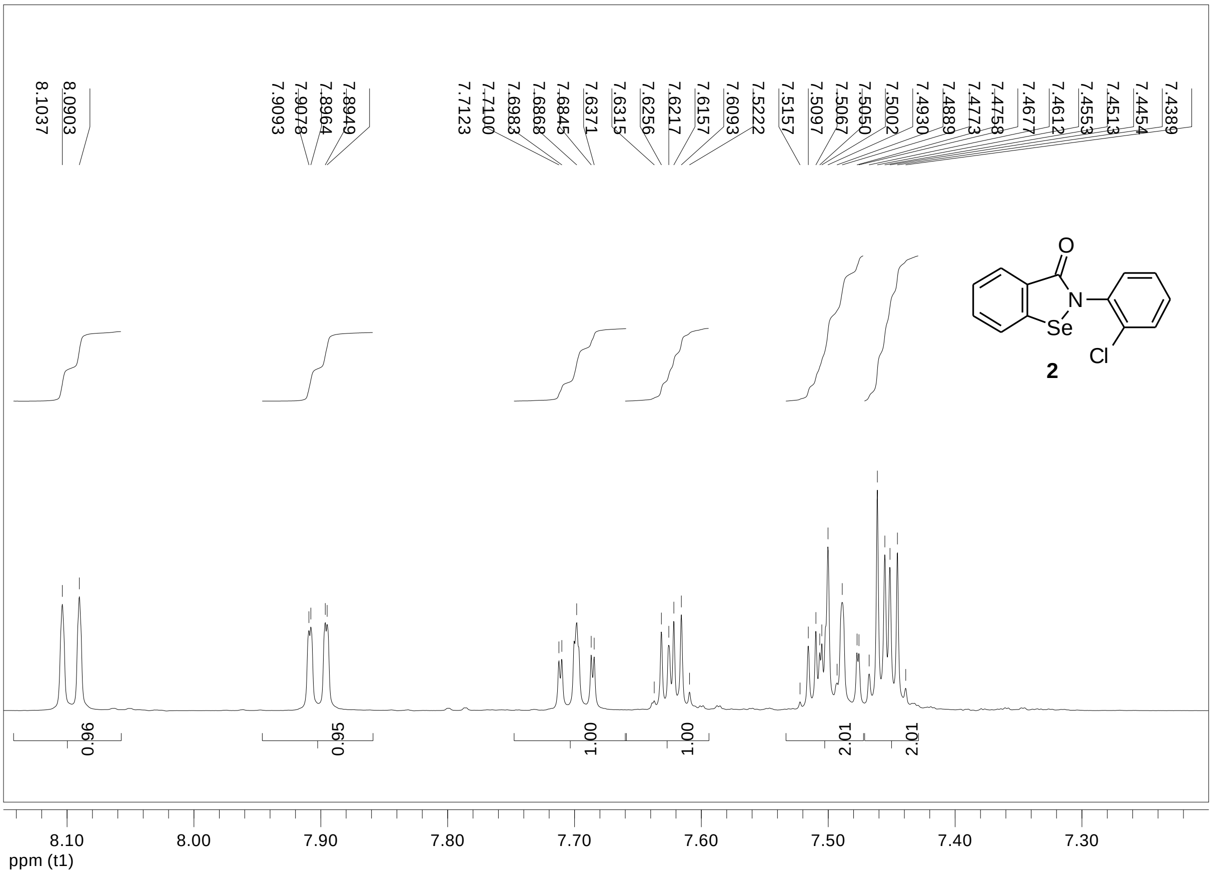

Fig. S14. ^{1}H-NMR (600.58 MHz, DMSO-d_6) spectrum of compound **2**

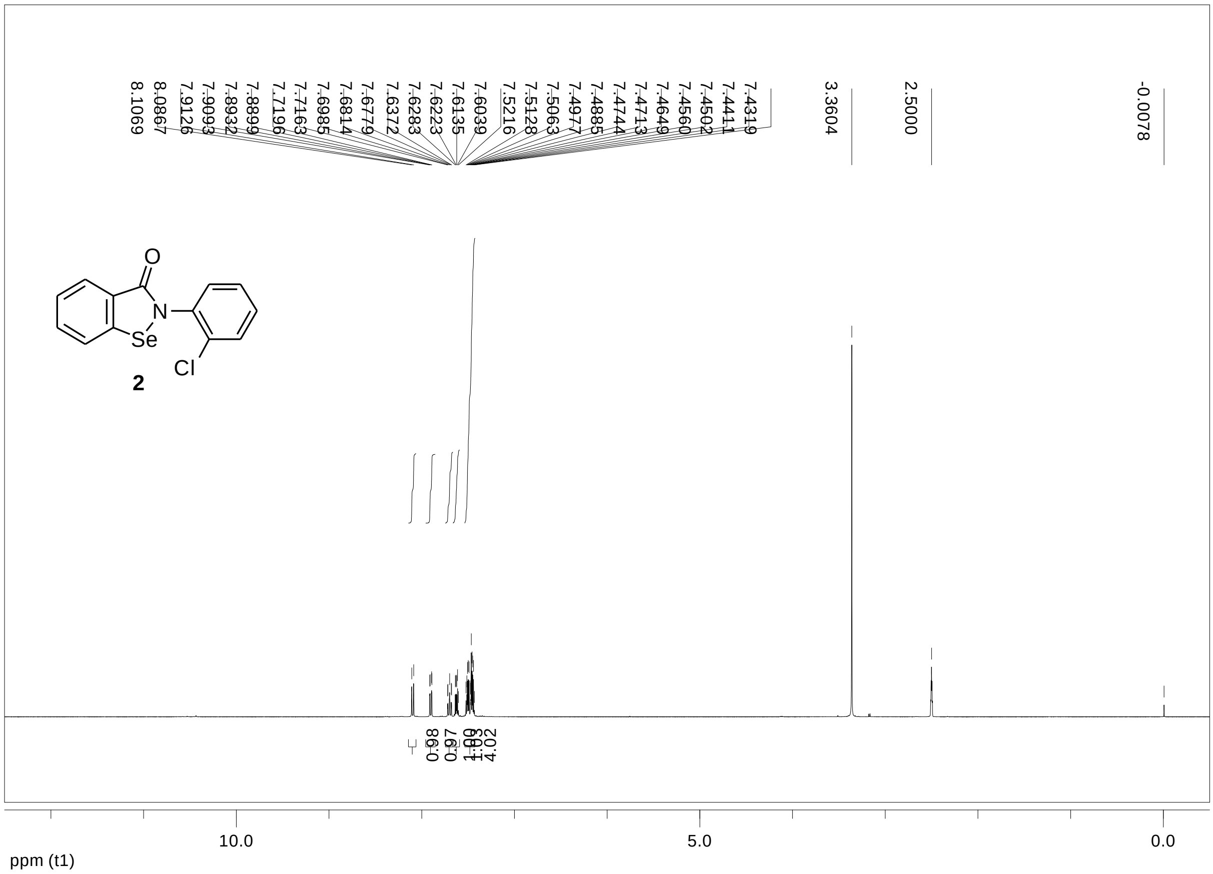

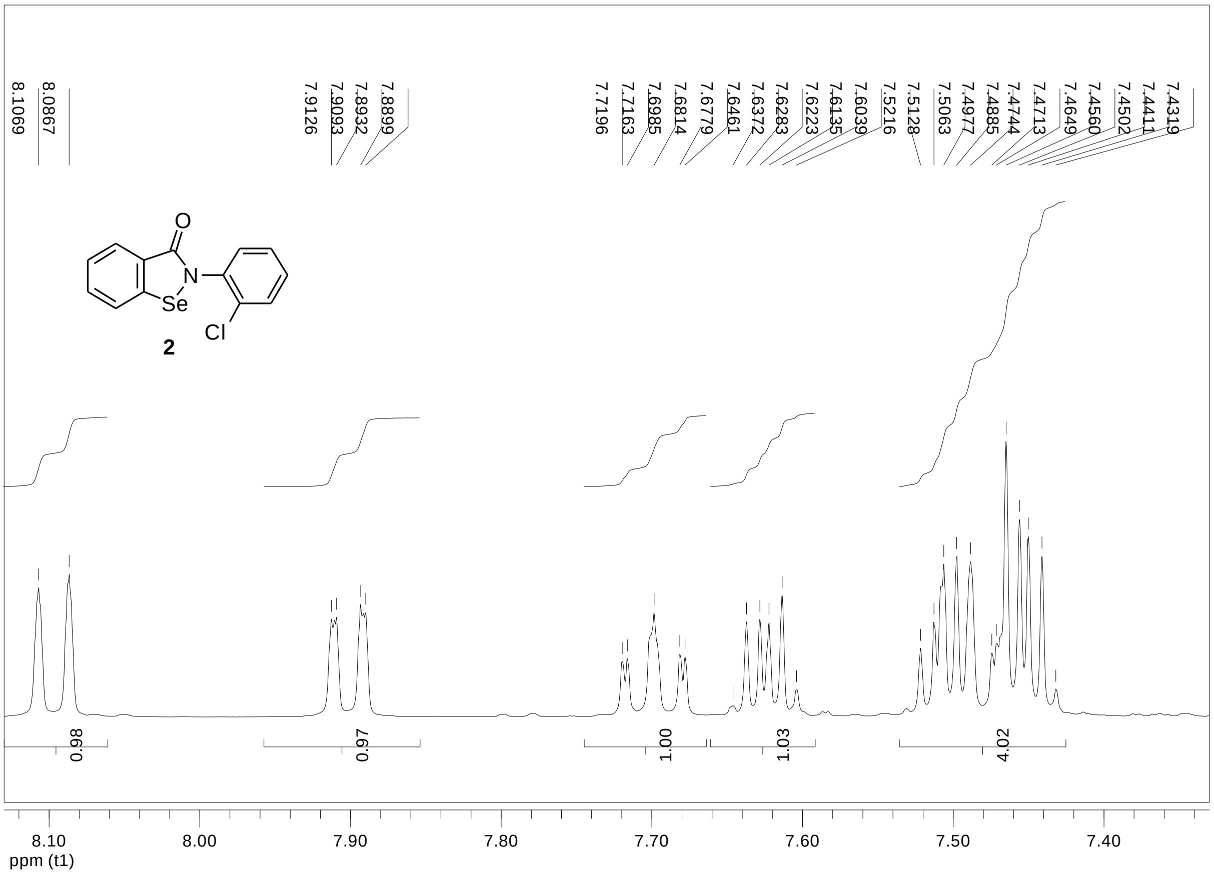

Fig. S15. ^{1}H-NMR (399.78 MHz, DMSO-d_6) spectrum of compound **2**

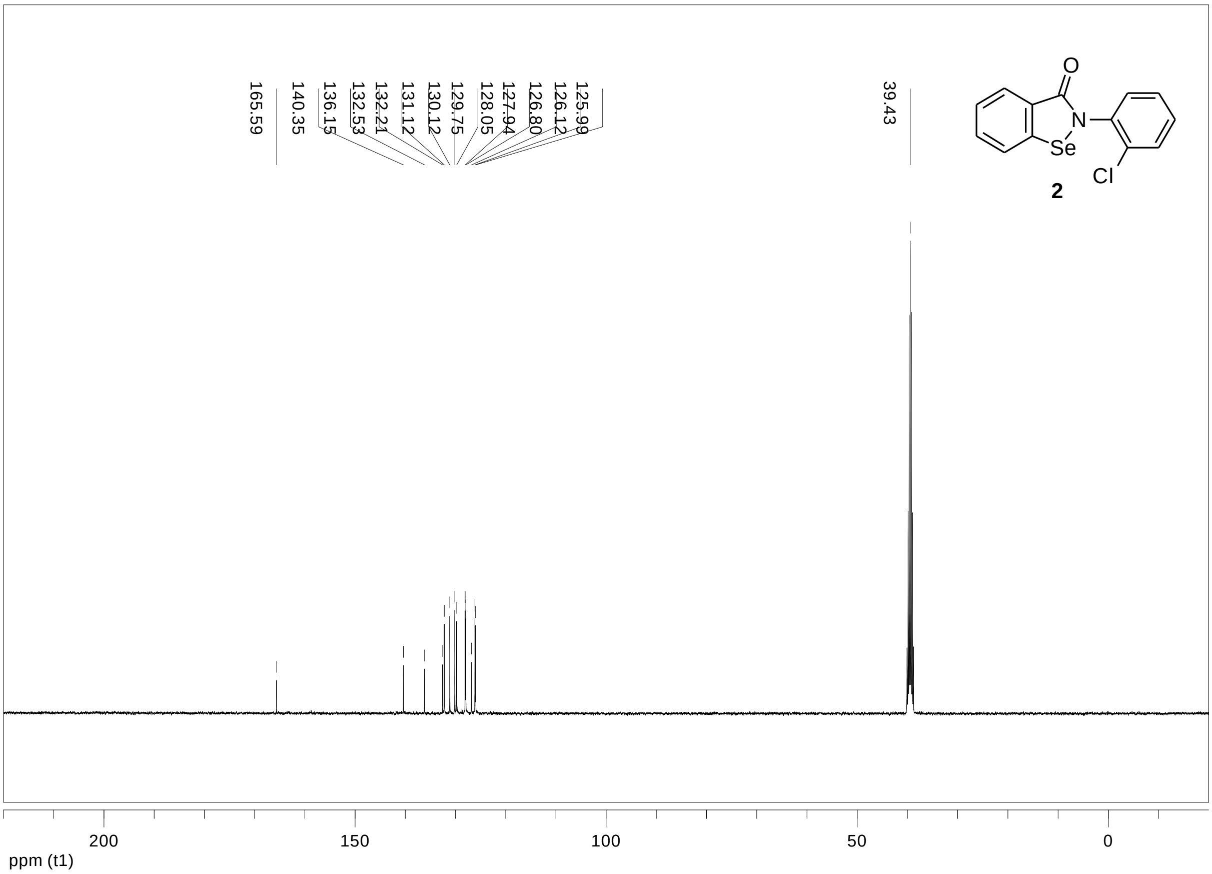

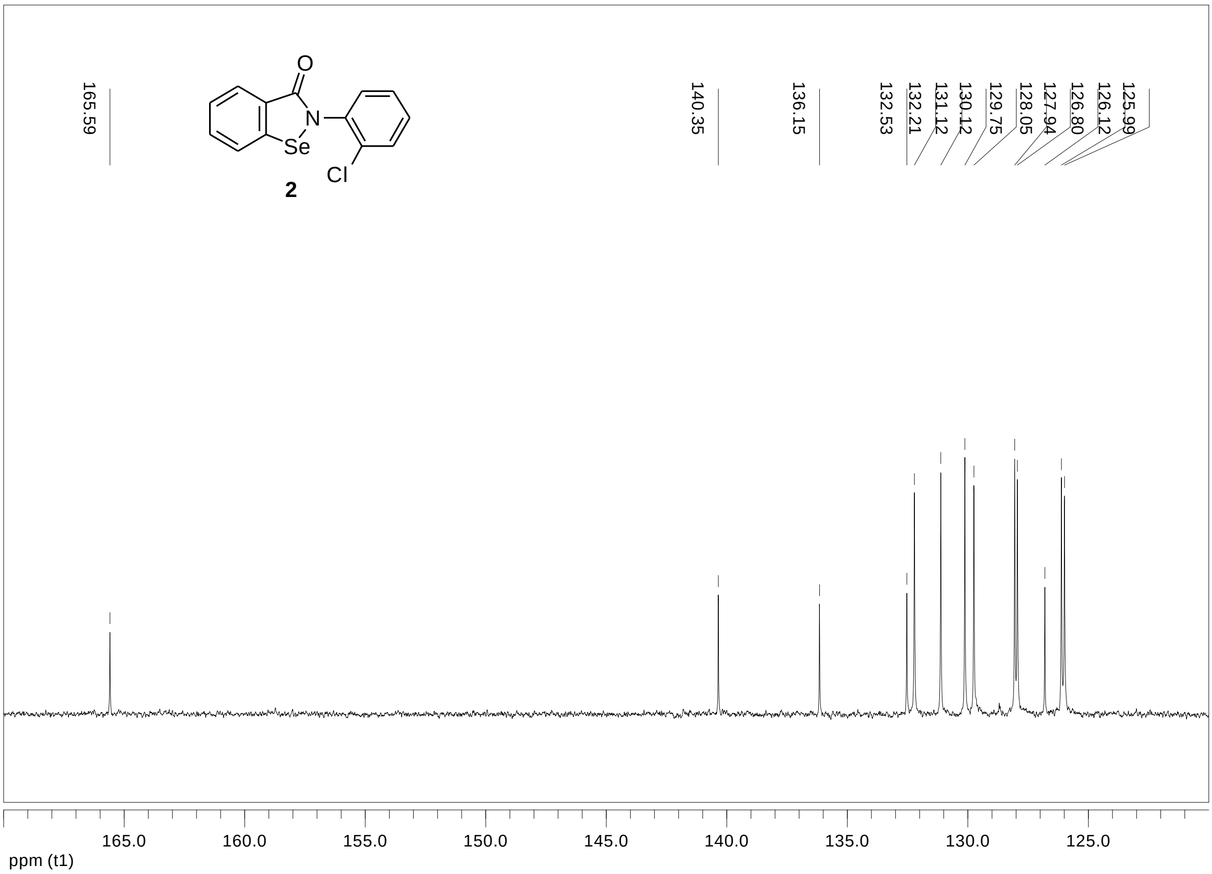

Fig. S16. ^{13}C-NMR (100.52 MHz, DMSO-d_6) spectrum of compound **2**

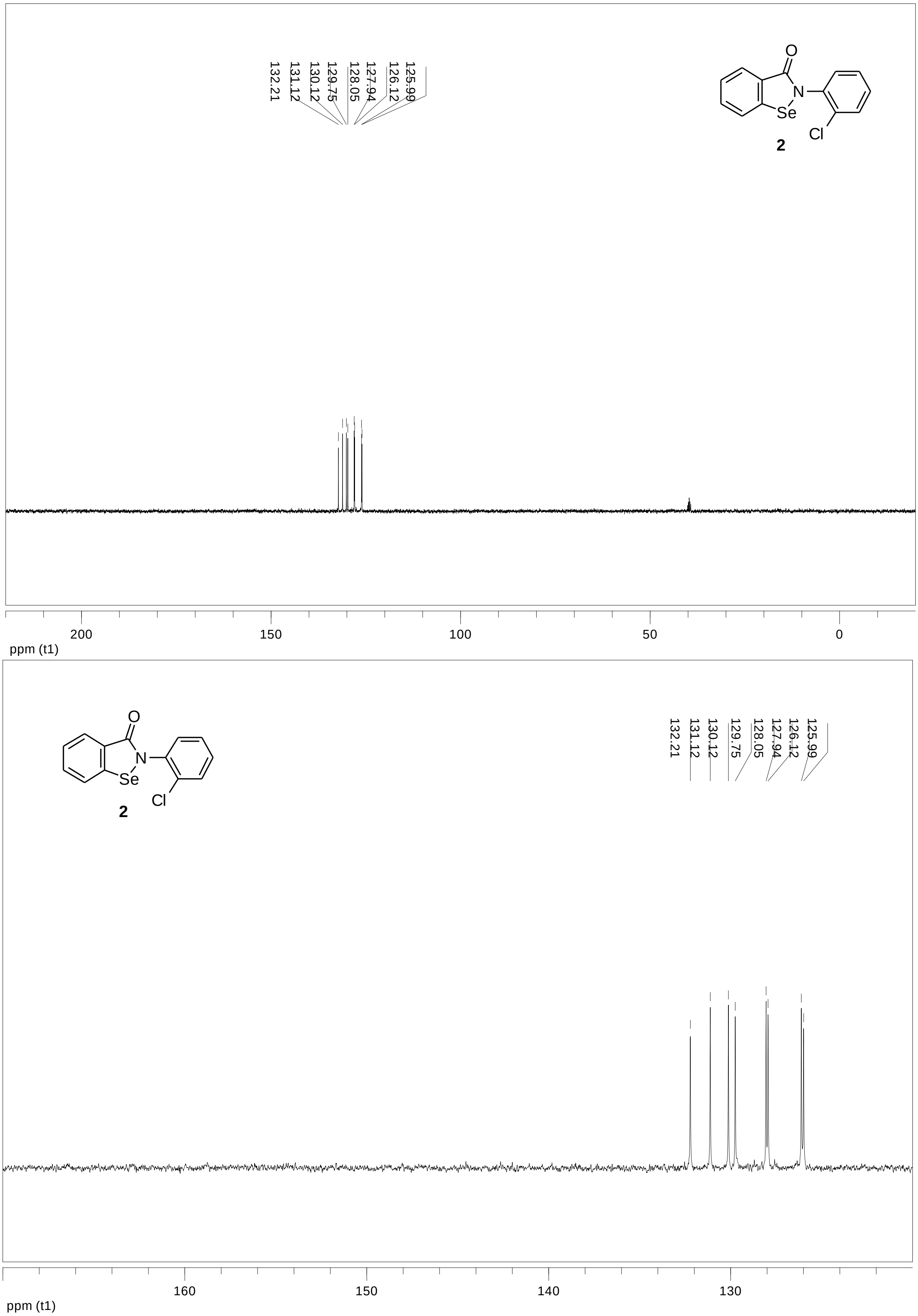

Fig. S17.^{13}C-NMR (100.52 MHz, DMSO-d_6) dept-135 experiment of compound **2**

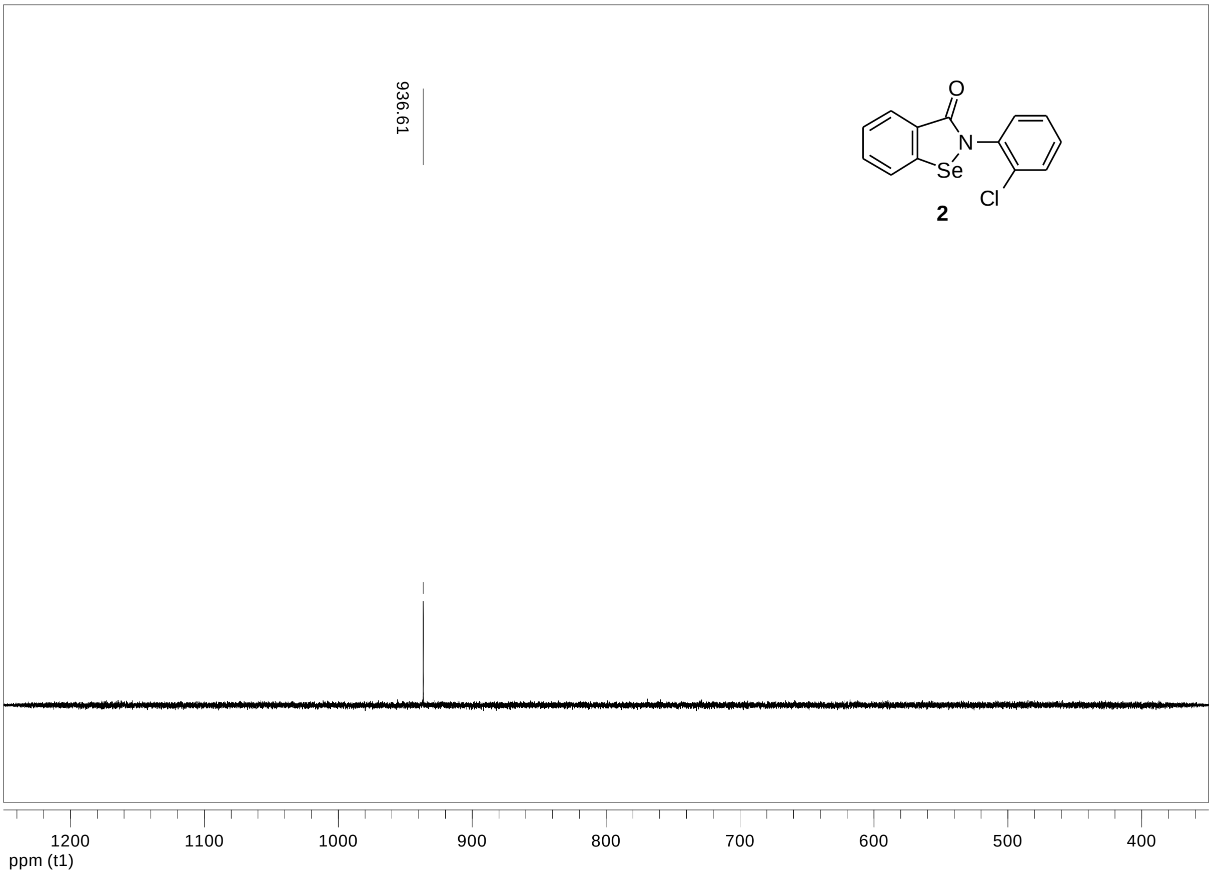

Fig. S18. ^{77}Se-NMR (76.24 MHz, DMSO-d_6) spectrum of compound **2**

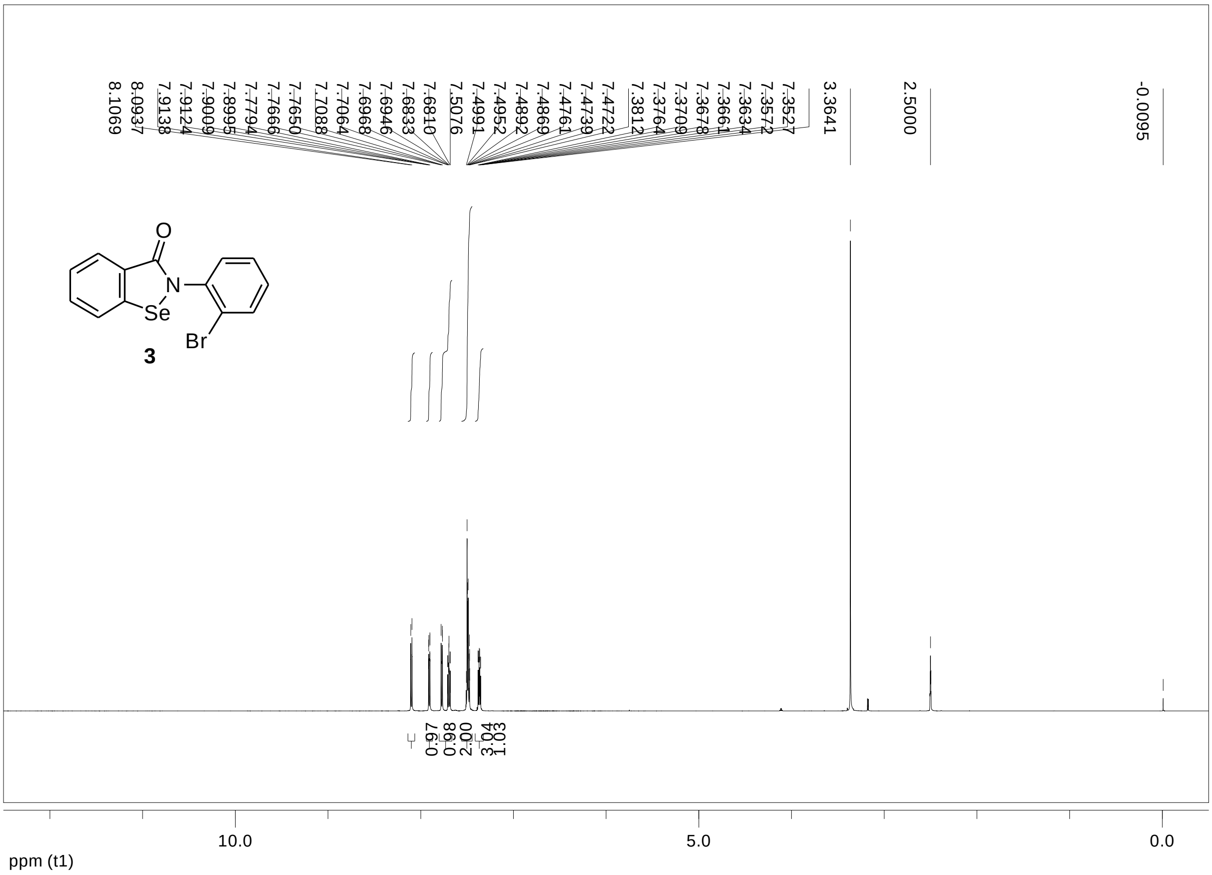

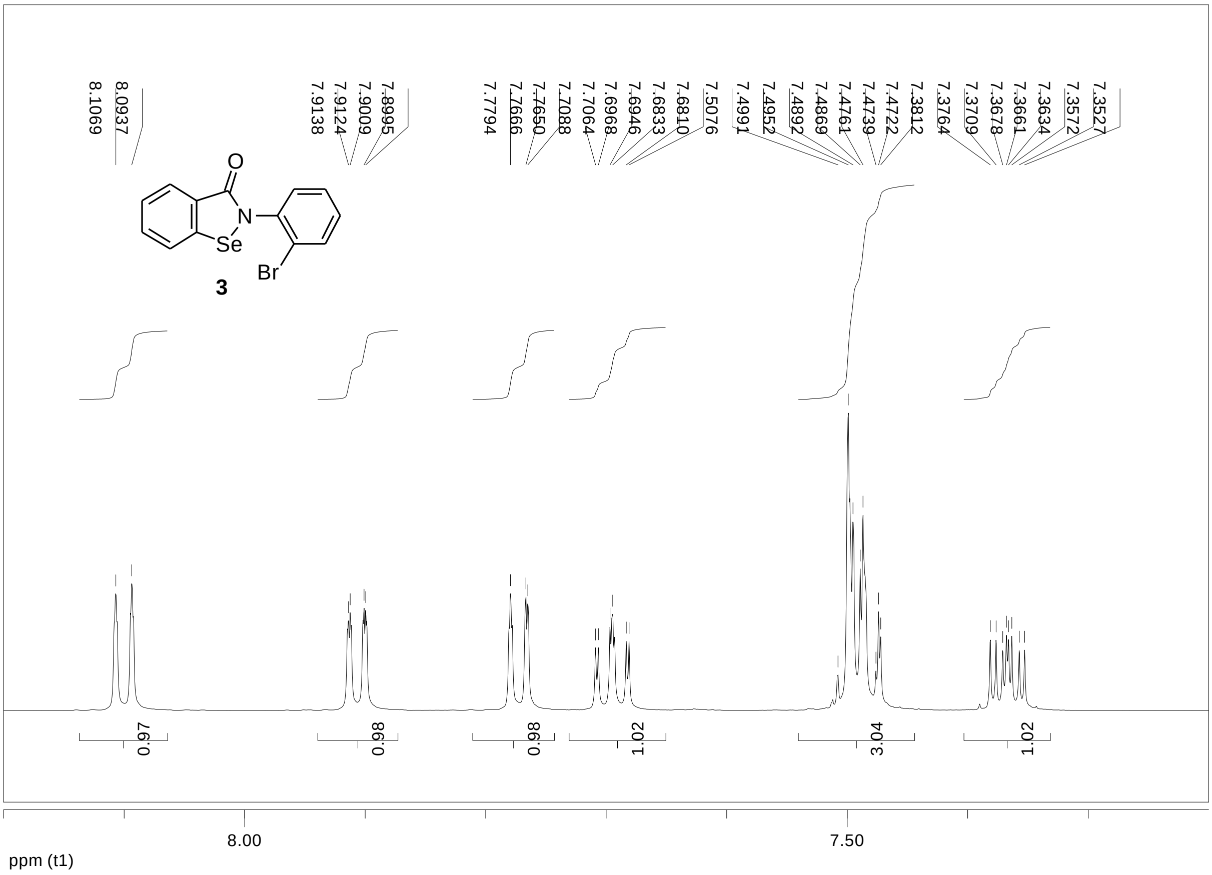

Fig. S19. ^{1}H-NMR (600.58 MHz, DMSO-d_6) spectrum of compound **3**

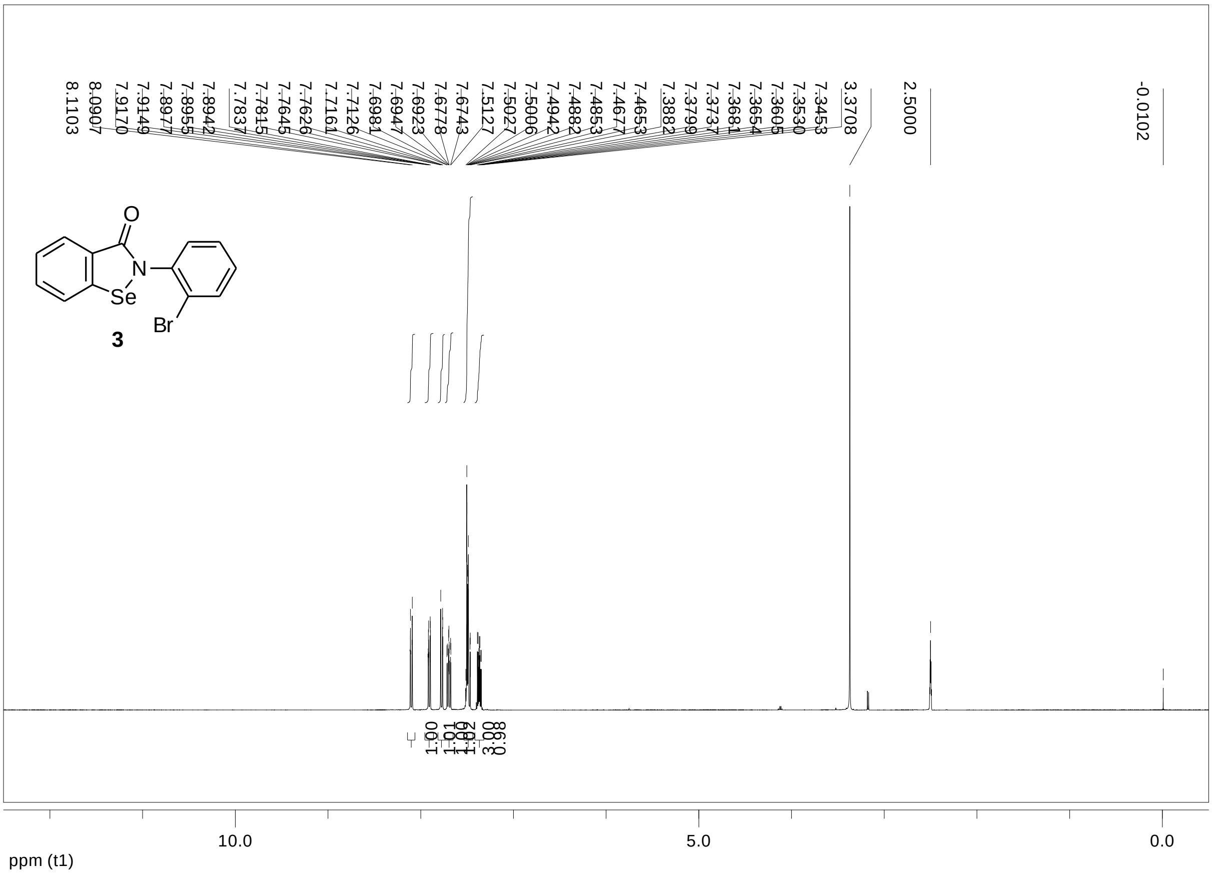

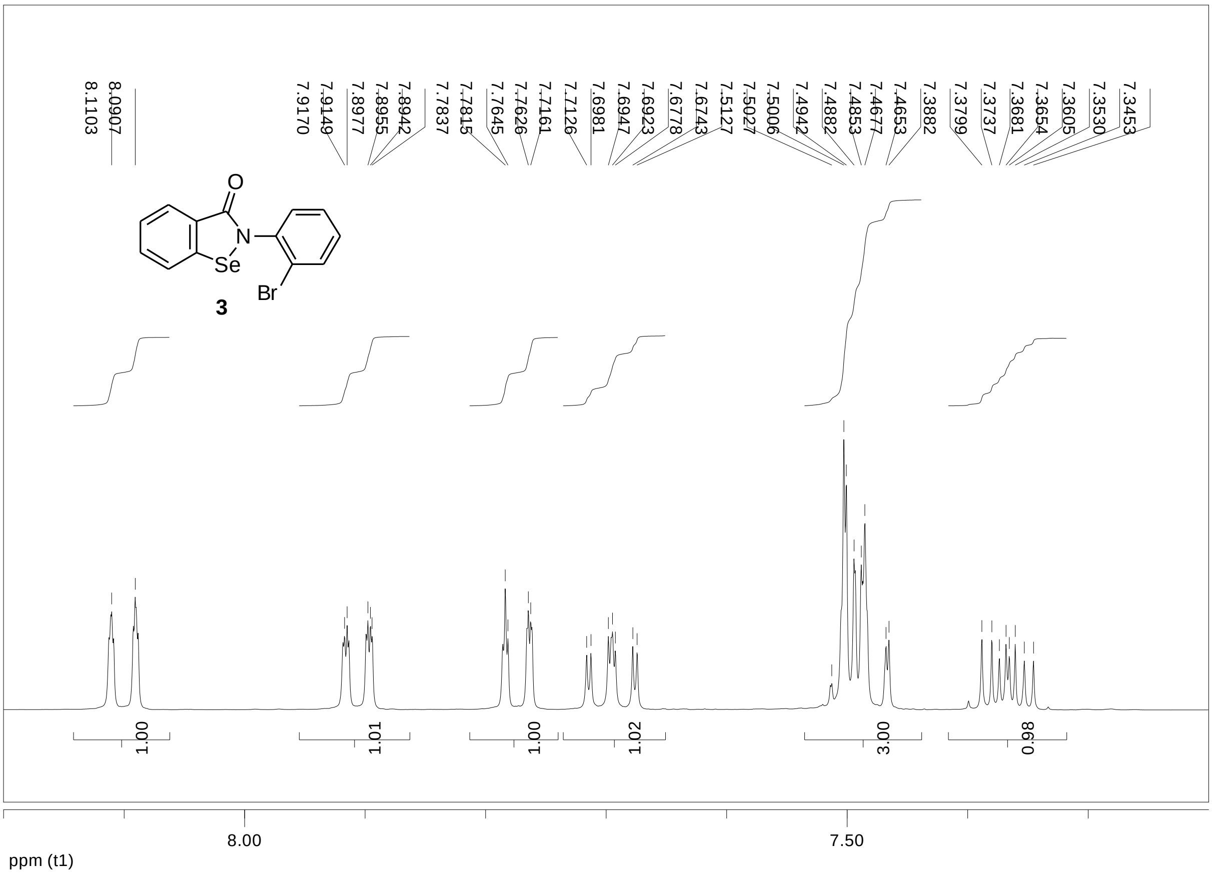

Fig. S20. ^{1}H-NMR (399.78 MHz, DMSO-d_6) spectrum of compound **3**

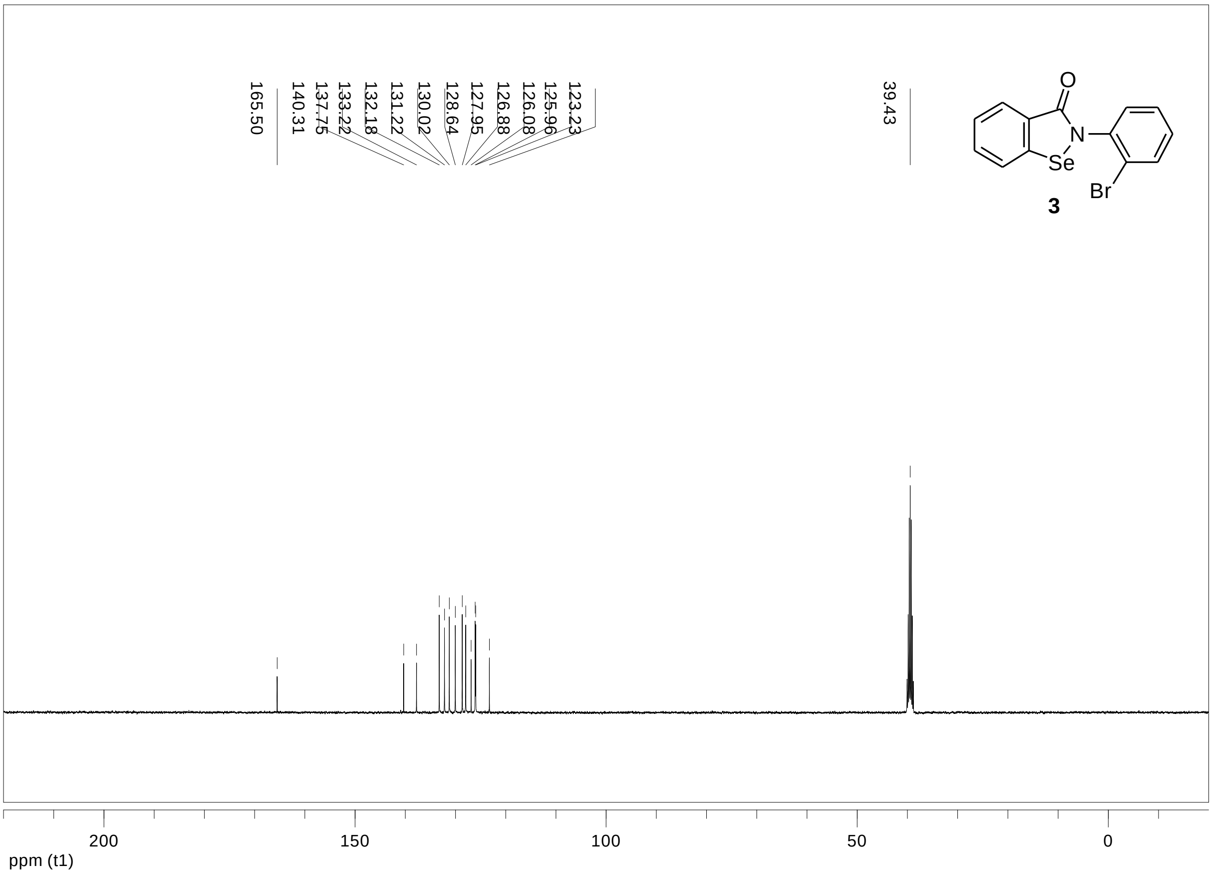

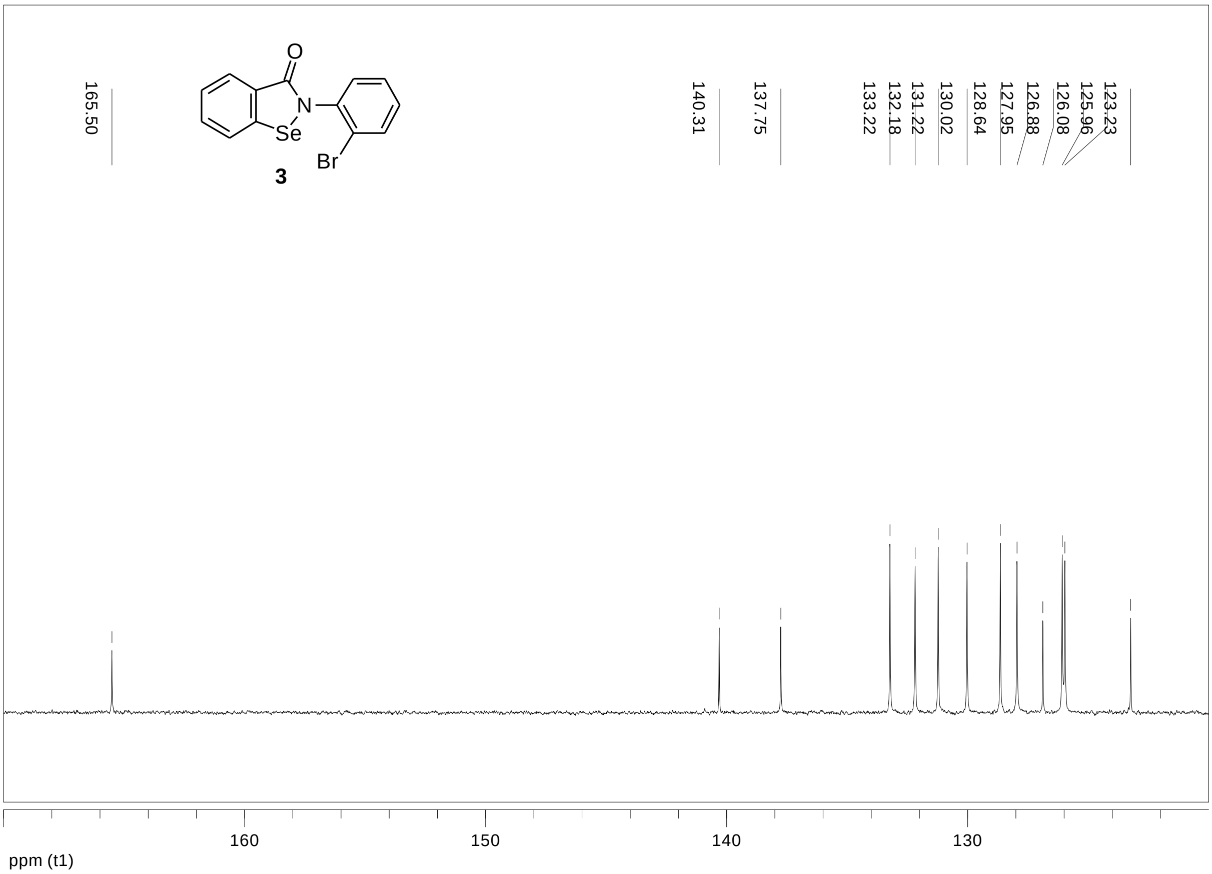

Fig. S21. ^{13}C-NMR (100.52 MHz, DMSO-d_6) spectrum of compound **3**

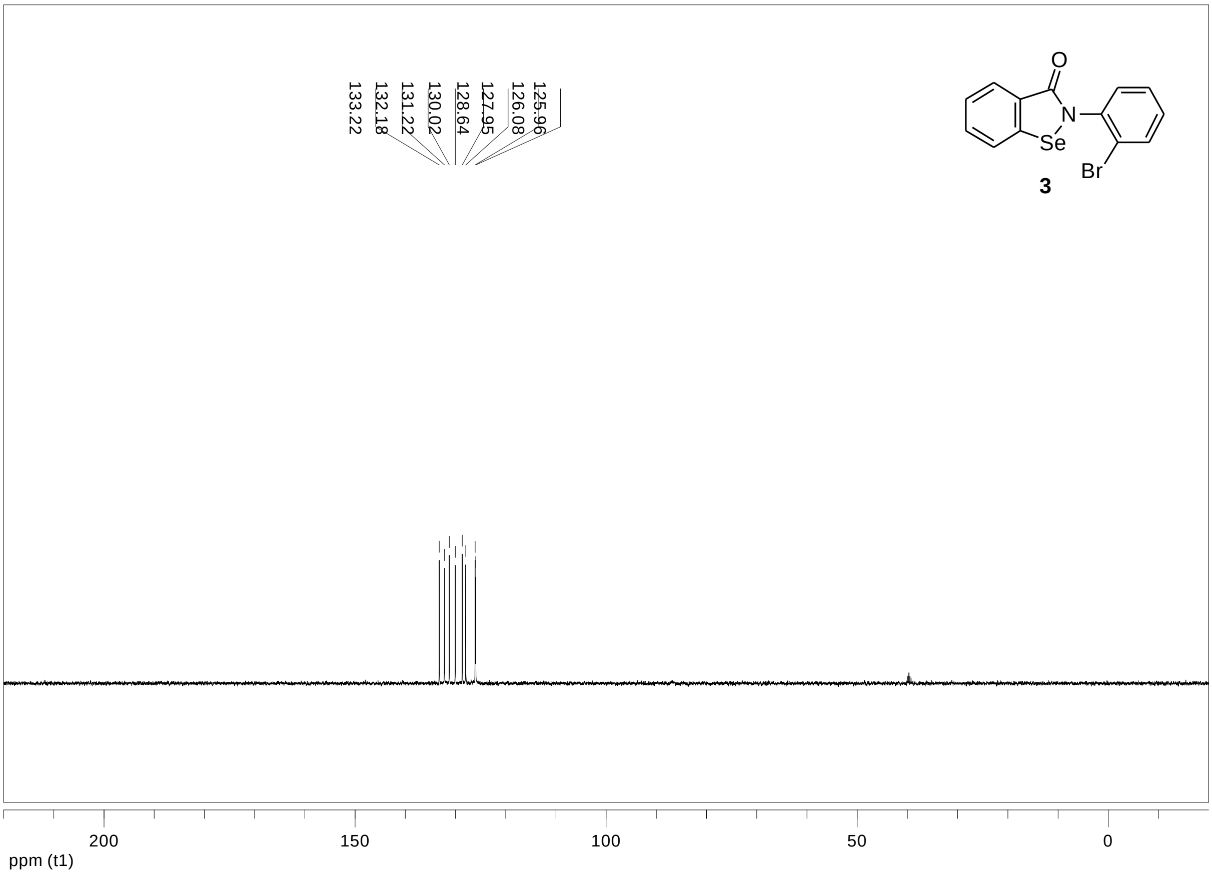

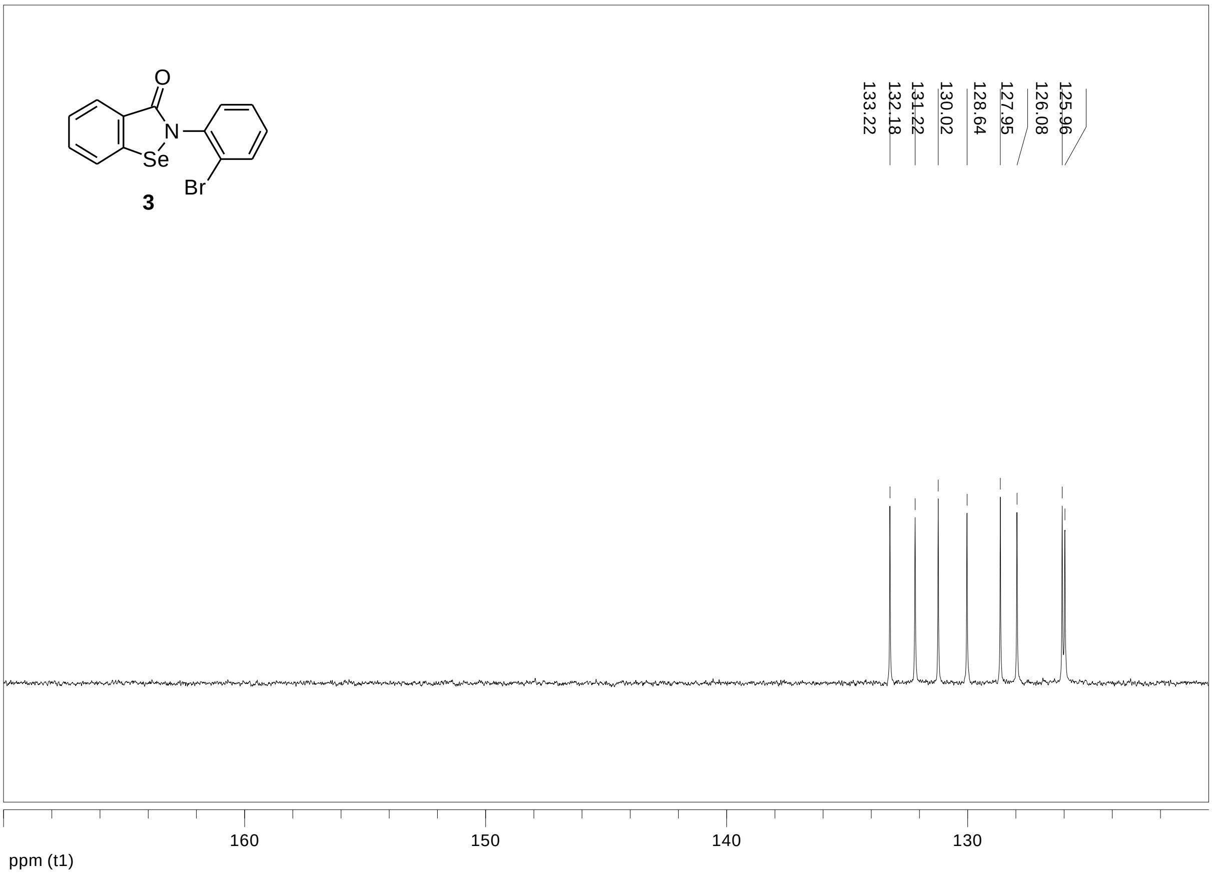

Fig. S22. ^{13}C-NMR (100.52 MHz, DMSO-d_6) dept-135 experiment of compound **3**

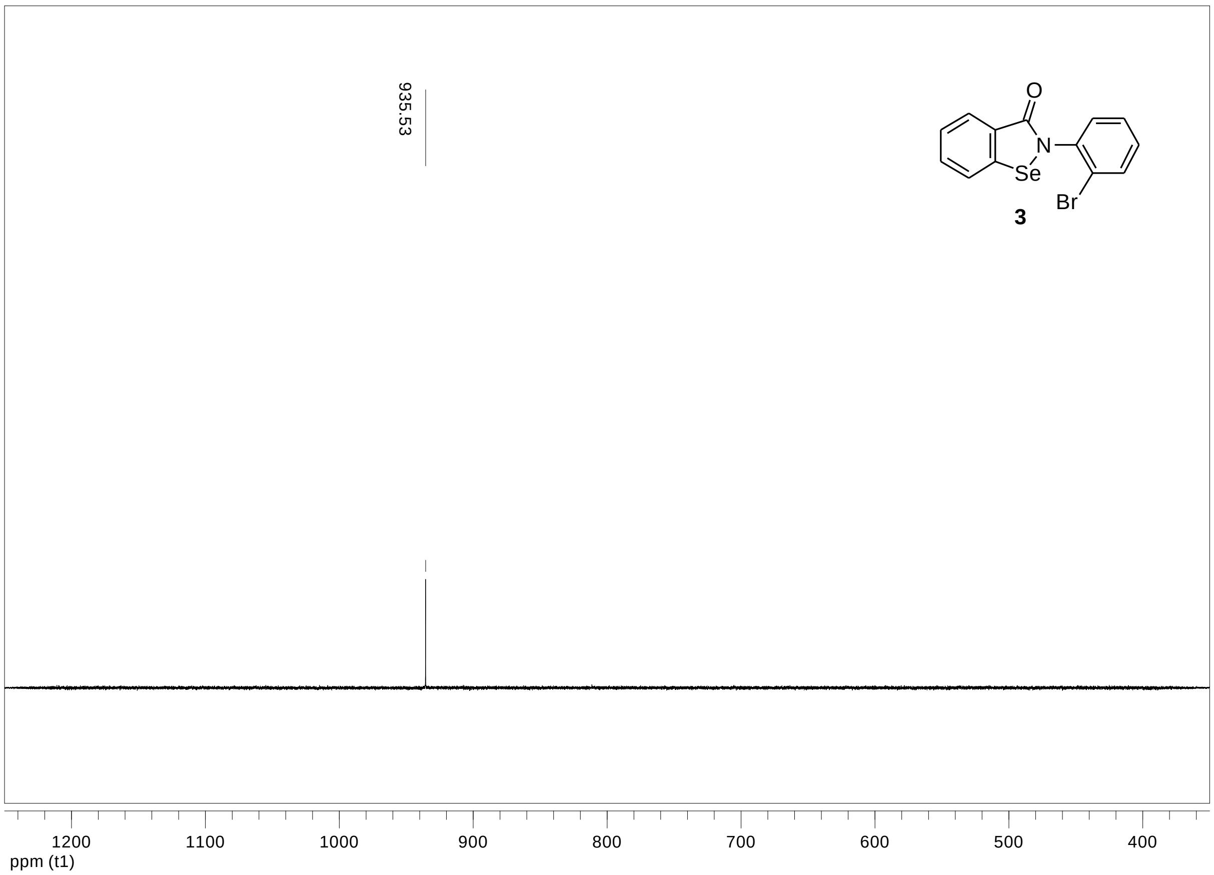

Fig. S23. ^{77}Se-NMR (76.24 MHz, DMSO-d_6) spectrum of compound **3**

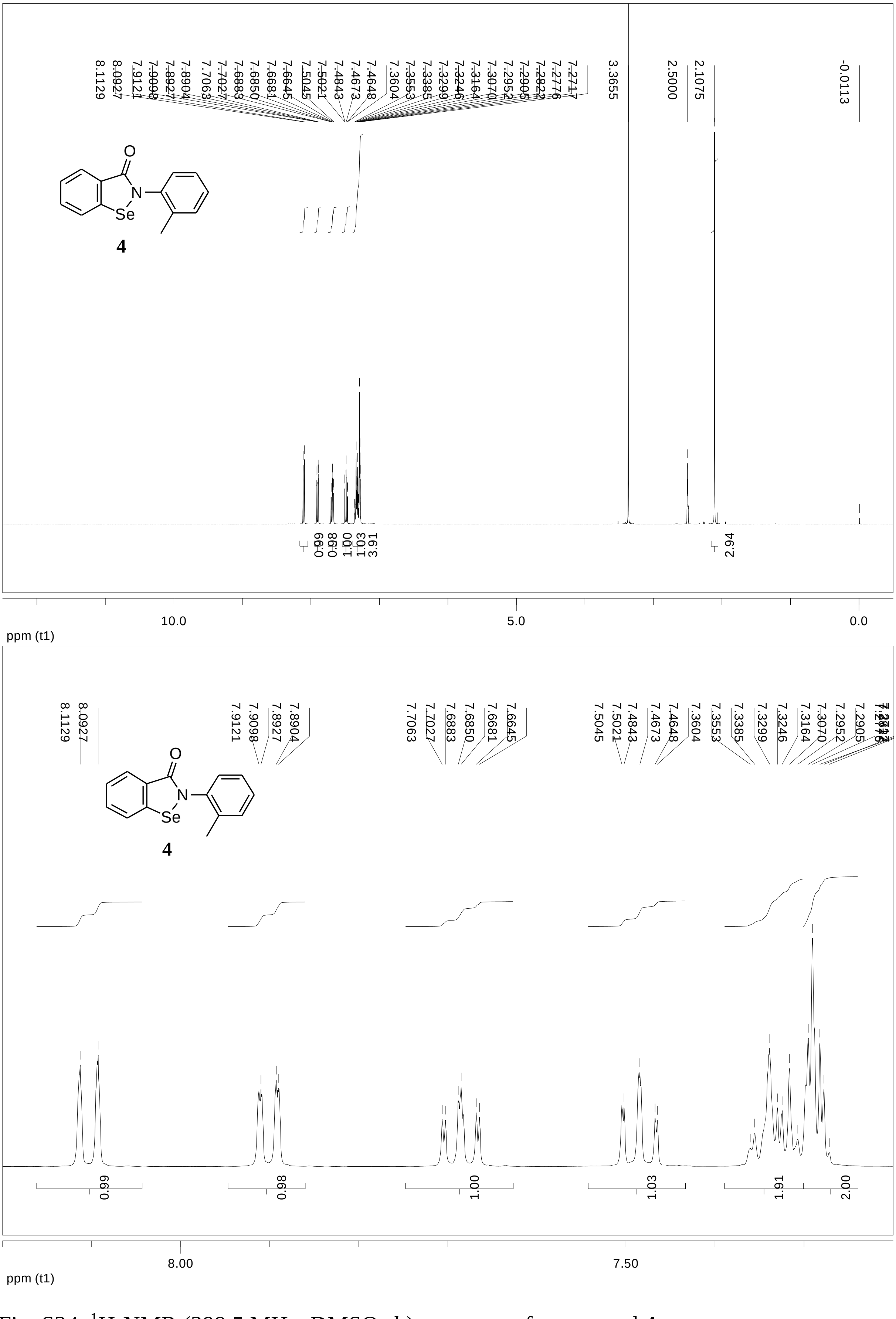

Fig. S24. ^{1}H-NMR (399.5 MHz, DMSO-d_6) spectrum of compound **4**

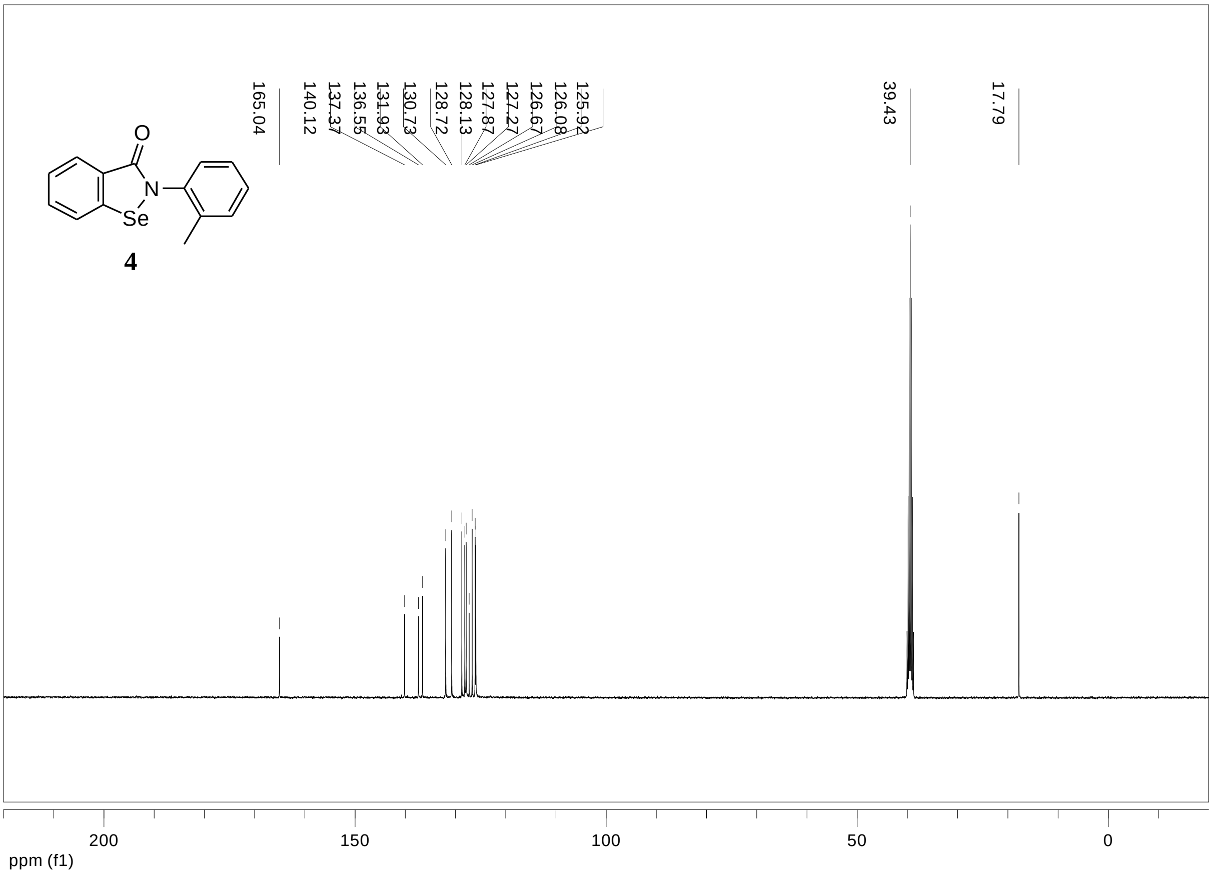

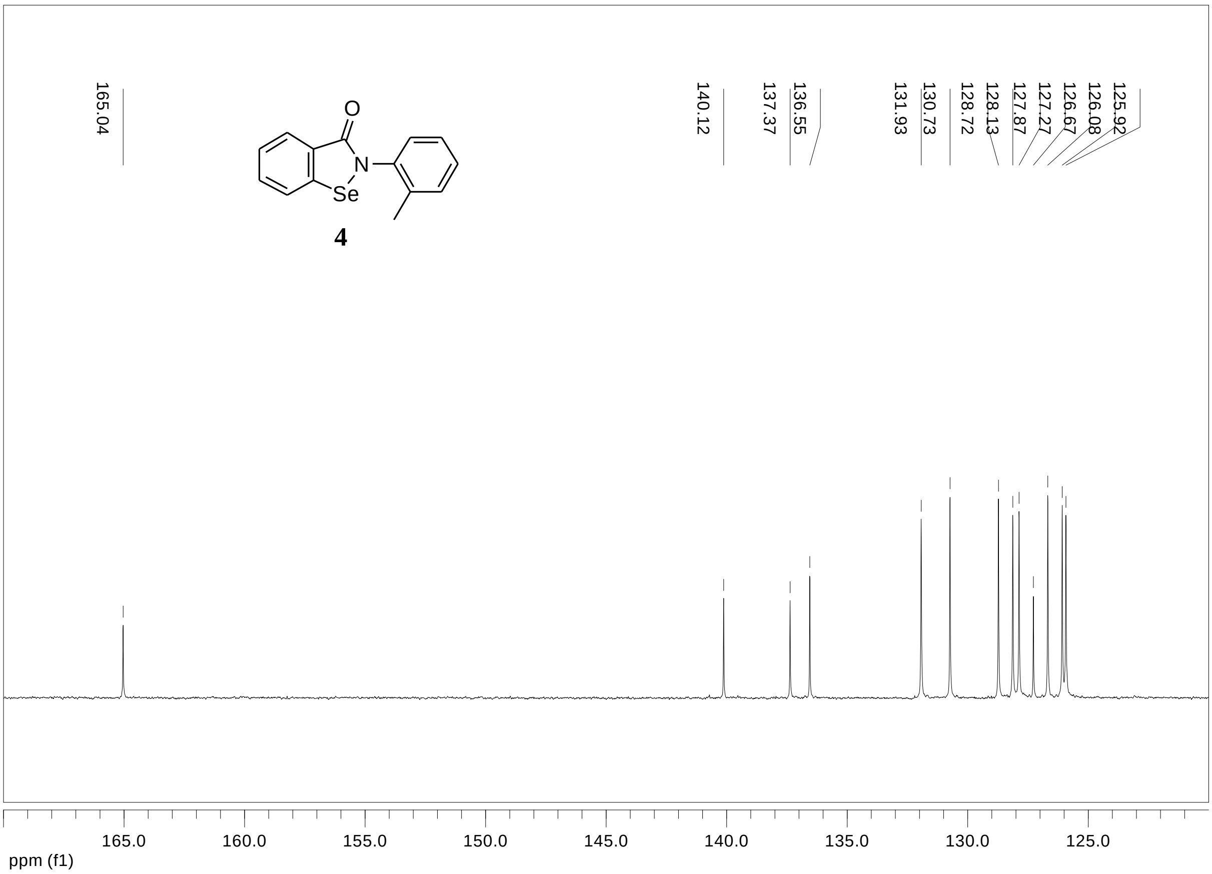

Fig. S25. ^{13}C-NMR (100.52 MHz, DMSO-d_6) spectrum of compound **4**

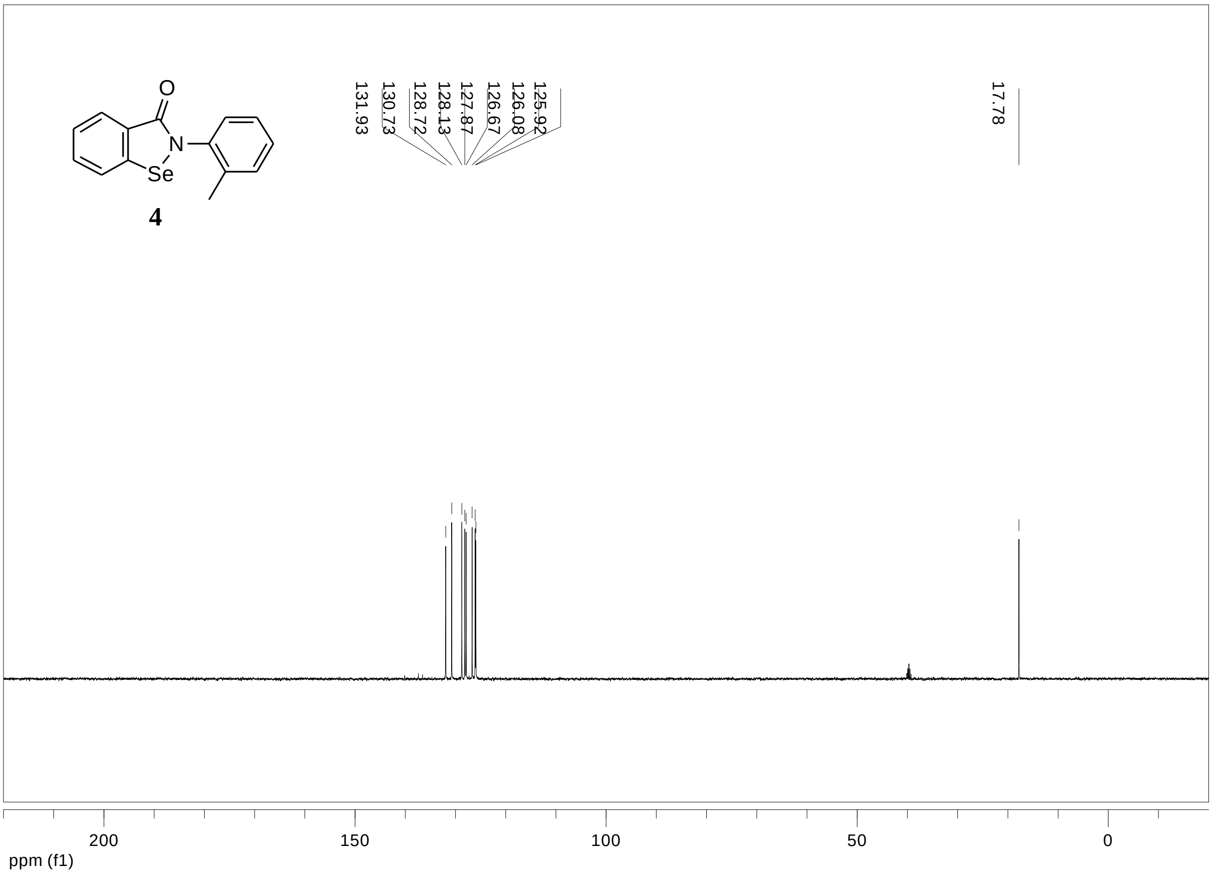

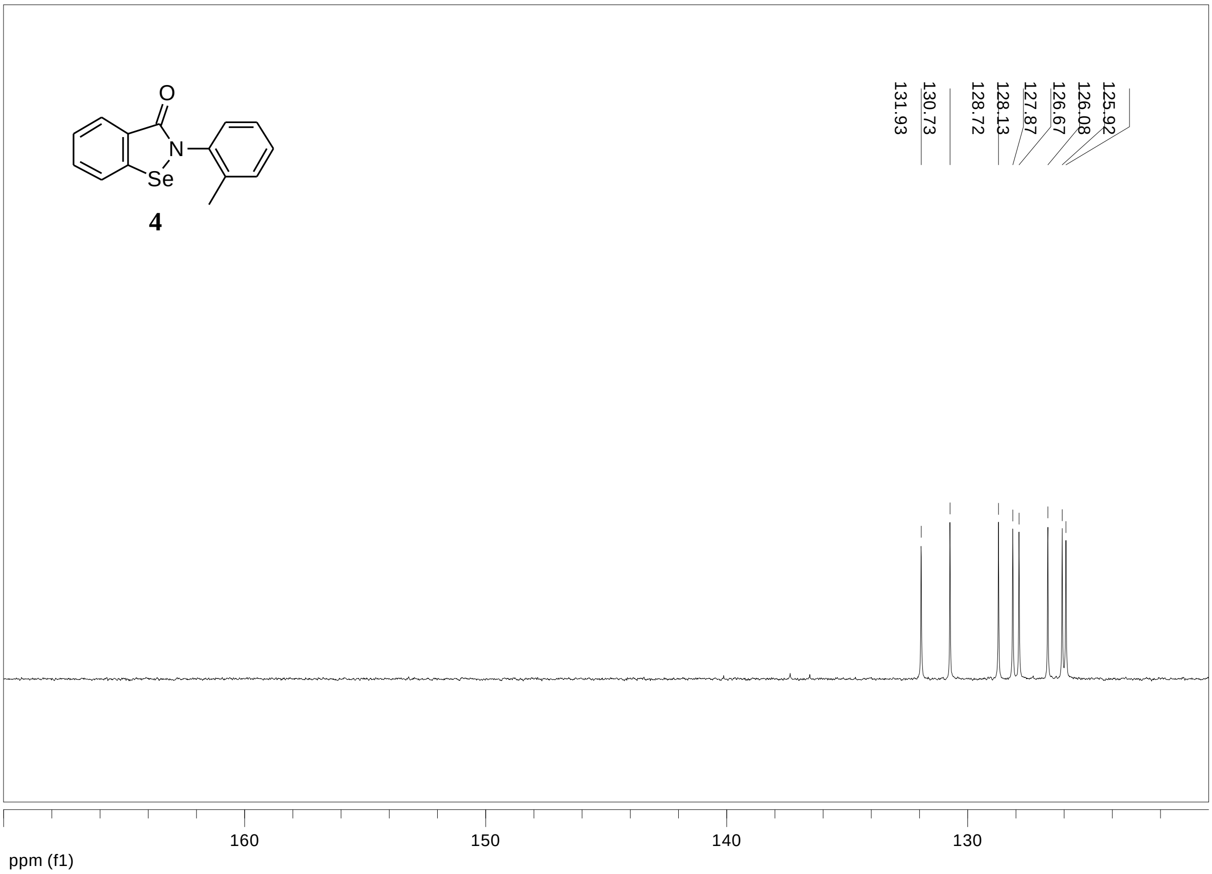

Fig. S26. ^{13}C-NMR (100.52 MHz, DMSO-d_6) dept-135 experiment of compound **4**

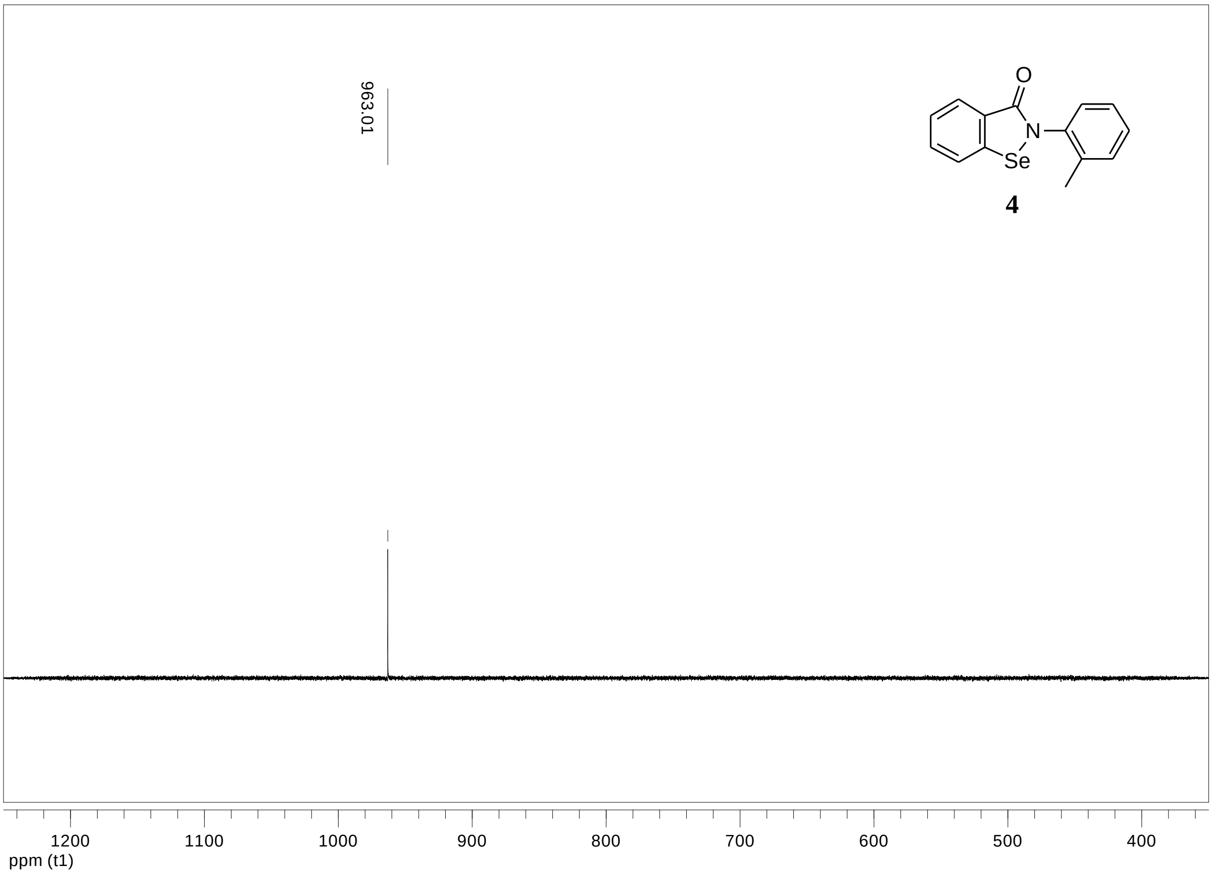

Fig. S27. ^{77}Se-NMR (76.24 MHz, $CDCl_3$) spectrum of compound **4**

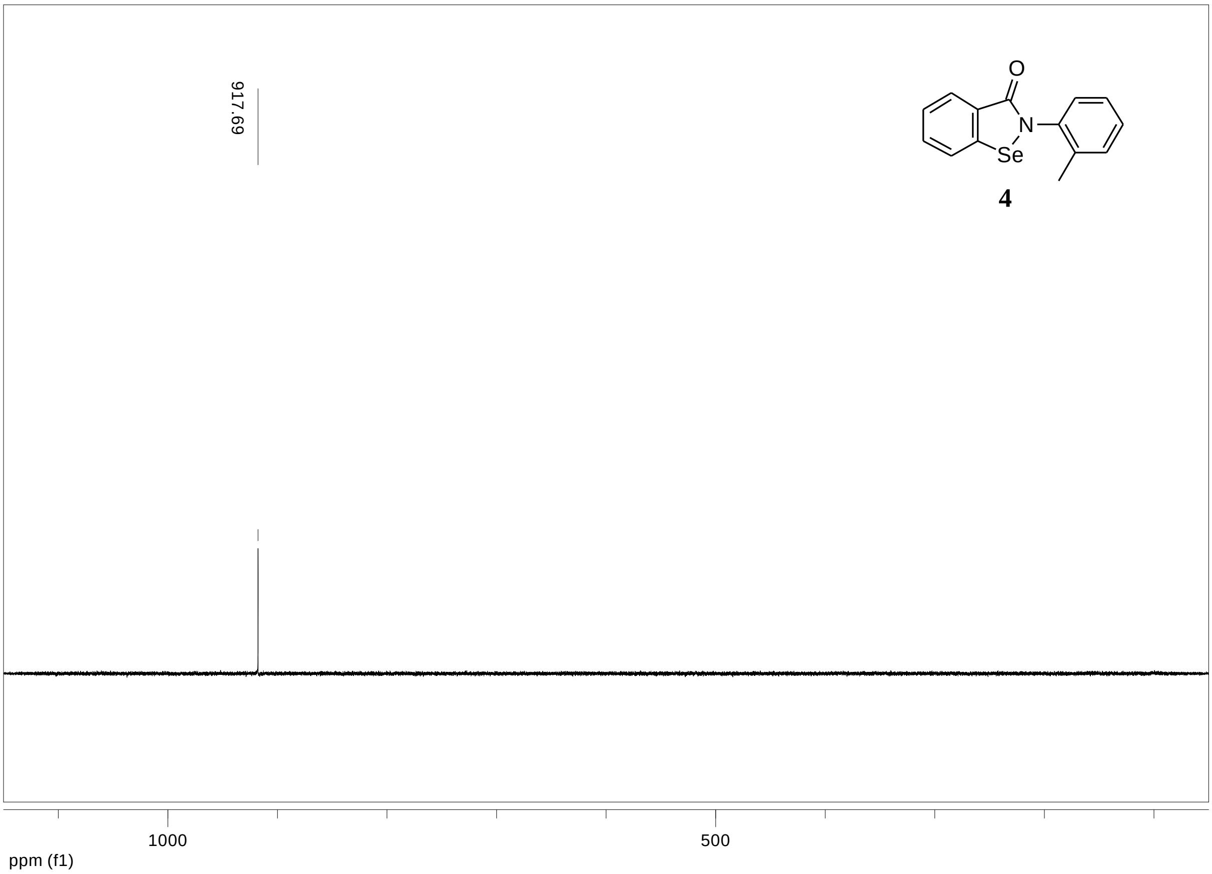

Fig. S28. ^{77}Se-NMR (76.24 MHz, DMSO-d_6) spectrum of compound **4**

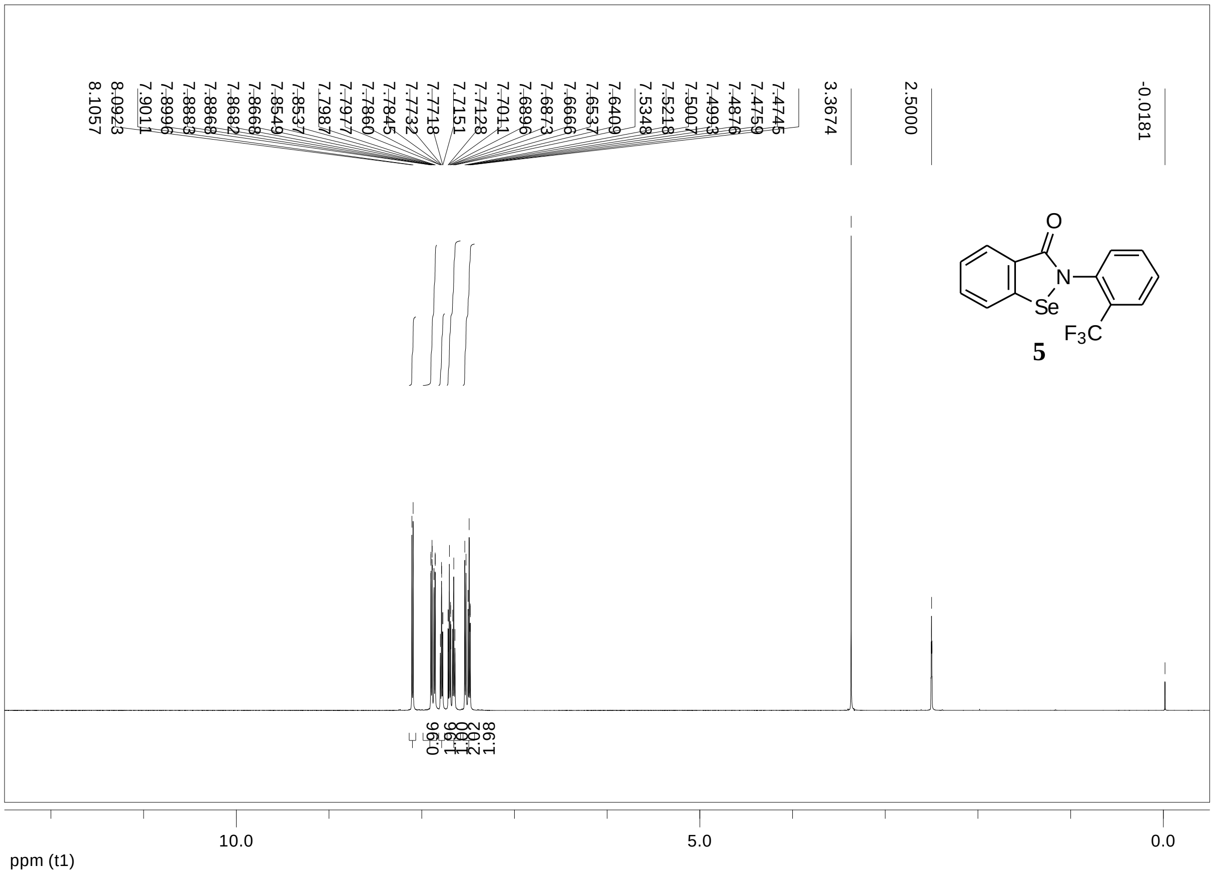

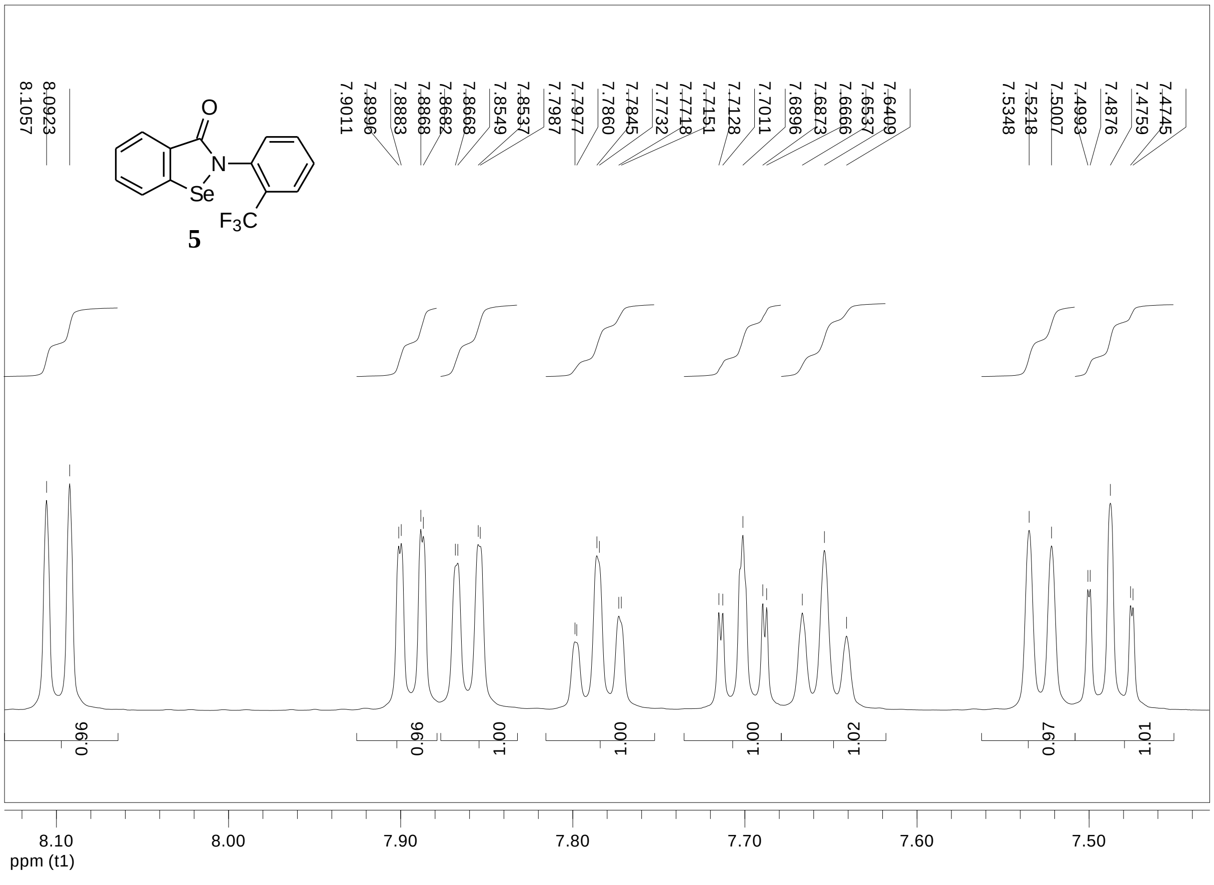

Fig. S29. ^{1}H-NMR (600.58MHz, DMSO-d_6) spectrum of compound **5**

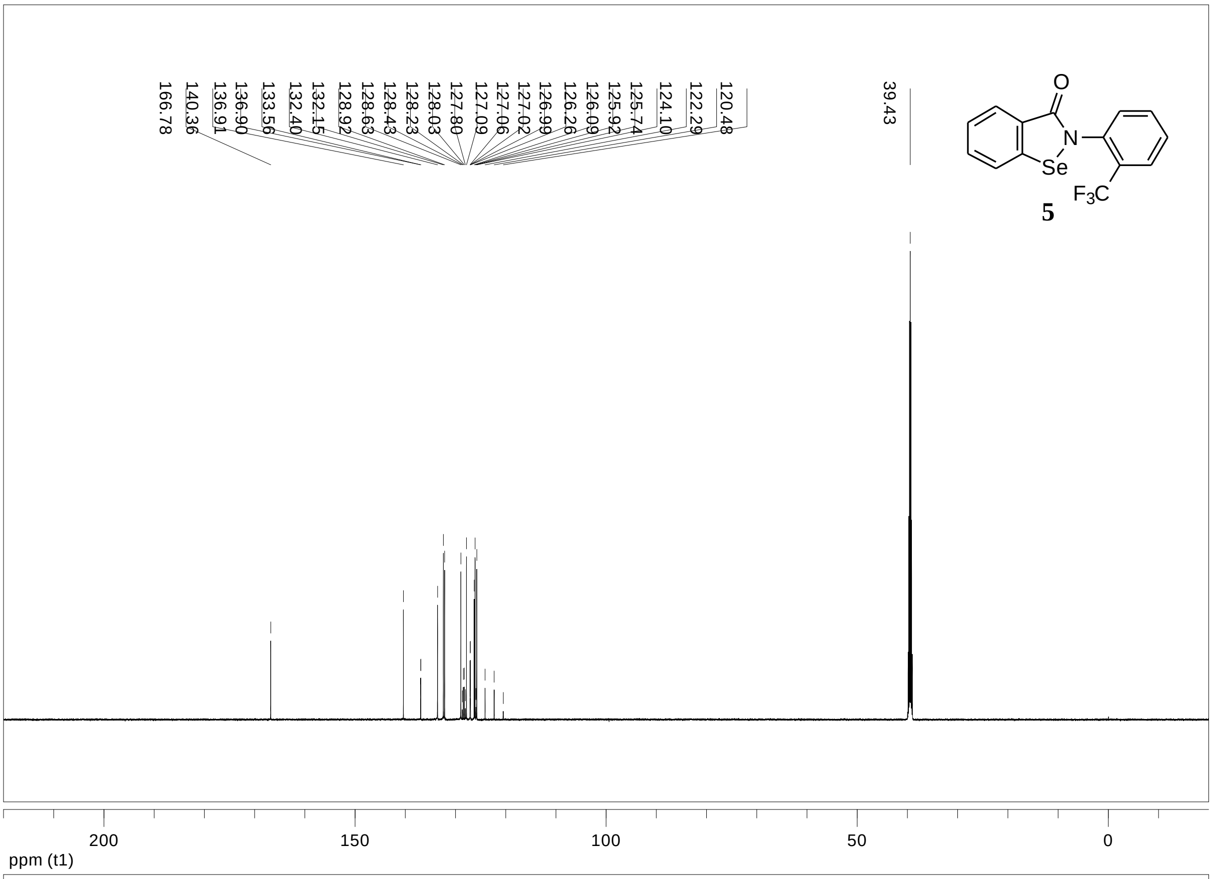

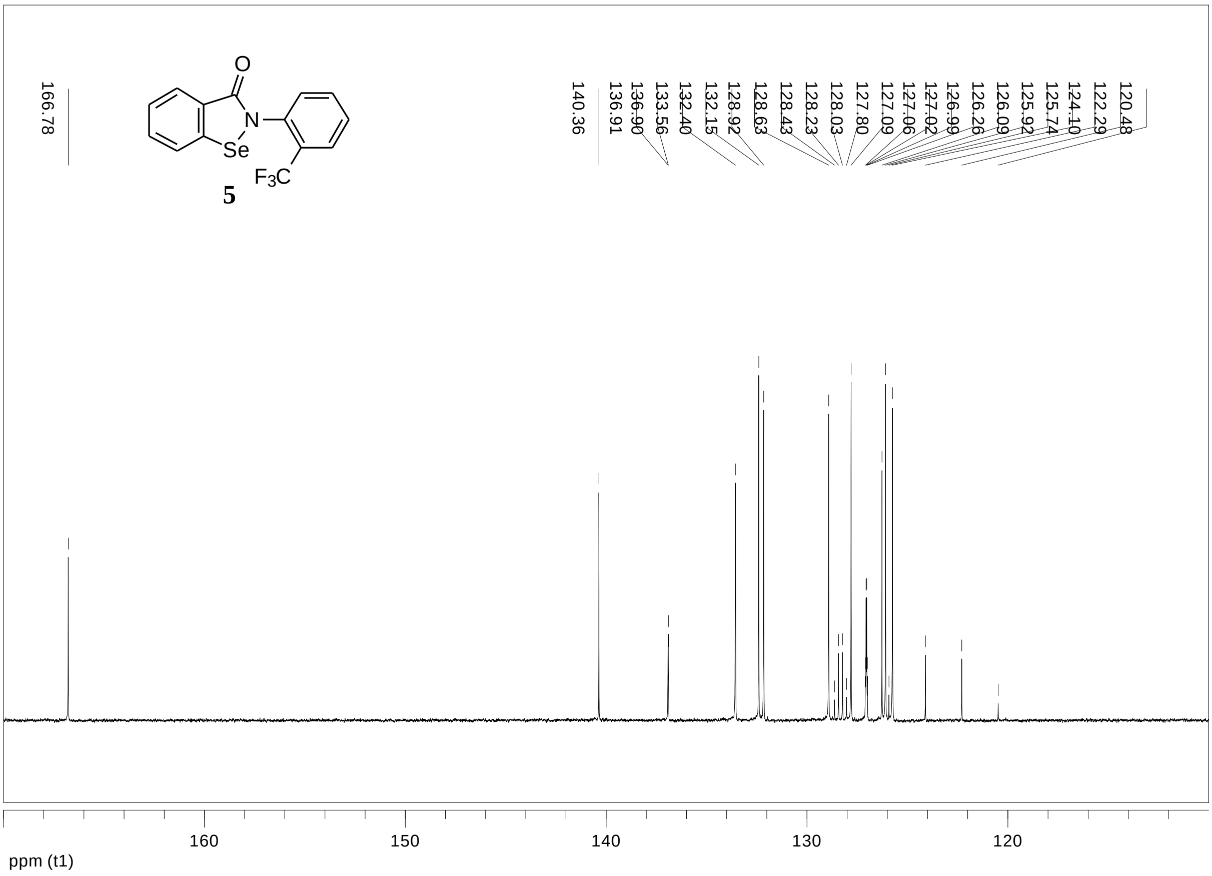

Fig. S30. ^{13}C-NMR (151.03 MHz, DMSO-d_6) spectrum of compound **5**

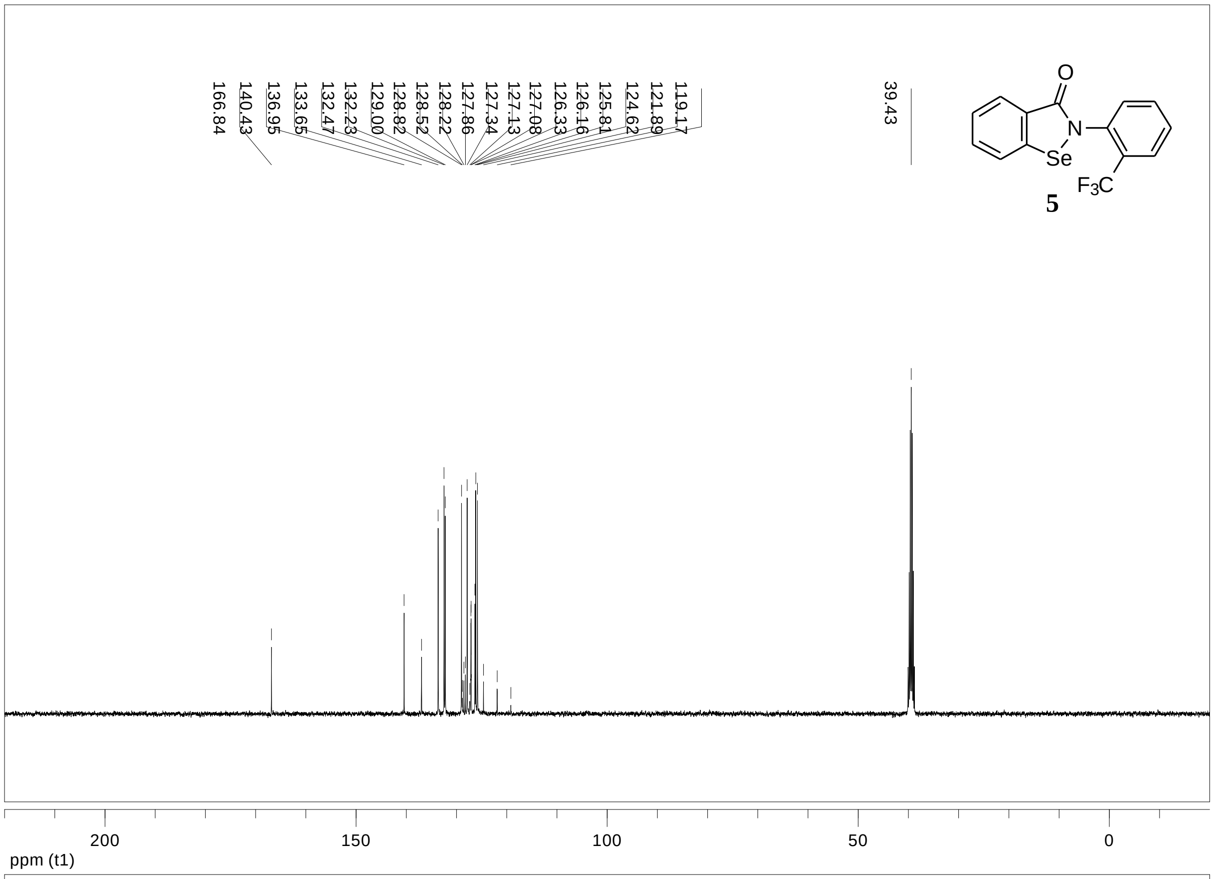

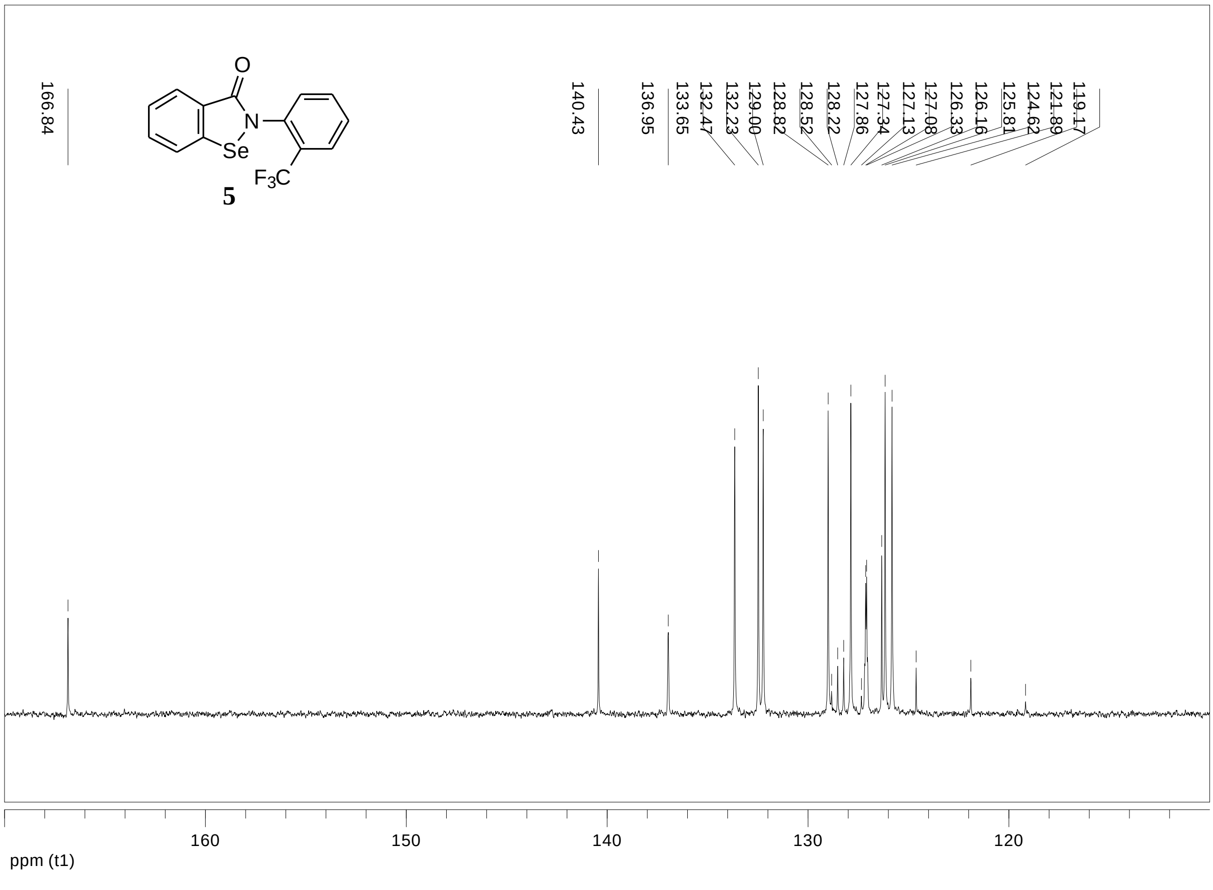

Fig. S31. ^{13}C-NMR (100.5 MHz, DMSO-d_6) spectrum of compound **5**

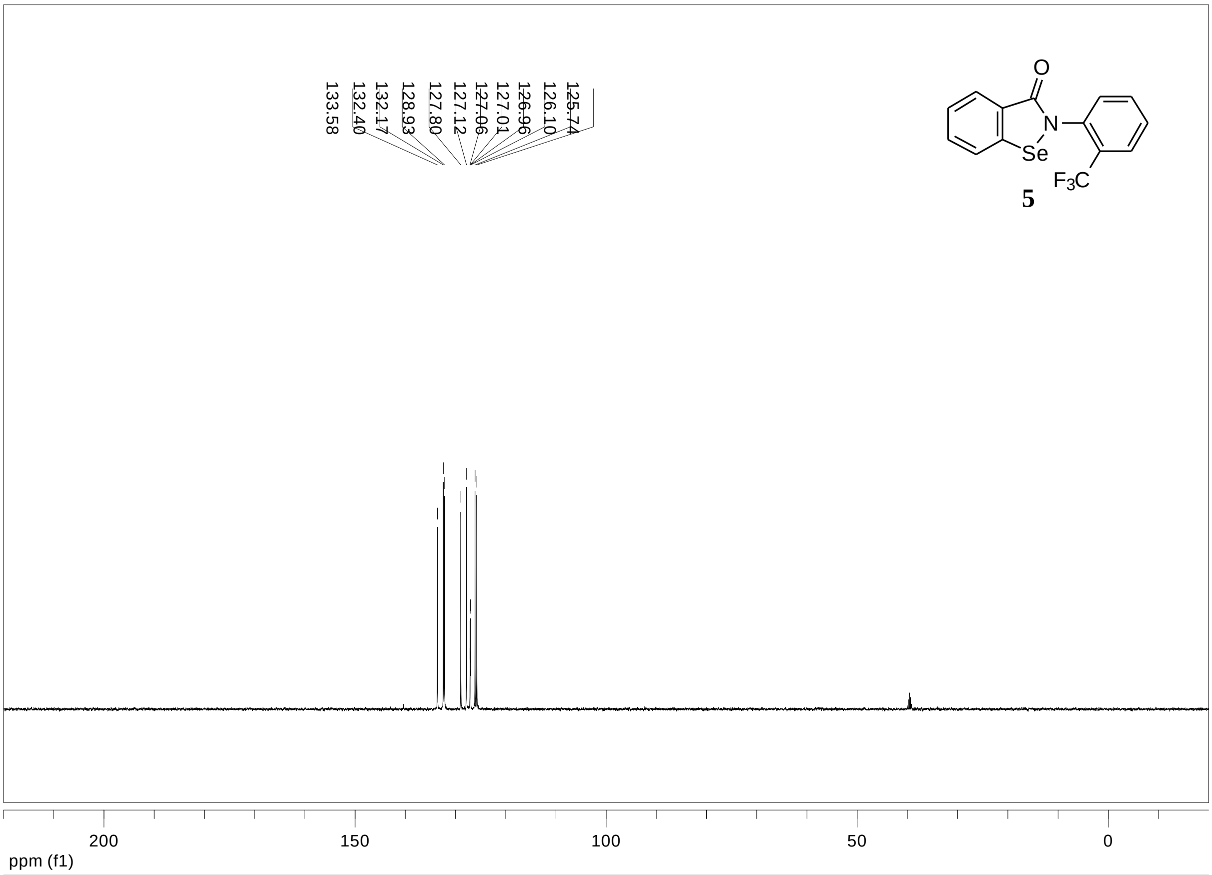

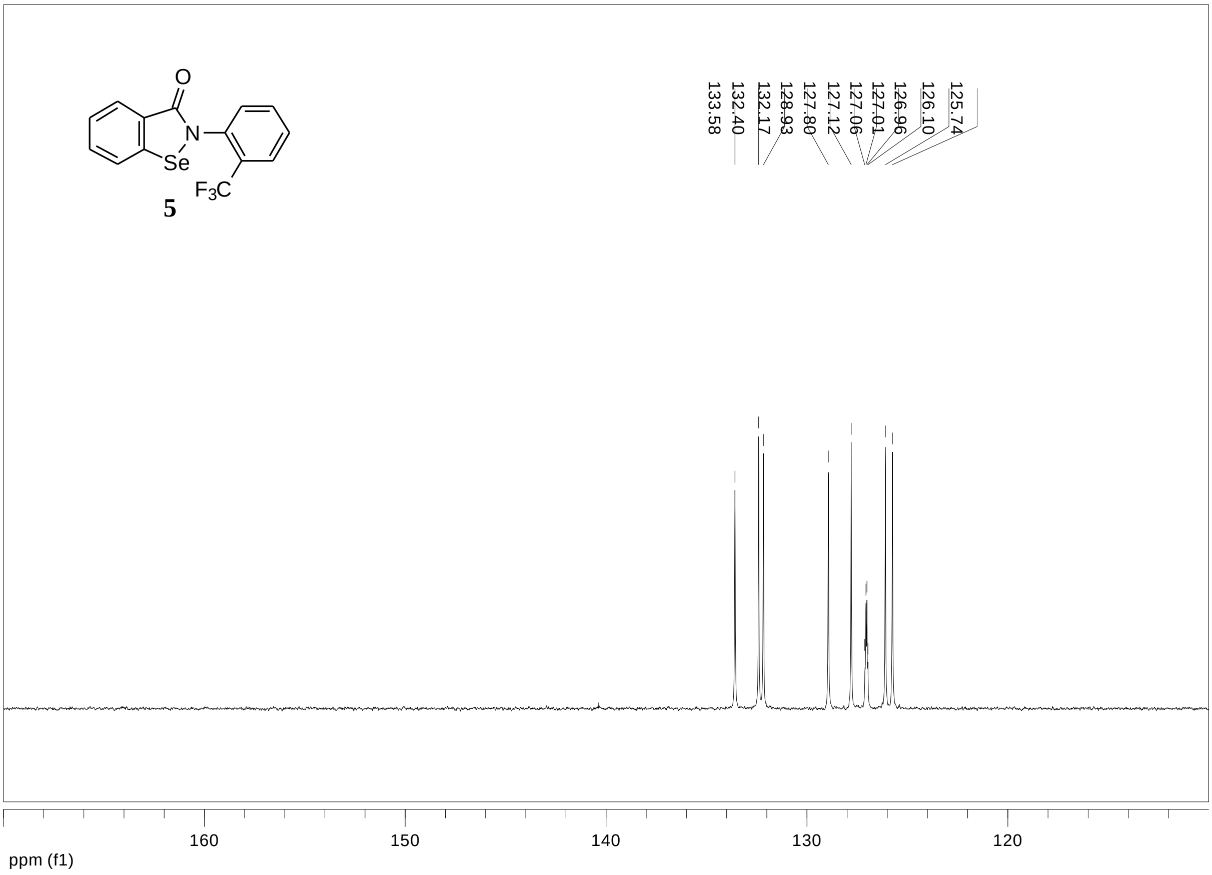

Fig. S32. ^{13}C-NMR (100.5 MHz, DMSO-d_6) dept-135 experiment of compound **5**

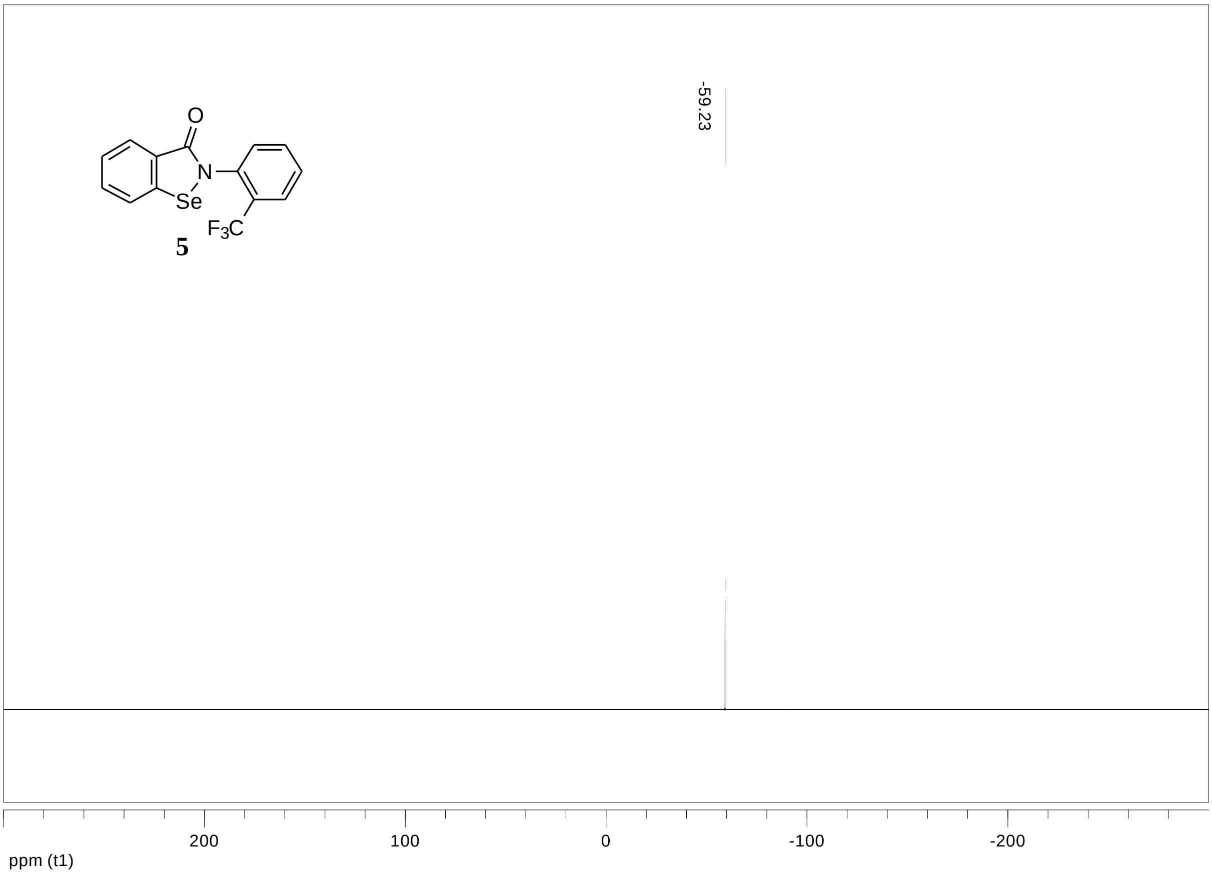

Fig. S33. ^{19}F-NMR (376.2 MHz, DMSO-d_6) spectrum of compound **5**

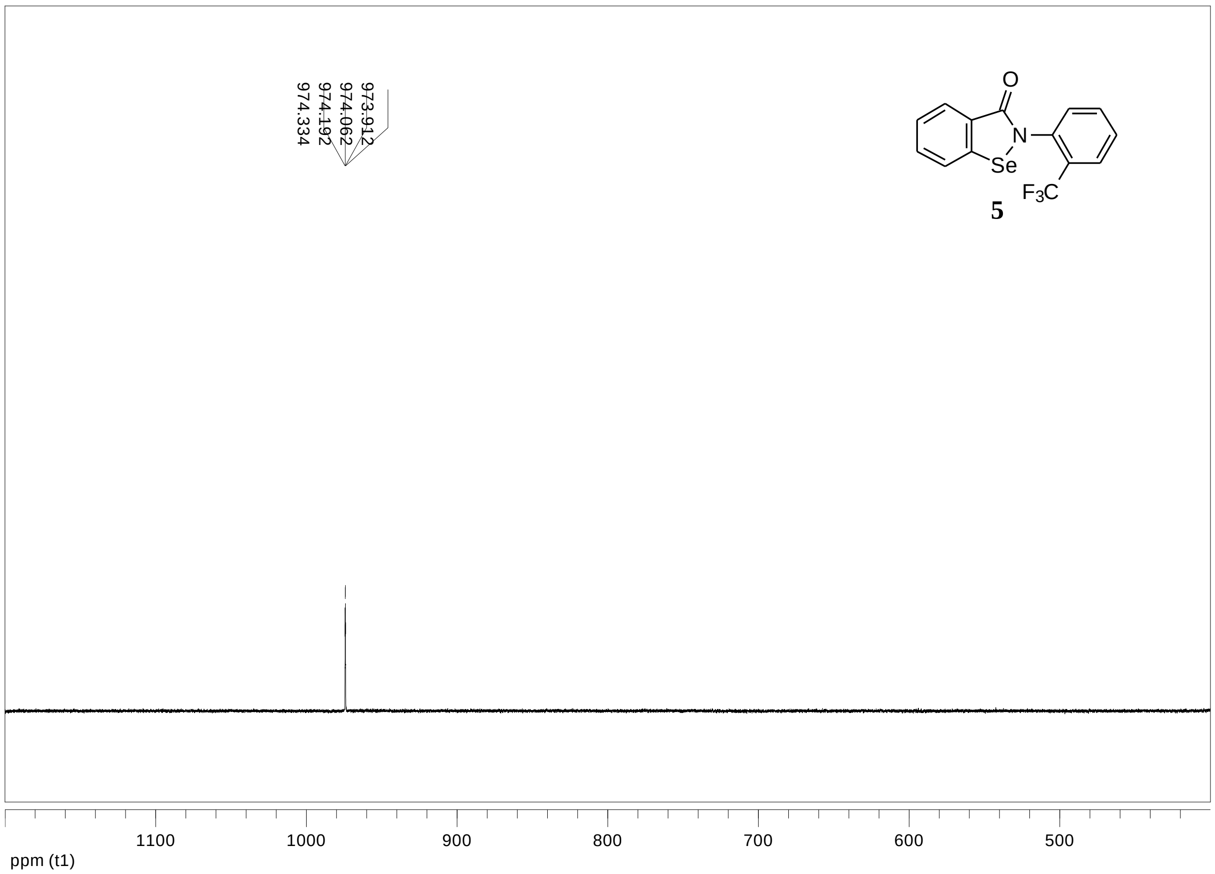

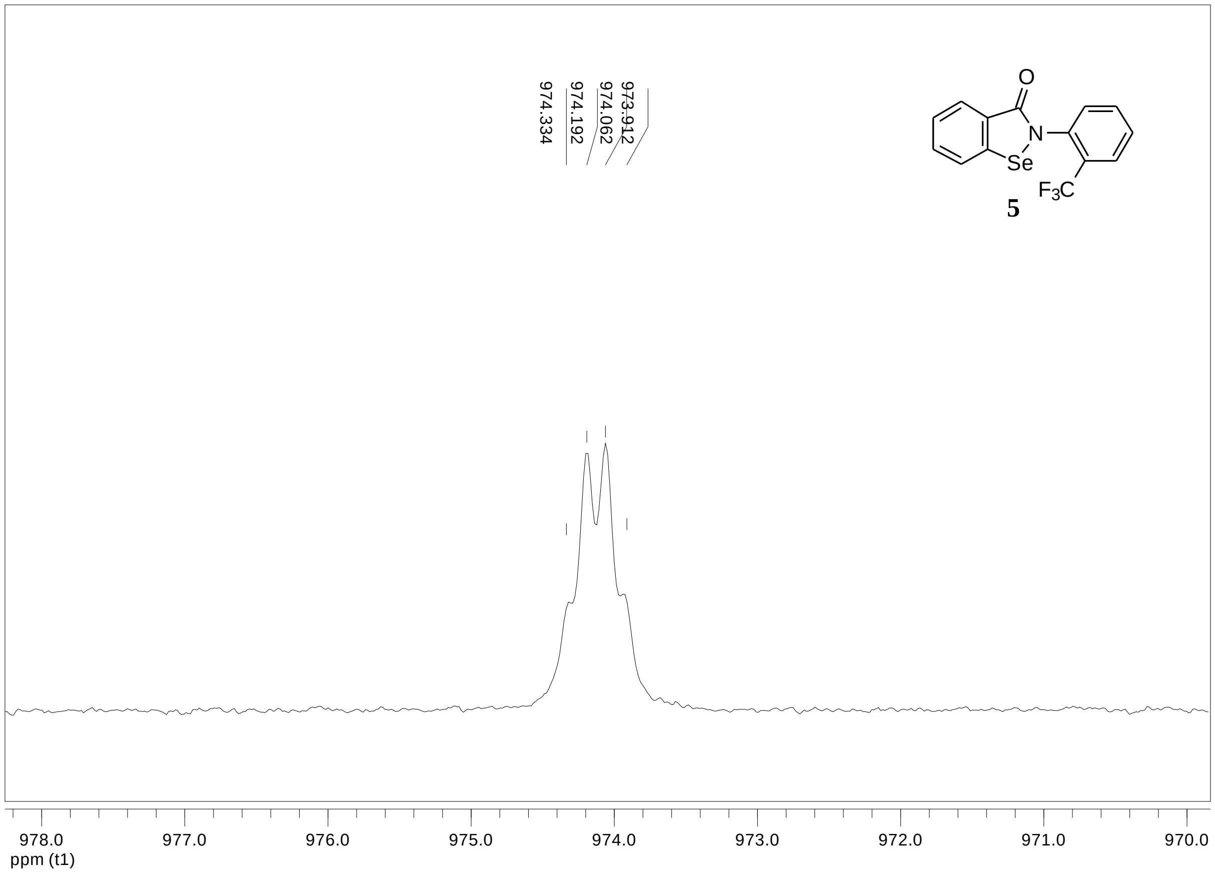

Fig. S34. ^{77}Se-NMR (76.24 MHz, DMSO-d_6) spectrum of compound **5**

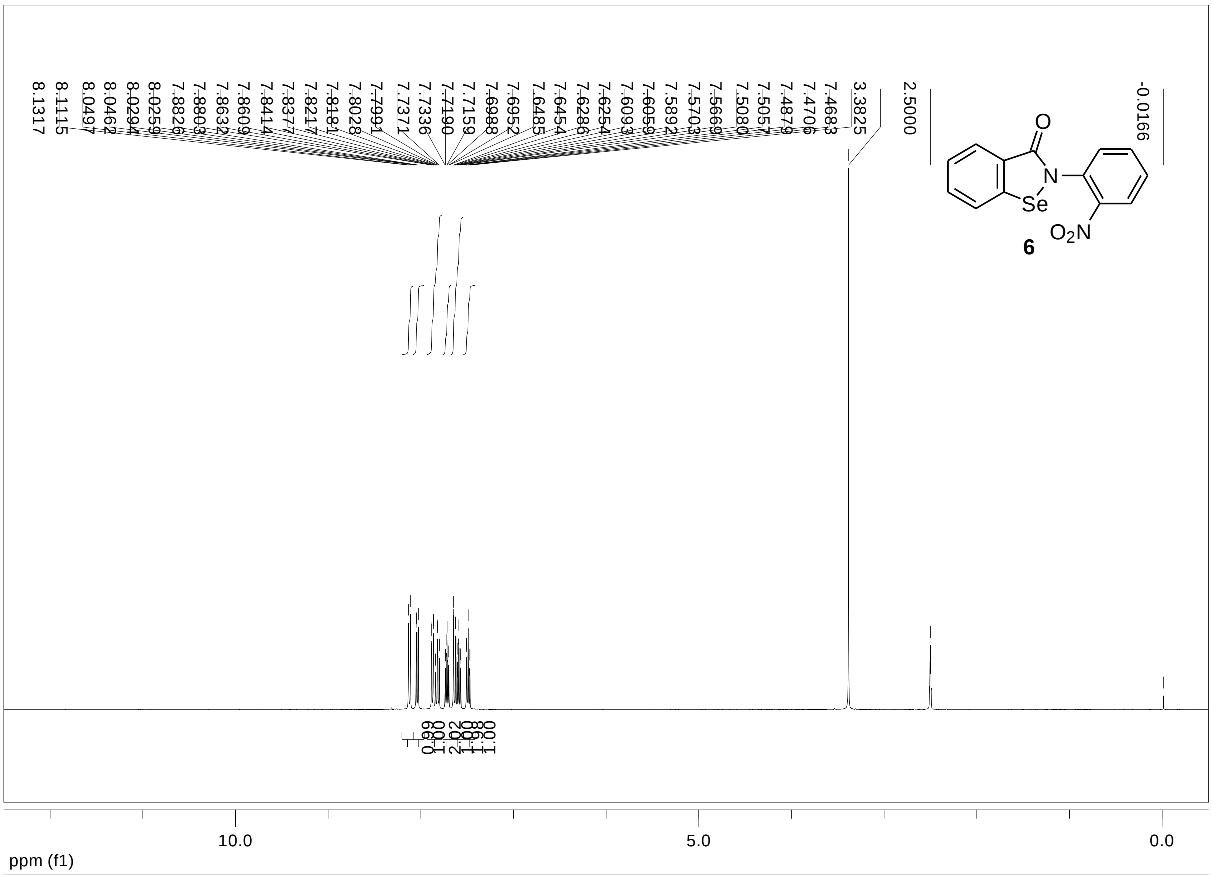

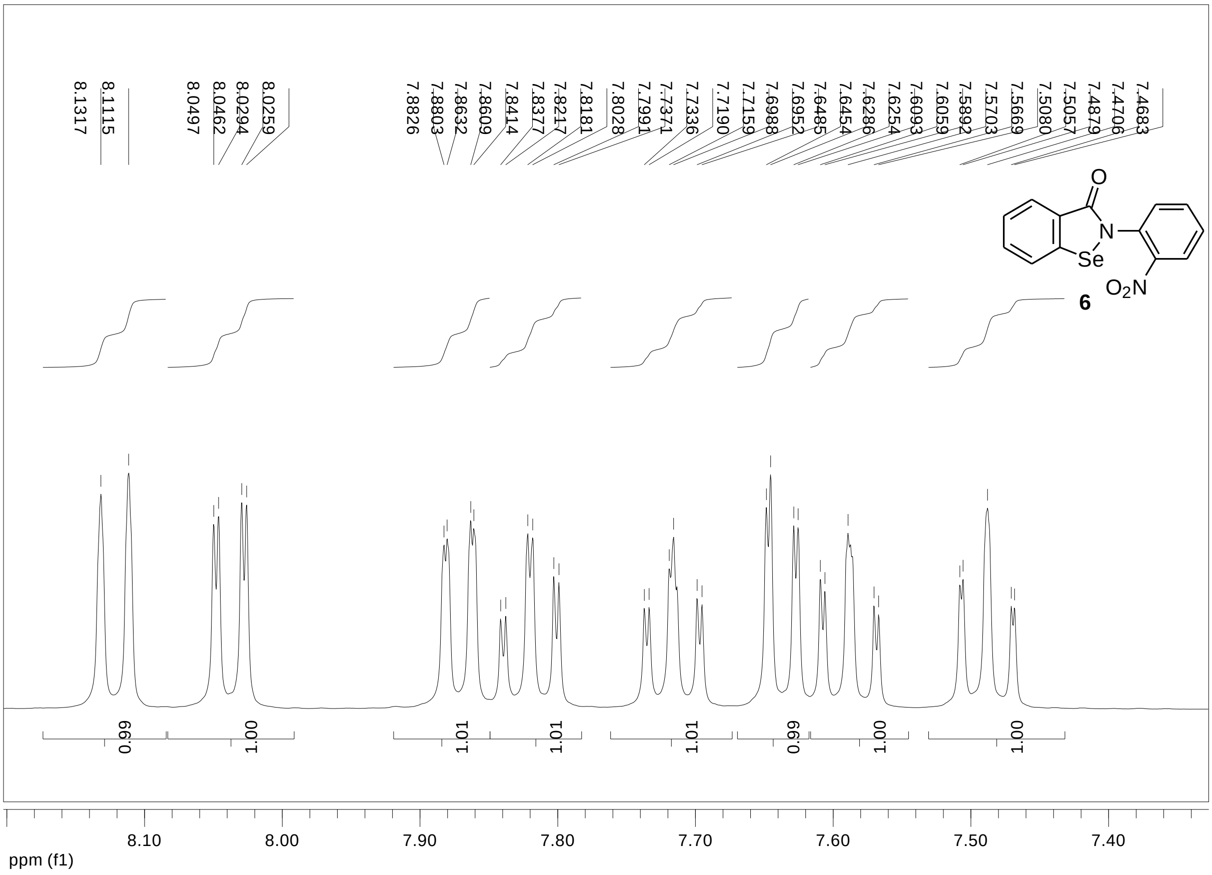

Fig. S35. ^{1}H-NMR (399.8 MHz, DMSO-d_6) spectrum of compound **6**

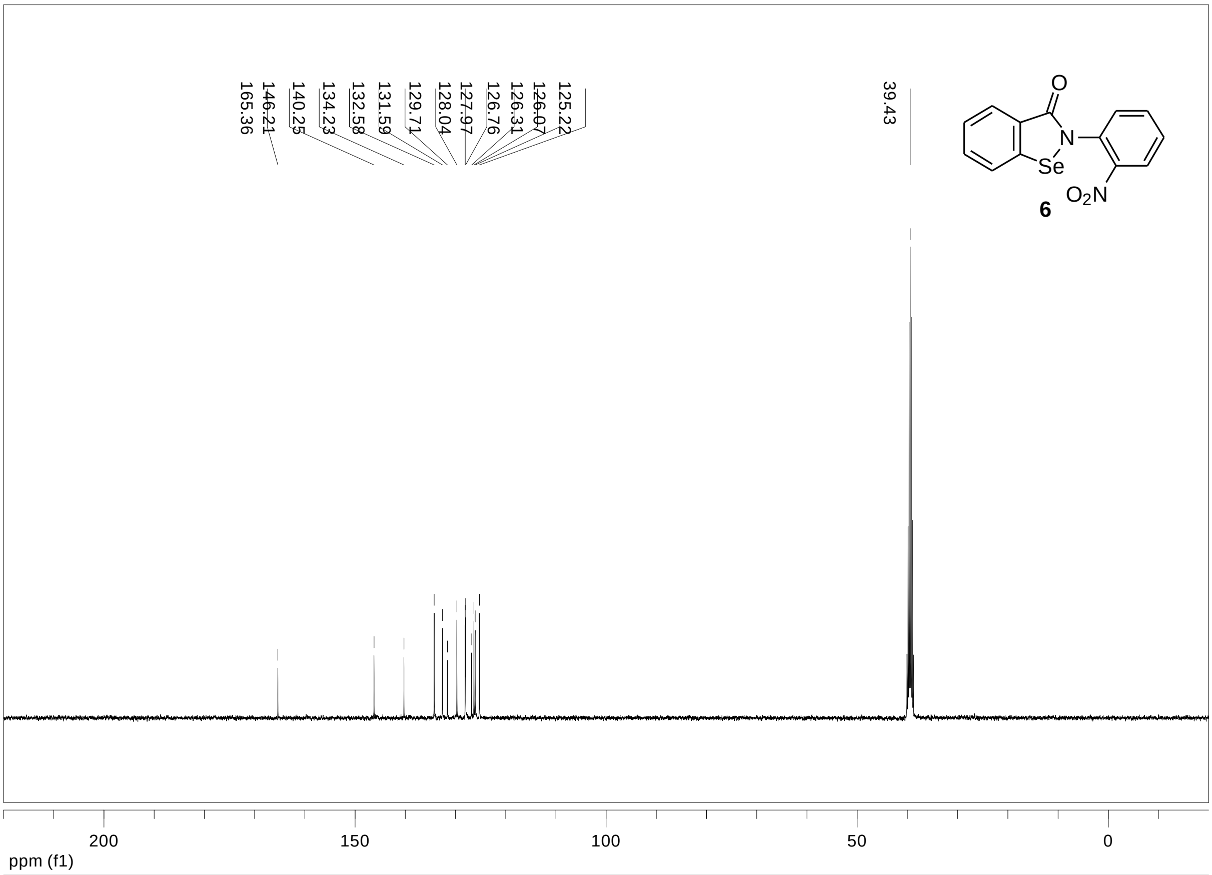

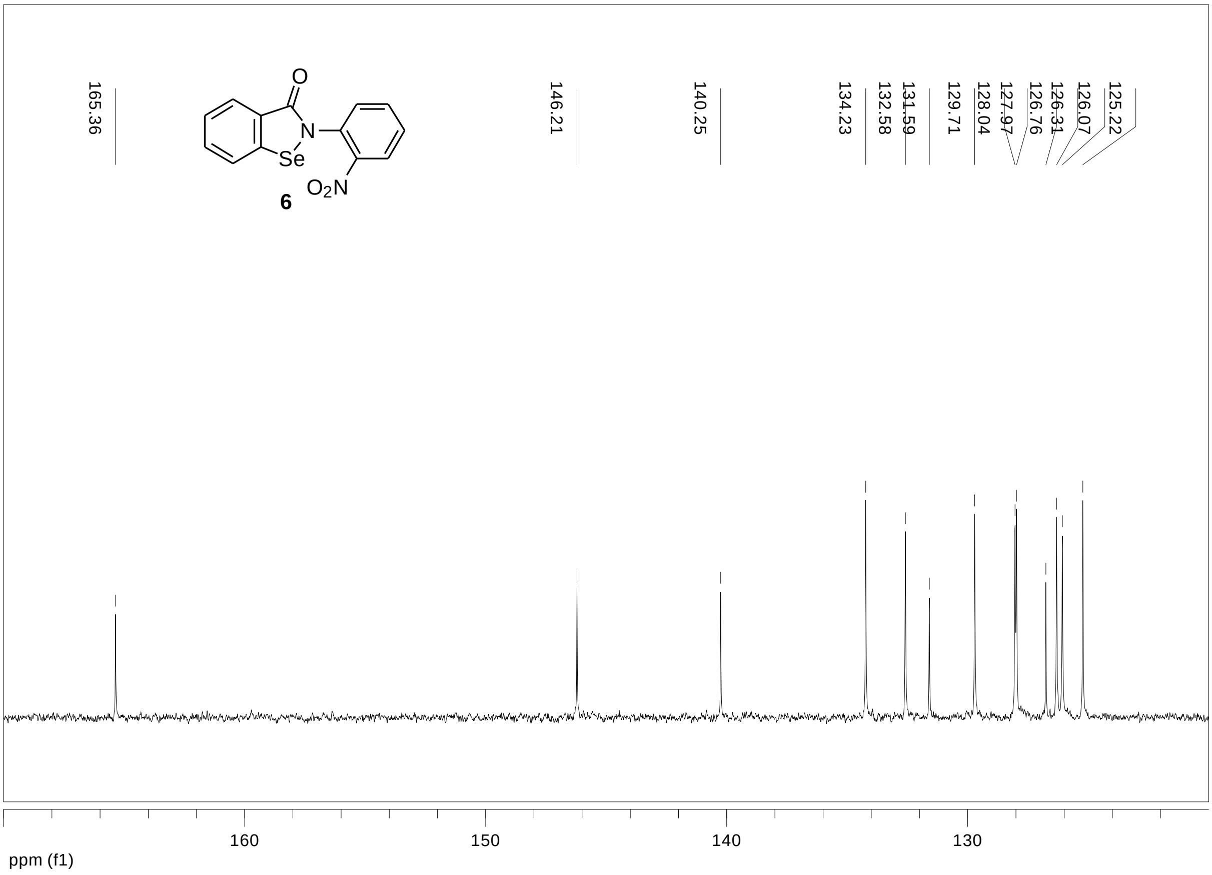

Fig. S36. ^{13}C-NMR (100.5 MHz, DMSO-d_6) spectrum of compound **6**

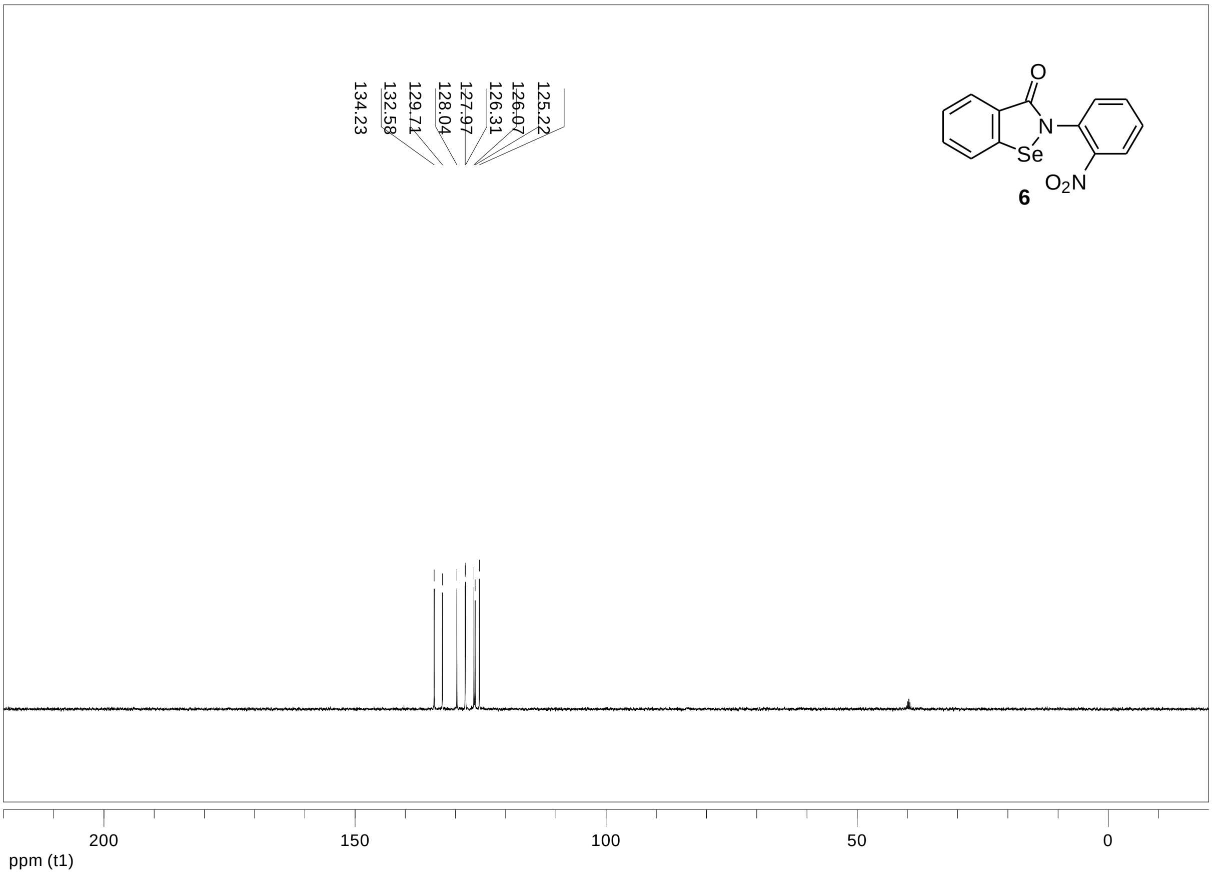

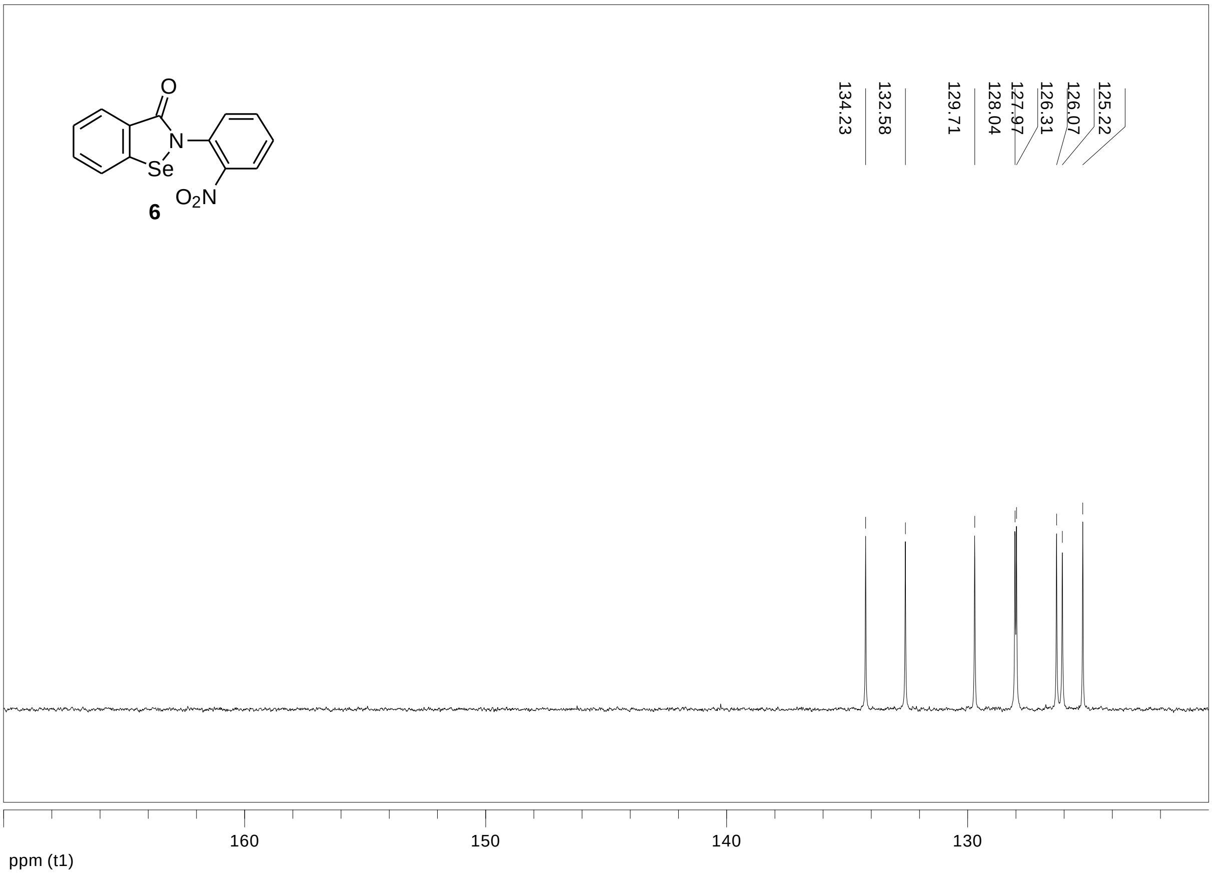

Fig. S37. ^{13}C-NMR (100.5 MHz, DMSO-d_6) dept-135 experiment of compound **6**

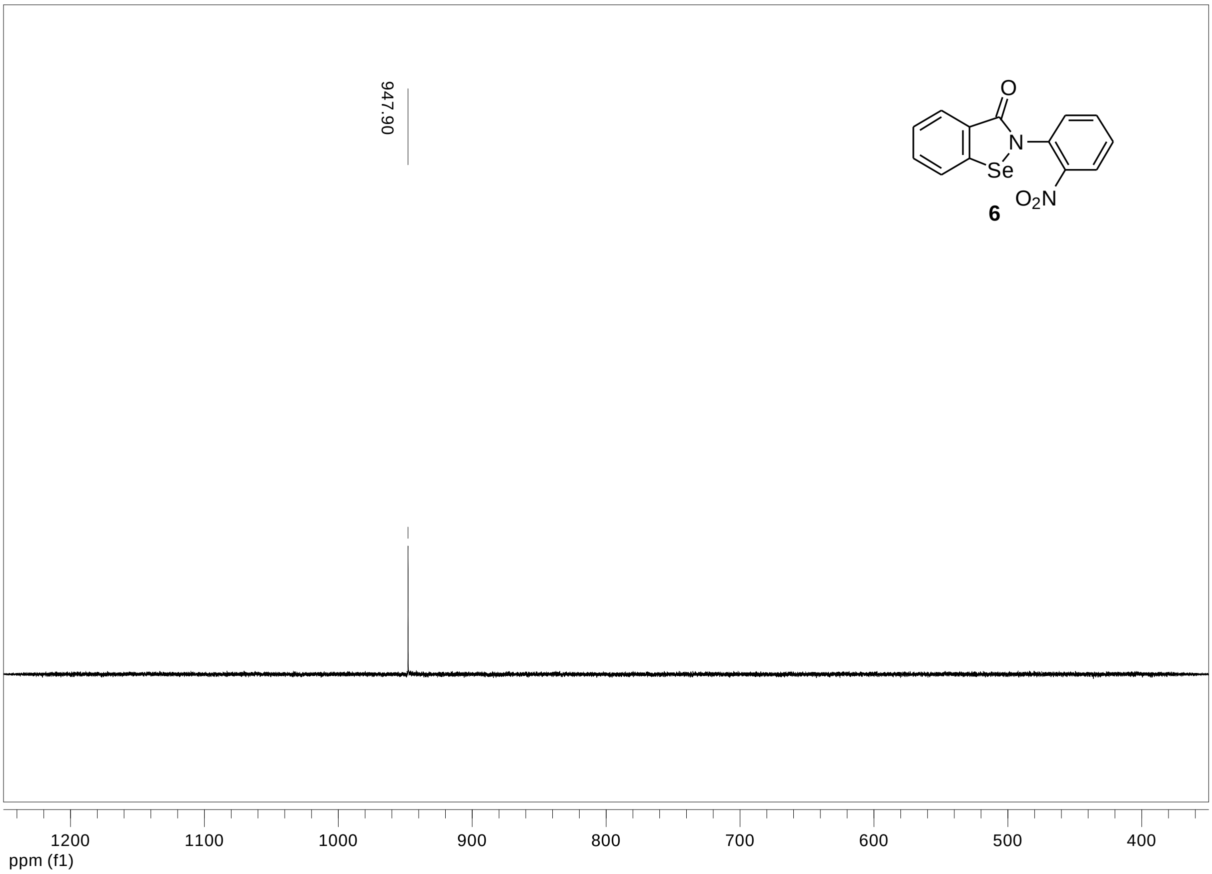

Fig. S38. ^{77}Se-NMR (76.24 MHz, DMSO-d_6) spectrum of compound **6**

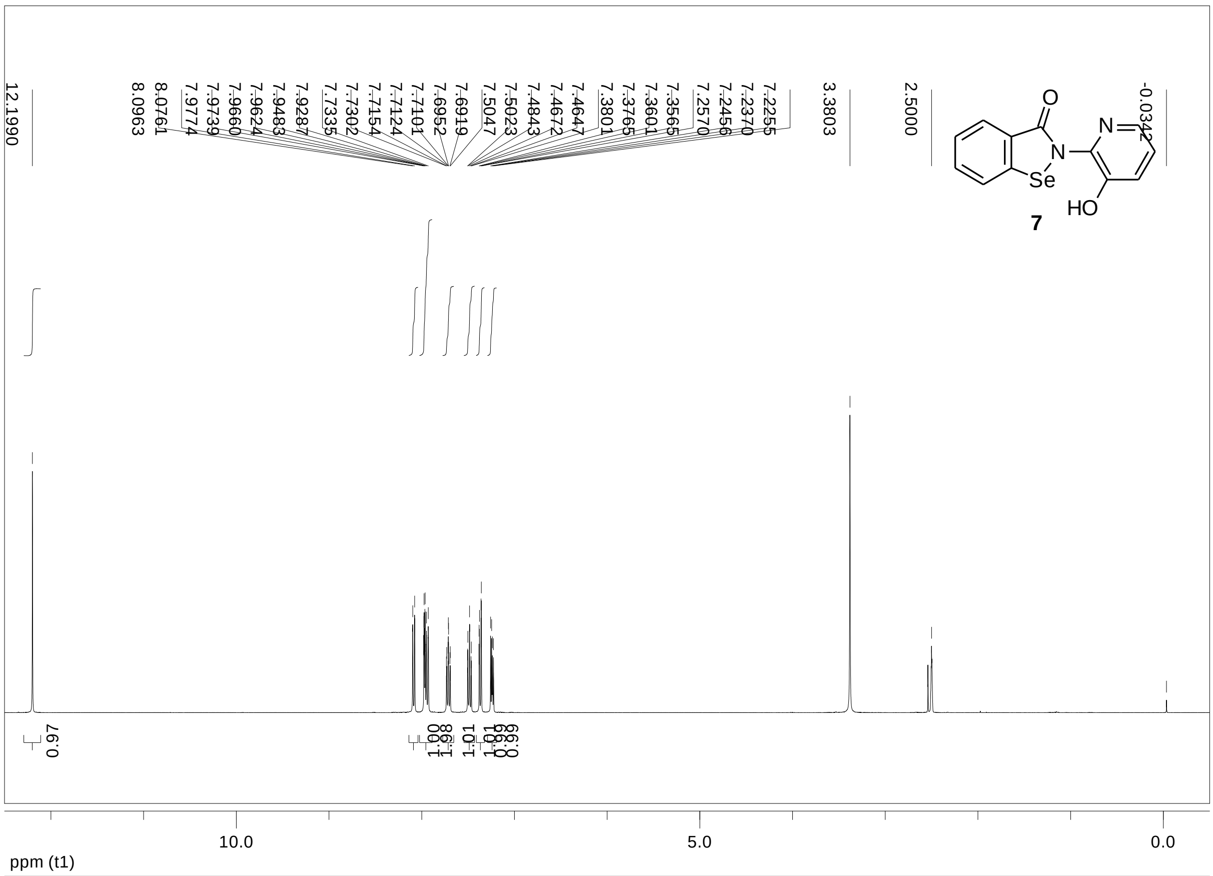

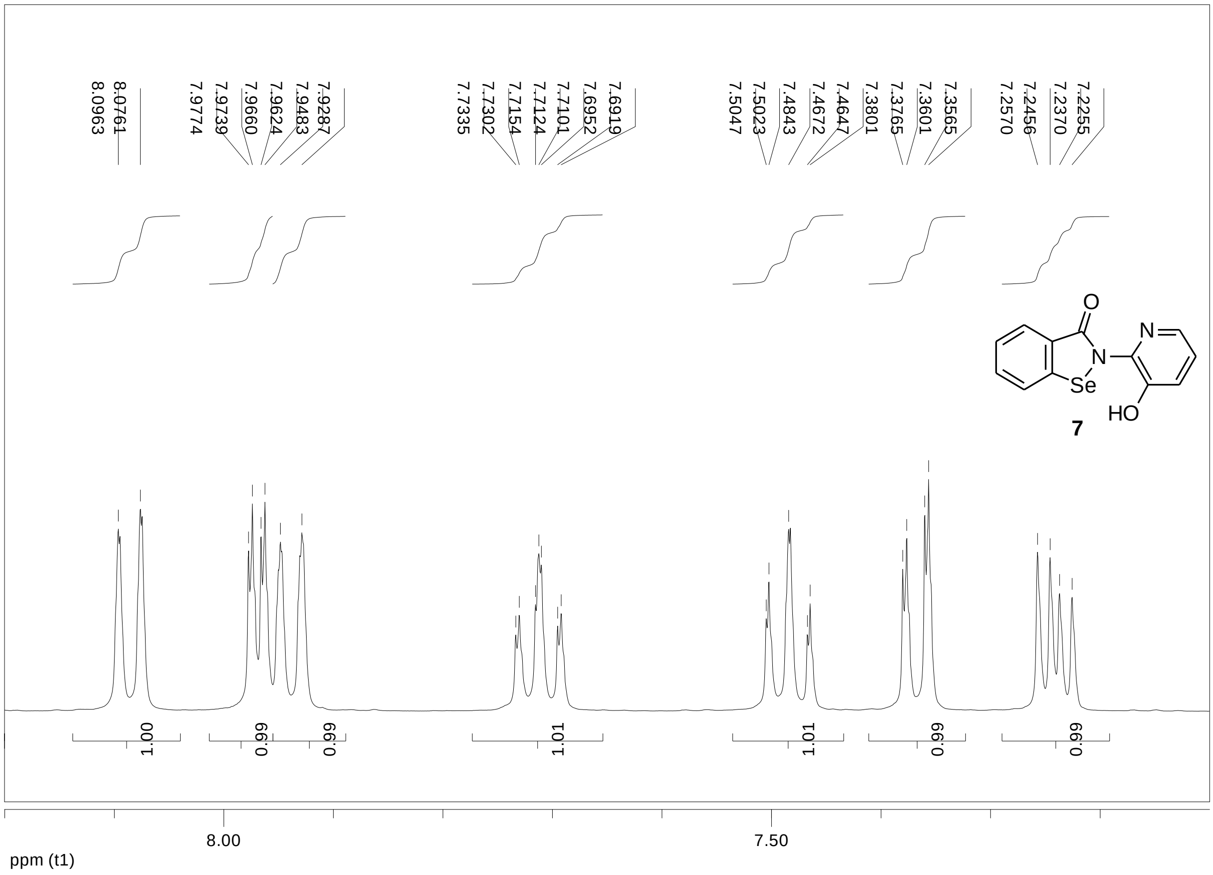

Fig. S39. ^{1}H-NMR (399.8 MHz, DMSO-d_6) spectrum of compound **7**

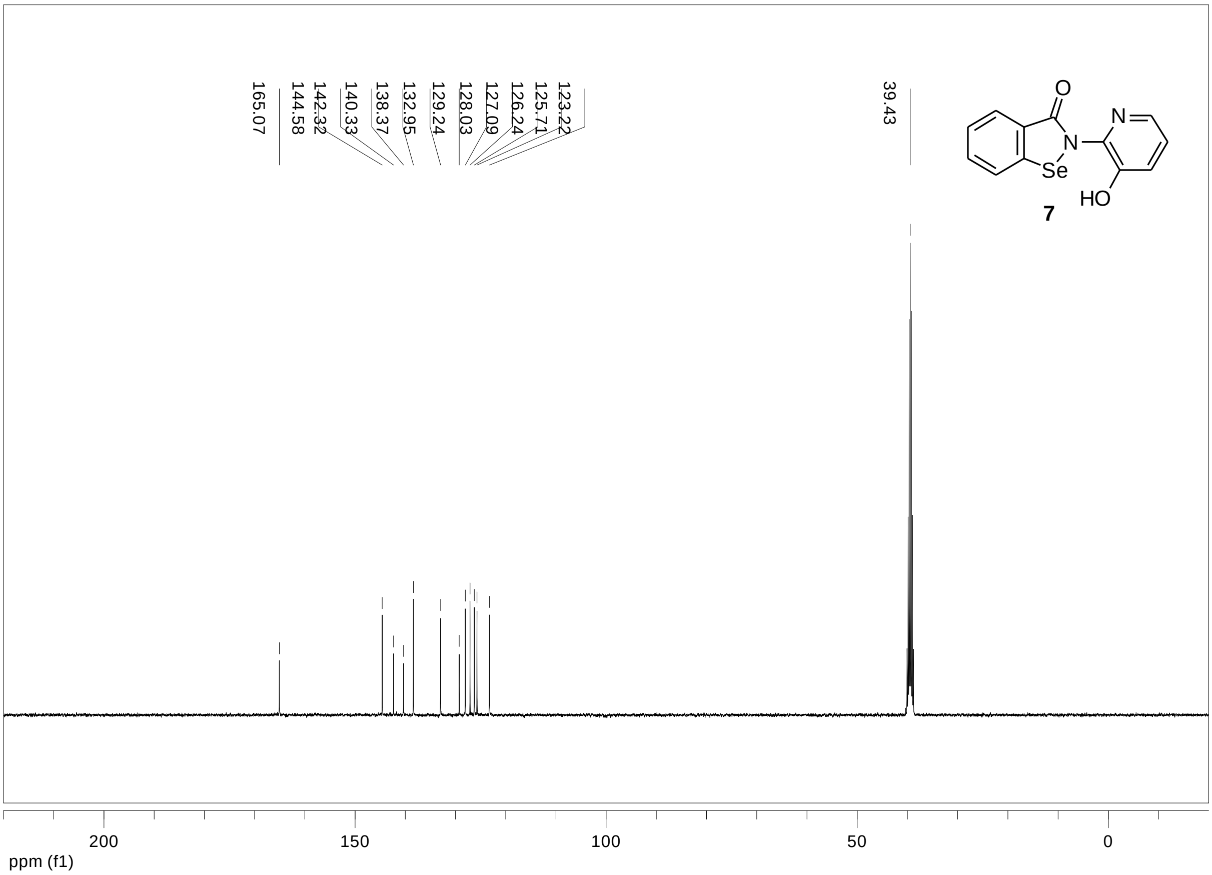

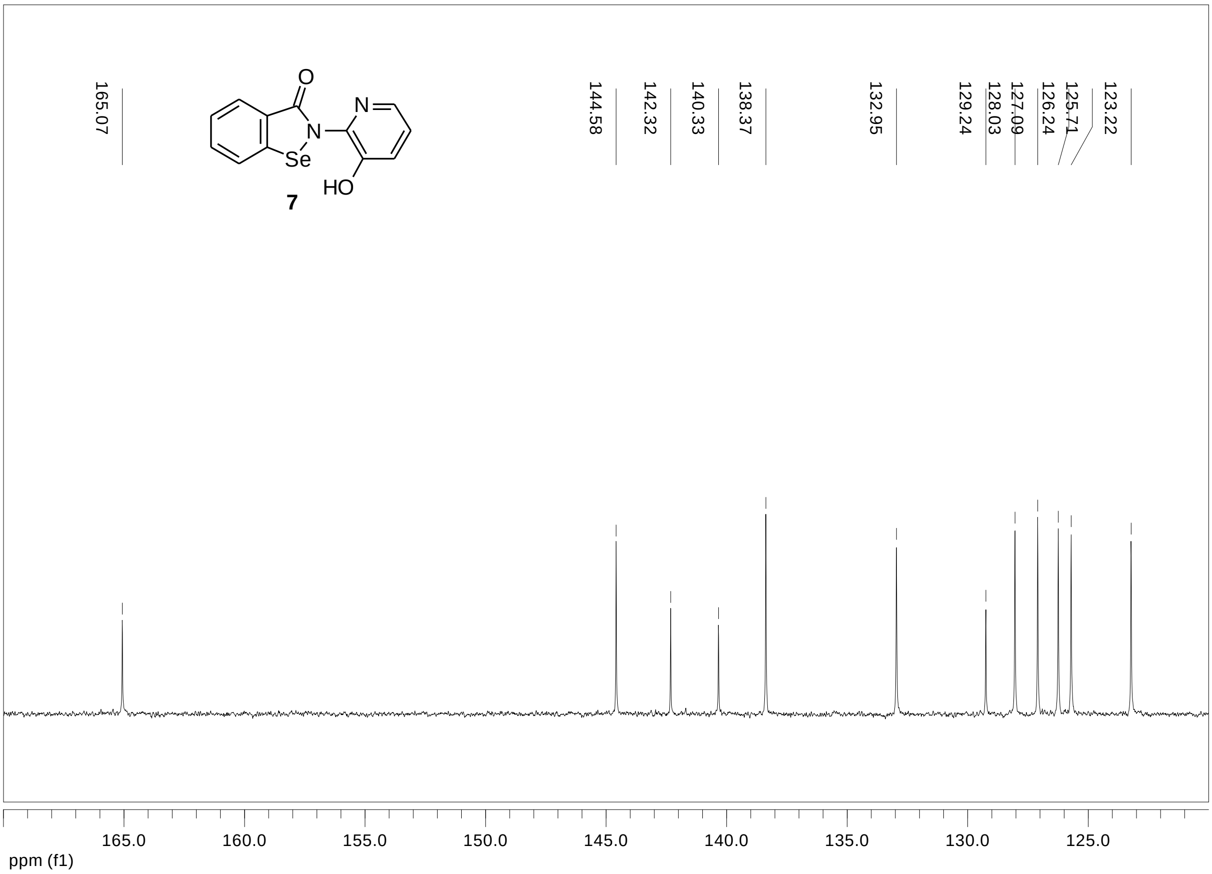

Fig. S40. ^{13}C-NMR (100.5 MHz, DMSO-d_6) spectrum of compound **7**

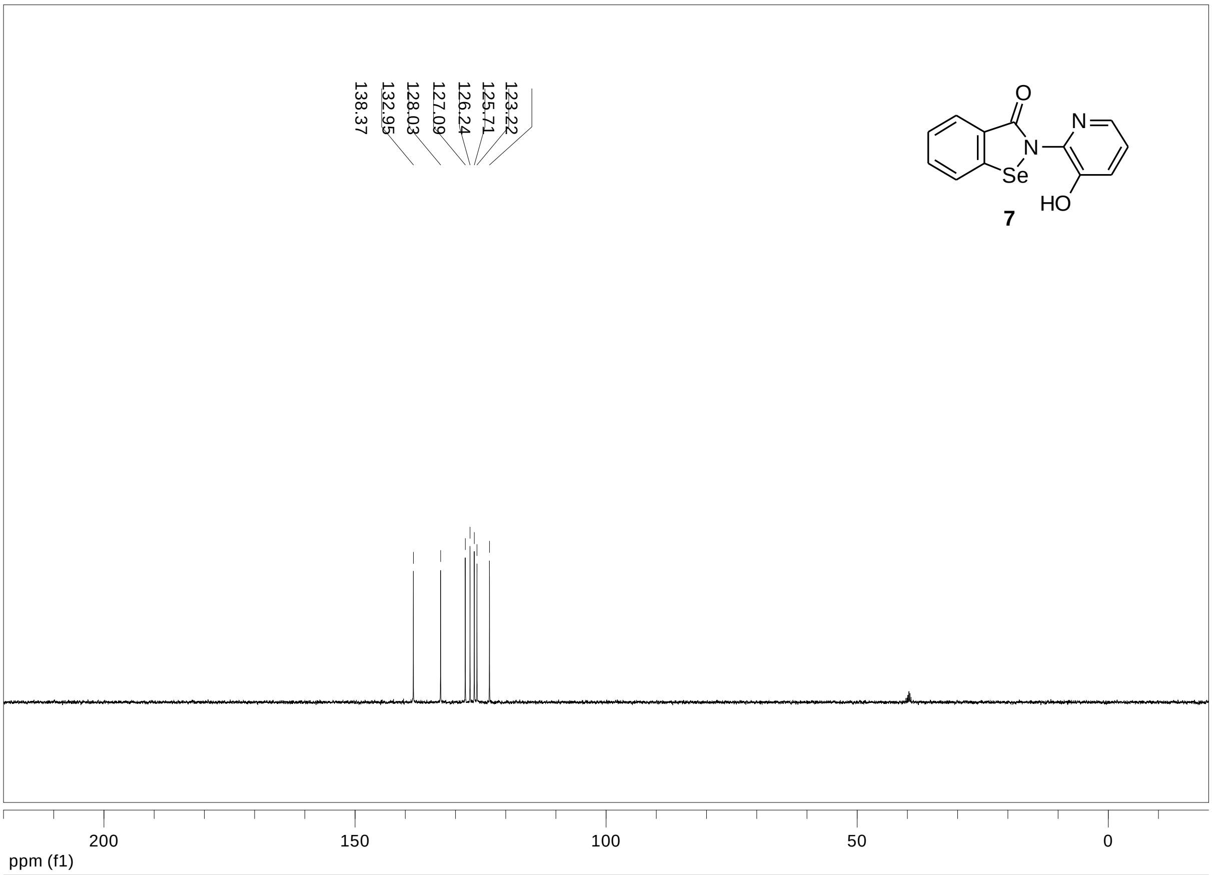

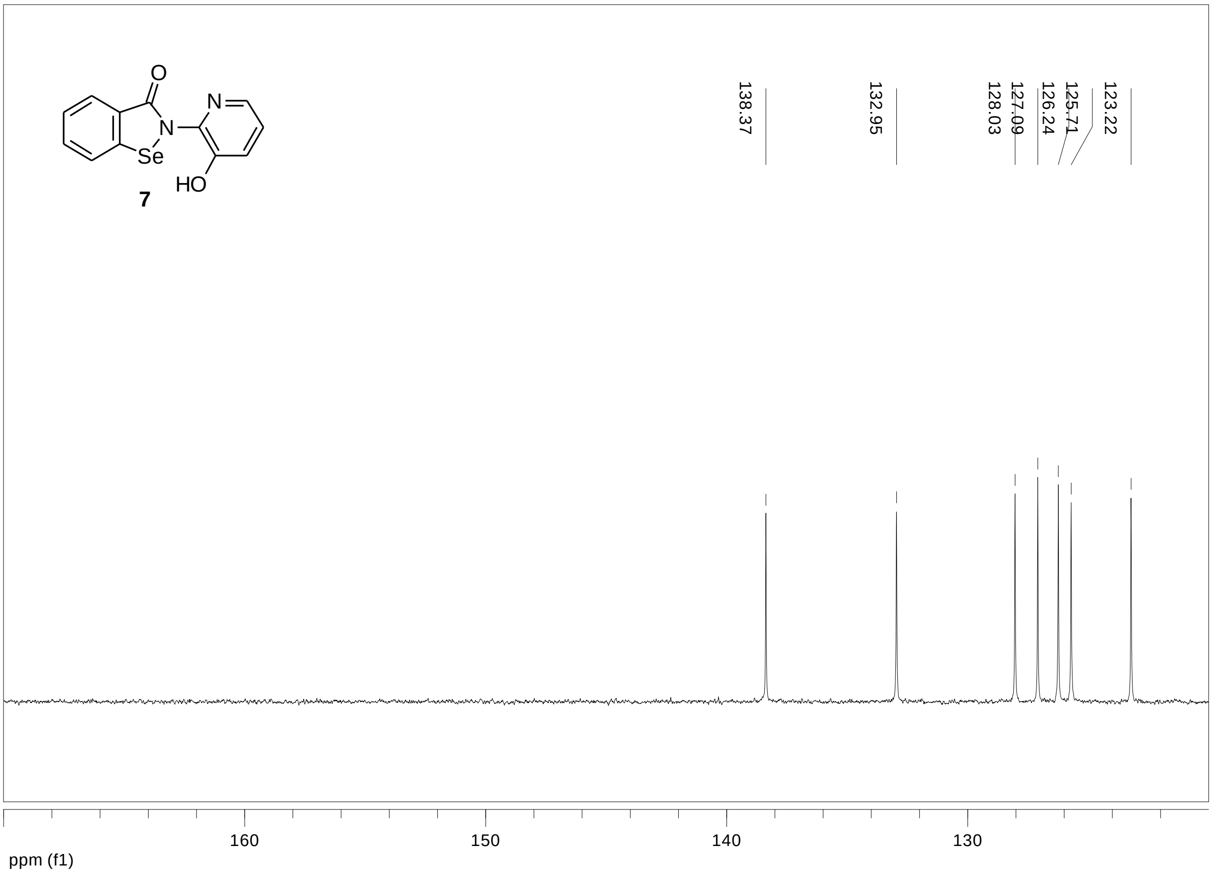

Fig. S41. ^{13}C-NMR (100.5 MHz, DMSO-d_6) dept-135 experiment of compound **7**

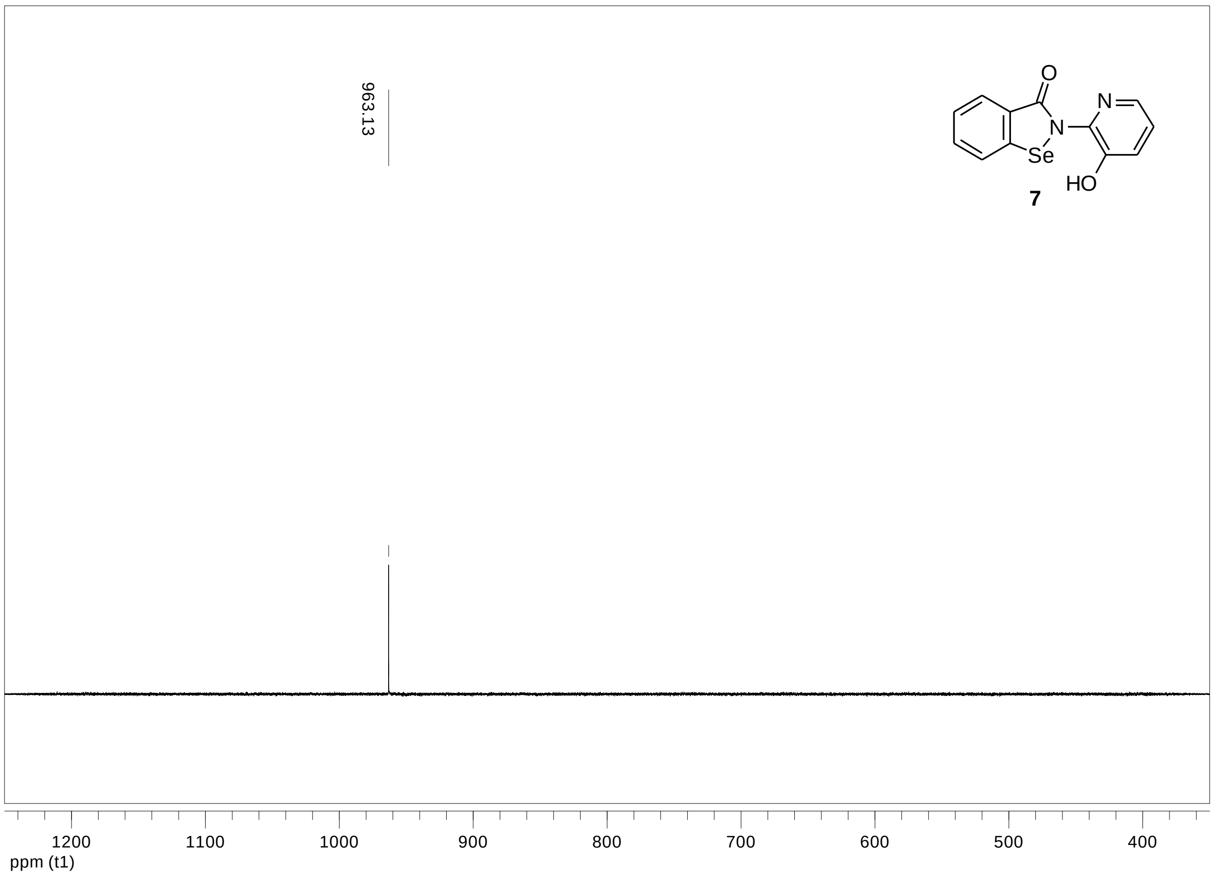

Fig. S42. ^{77}Se-NMR (76.24 MHz, DMSO-d_6) spectrum of compound **7**

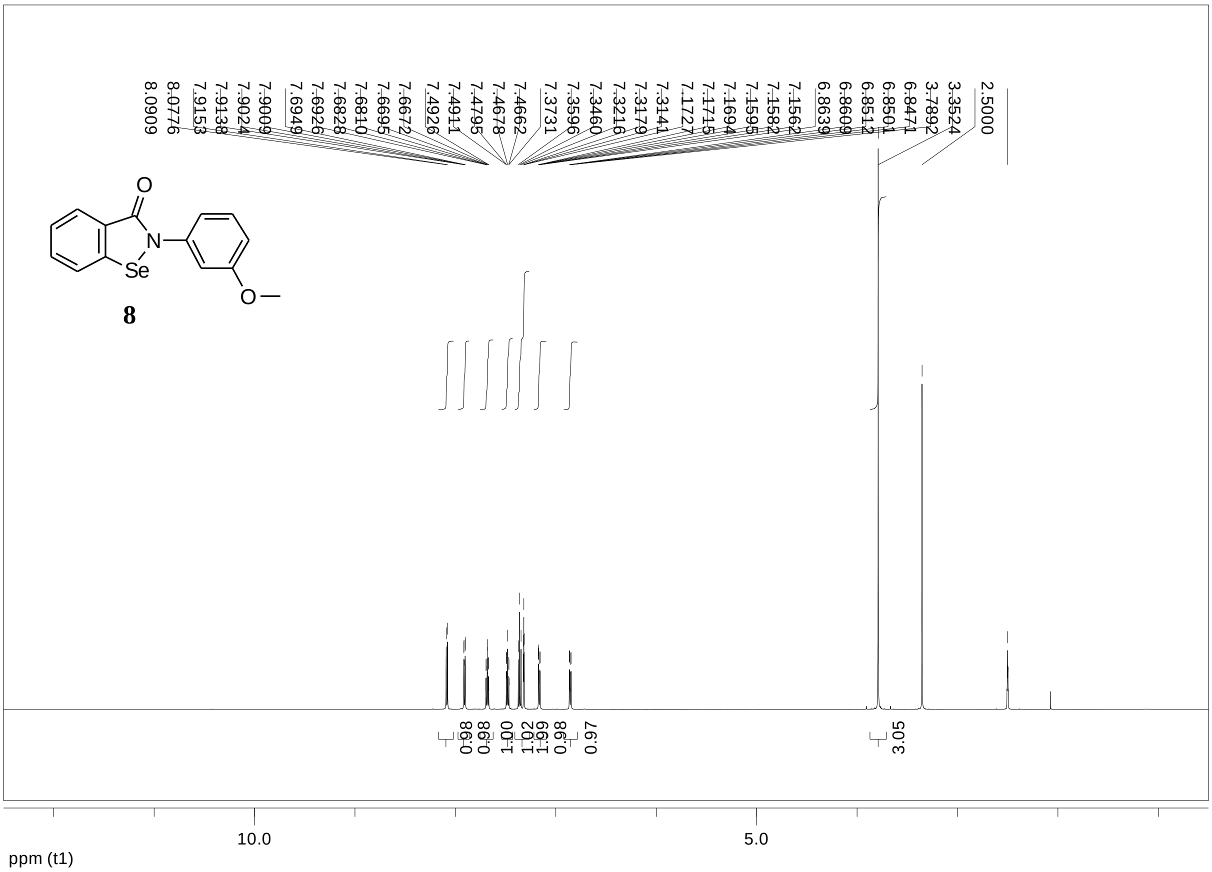

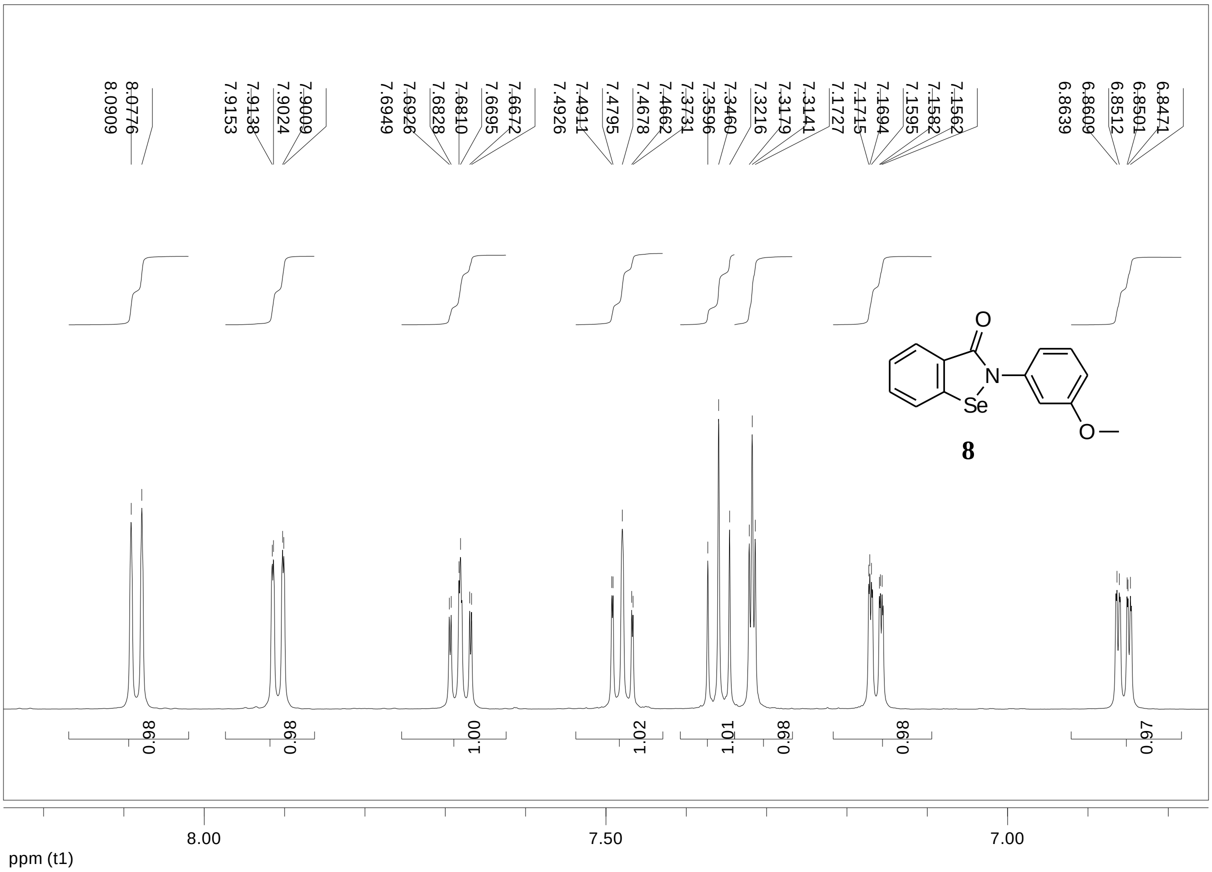

Fig. S43. ^{1}H-NMR (600.6 MHz, DMSO-d_6) spectrum of compound **8**

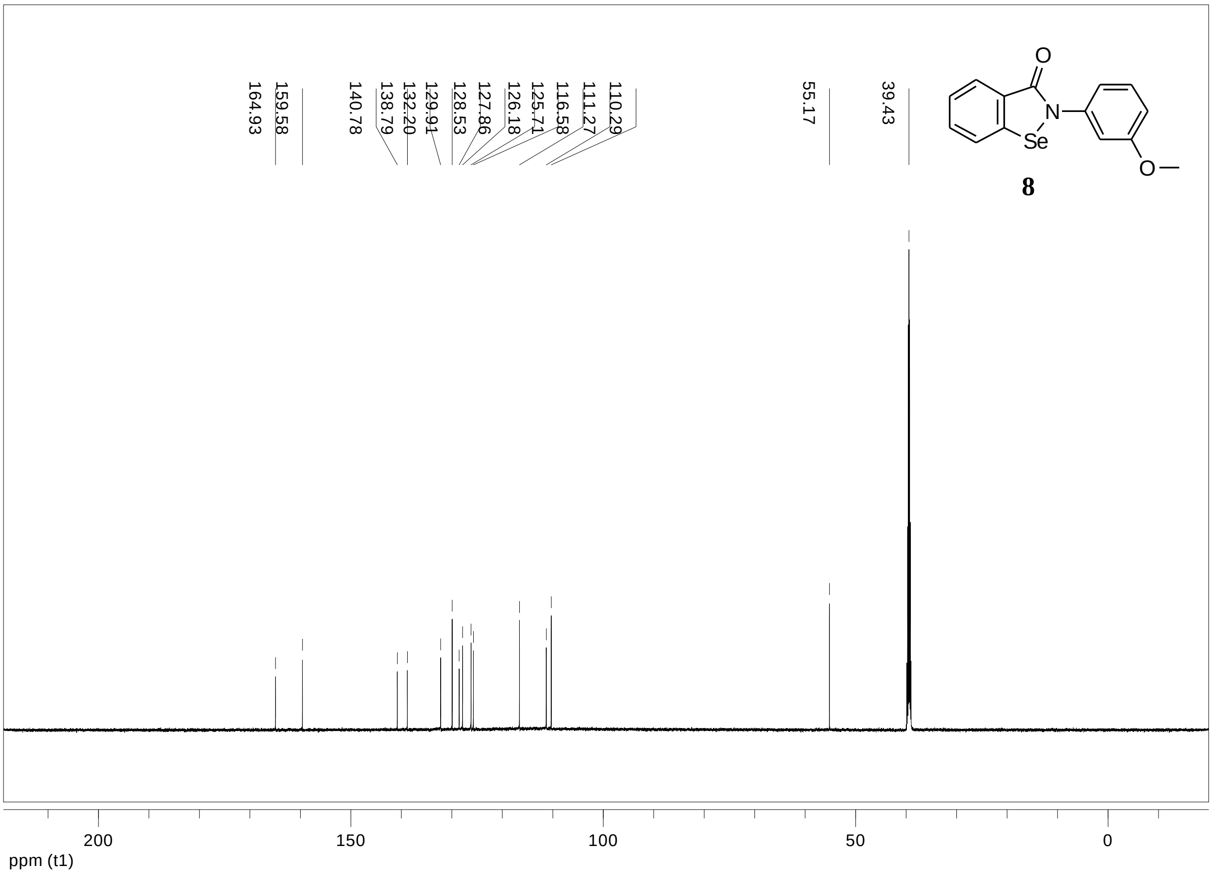

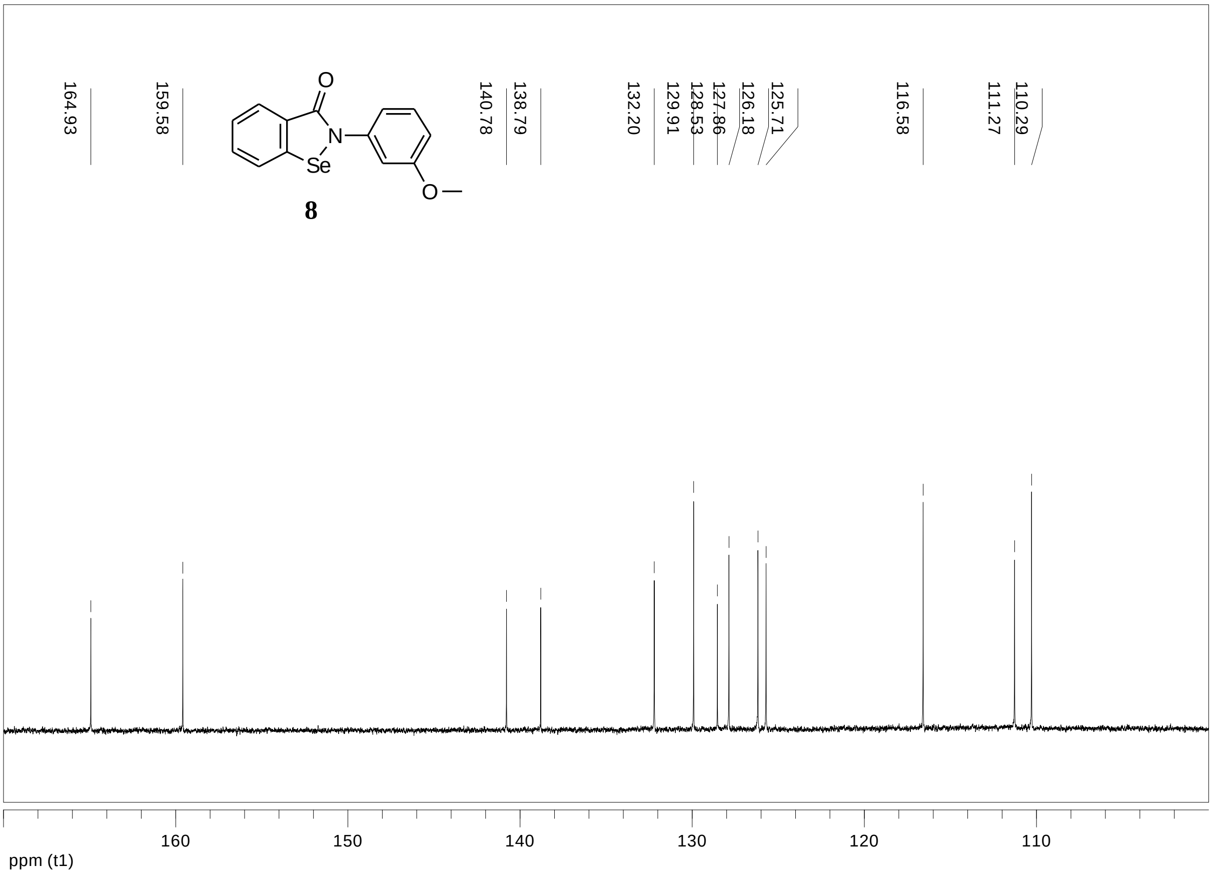

Fig. S44. ^{13}C-NMR (150.1 MHz, DMSO-d_6) spectrum of compound **8**

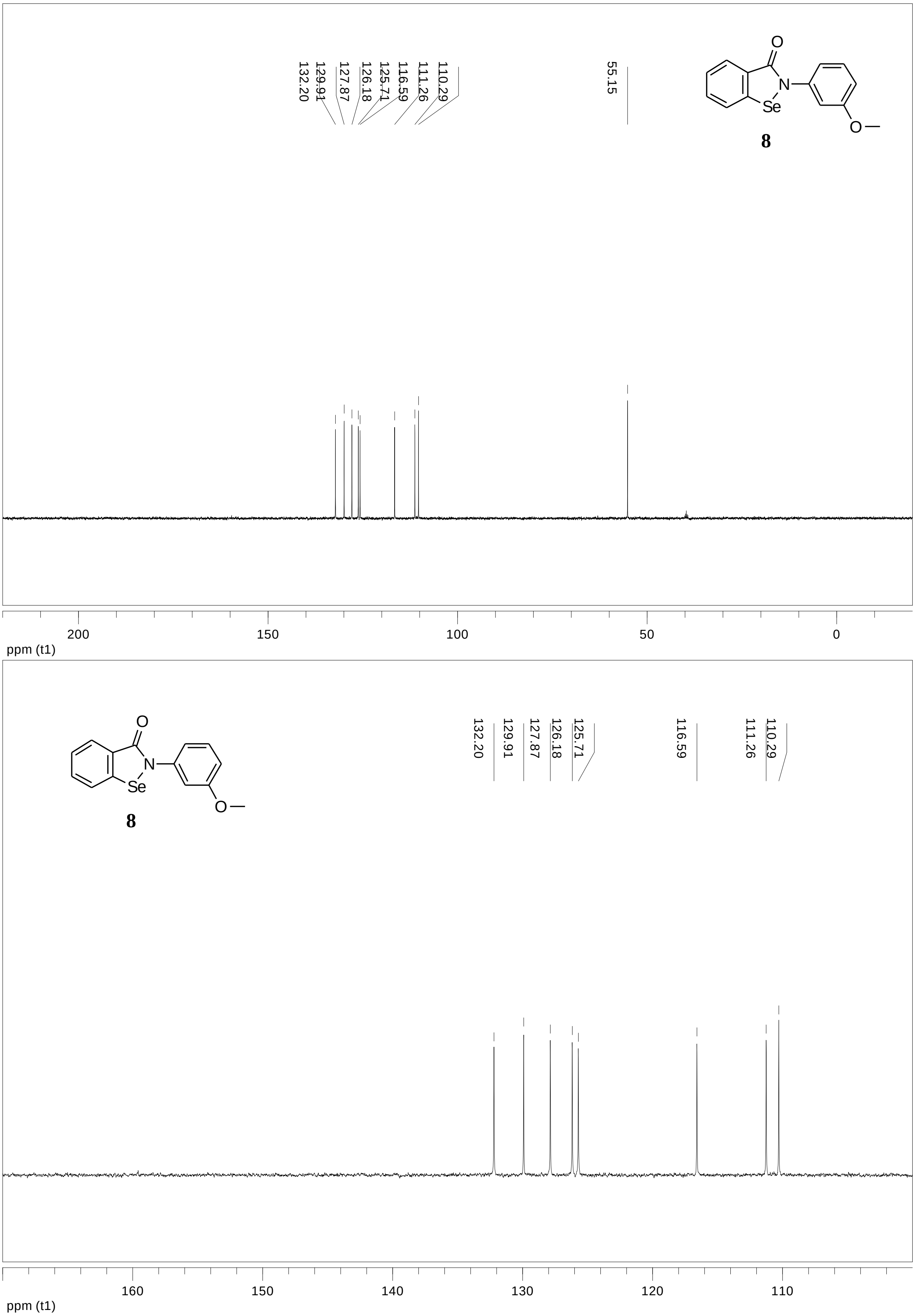

Fig. S45. ^{13}C-NMR (100.5 MHz, DMSO-d_6) dept-135 experiment of compound **8**

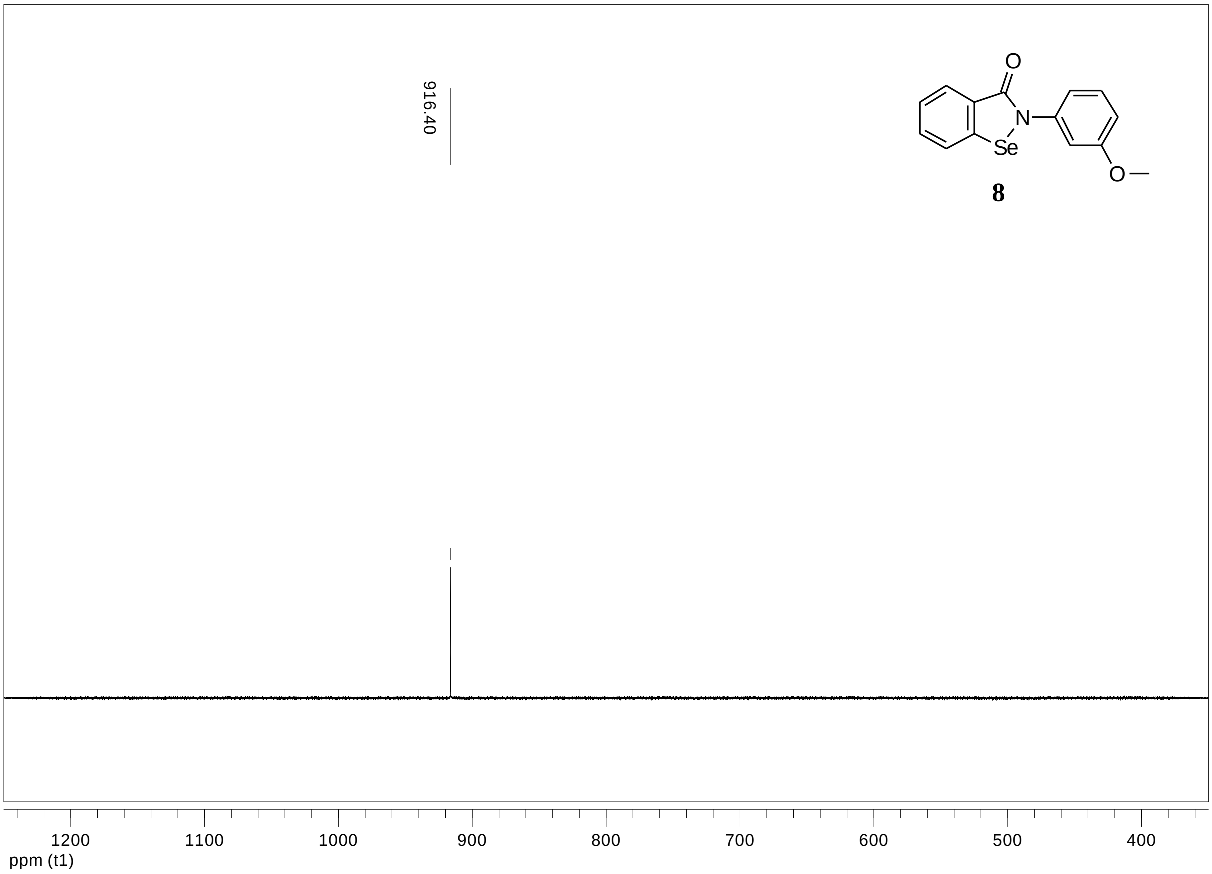

Fig. S46. ^{77}Se-NMR (76.24 MHz, DMSO-d_6) spectrum of compound **8**

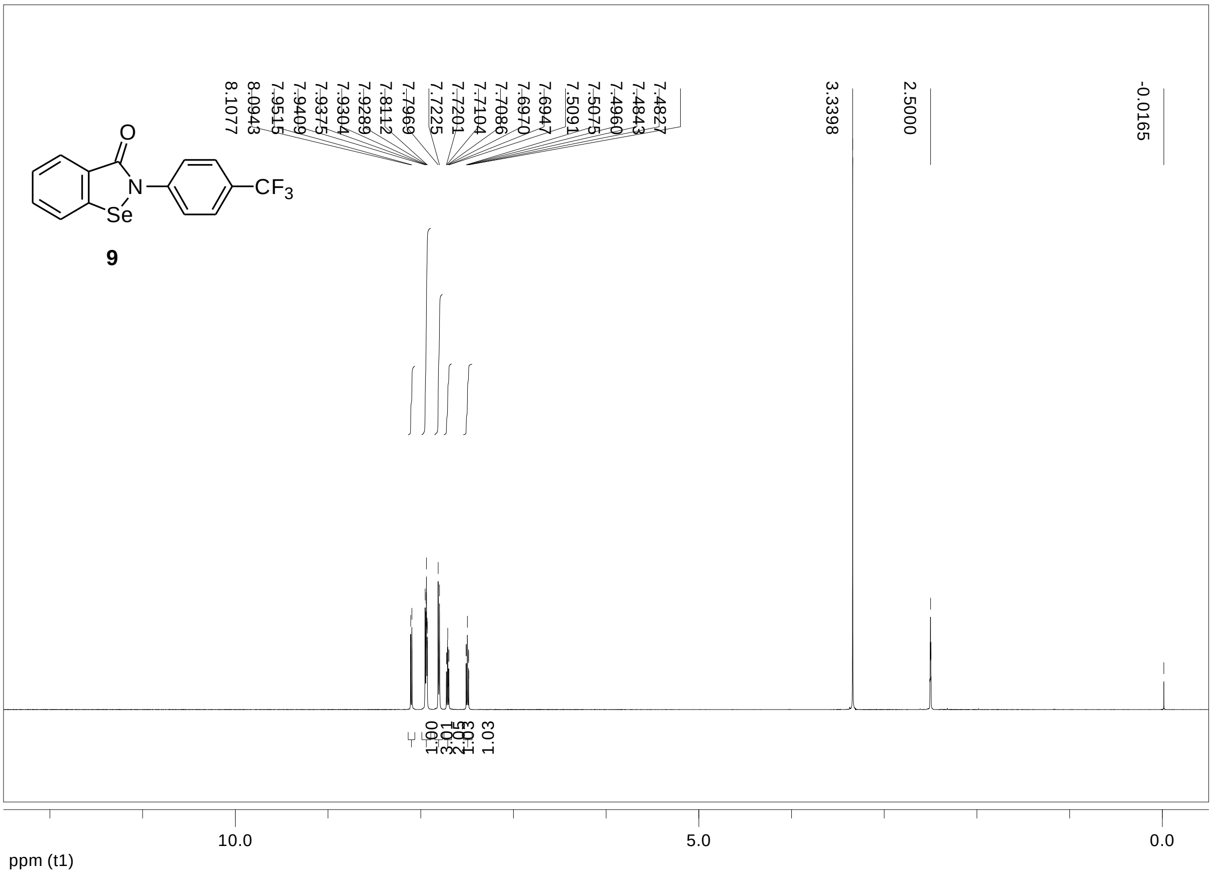

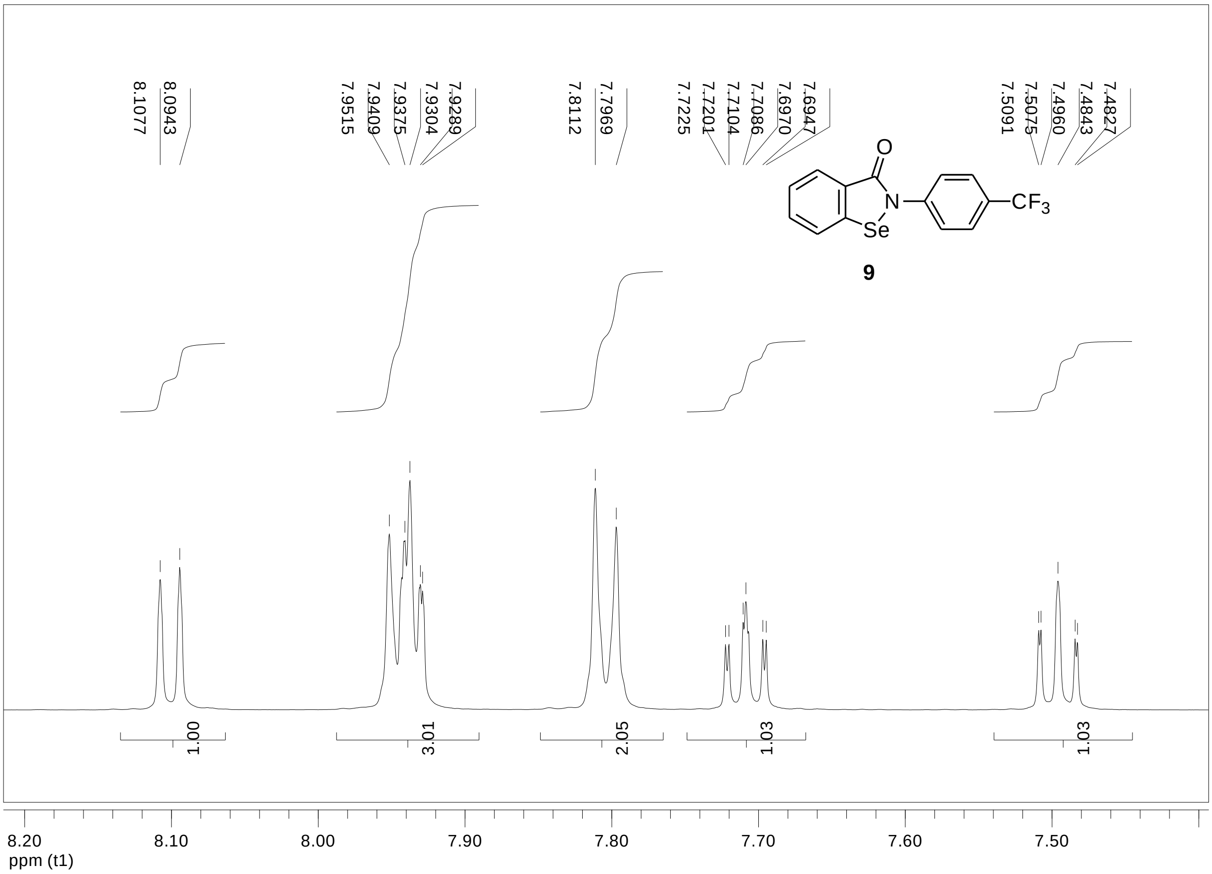

Fig. S47. ^{1}H-NMR (600.6 MHz, DMSO-d_6) spectrum of compound **9**

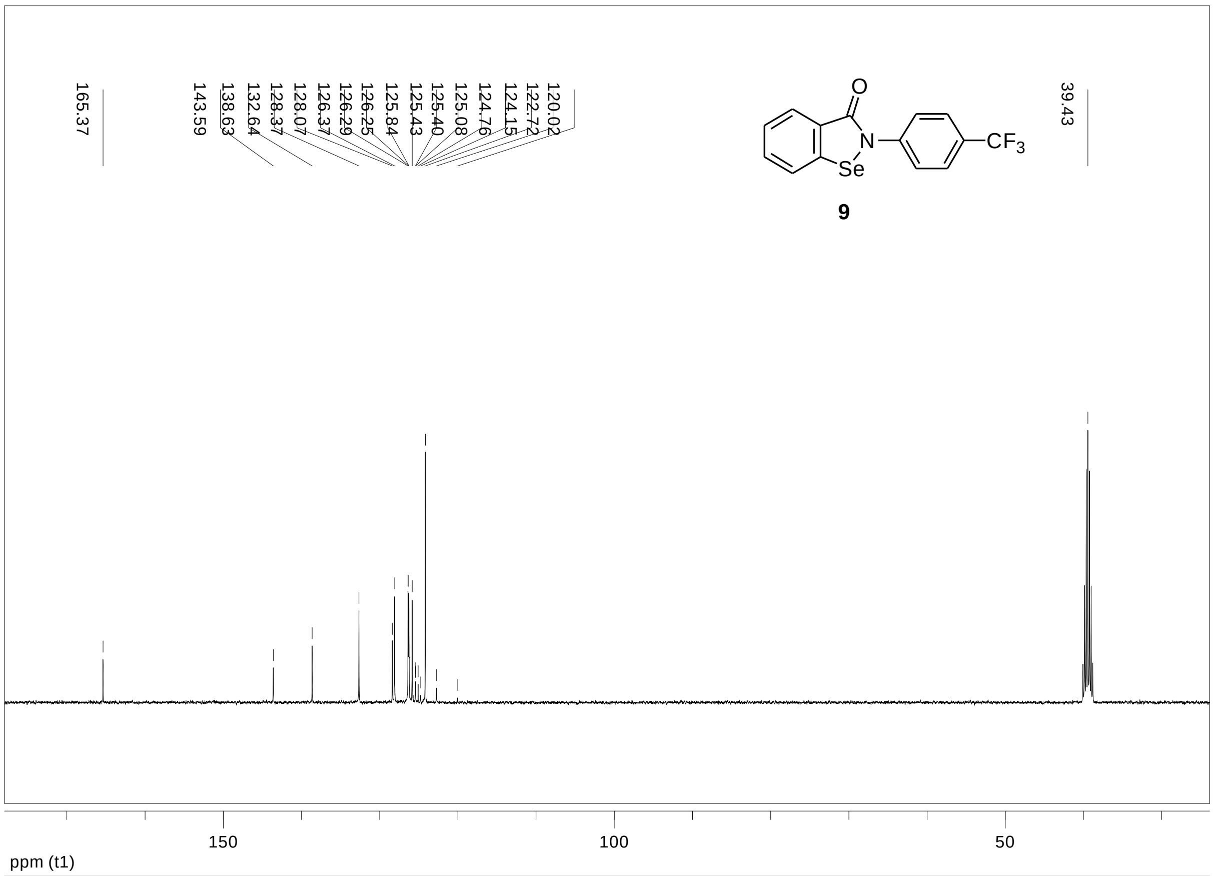

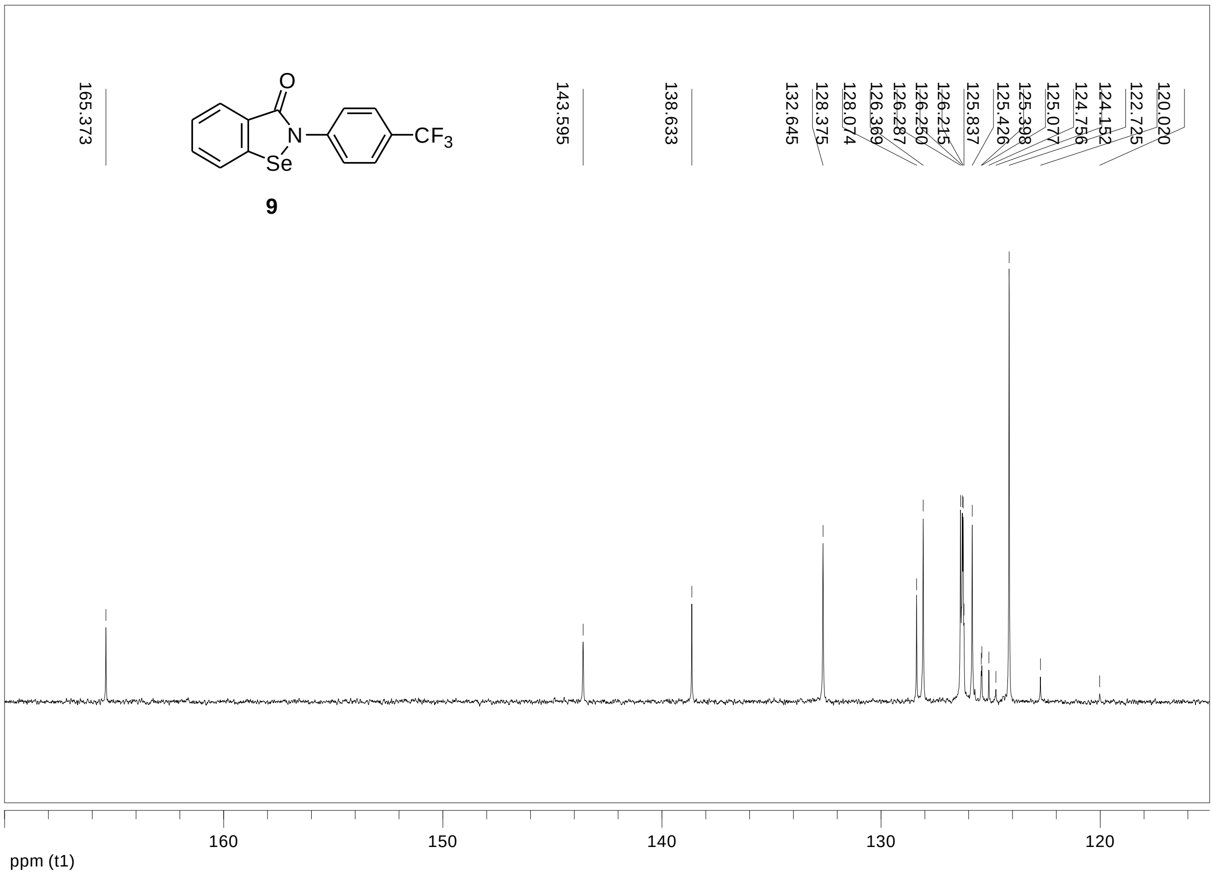

Fig. S48. ^{13}C-NMR (100.5 MHz, DMSO-d_6) spectrum of compound **9**

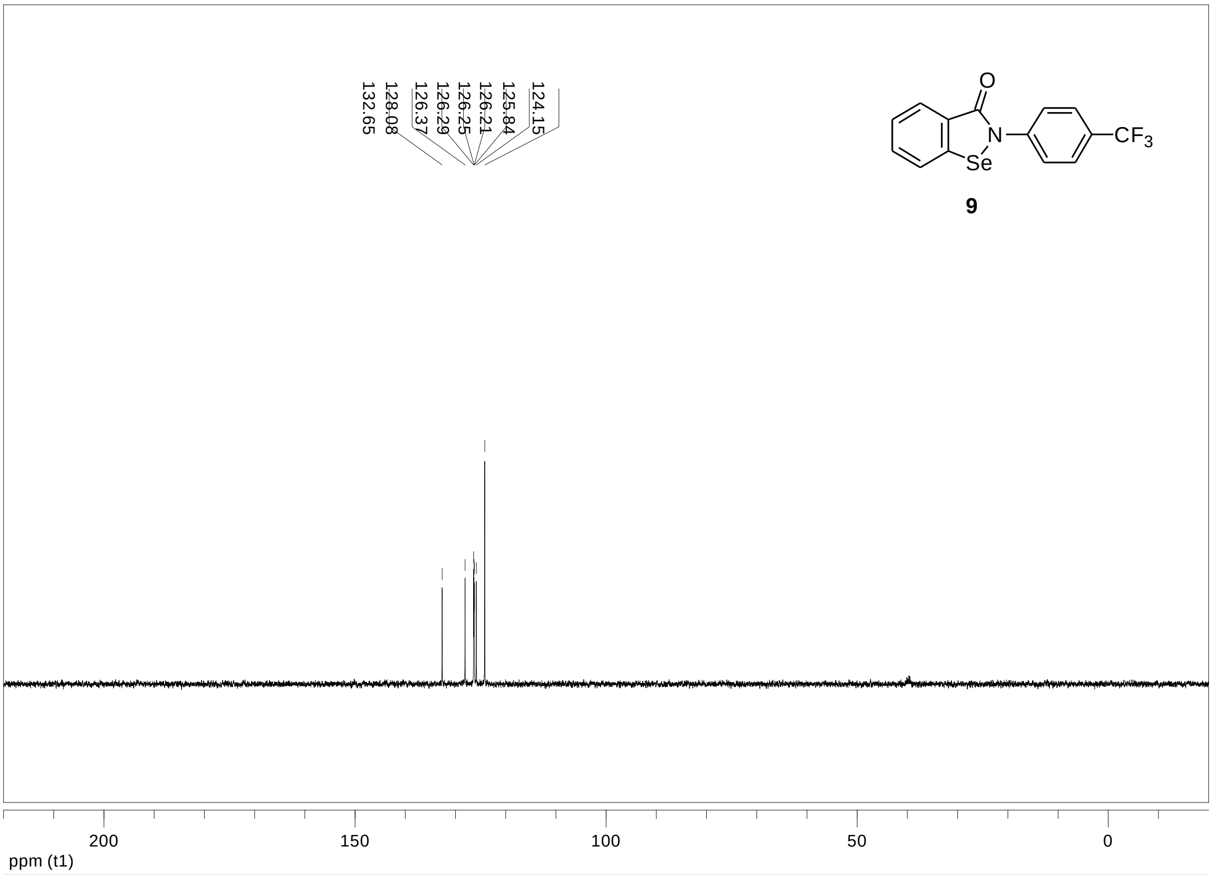

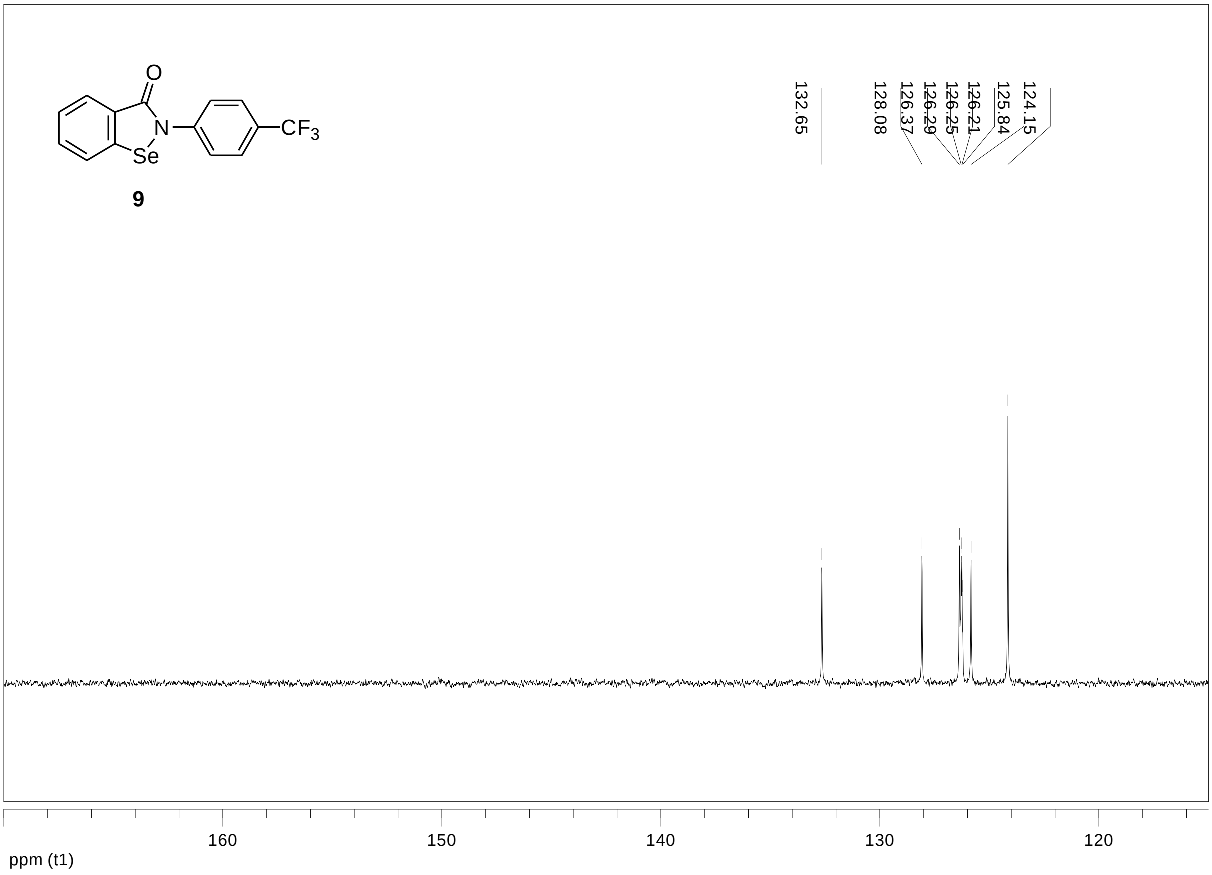

Fig. S49. ^{13}C-NMR (100.5 MHz, DMSO-d_6) dept-135 experiment of compound **9**

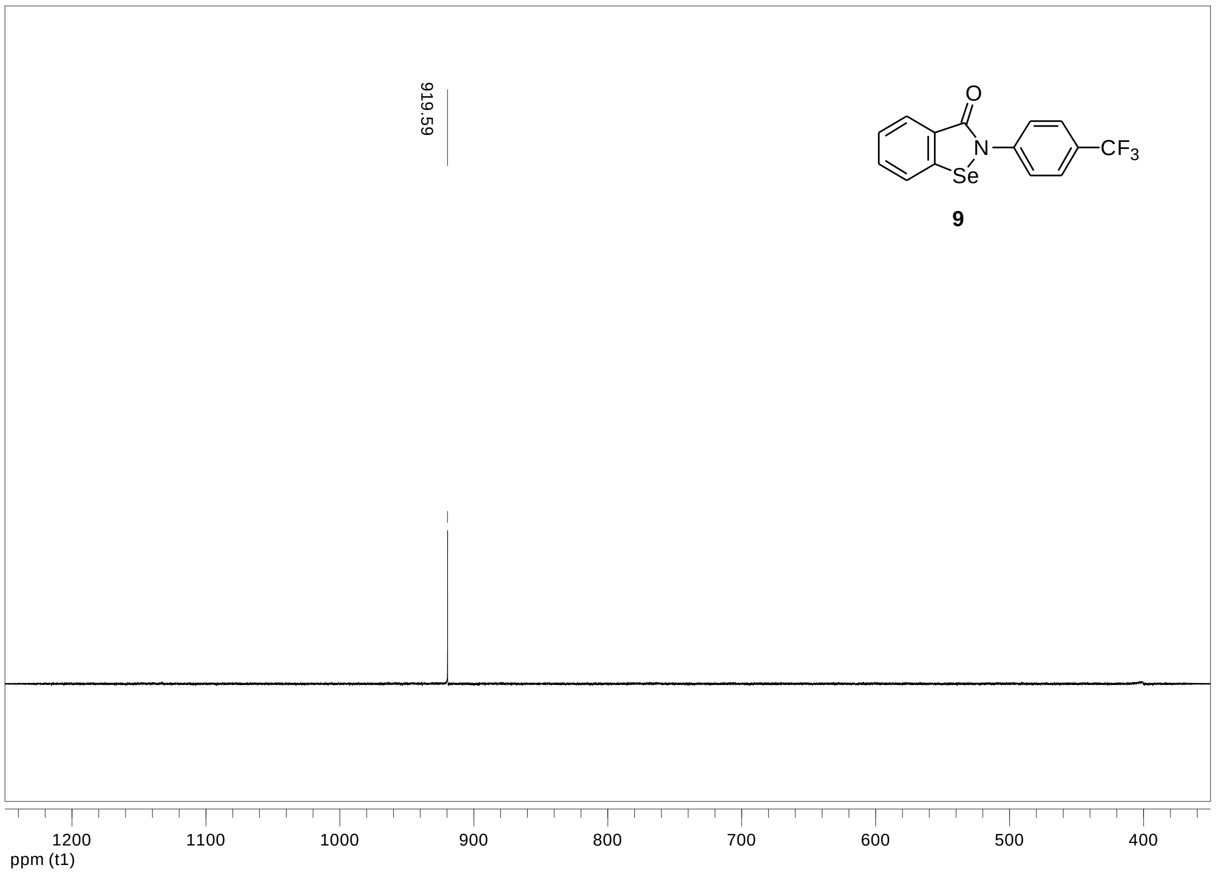

Fig. S50. ^{77}Se-NMR (76.24 MHz, DMSO-d_6) spectrum of compound **9**

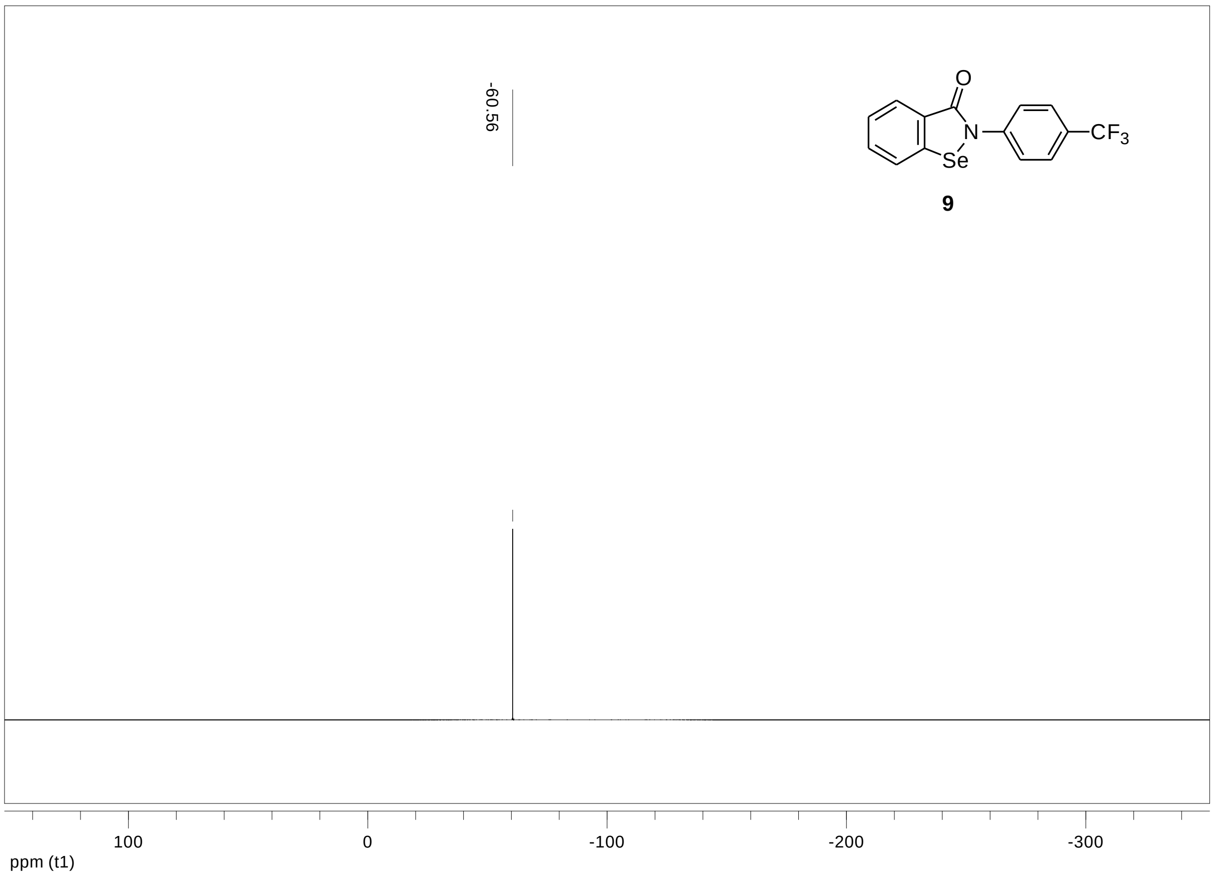

Fig. S51. ^{19}F-NMR (376.2 MHz, DMSO-d_6) spectrum of compound **9**

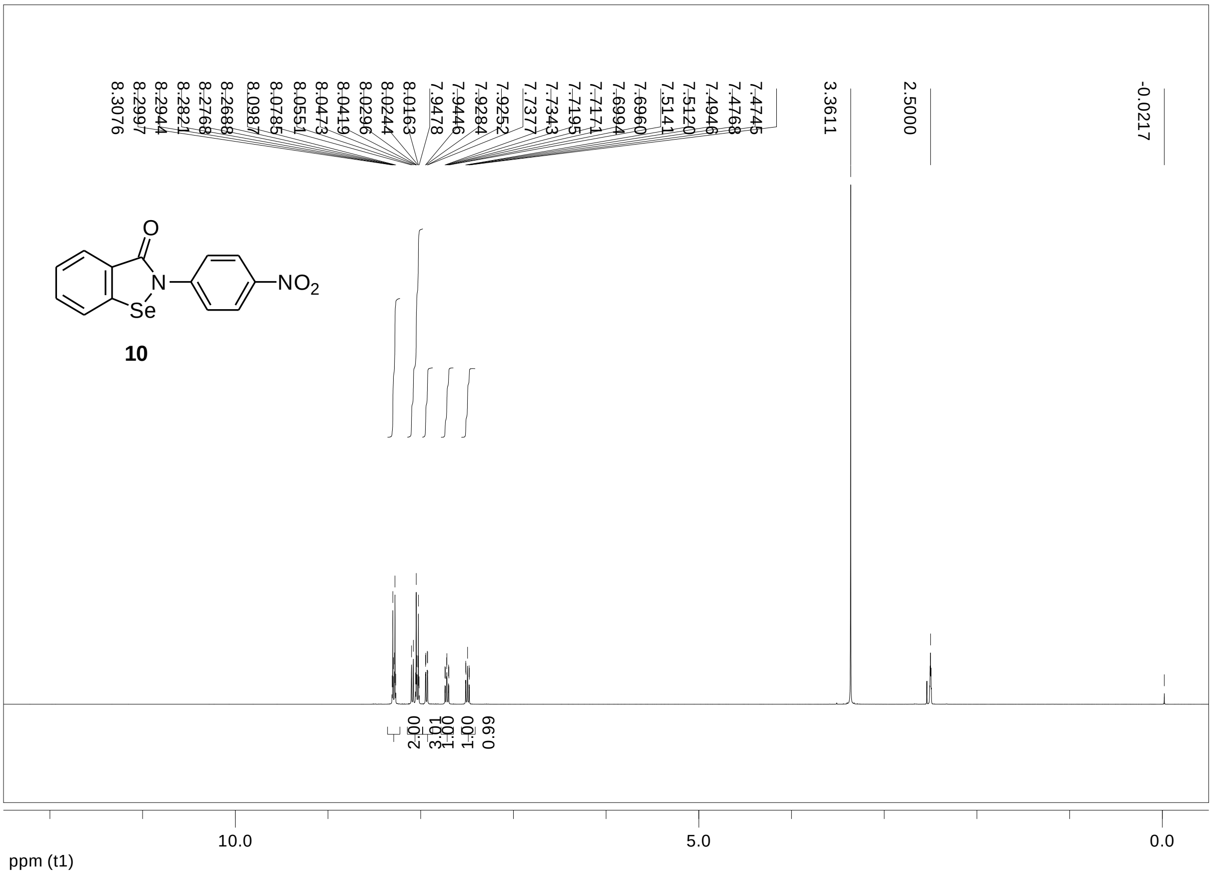

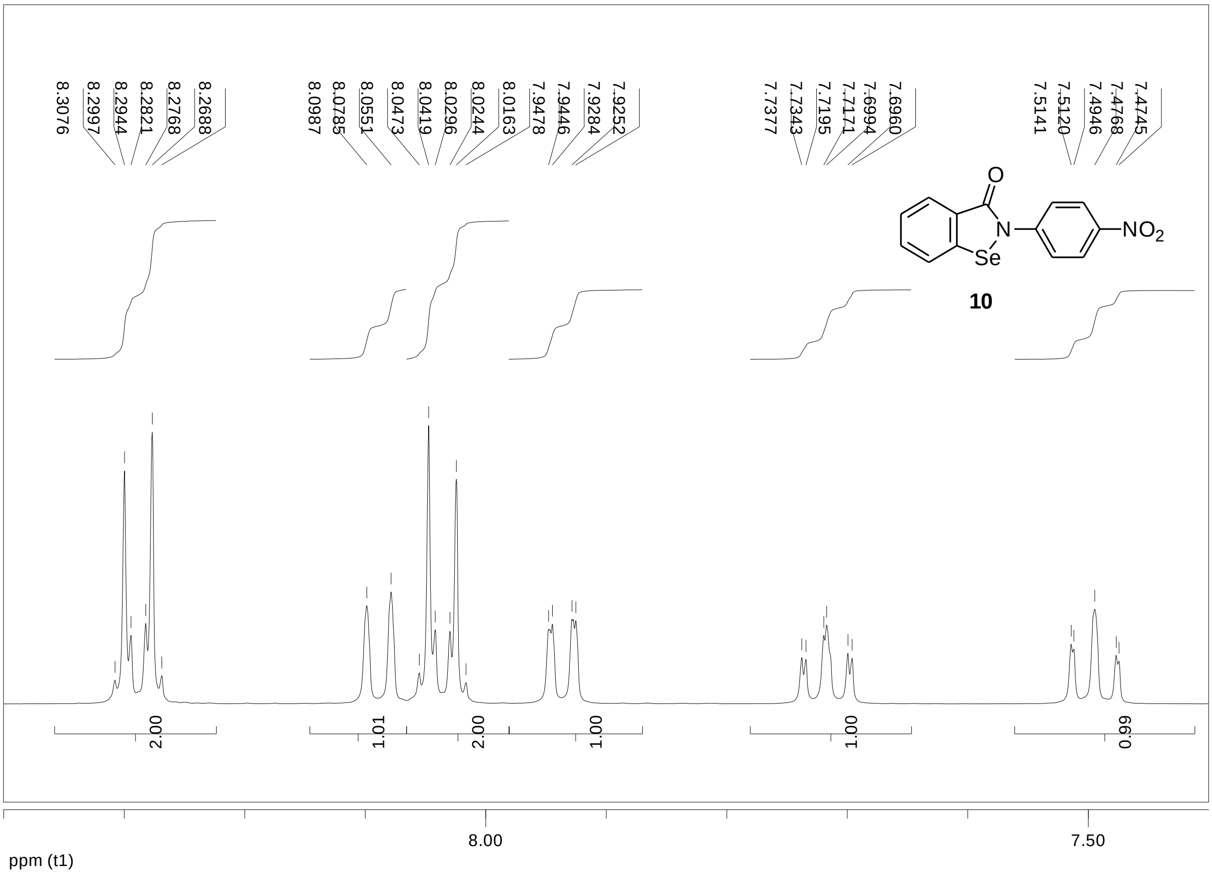

Fig. S52. ^{1}H-NMR (399.78 MHz, DMSO-d_6) spectrum of compound **10**

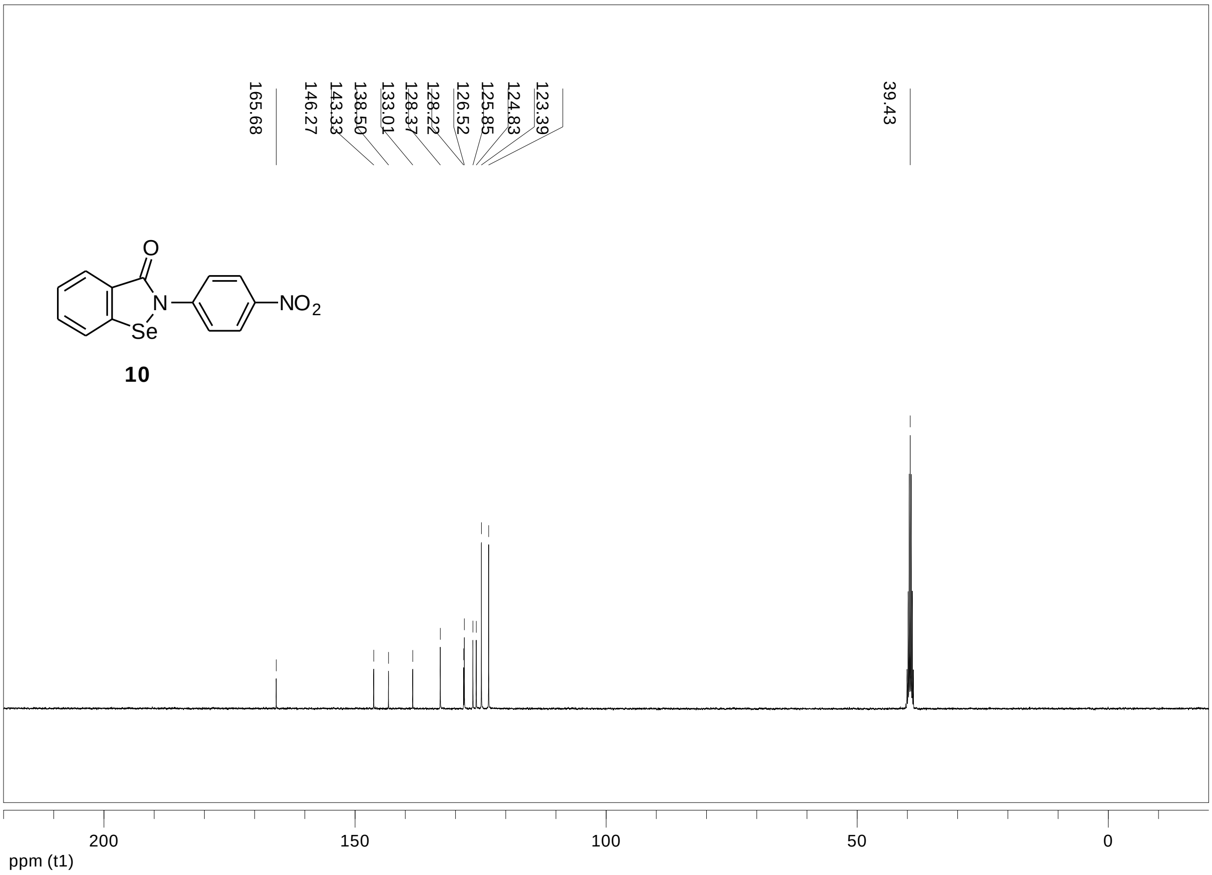

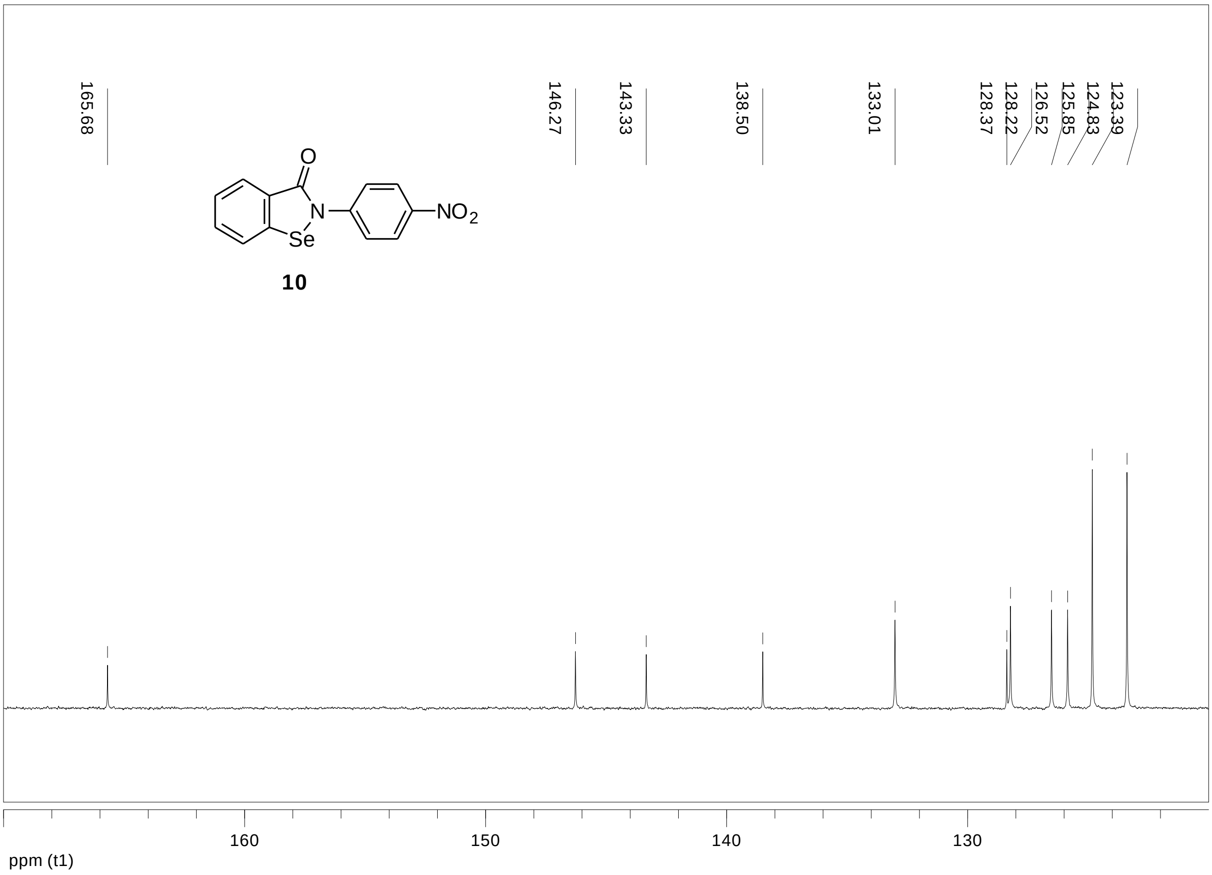

Fig. S53. ^{13}C-NMR (100.5 MHz, DMSO-d_6) spectrum of compound **10**

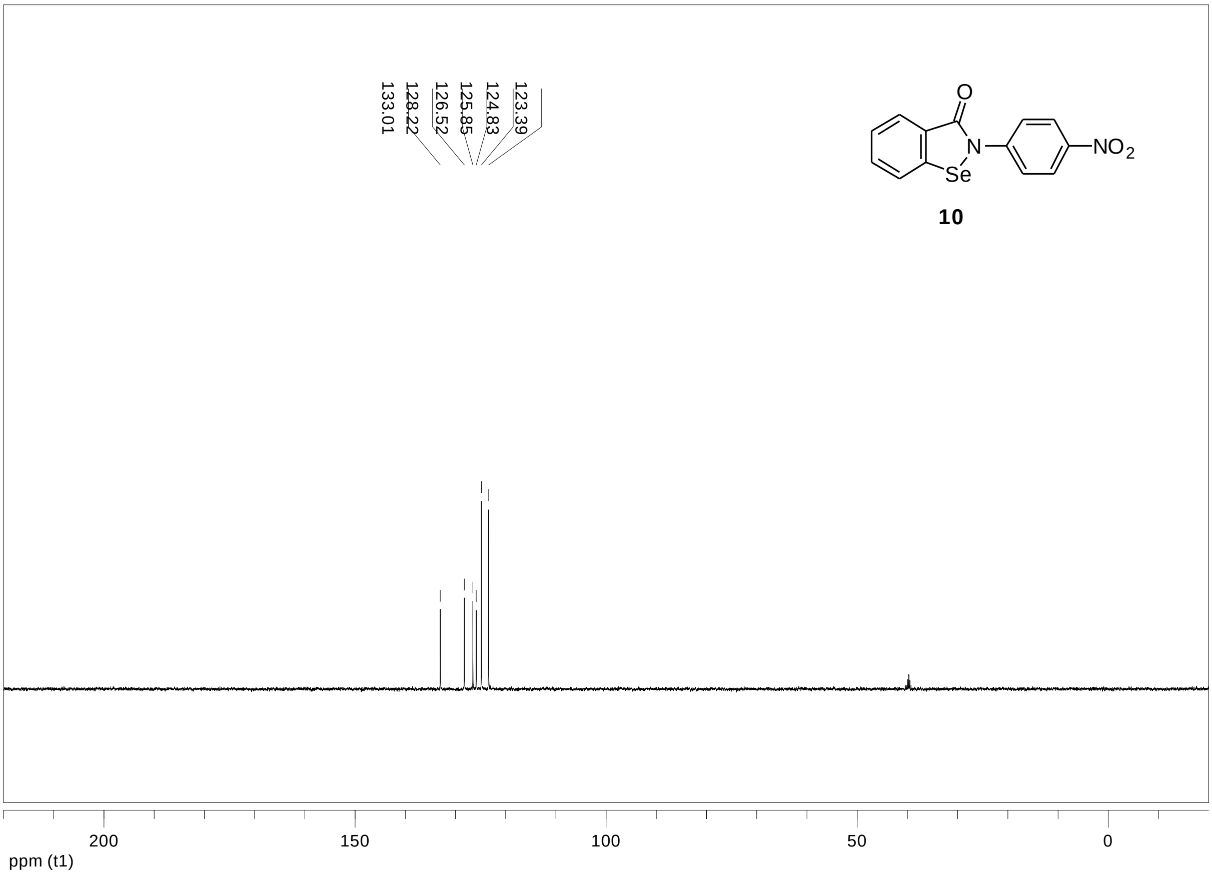

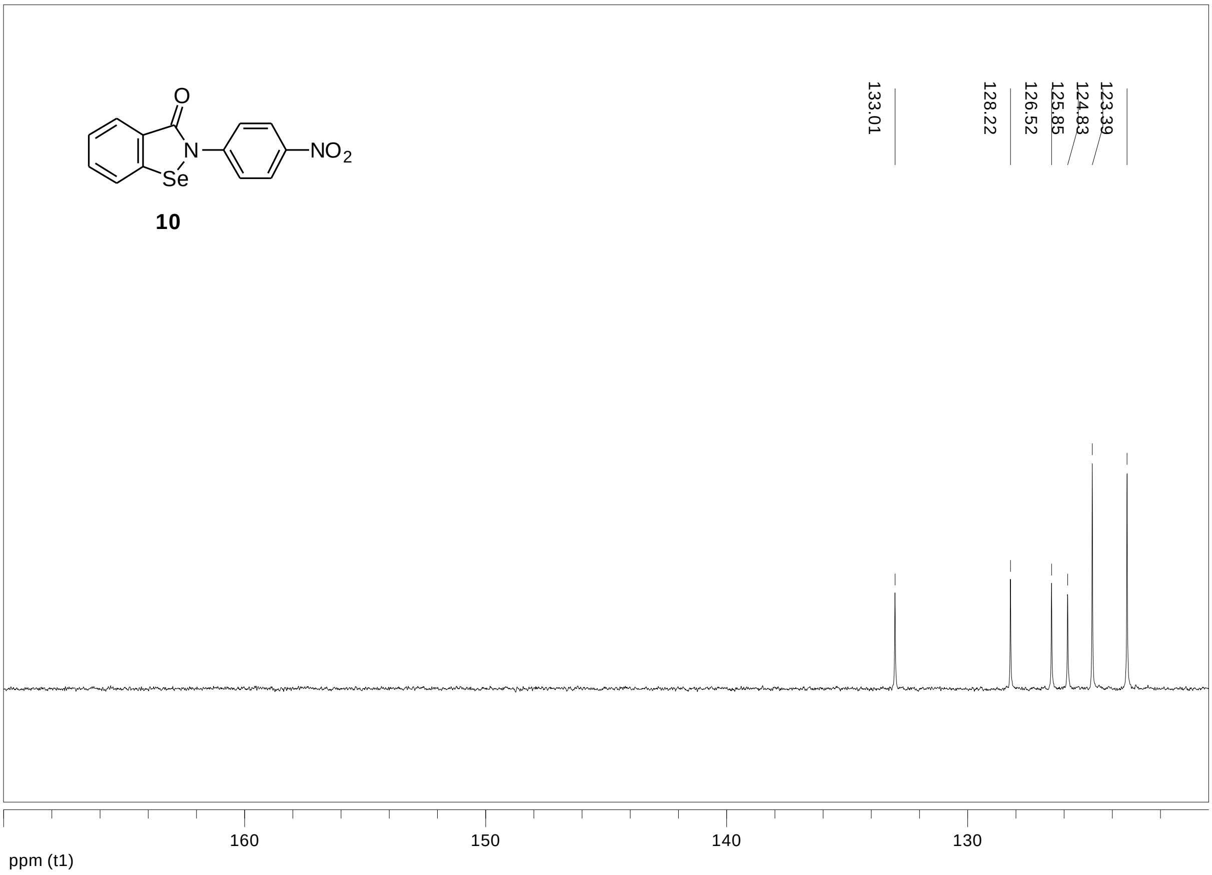

Fig. S54. ^{13}C-NMR (100.5 MHz, DMSO-d_6) dept-135 experiment of compound **10**

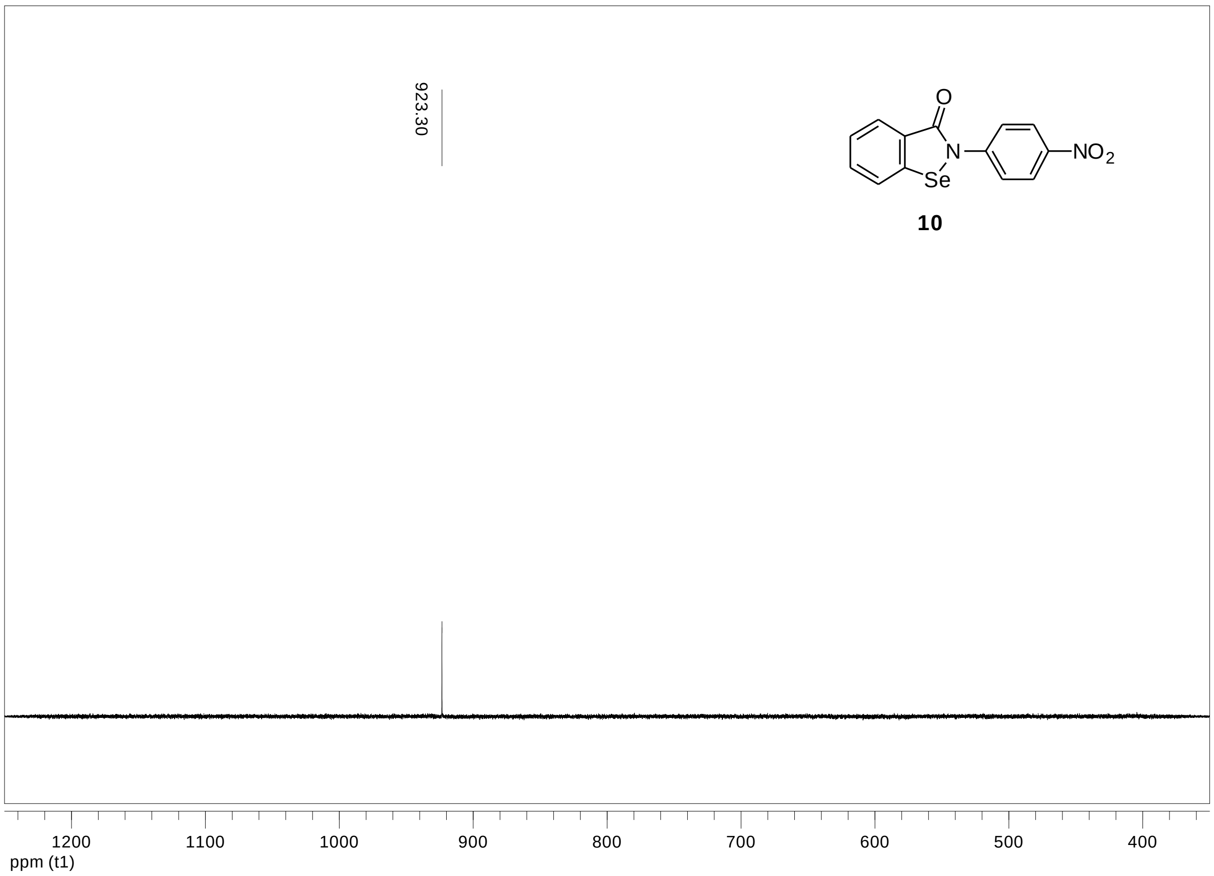

Fig. S55. ^{77}Se-NMR (76.24 MHz, DMSO-d_6) spectrum of compound **10**

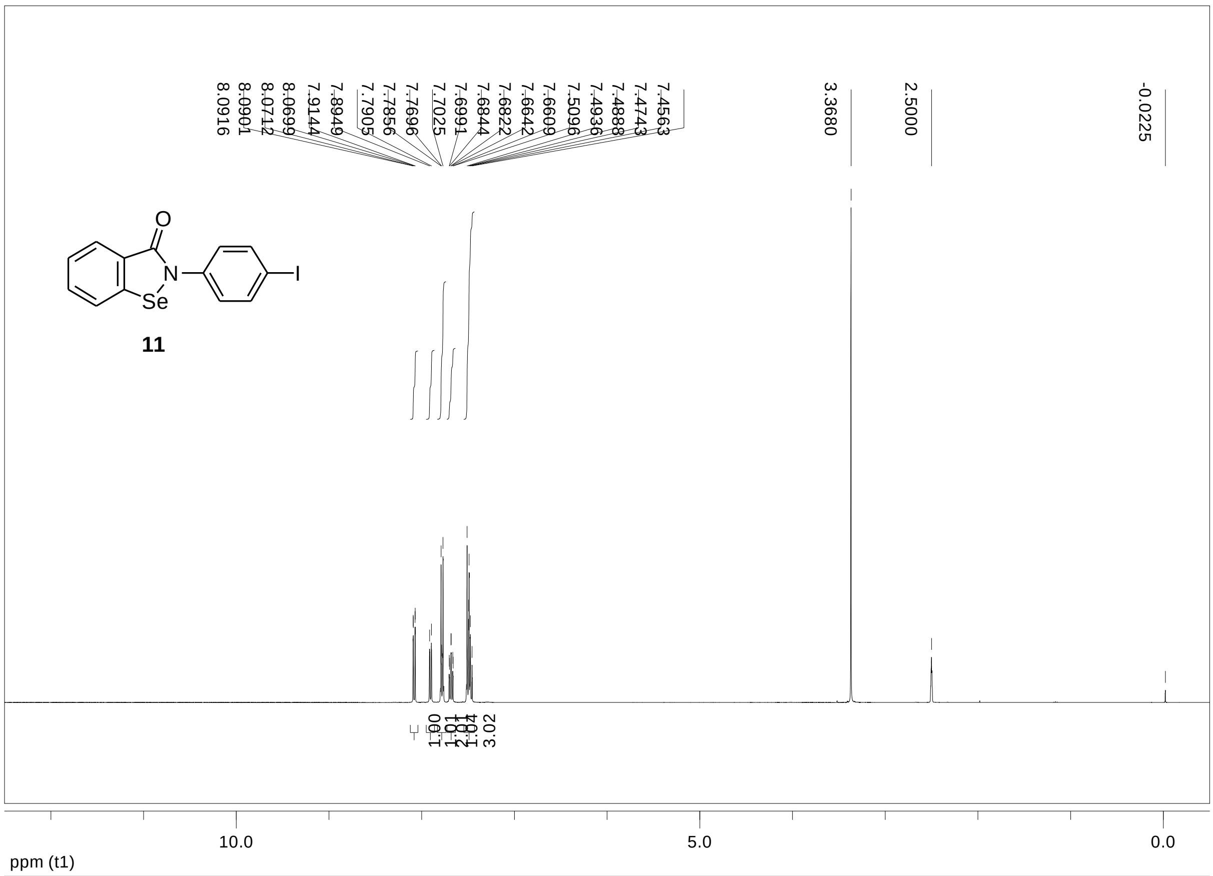

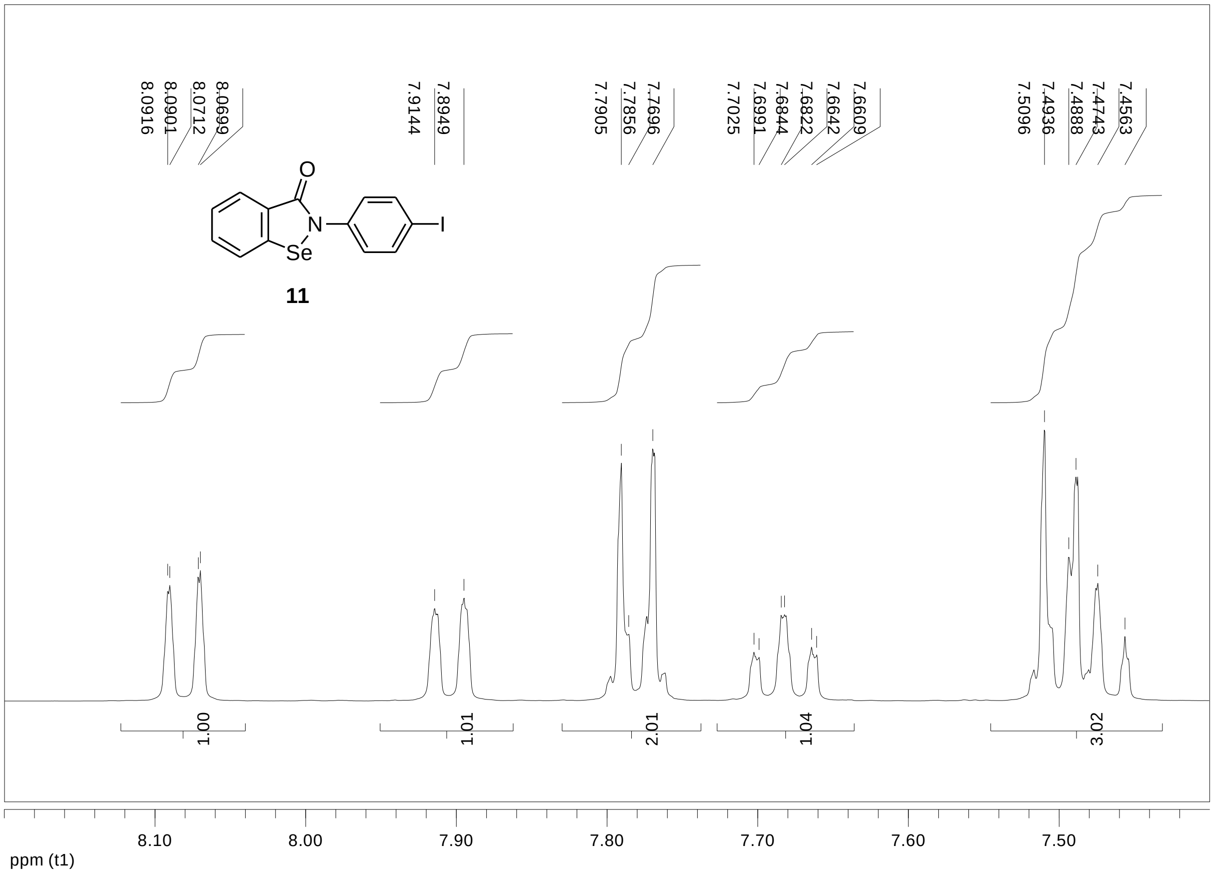

Fig. S56. ^{1}H-NMR (399.8 MHz, DMSO-d_6) spectrum of compound **11**

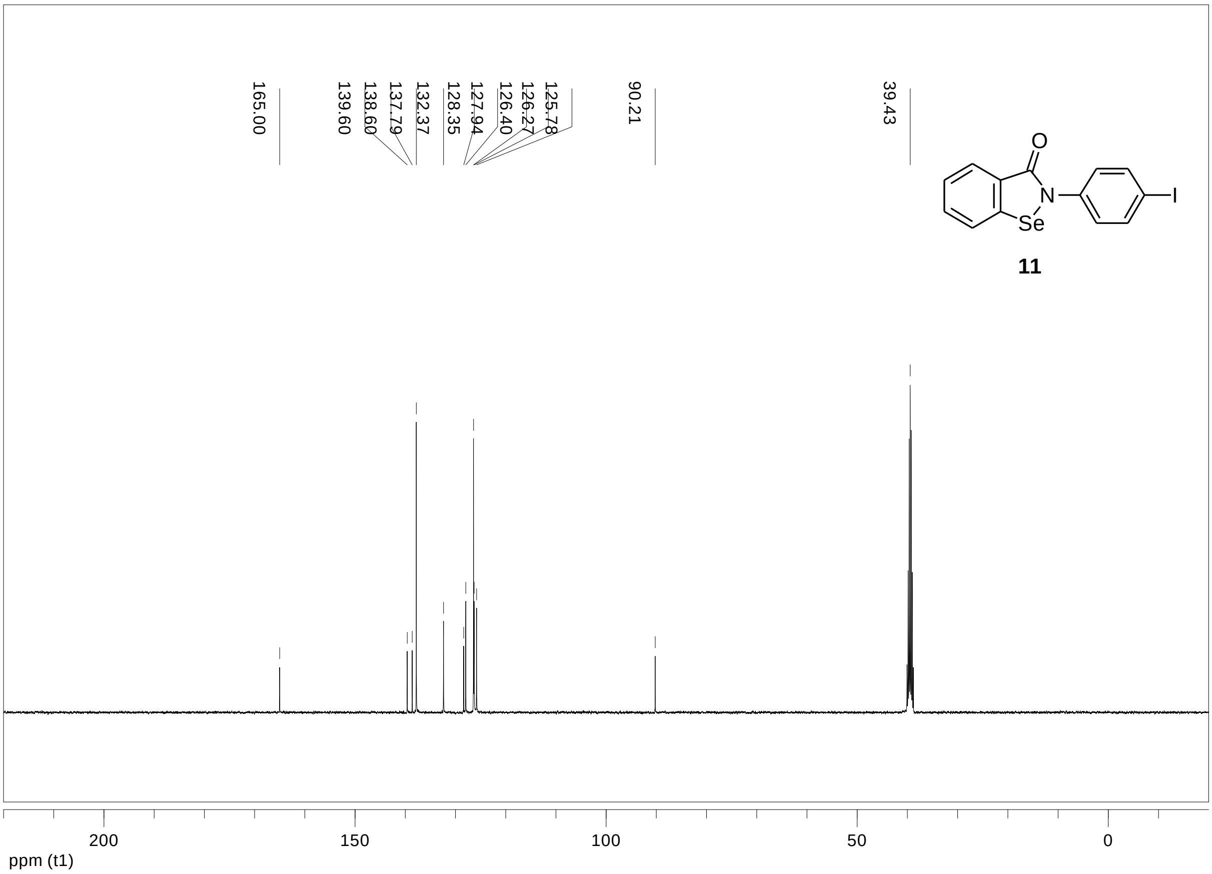

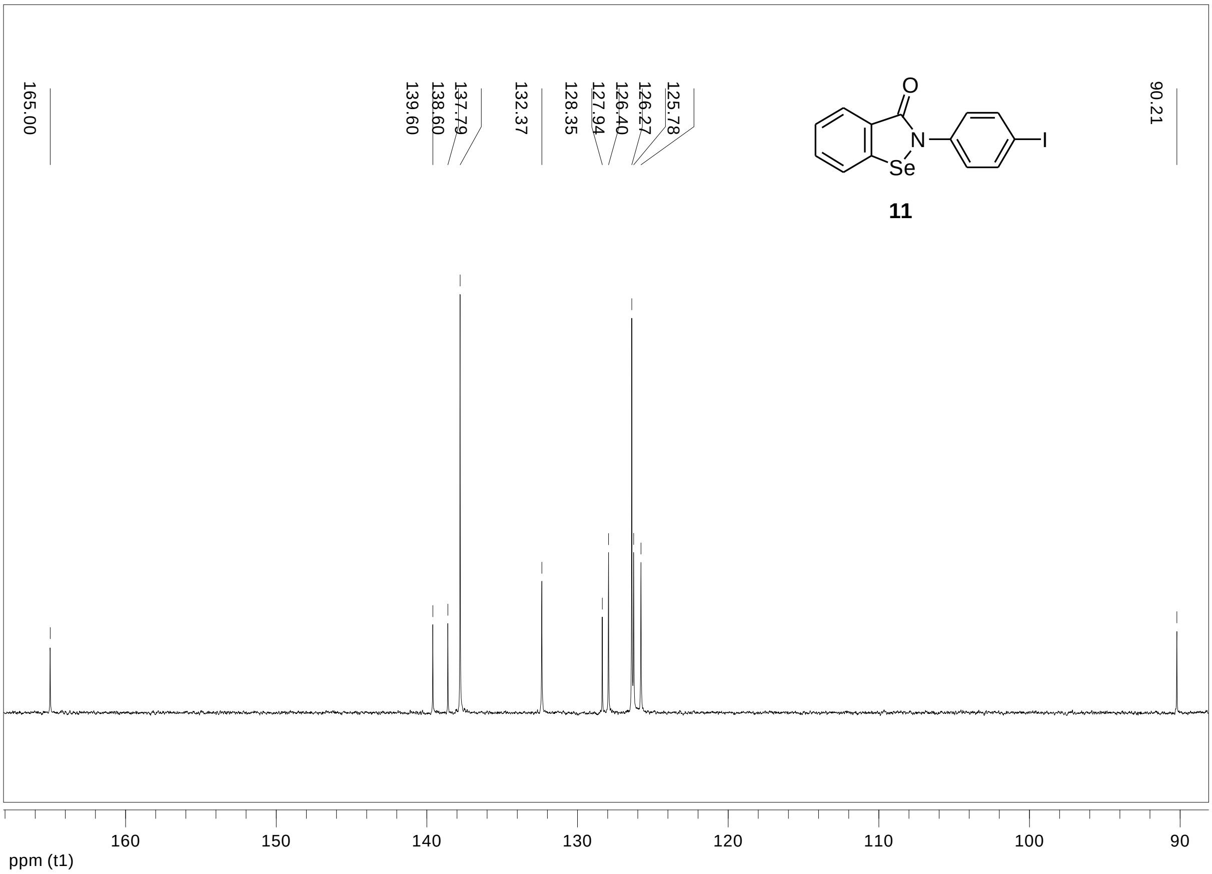

Fig. S57. ^{13}C-NMR (100.5 MHz, DMSO-d_6) spectrum of compound **11**

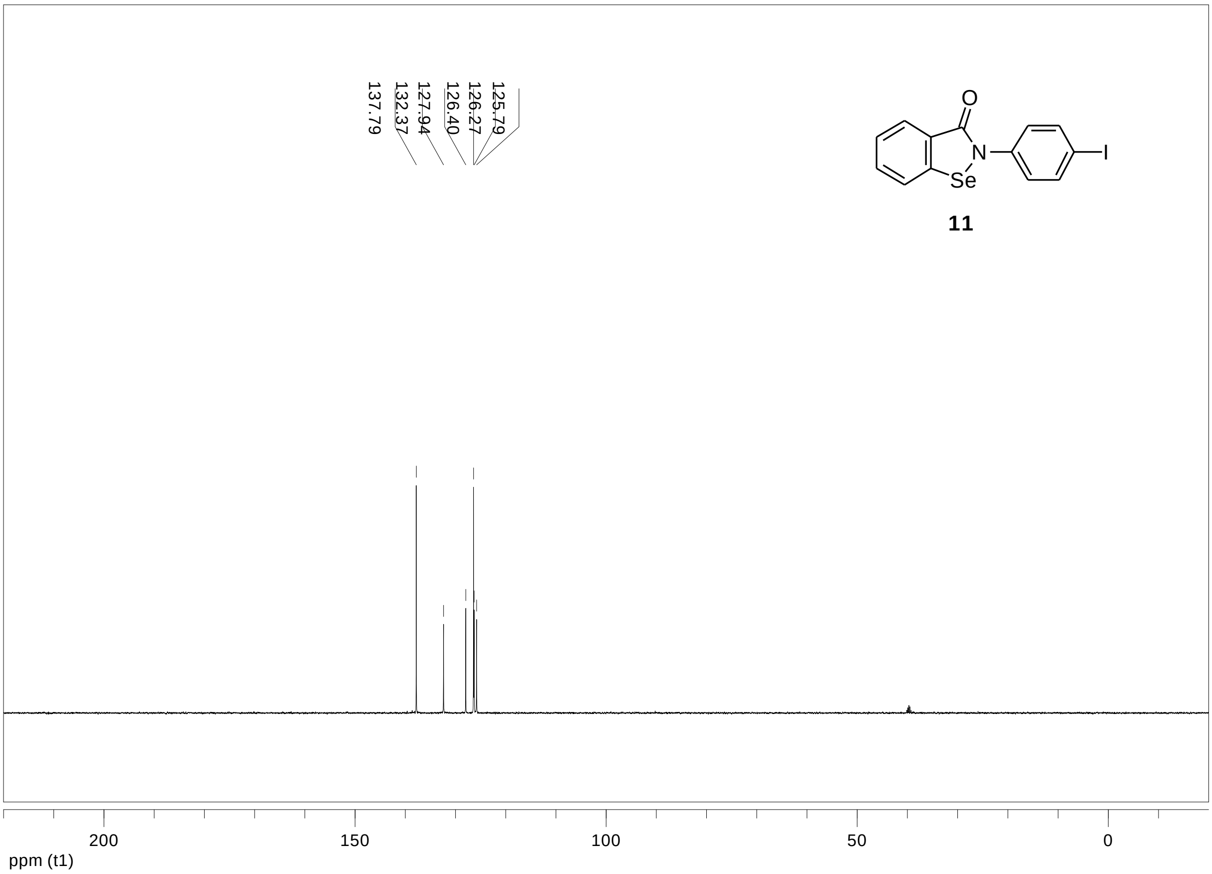

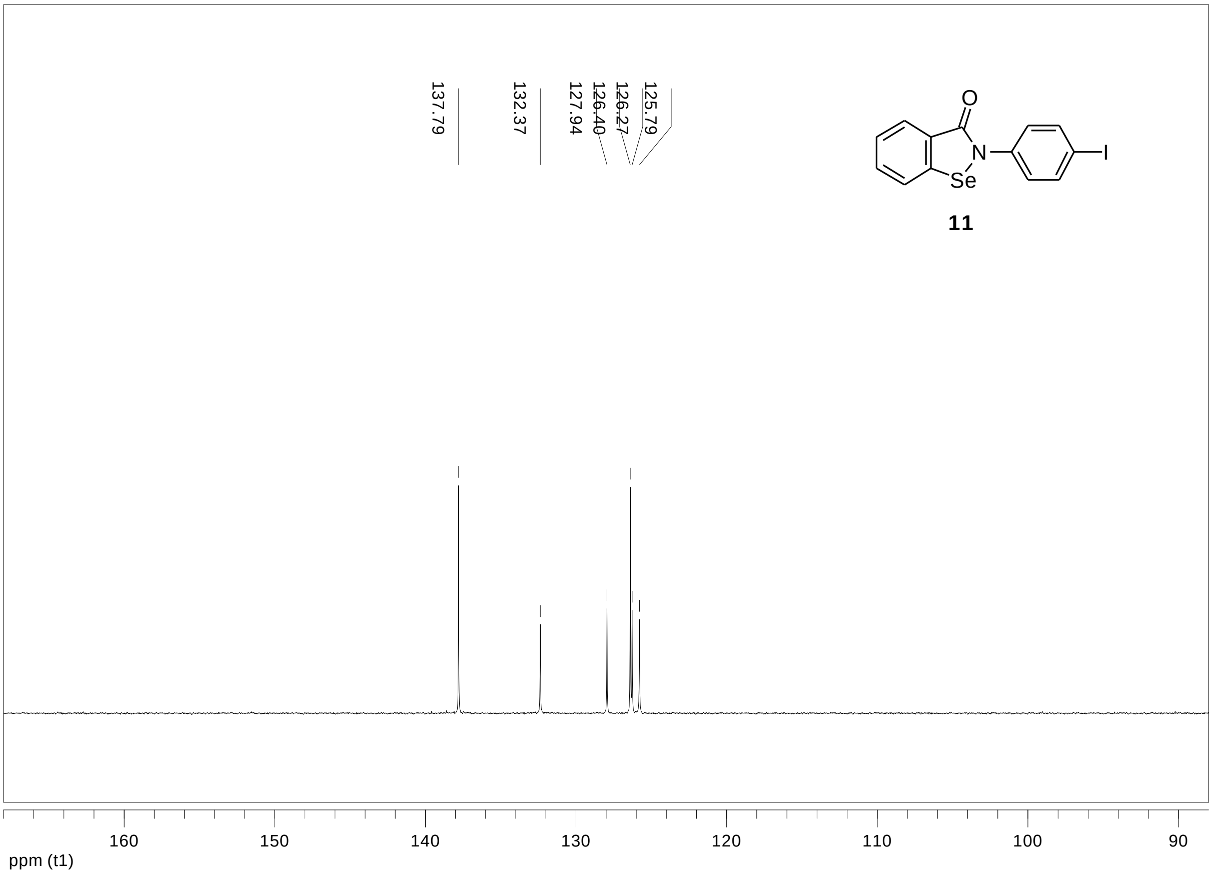

Fig. S58. ^{13}C-NMR (100.5 MHz, DMSO-d_6) dept-135 experiment of compound **11**

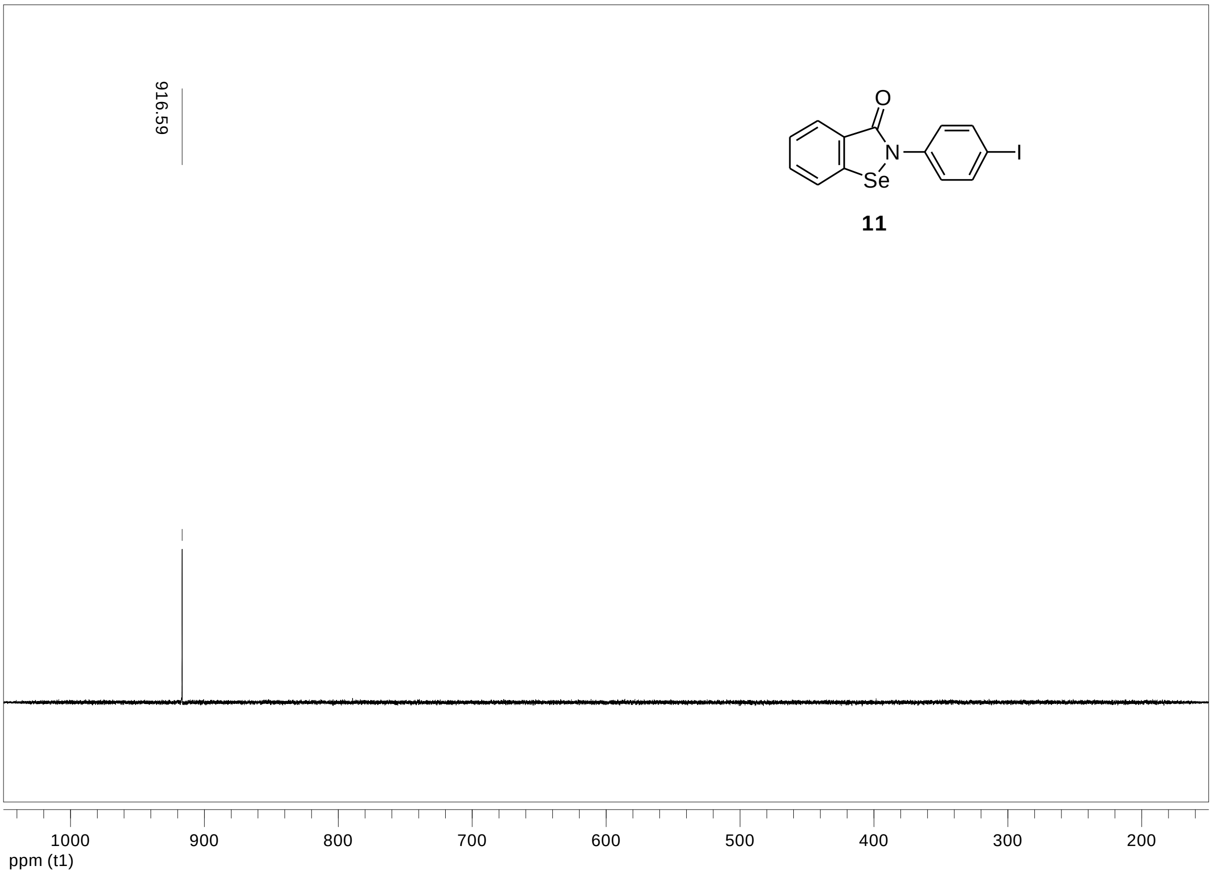

Fig. S59. ^{77}Se-NMR (76.24 MHz, DMSO-d_6) spectrum of compound **11**

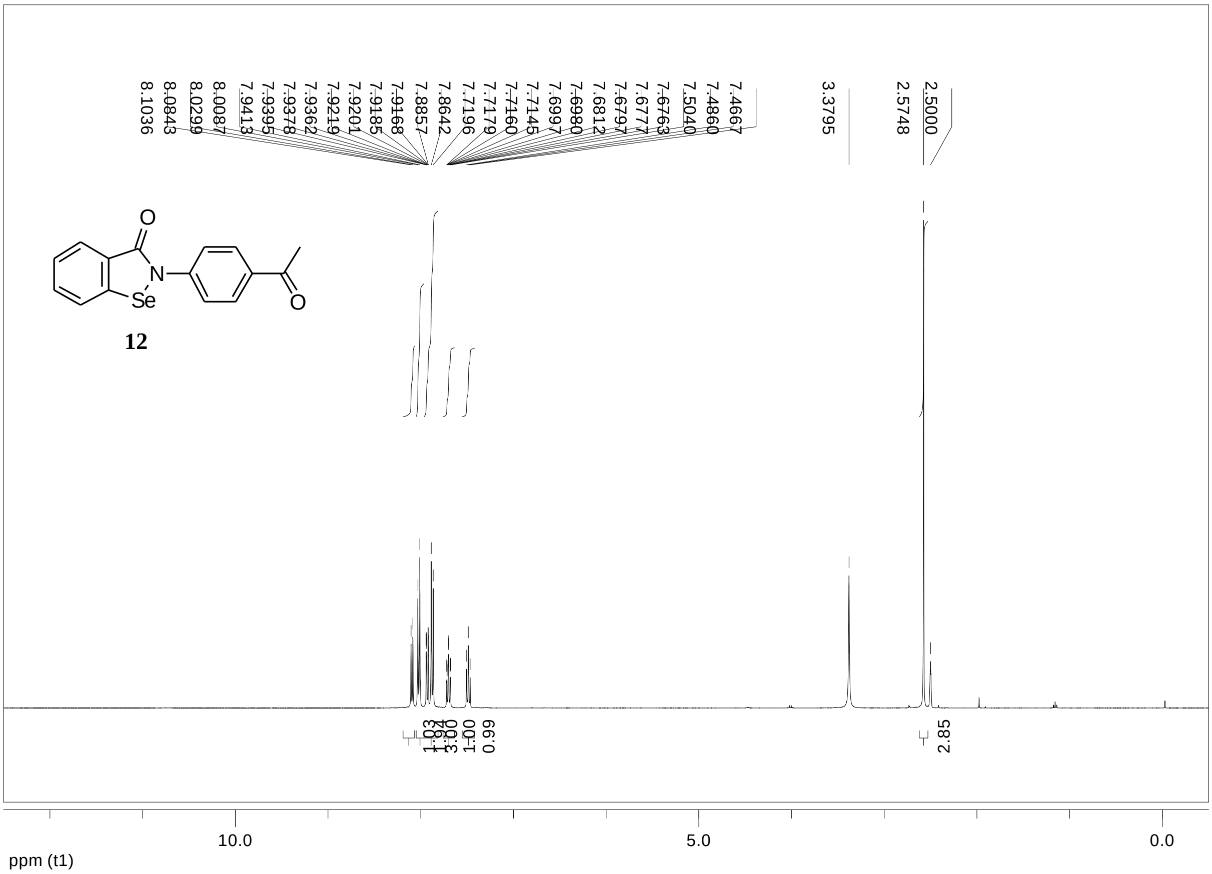

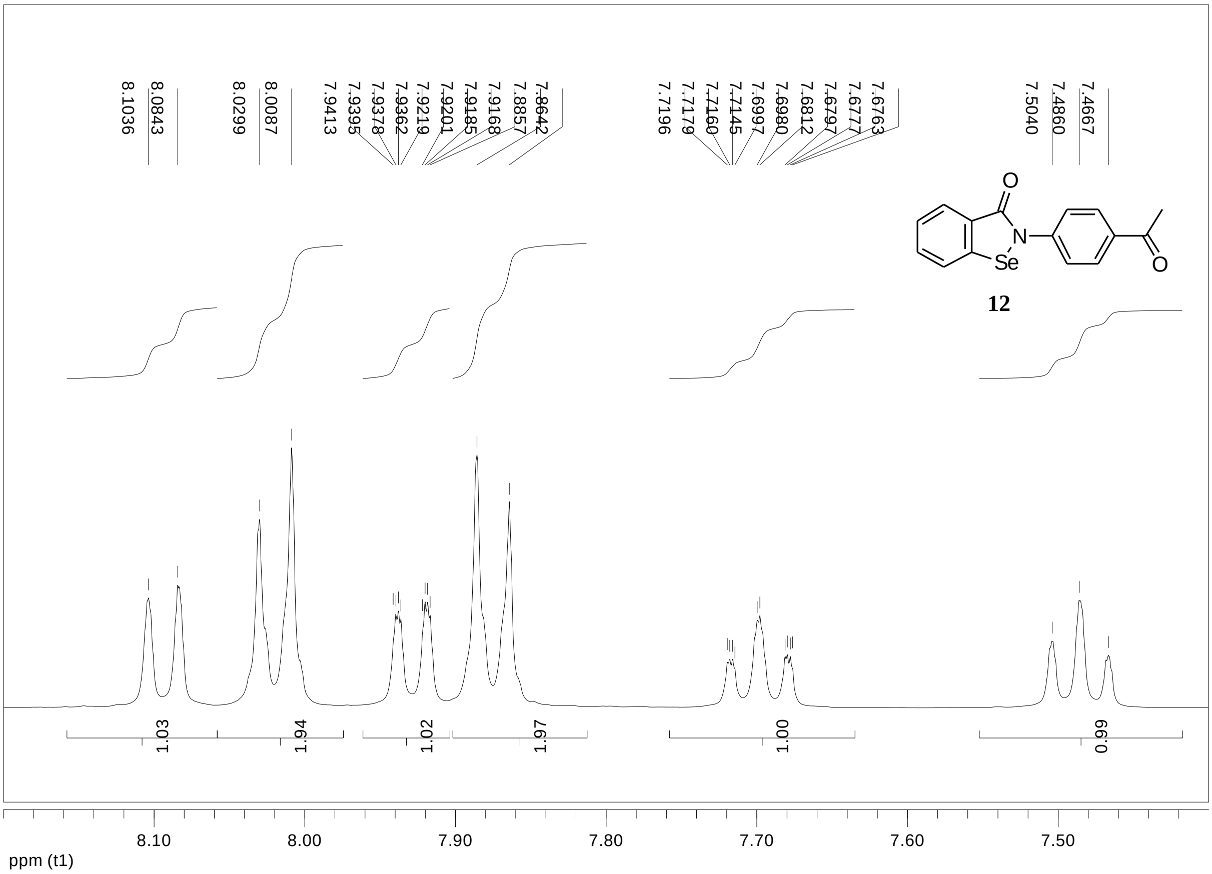

Fig. S60. ^{1}H-NMR (399.8 MHz, DMSO-d_6) spectrum of compound **12**

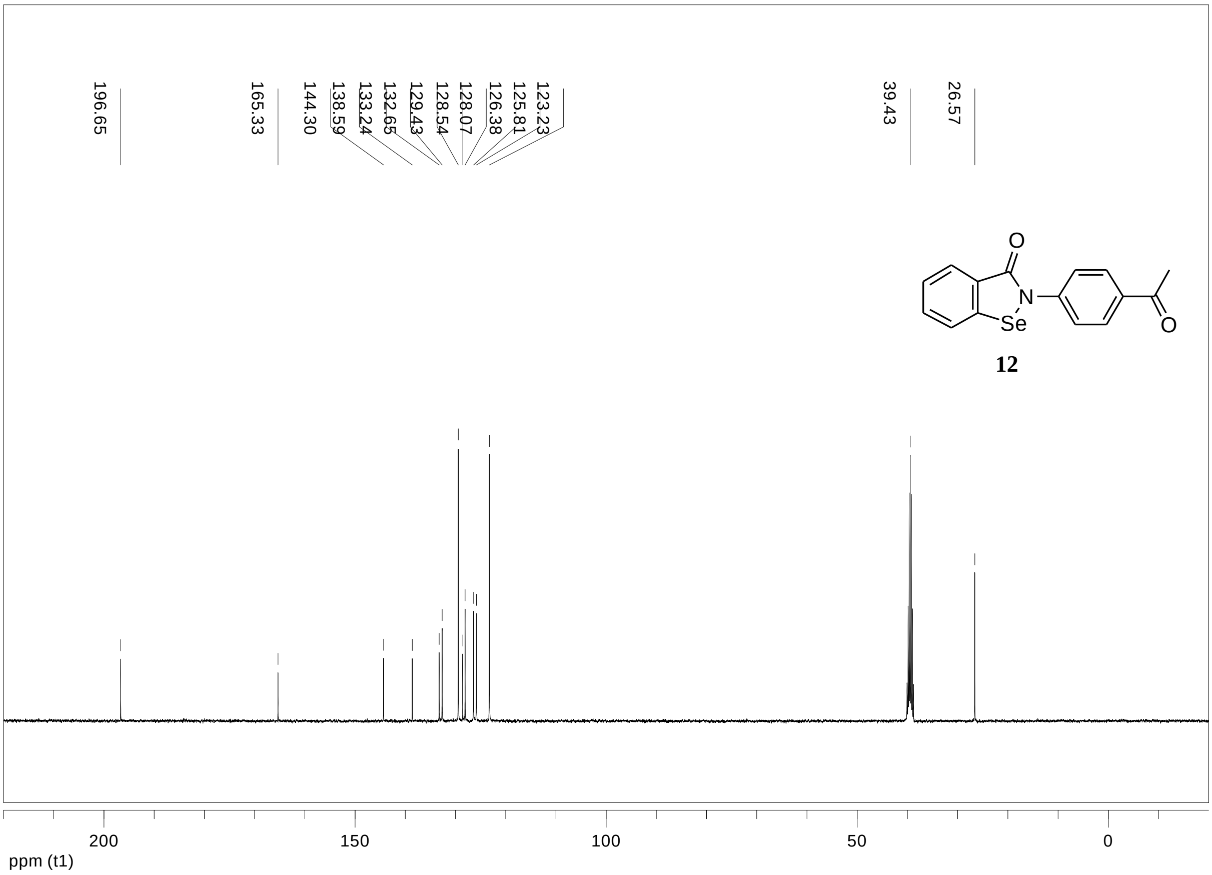

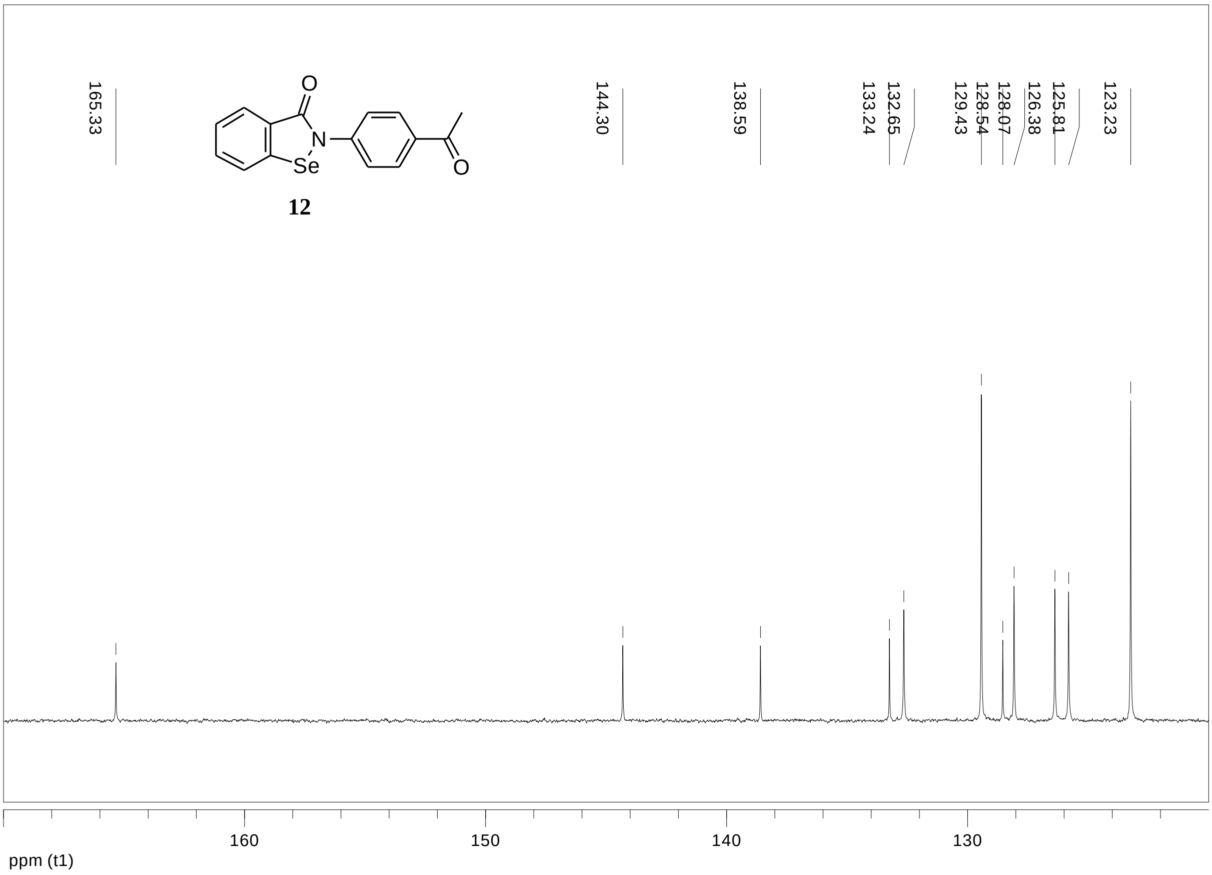

Fig. S61. ^{13}C-NMR (100.5 MHz, DMSO-d_6) spectrum of compound **12**

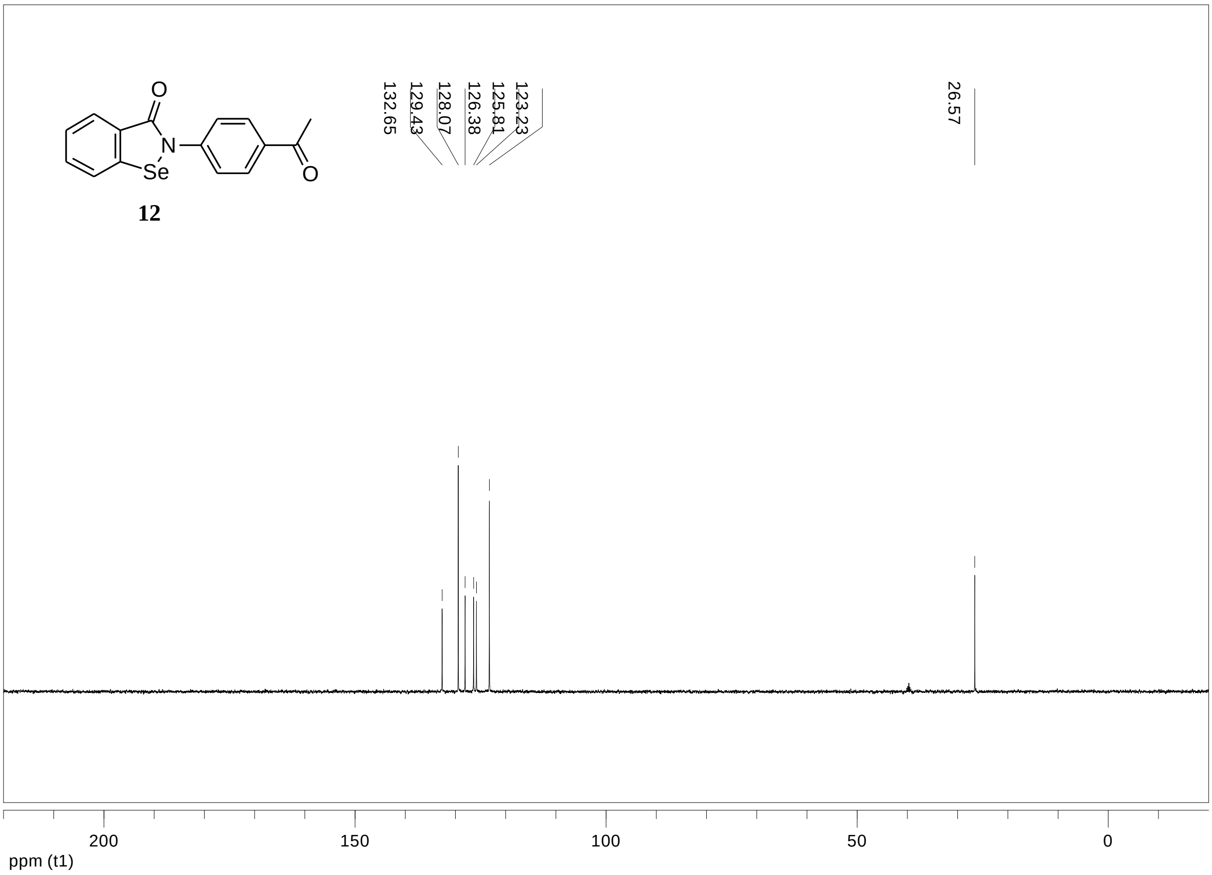

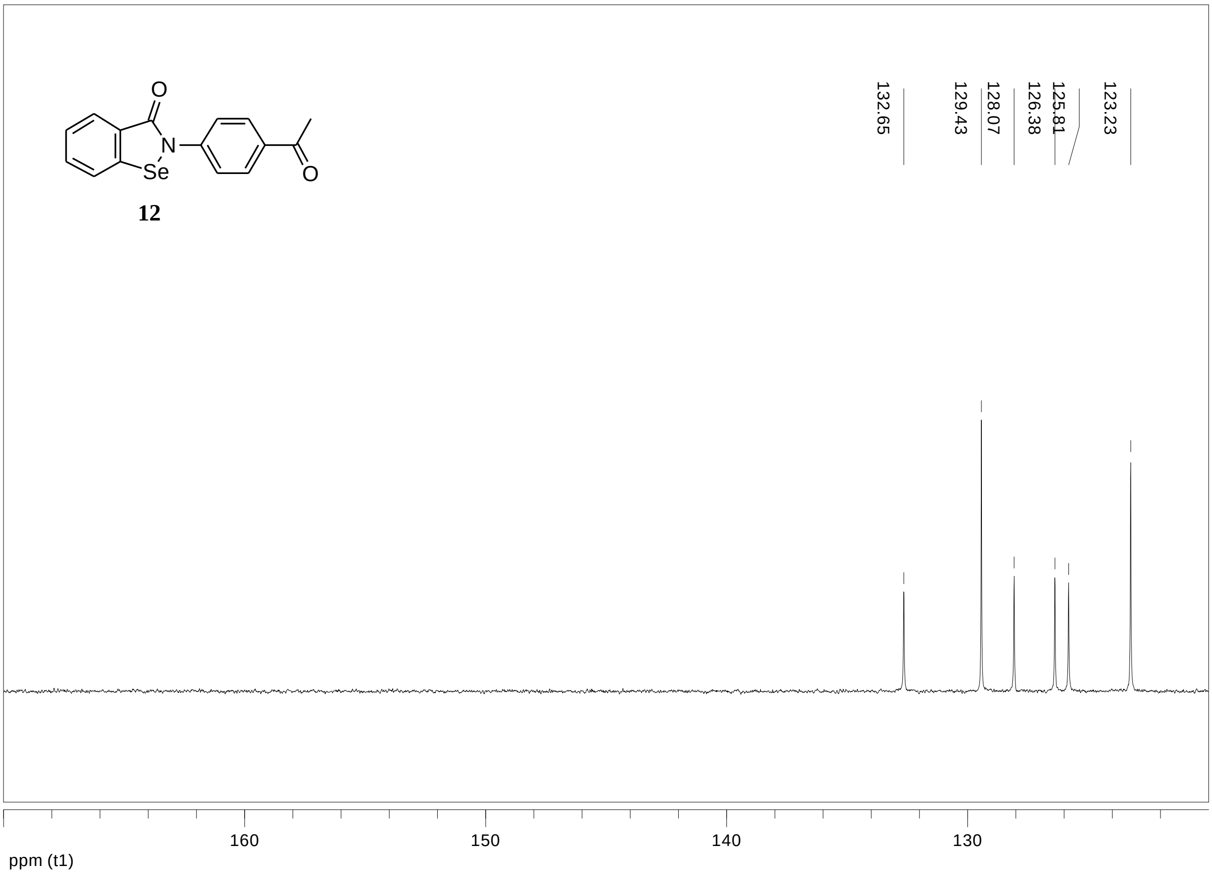

Fig. S62. ^{13}C-NMR (100.52 MHz, DMSO-d_6) dept-135 experiment of compound **12**

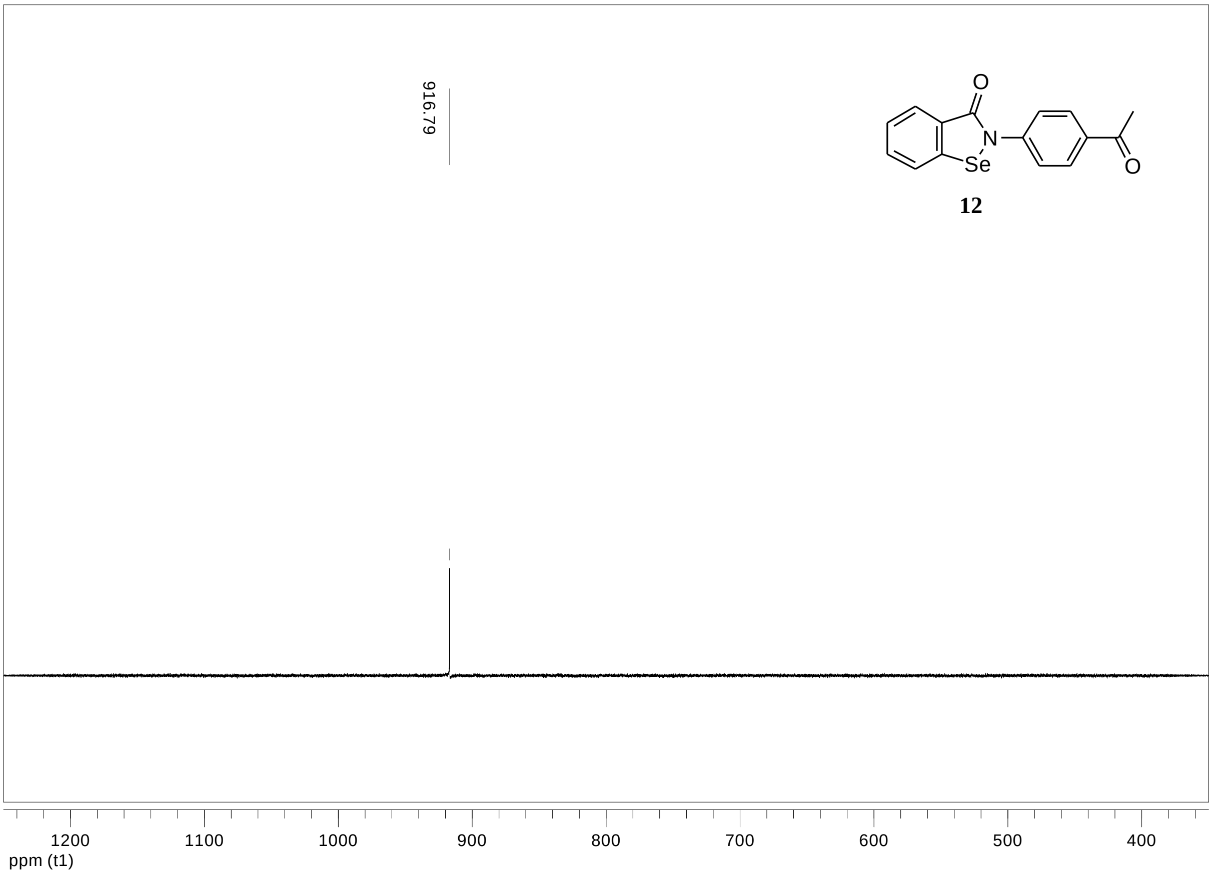

Fig. S63. ^{77}Se-NMR (76.24 MHz, DMSO-d_6) spectrum of compound **12**

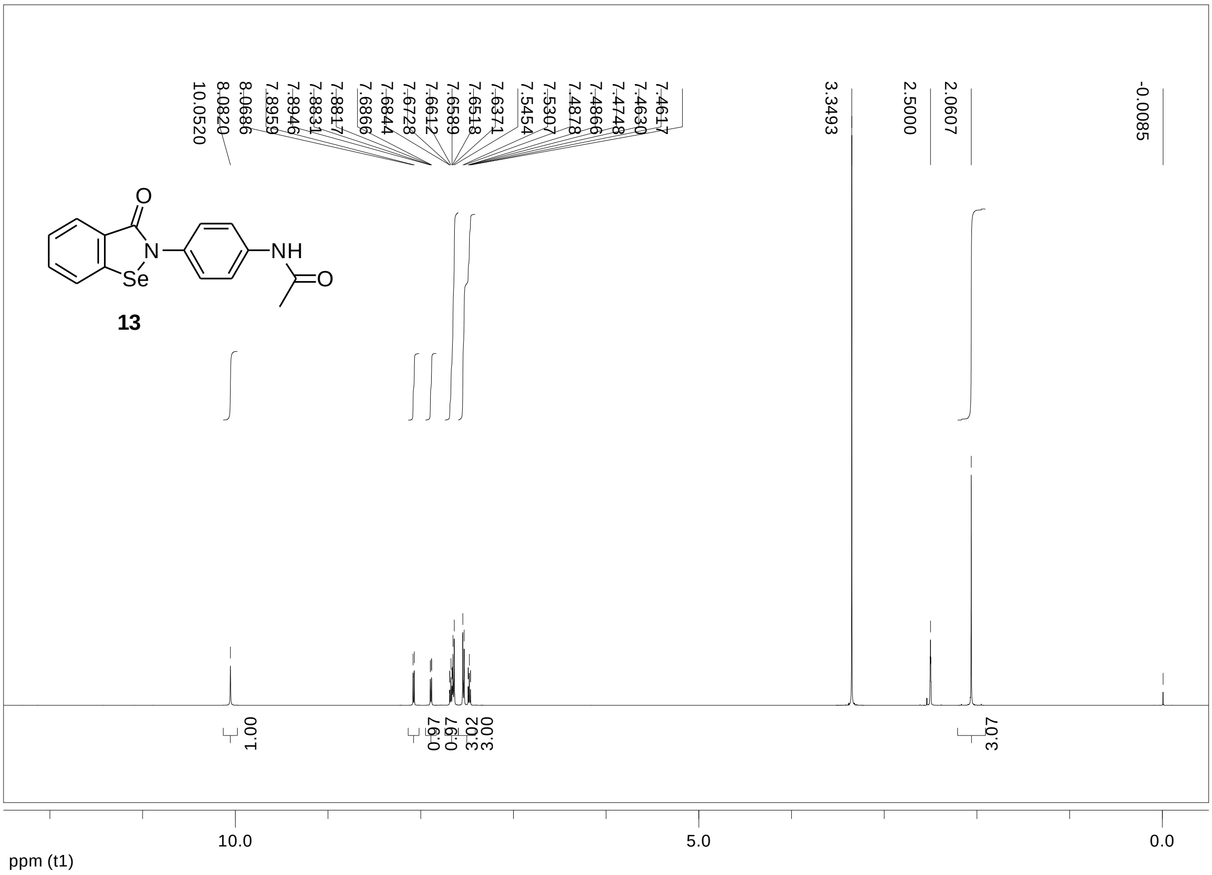

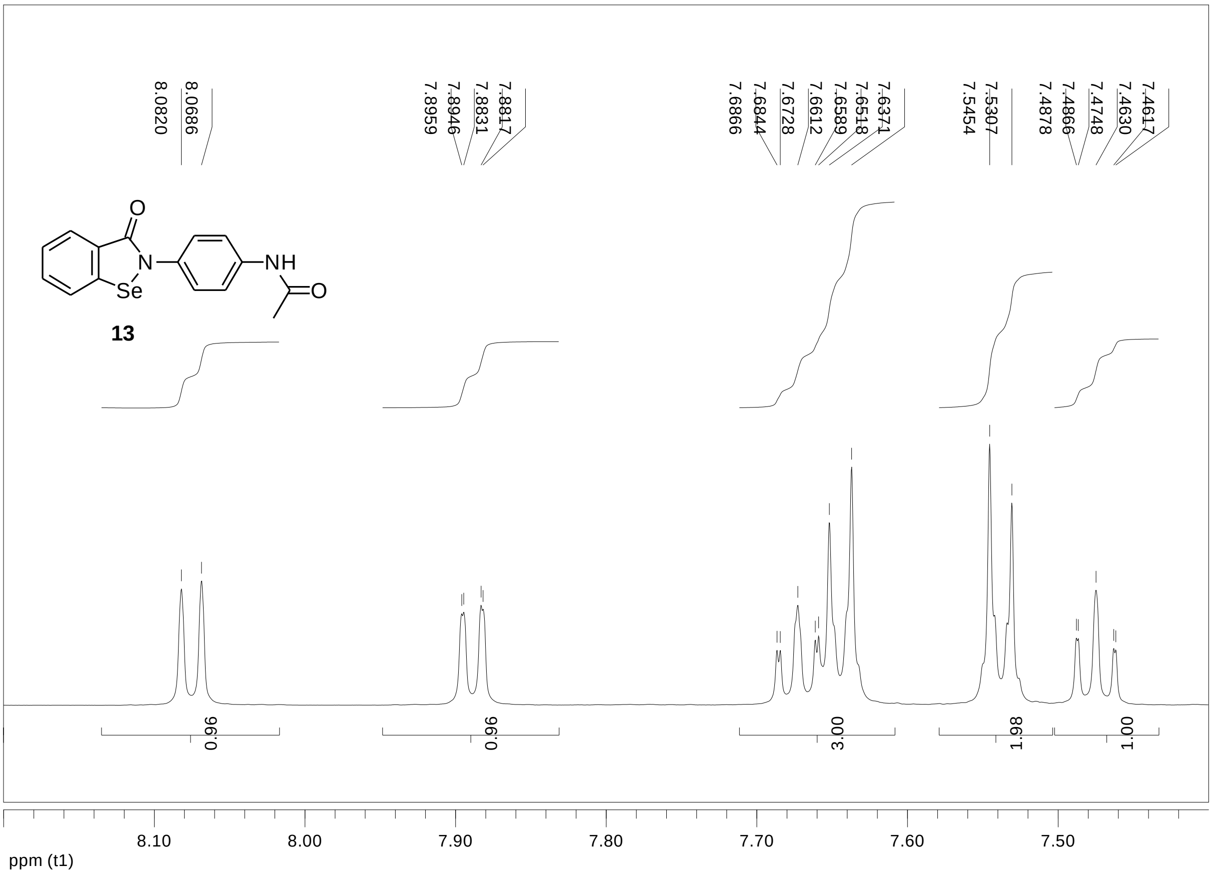

Fig. S64. ^{1}H-NMR (600.6 MHz, DMSO-d_6) spectrum of compound **13**

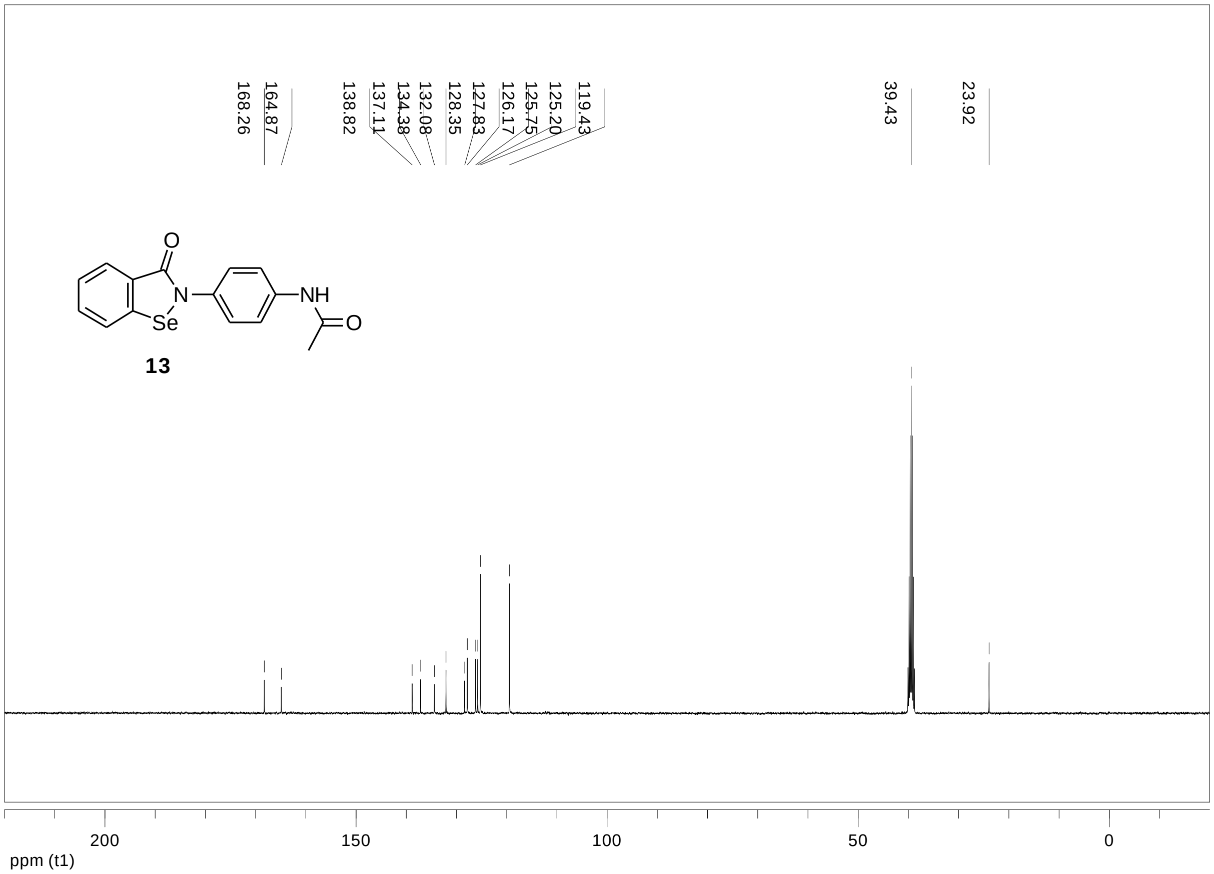

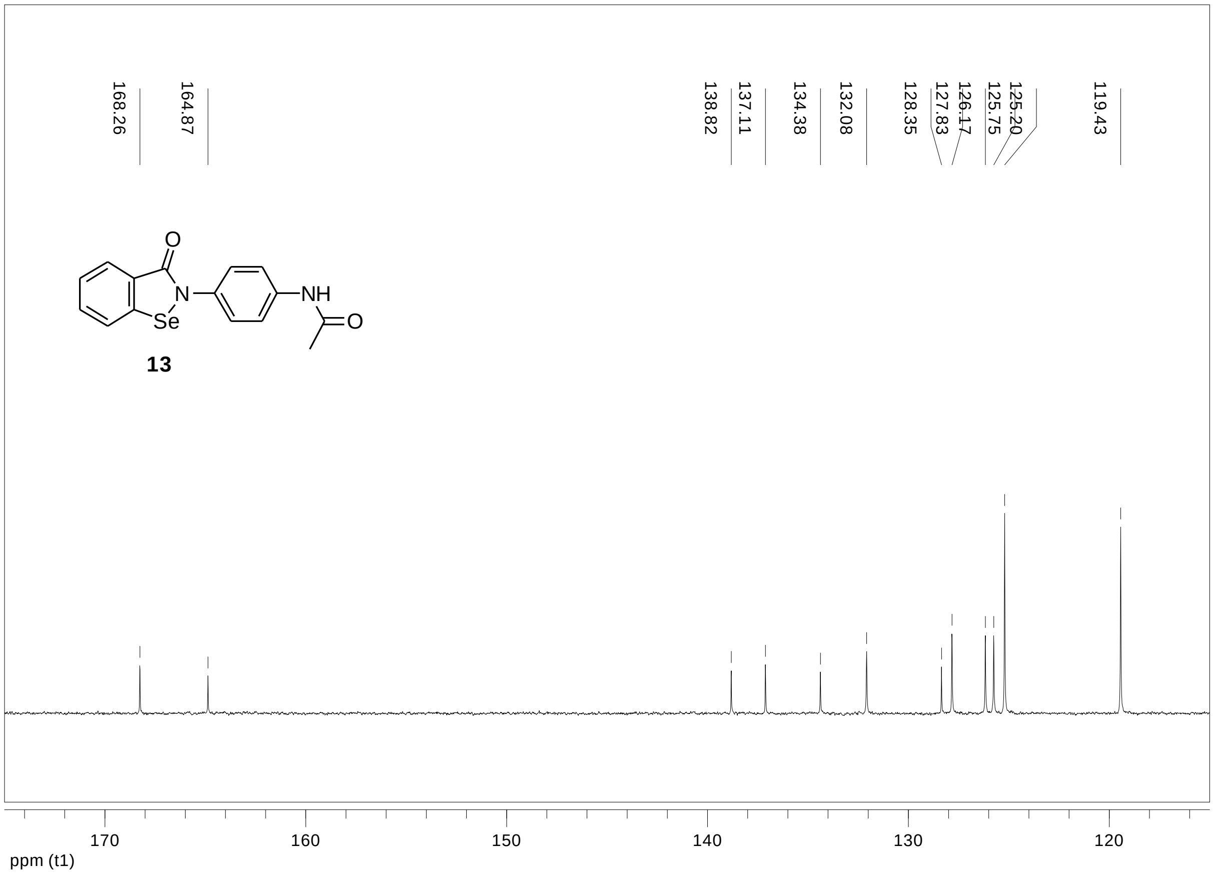

Fig. S65. ^{13}C-NMR (100.5 MHz, DMSO-d_6) spectrum of compound **13**

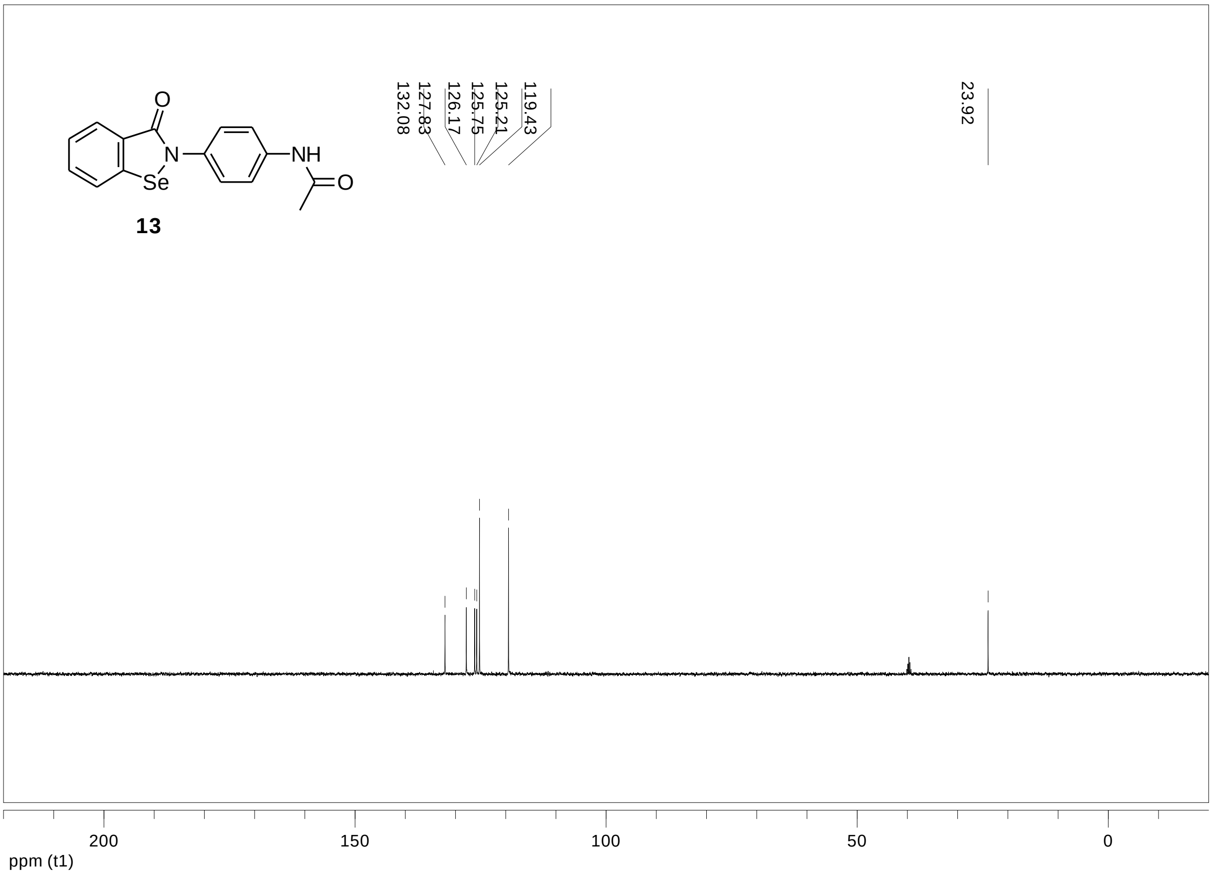

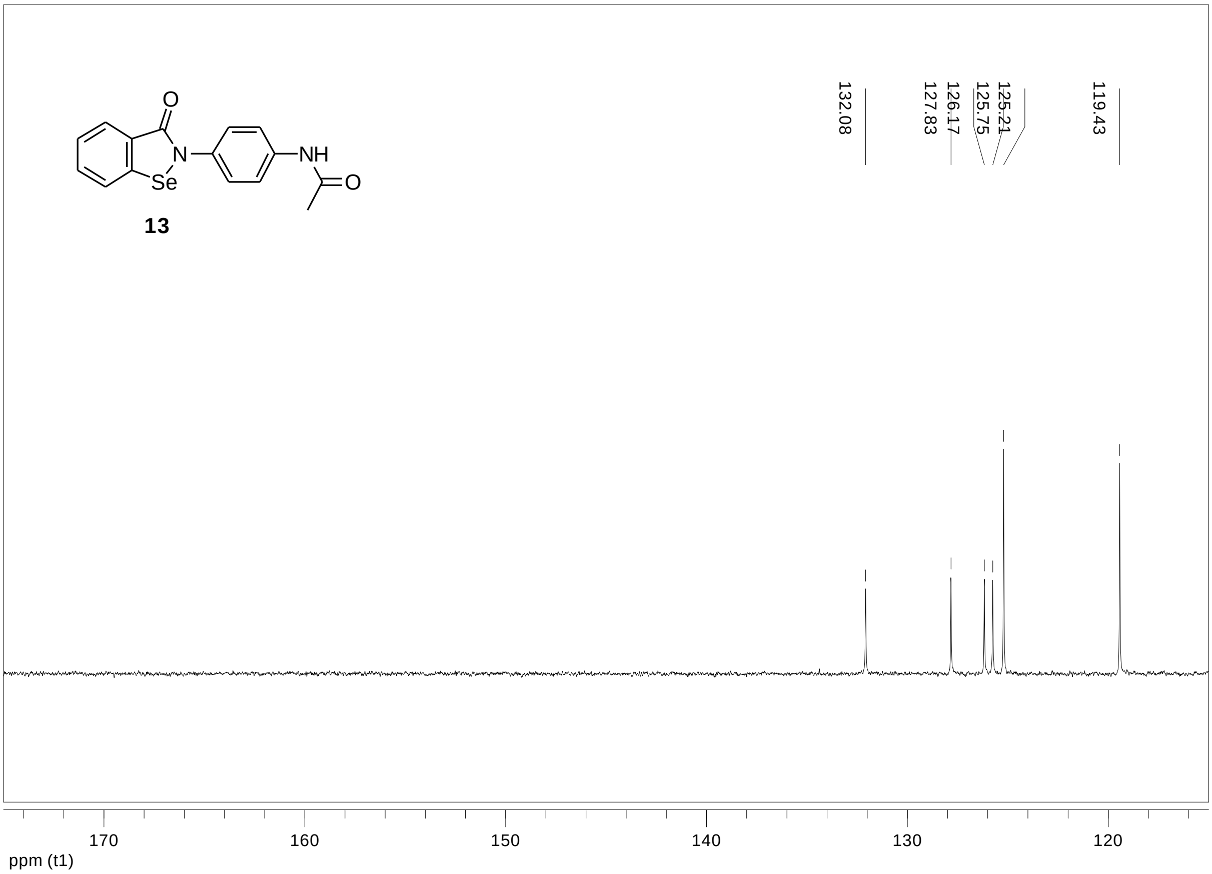

Fig. S66. ^{13}C-NMR (100.5 MHz, DMSO-d_6) dept-135 experiment of compound **13**

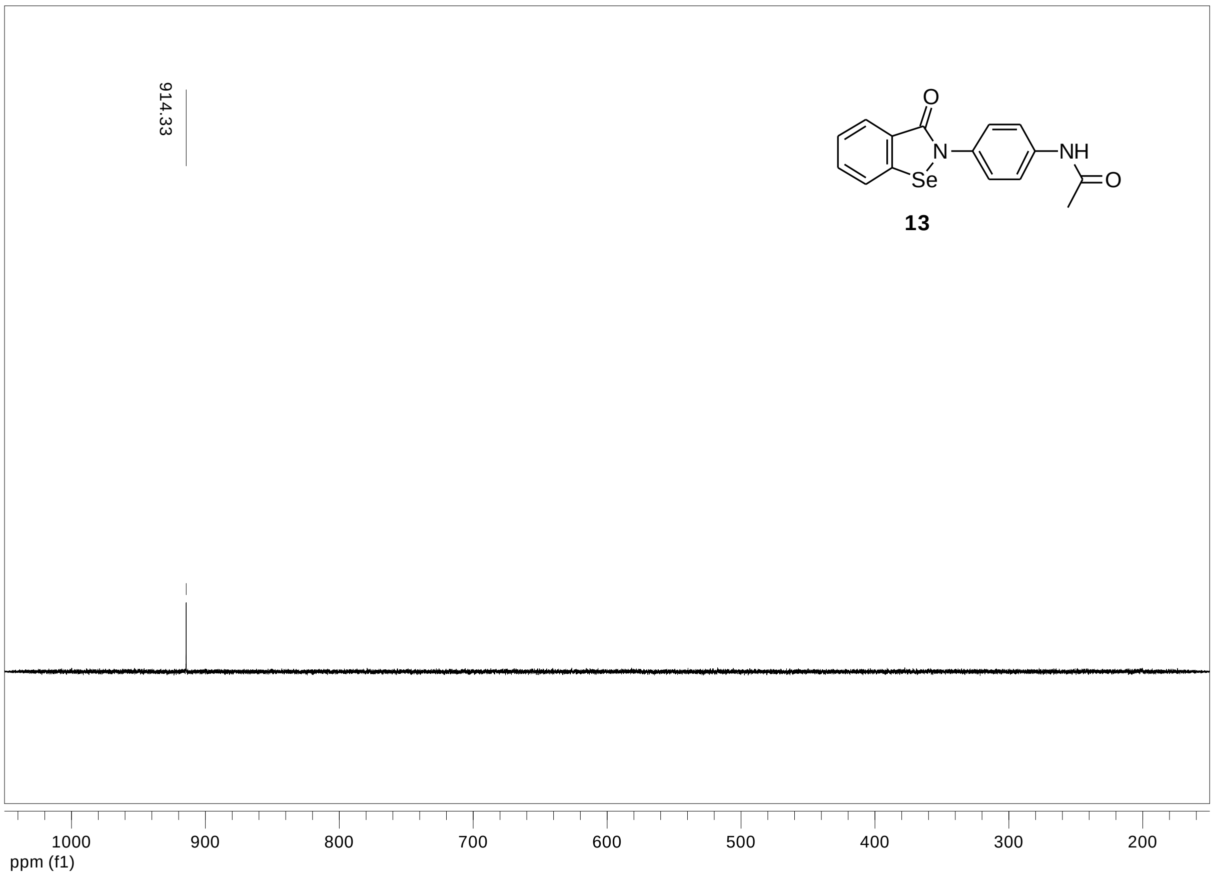

Fig. S67. ^{77}Se-NMR (76.24 MHz, DMSO-d_6) spectrum of compound **13**

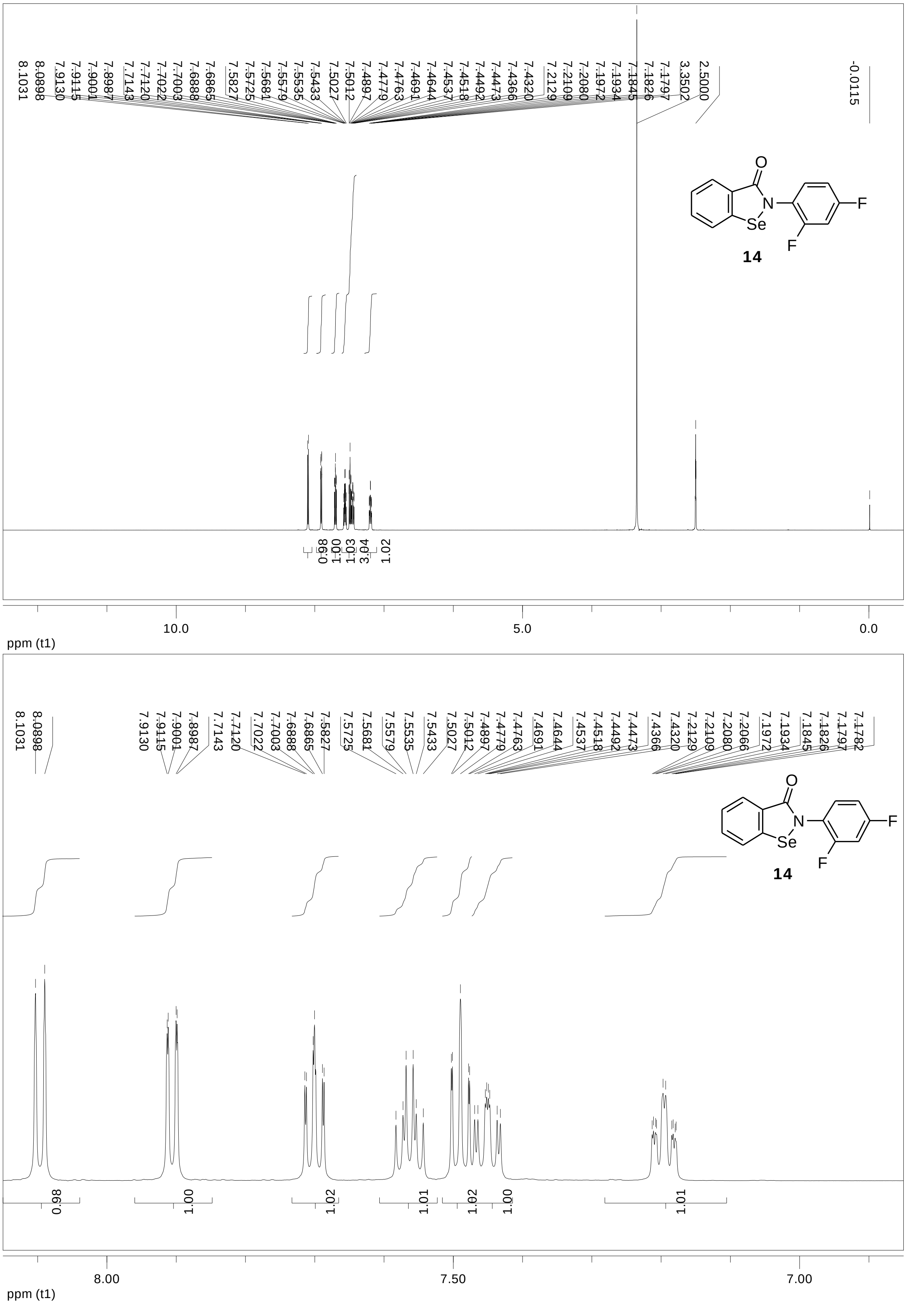

Fig. S68. ^{1}H-NMR (600.6 MHz, DMSO-d_6) spectrum of compound **14**

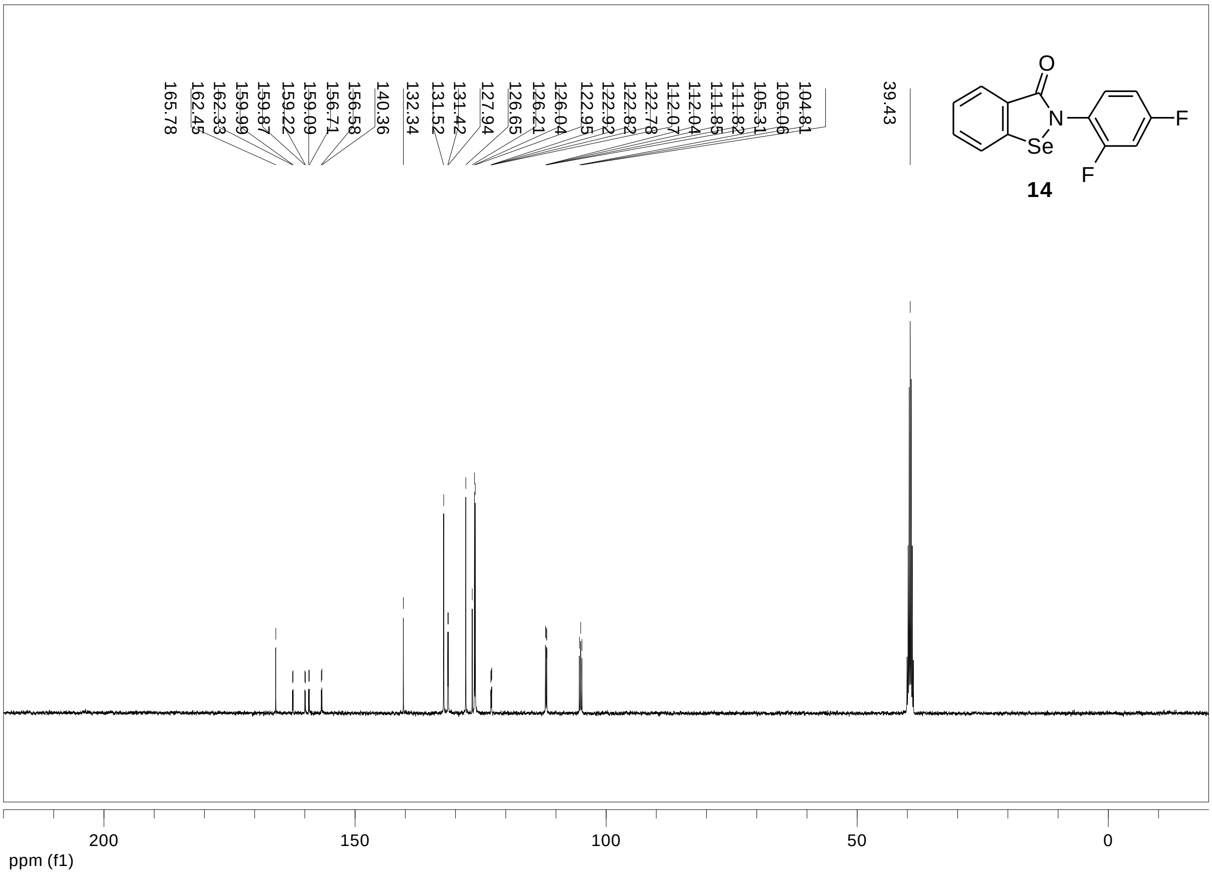

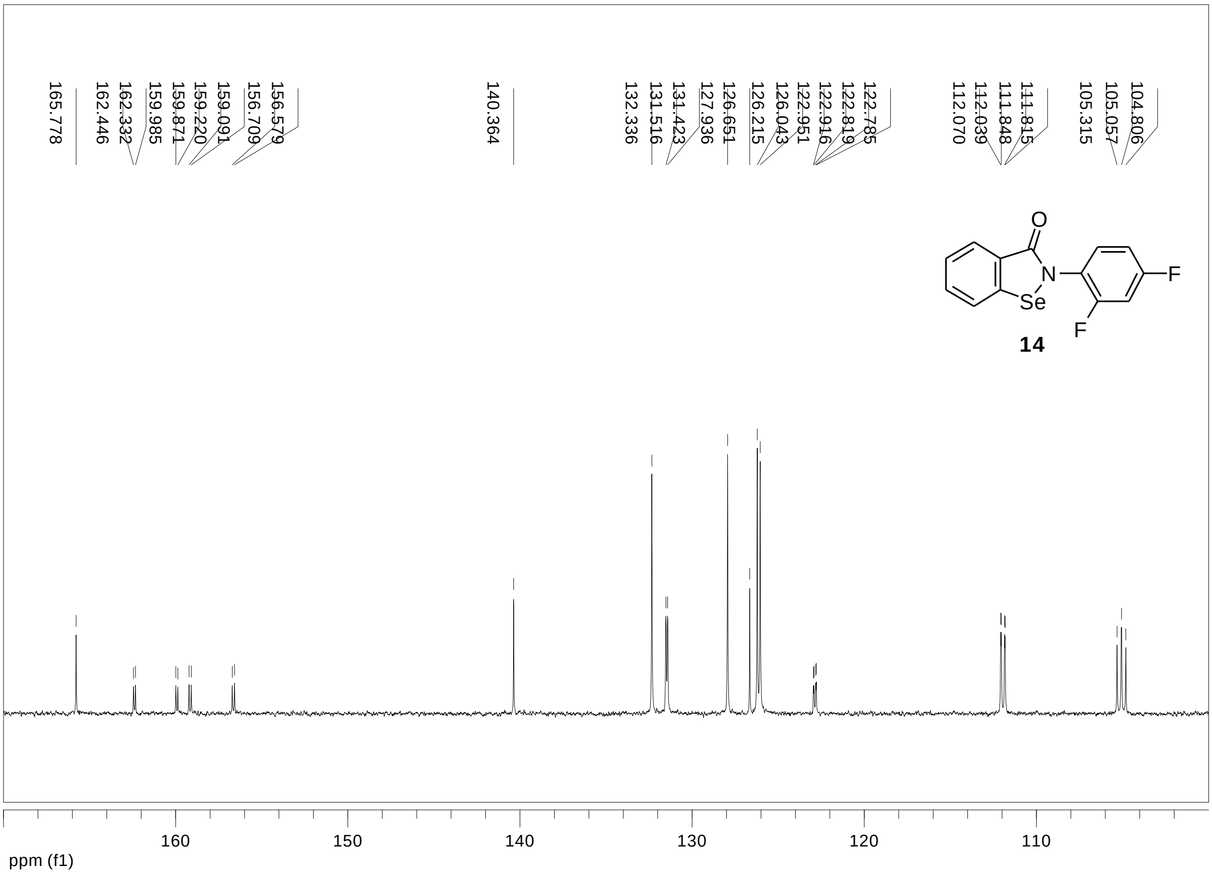

Fig. S69. ^{13}C-NMR (100.5 MHz, DMSO-d_6) spectrum of compound **14**

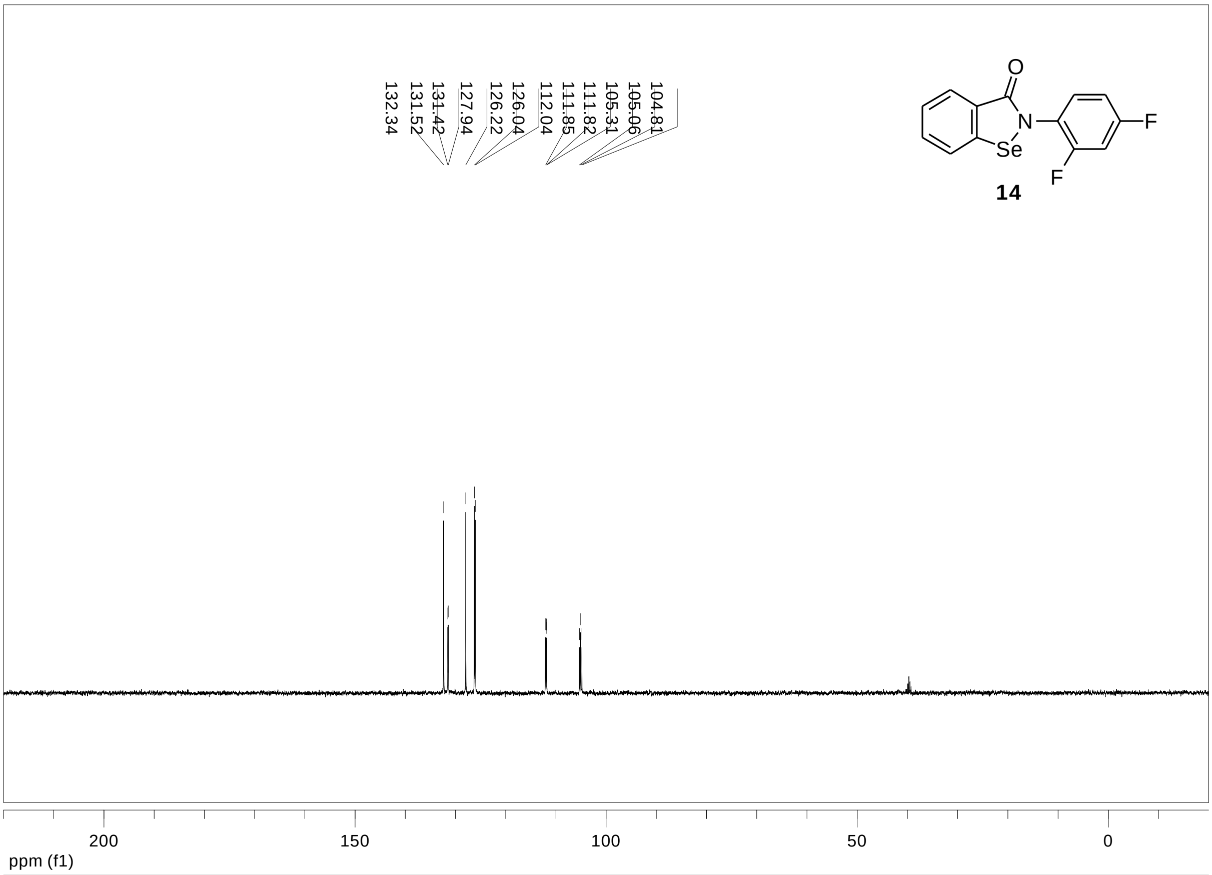

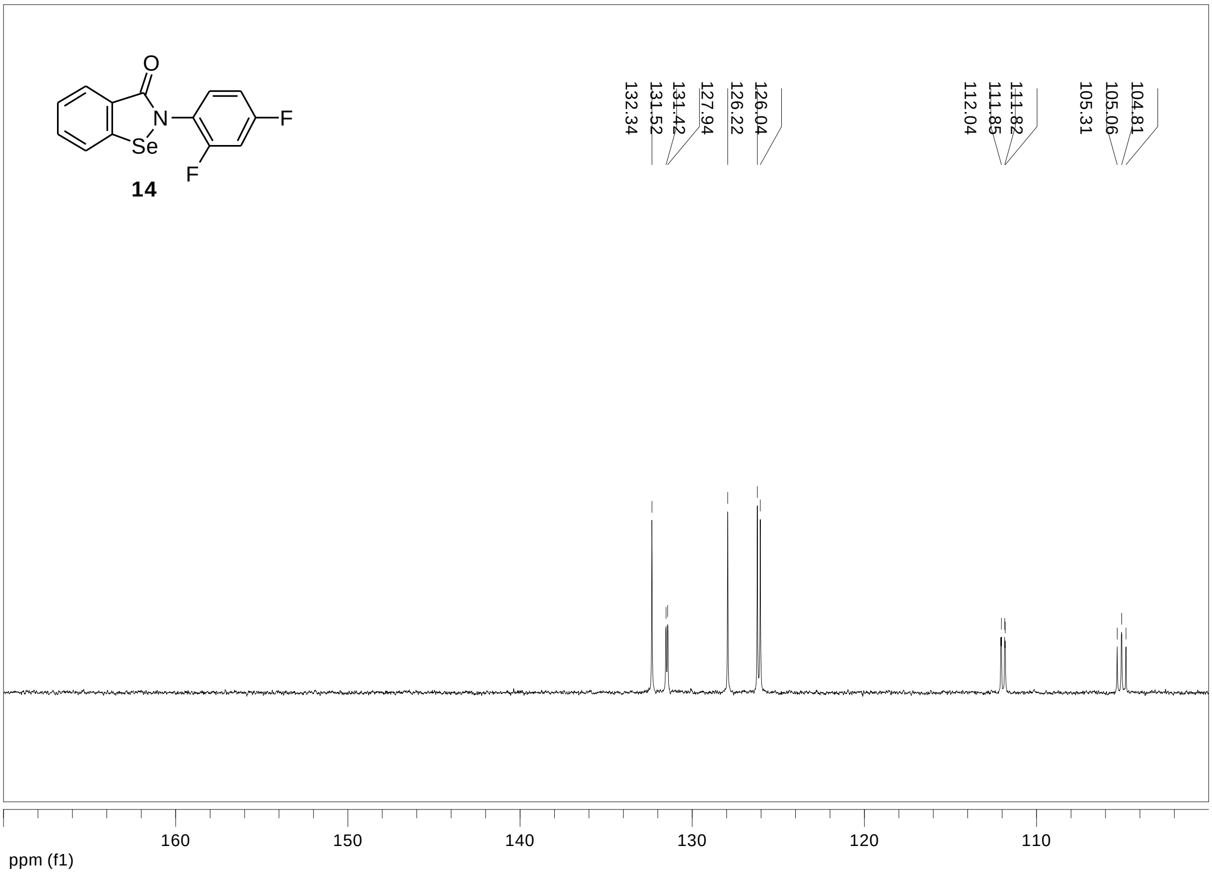

Fig. S70. ^{13}C-NMR (100.5 MHz, DMSO-d_6) dept-135 experiment of compound **14**

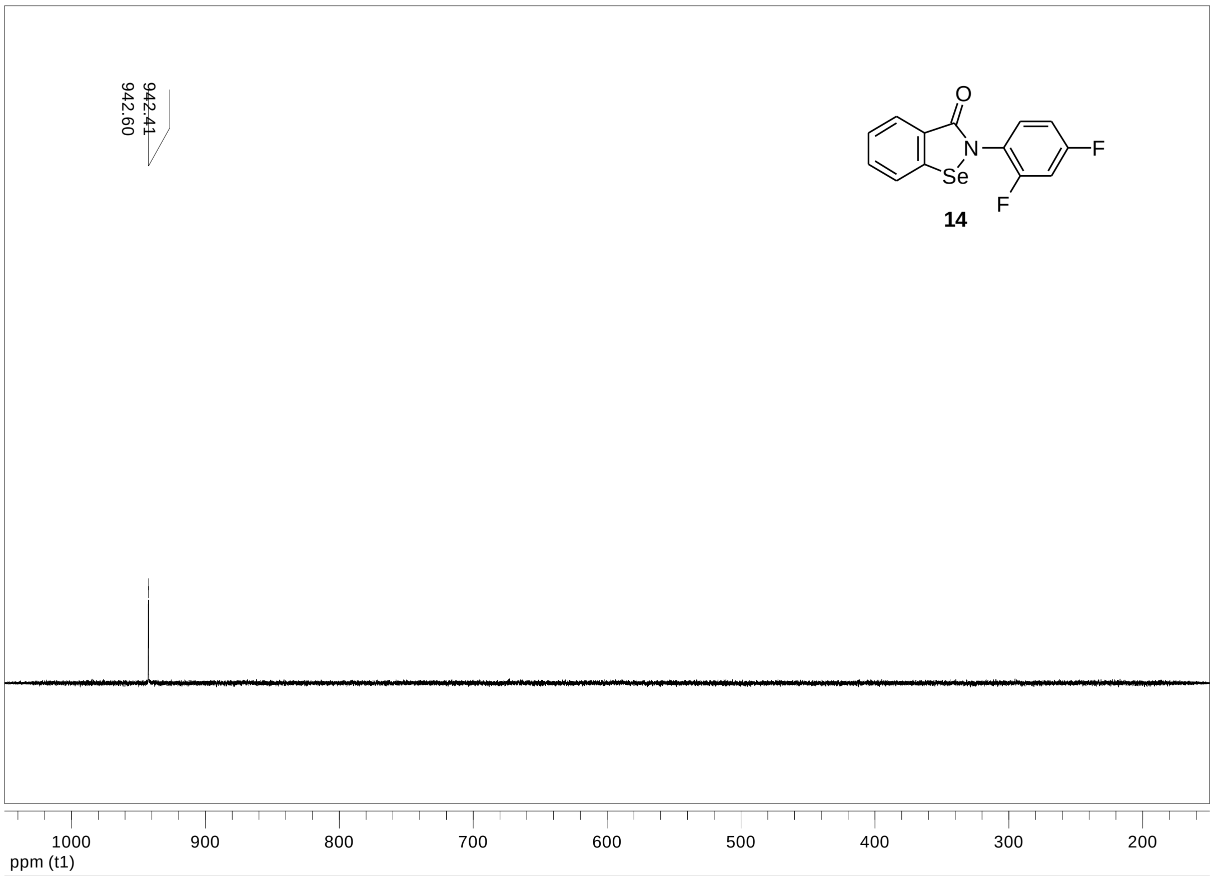

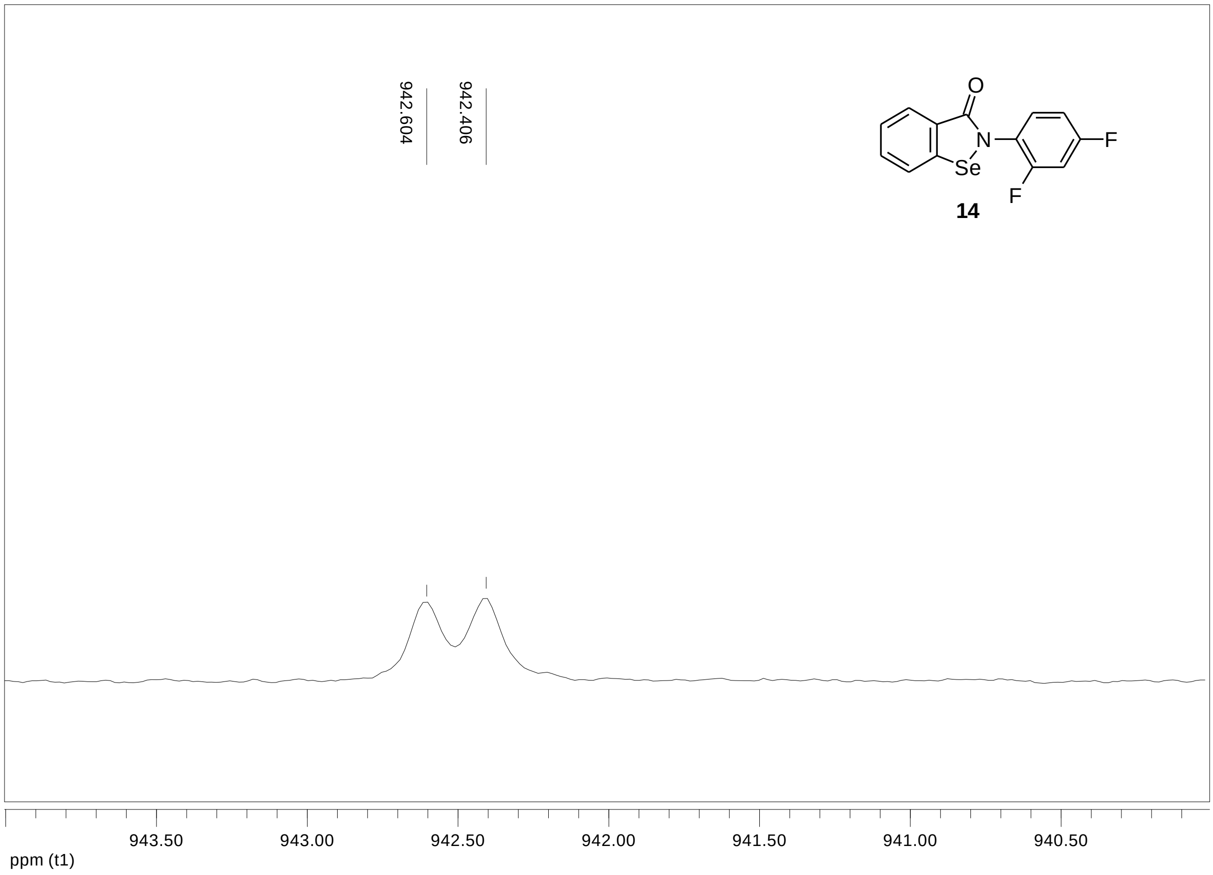

Fig. S71. ^{77}Se-NMR (76.24 MHz, DMSO-d_6) spectrum of compound **14**

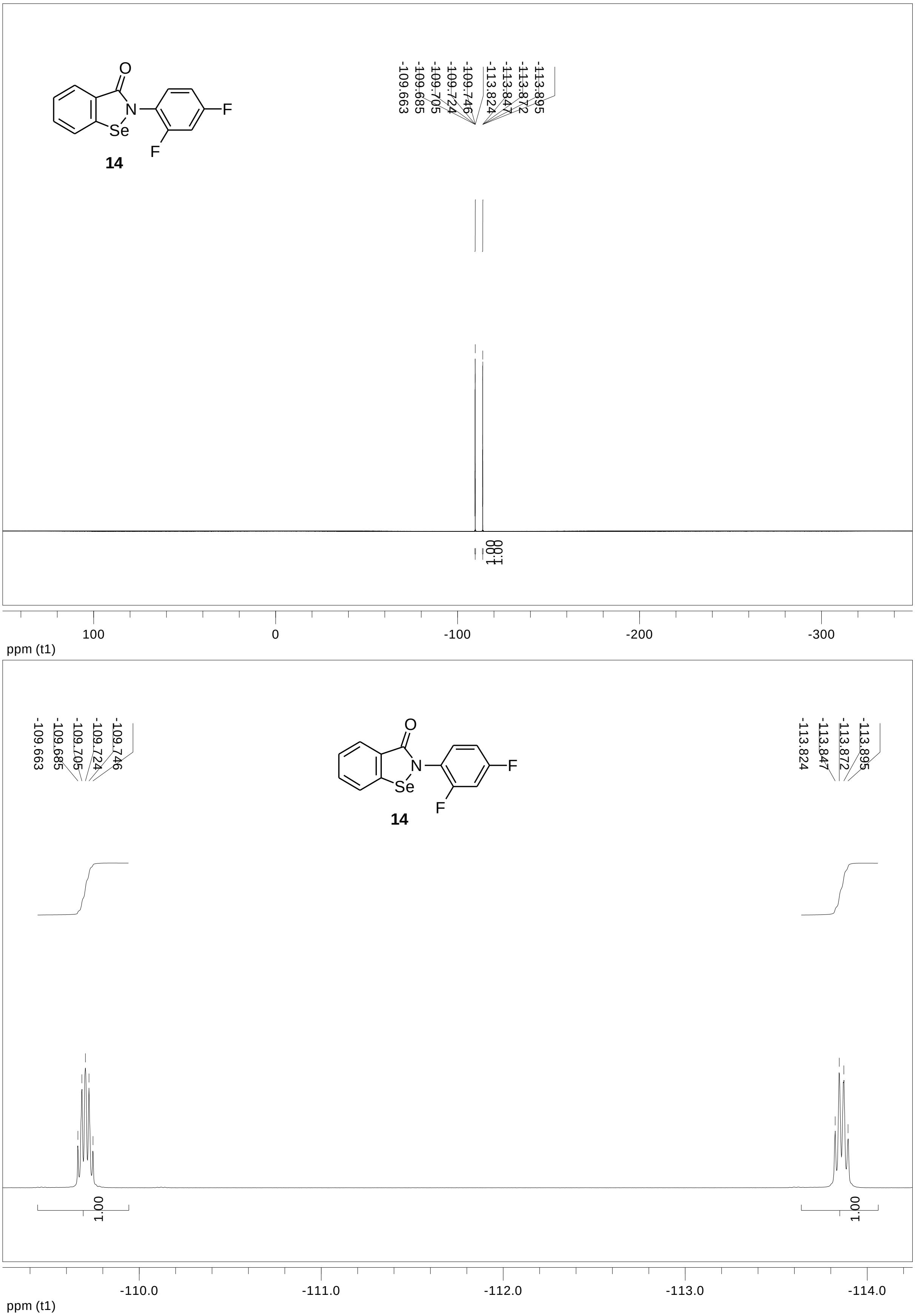

Fig. S72. ^{19}F-NMR (376.2 MHz, DMSO-d_6) spectrum of compound **14**

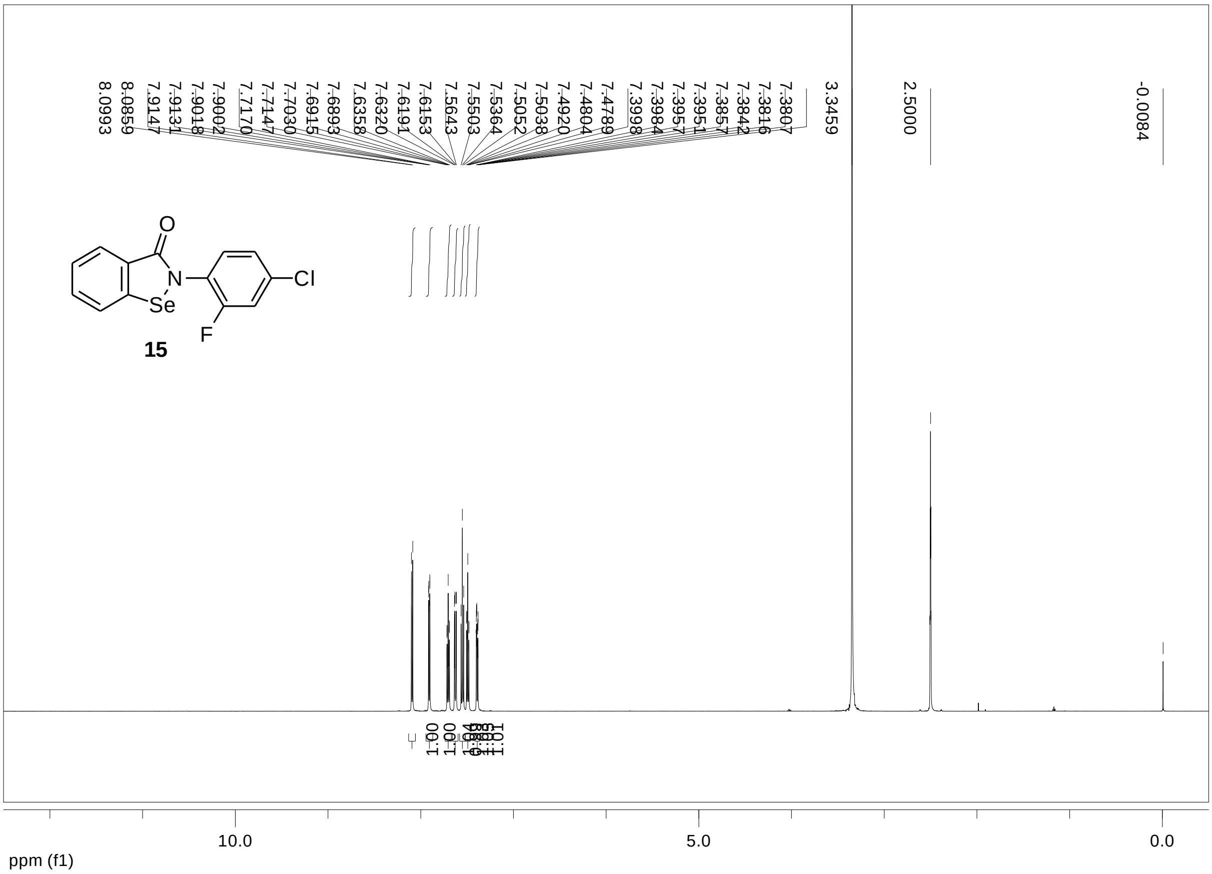

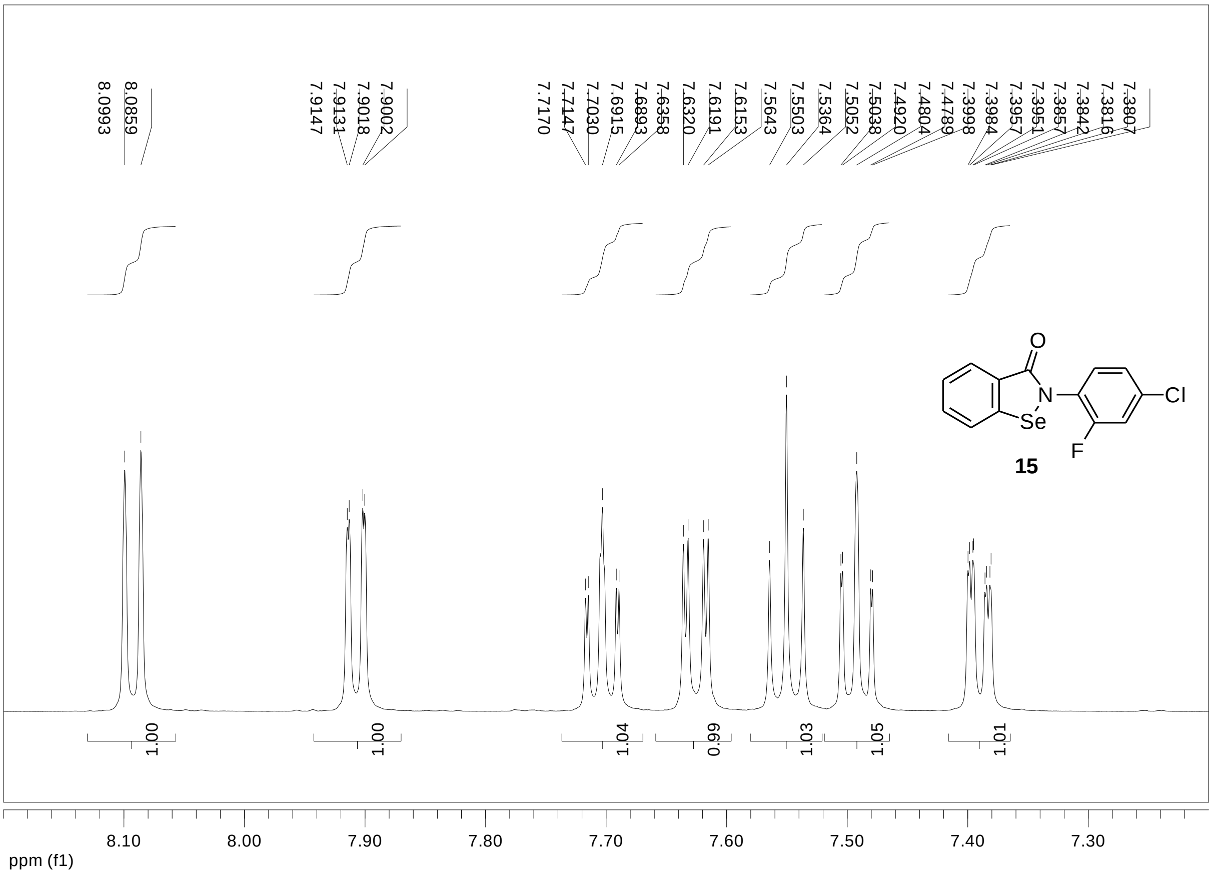

Fig. S73. ^{1}H-NMR (600.6 MHz, DMSO-d_6) spectrum of compound **15**

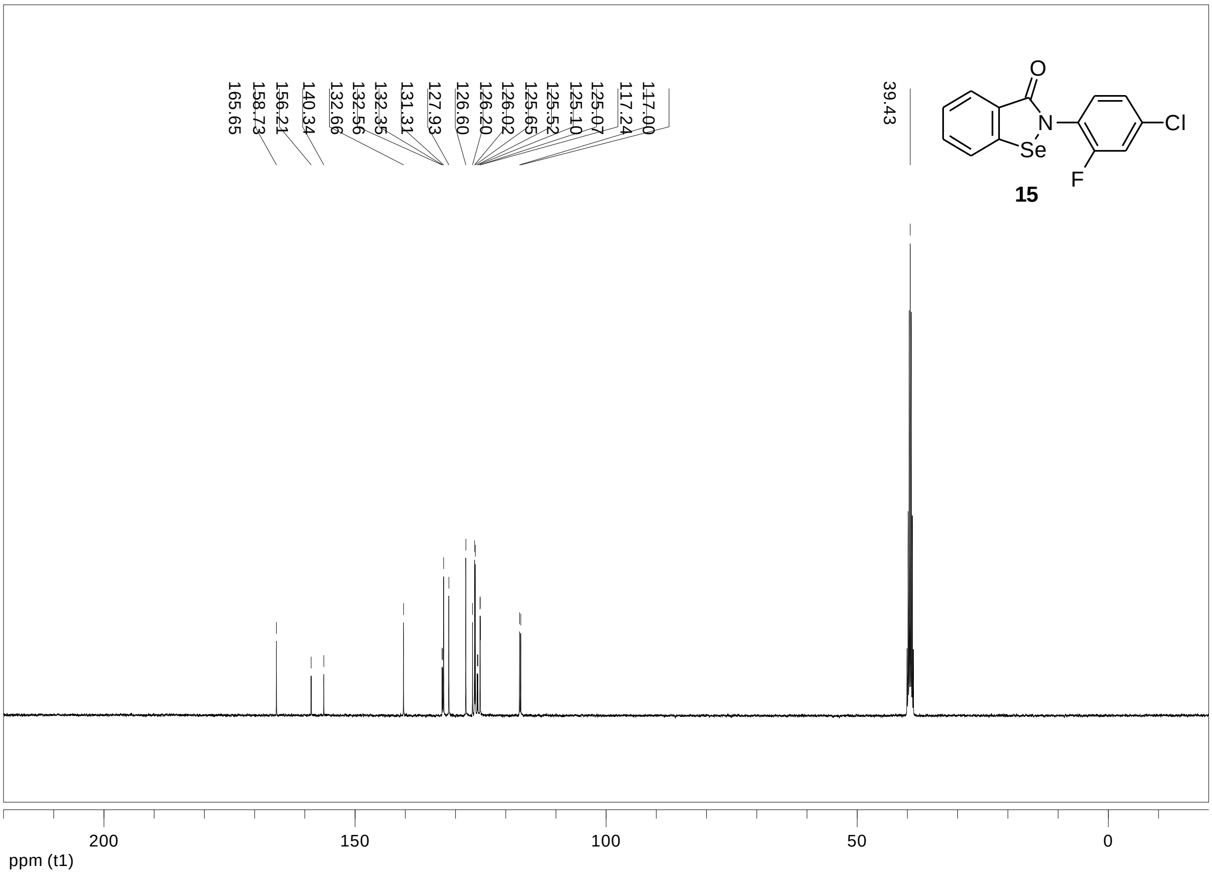

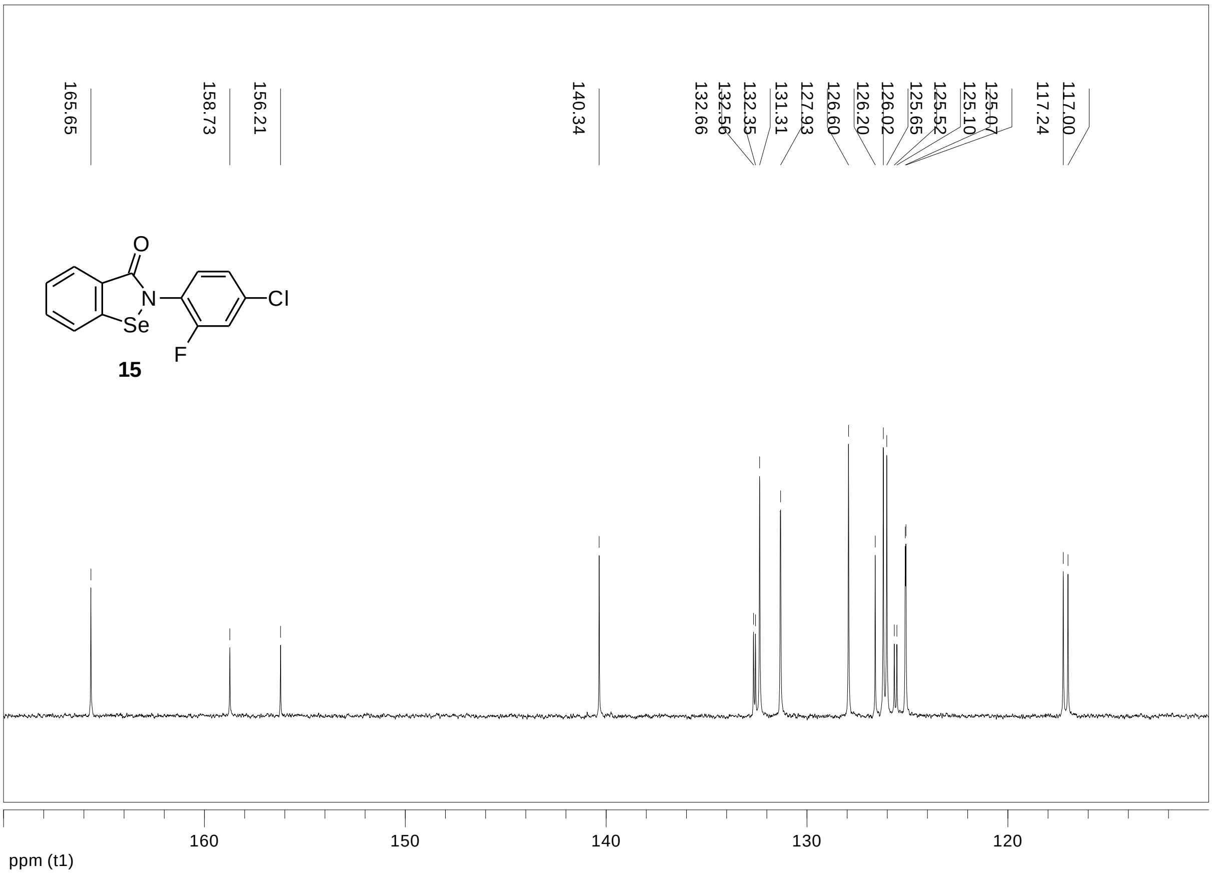

Fig. S74. ^{13}C-NMR (100.5 MHz, DMSO-d_6) spectrum of compound **15**

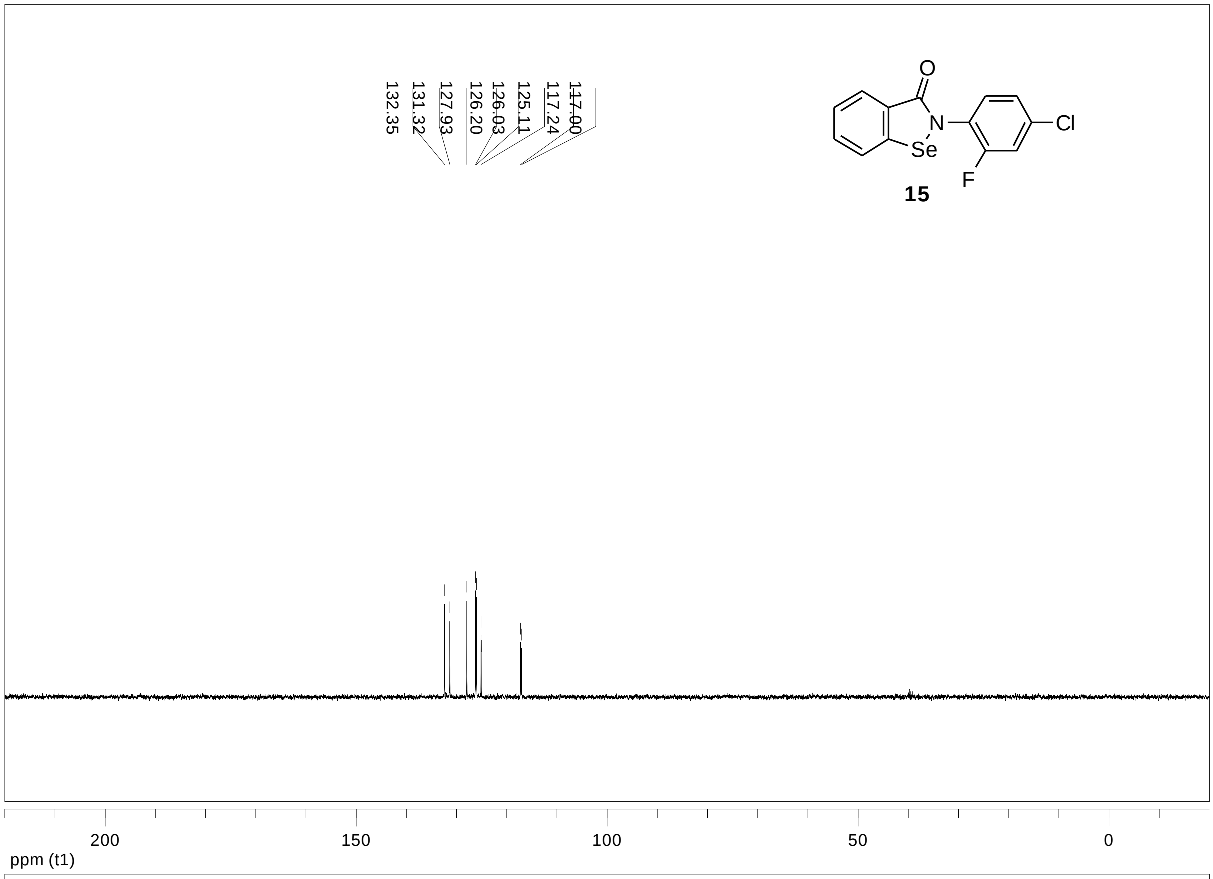

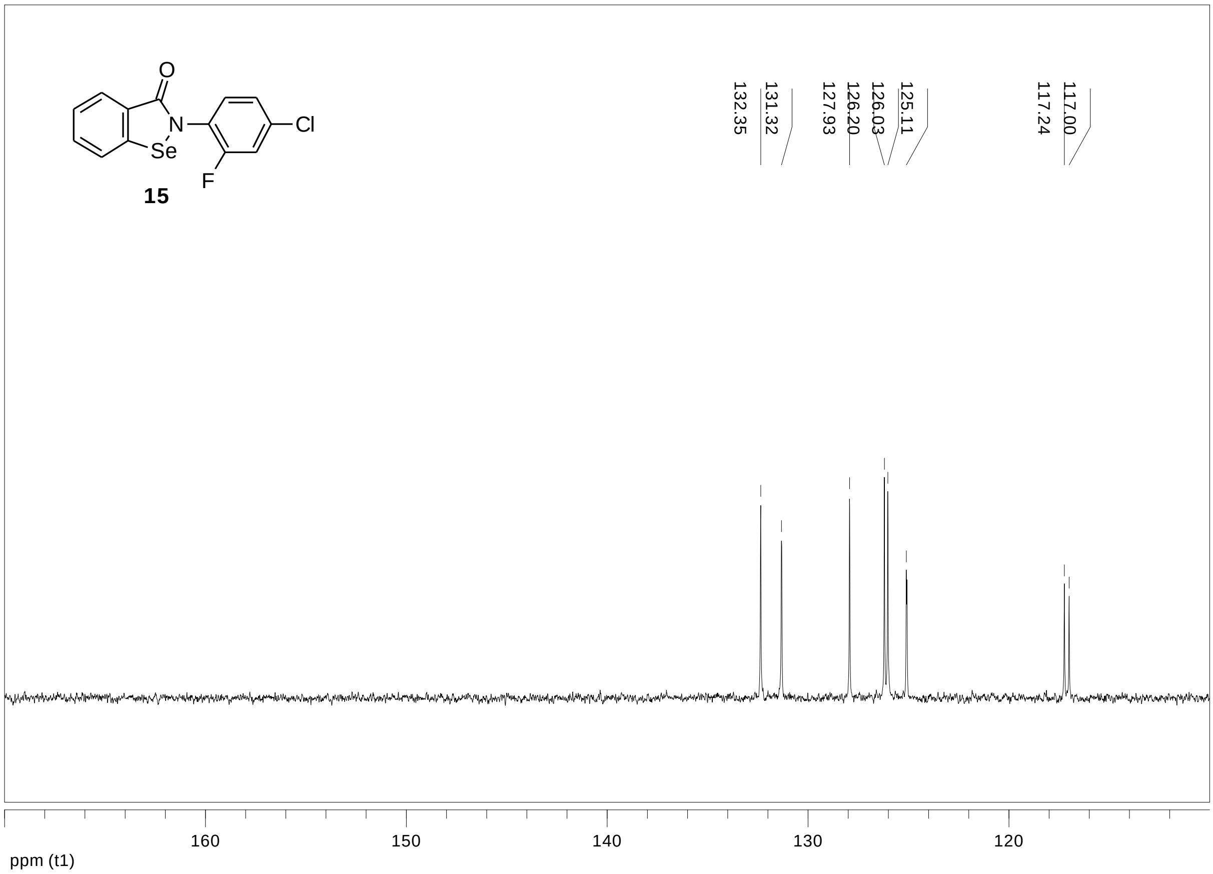

Fig. S75. ^{13}C-NMR (100.52 MHz, DMSO-d_6) dept-135 experiment of compound **15**

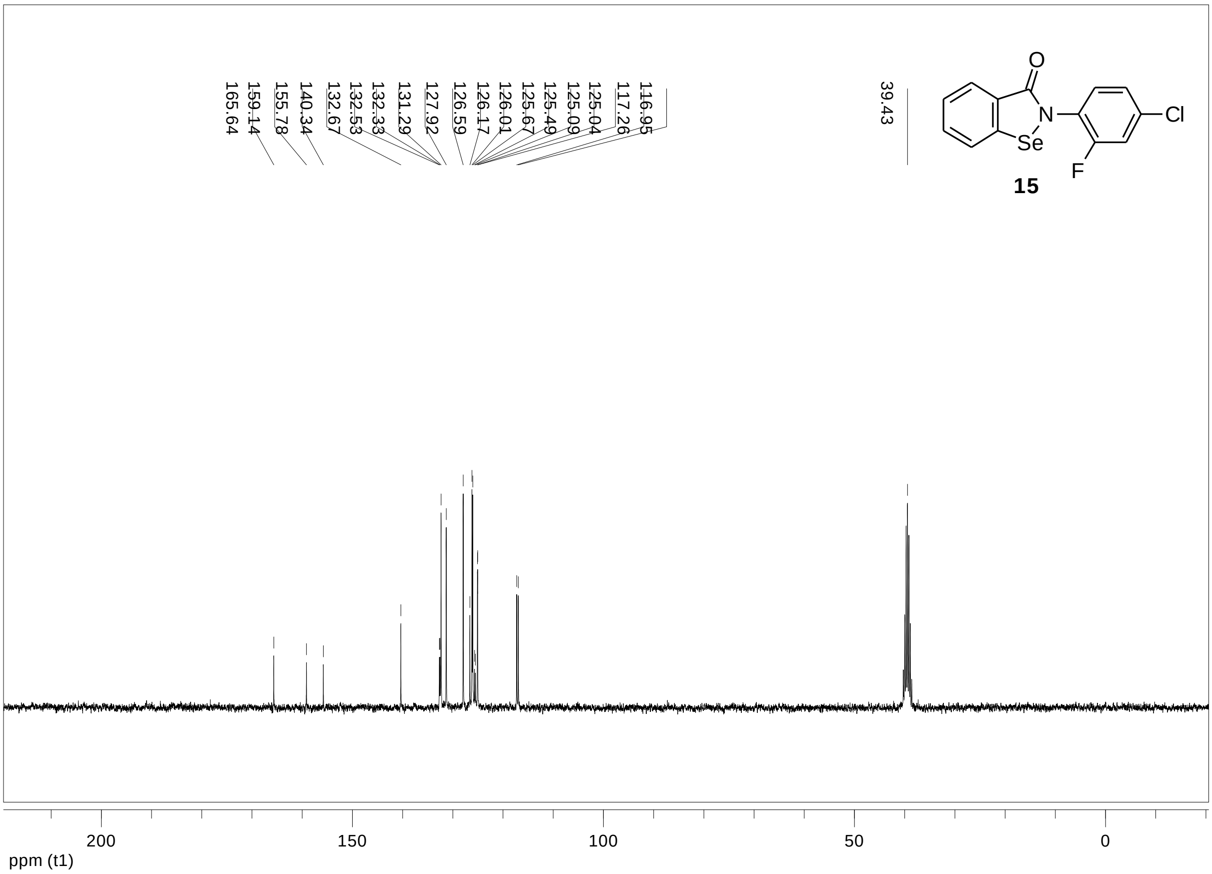

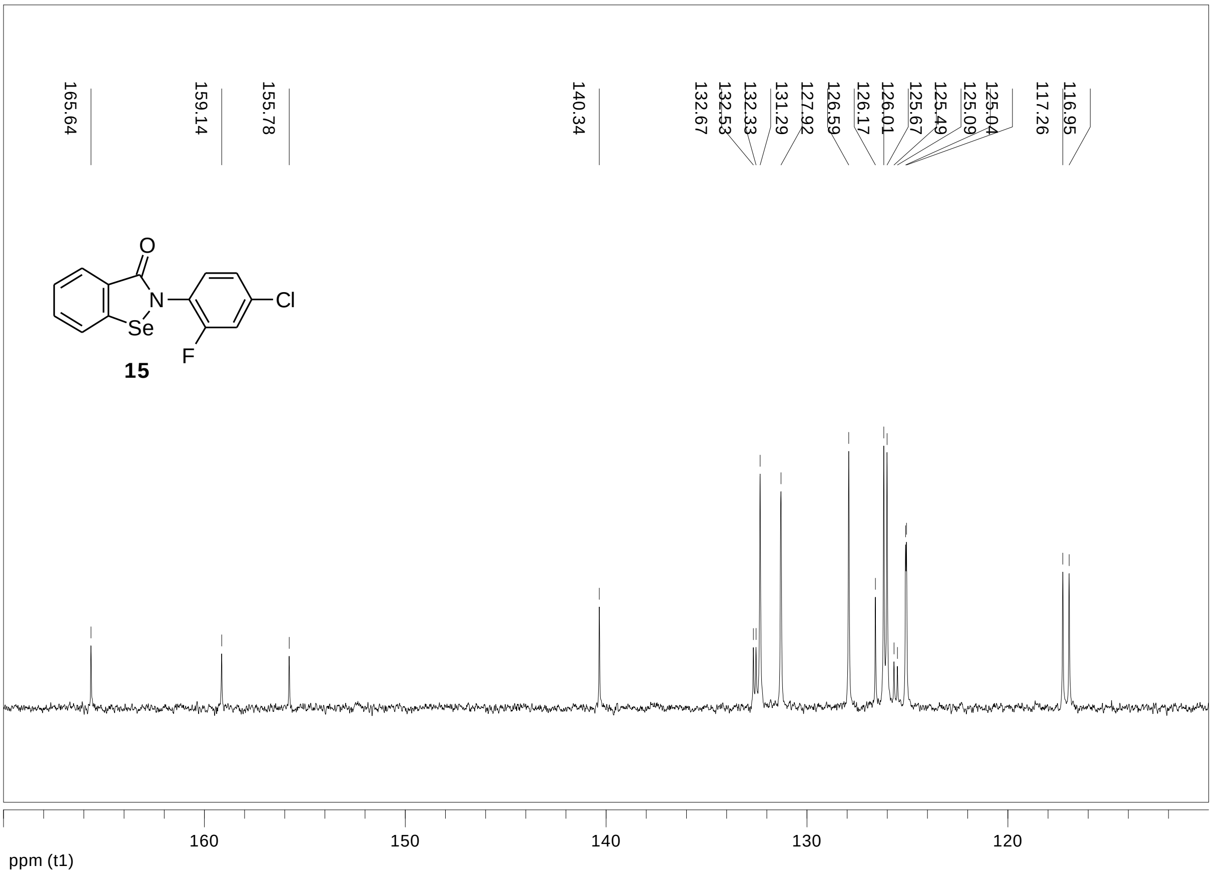

Fig. S76. ^{13}C-NMR (75.48 MHz, DMSO-d_6) spectrum of compound **15**

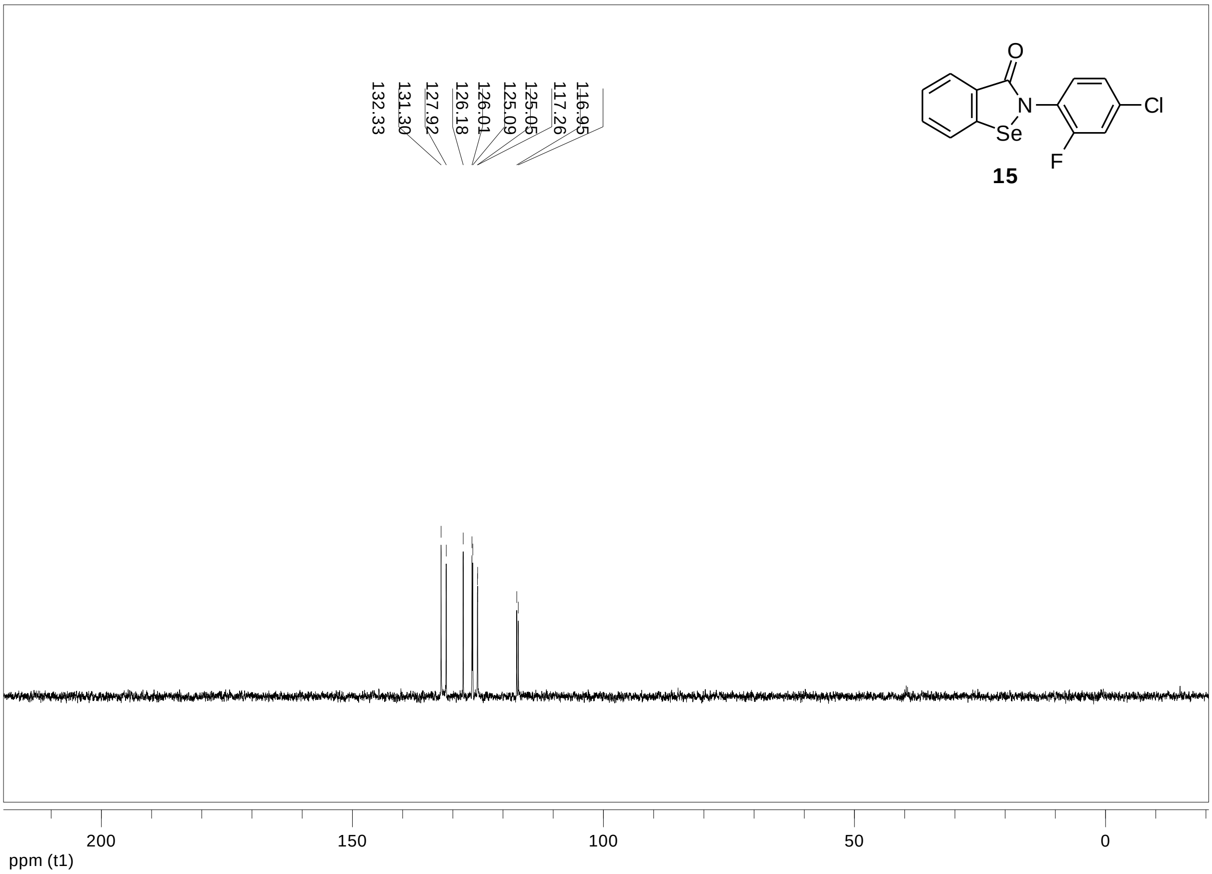

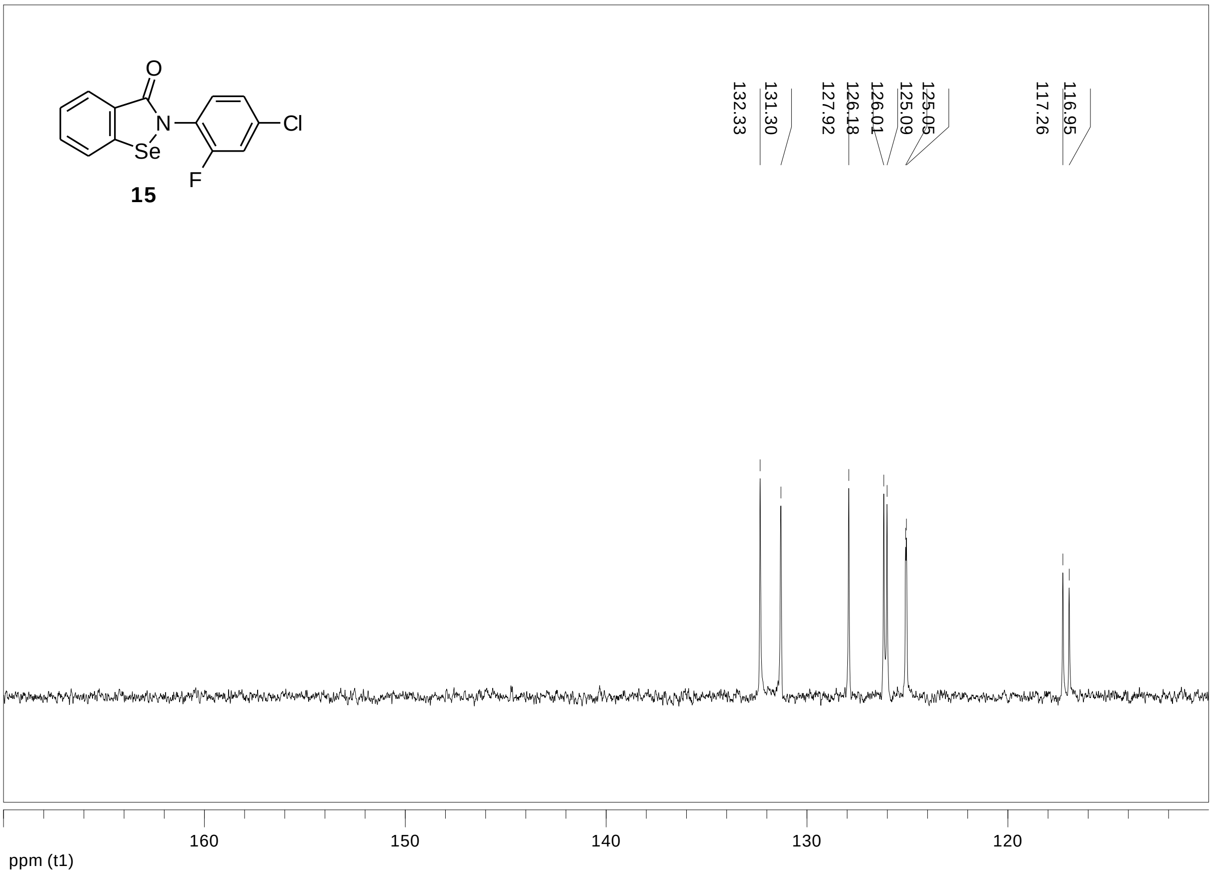

Fig. S77. ^{13}C-NMR (75.5 MHz, DMSO-d_6) dept-135 experiment of compound **15**

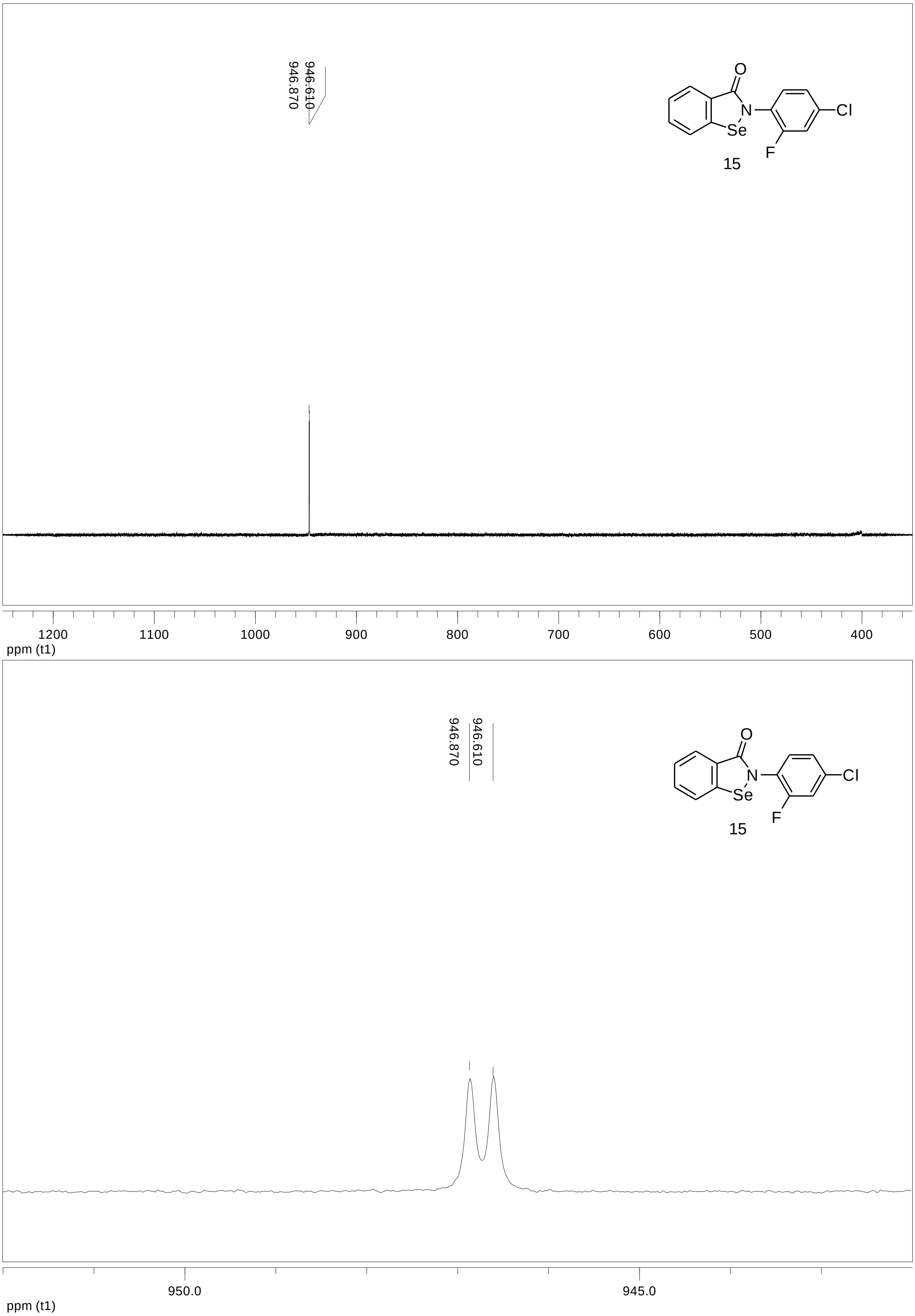

Fig. S78. ^{77}Se-NMR (76.24 MHz, DMSO-d_6) spectrum of compound **15**

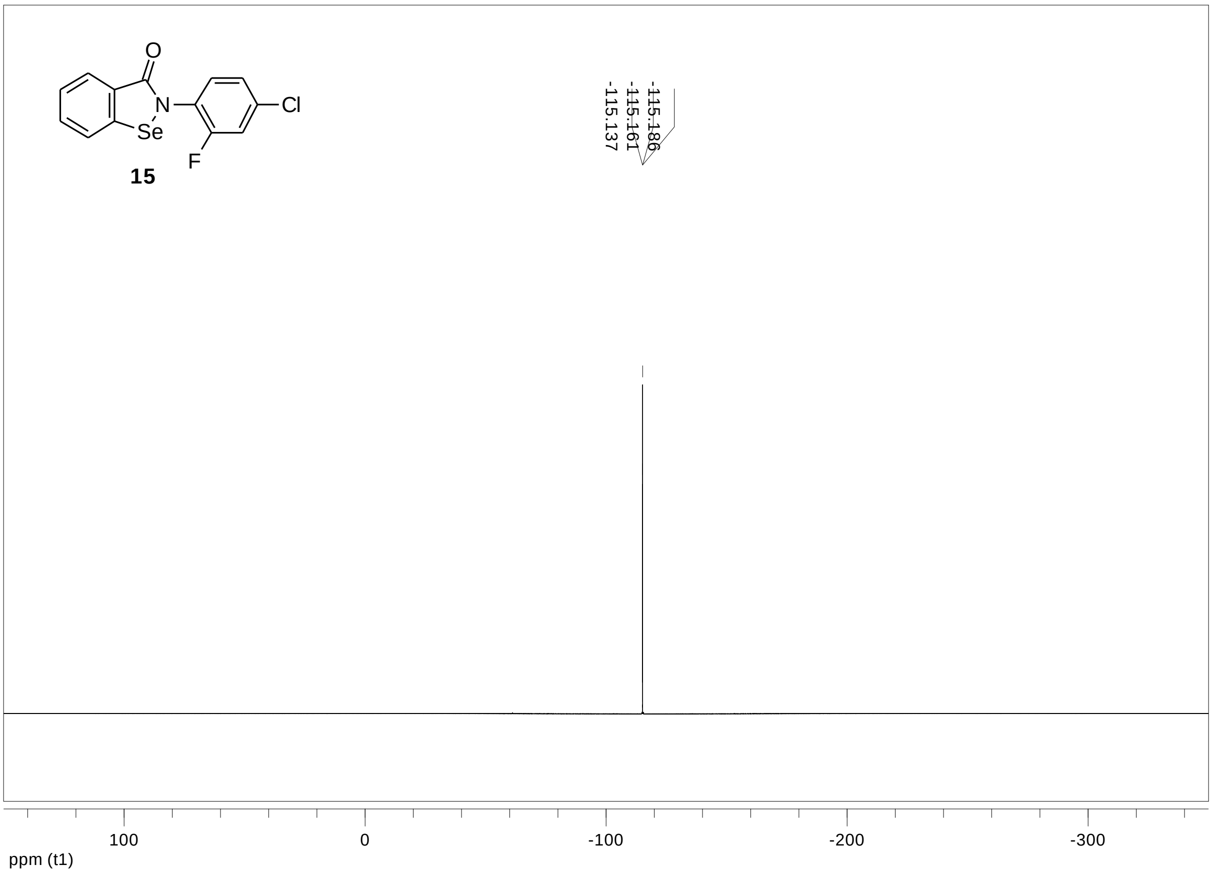

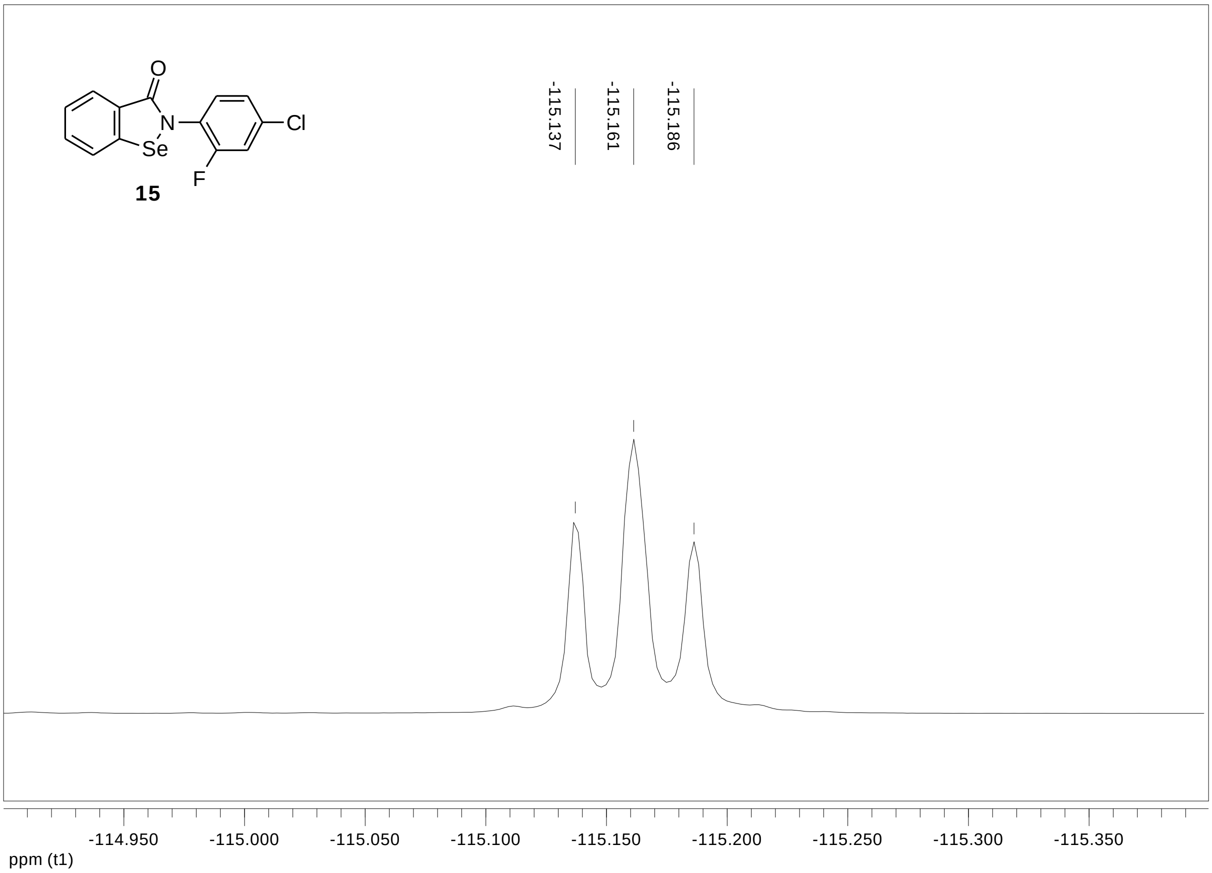

Fig. S79. ^{19}F-NMR (376.17 MHz, DMSO-d_6) spectrum of compound **15**

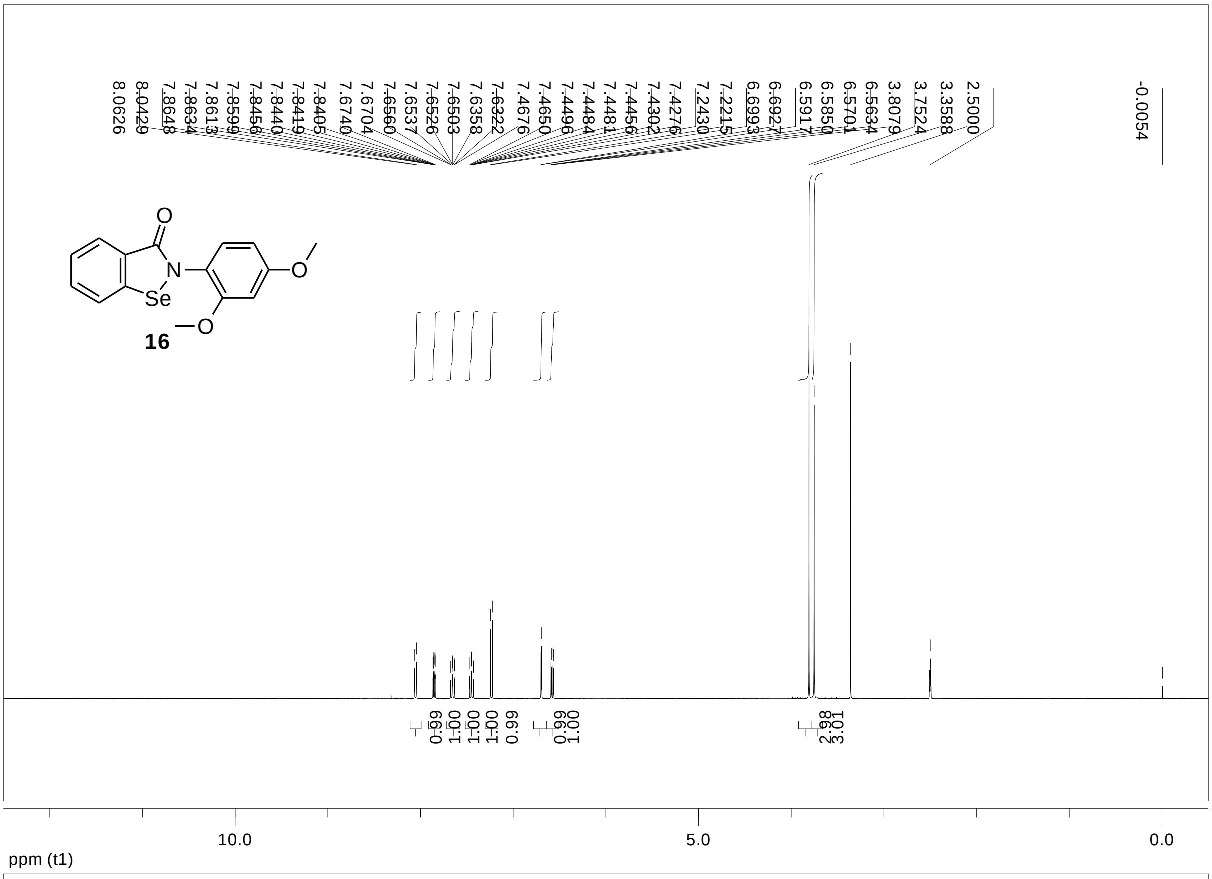

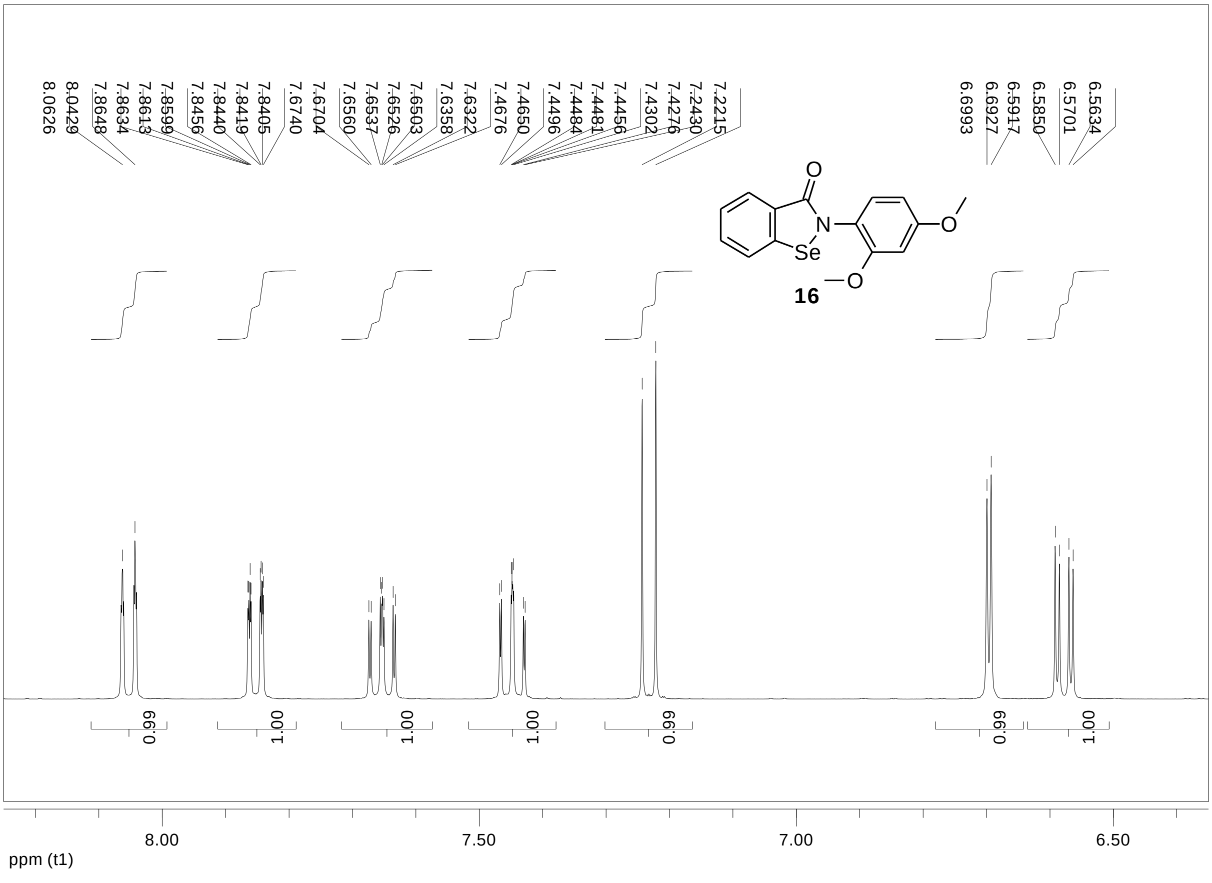

Fig. S80. ^{1}H-NMR (399.8 MHz, DMSO-d_6) spectrum of compound **16**

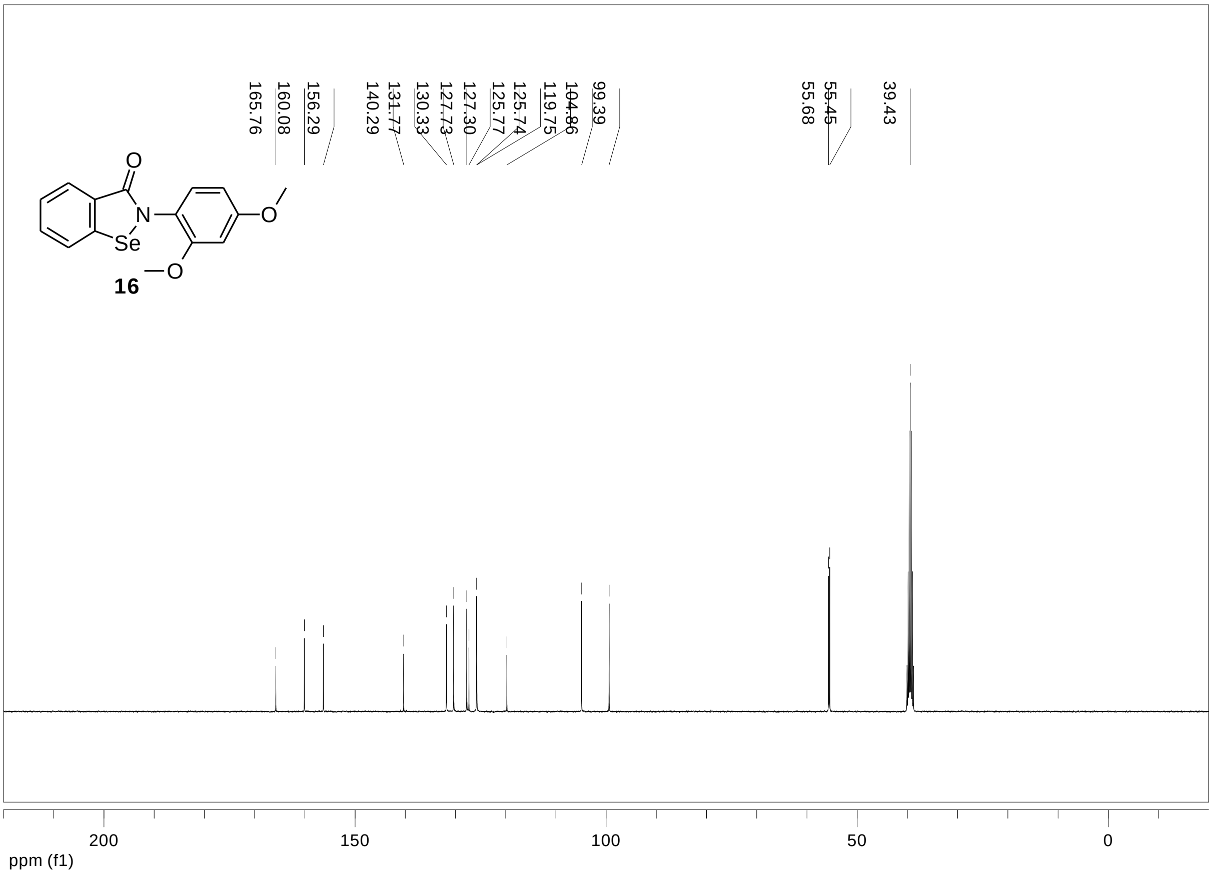

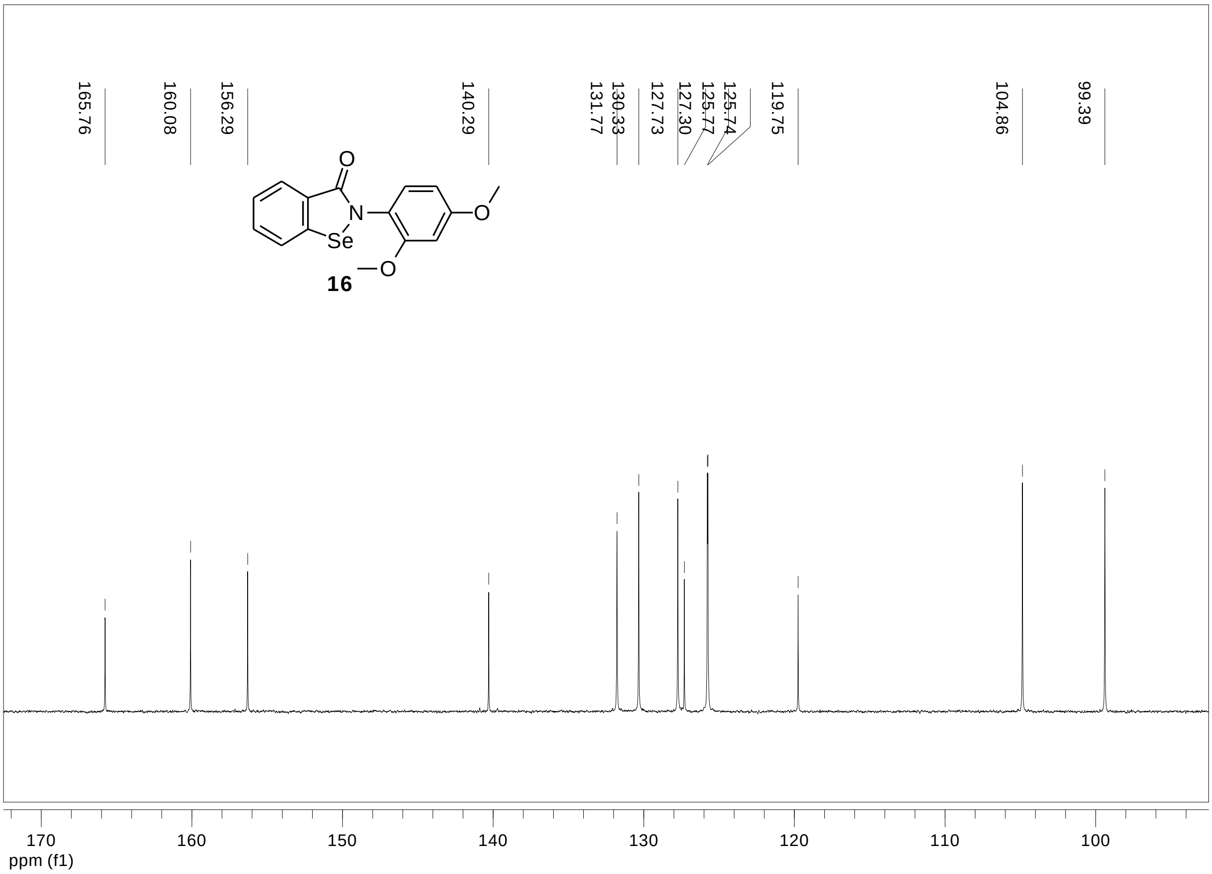

Fig. S81. ^{13}C-NMR (100.5 MHz, DMSO-d_6) spectrum of compound **16**

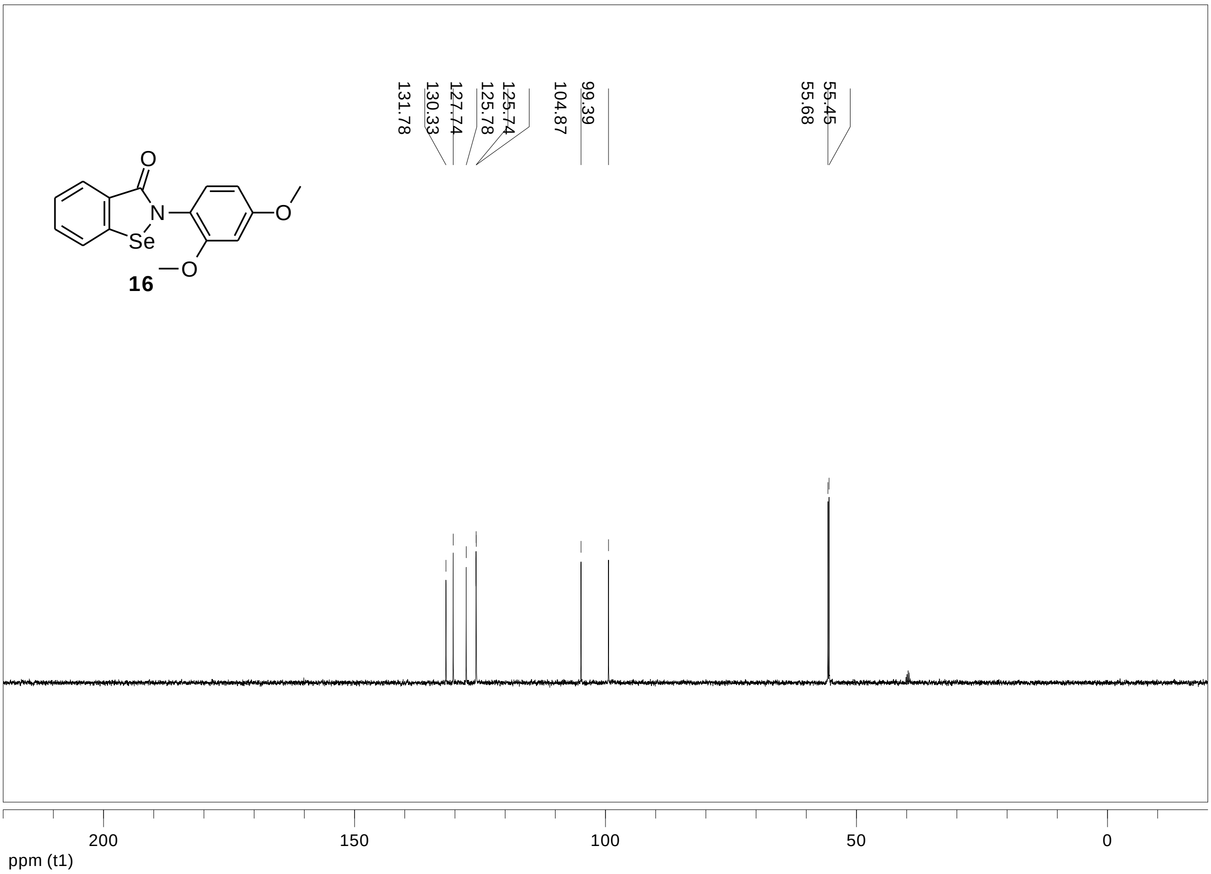

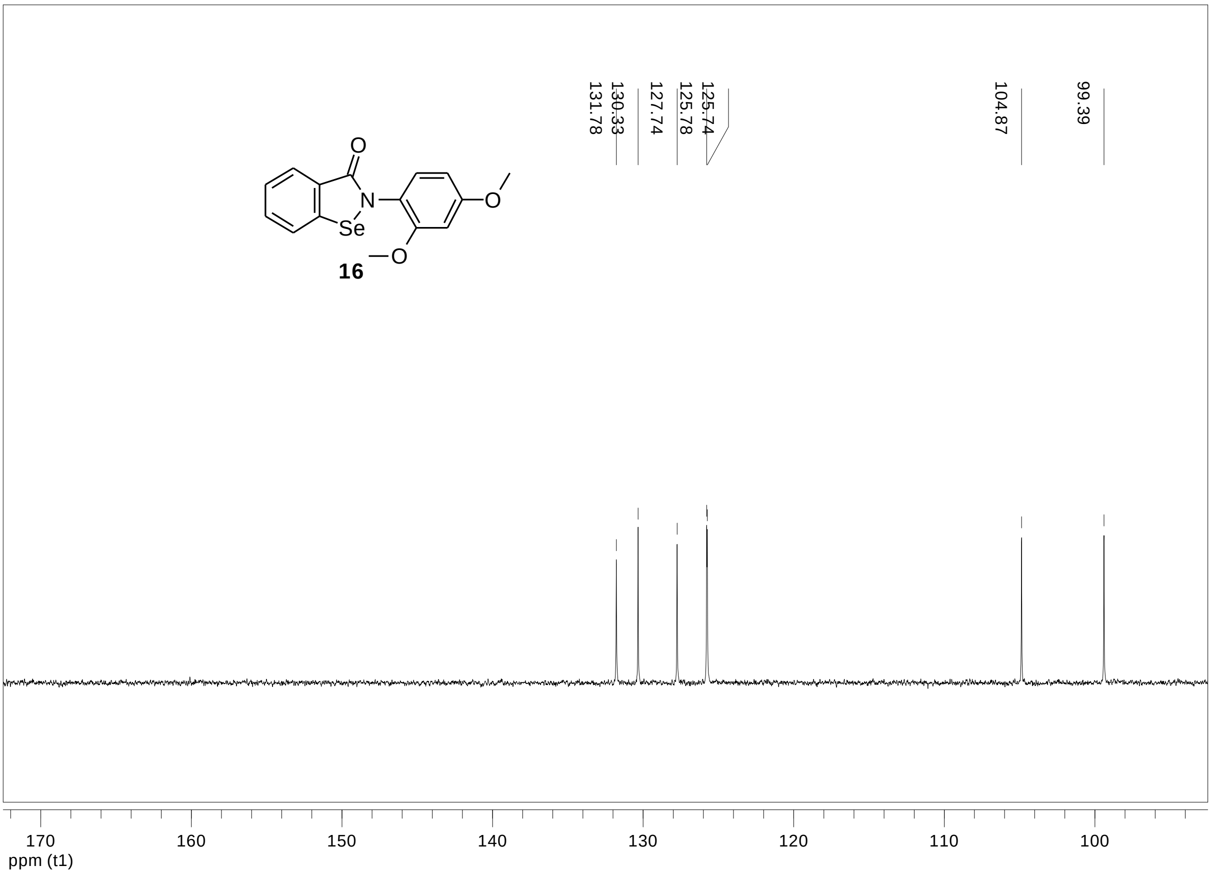

Fig. S82. ^{13}C-NMR (100.5 MHz, DMSO-d_6) dept-135 experiment of compound **16**

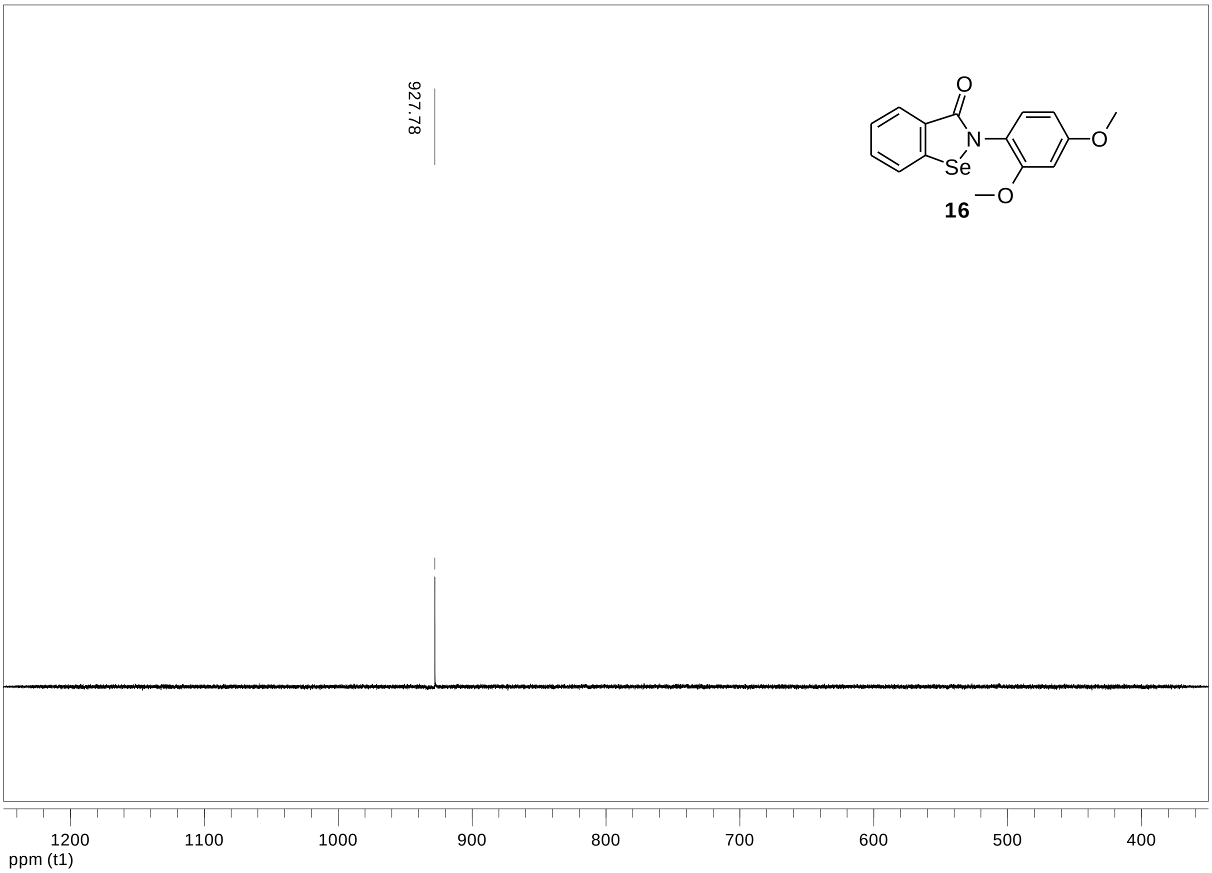

Fig. S83. ^{77}Se-NMR (76.24 MHz, DMSO-d_6) spectrum of compound **16**

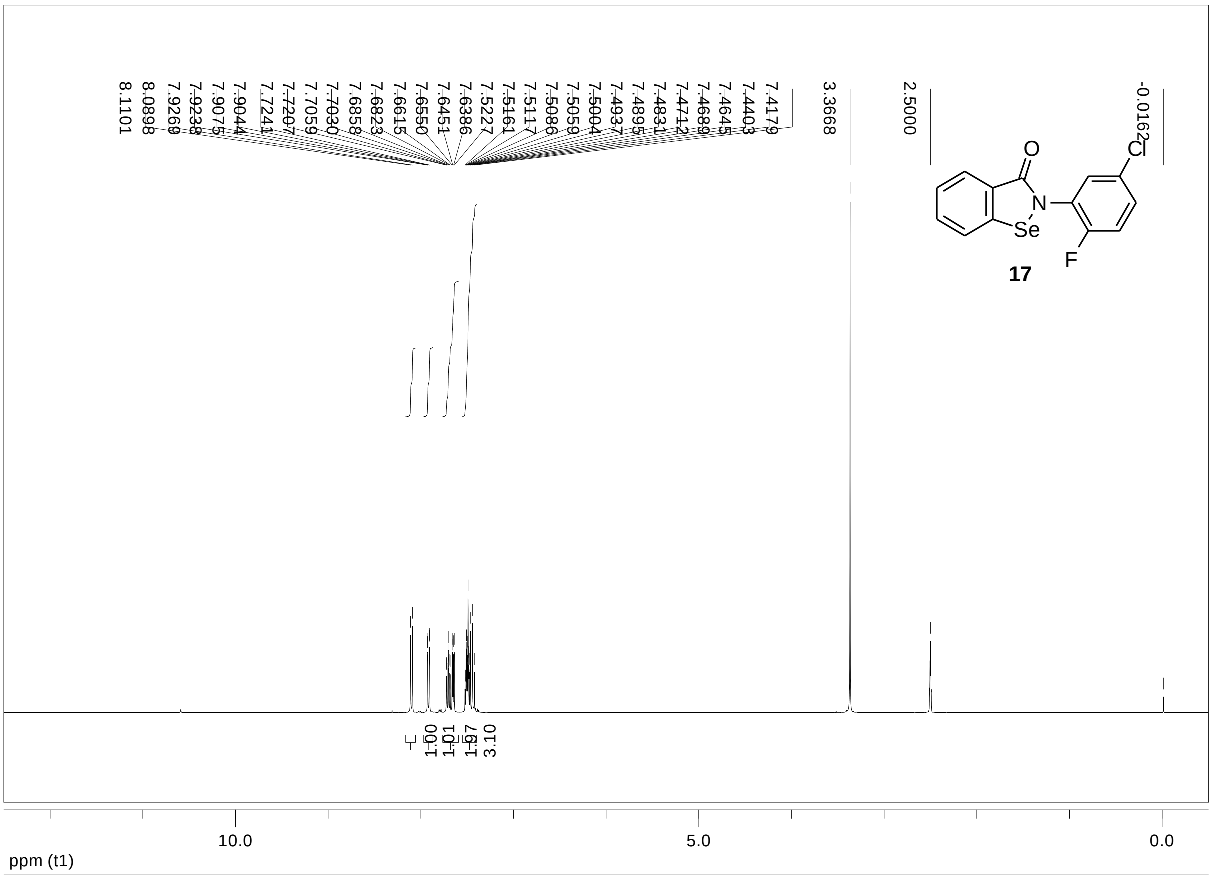

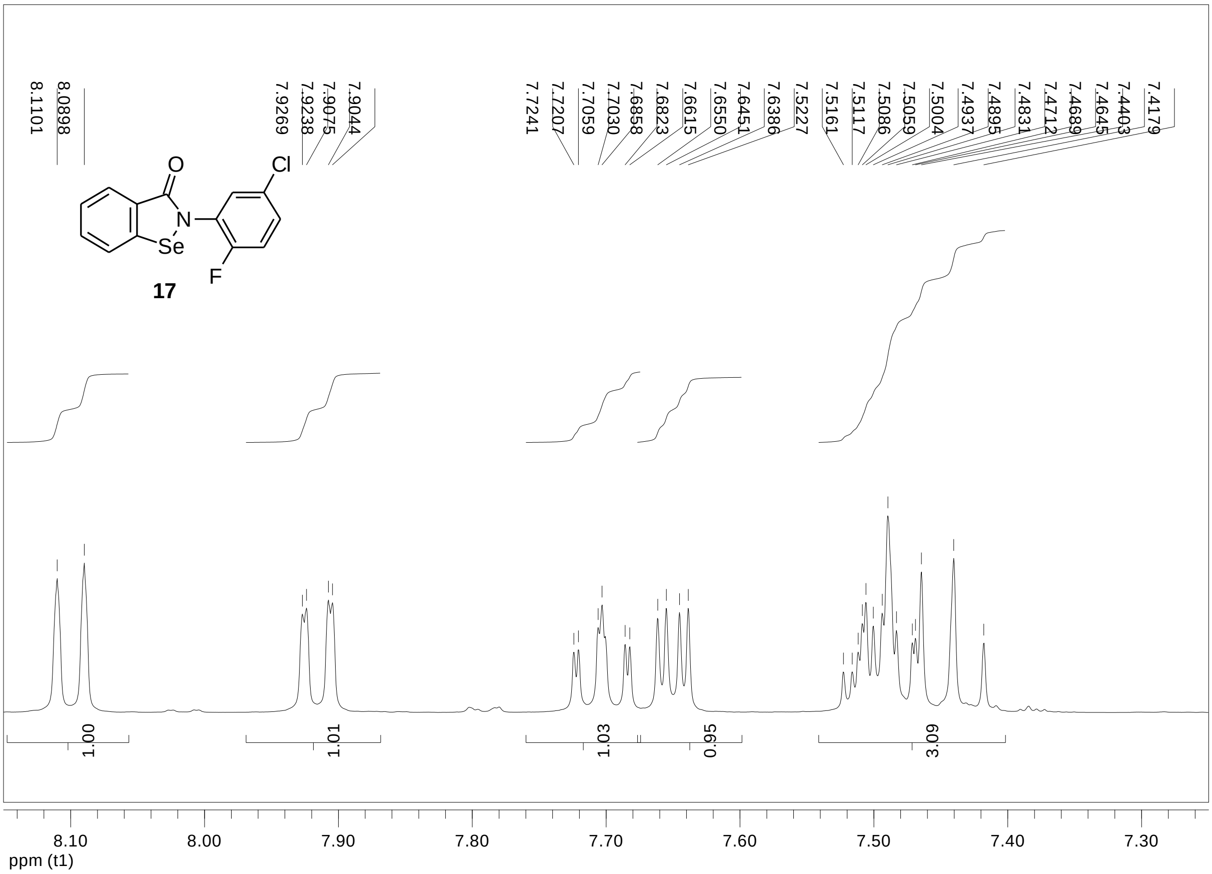

Fig. S84. ^{1}H-NMR (399.8 MHz, DMSO-d_6) spectrum of compound **17**

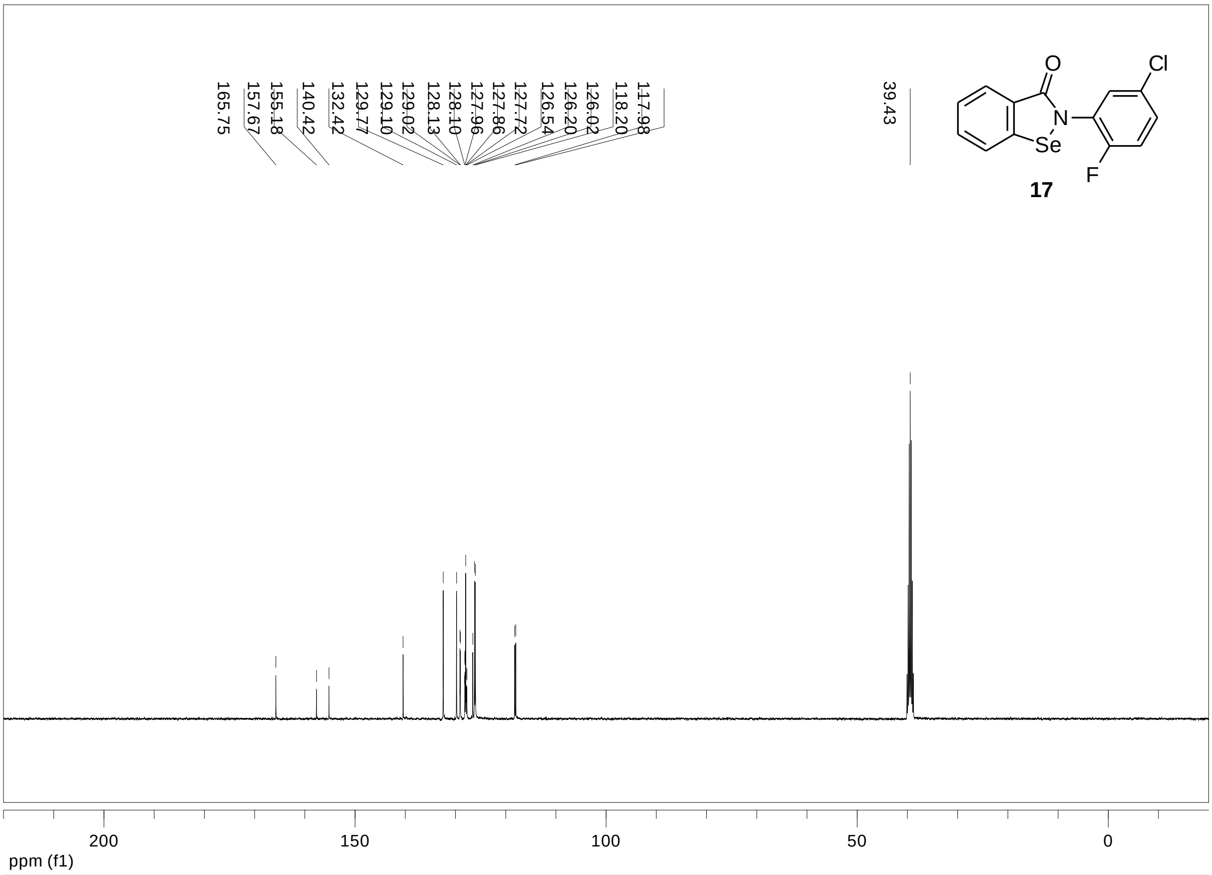

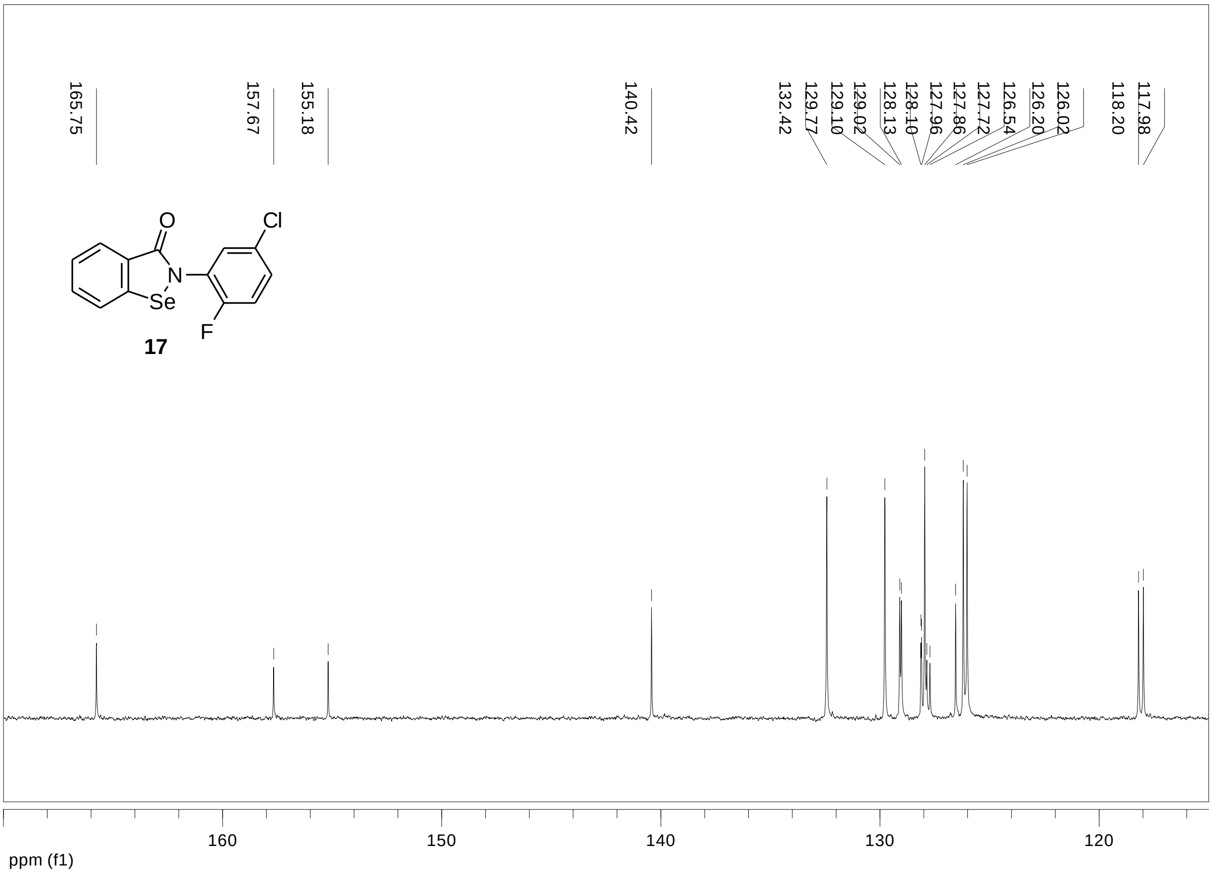

Fig. S85. ^{13}C-NMR (100.5 MHz, DMSO-d_6) spectrum of compound **17**

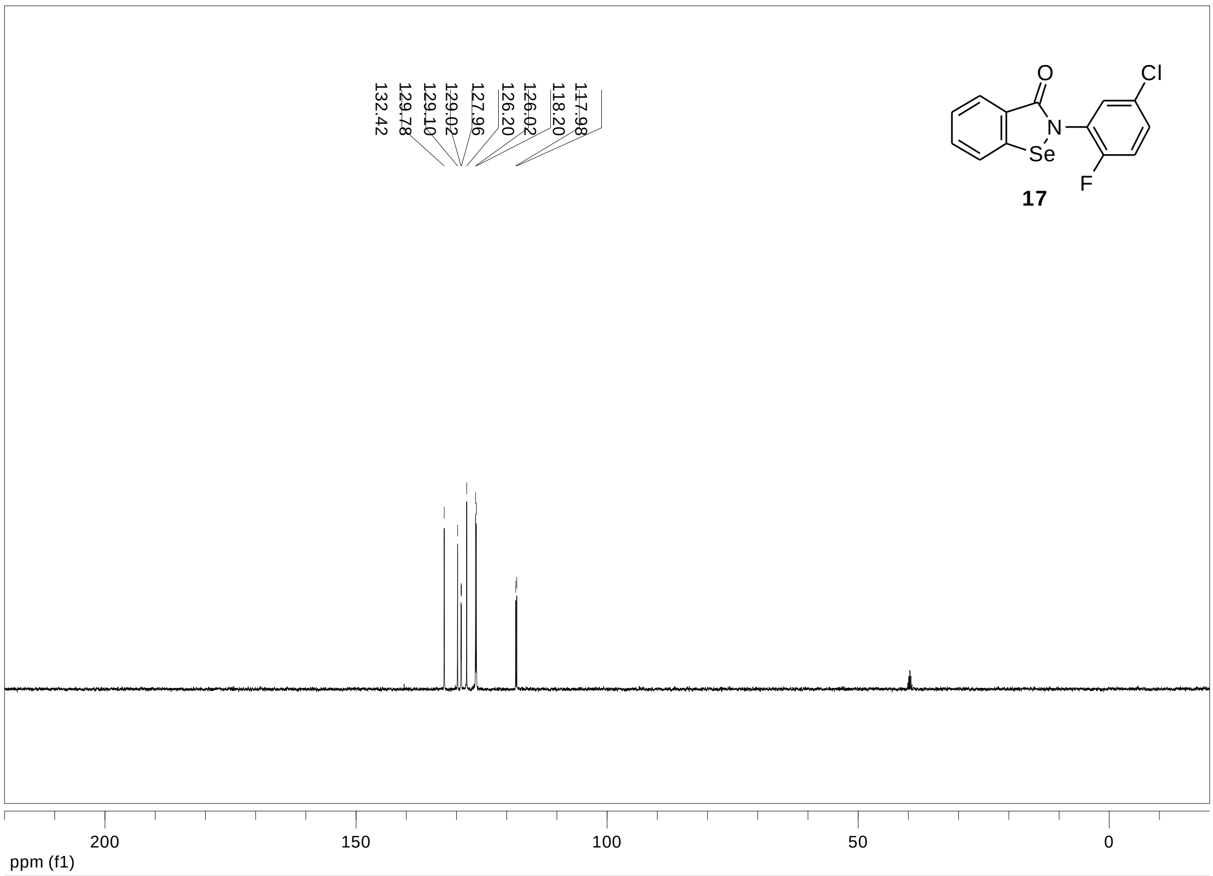

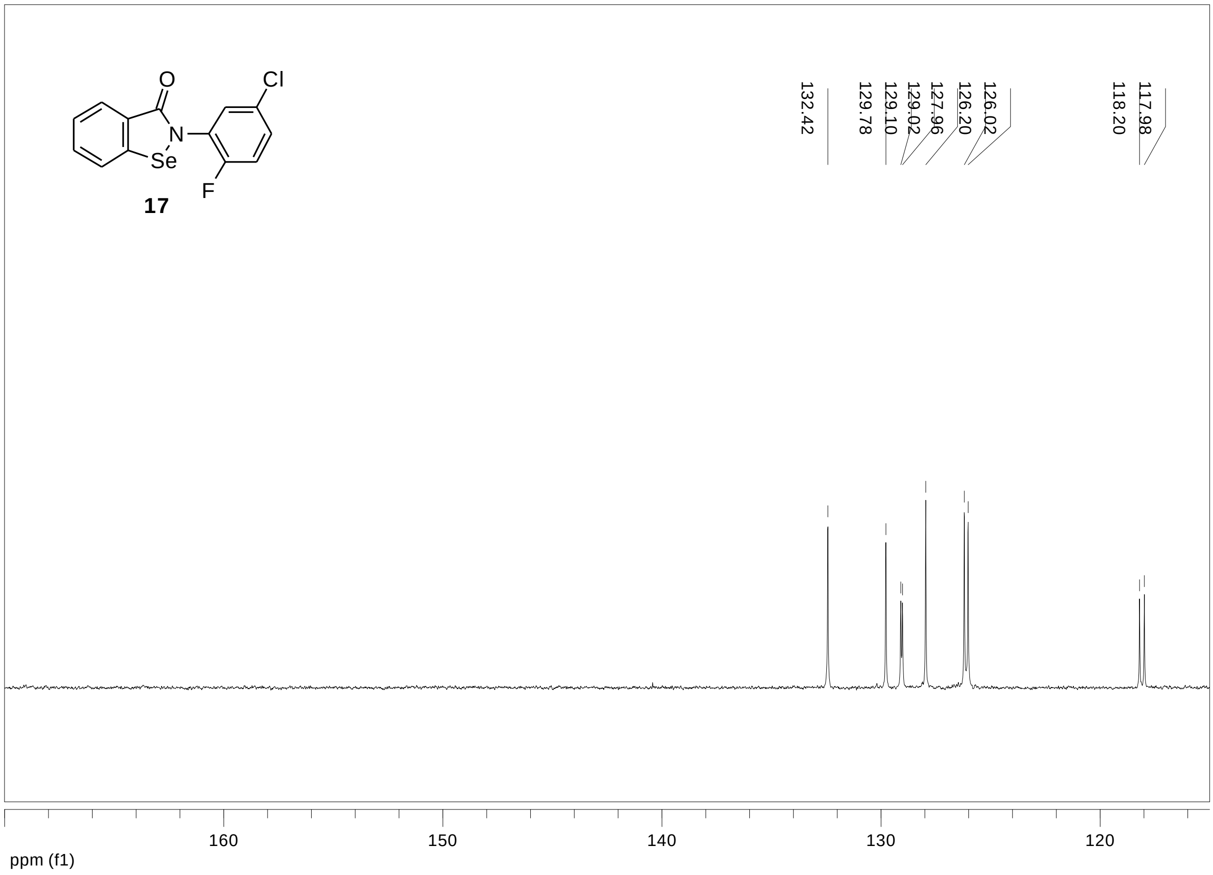

Fig. S86. ^{13}C-NMR (100.5 MHz, DMSO-d_6) dept-135 experiment of compound **17**

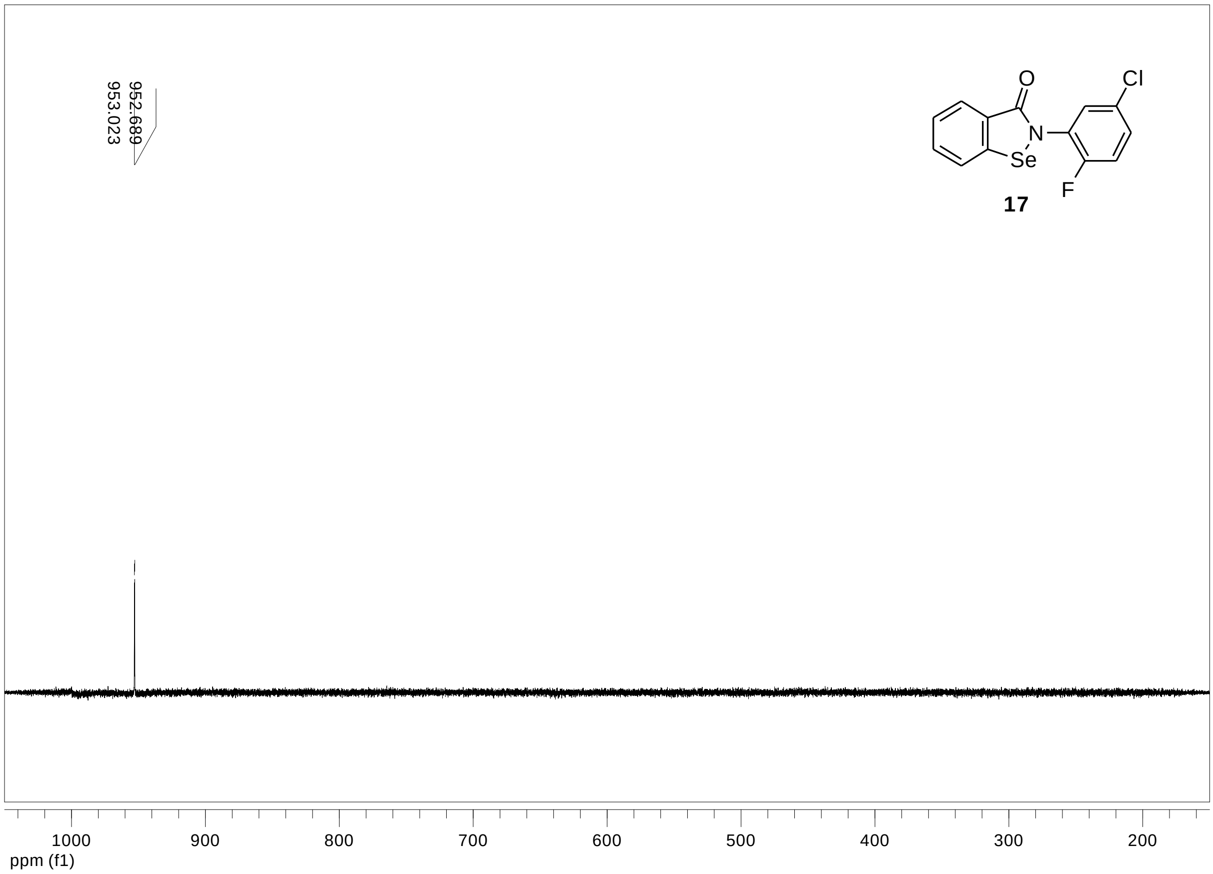

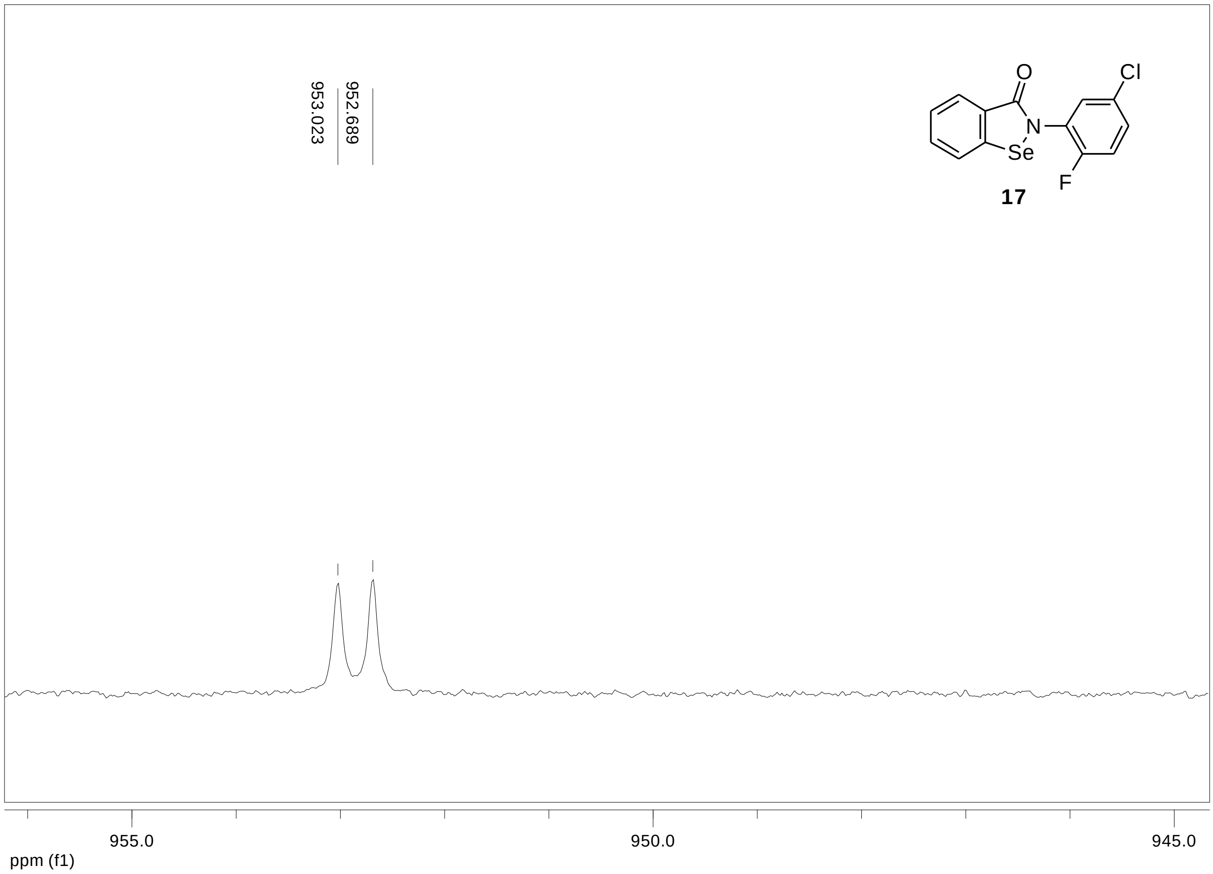

Fig. S87. ^{77}Se-NMR (76.24 MHz, DMSO-d_6) spectrum of compound **17**

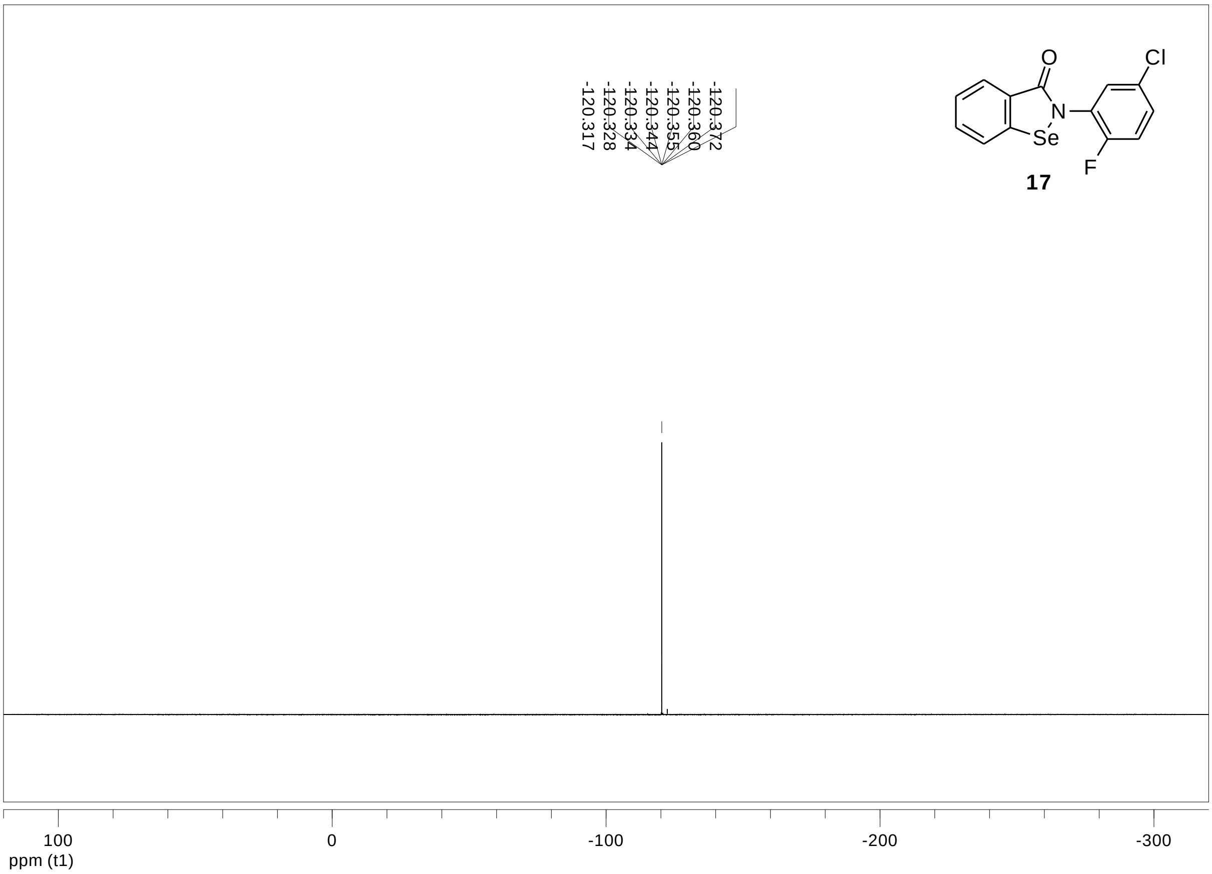

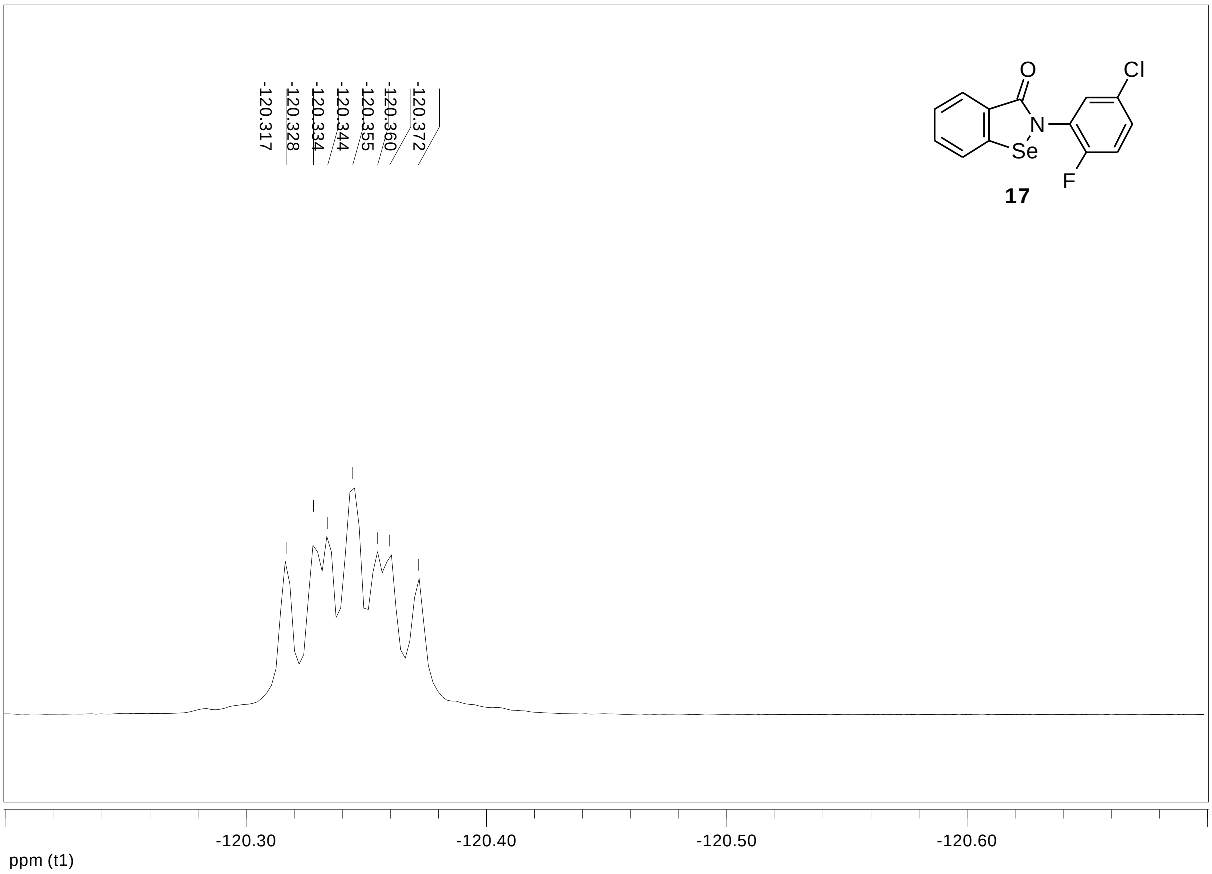

Fig. S88. ^{19}F-NMR (376.2 MHz, DMSO-d_6) spectrum of compound **17**

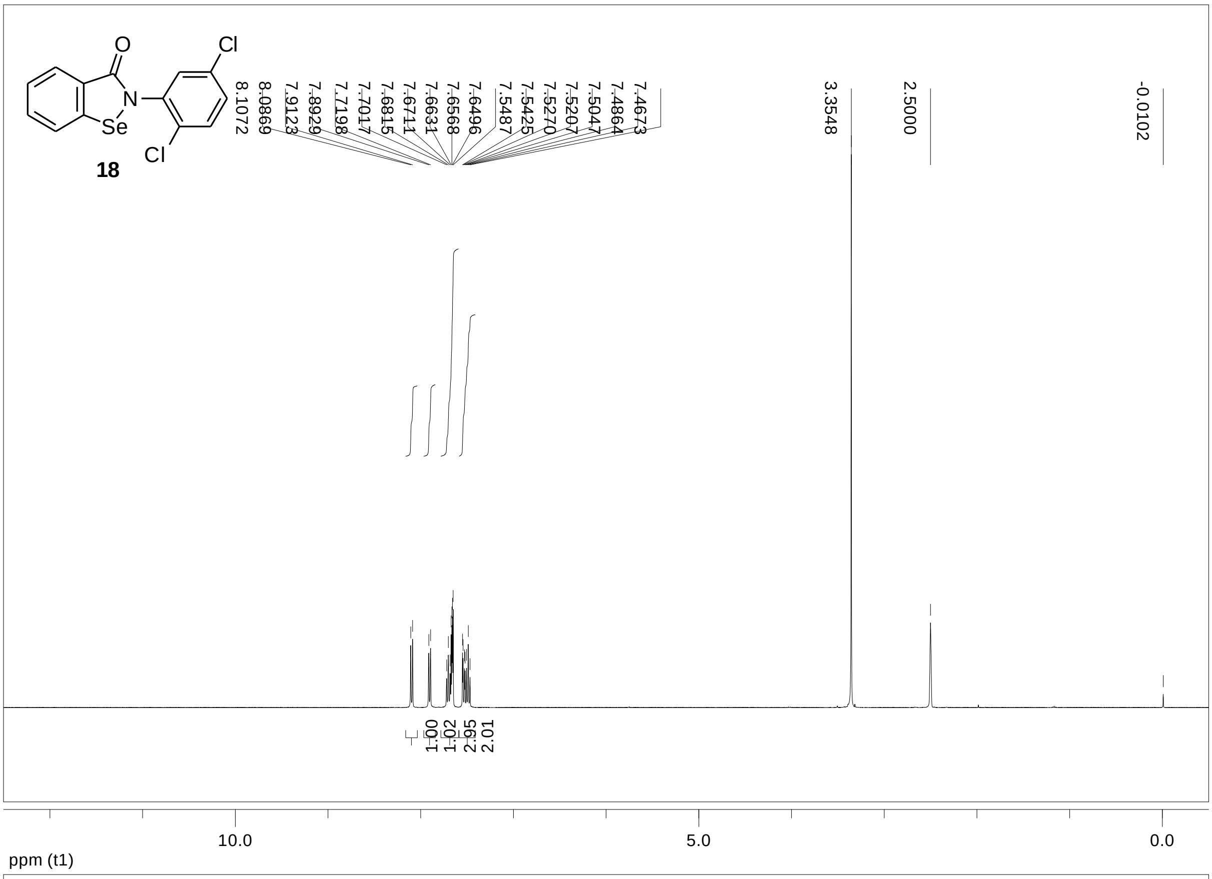

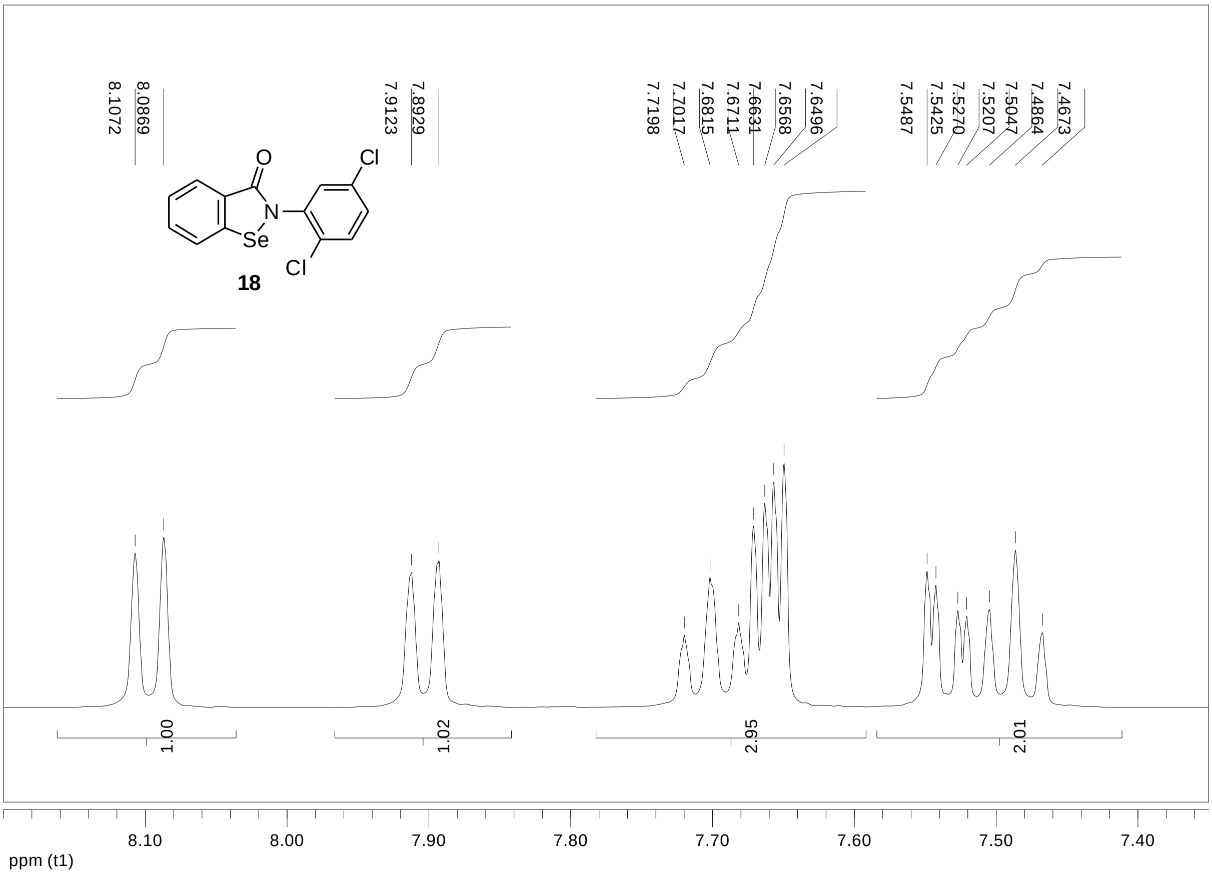

Fig. S89. ^{1}H-NMR (399.8 MHz, DMSO-d_6) spectrum of compound **18**

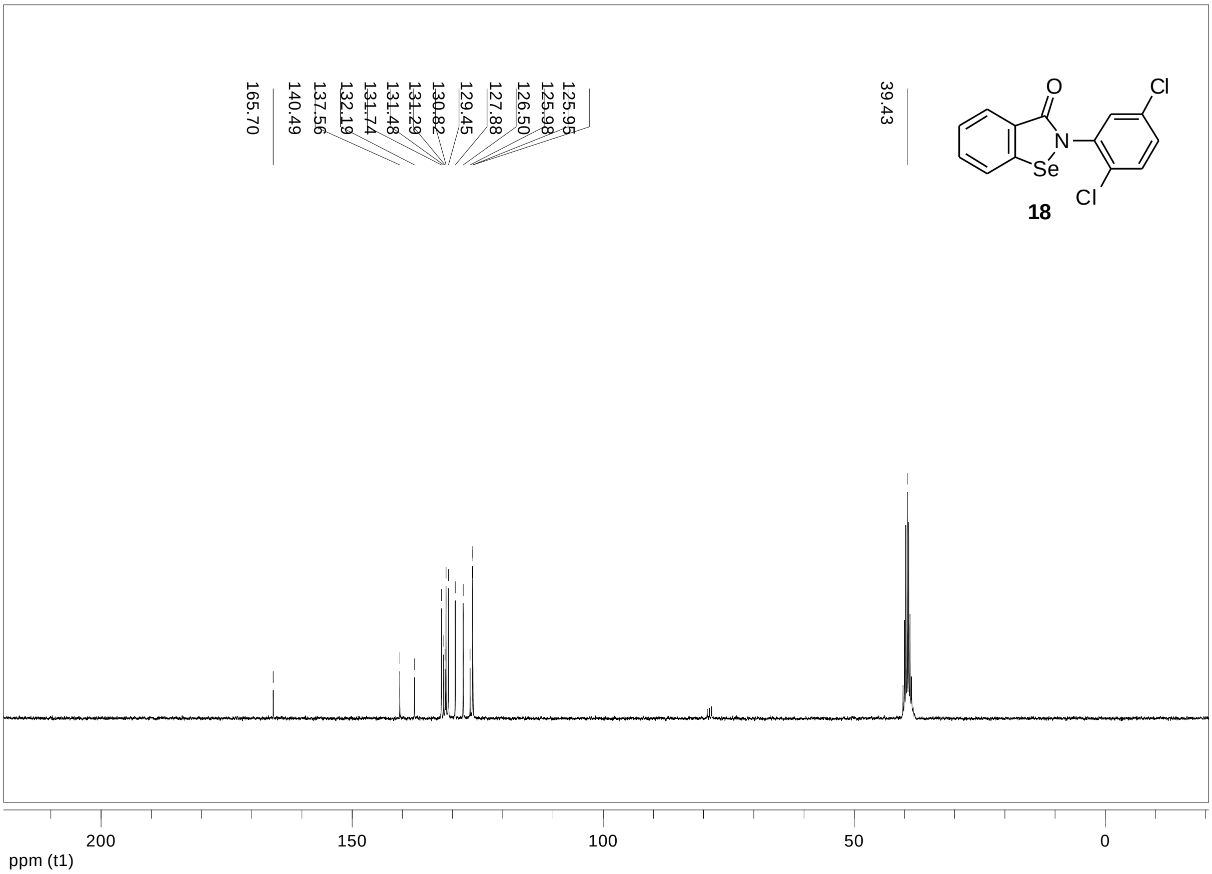

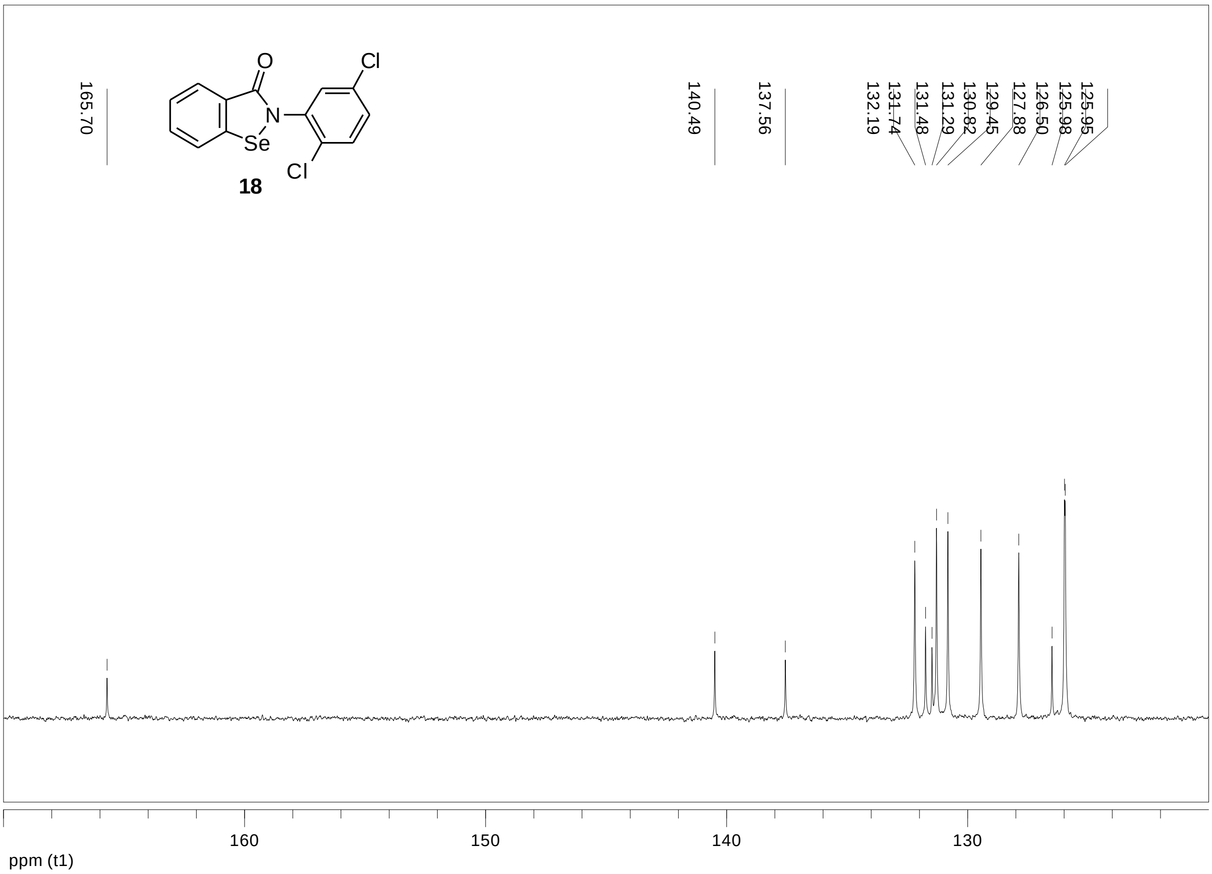

Fig. S90. ^{13}C-NMR (75.5 MHz, DMSO-d_6) spectrum of compound **18**

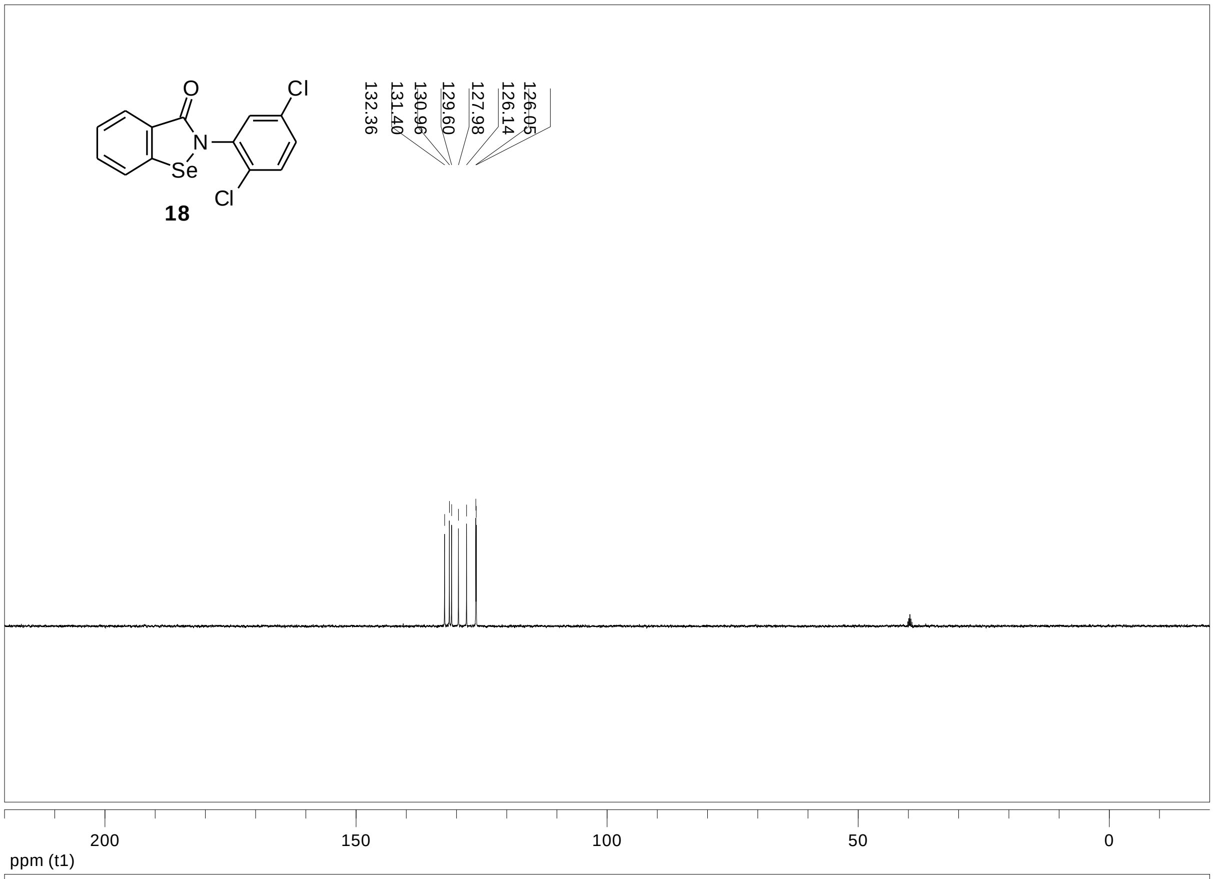

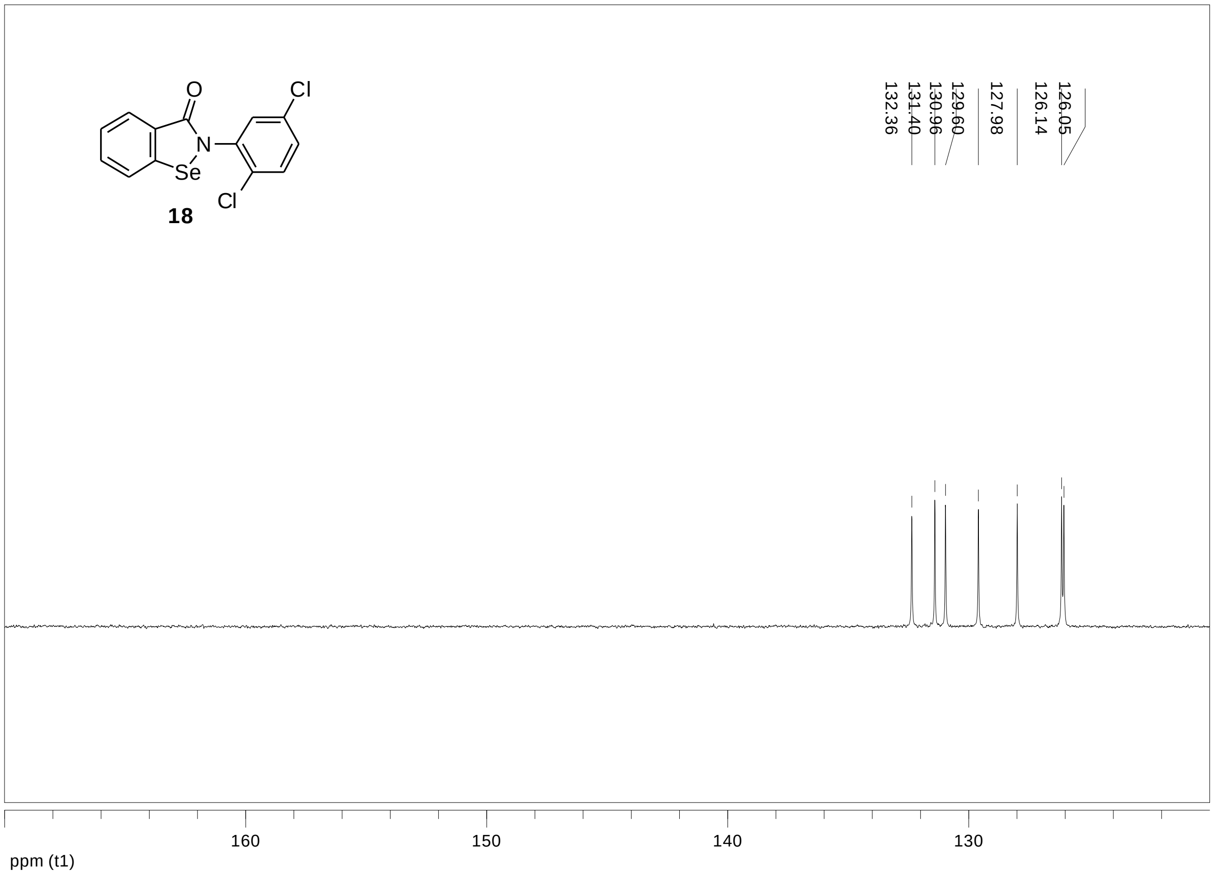

Fig. S91. ^{13}C-NMR (100.5 MHz, DMSO-d_6) dept-135 experiment of compound **18**

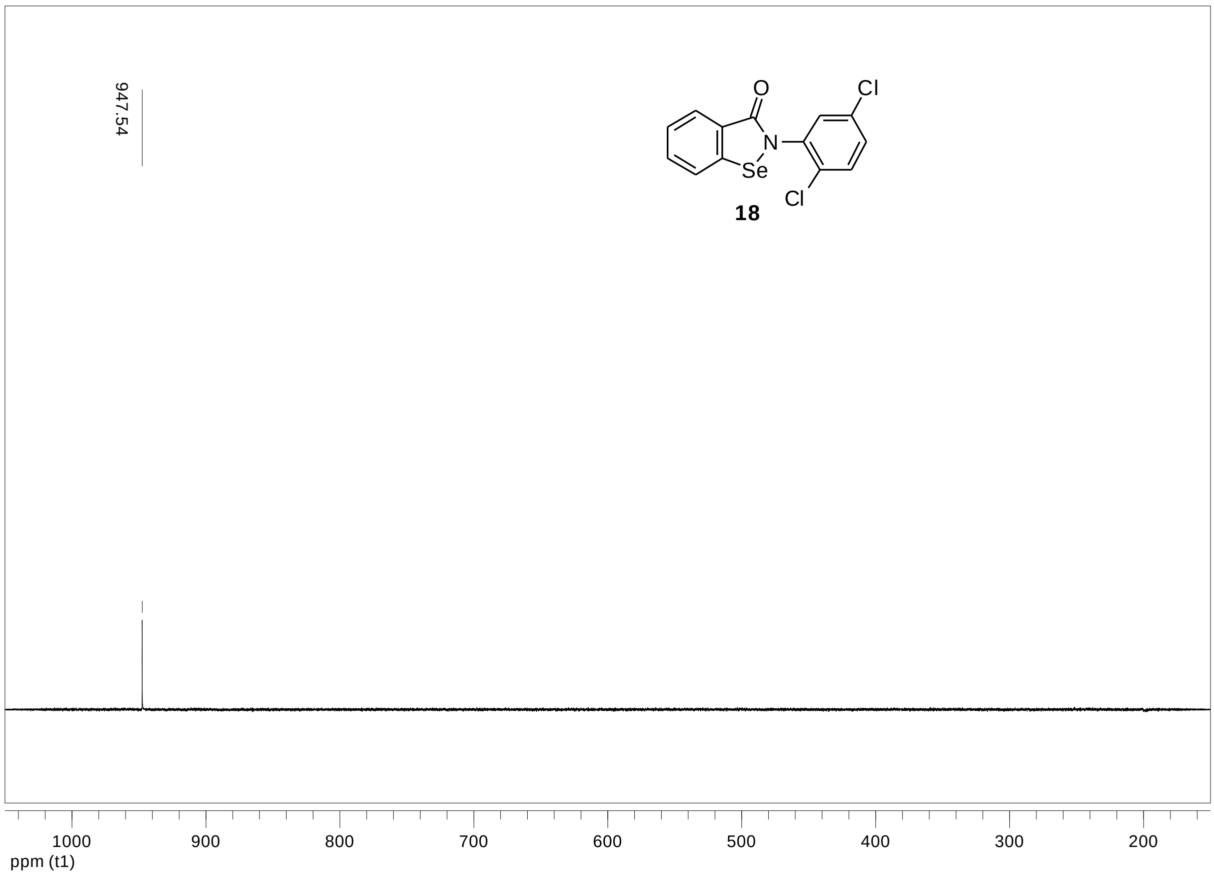

Fig. S92. ^{77}Se-NMR (76.24 MHz, DMSO-d_6) spectrum of compound **18**

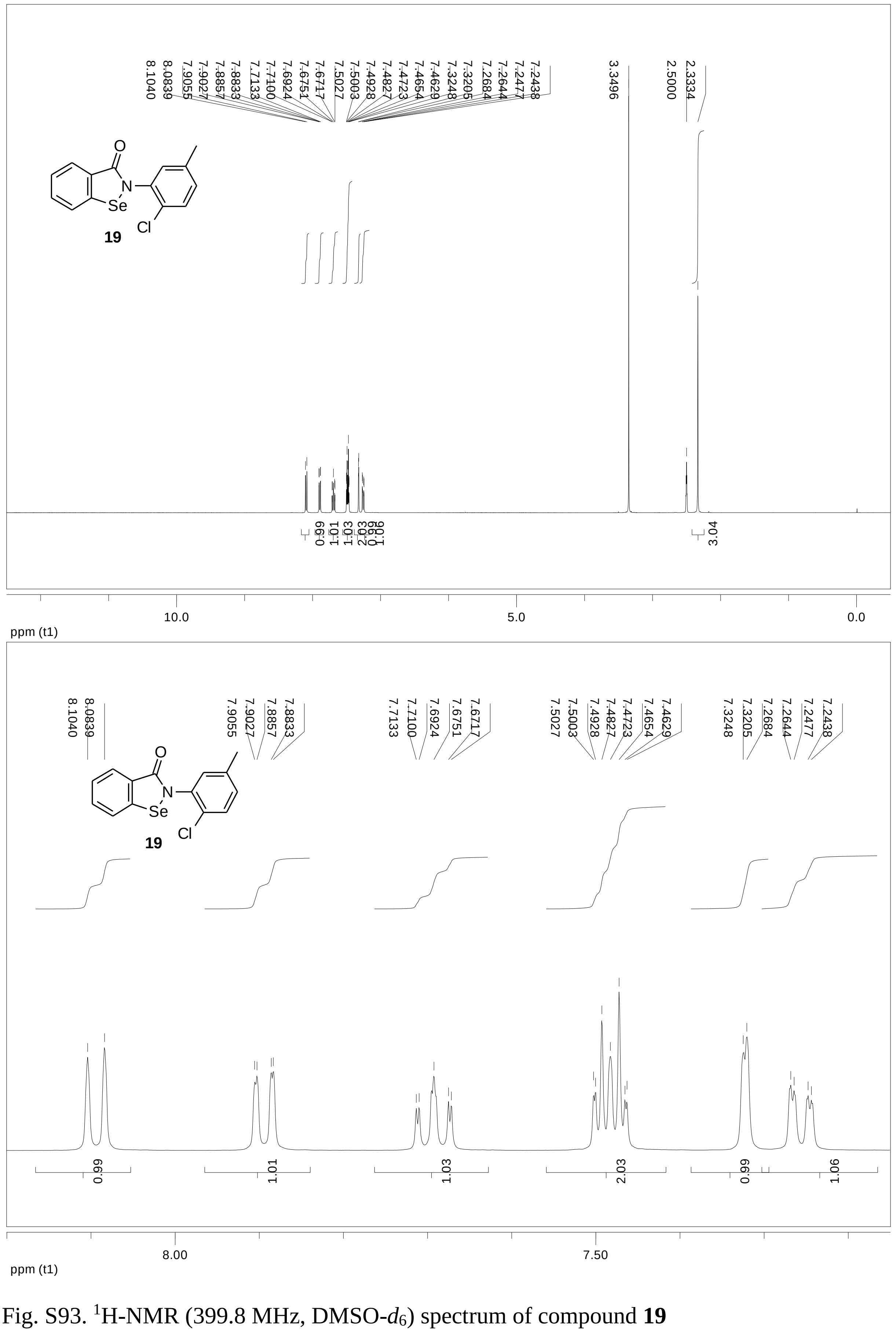

Fig. S93. ^{1}H-NMR (399.8 MHz, DMSO-d_6) spectrum of compound **19**

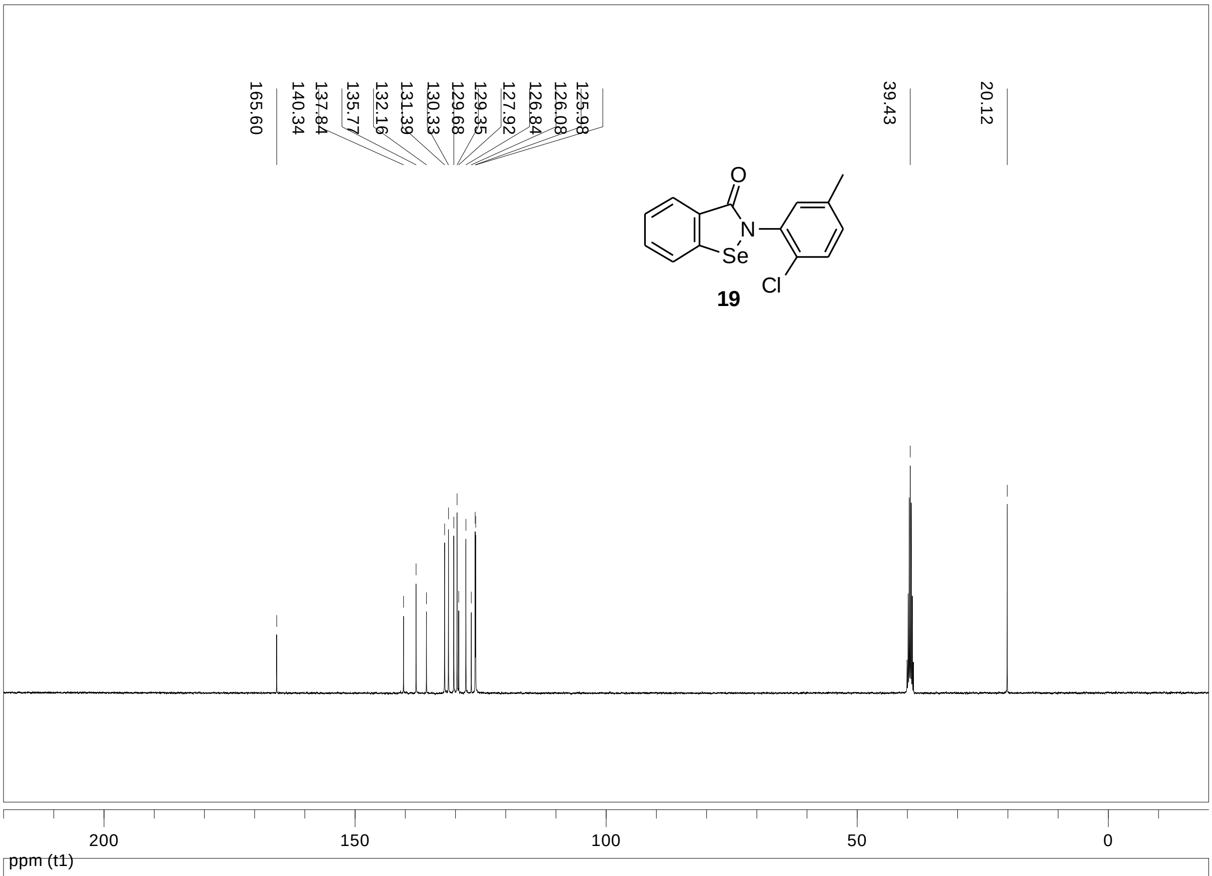

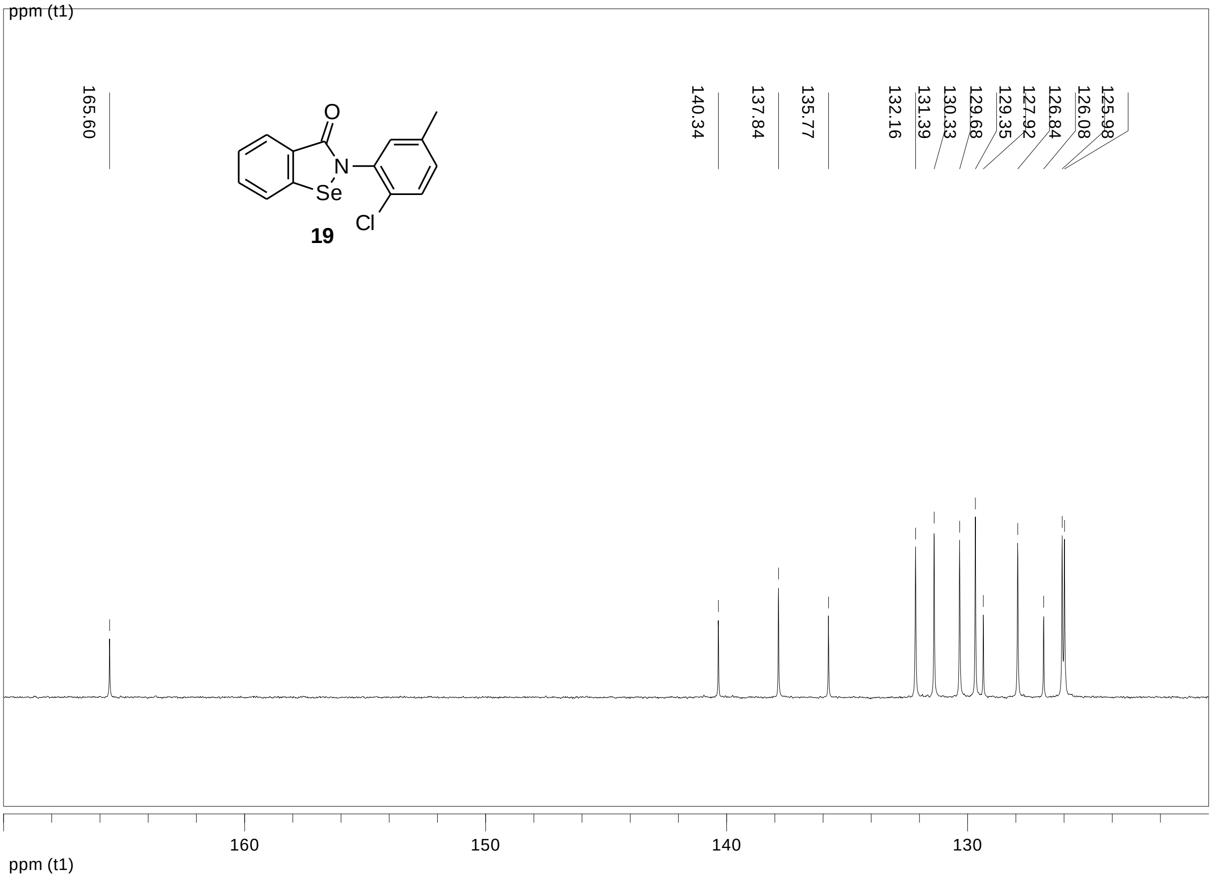

Fig. S94. ^{13}C-NMR (100.5 MHz, DMSO-d_6) spectrum of compound **19**

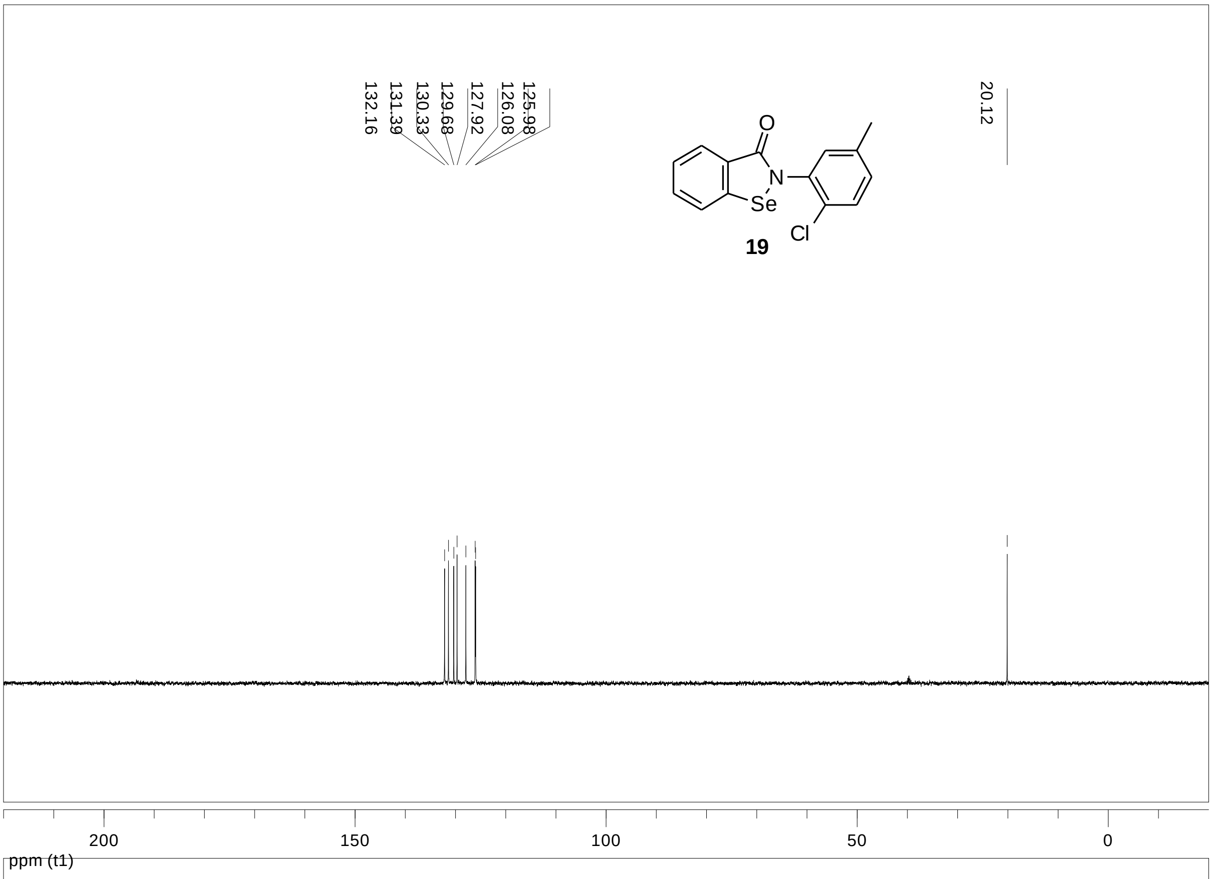

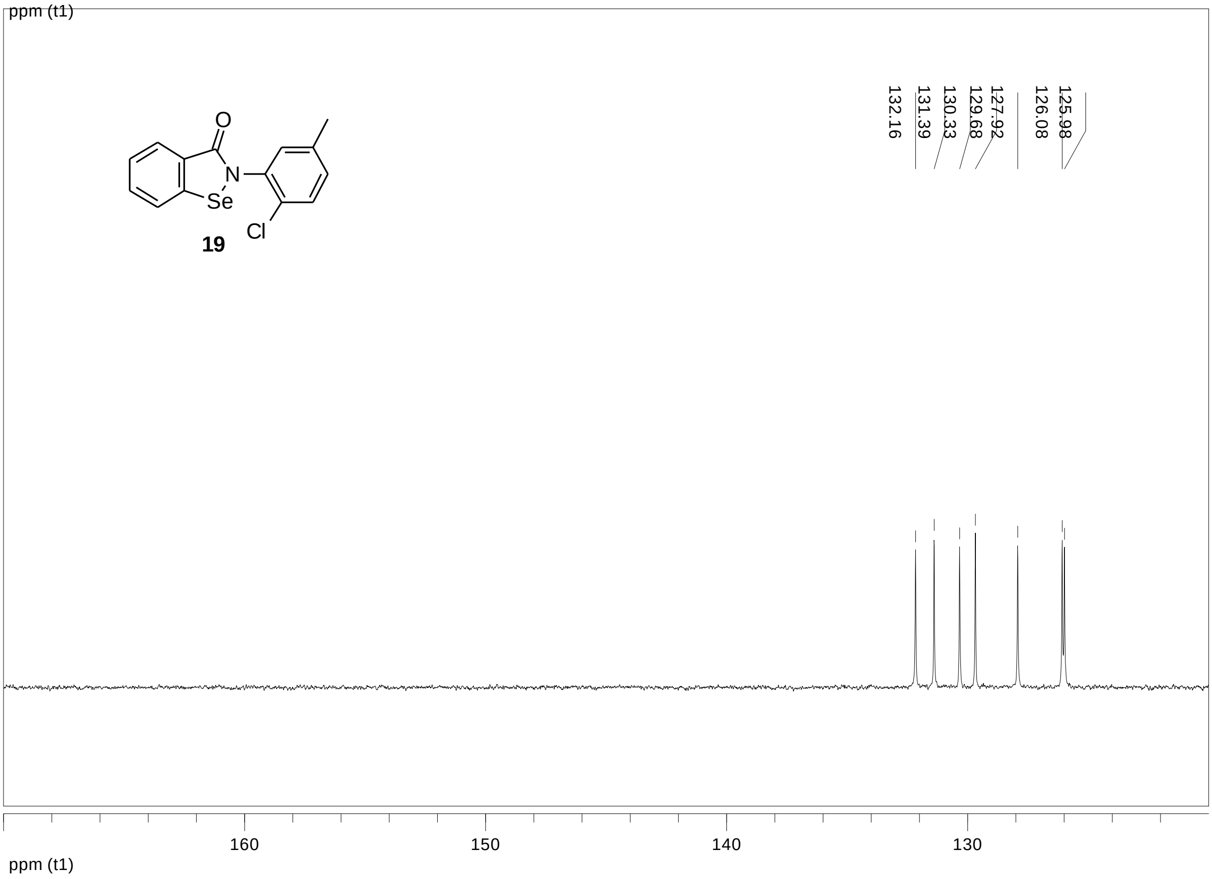

Fig. S95. ^{13}C-NMR (100.5 MHz, DMSO-d_6) dept-135 experiment of compound **19**

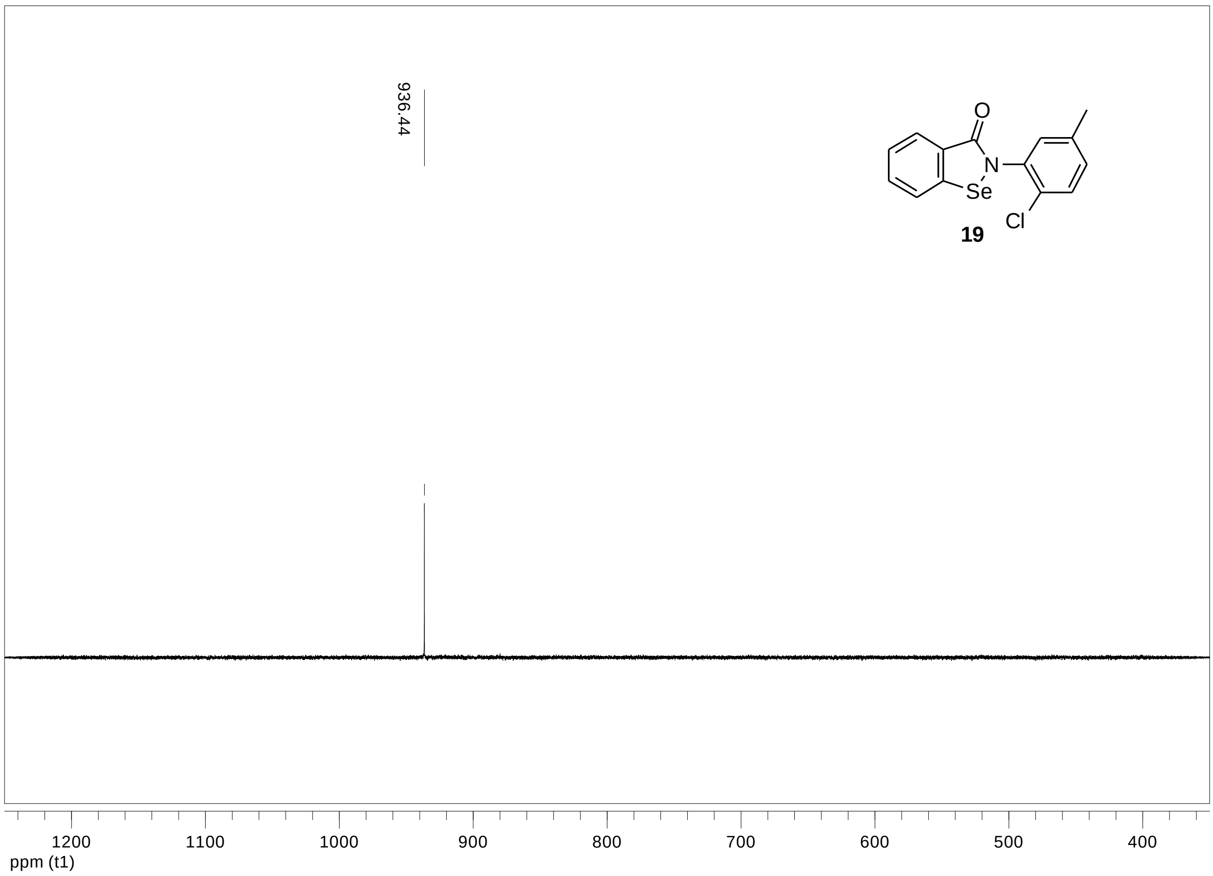

Fig. S96. ^{77}Se-NMR (76.24 MHz, DMSO-d_6) spectrum of compound **19**

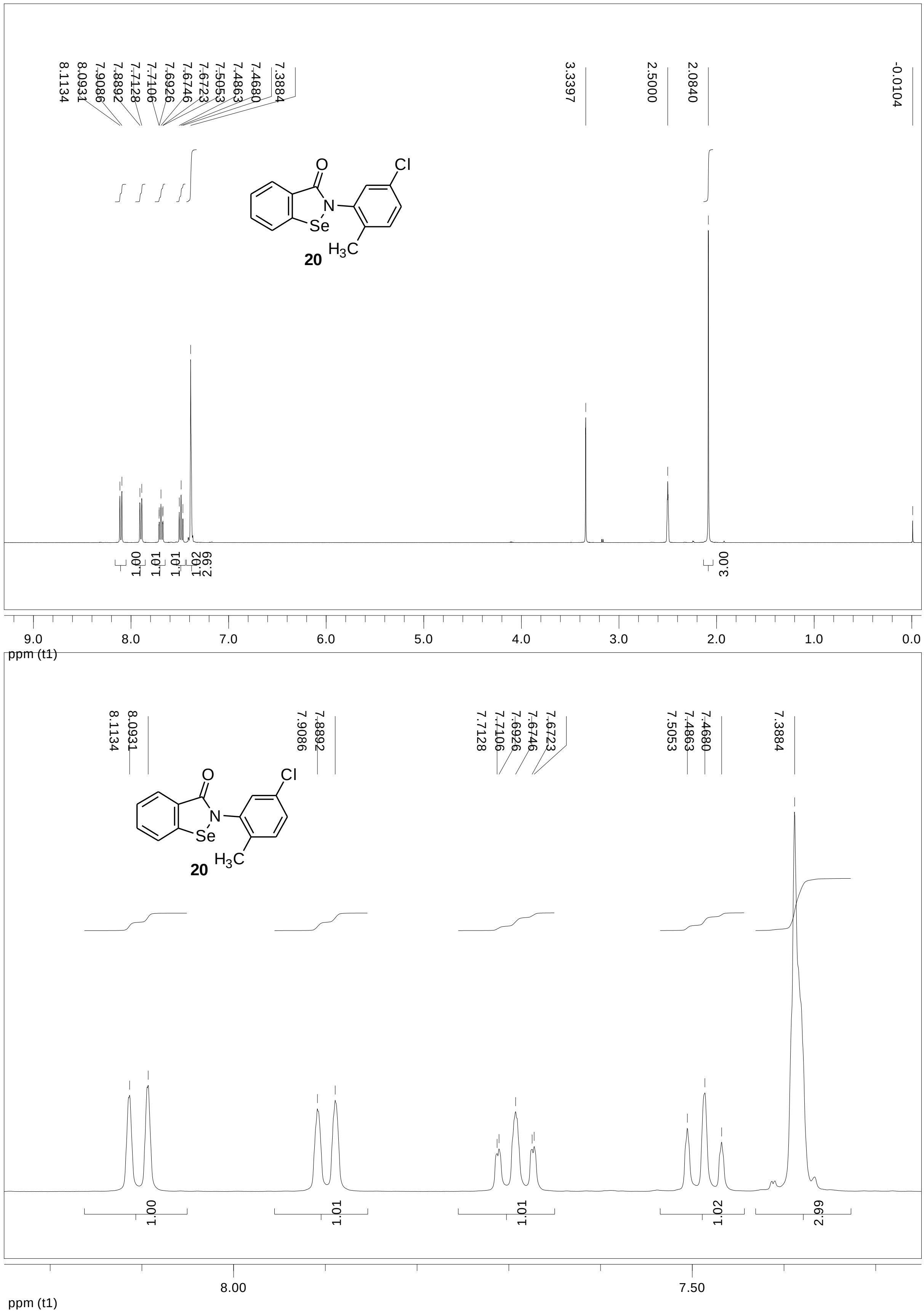

Fig. S97. ^{1}H-NMR (399.8 MHz, DMSO-d_6) spectrum of compound **20**

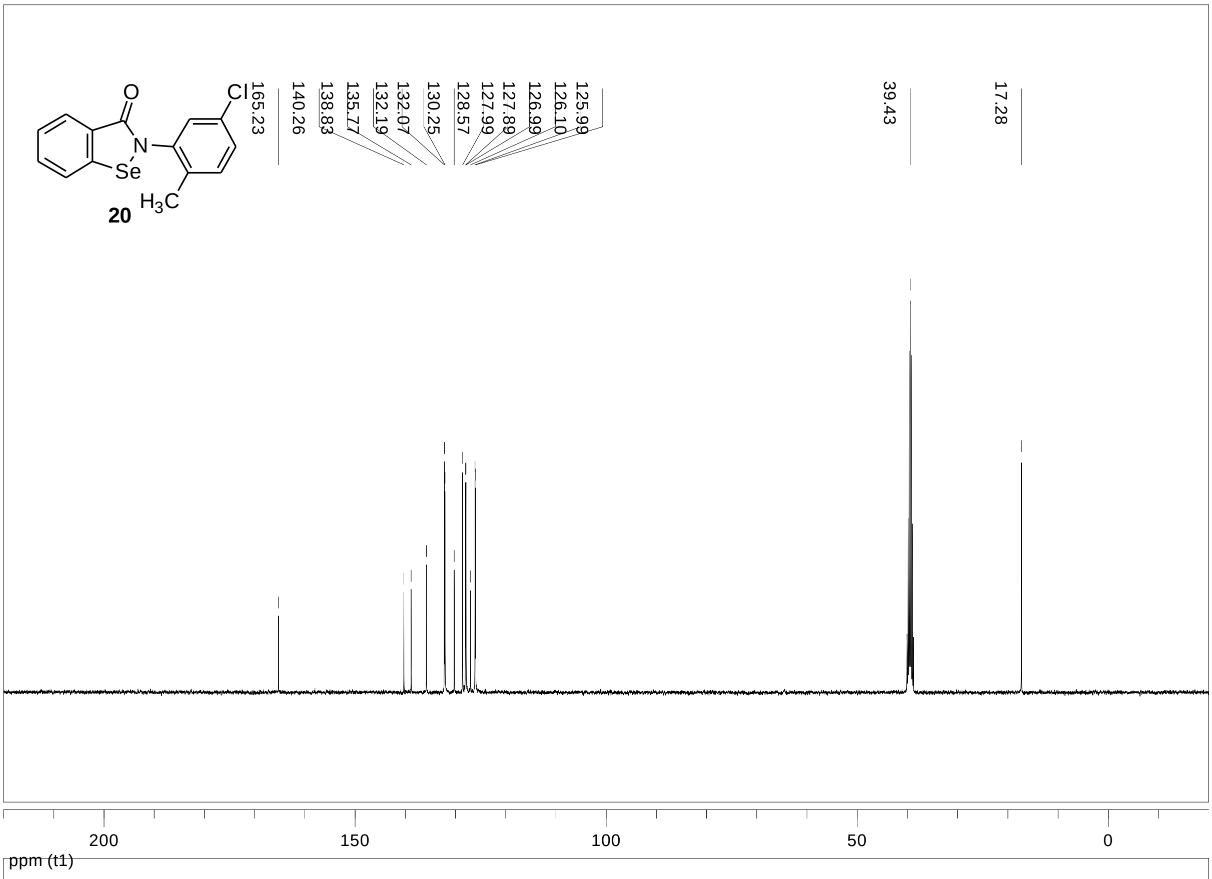

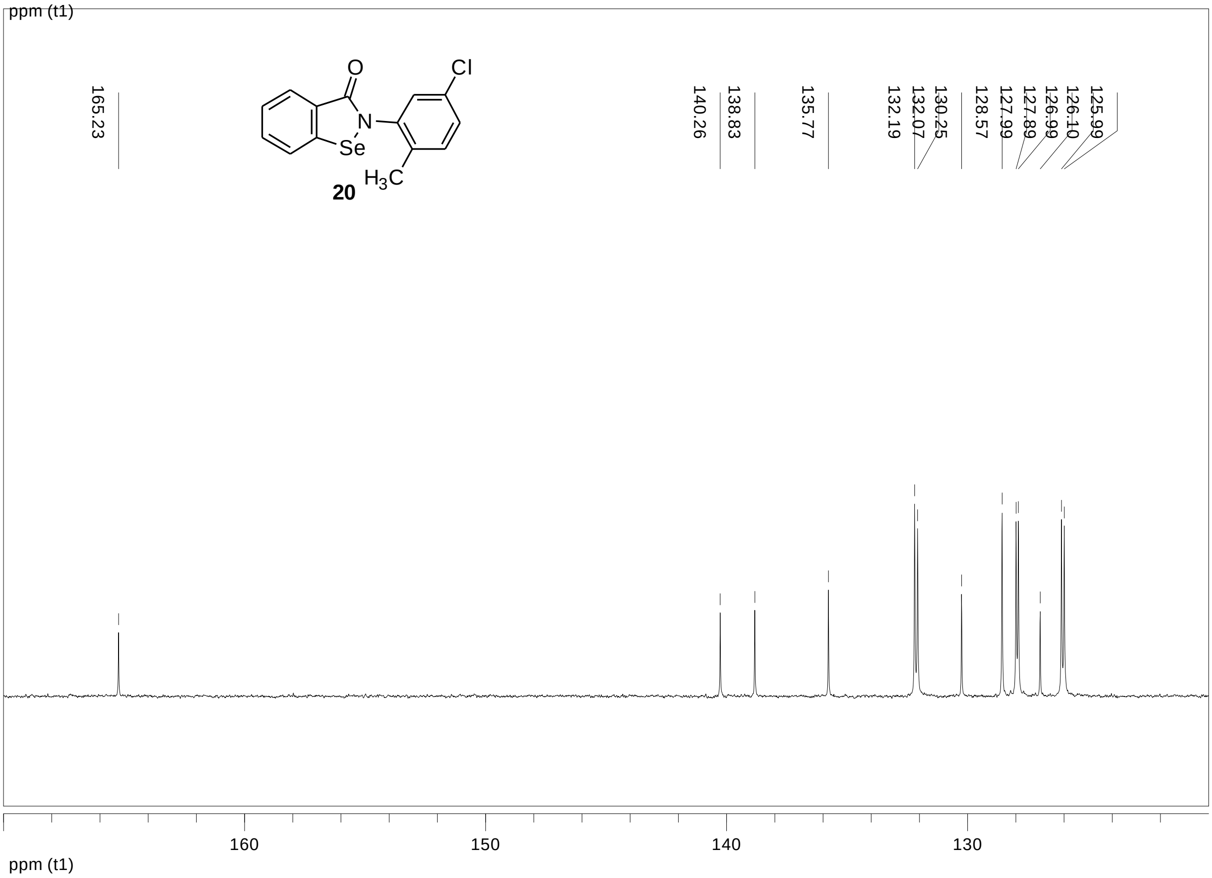

Fig. S98. ^{13}C-NMR (100.5 MHz, DMSO-d_6) spectrum of compound **20**

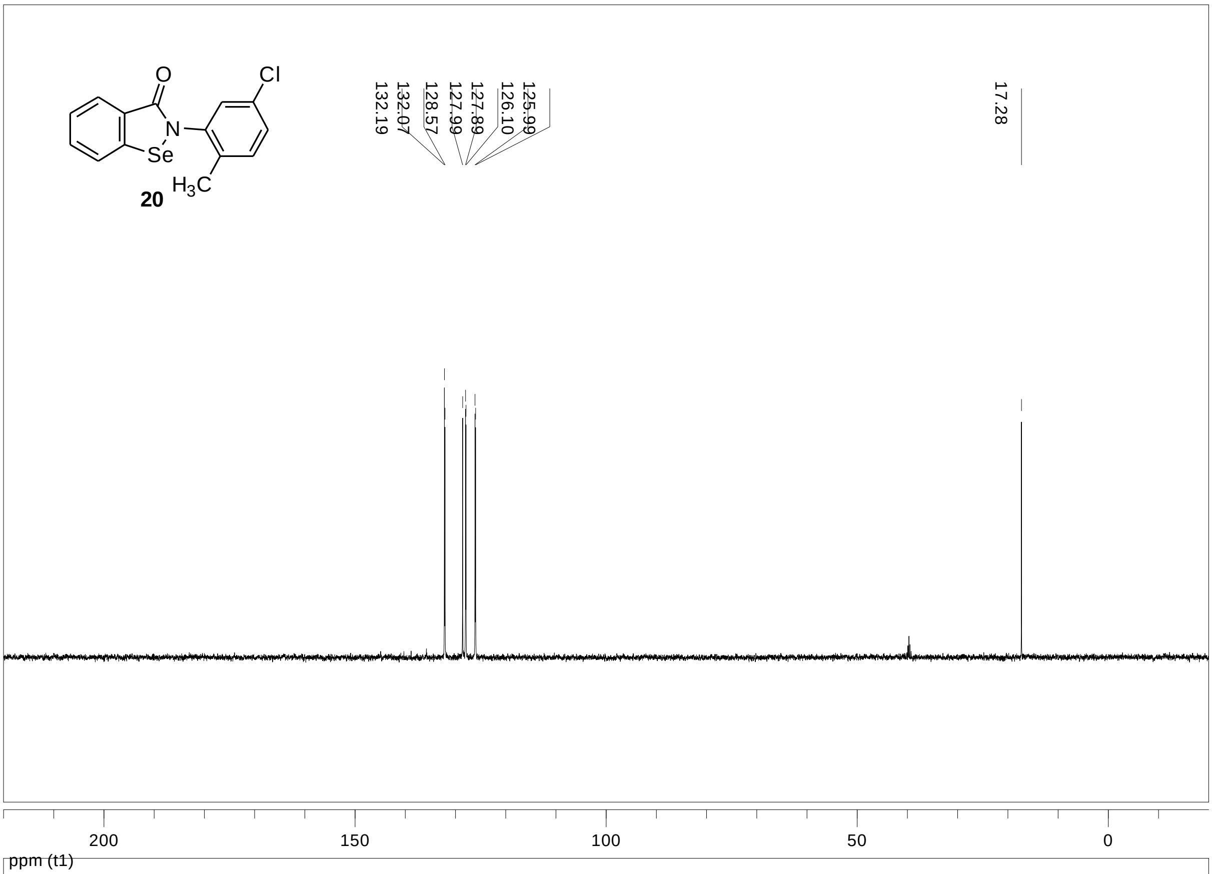

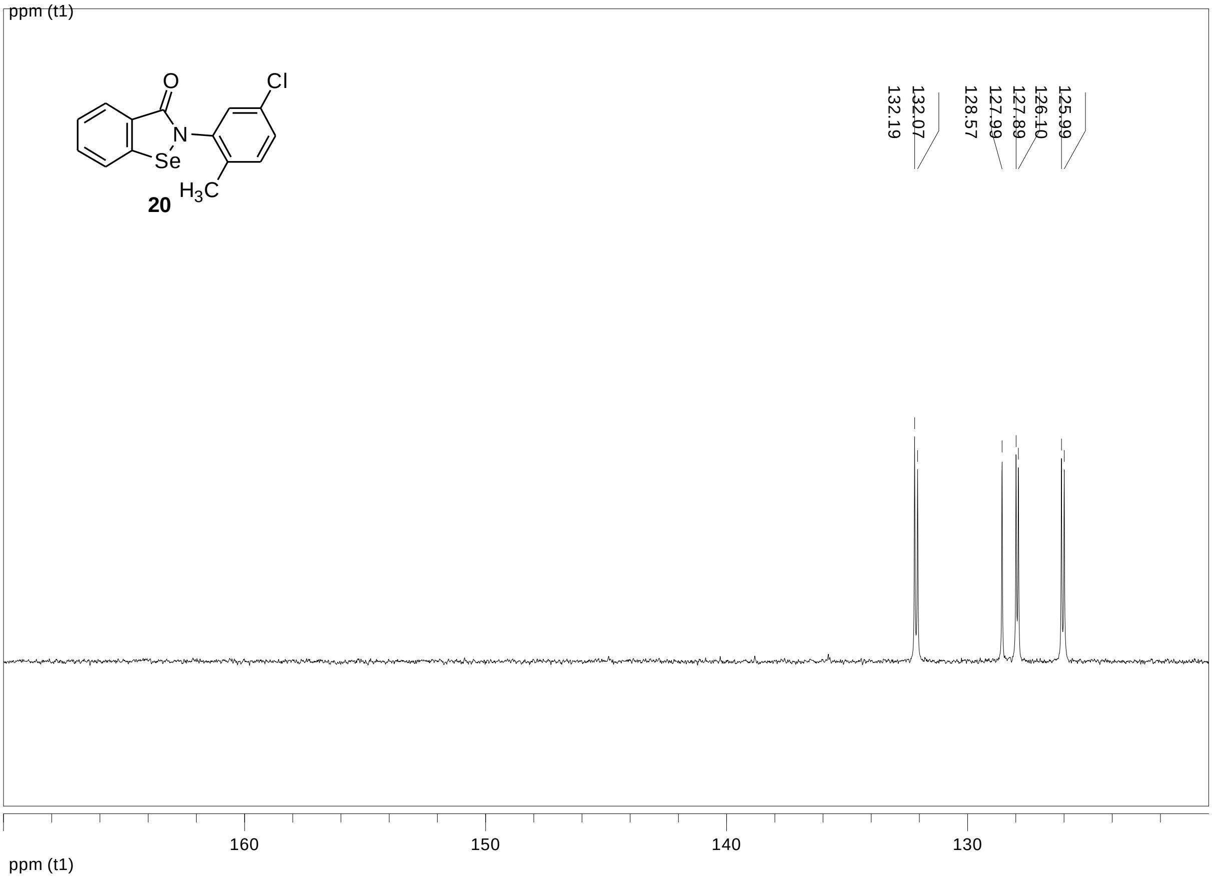

Fig. S99. ^{13}C-NMR (100.5 MHz, DMSO-d_6) dept-135 experiment of compound **20**

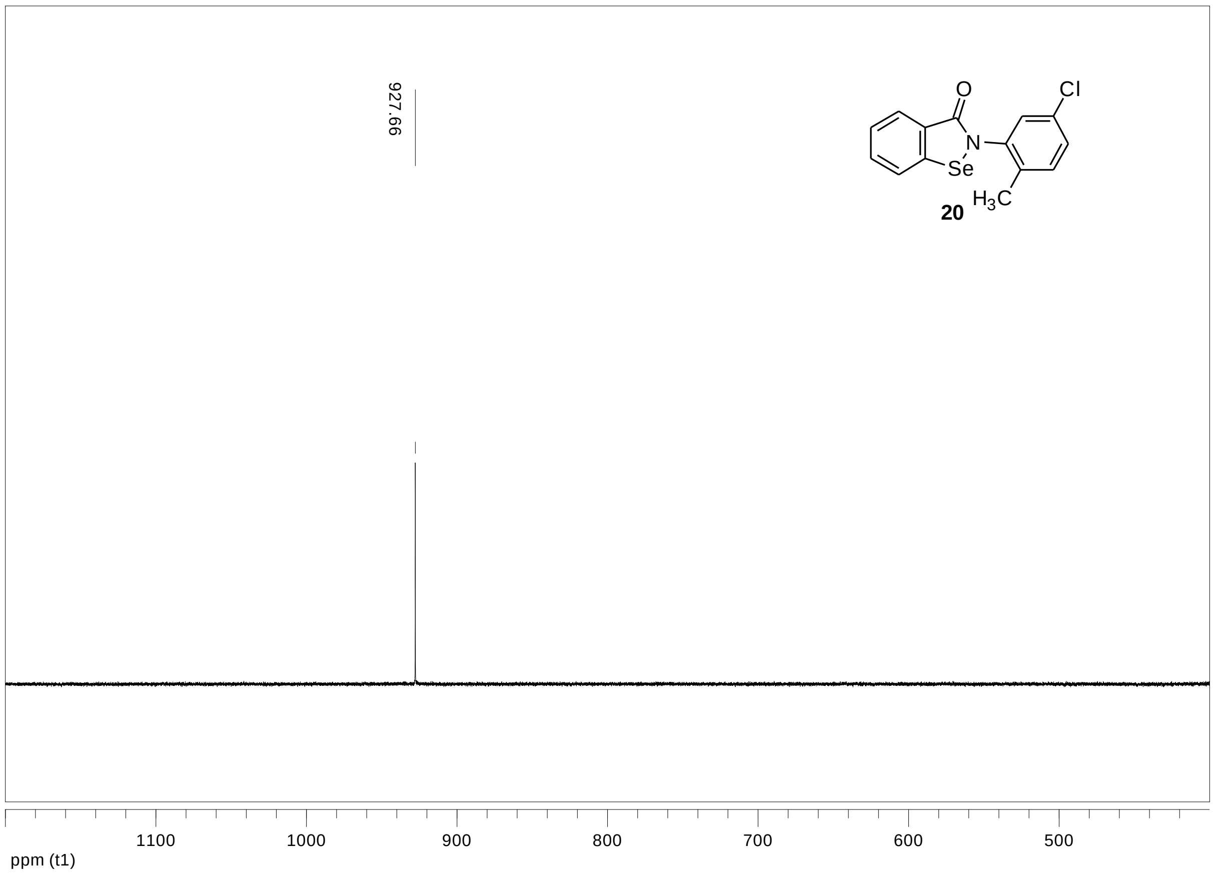

Fig. S100. ^{77}Se-NMR (76.24 MHz, DMSO-d_6) spectrum of compound **20**

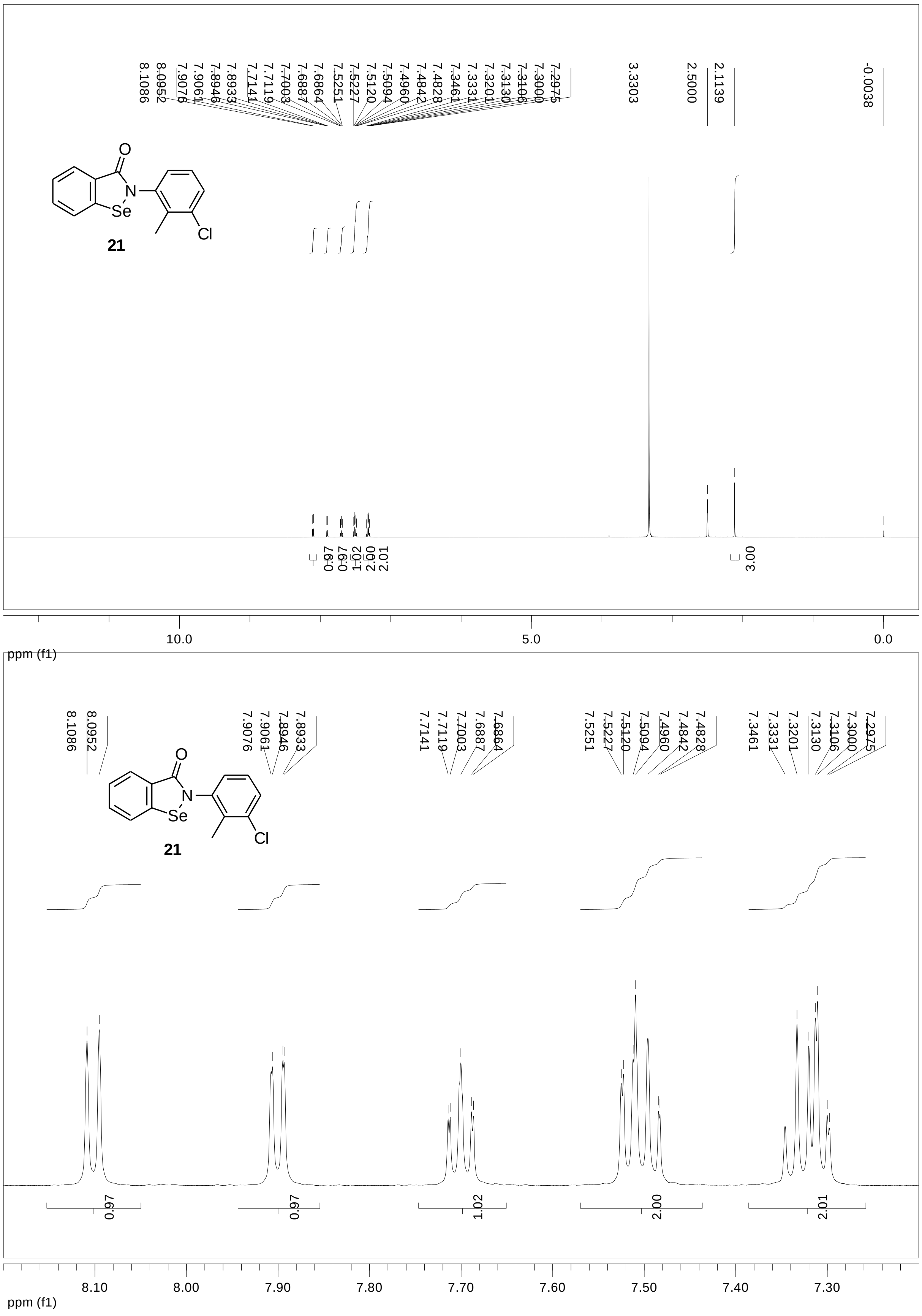

Fig. S101. ^{1}H-NMR (600.6 MHz, DMSO-d_6) spectrum of compound **21**

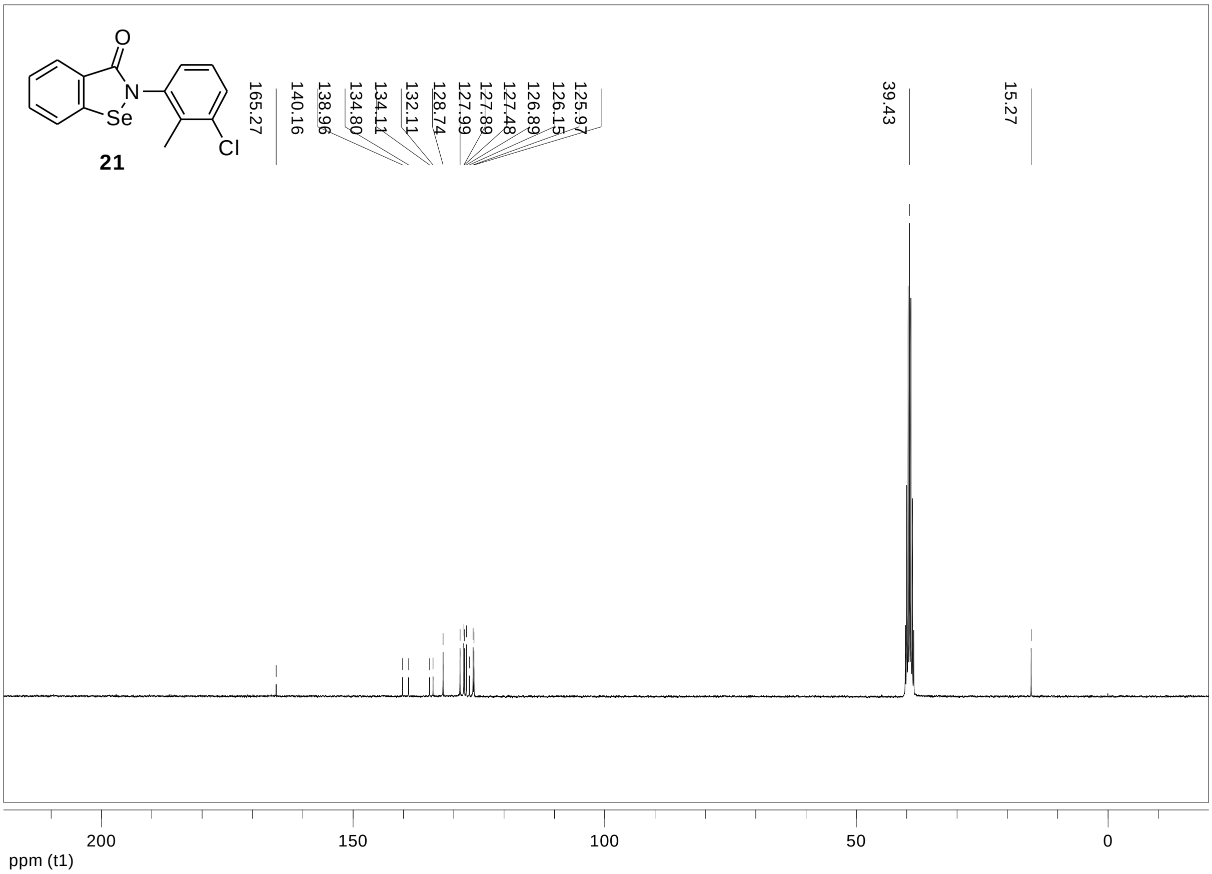

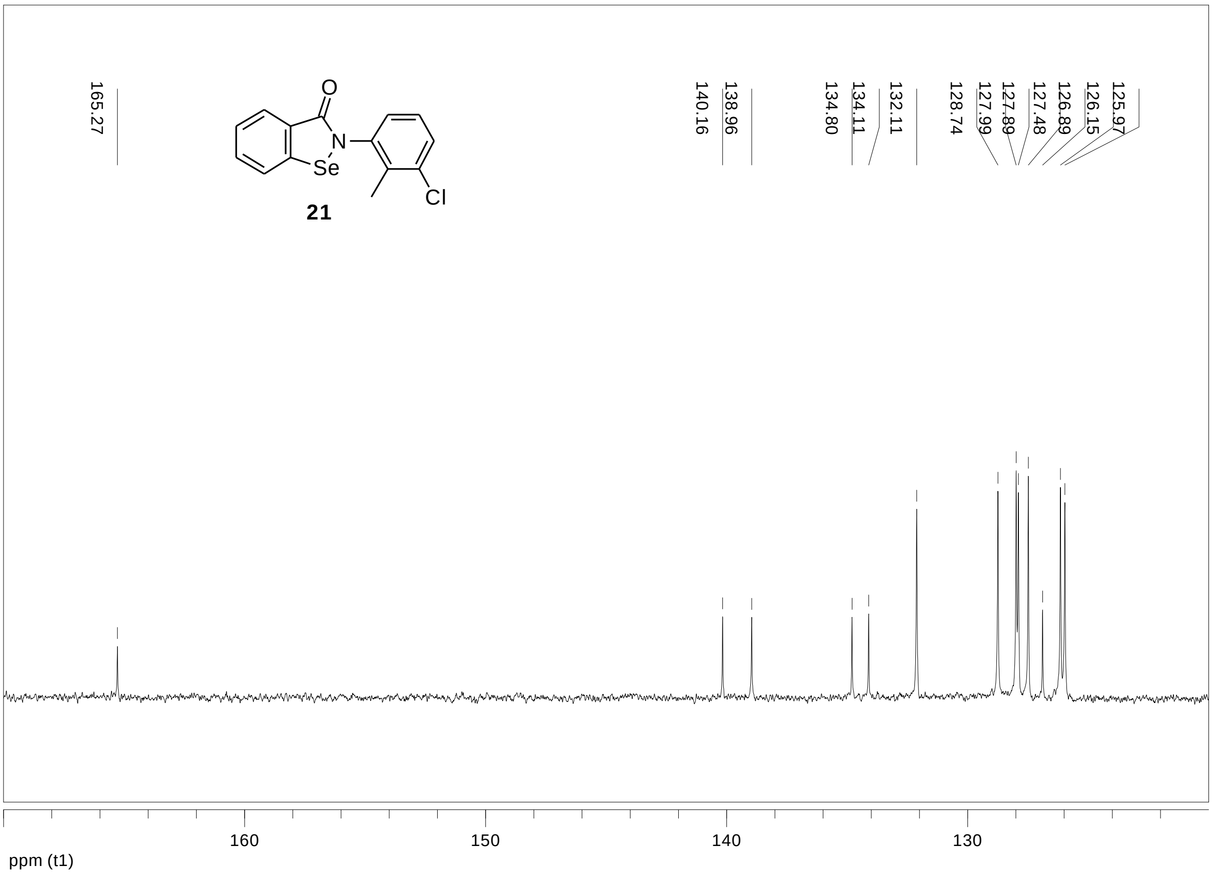

Fig. S102. ^{13}C-NMR (75.5 MHz, DMSO-d_6) spectrum of compound **21**

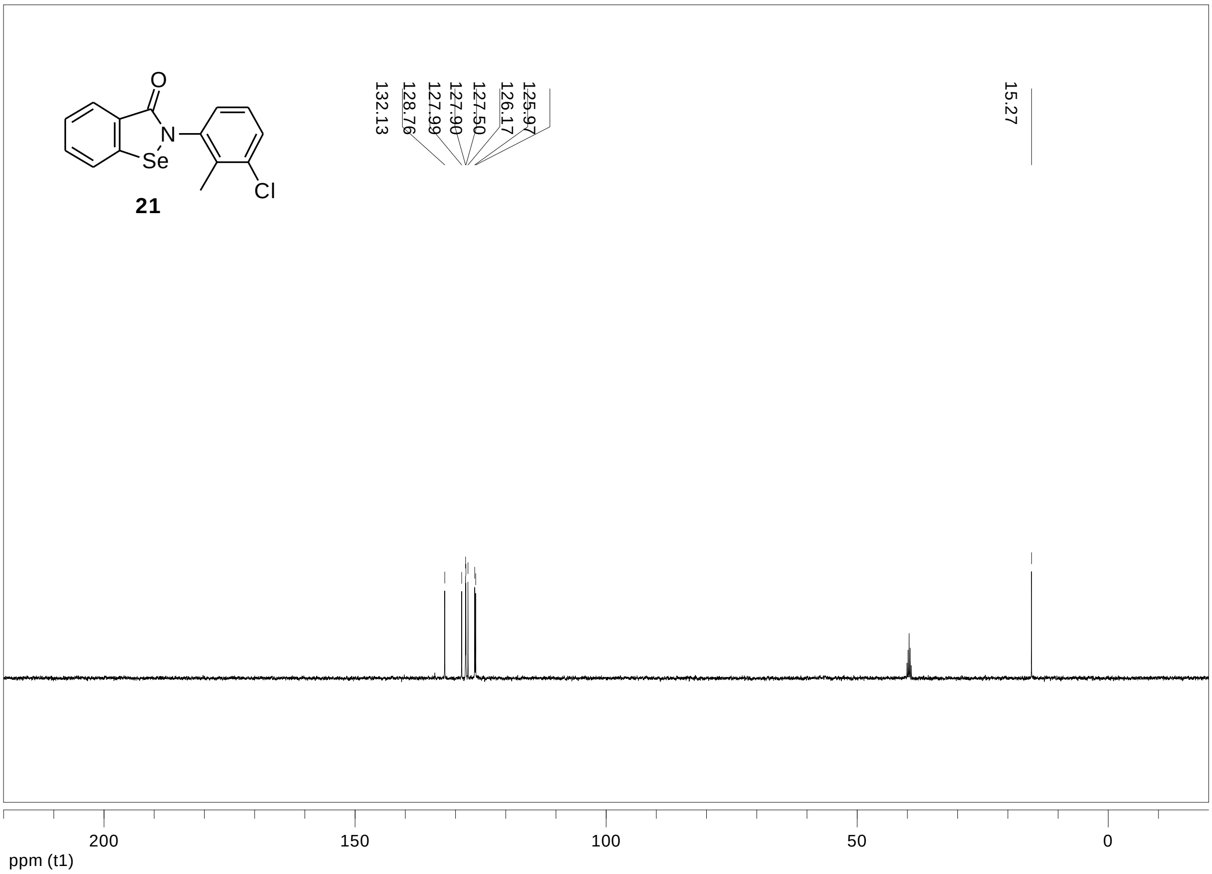

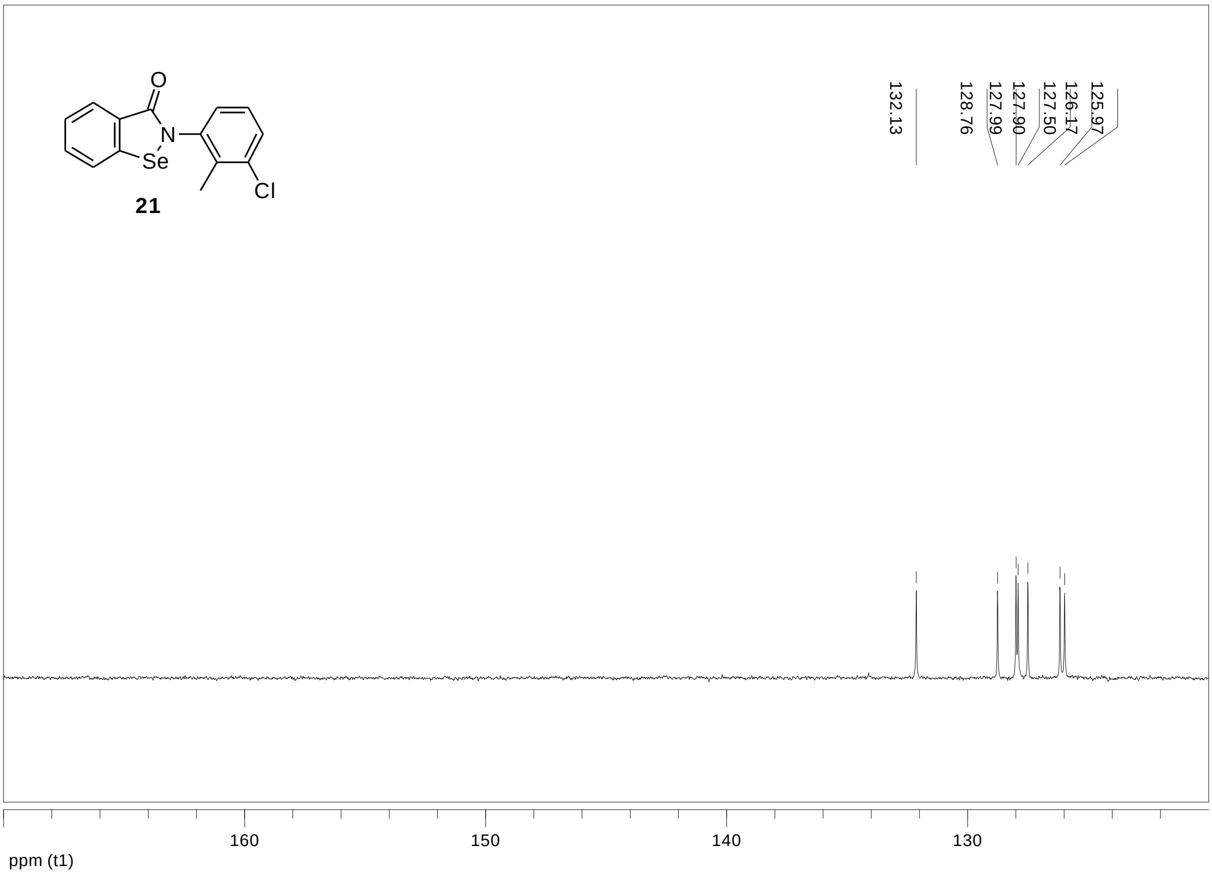

Fig. S103. ^{13}C-NMR (100.5 MHz, DMSO-d_6) dept-135 experiment of compound **21**

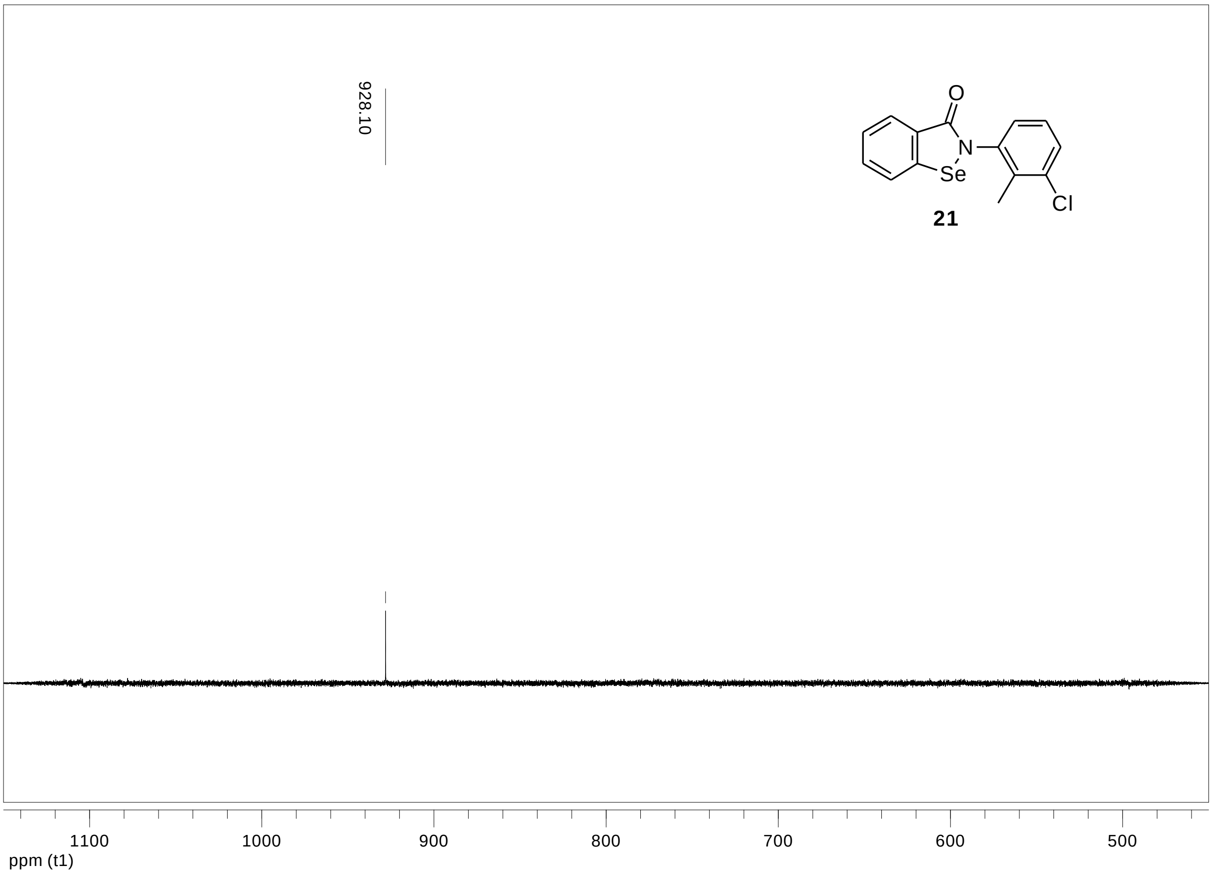

Fig. S104. ^{77}Se-NMR (76.24 MHz, DMSO-d_6)spectrum of compound **21**

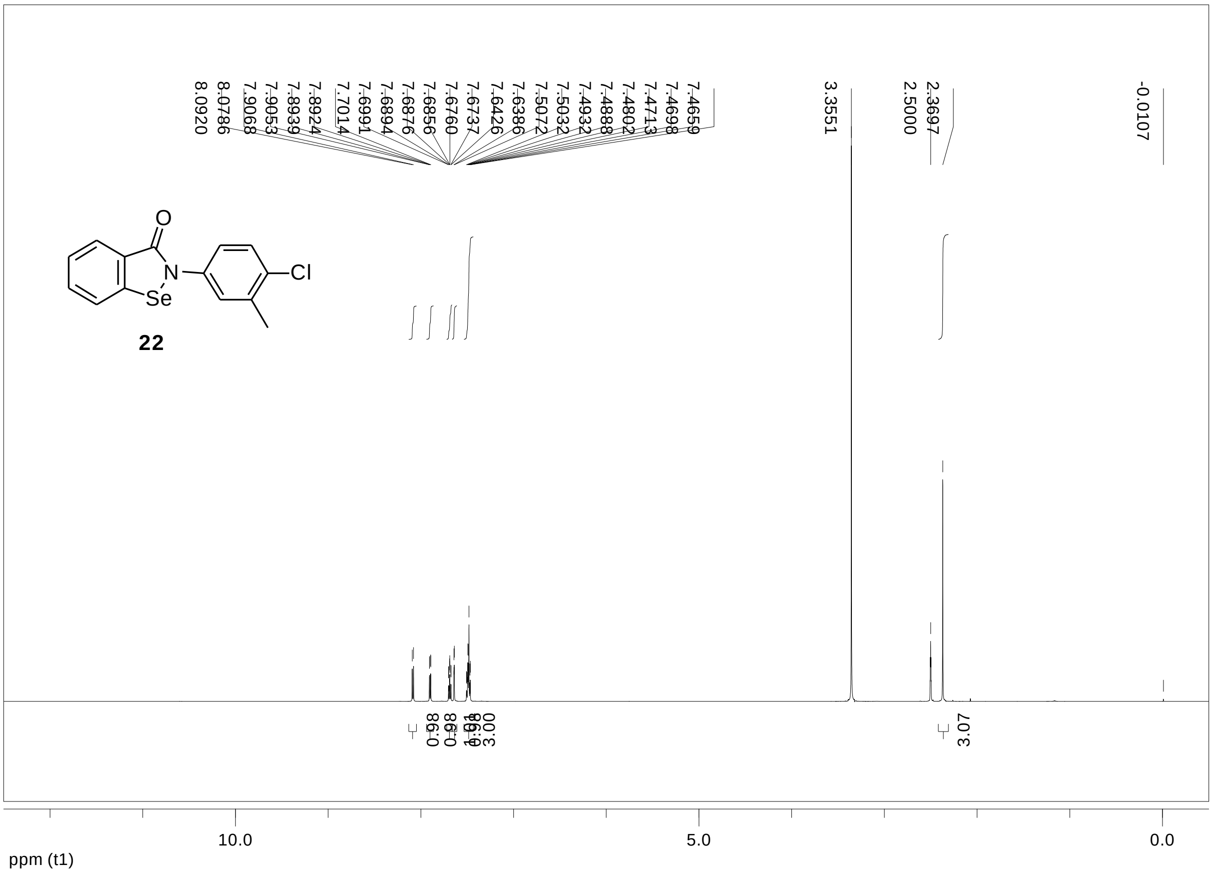

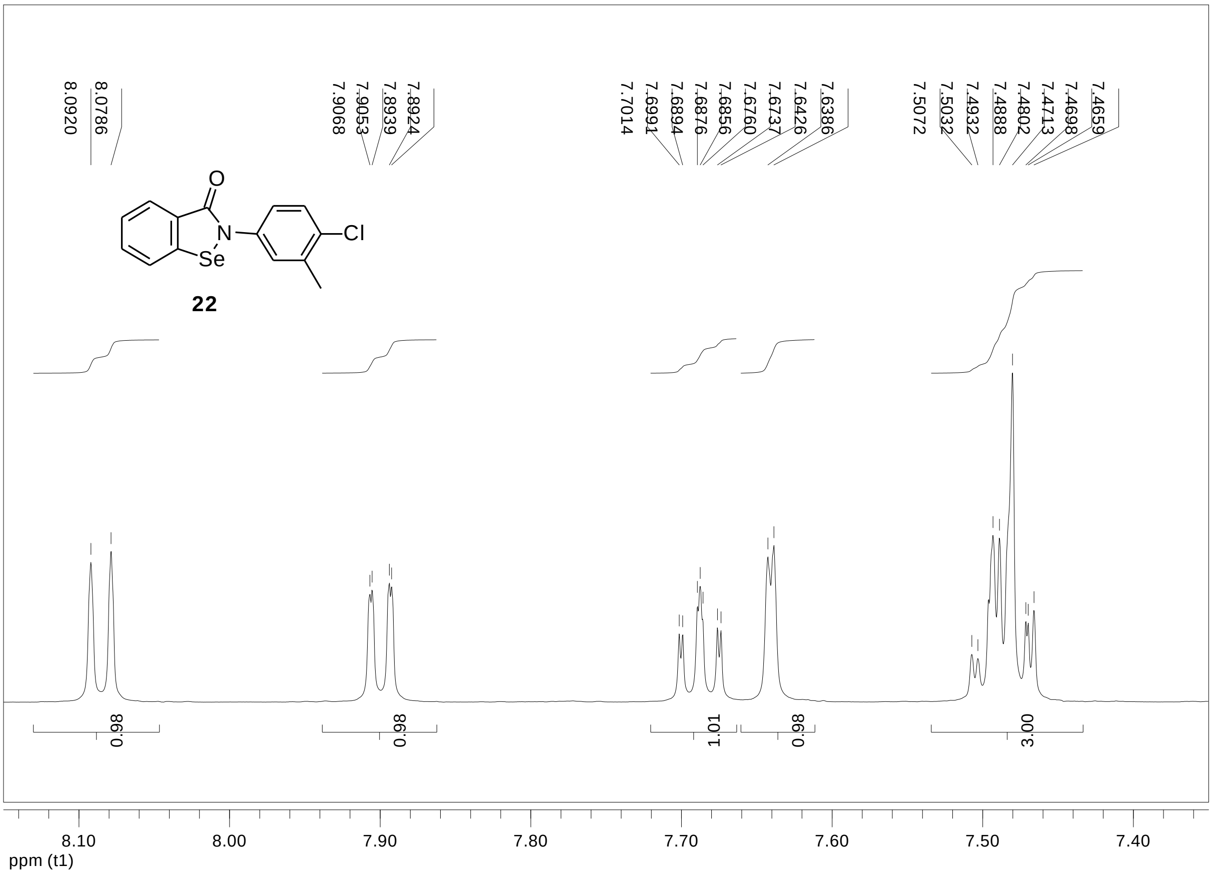

Fig. S105. ^{1}H-NMR (600.6 MHz, DMSO-d_6) spectrum of compound **22**

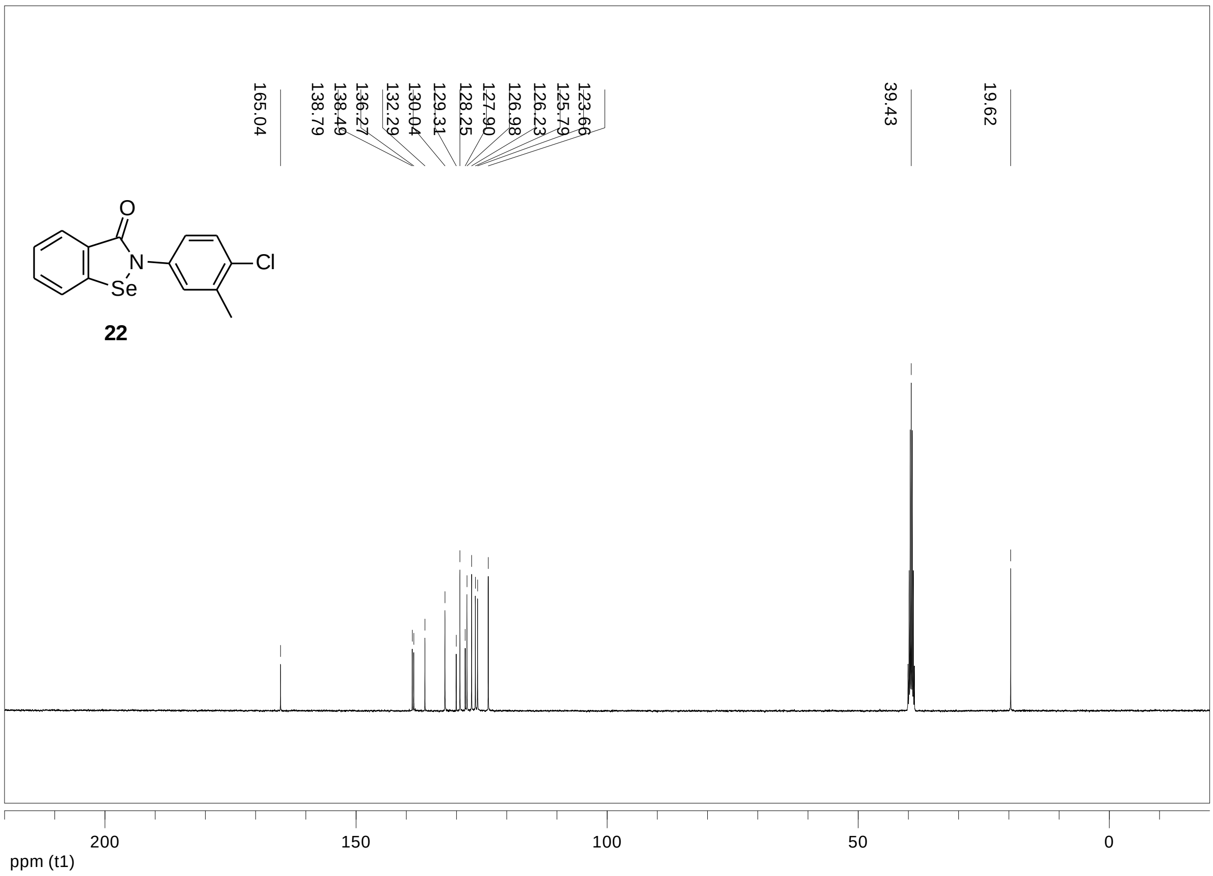

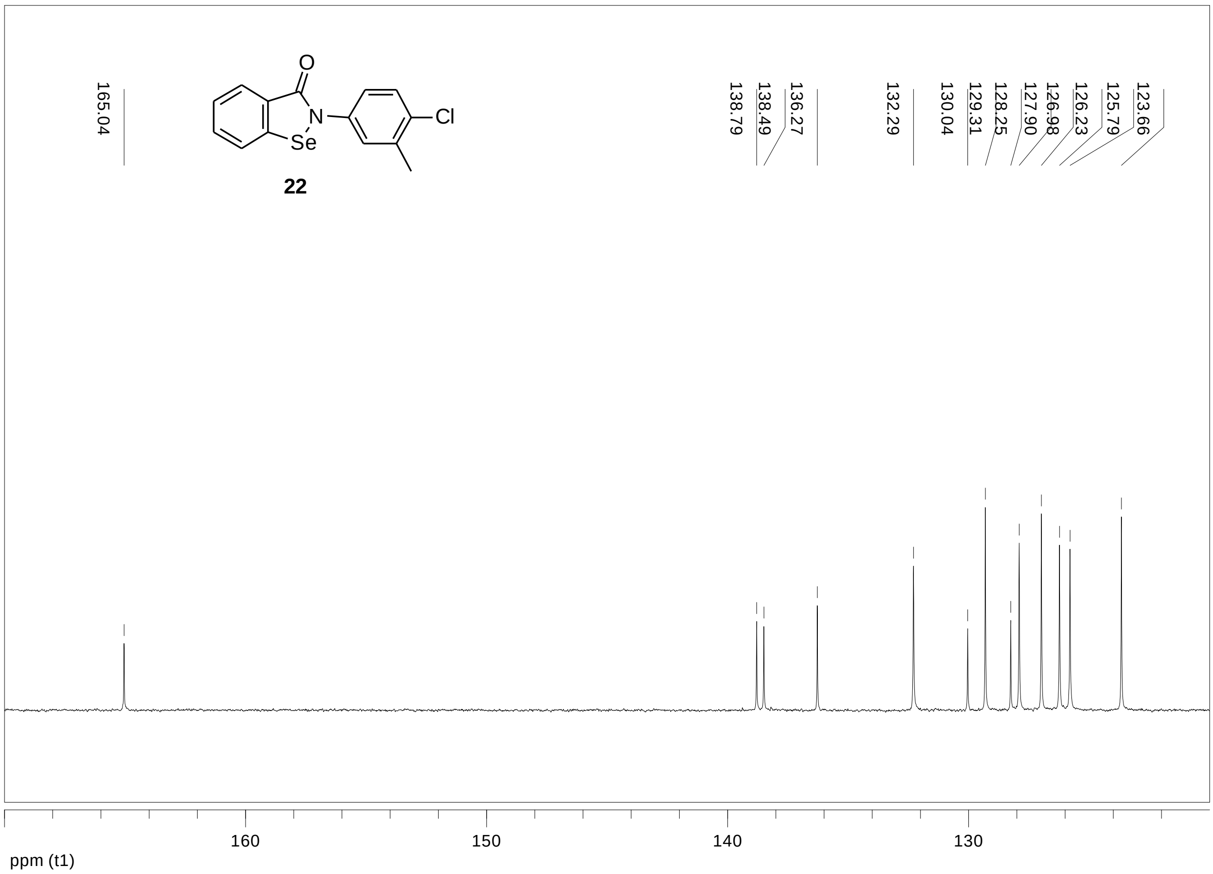

Fig. S106. ^{13}C-NMR (100.5 MHz, DMSO-d_6) spectrum of compound **22**

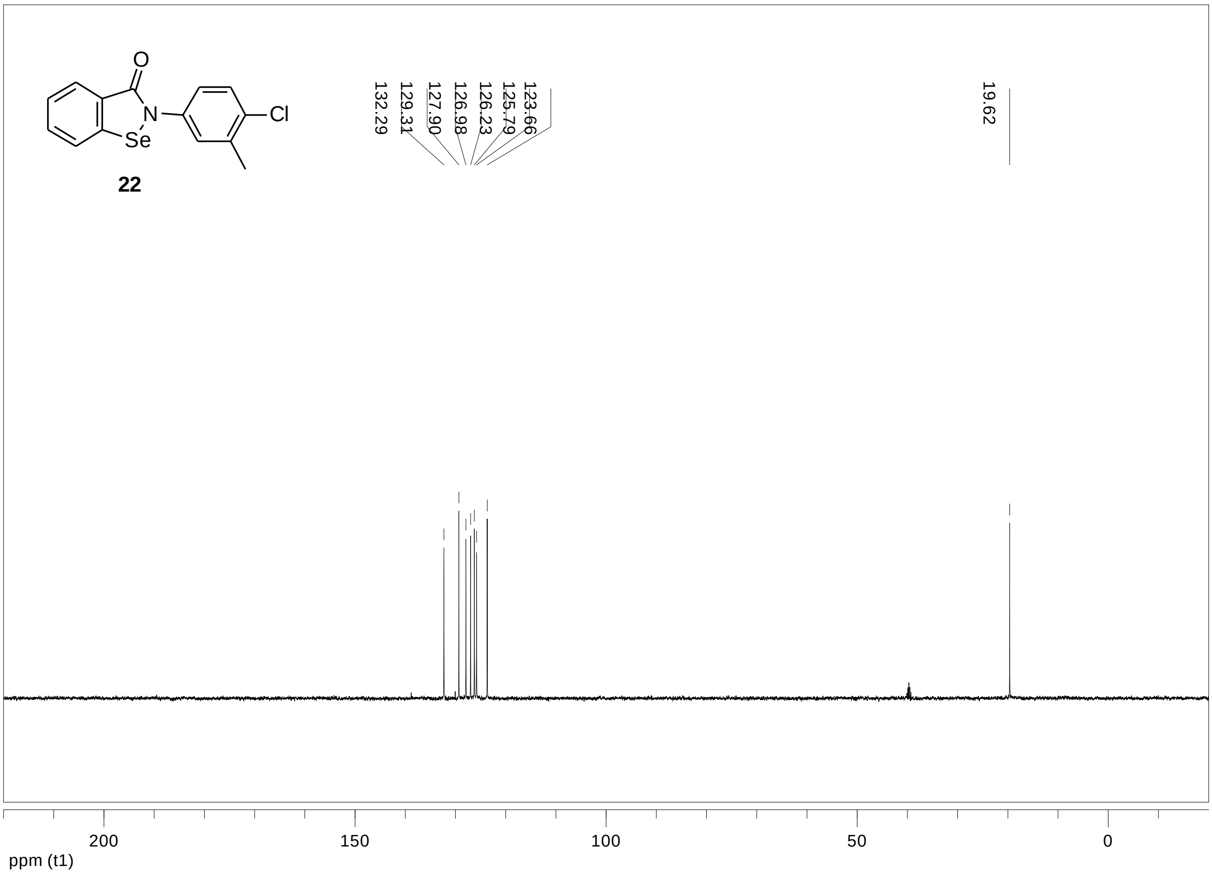

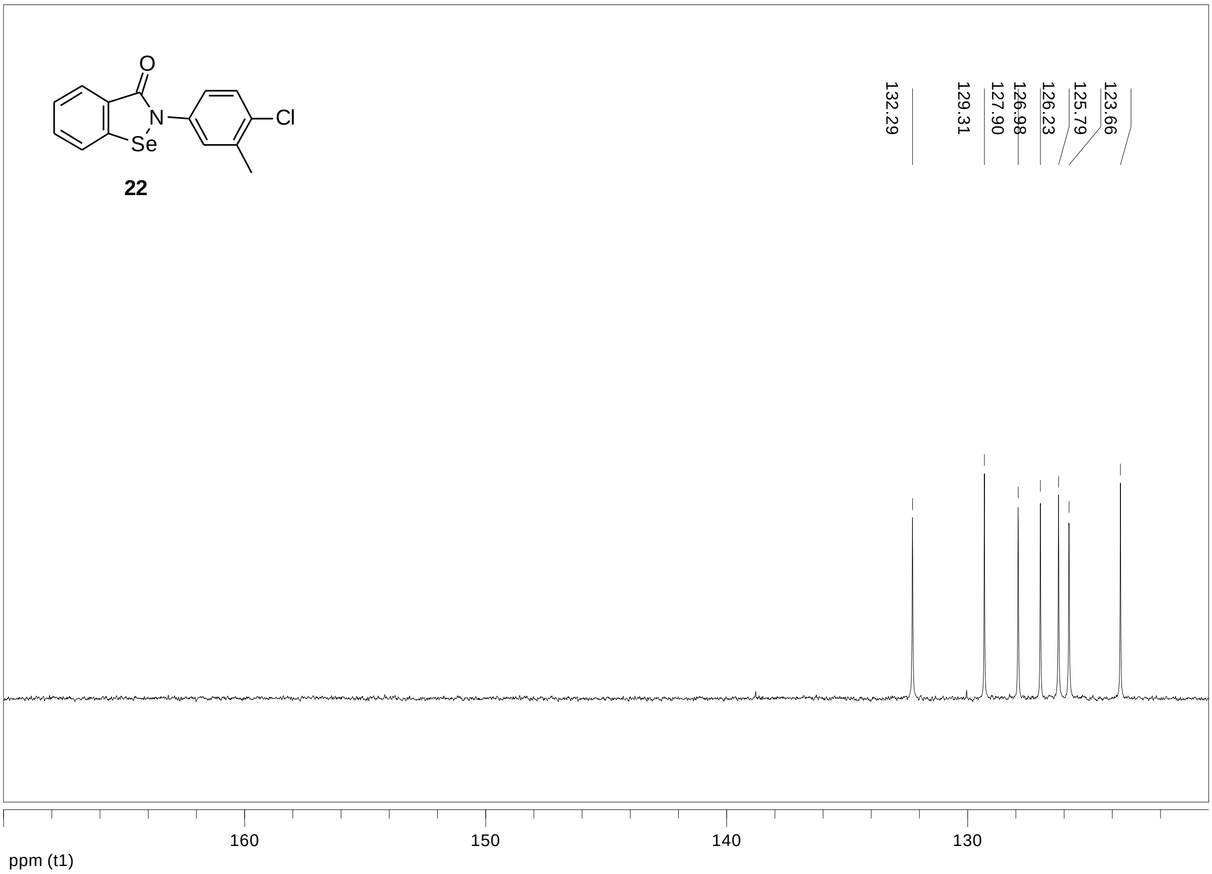

Fig. S107. ^{13}C-NMR (100.5 MHz, DMSO-d_6) dept-135 experiment of compound **22**

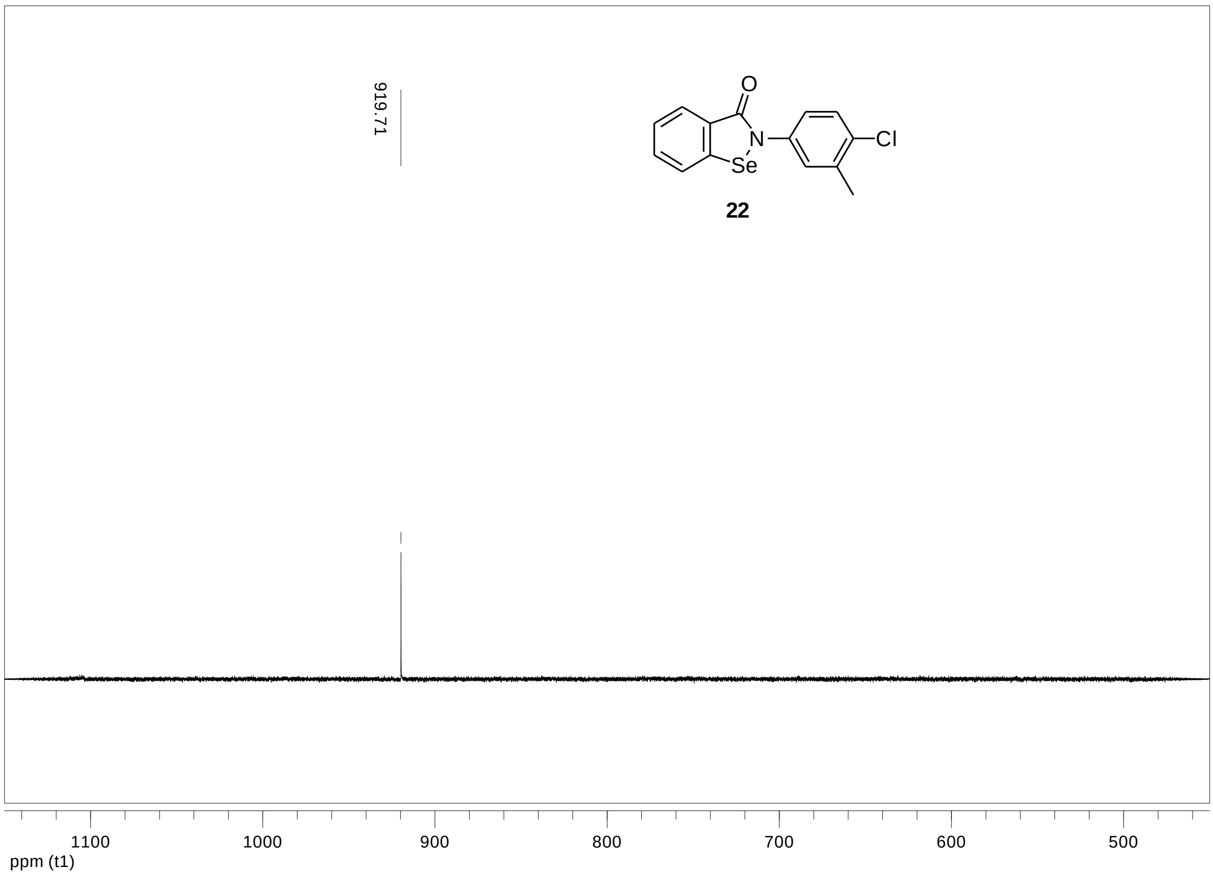

Fig. S108. ^{77}Se-NMR (76.24 MHz, DMSO-d_6) spectrum of compound **22**

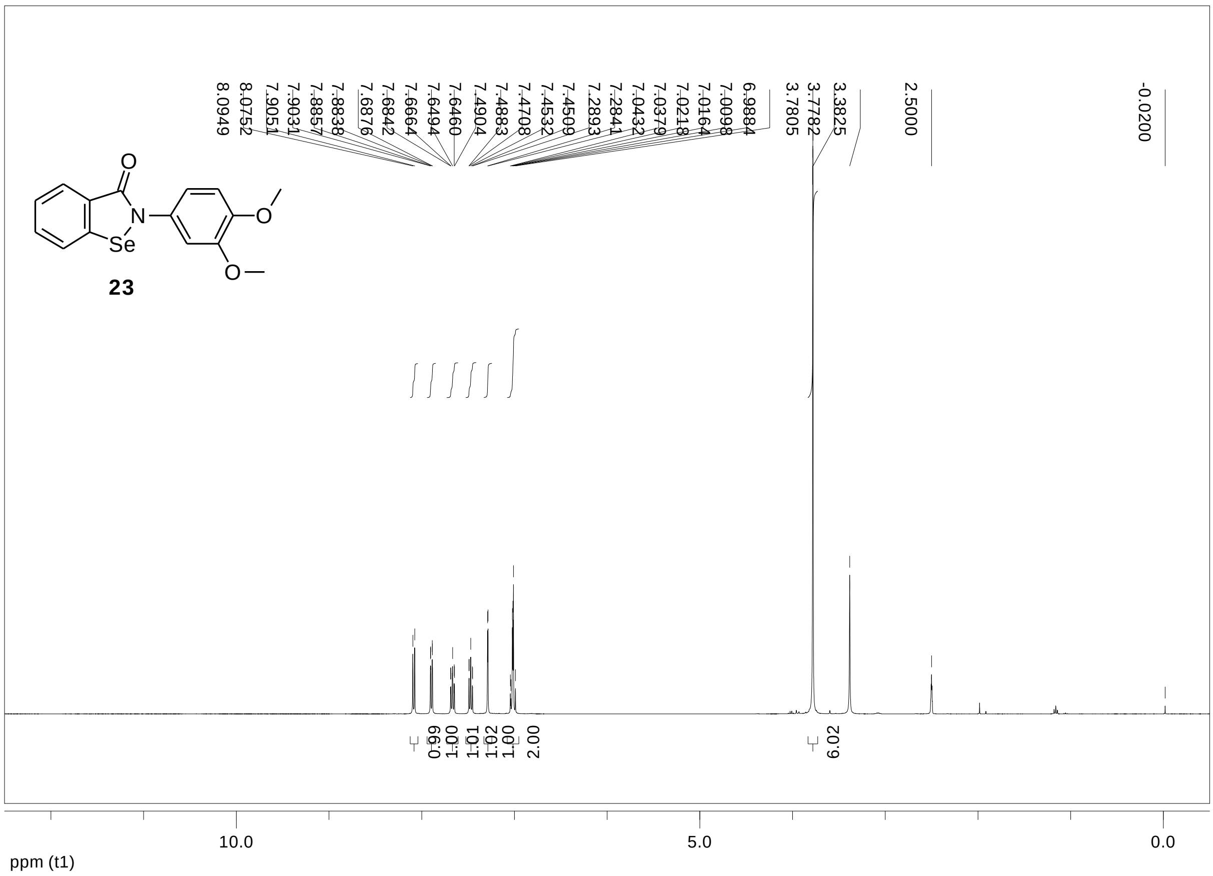

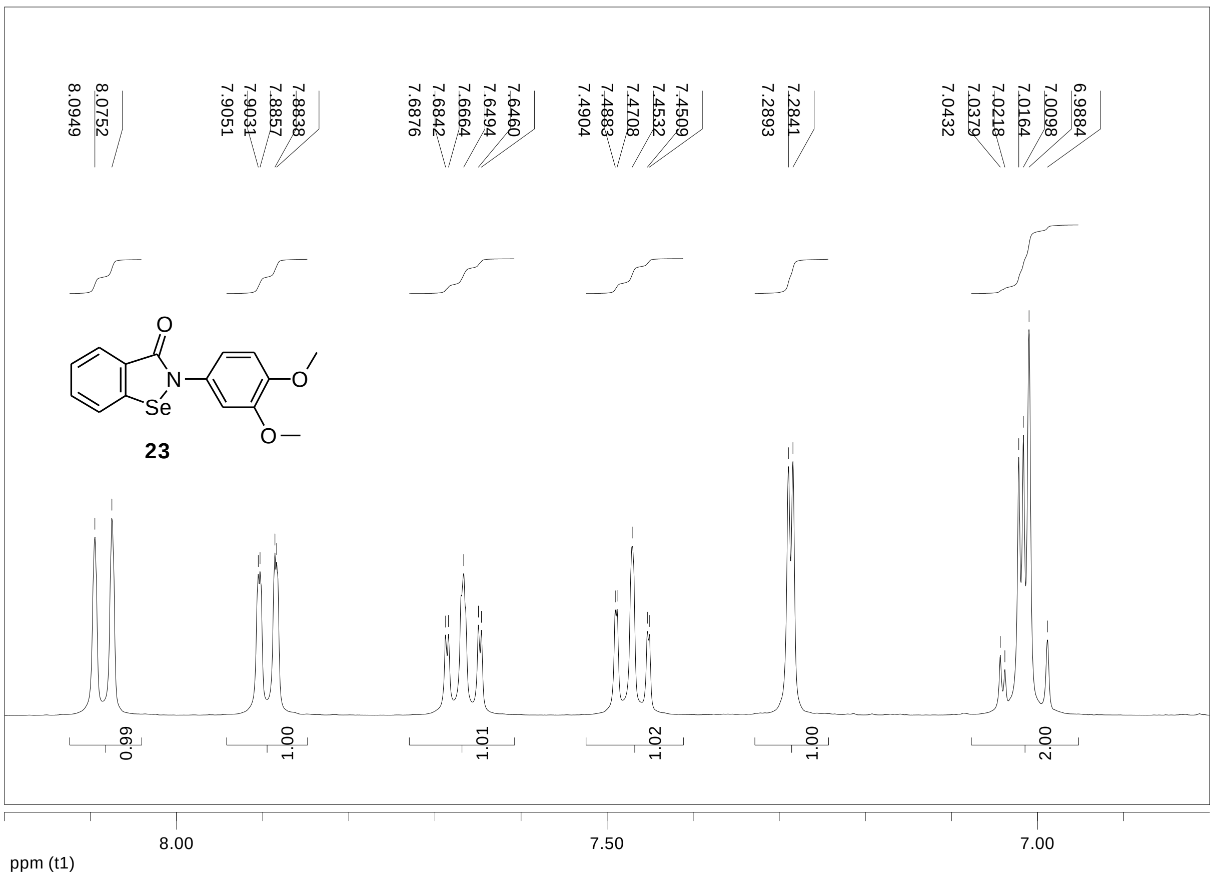

Fig. S109. ^{1}H-NMR (399.8 MHz, DMSO-d_6) spectrum of compound **23**

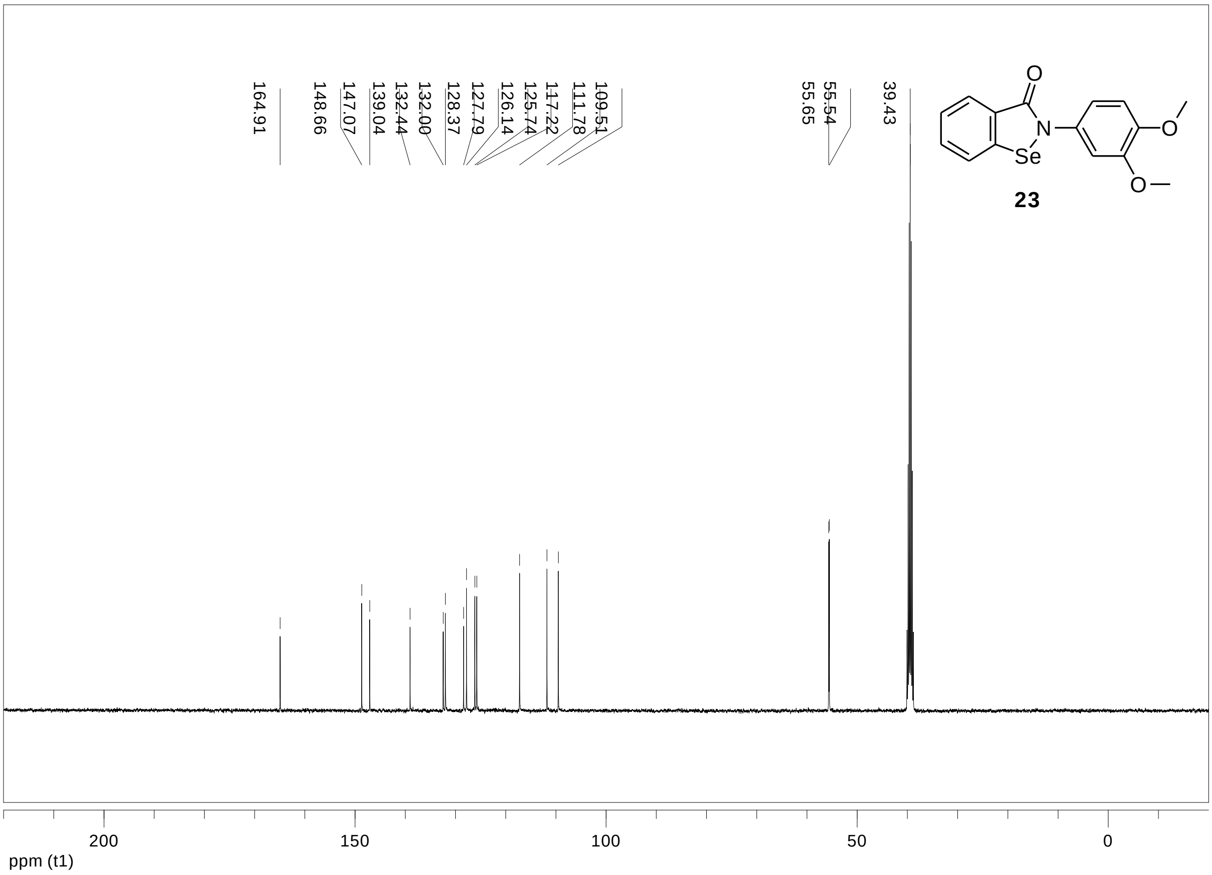

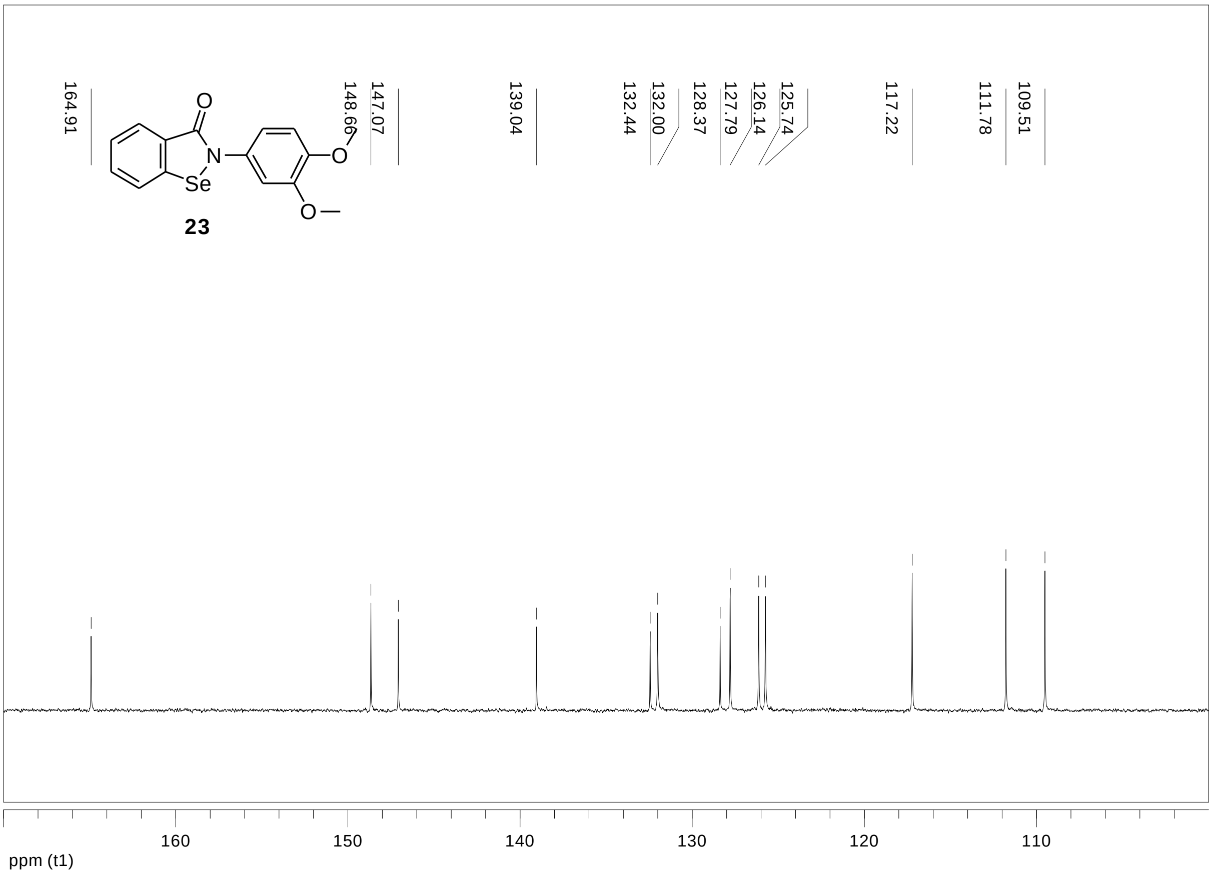

Fig. S110. ^{13}C-NMR (100.5 MHz, DMSO-d_6) spectrum of compound **23**

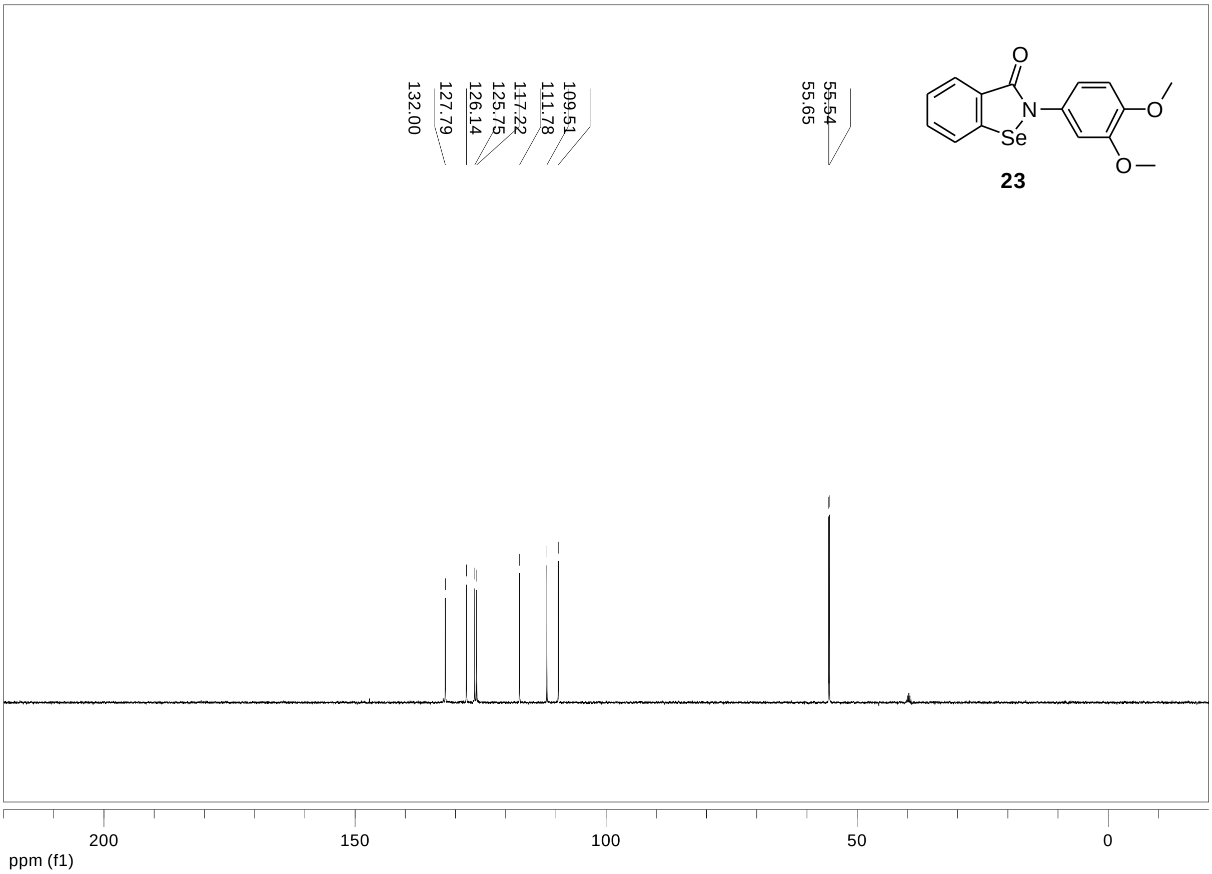

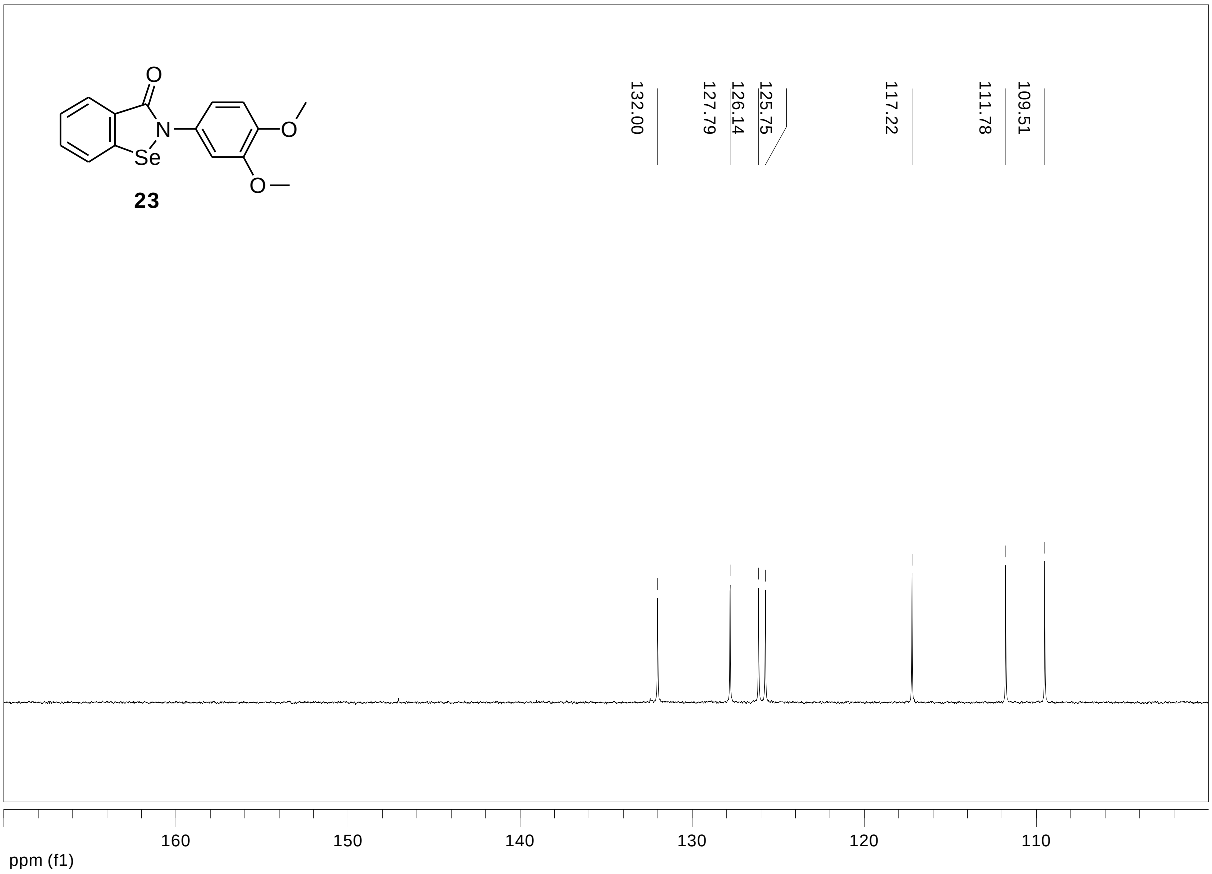

Fig. S111. ^{13}C-NMR (100.5 MHz, DMSO-d_6) dept-135 experiment of compound **23**

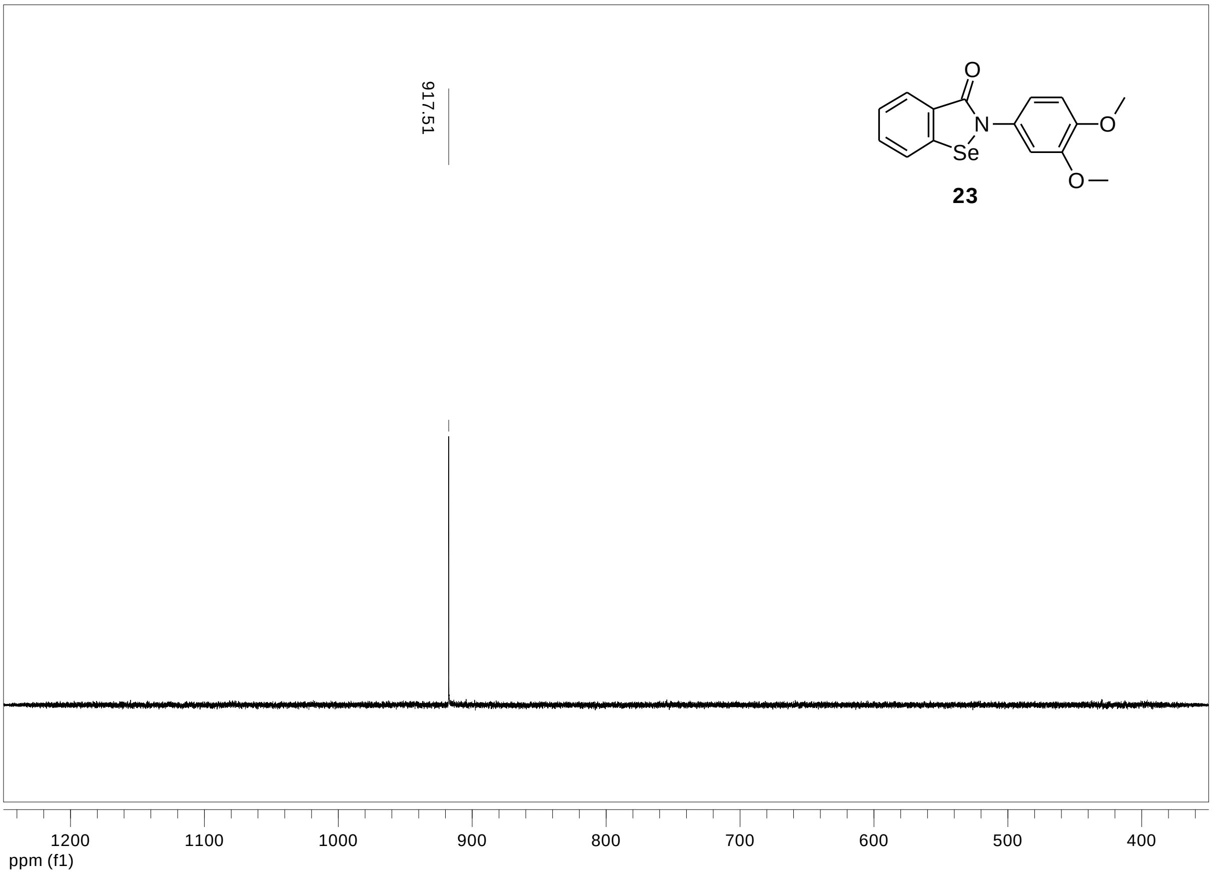

Fig. S112. ^{77}Se-NMR (76.24 MHz, DMSO-d_6) spectrum of compound **23**

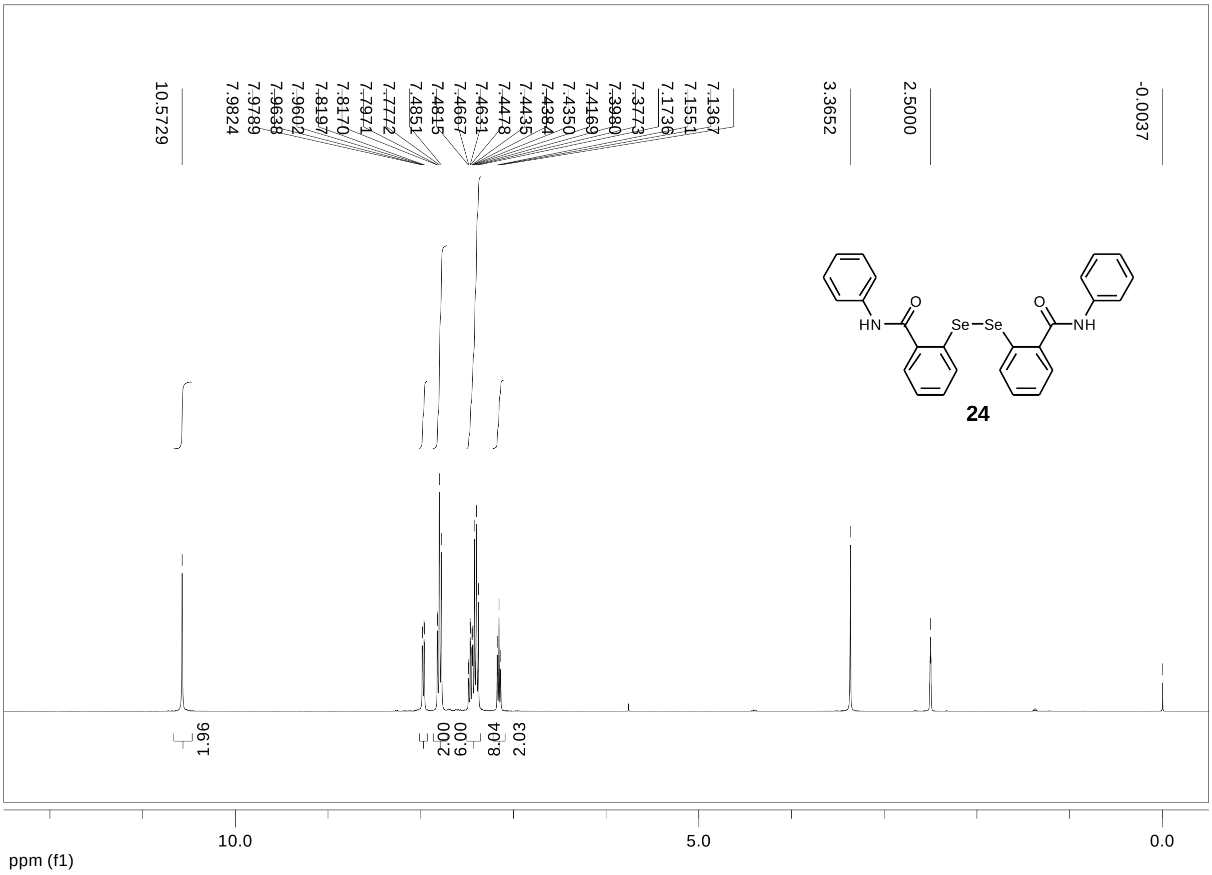

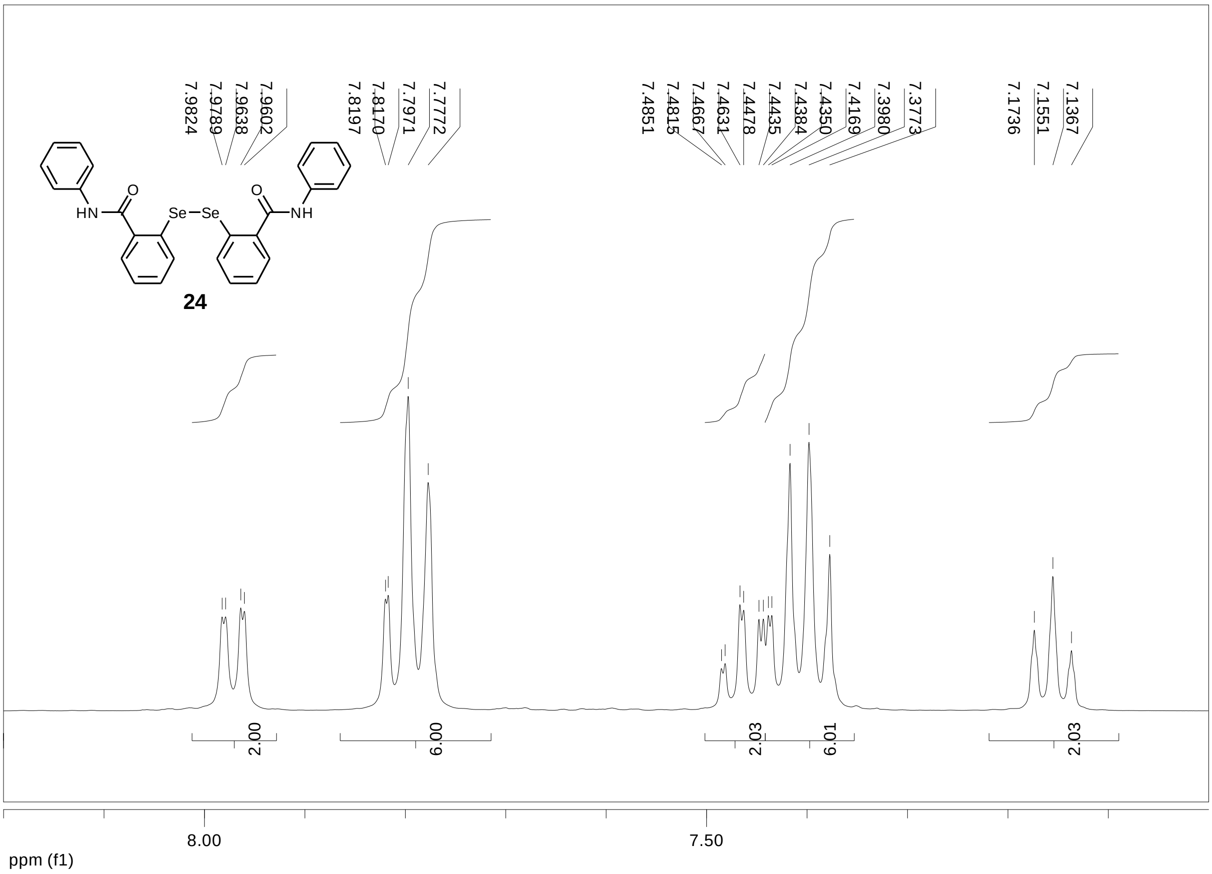

Fig. S113. ^{1}H-NMR (399.8 MHz, DMSO-d_6) spectrum of compound **24**

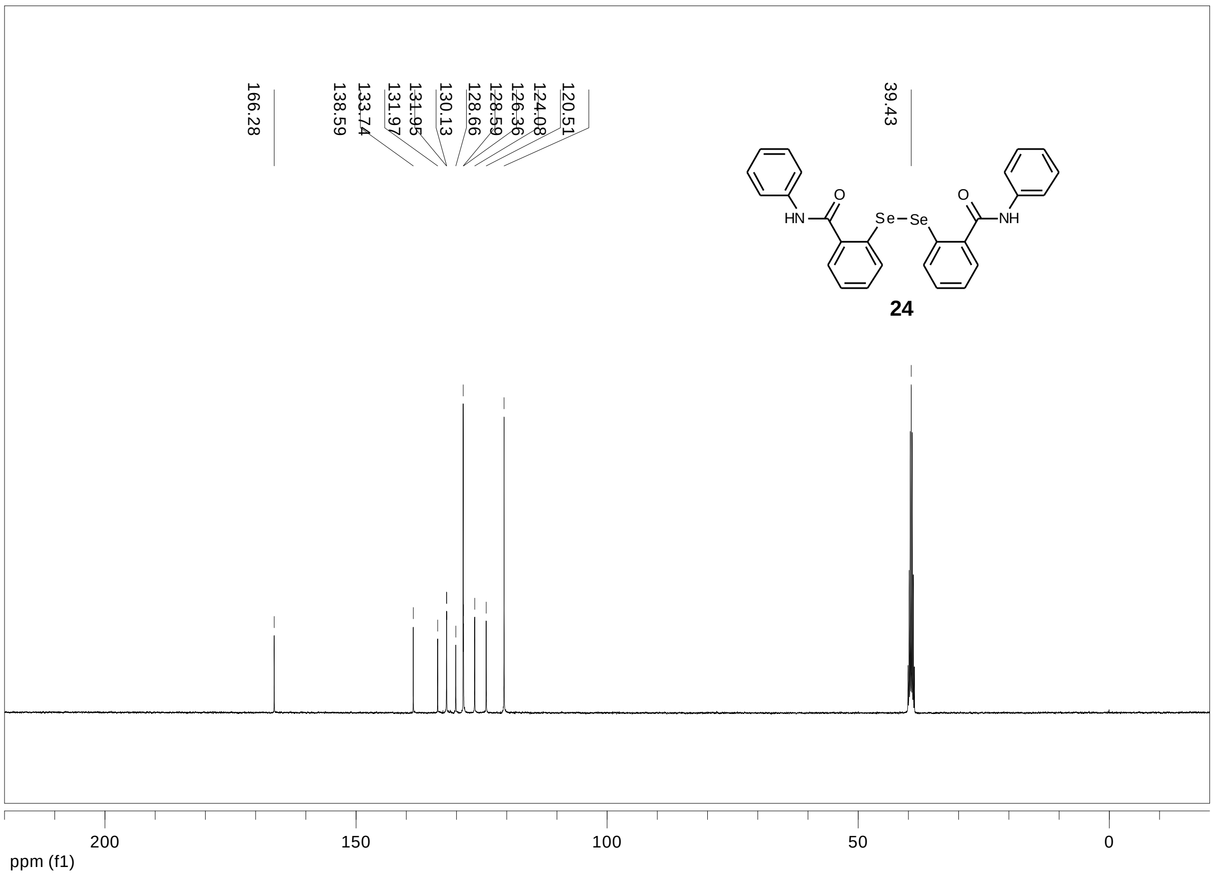

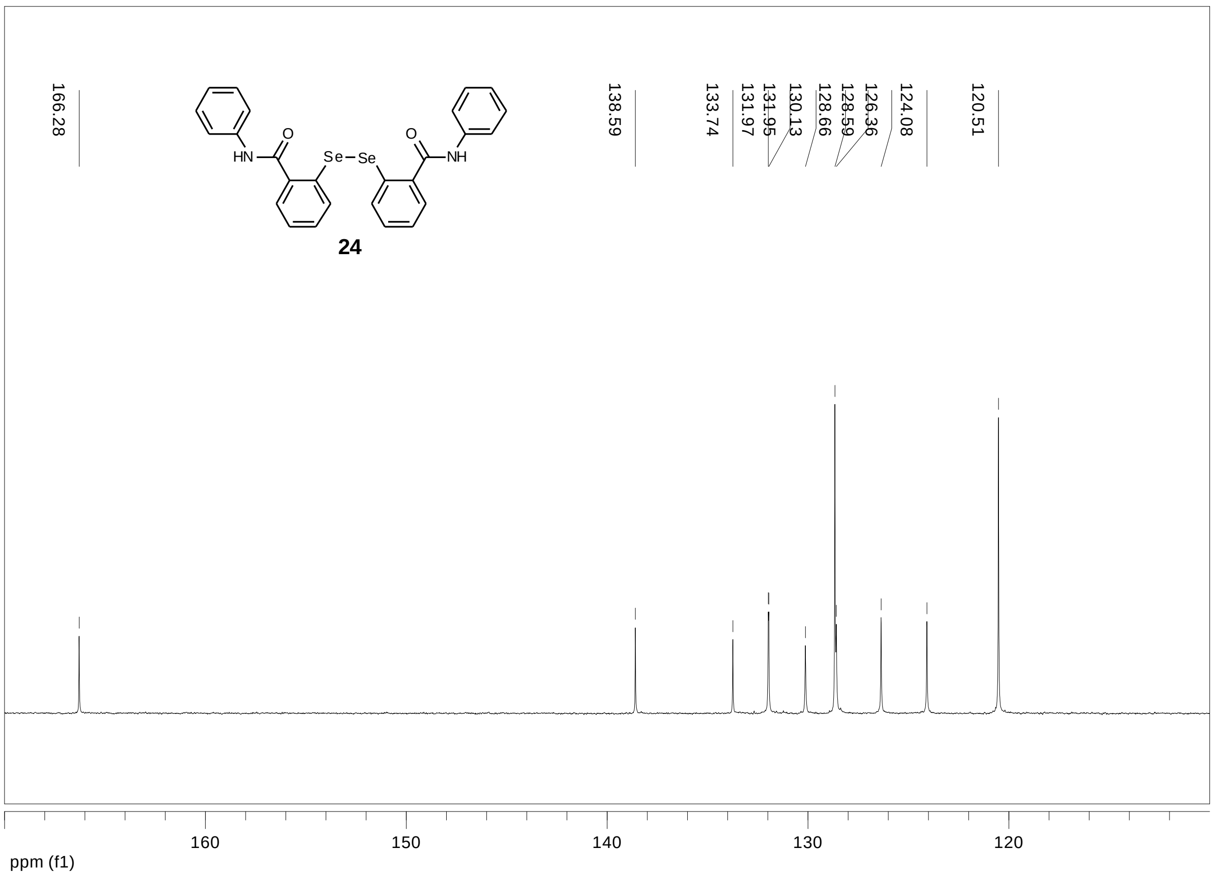

Fig. S114. ^{13}C-NMR (100.5 MHz, DMSO-d_6) spectrum of compound **24**

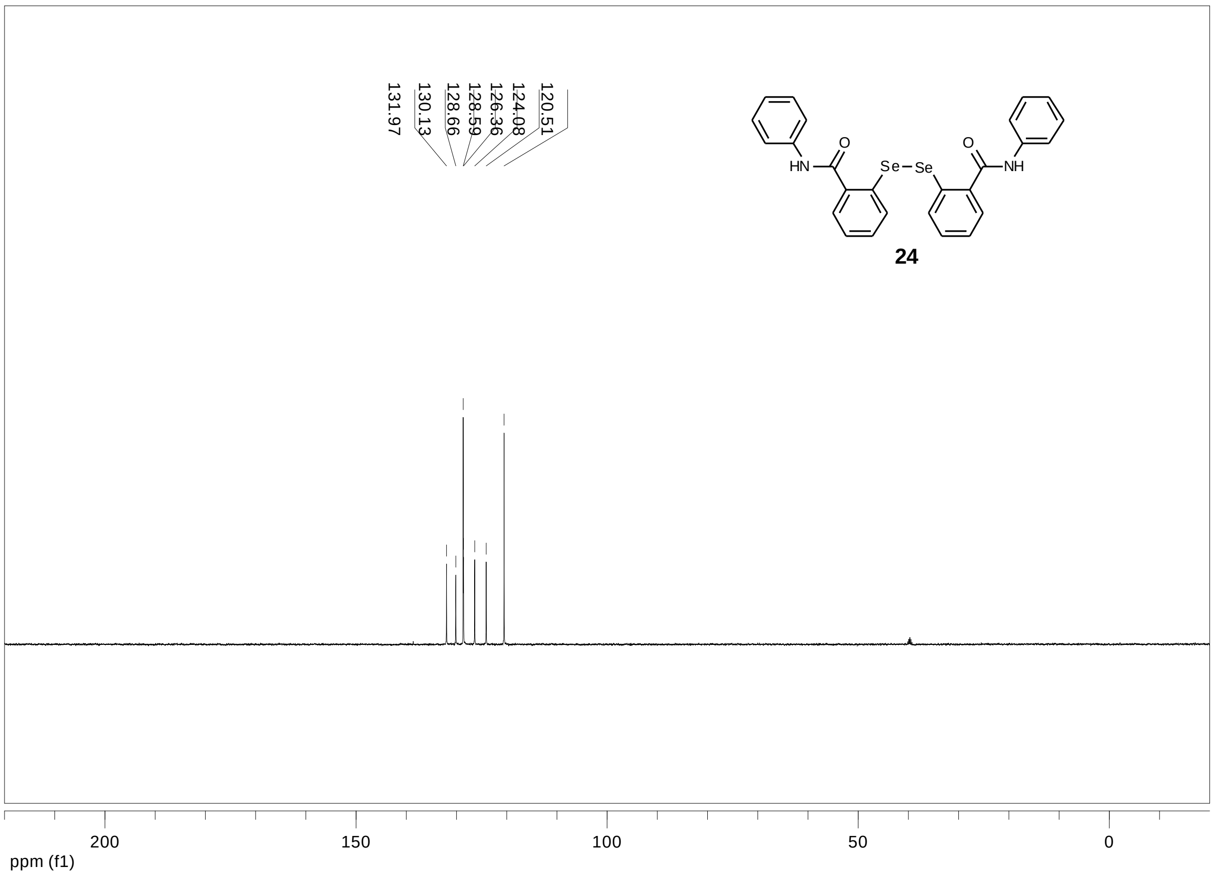

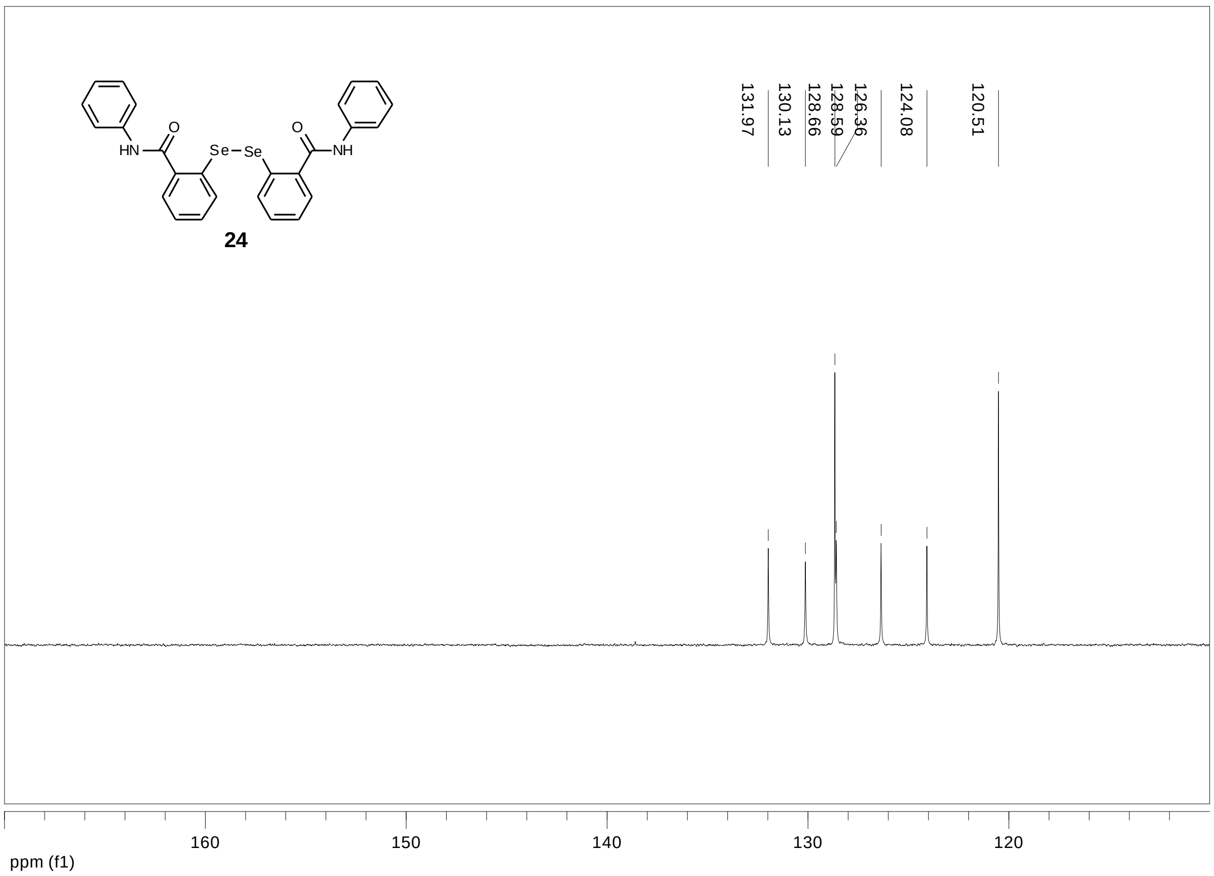

Fig. S115. ^{13}C-NMR (100.5 MHz, DMSO-d_6) dept-135 experiment of compound **24**

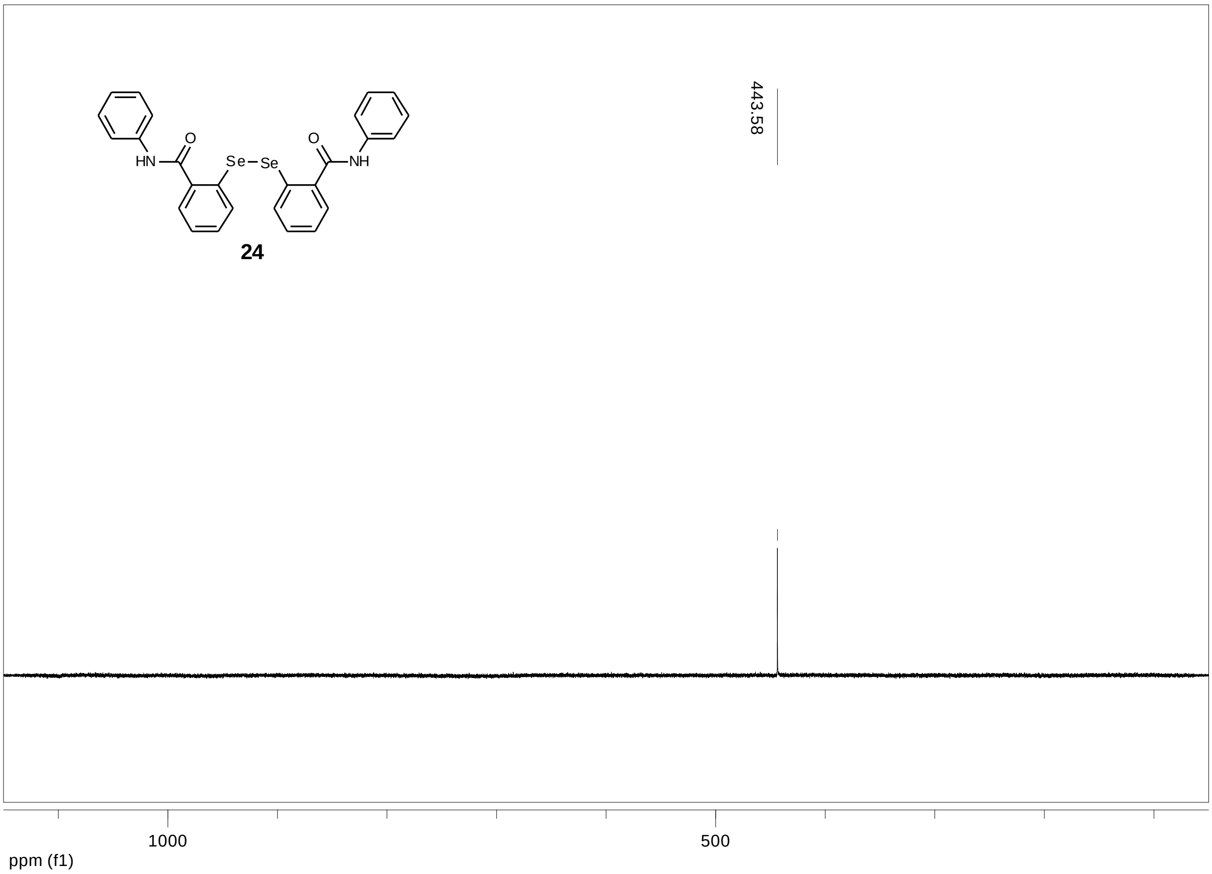

Fig. S116. ^{77}Se-NMR (76.24 MHz, DMSO-d_6) spectrum of compound **24**

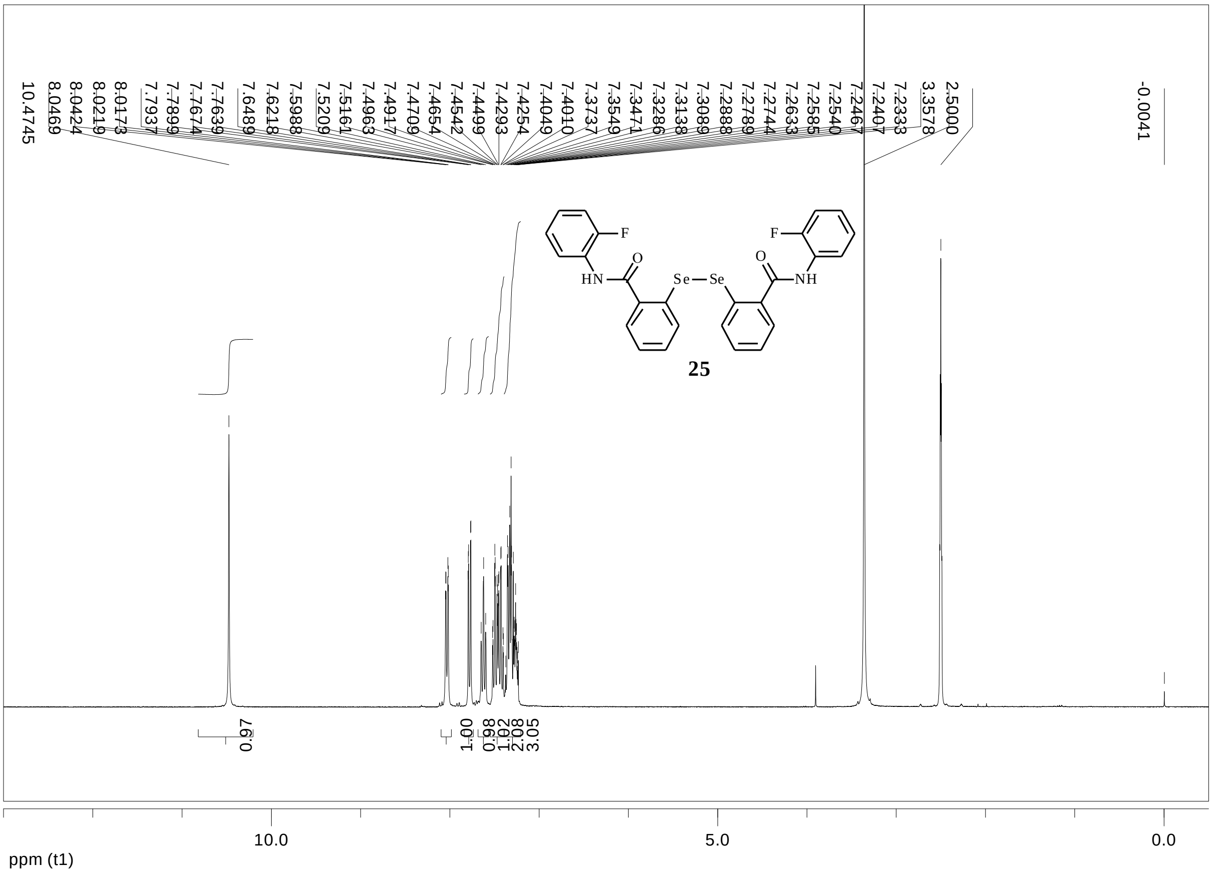

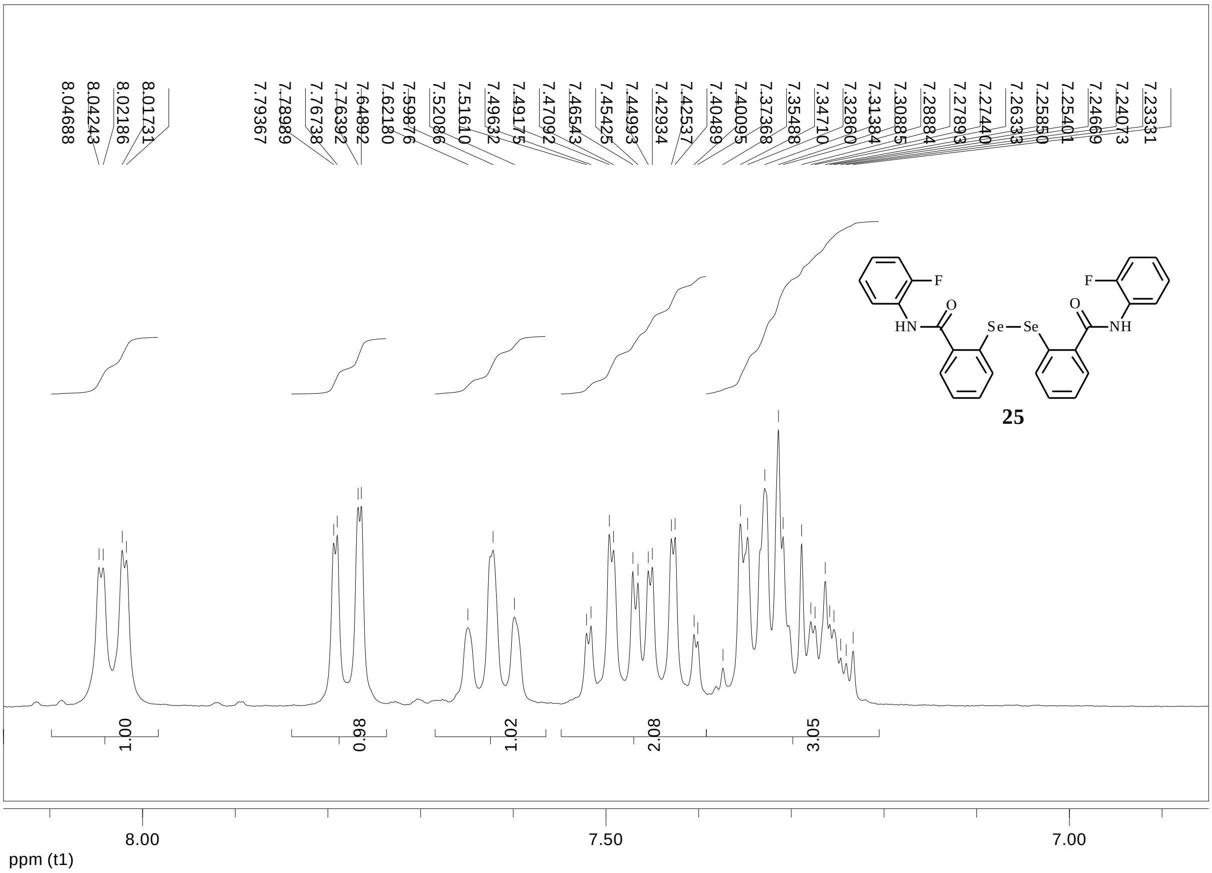

Fig. S117. ^{1}H-NMR (300.1 MHz, DMSO-d_6) spectrum of compound **25**

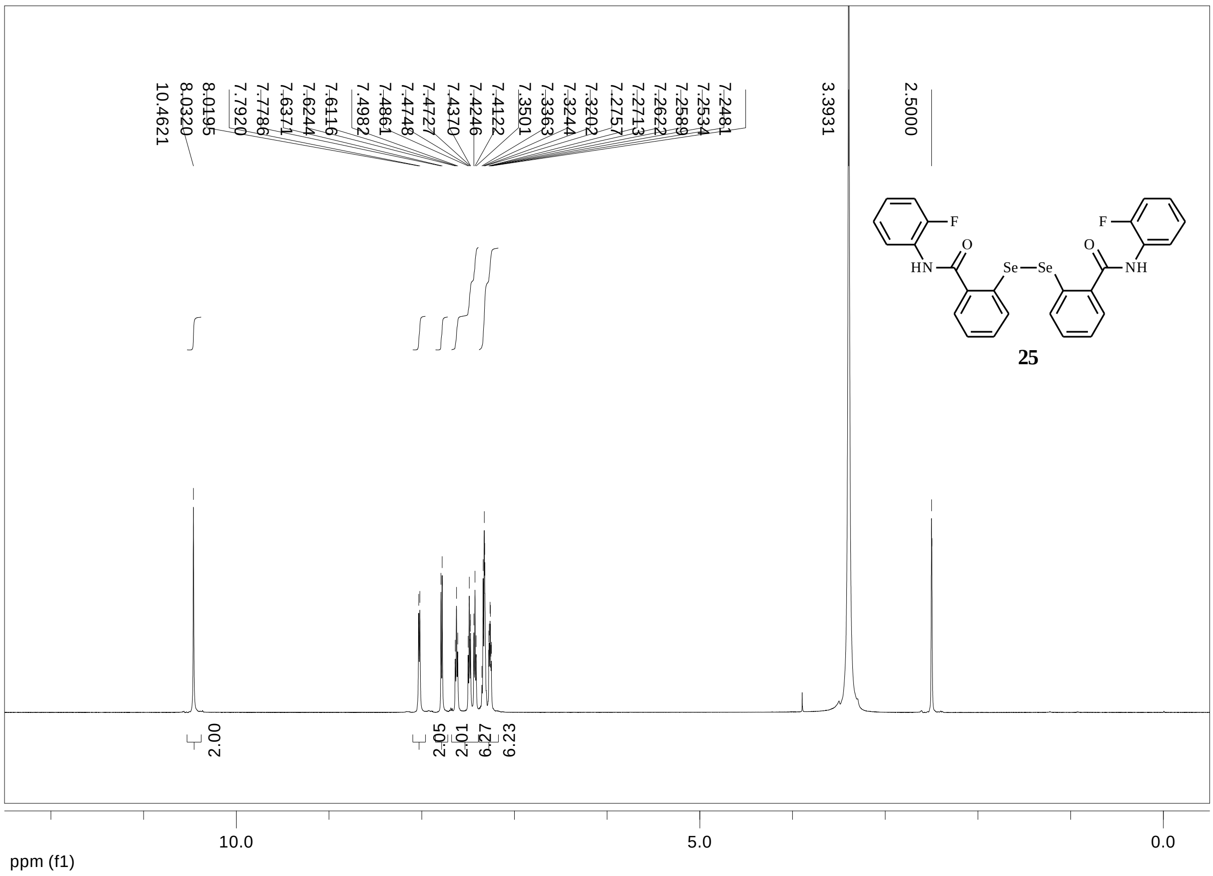

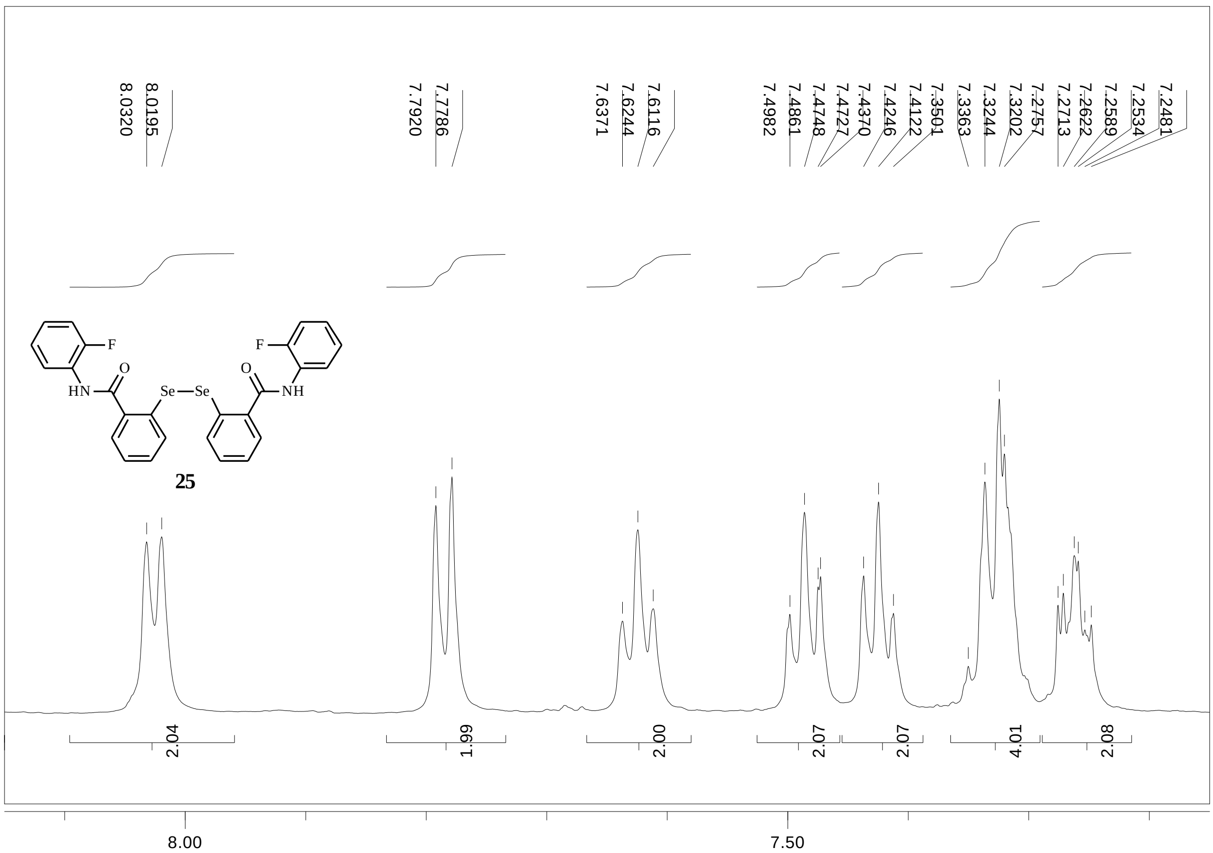

Fig. S118.^{1}H-NMR (600.6 MHz, DMSO-d_6) spectrum of compound **25**

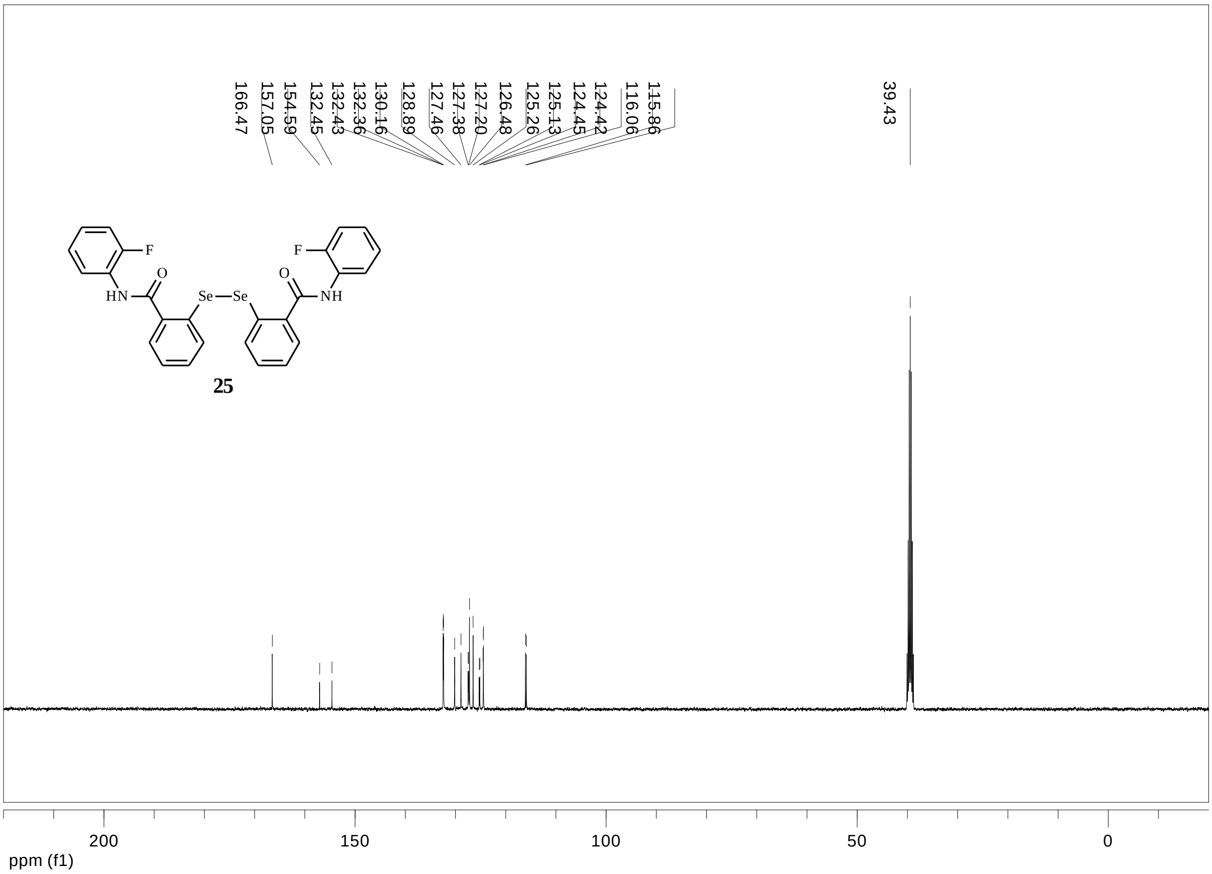

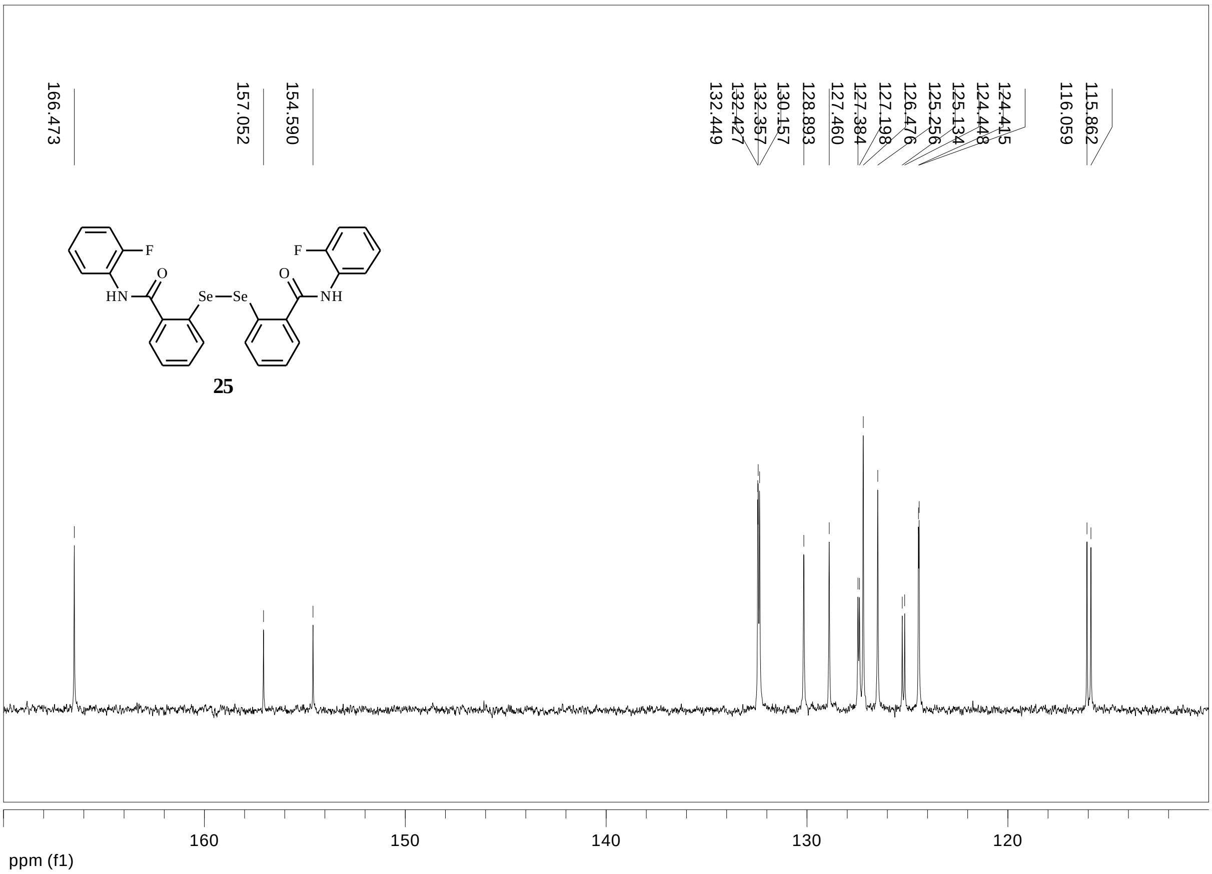

Fig. S119. ^{13}C-NMR (100.5 MHz, DMSO-d_6) spectrum of compound **25**

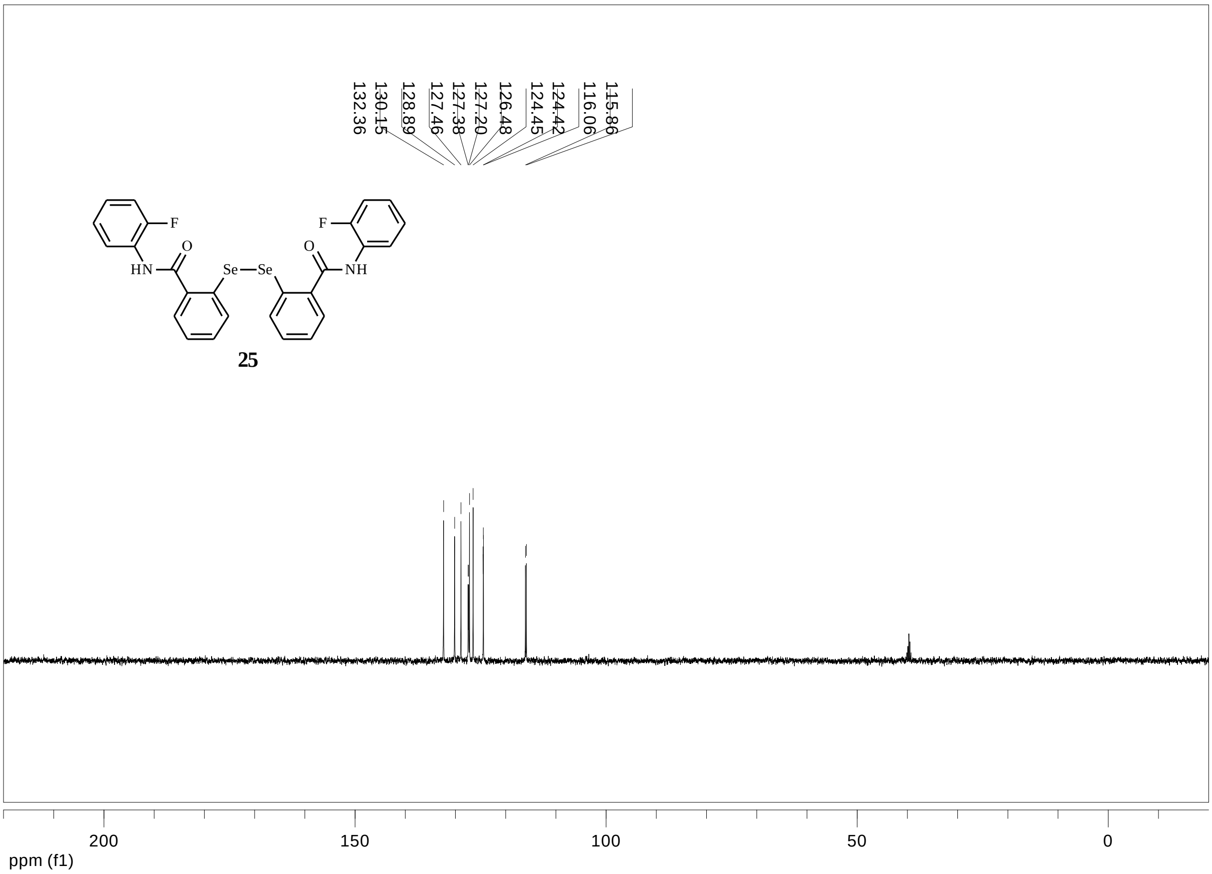

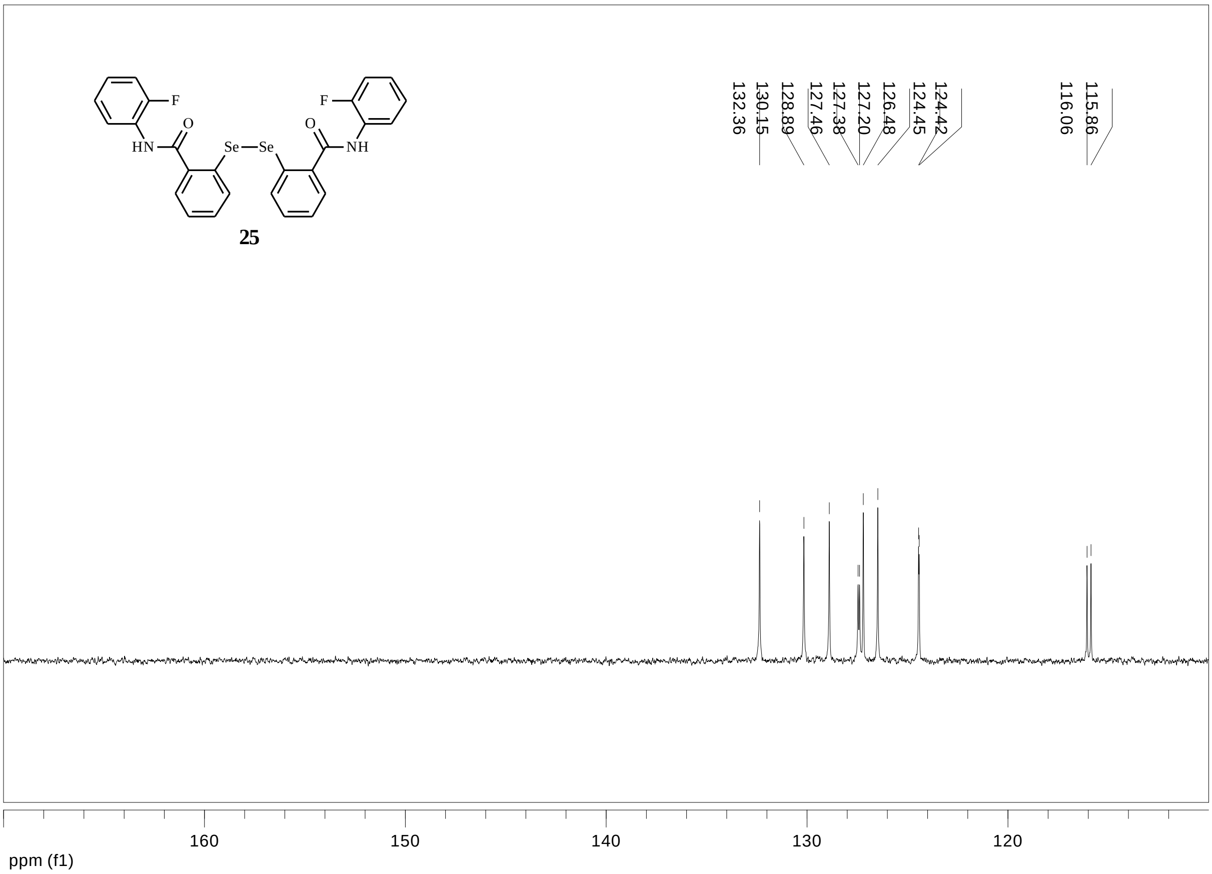

Fig. S120. ^{13}C-NMR (100.5 MHz, DMSO-d_6) dept-135 experiment of compound **25**

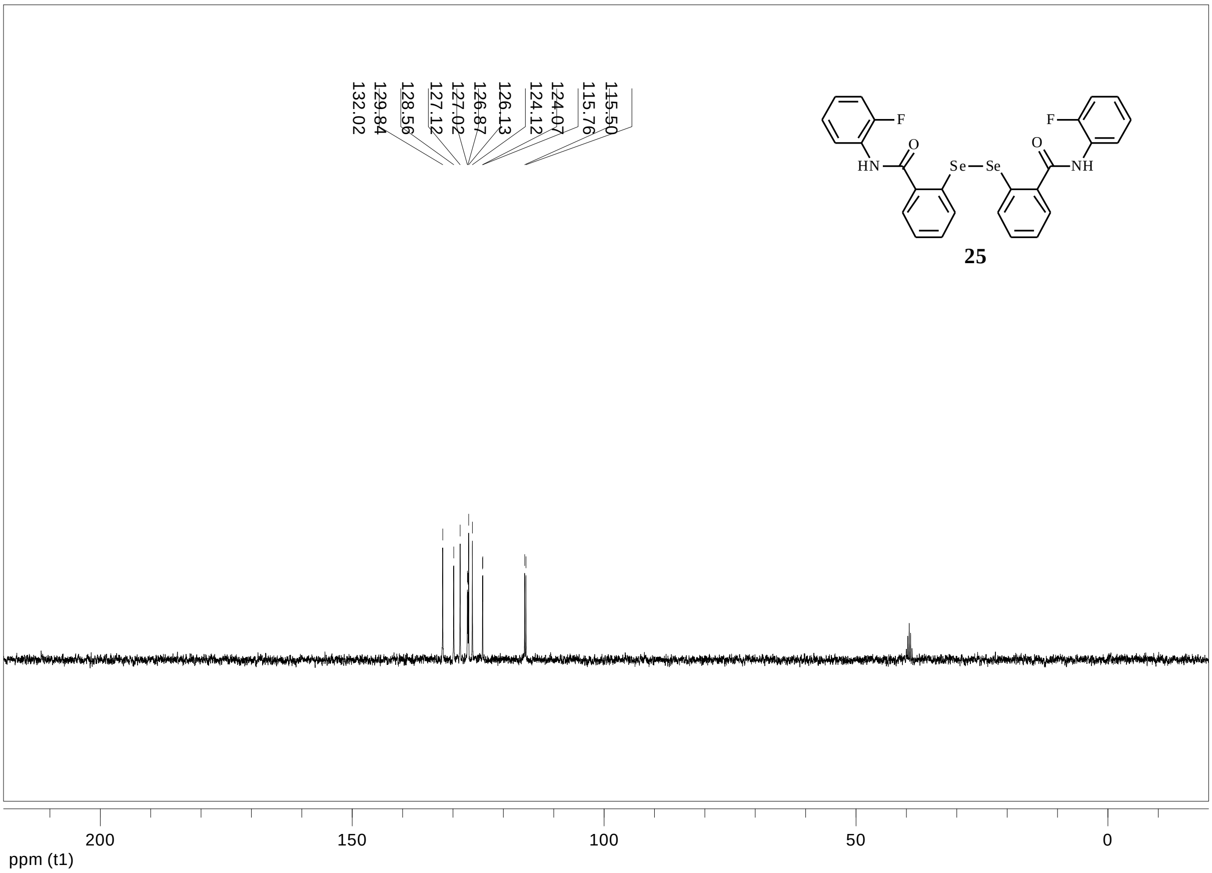

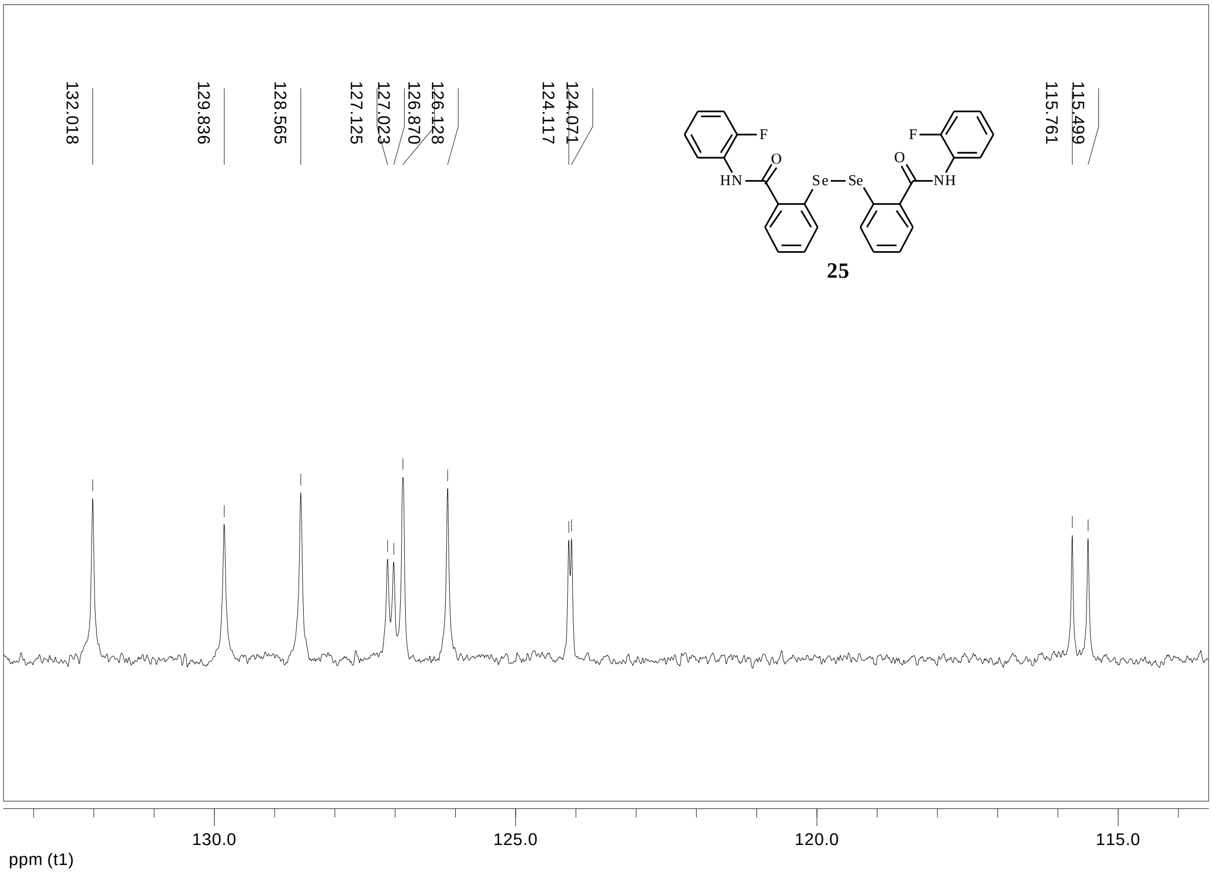

Fig. S121. ^{13}C-NMR (75.47 MHz, DMSO-d_6) dept-135 experiment of compound **25**

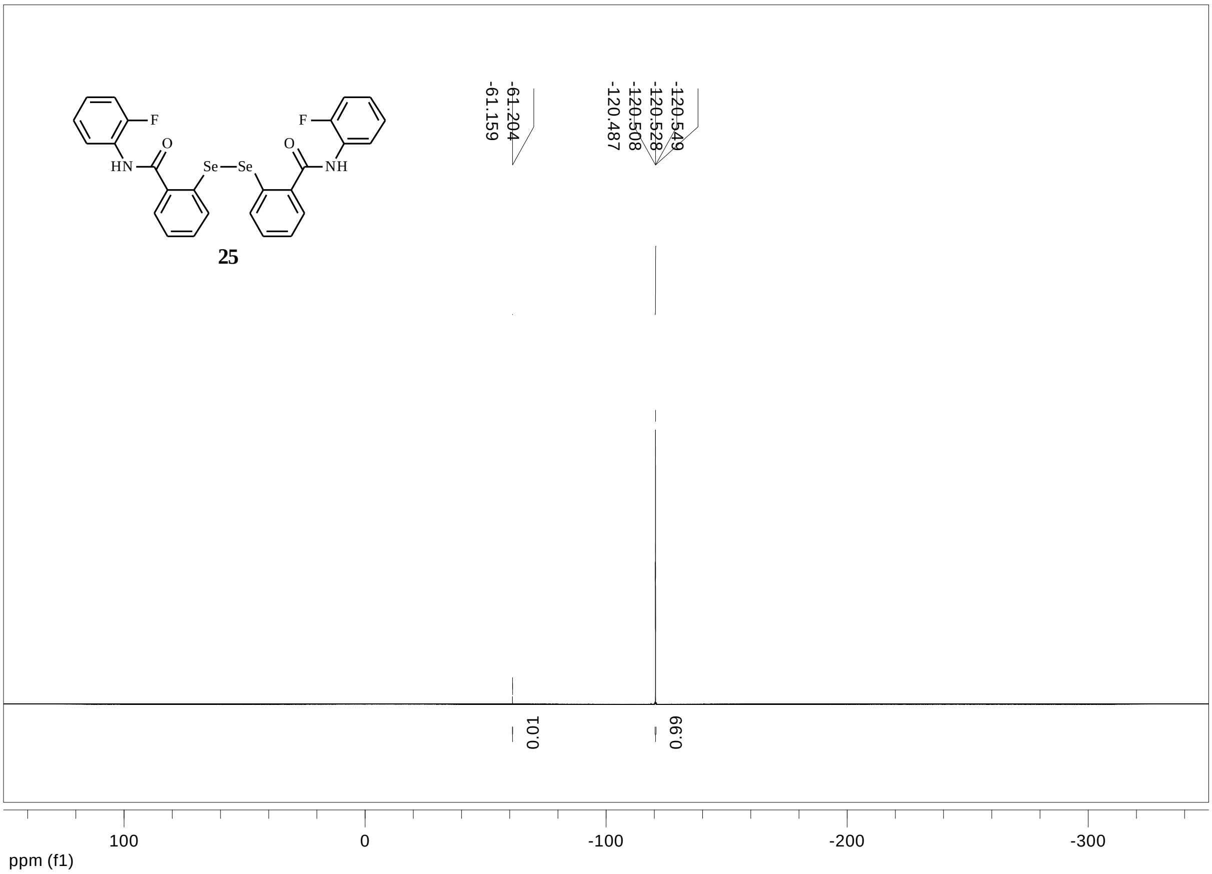

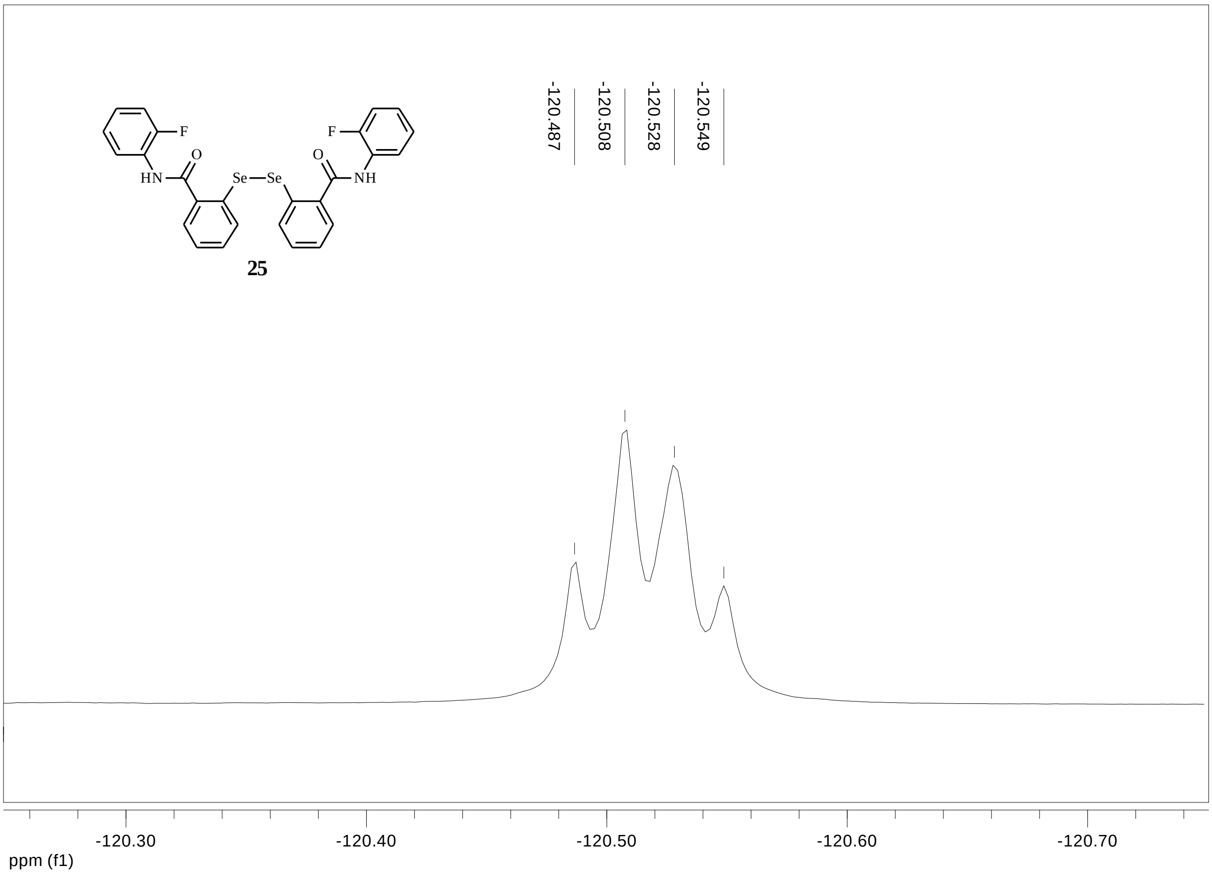

Fig. S122. ^{19}F-NMR (376.2 MHz, DMSO-d_6) spectrum of compound **25**

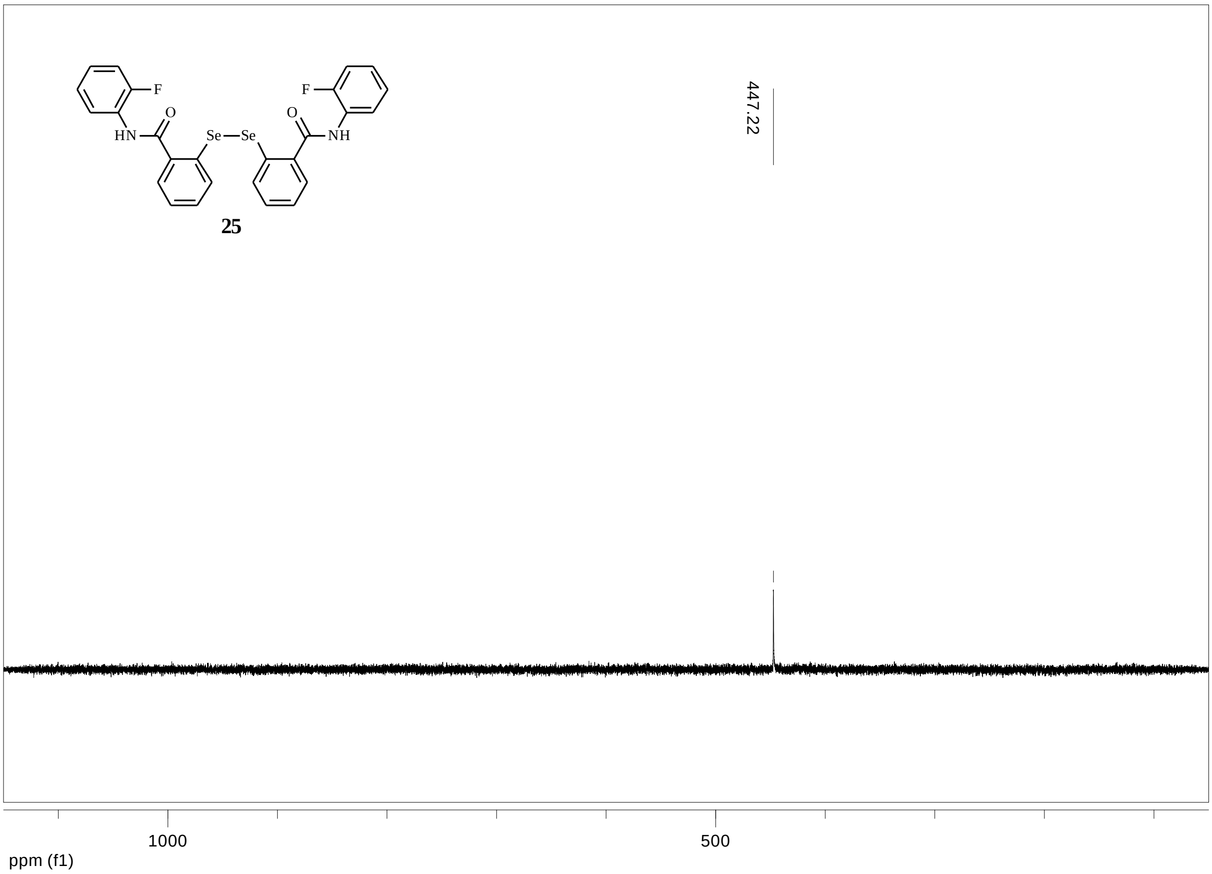

Fig. S123. ^{77}Se-NMR (76.24 MHz, DMSO-d_6) spectrum of compound **25**

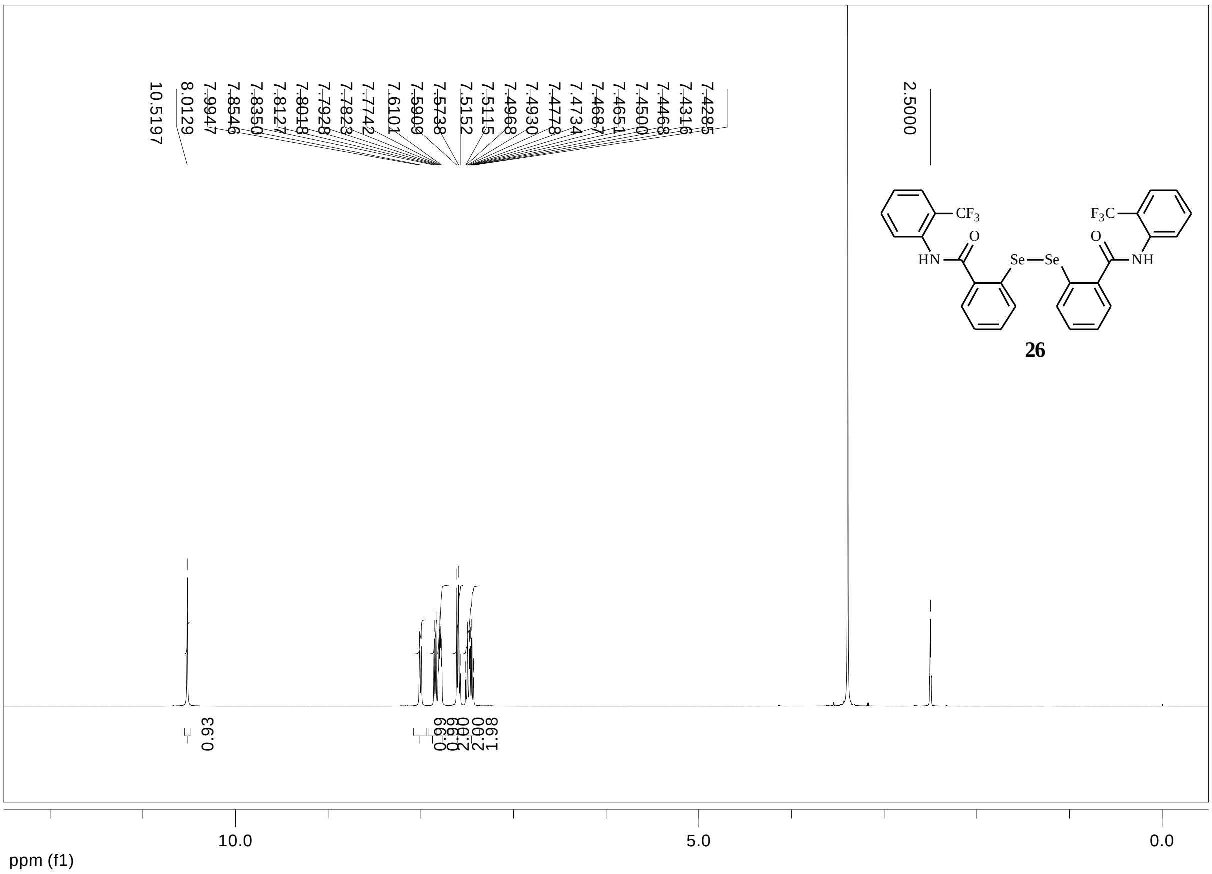

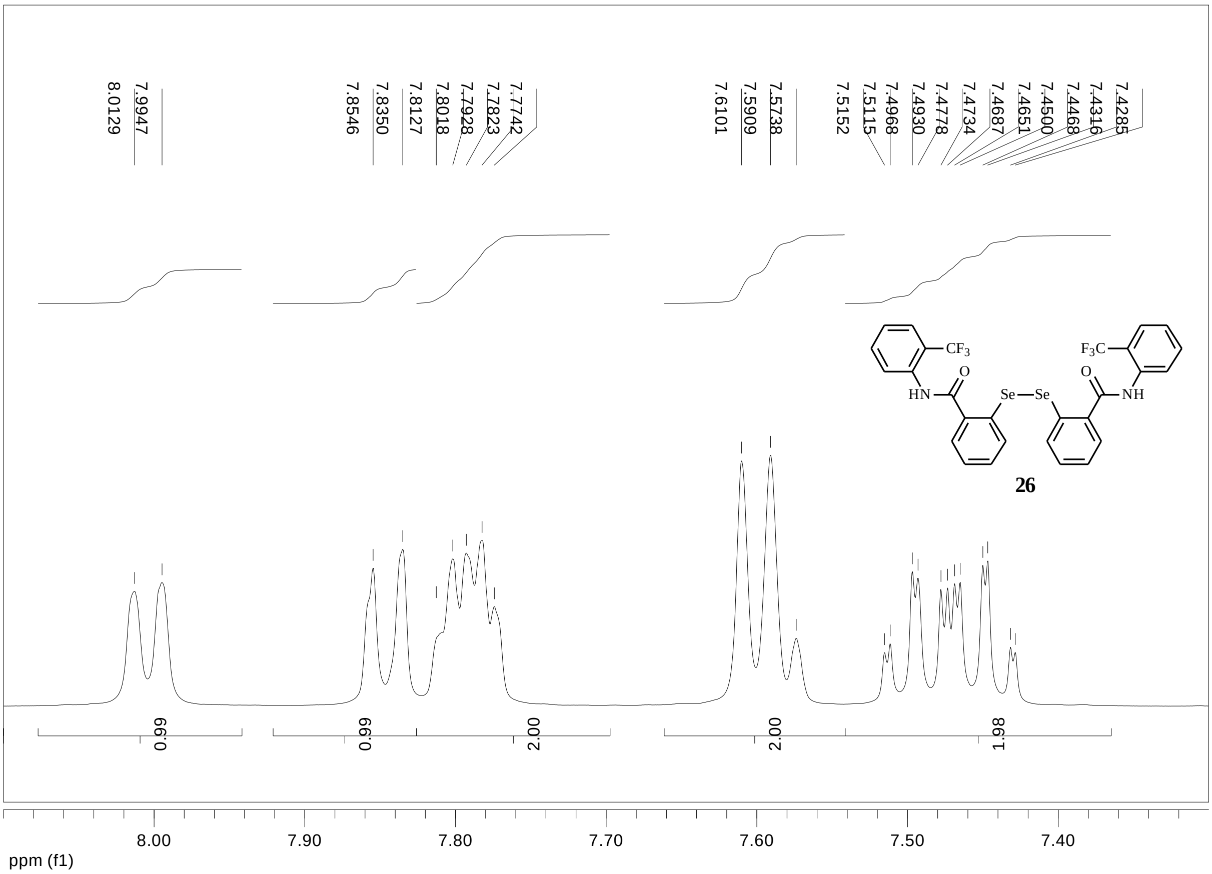

Fig. S124. ^{1}H-NMR (399.8 MHz, DMSO-d_6) spectrum of compound **26**

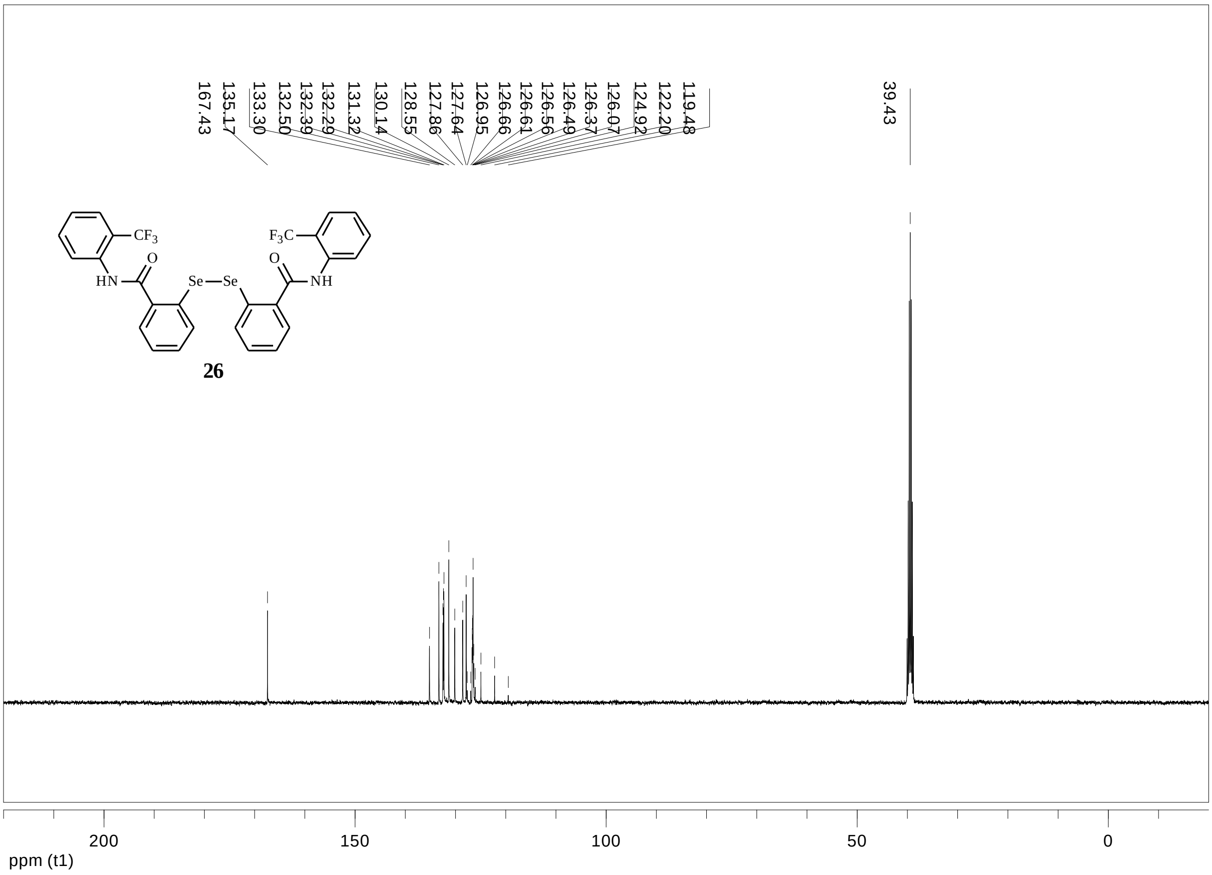

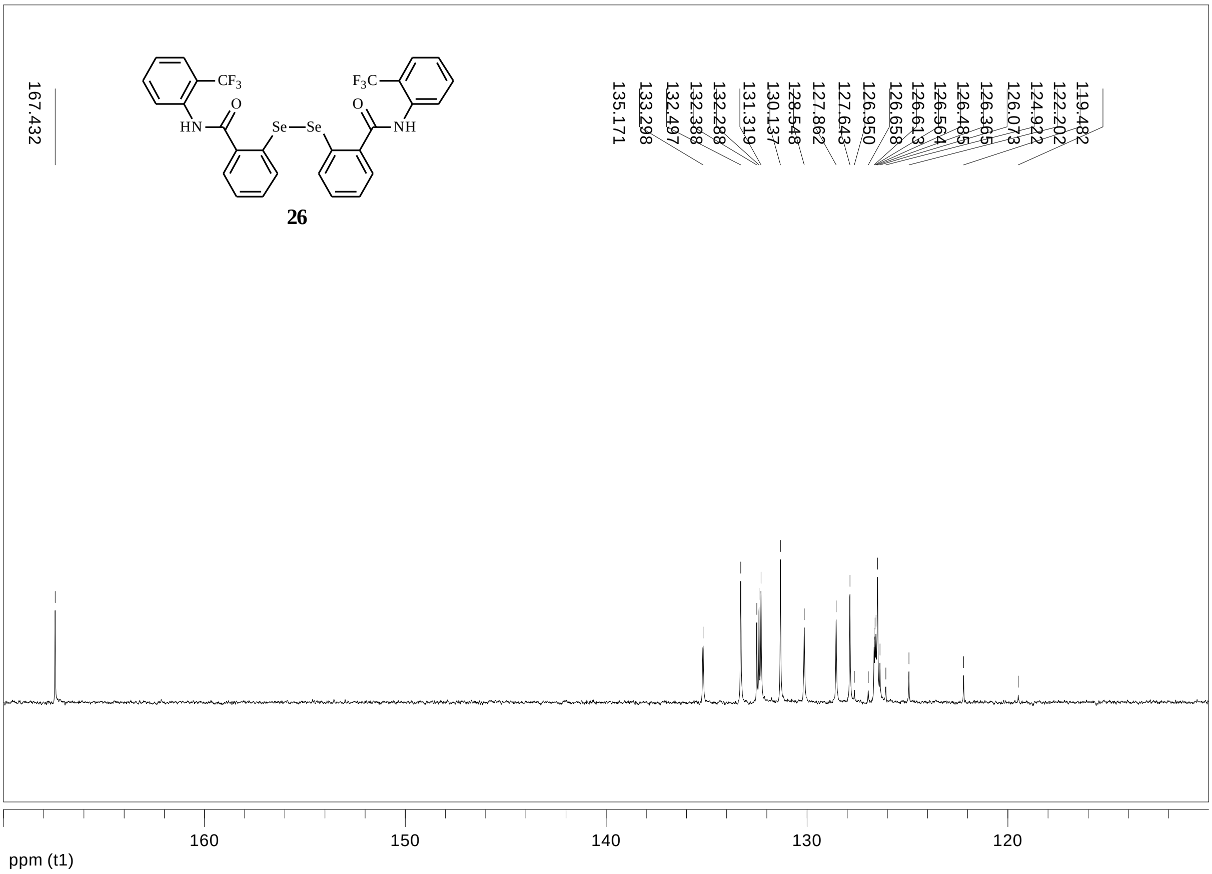

Fig. S125. ^{13}C-NMR (100.5 MHz, DMSO-d_6) spectrum of compound **26**

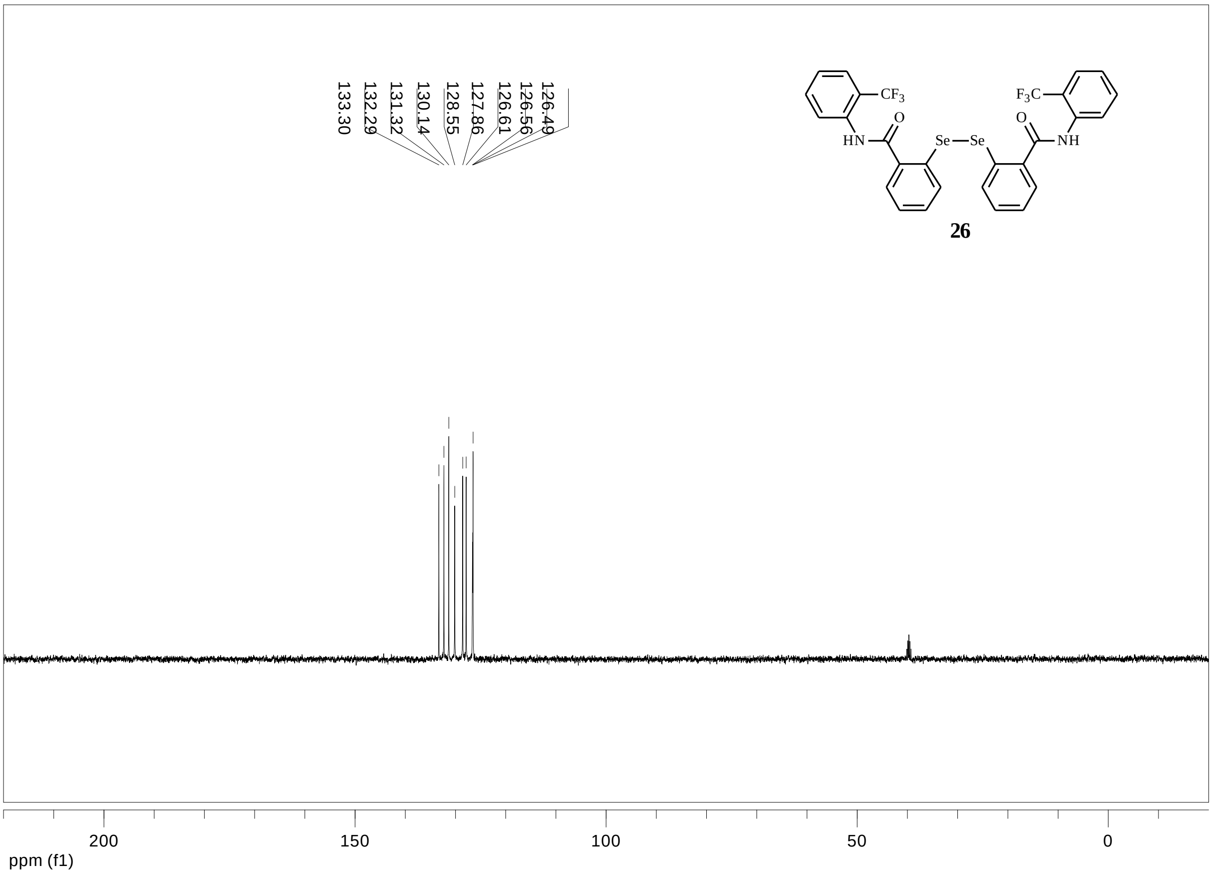

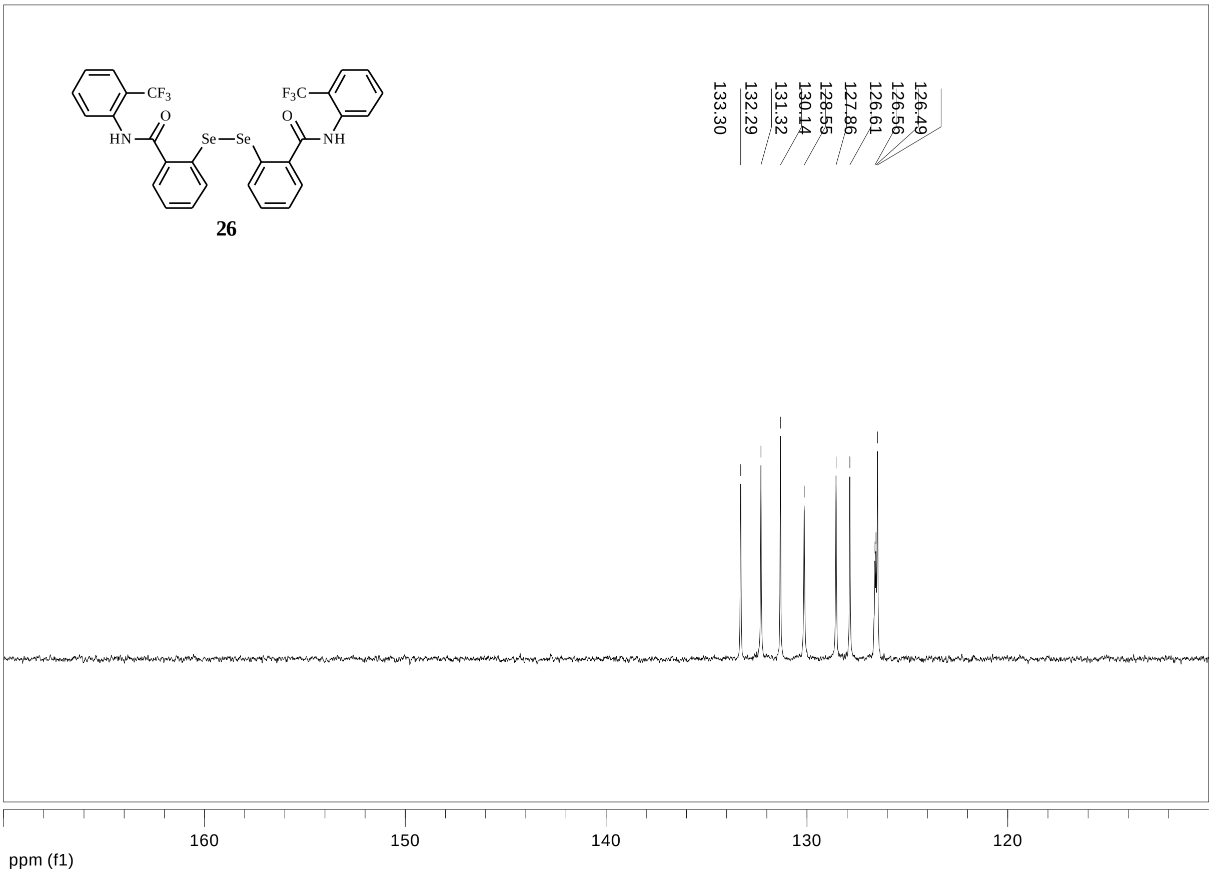

Fig. S126. ^{13}C-NMR (100.5 MHz, DMSO-d_6) dept-135 experiment of compound **26**

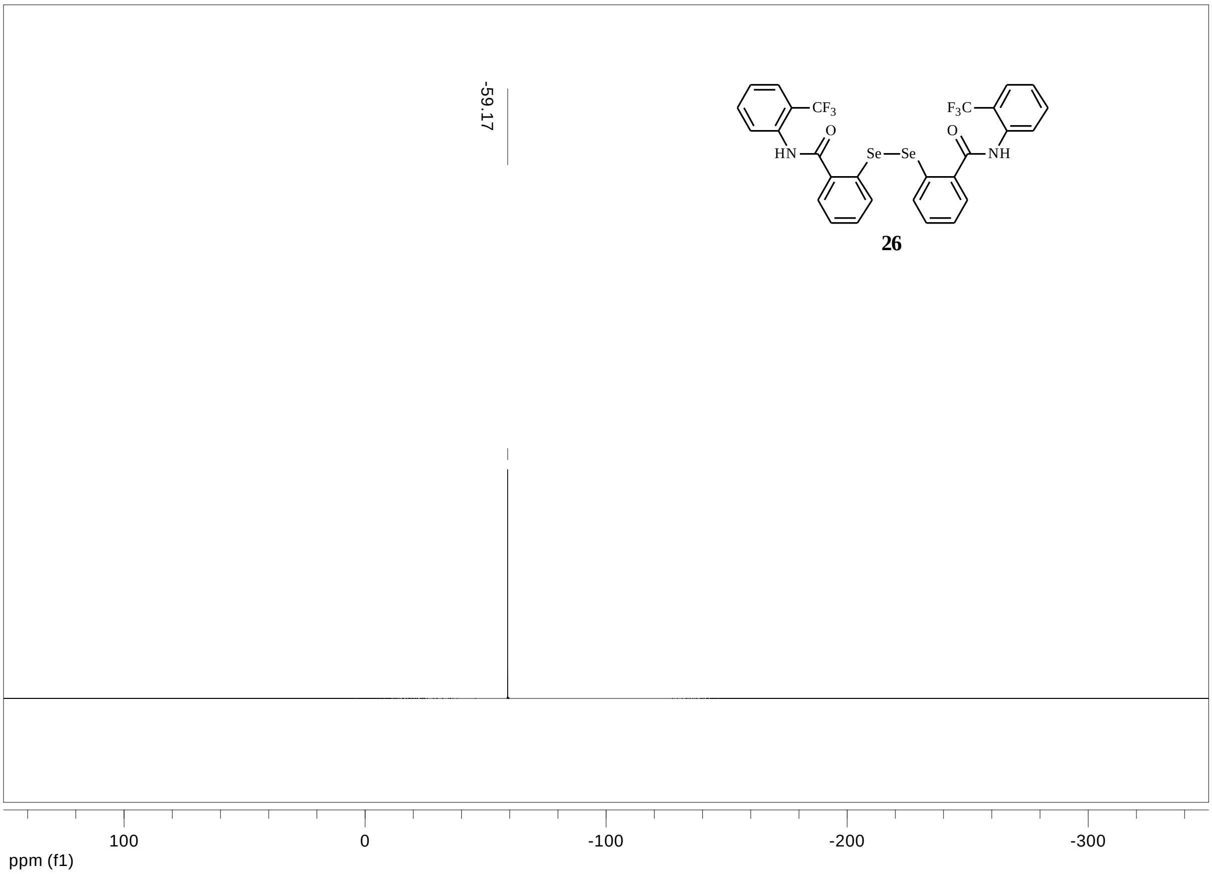

Fig. S127. ^{19}F-NMR (376.2 MHz, DMSO-d_6) spectrum of compound **26**

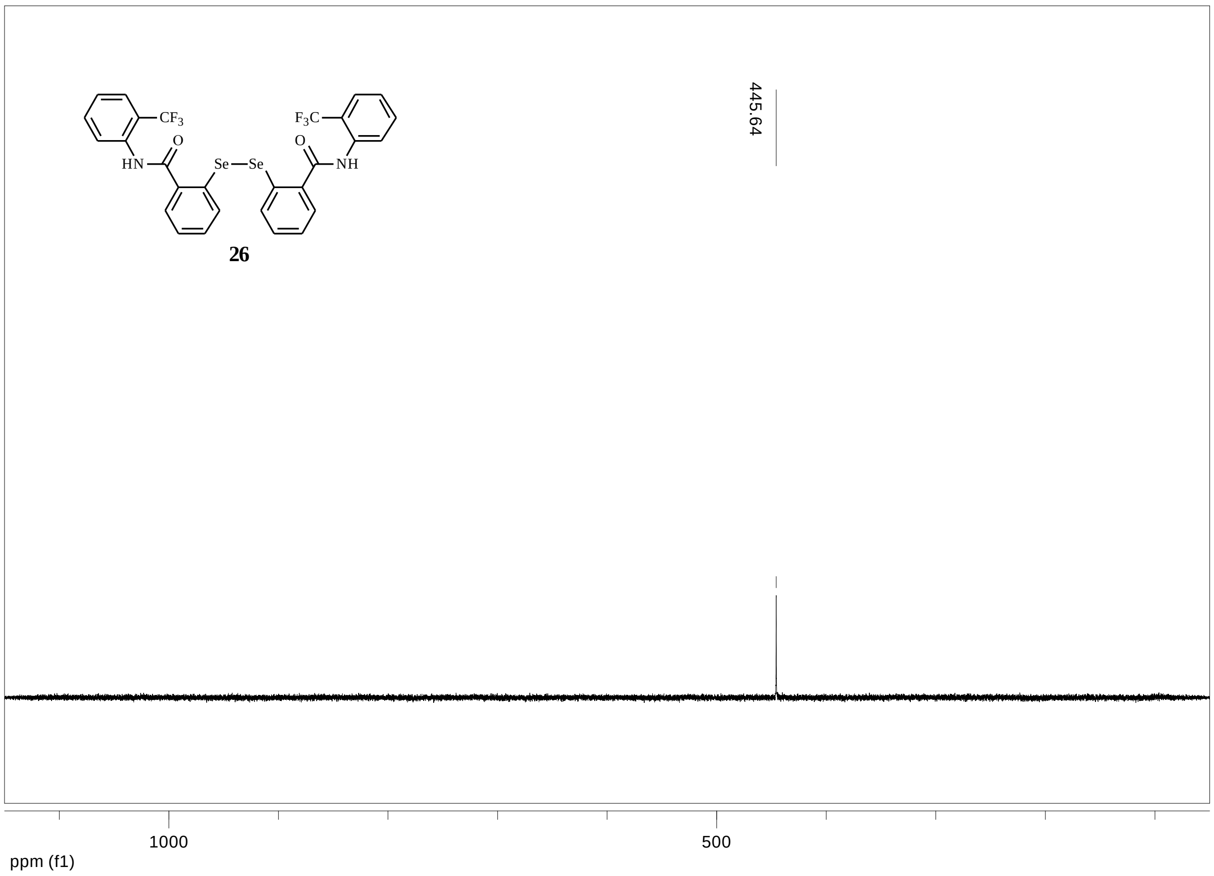

Fig. S128. ^{77}Se-NMR (76.24 MHz, DMSO-d_6) spectrum of compound **26**

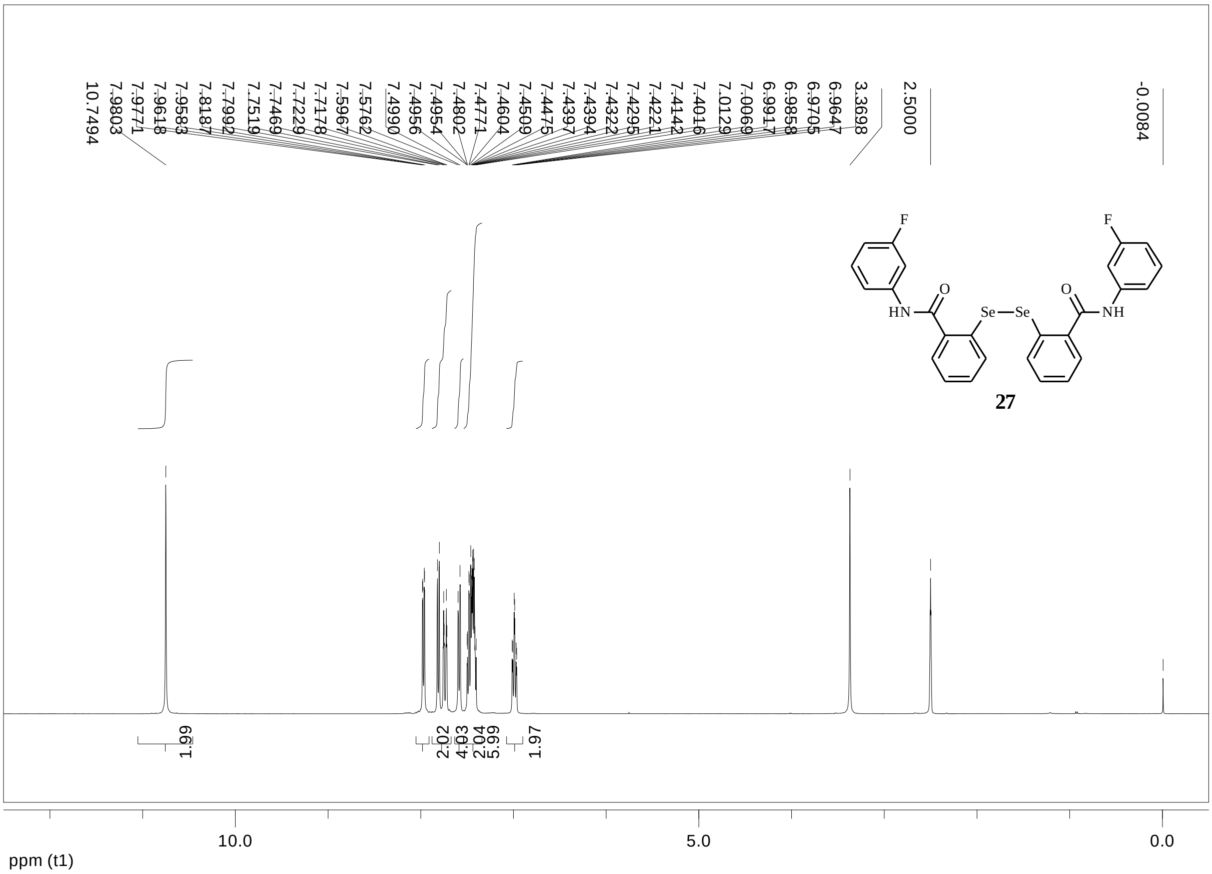

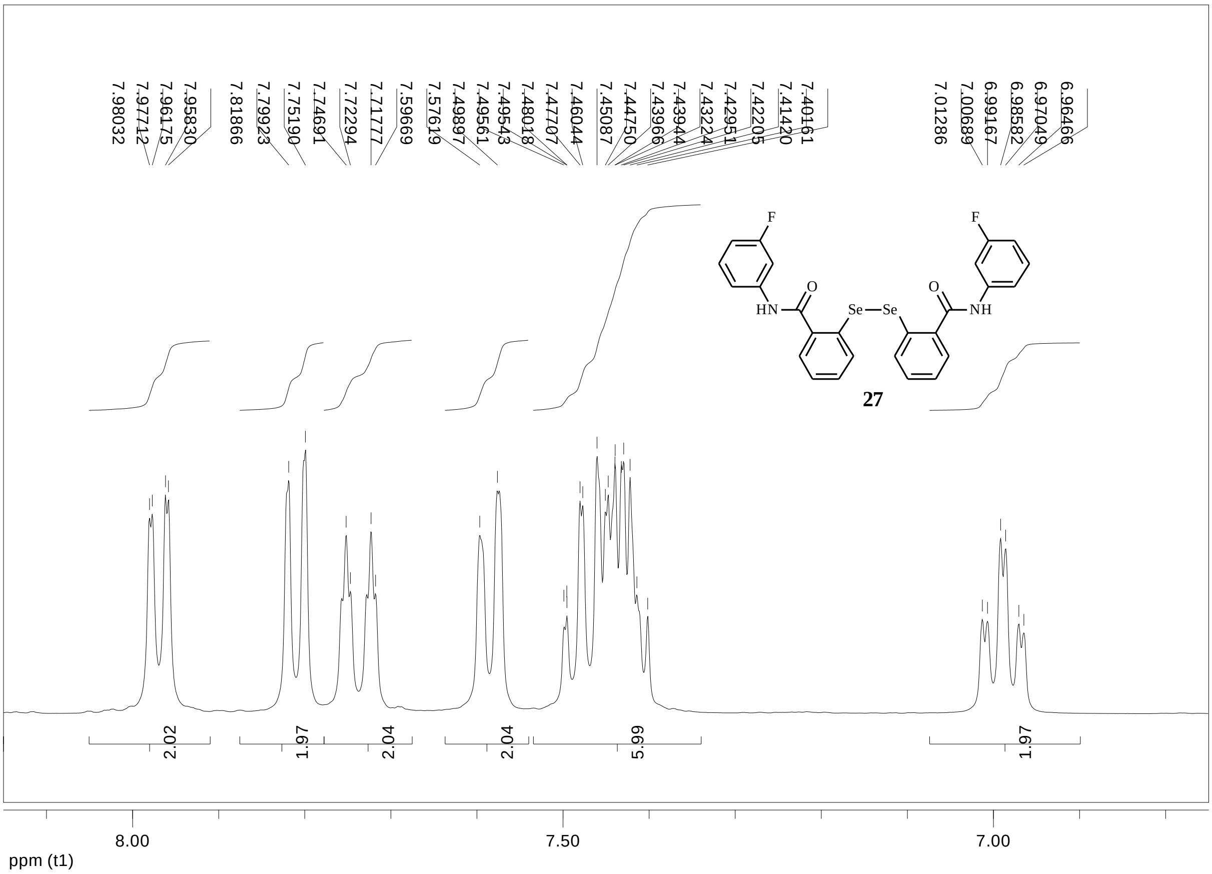

Fig. S129. ^{1}H-NMR (399.8 MHz, DMSO-d_6) spectrum of compound **27**

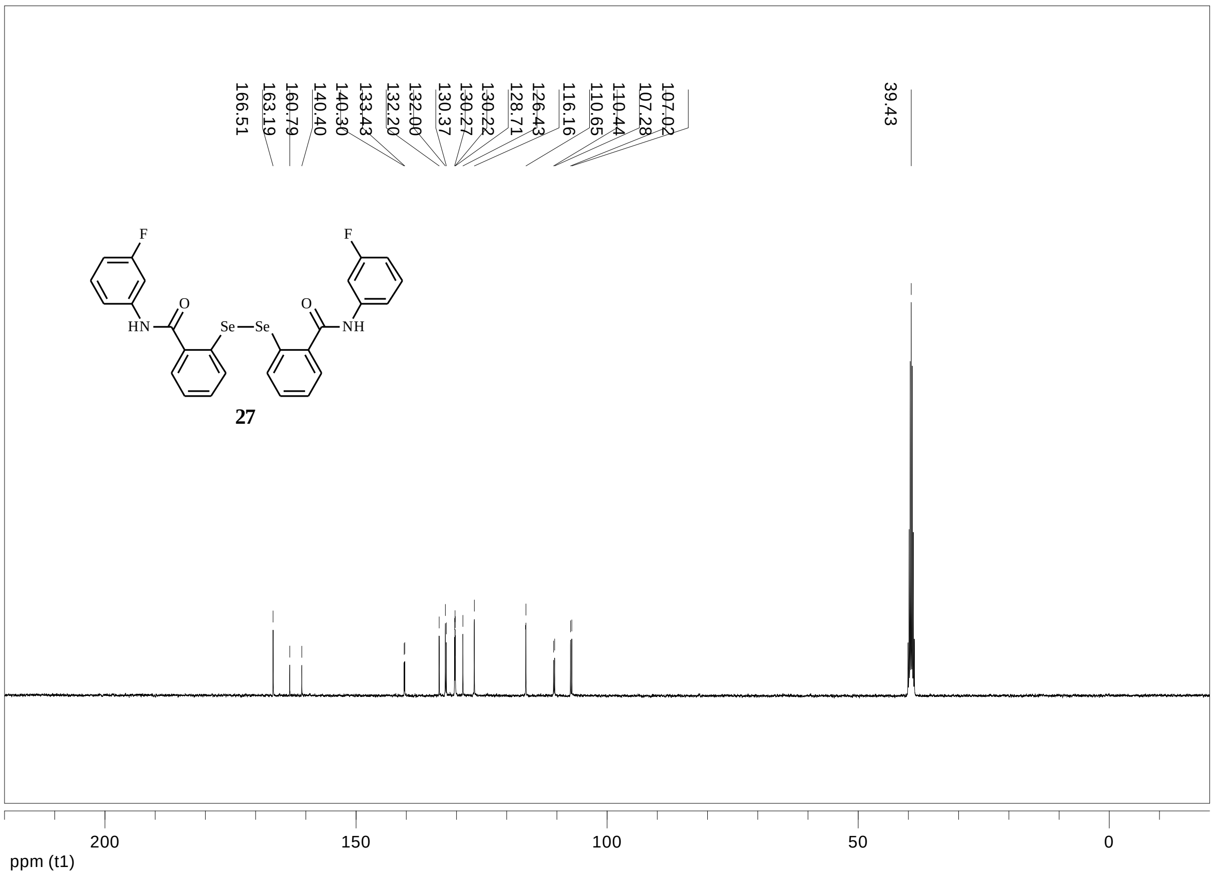

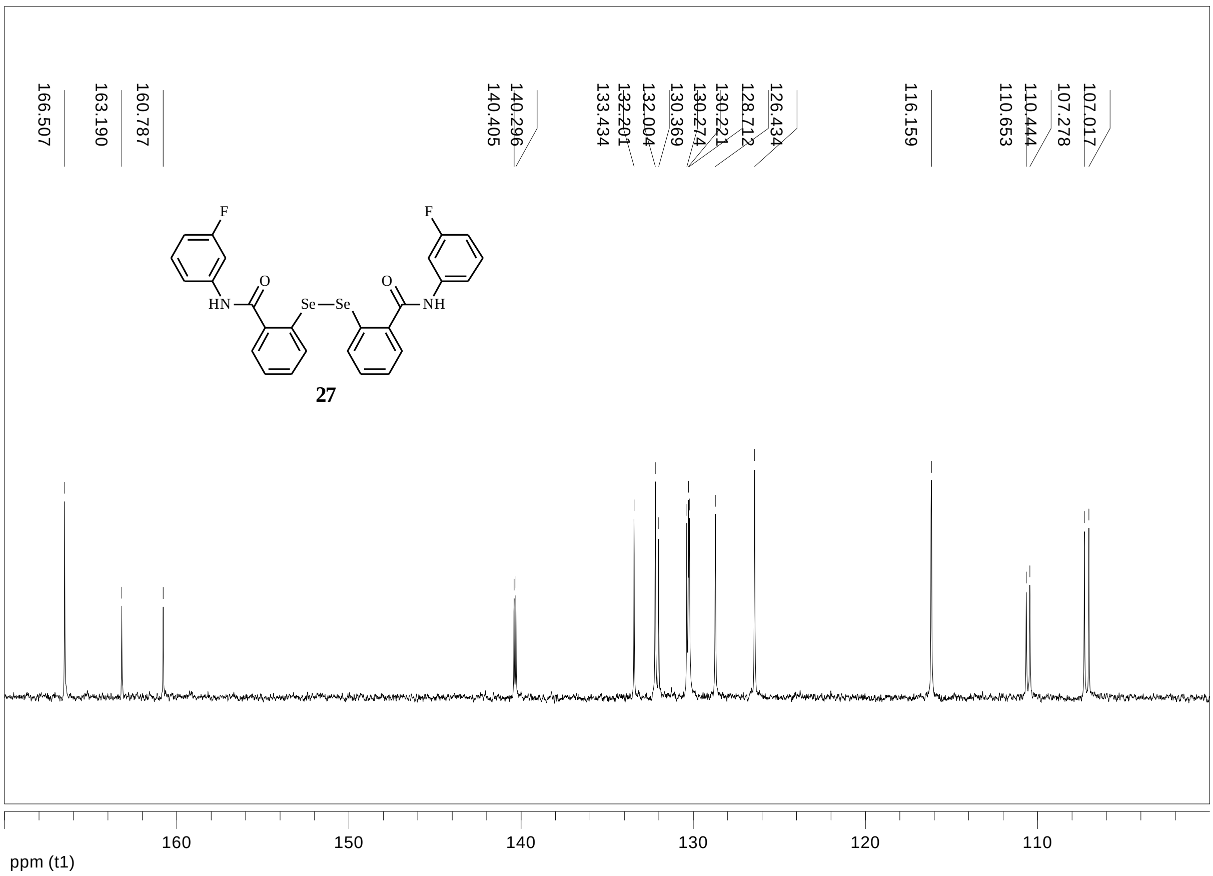

Fig. S130. ^{13}C-NMR (100.5 MHz, DMSO-d_6) spectrum of compound **27**

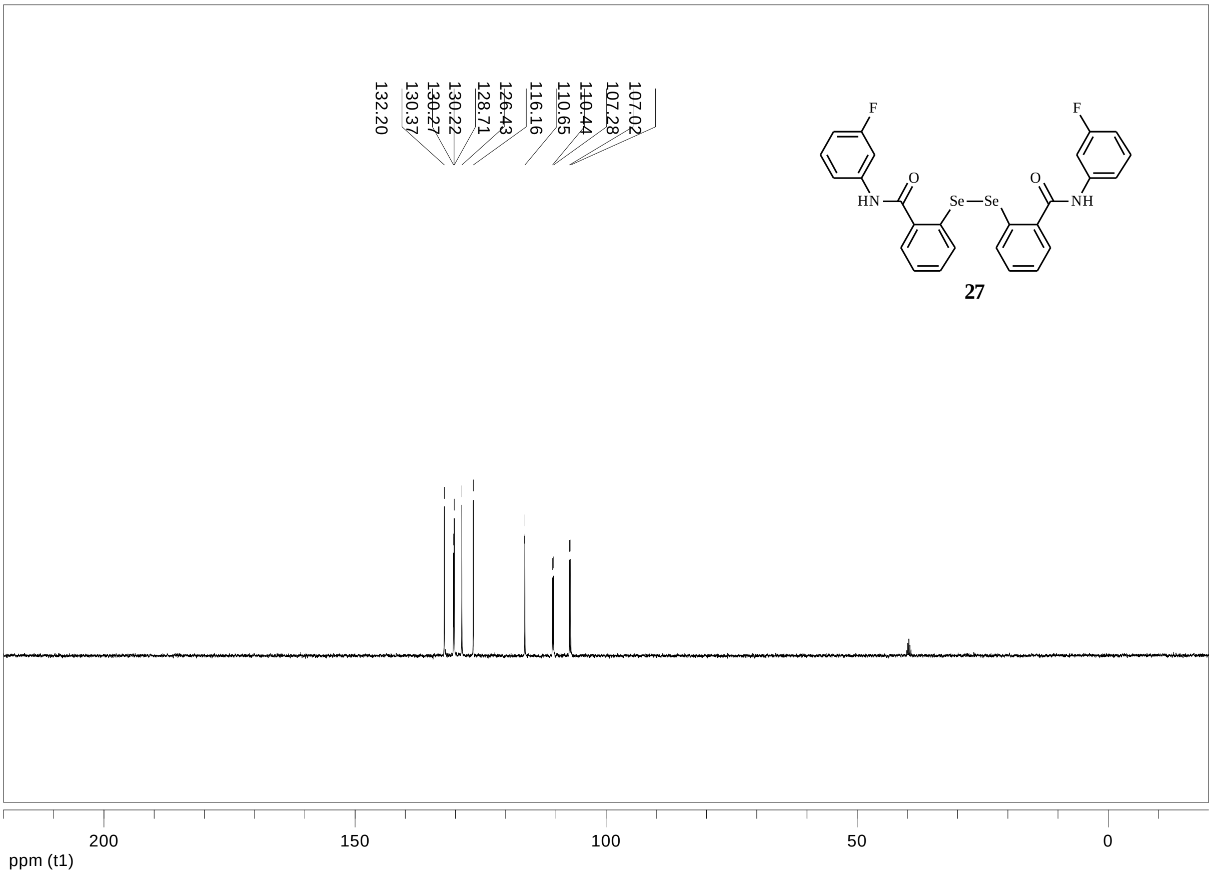

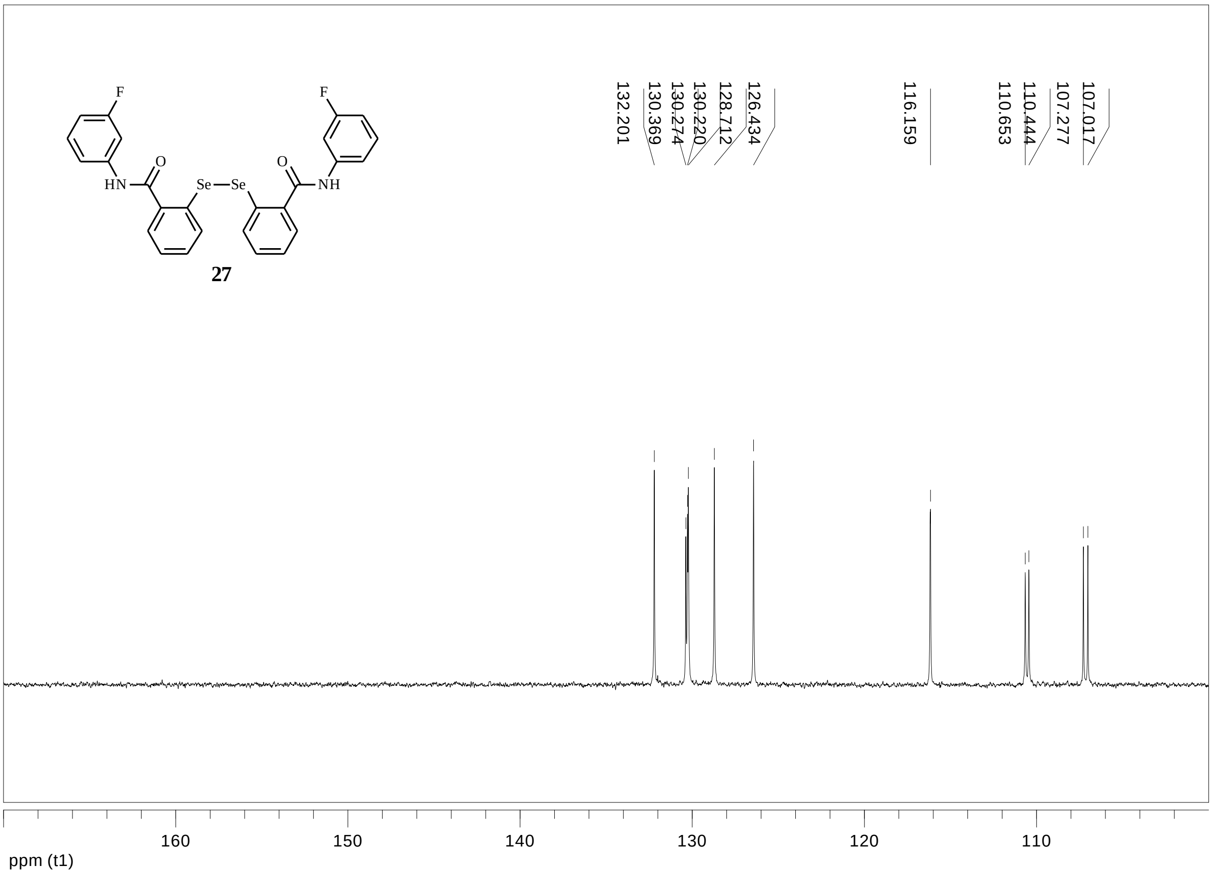

Fig. S131. ^{13}C-NMR (100.5 MHz, DMSO-d_6) dept-135 experiment of compound **27**

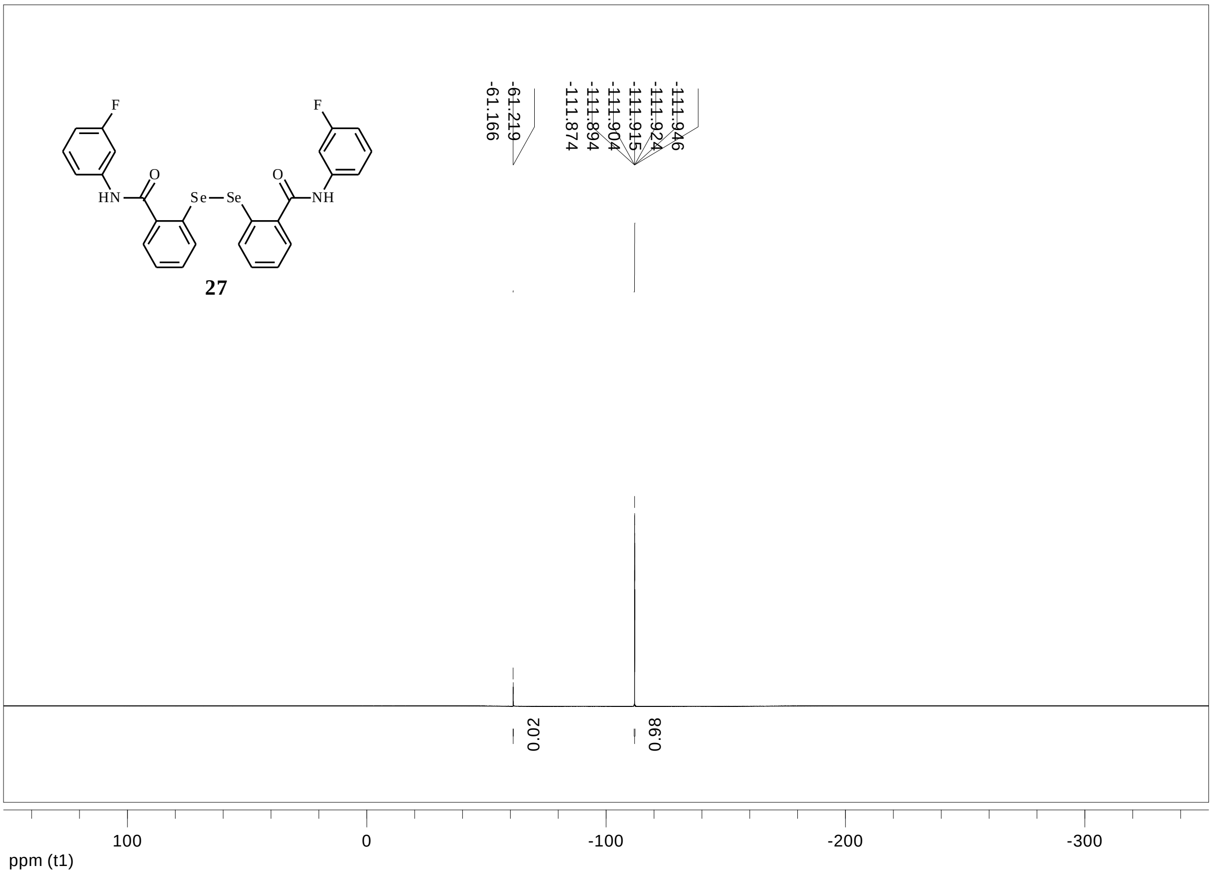

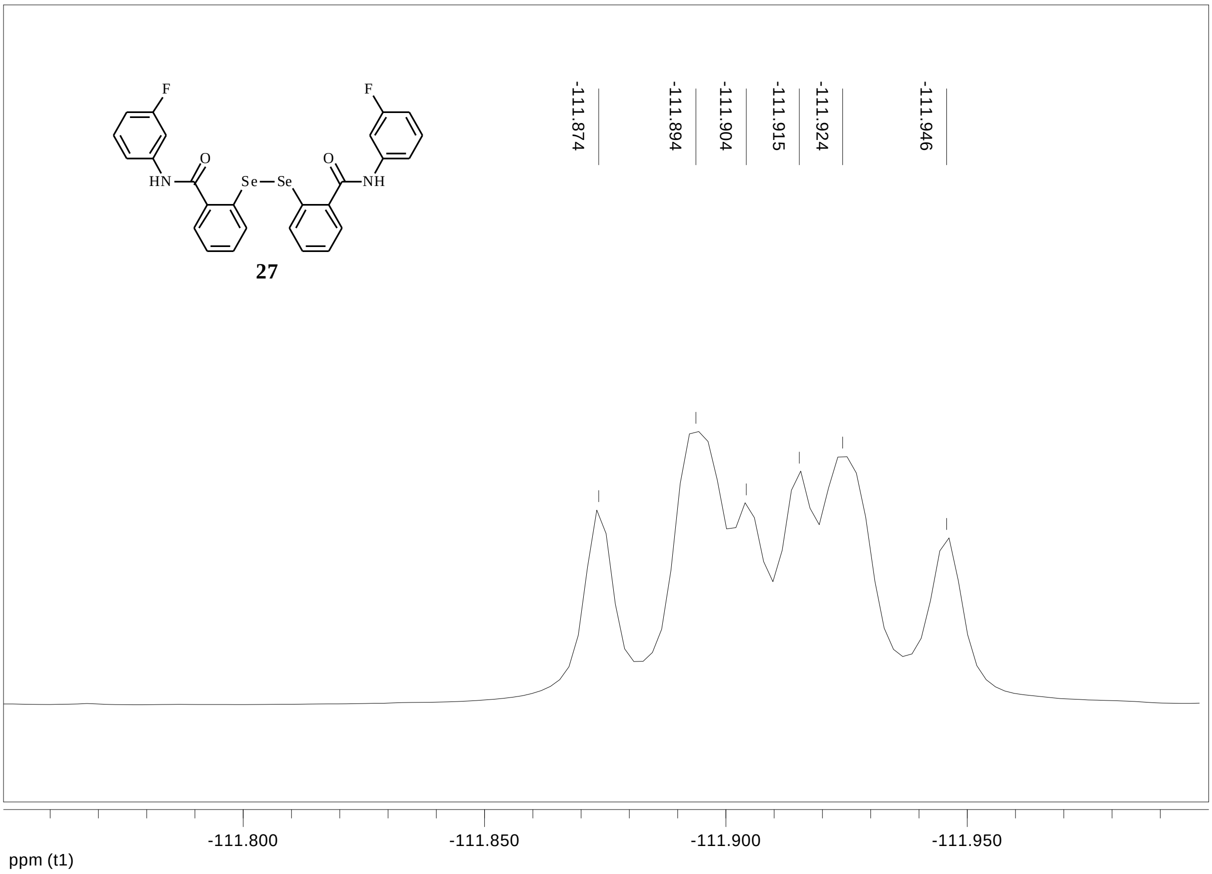

Fig. S132. ^{19}F-NMR (376.2 MHz, DMSO-d_6) spectrum of compound **27**

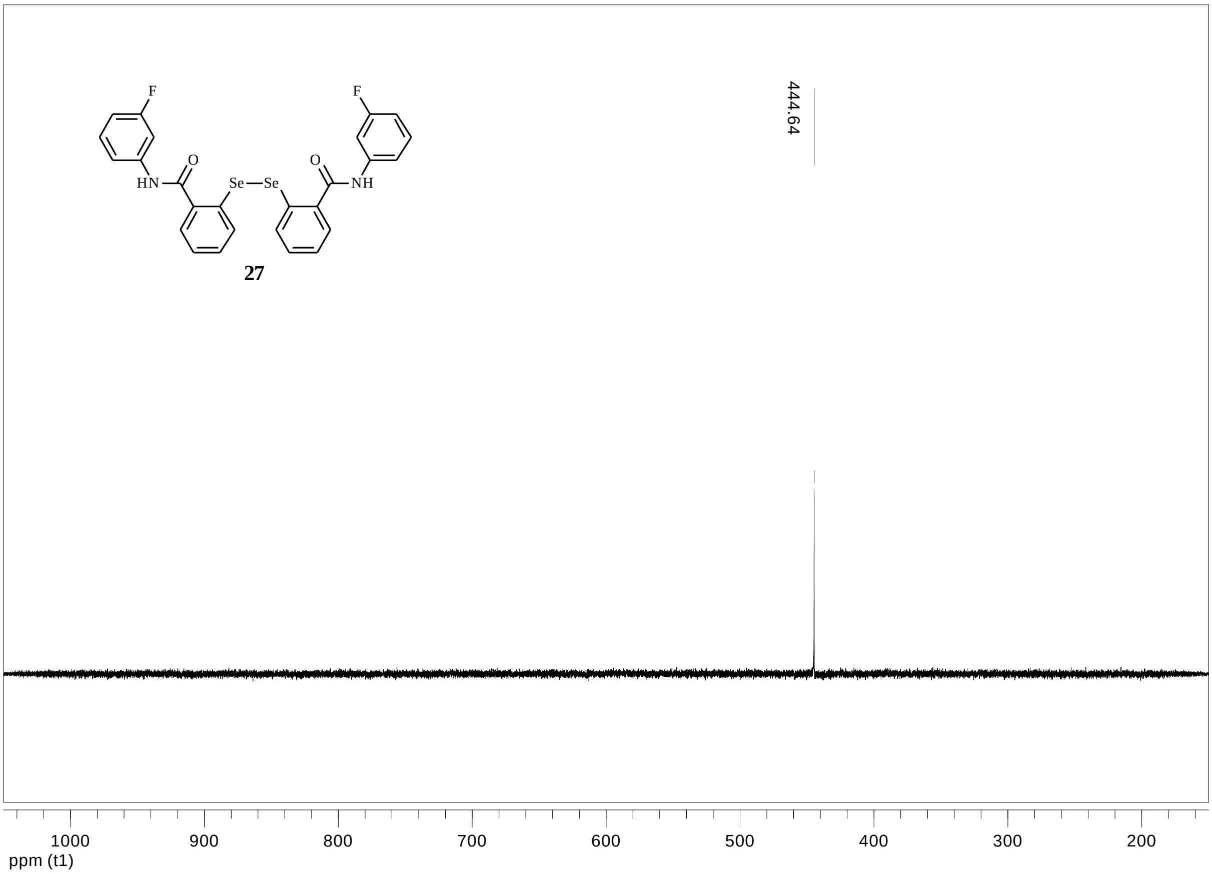

Fig. S133. ^{77}Se-NMR (76.24 MHz, DMSO-d_6) spectrum of compound **27**

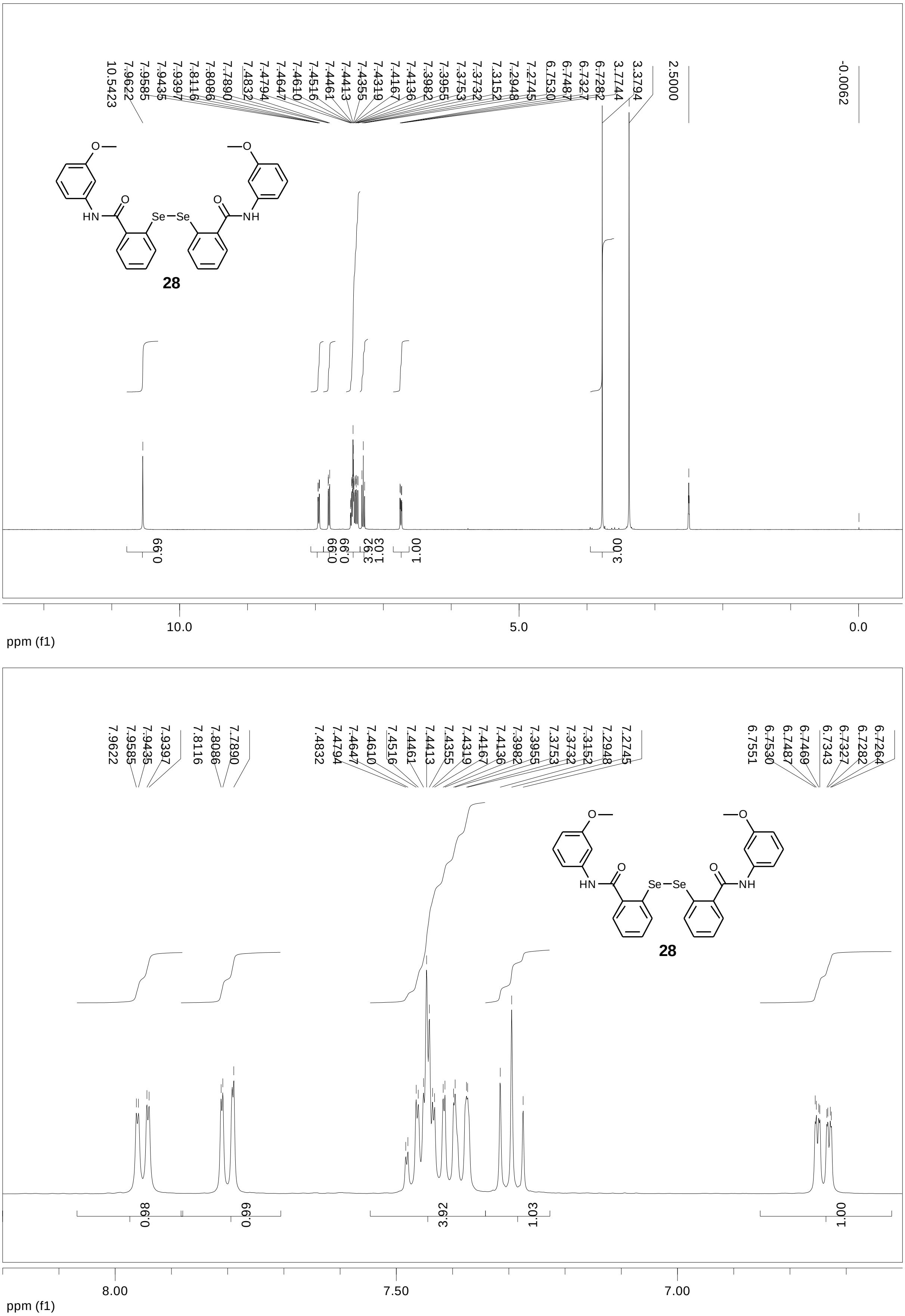

Fig. S134. ^{1}H-NMR (399.8 MHz, DMSO-d_6) spectrum of compound **28**

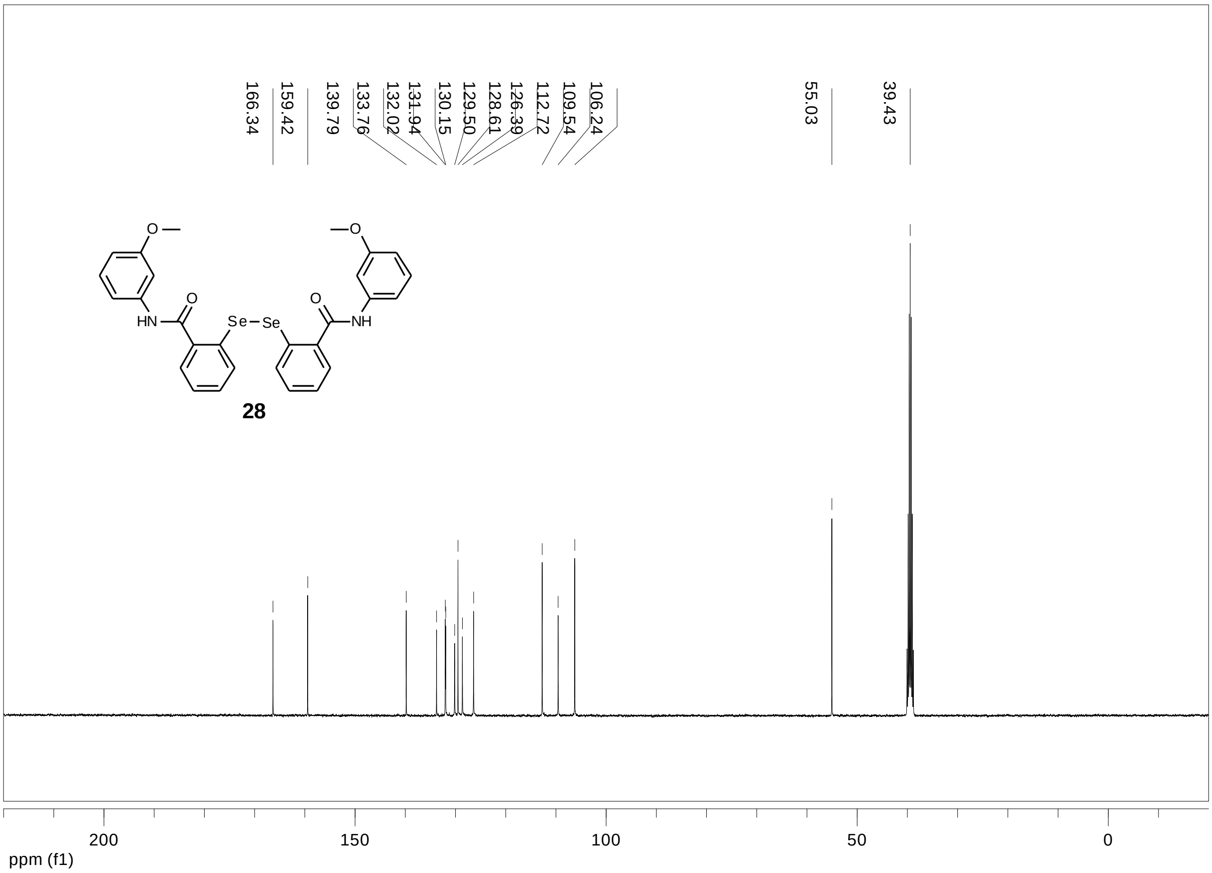

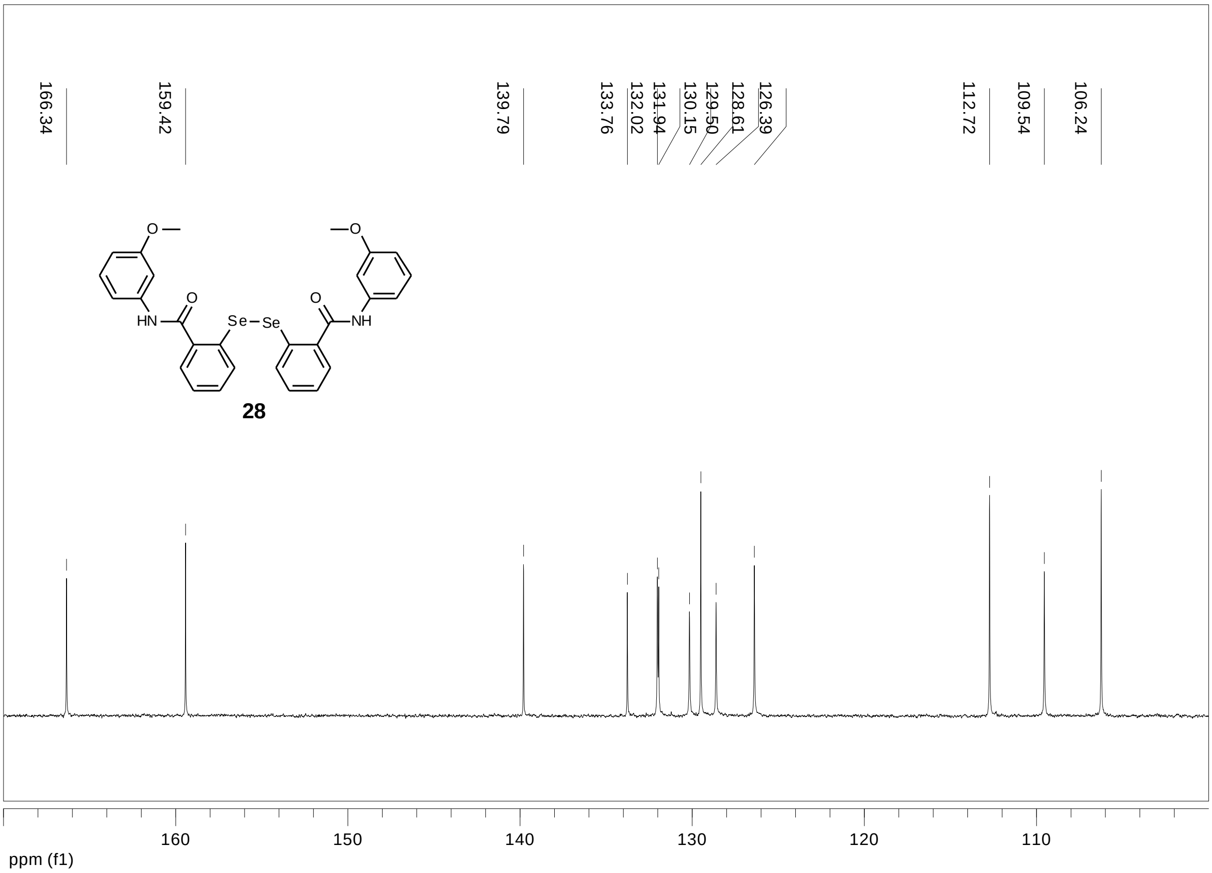

Fig. S135. ^{13}C-NMR (100.5 MHz, DMSO-d_6) spectrum of compound **28**

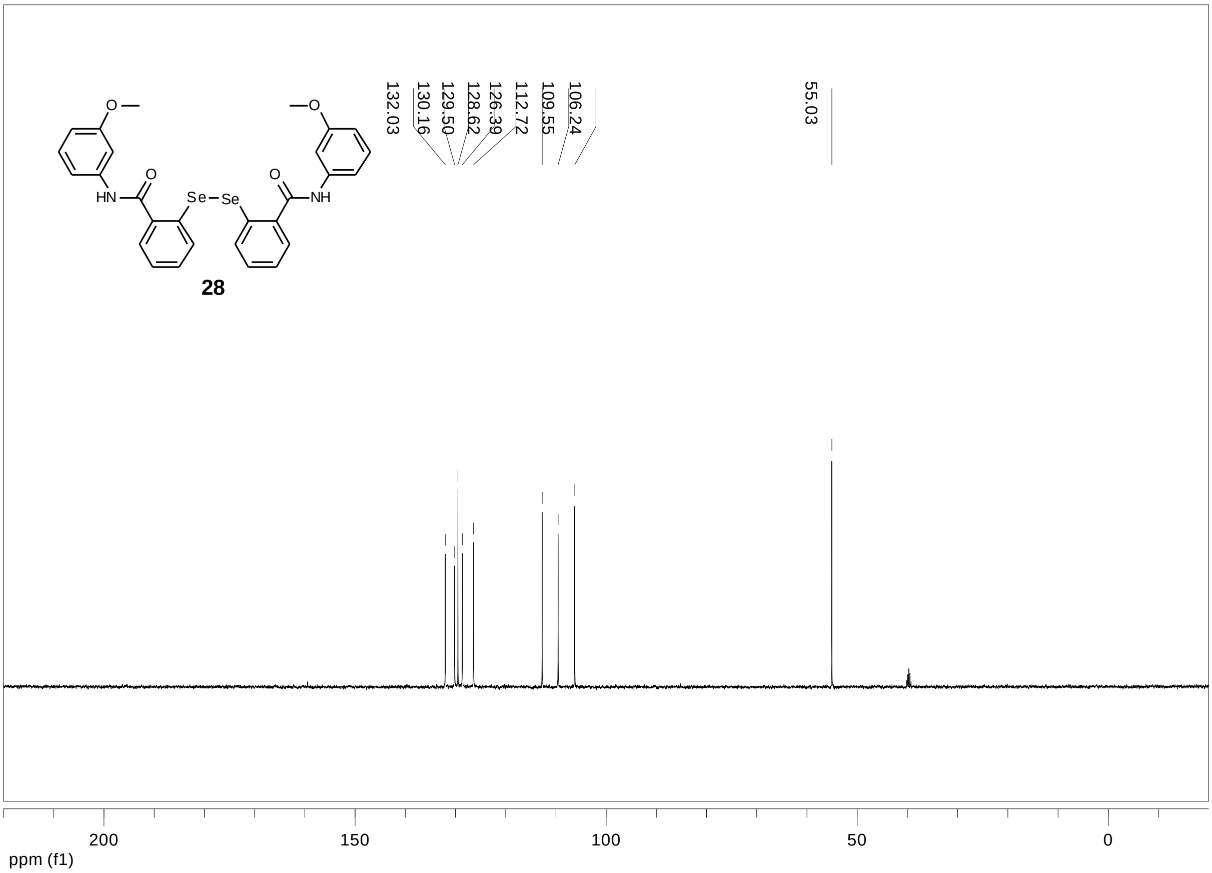

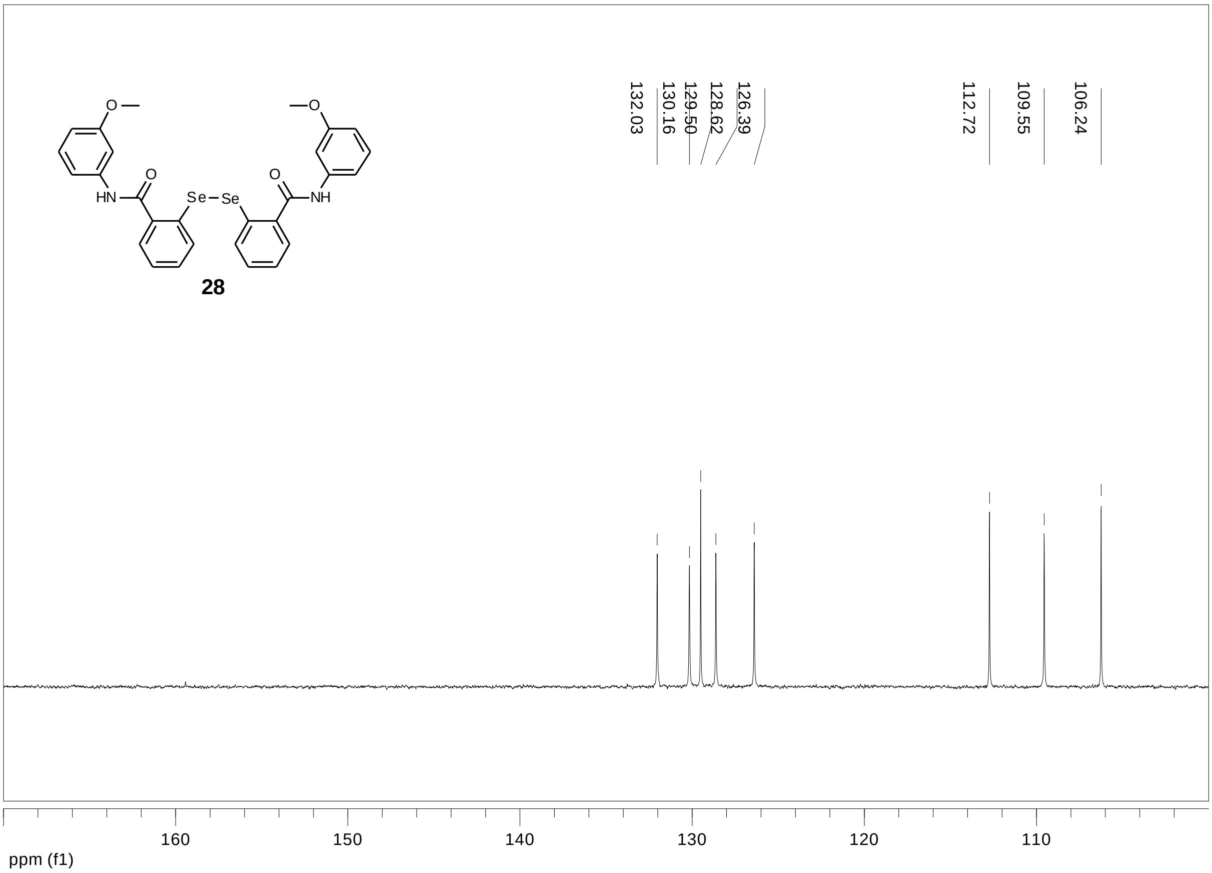

Fig. S136. ^{13}C-NMR (100.5 MHz, DMSO-d_6) dept-135 experiment of compound **28**

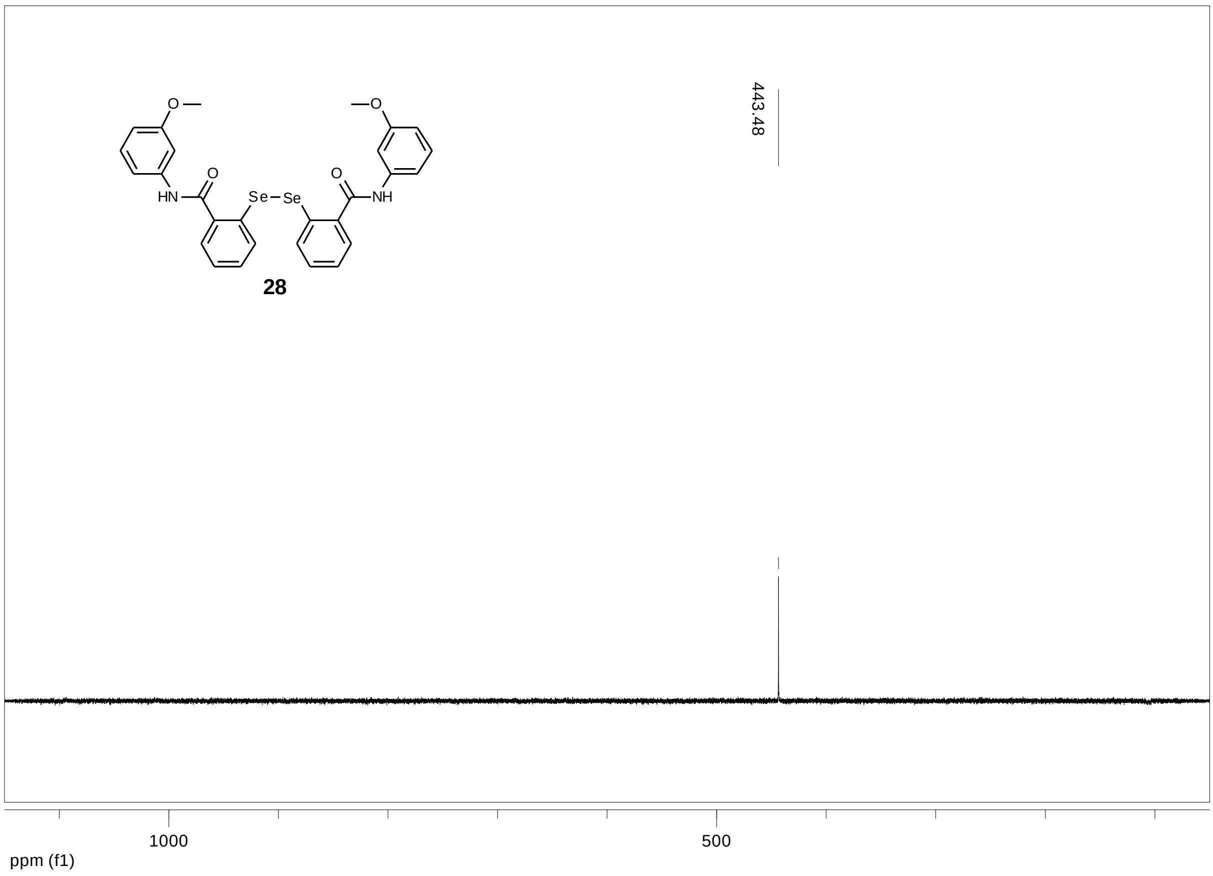

Fig. S137. ^{77}Se-NMR (76.24 MHz, DMSO-d_6) spectrum of compound **28**

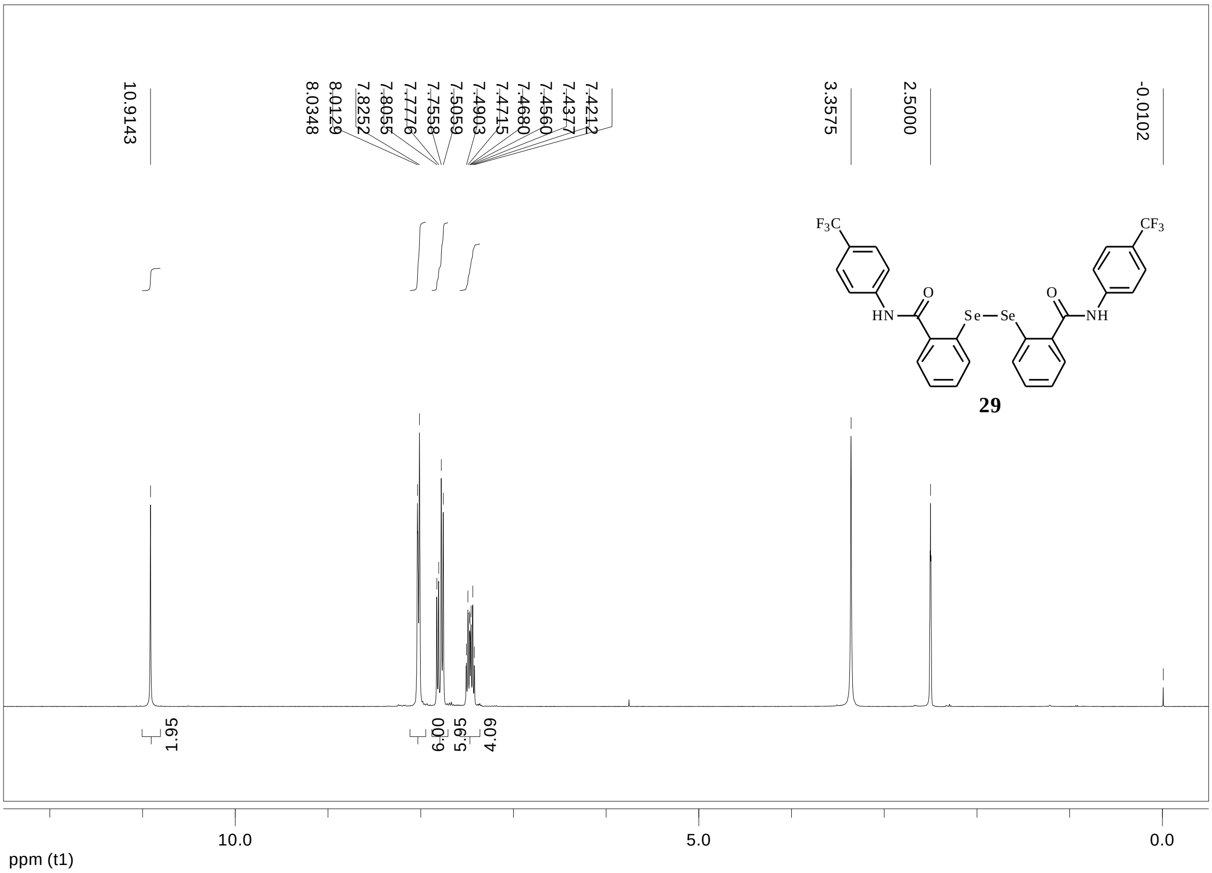

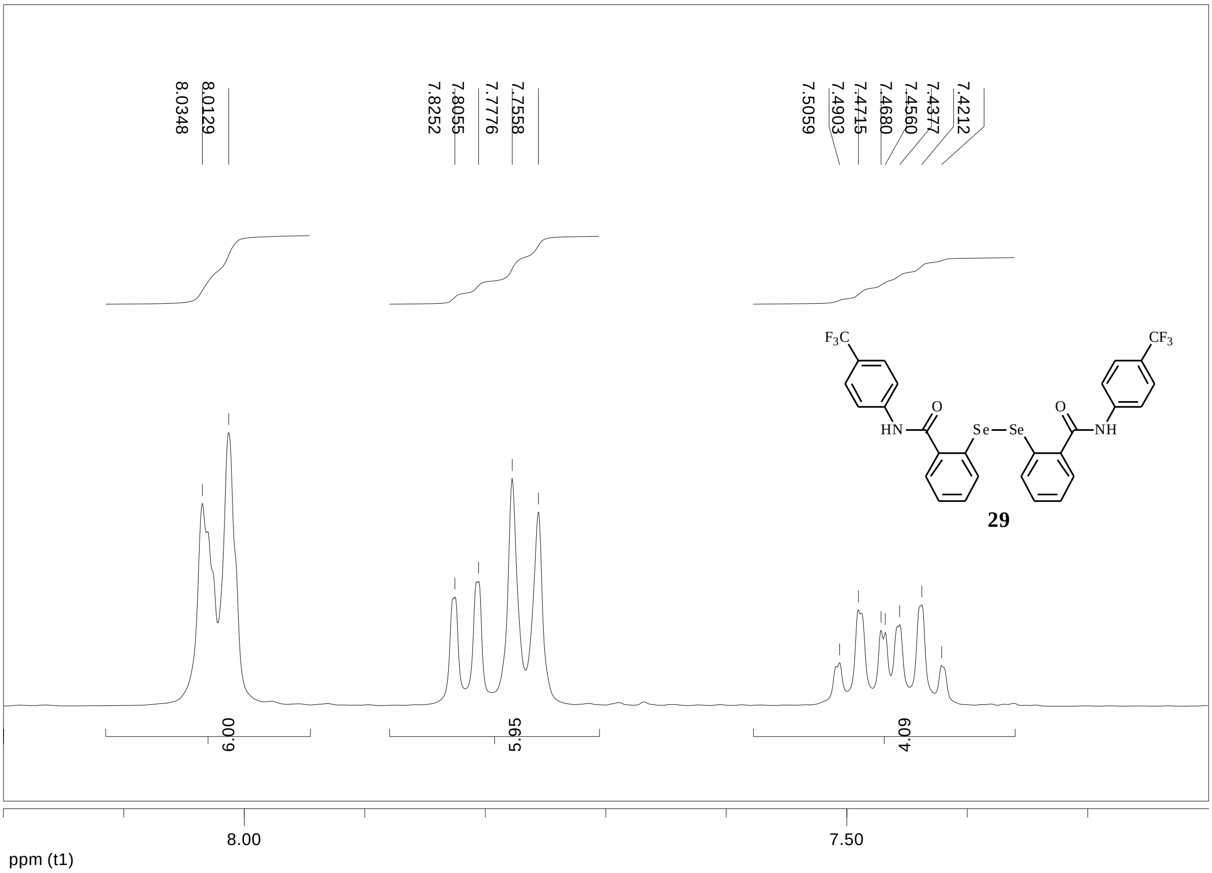

Fig. S138. ^{1}H-NMR (399.8 MHz, DMSO-d_6) spectrum of compound **29**

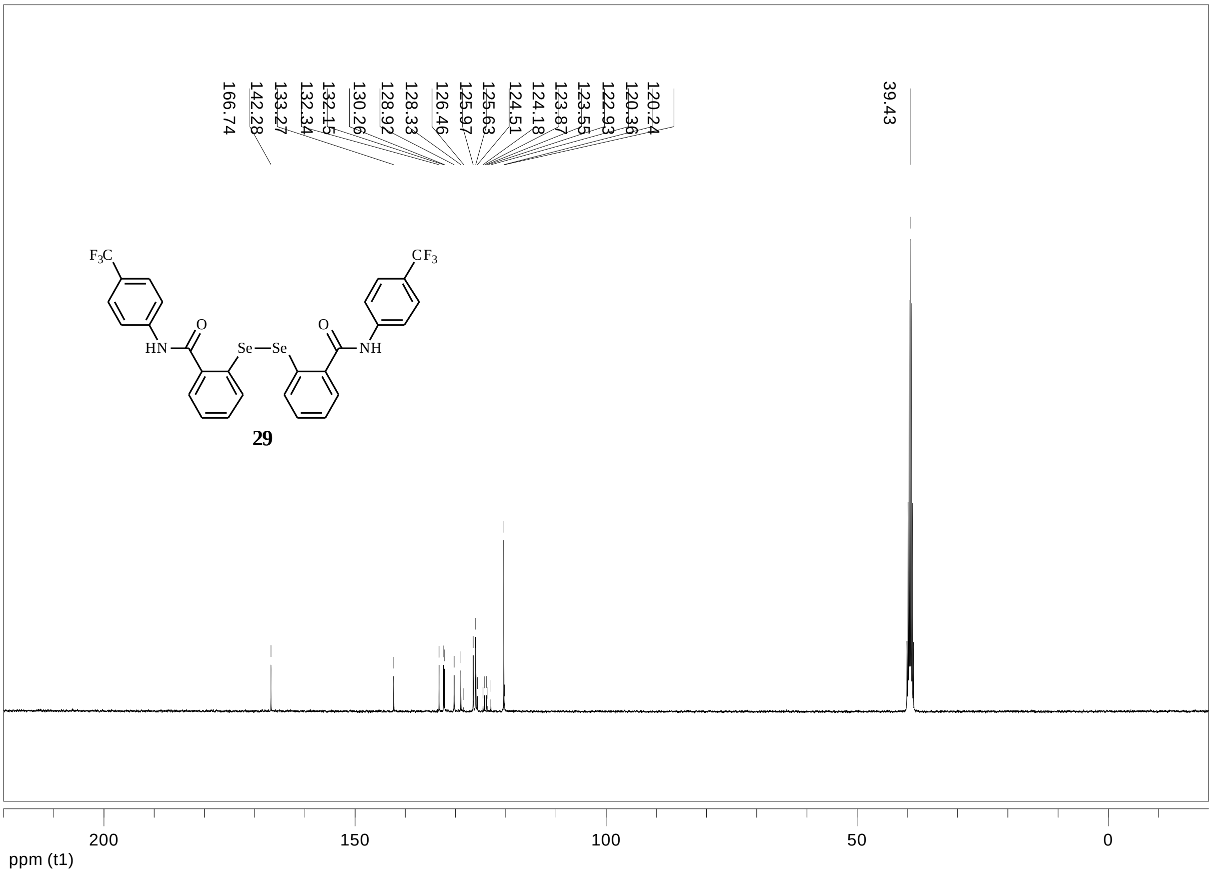

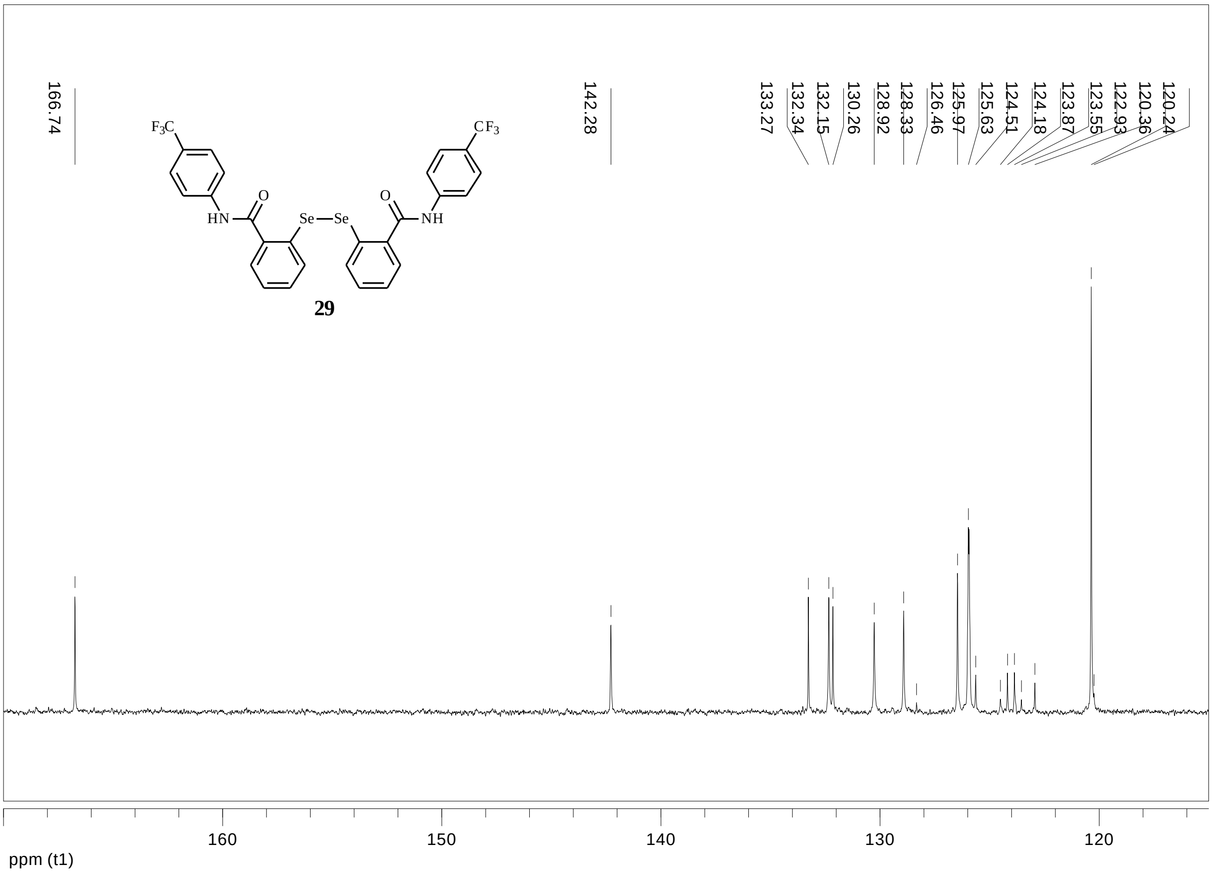

Fig. S139. ^{13}C-NMR (100.5 MHz, DMSO-d_6) spectrum of compound **29**

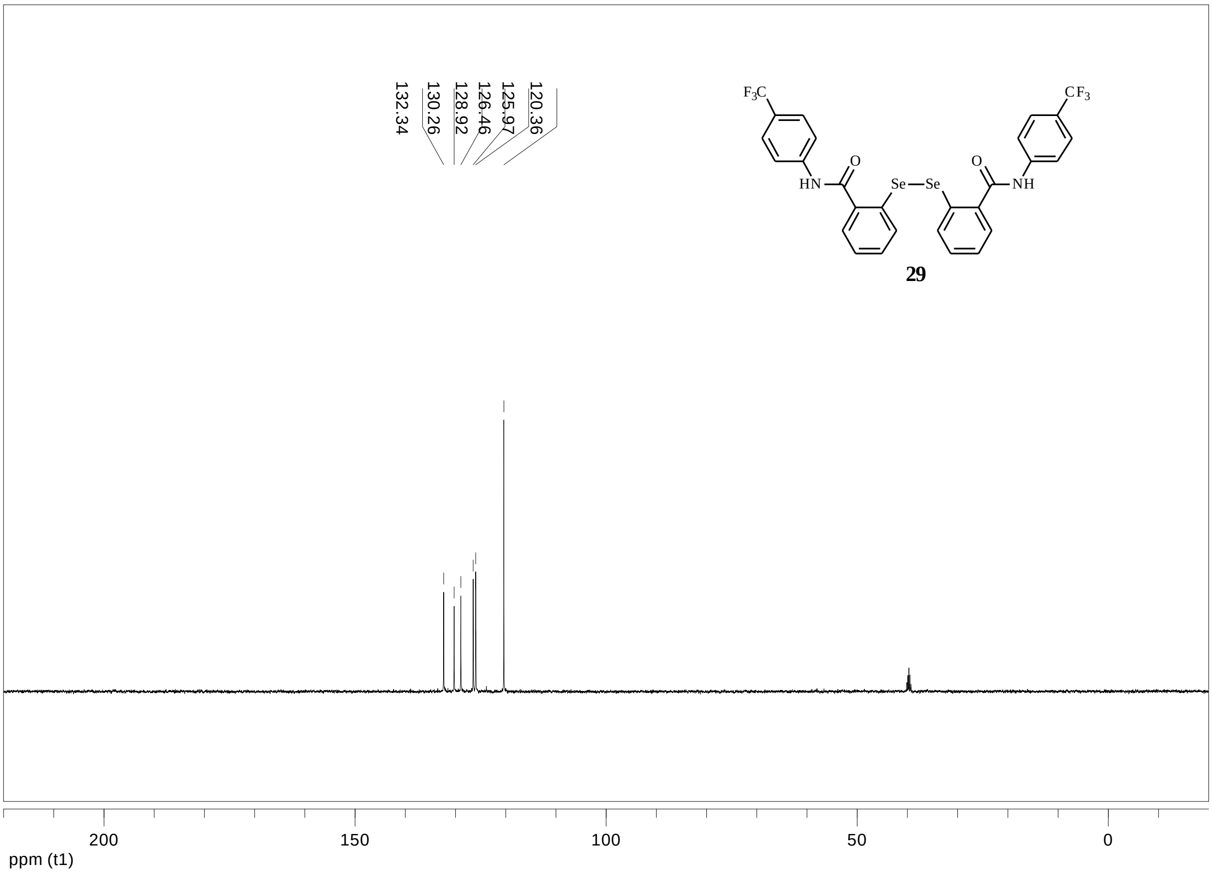

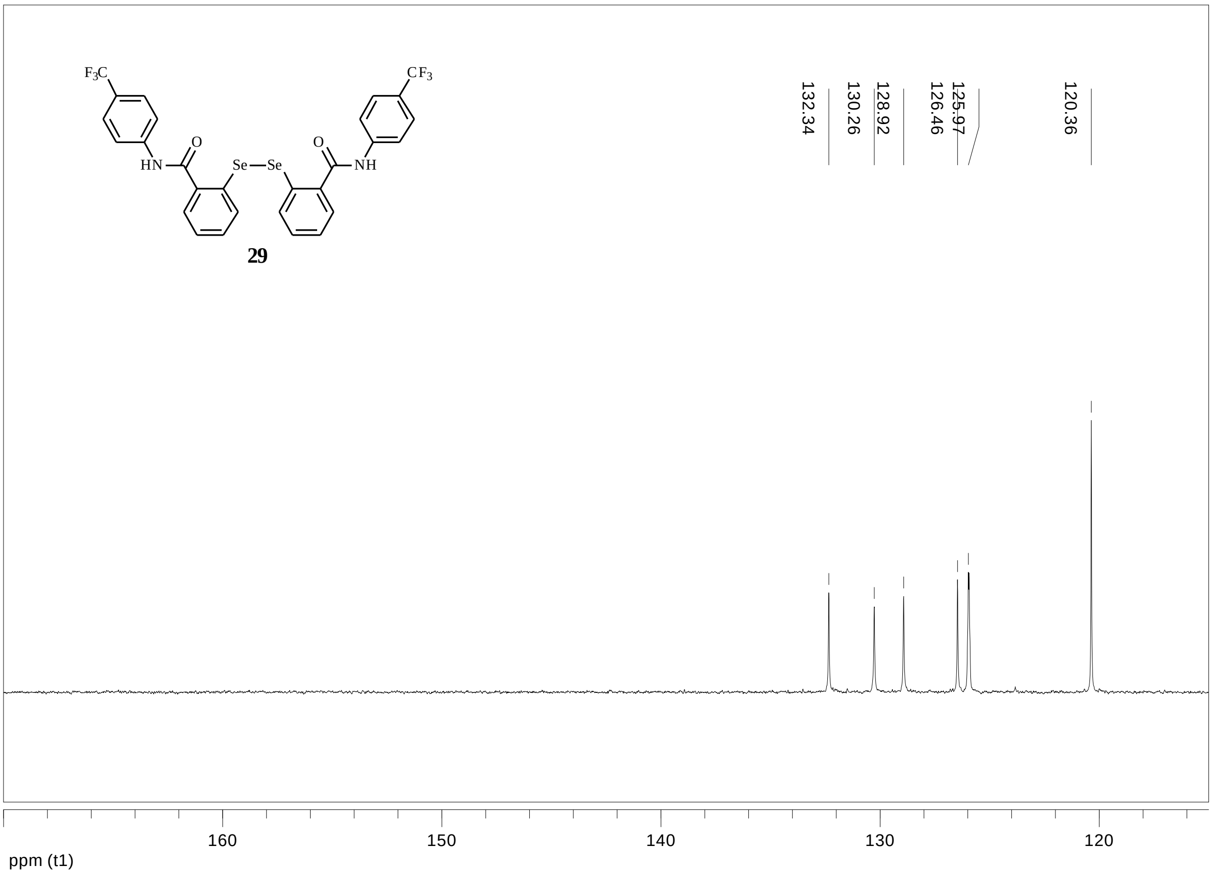

Fig. S140. ^{13}C-NMR (100.5 MHz, DMSO-d_6) dept-135 experiment of compound **29**

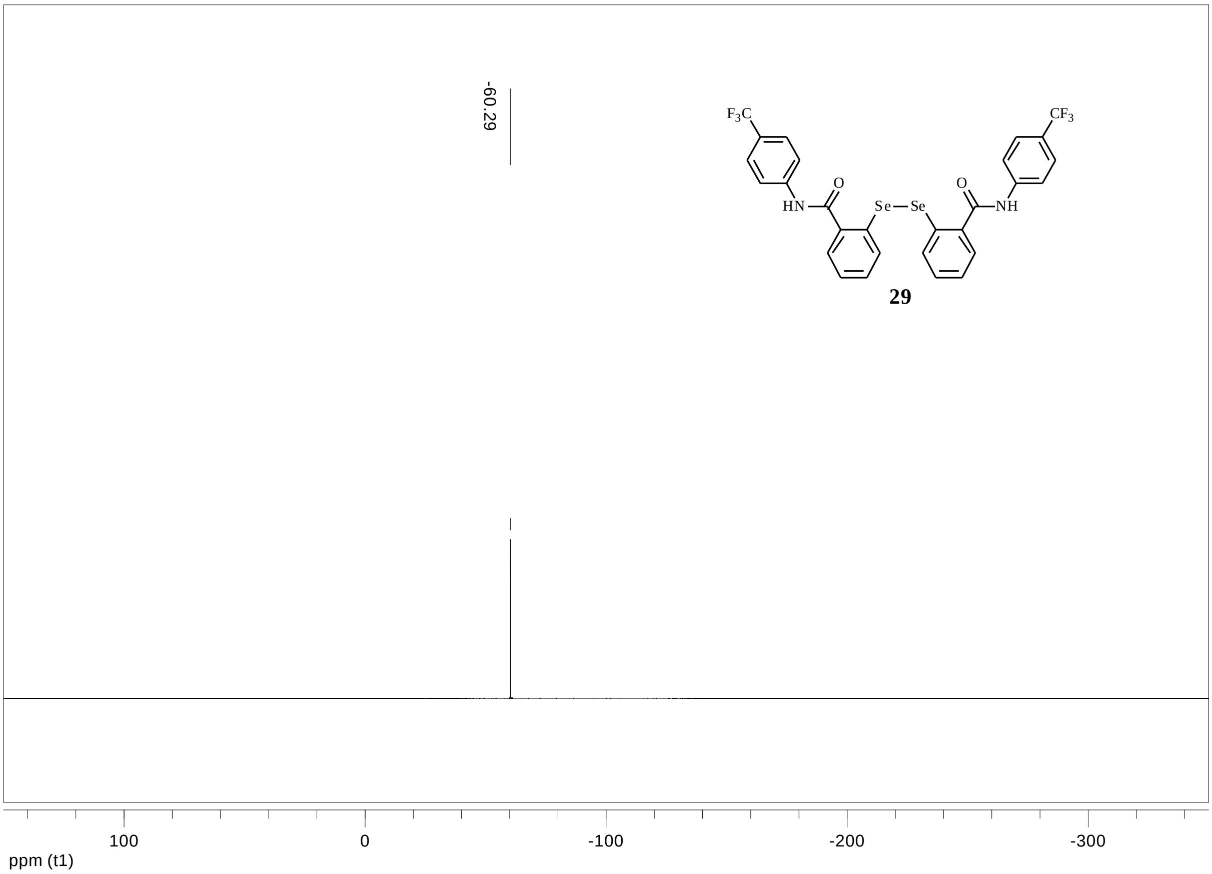

Fig. S141. ^{19}F-NMR (376.2 MHz, DMSO-d_6) spectrum of compound **29**

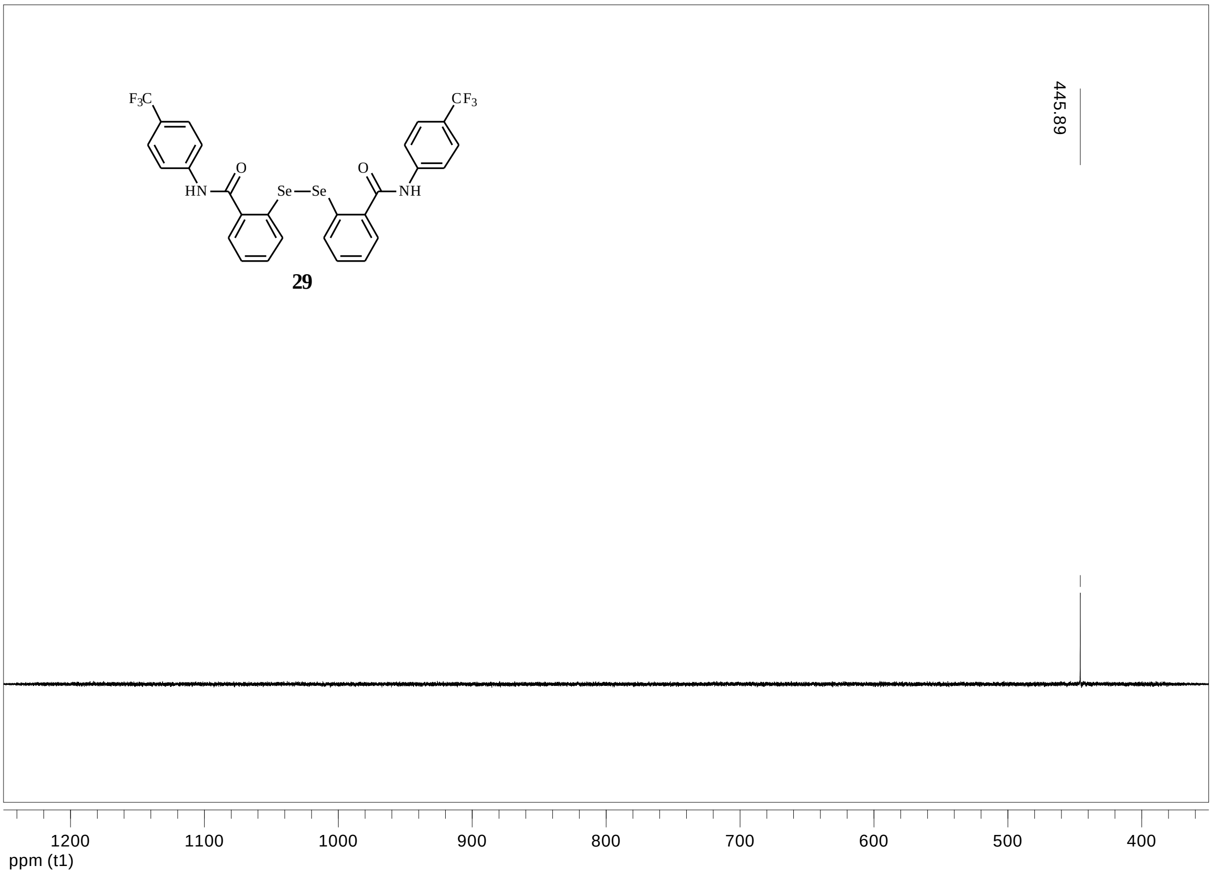

Fig. S142. ^{77}Se-NMR (76.24 MHz, DMSO-d_6) spectrum of compound **29**

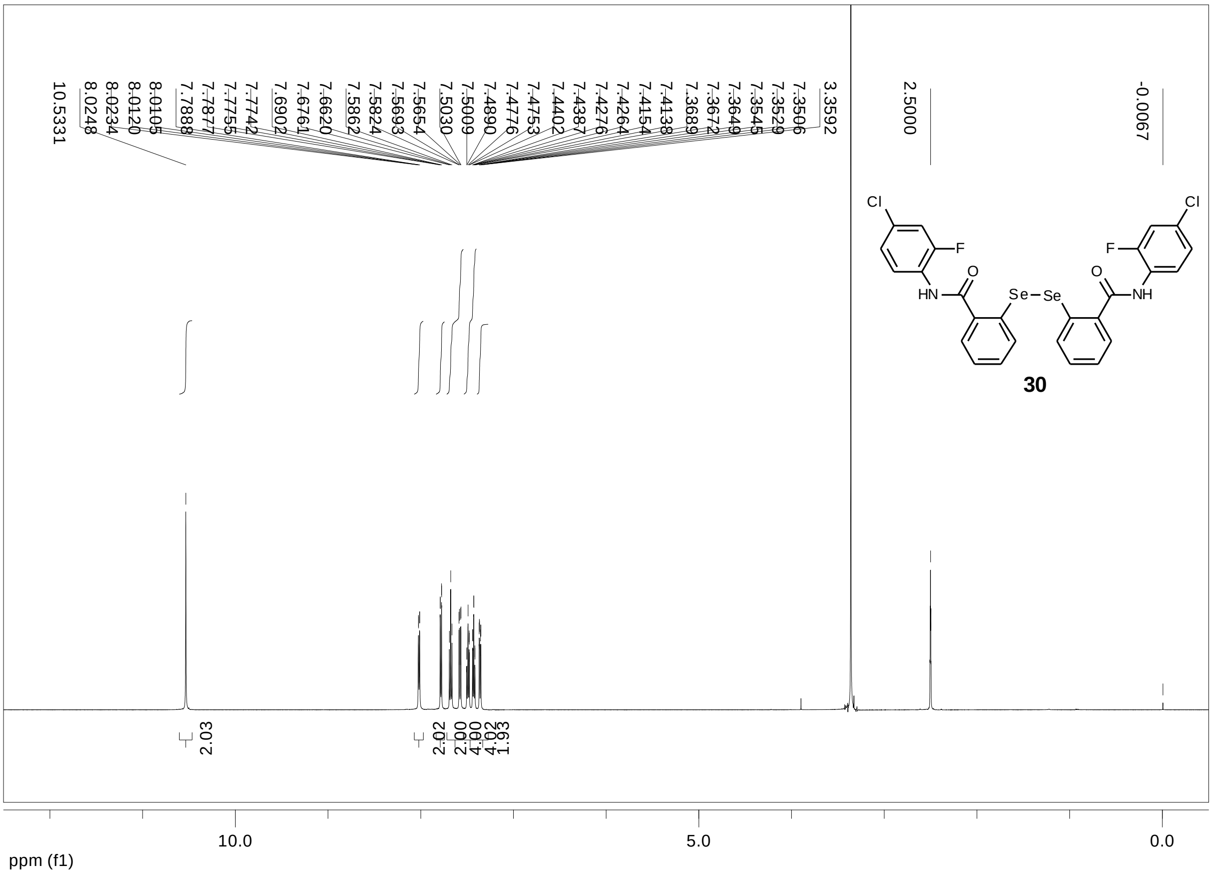

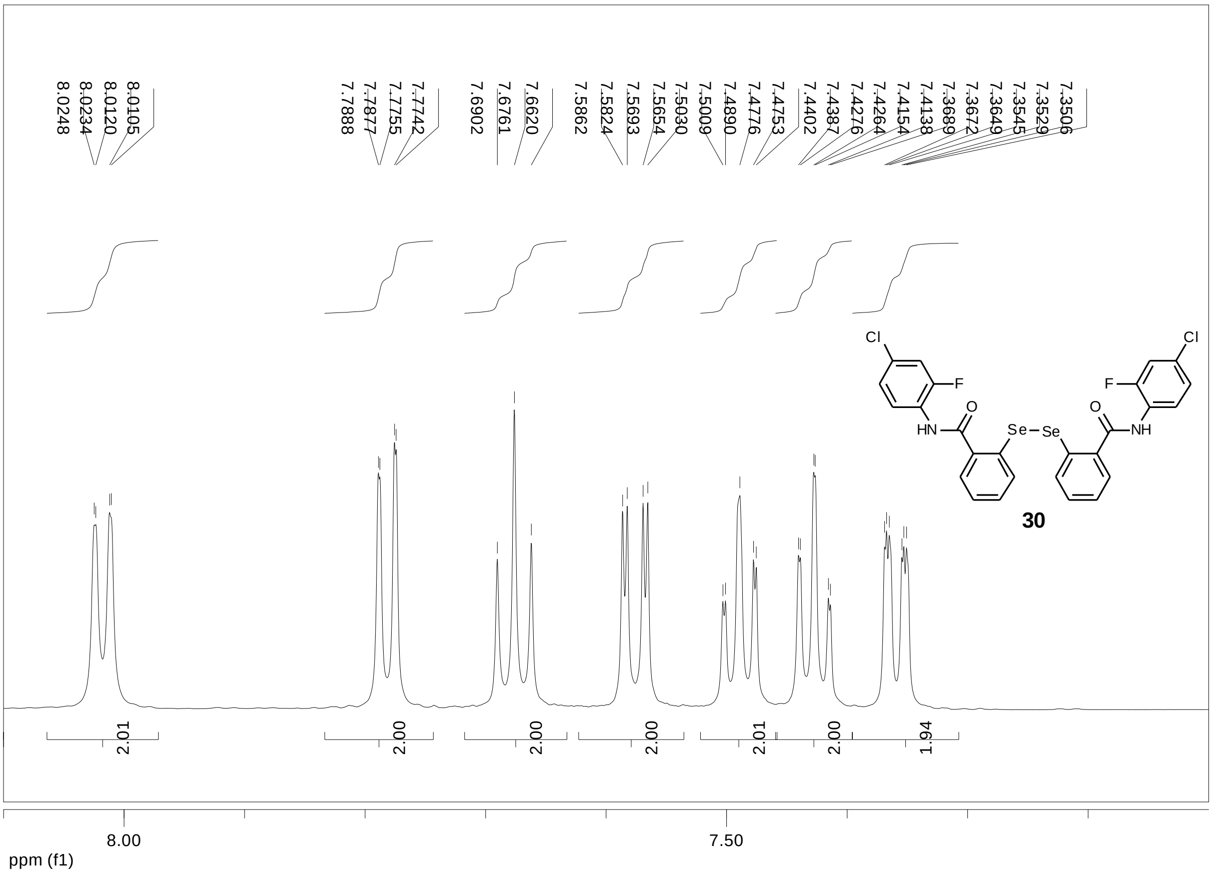

Fig. S143.^{1}H-NMR (600.6 MHz, DMSO-d_6) spectrum of compound **30**

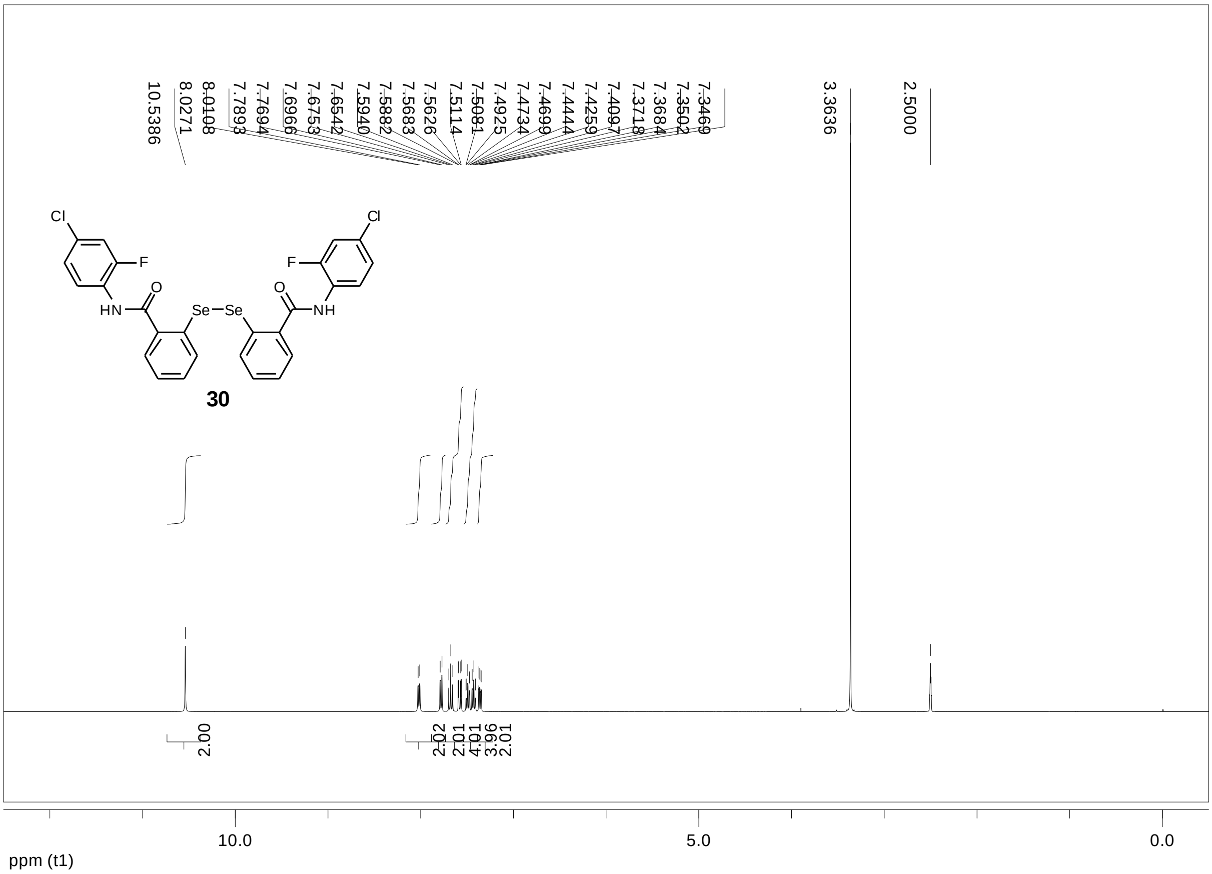

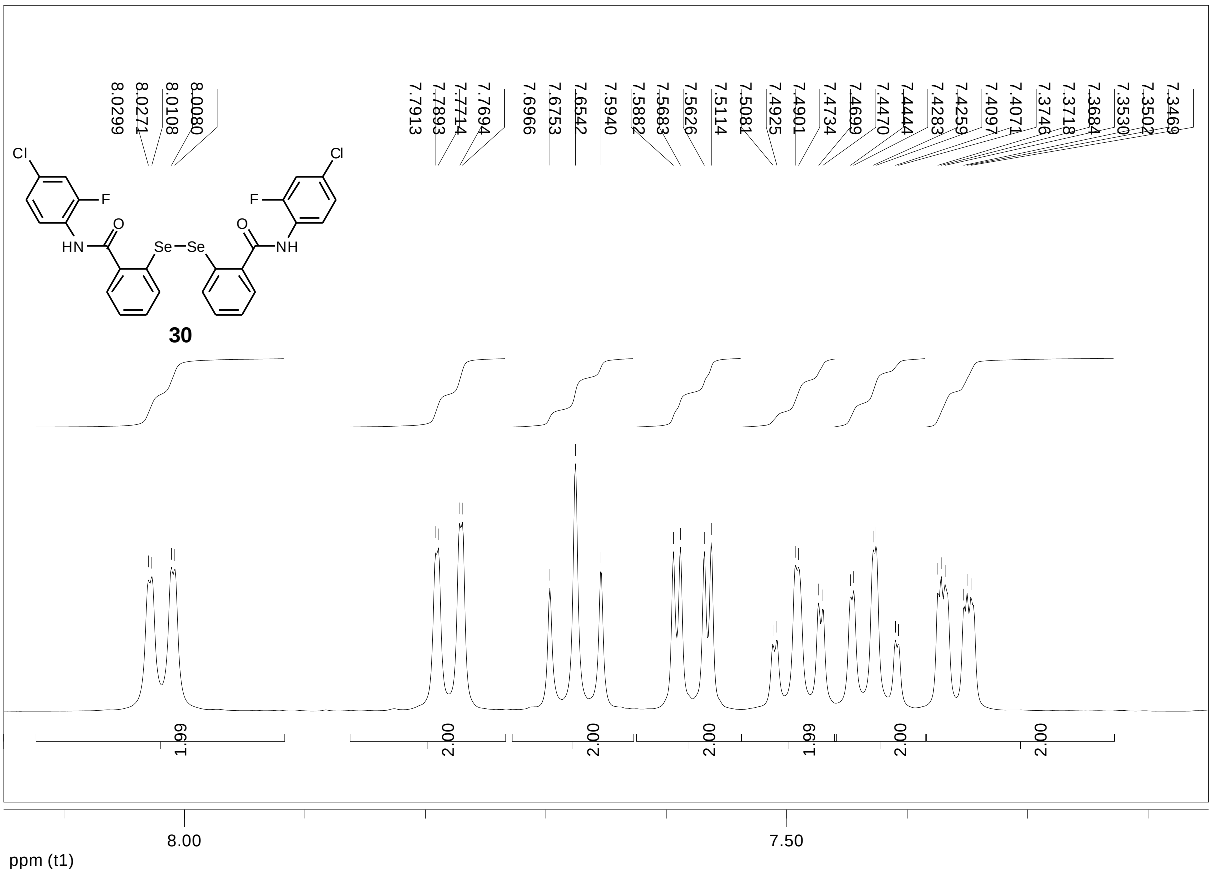

Fig. S144. ^{1}H-NMR (399.8 MHz, DMSO-d_6) spectrum of compound **30**

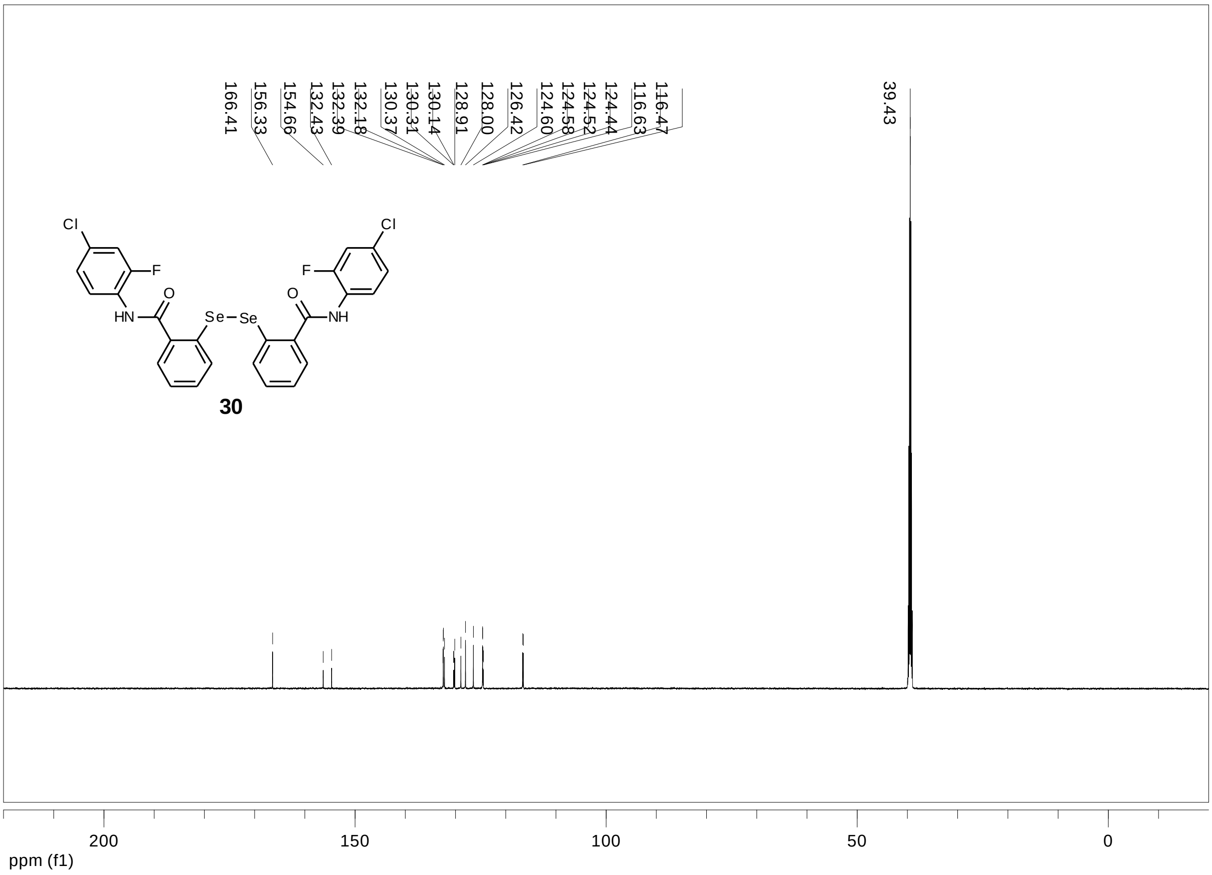

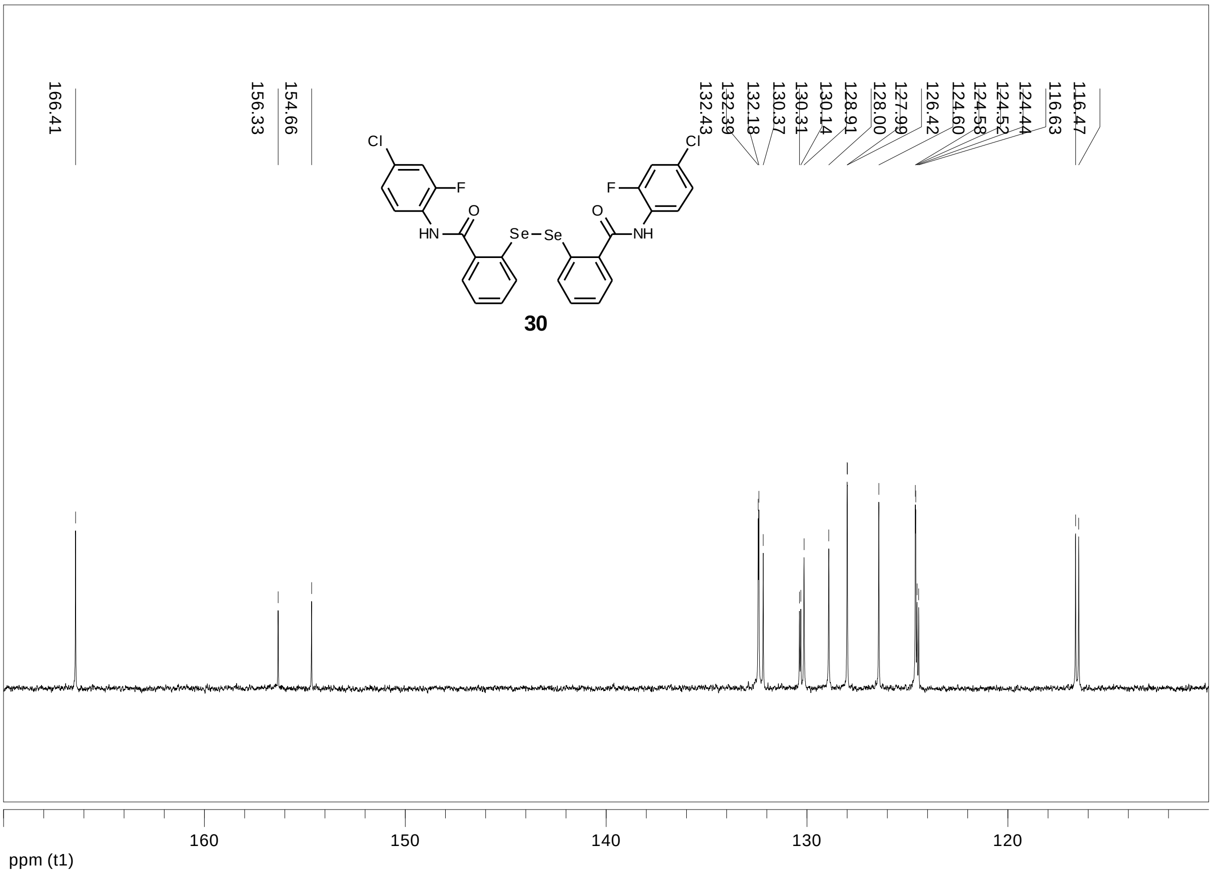

Fig. S145. ^{13}C-NMR (151.0 MHz, DMSO-d_6) spectrum of compound **30**

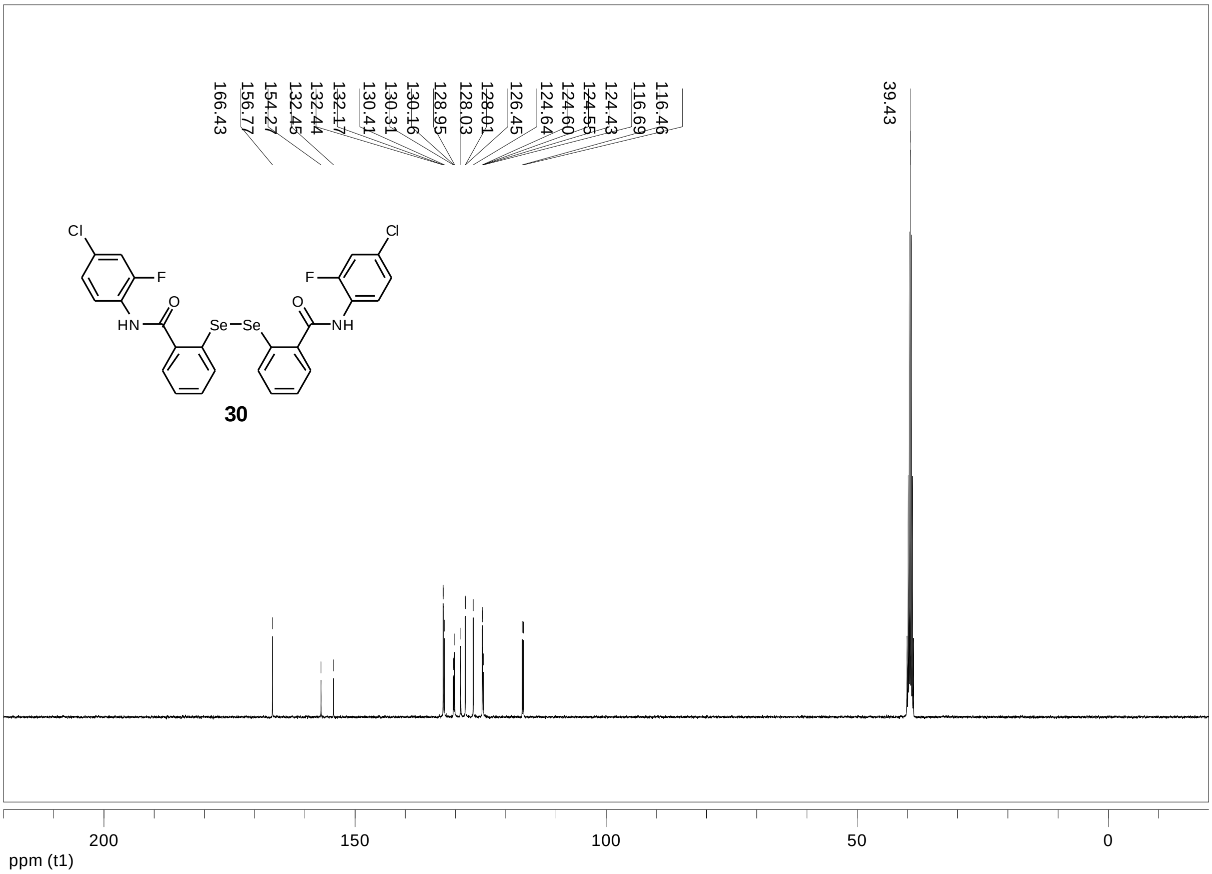
166.43
156.77
154.27
132.45
132.44
132.17
130.41
130.31
130.16
128.95
128.03
128.01
126.45
124.64
124.60
124.55
124.43
116.69
116.46
39.43
Cl
F
O
HN
Se
Se
O
NH
F
Cl
30
200
150
100
50
0
ppm (t1)

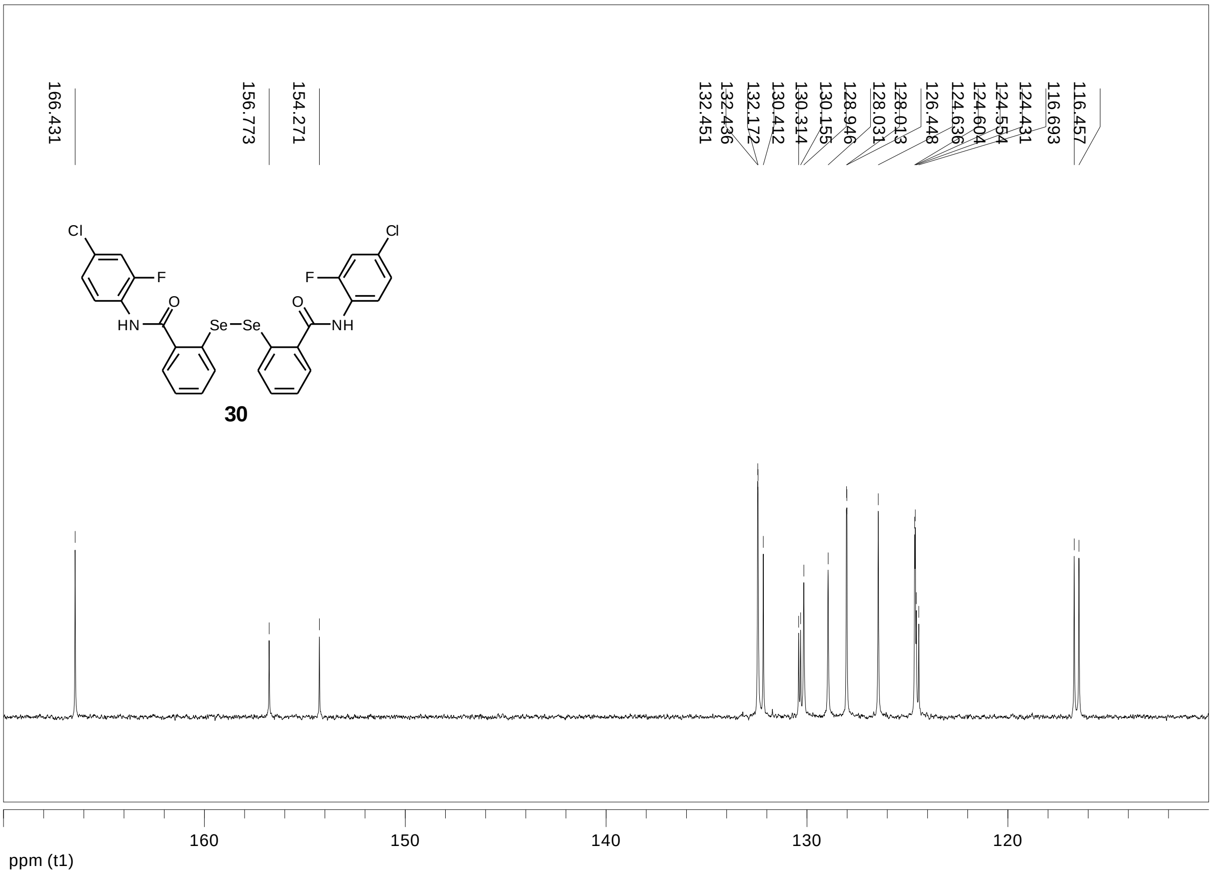
166.431
156.773
154.271
132.451
132.436
132.172
130.412
130.314
130.155
128.946
128.031
128.013
126.448
124.636
124.604
124.554
124.431
116.693
116.457
Cl
F
O
HN
Se
Se
O
NH
F
Cl
30
160
150
140
130
120
ppm (t1)

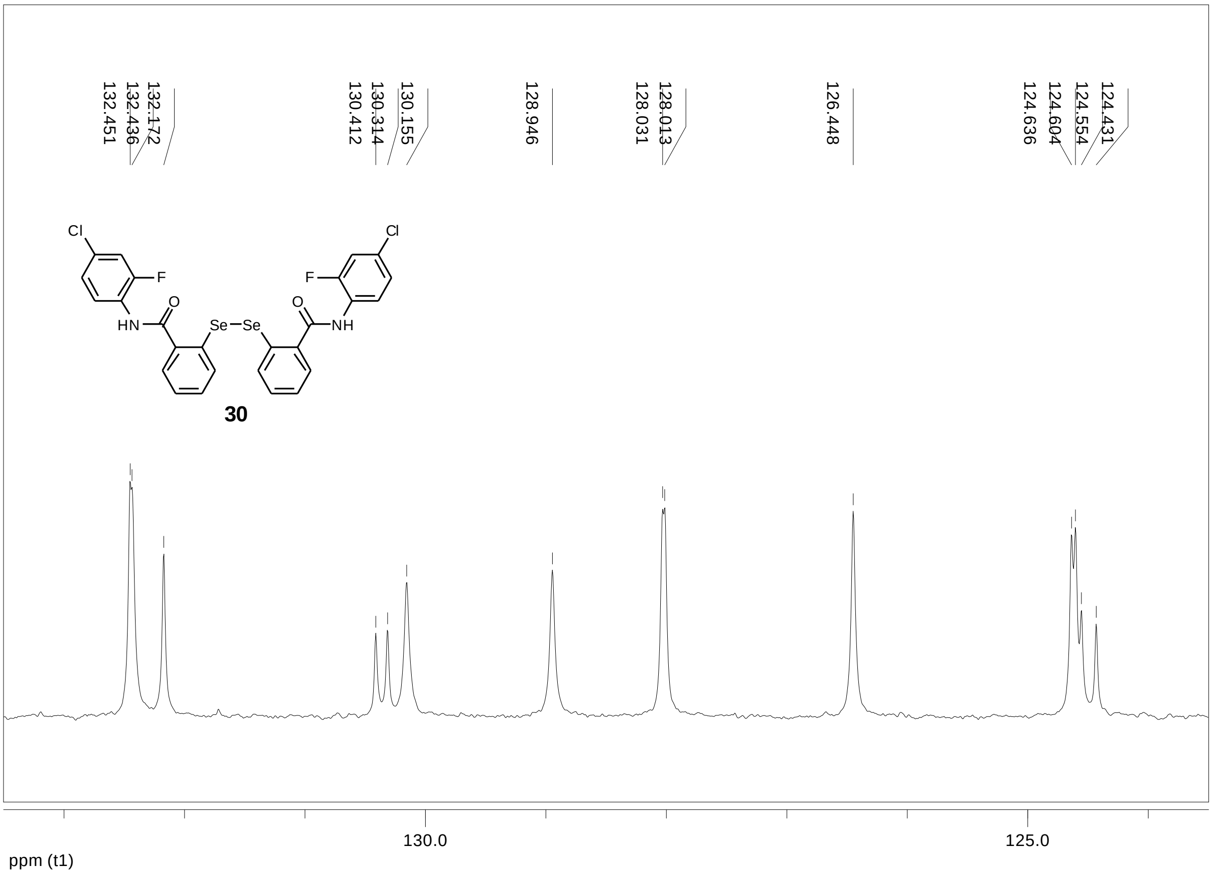

Fig. S146. ^{13}C-NMR (100.5 MHz, DMSO-d_6) spectrum of compound **30**

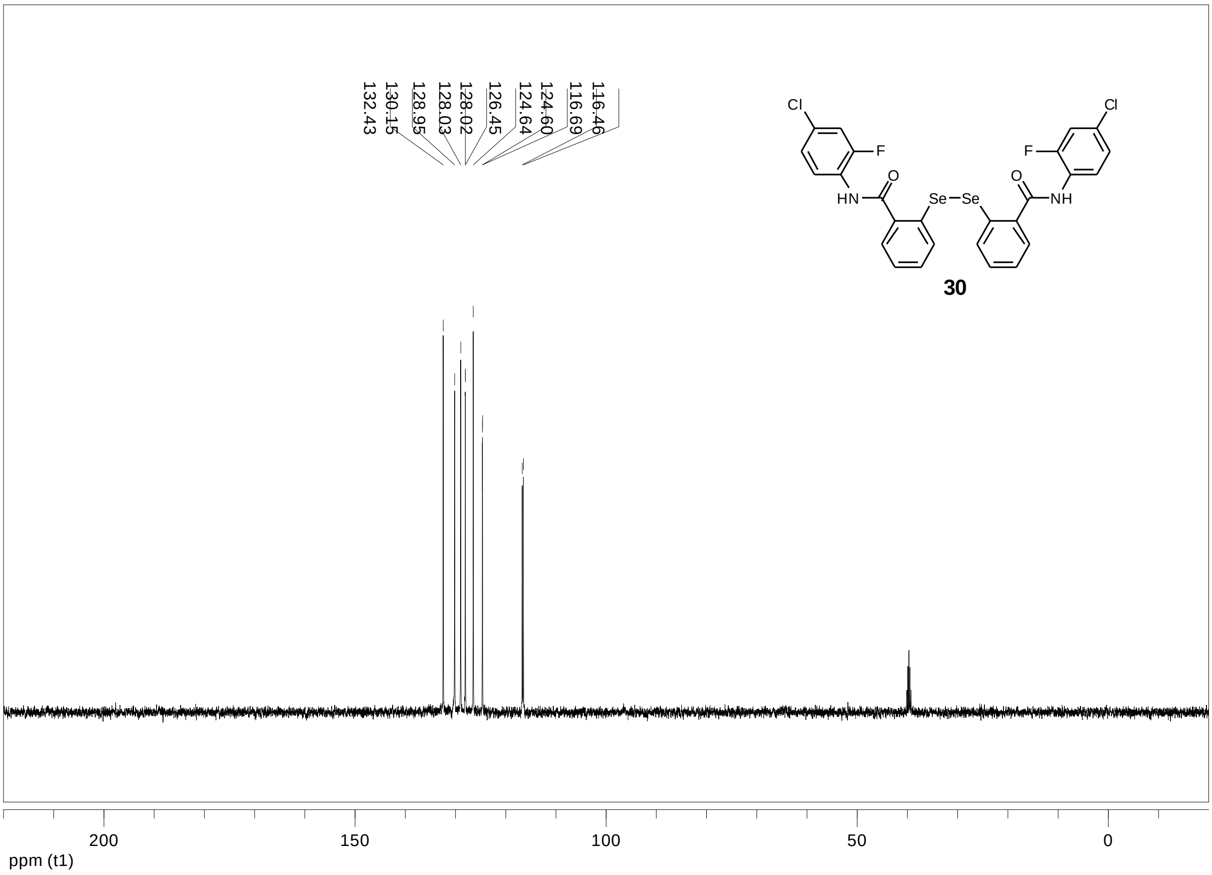
132.43
130.15
128.95
128.03
128.02
126.45
124.64
124.60
116.69
116.46
Cl
F
O
HN
Se
Se
NH
30
200
150
100
50
0
ppm (t1)

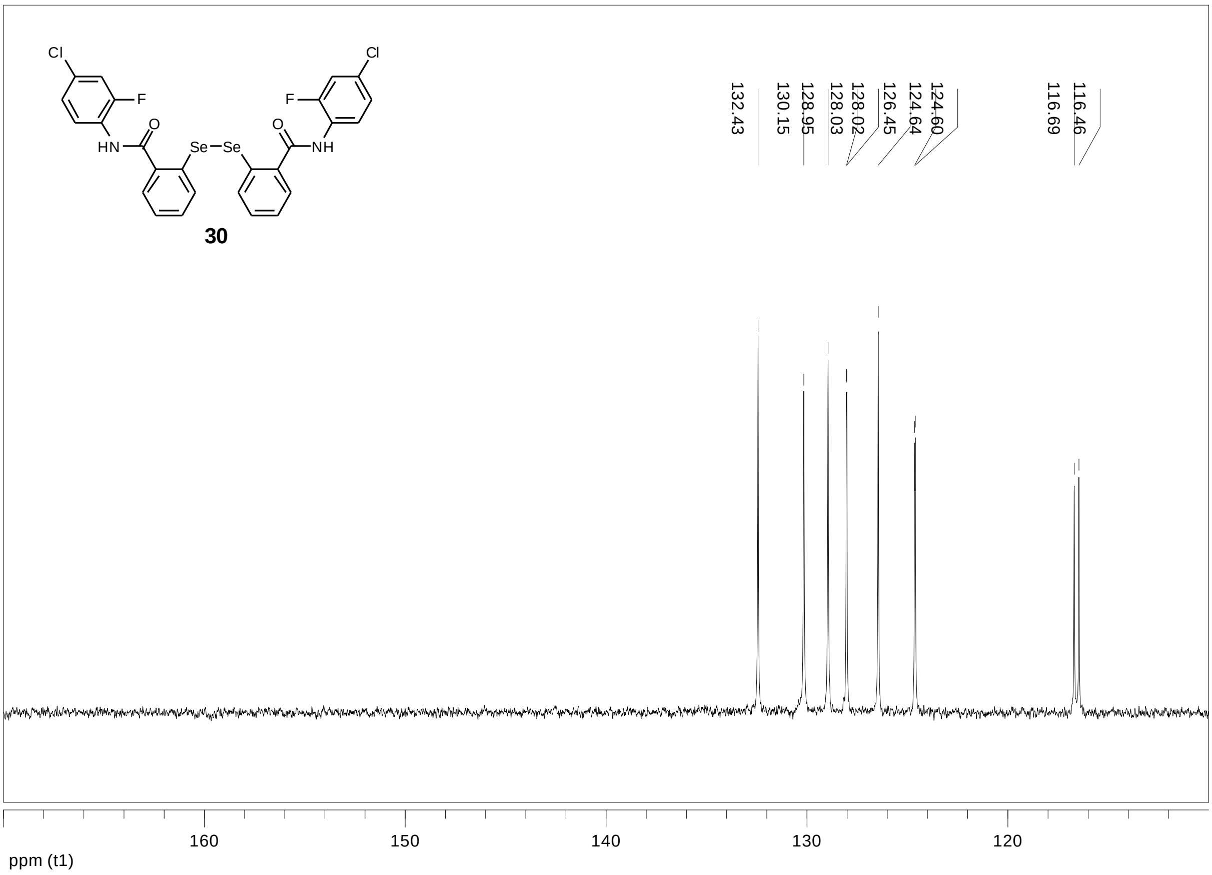
Cl
F
O
HN
Se
Se
NH
30
132.43
130.15
128.95
128.03
128.02
126.45
124.64
124.60
116.69
116.46
160
150
140
130
120
ppm (t1)

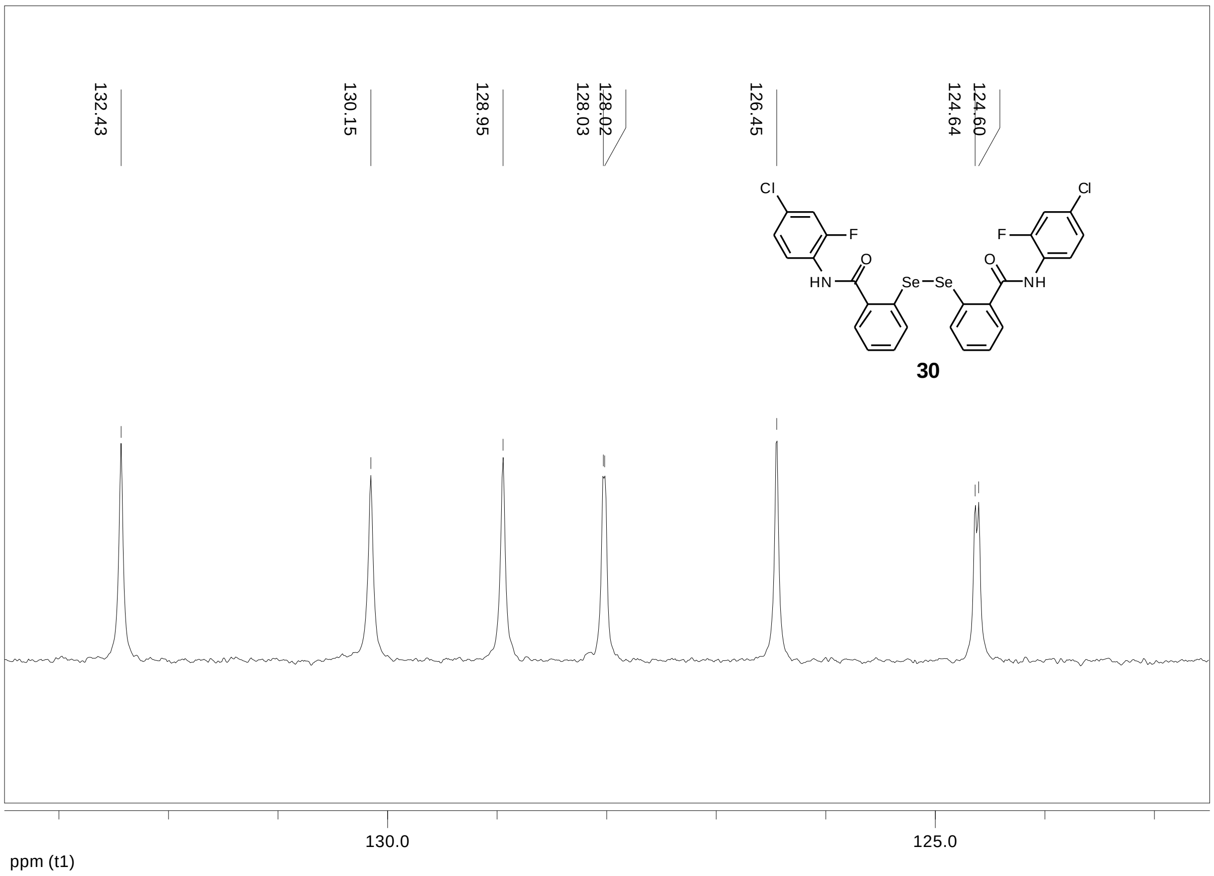

Fig. S147. ^{13}C-NMR (100.5 MHz, DMSO-d_6) dept-135 experiment of compound **30**

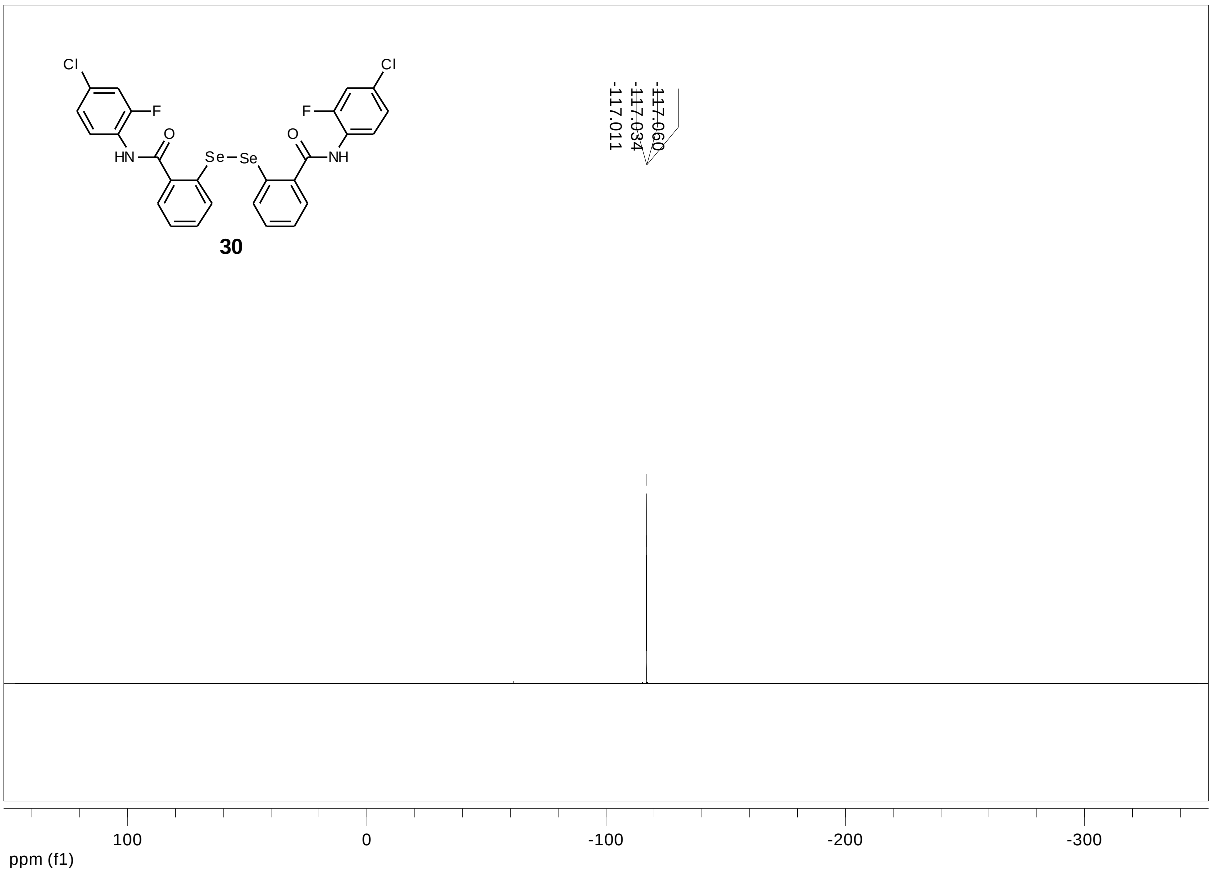

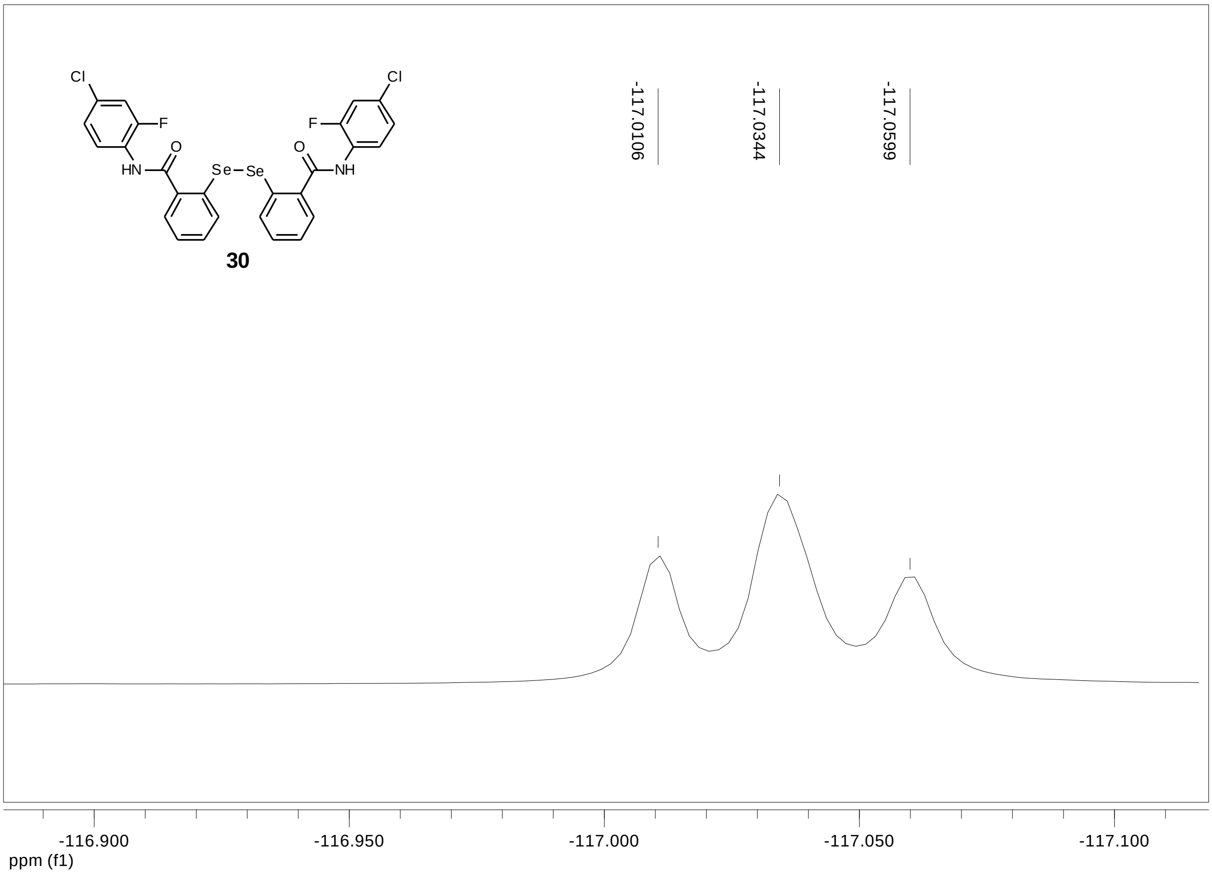

Fig. S148. ^{19}F-NMR (376.2 MHz, DMSO-d_6) spectrum of compound **30**

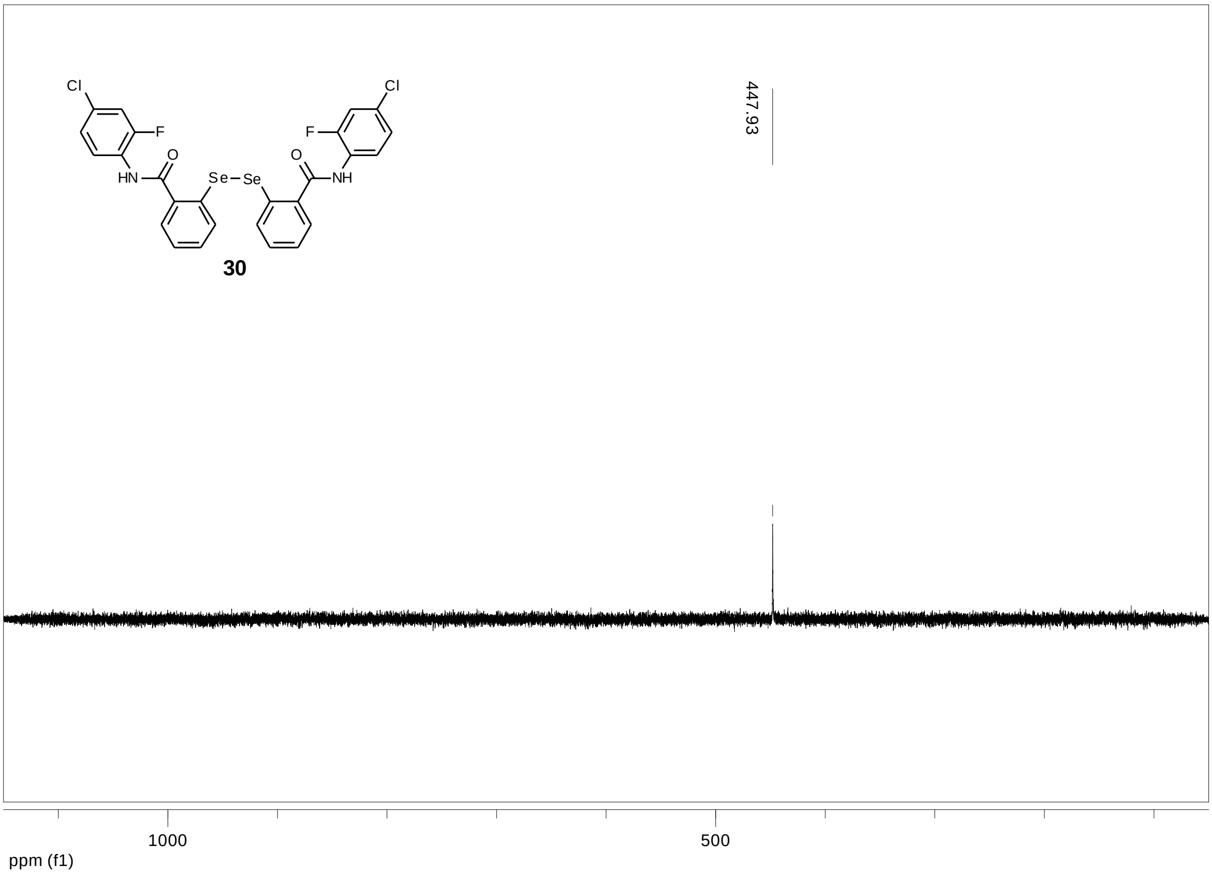

Fig. S149. ^{77}Se-NMR (76.24 MHz, DMSO-d_6) spectrum of compound **30**

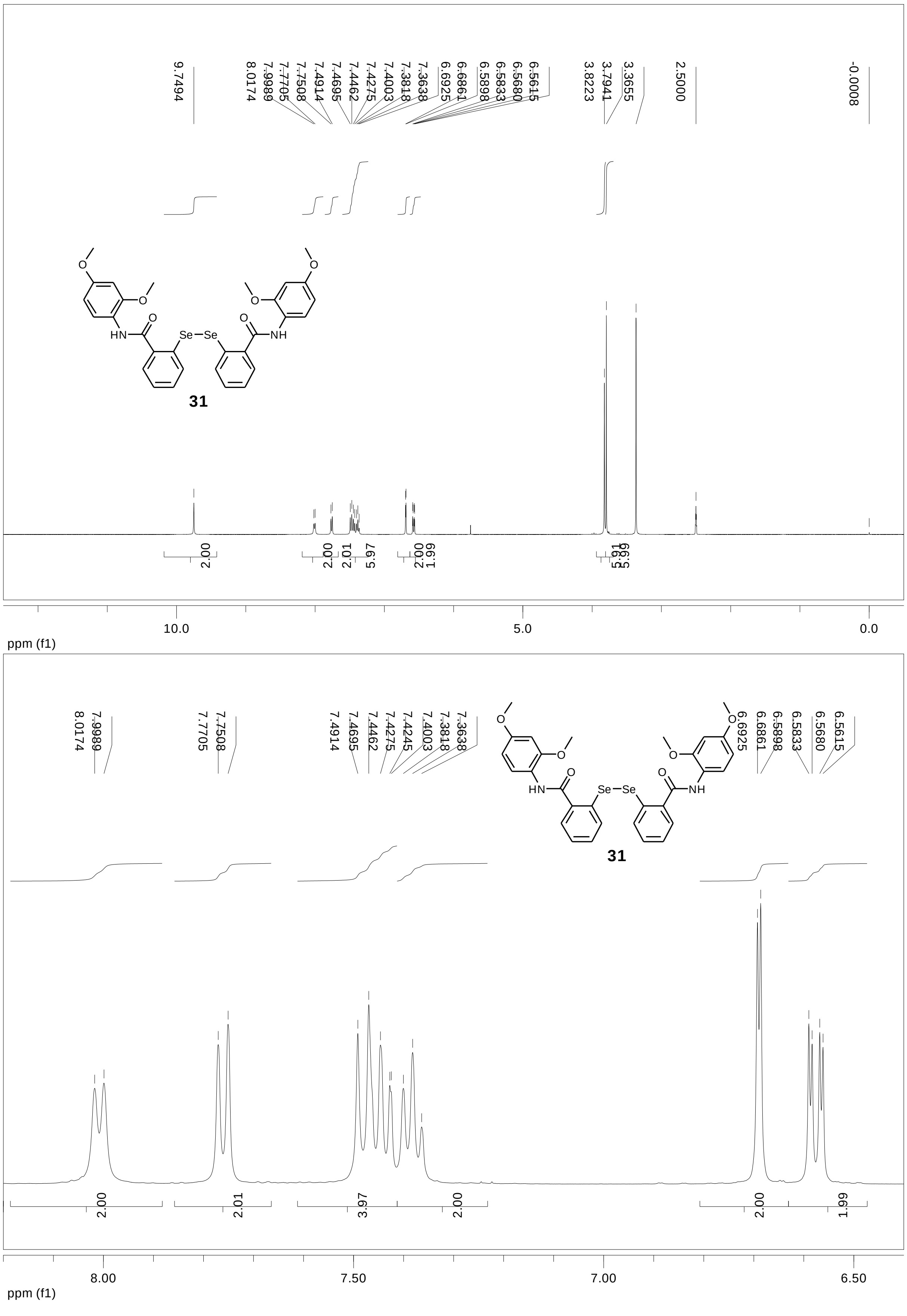

Fig. S150. ^{1}H-NMR (399.8 MHz, DMSO-d_6) spectrum of compound **31**

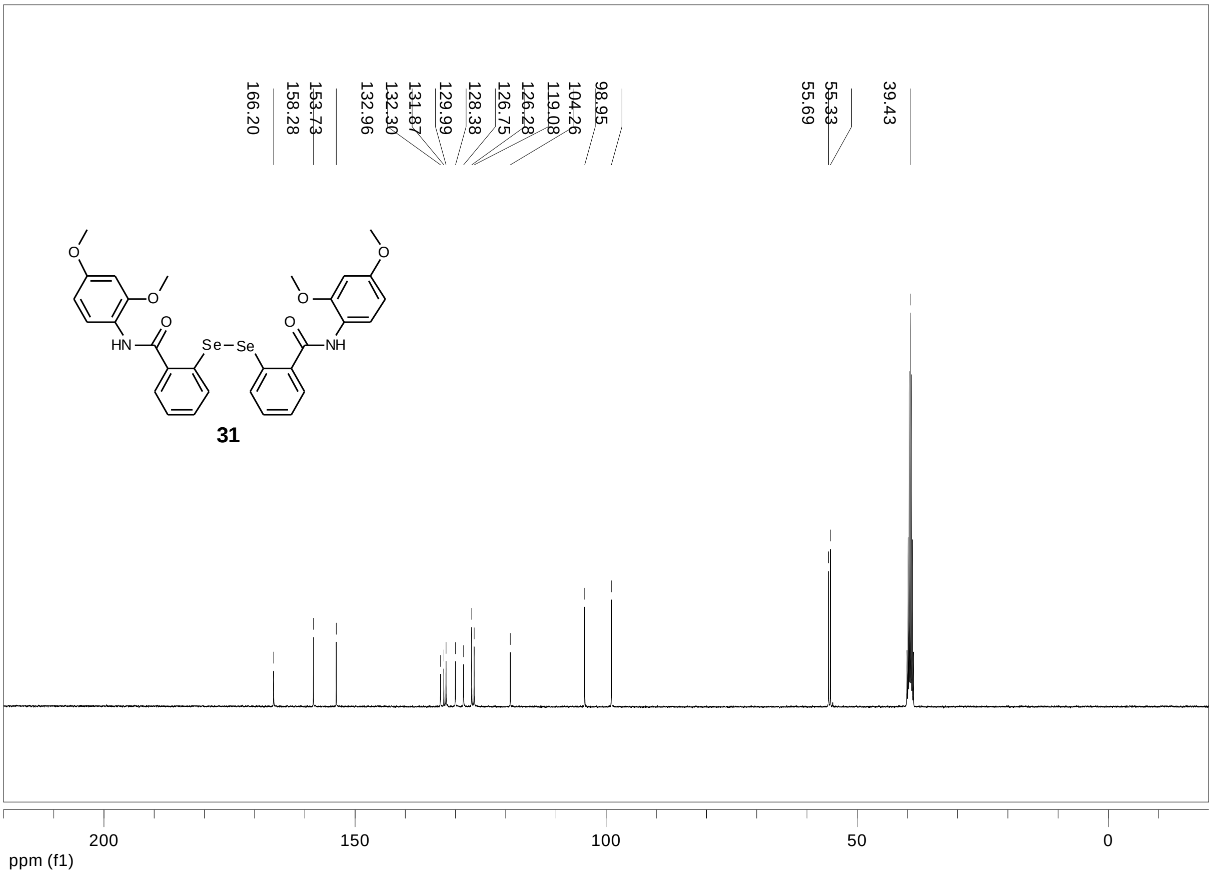

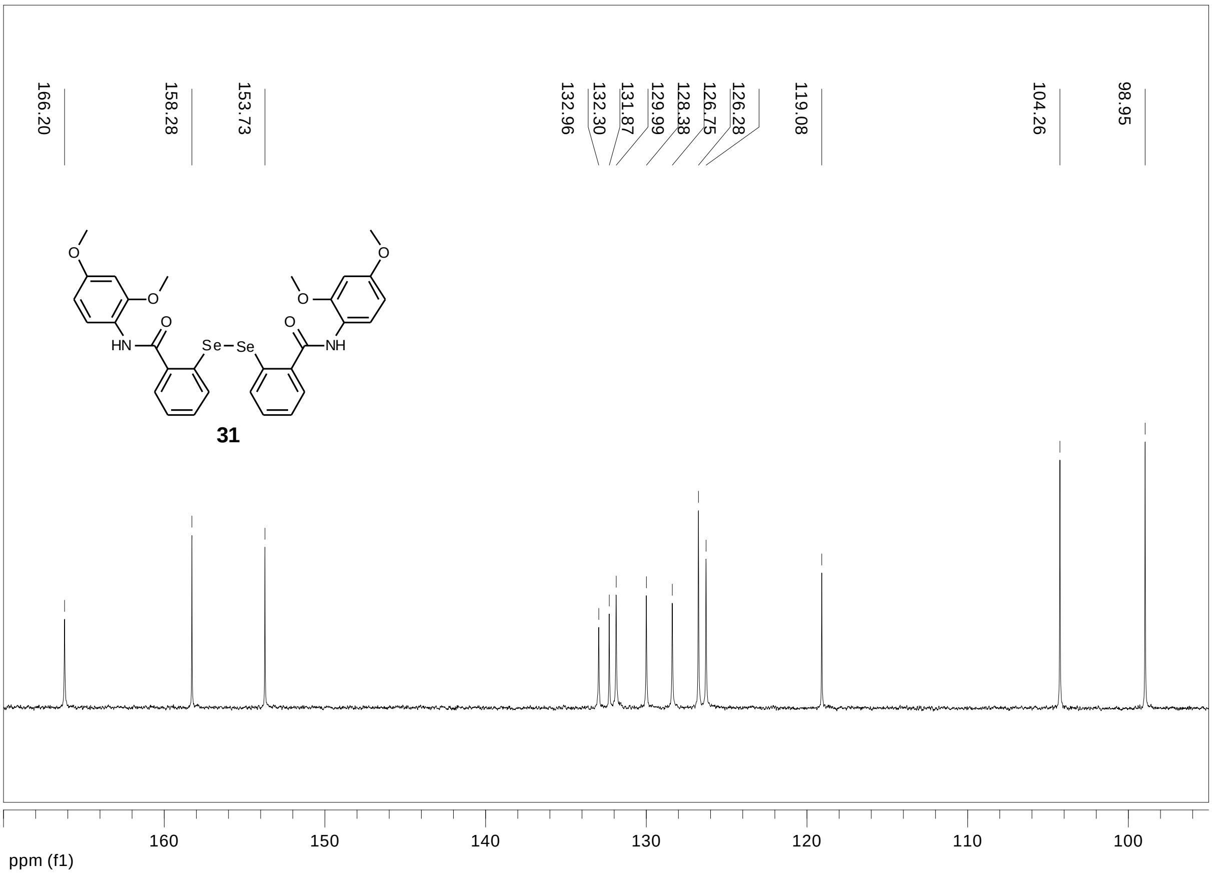

Fig. S151. ^{13}C-NMR (100.5 MHz, DMSO-d_6) spectrum of compound **31**

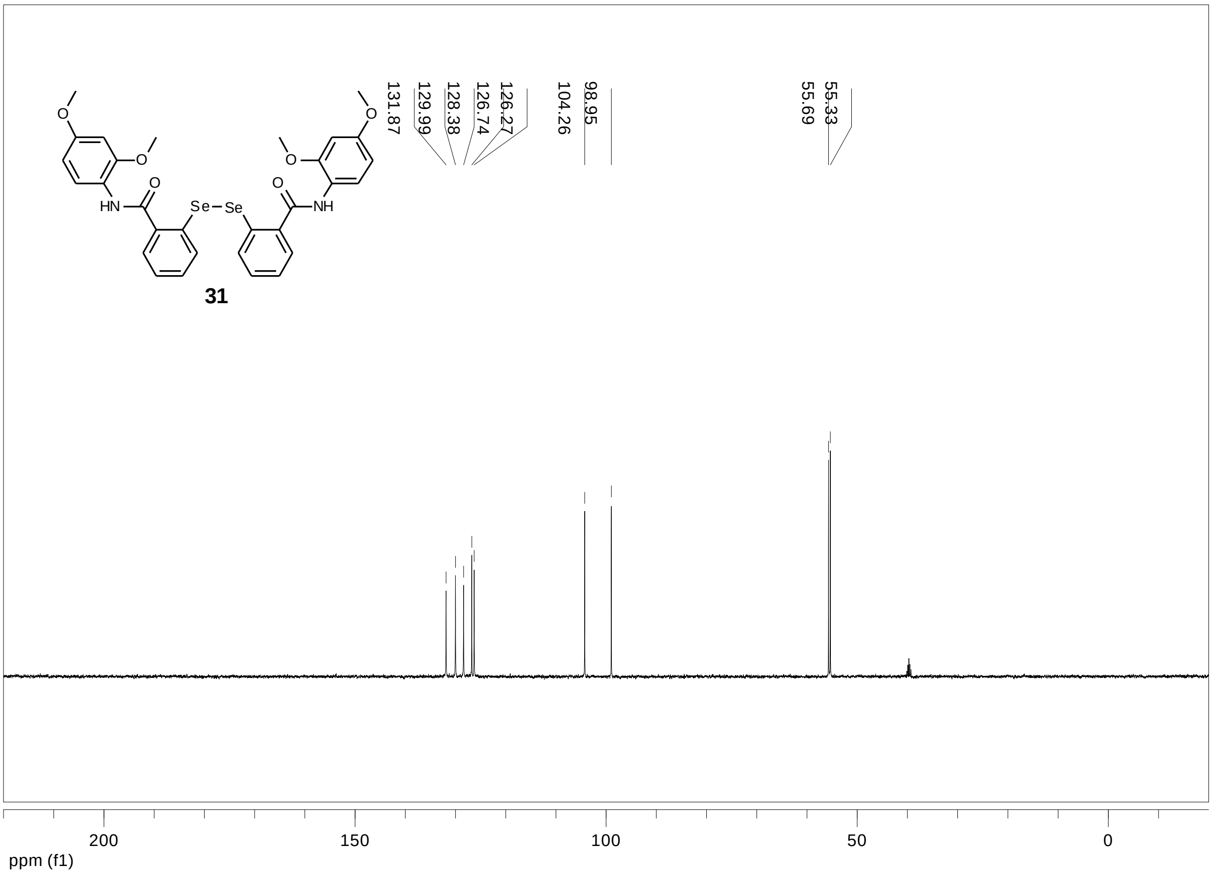

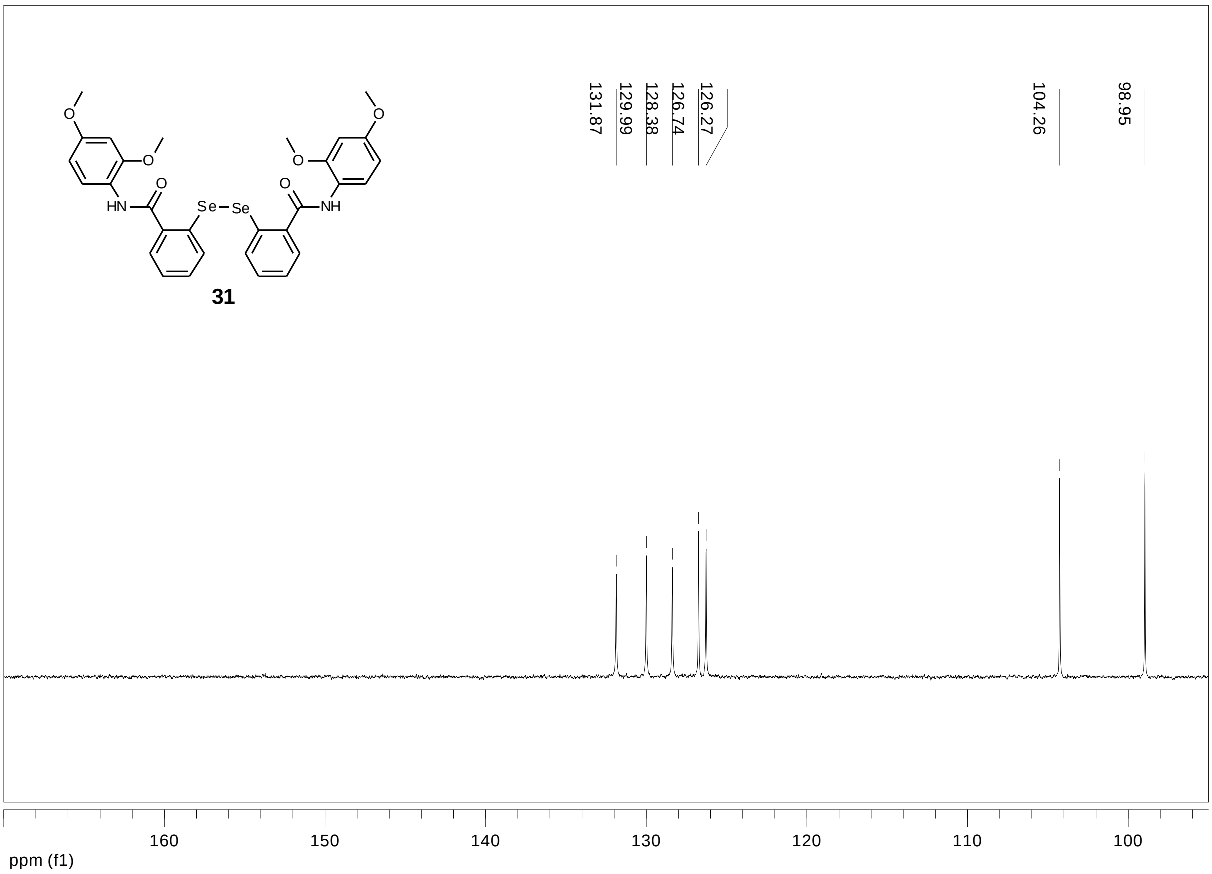

Fig. S152. ^{13}C-NMR (100.5 MHz, DMSO-d_6) dept-135 experiment of compound **31**

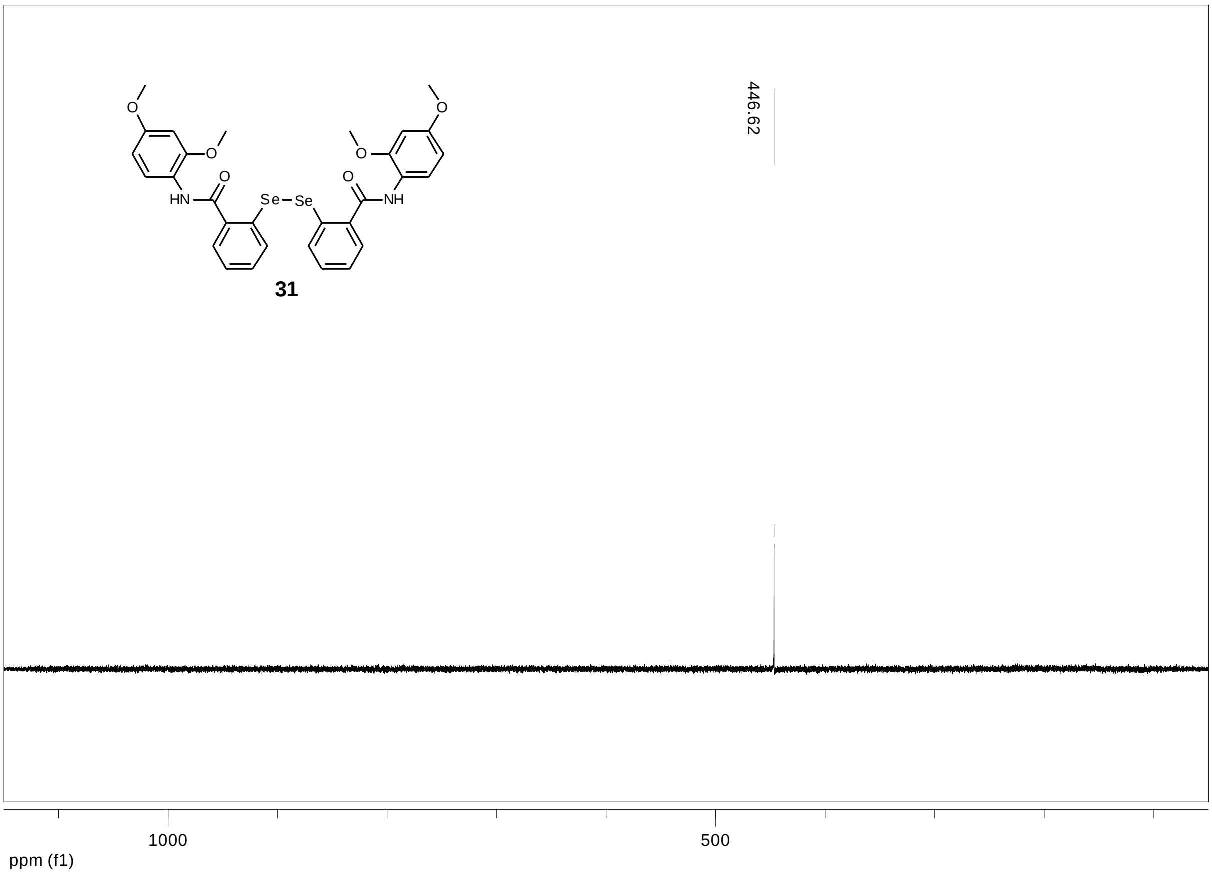

Fig. S153. ^{77}Se-NMR (76.24 MHz, DMSO-d_6) spectrum of compound **31**

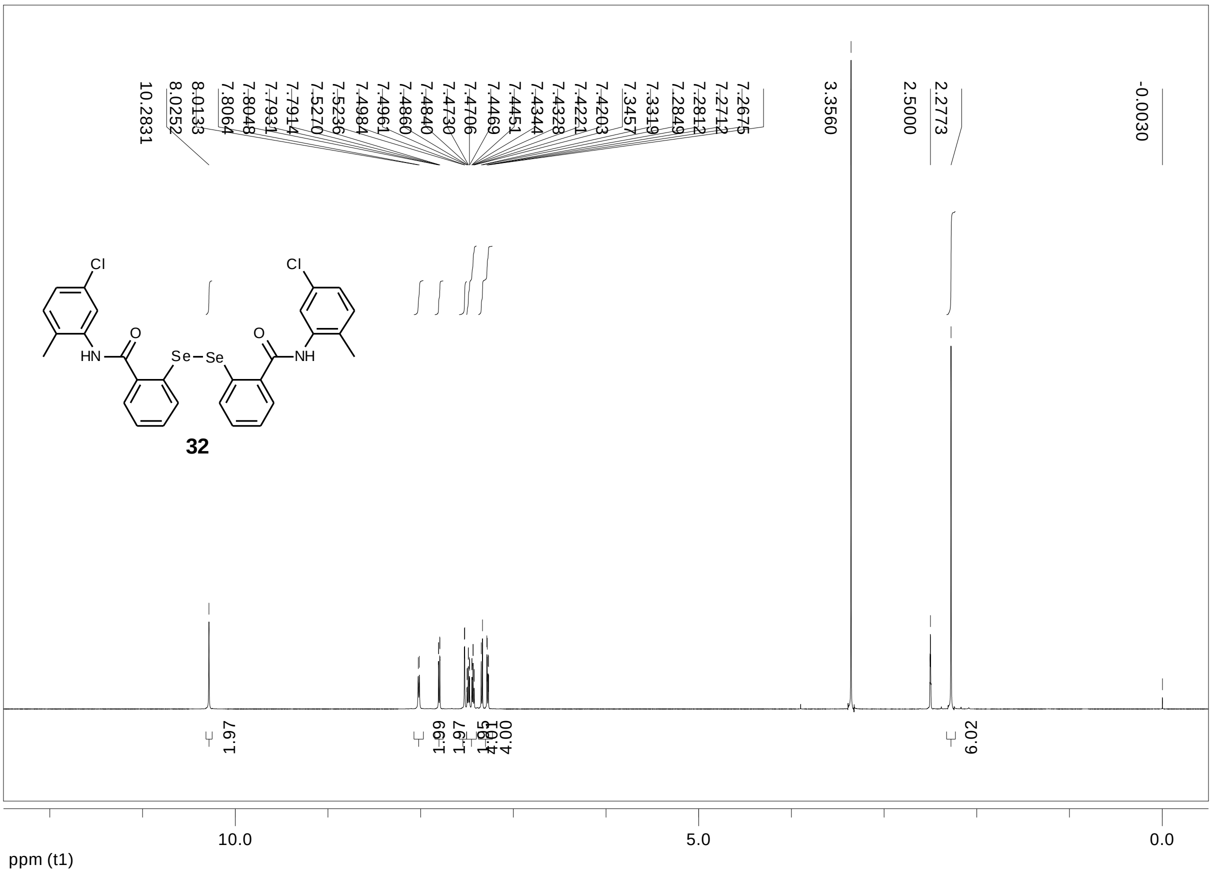

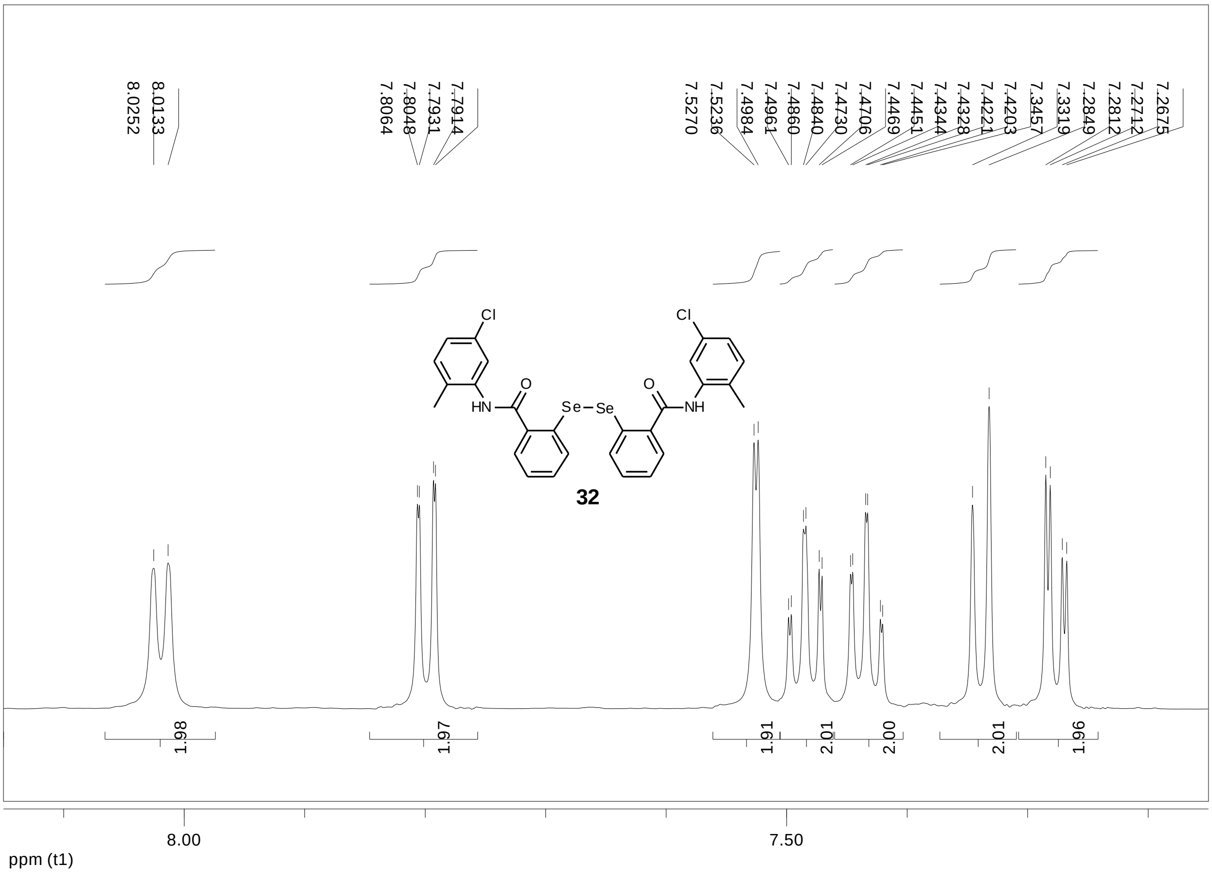

Fig. S154. ^{1}H-NMR (600.6 MHz, DMSO-d_6) spectrum of compound **32**

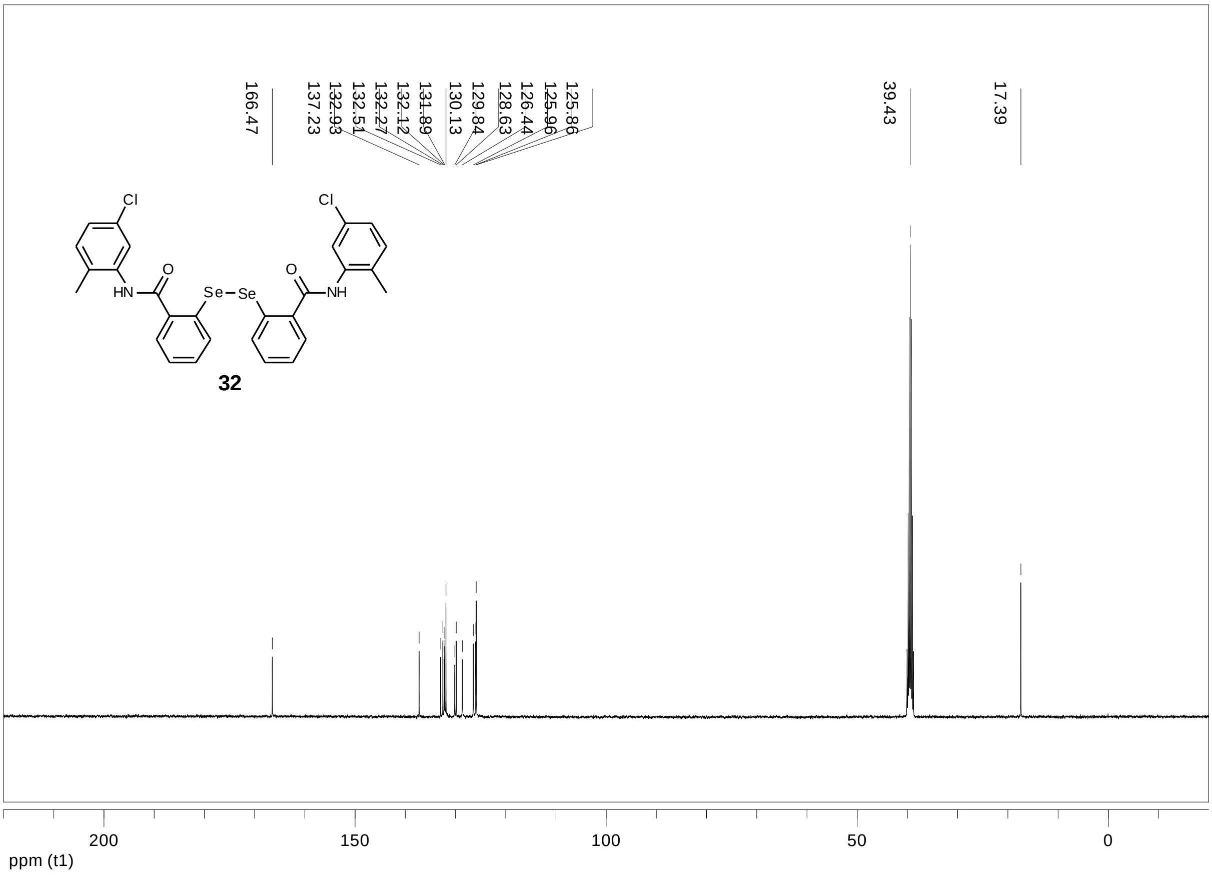

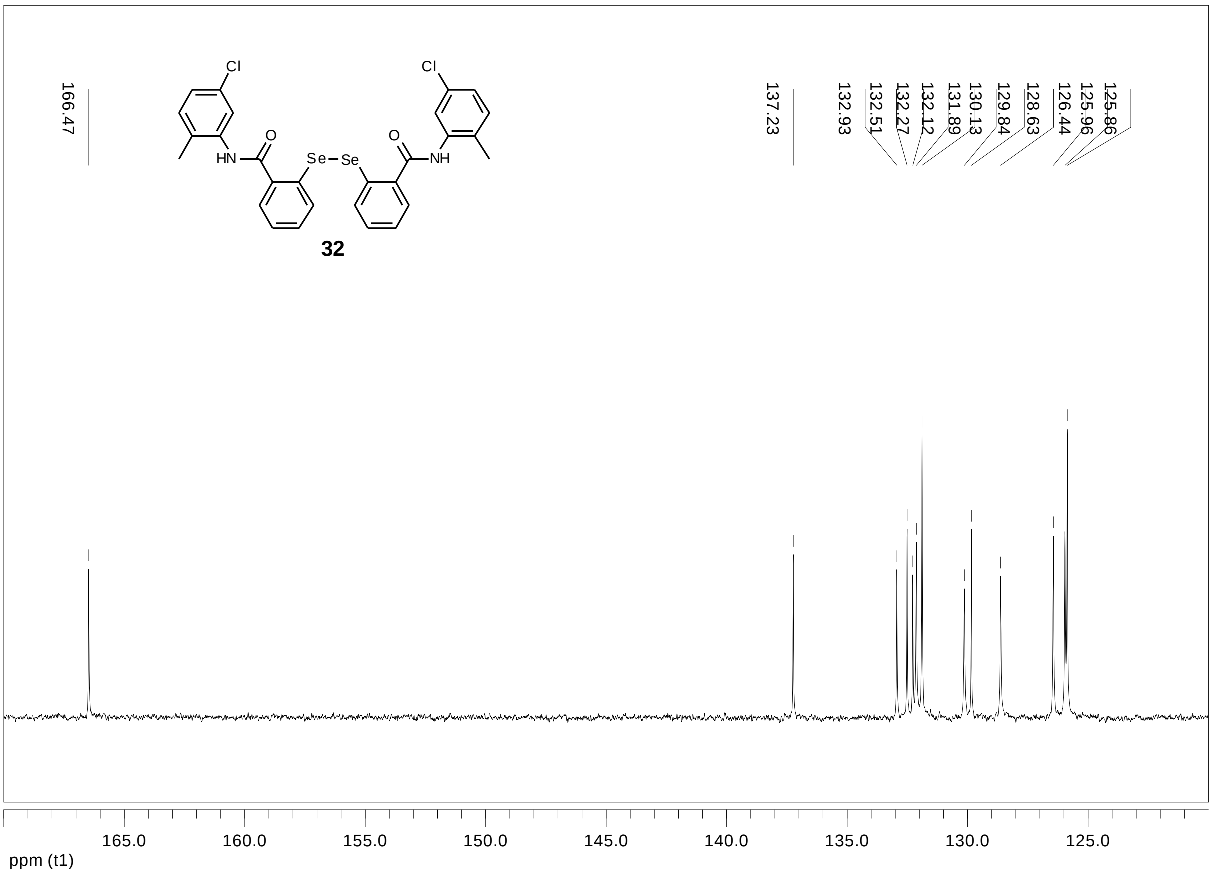

Fig. S155. ^{13}C-NMR (100.5 MHz, DMSO-d_6) spectrum of compound **32**

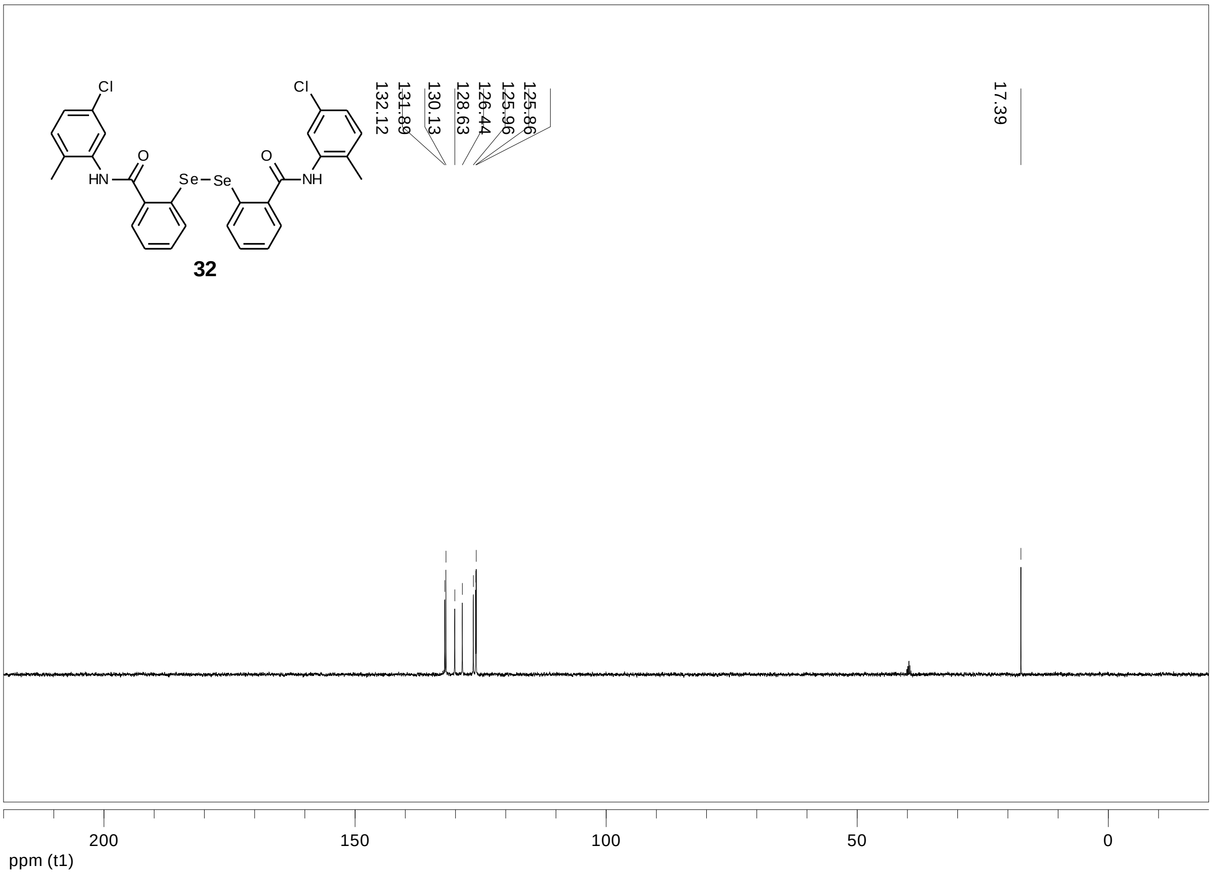

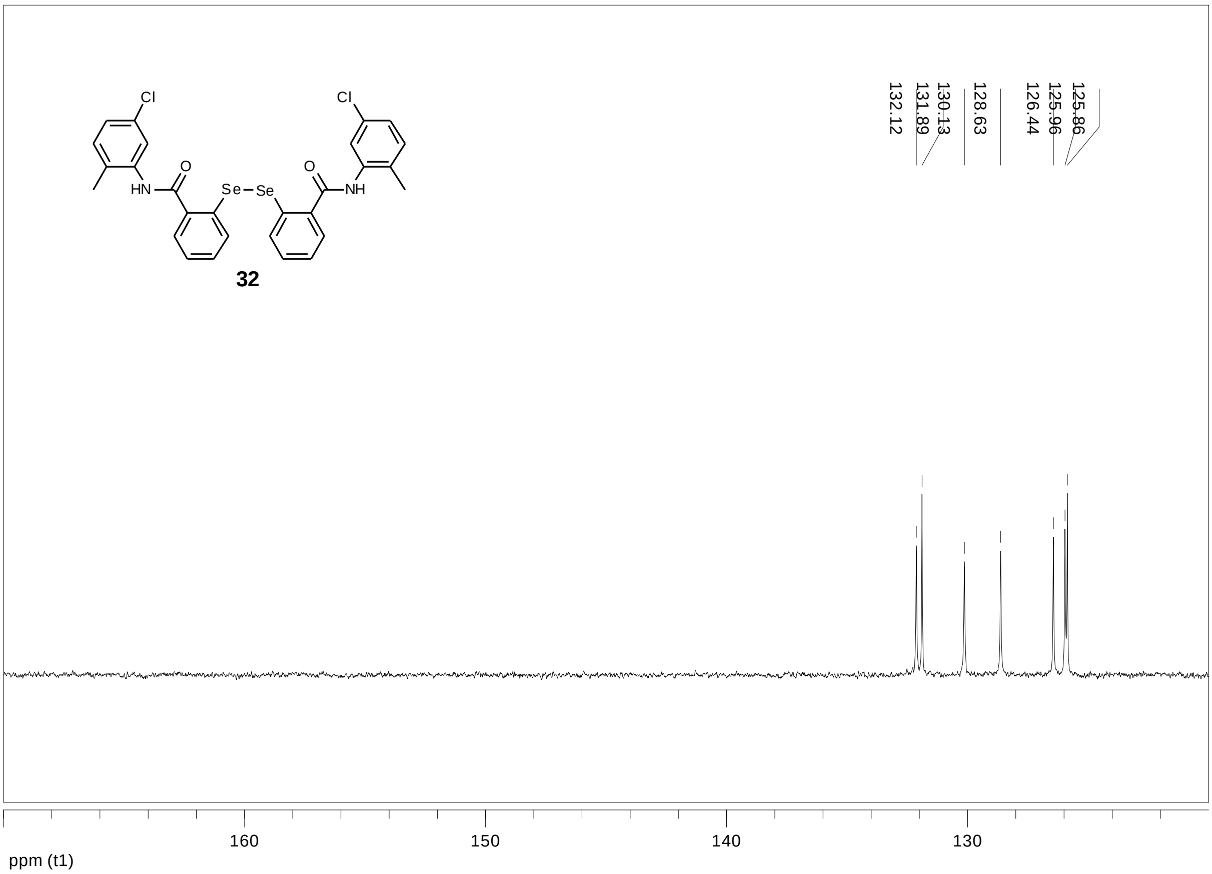

Fig. S156. ^{13}C-NMR (100.5 MHz, DMSO-d_6) dept-135 experiment of compound **32**

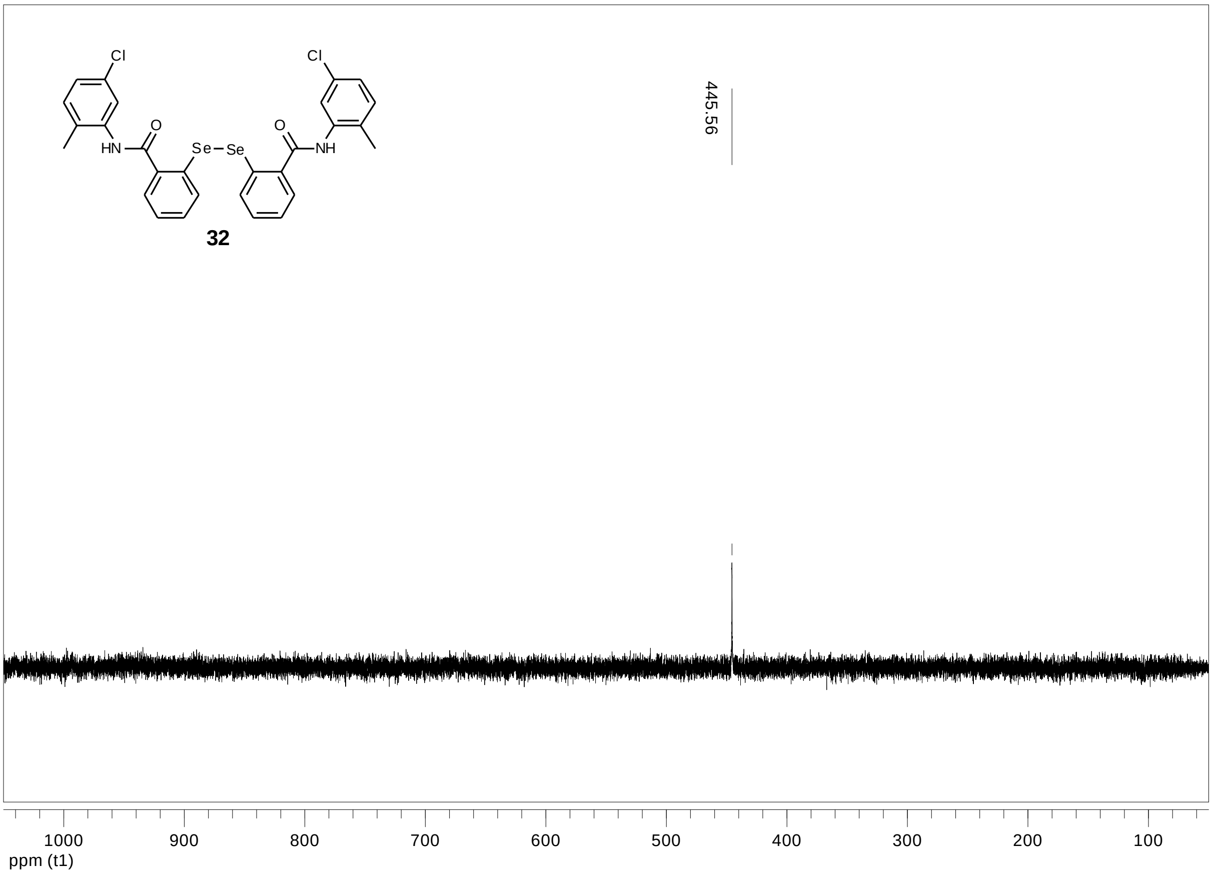

32

Fig. S157. ^{77}Se-NMR (76.24 MHz, DMSO-d_6) spectrum of compound **32**

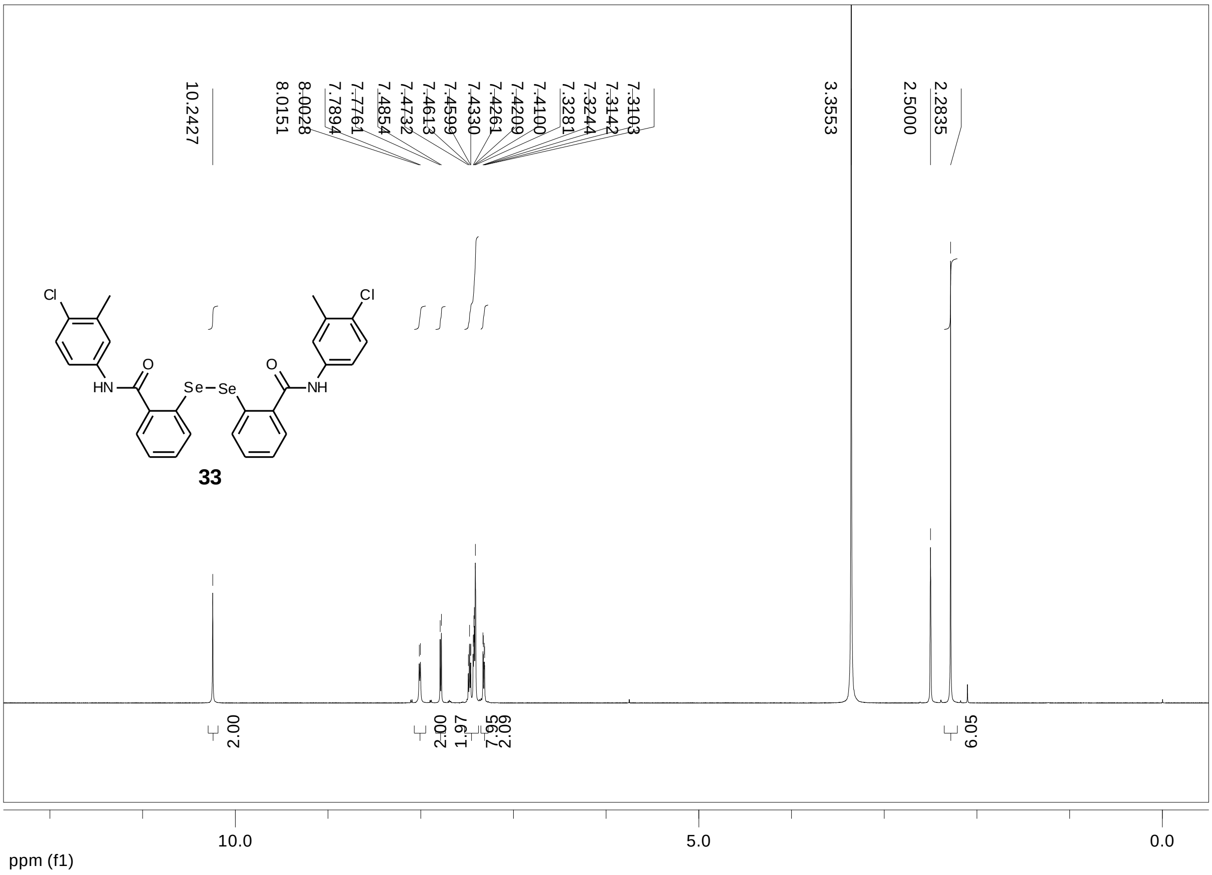

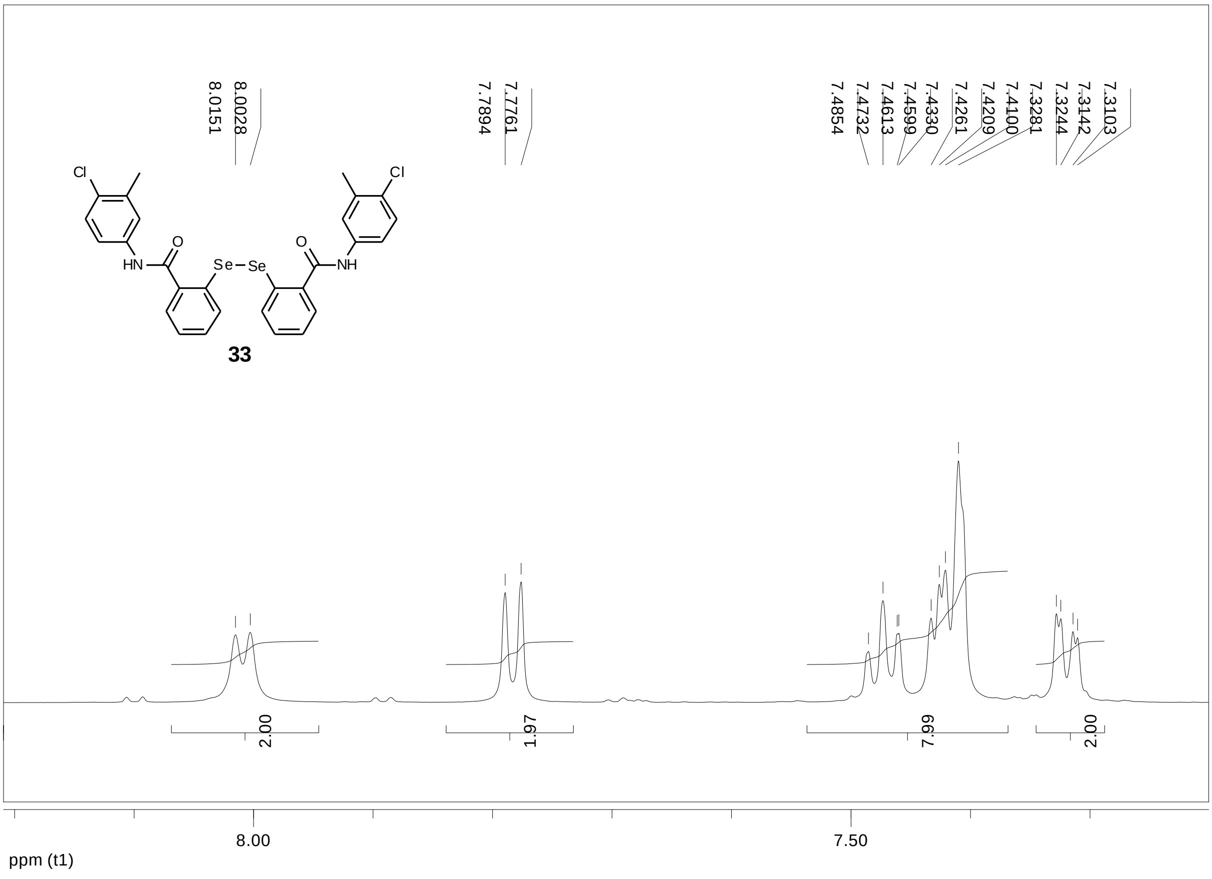

Fig. S158. ^{1}H-NMR (600.6 MHz, DMSO-d_6) spectrum of compound **33**

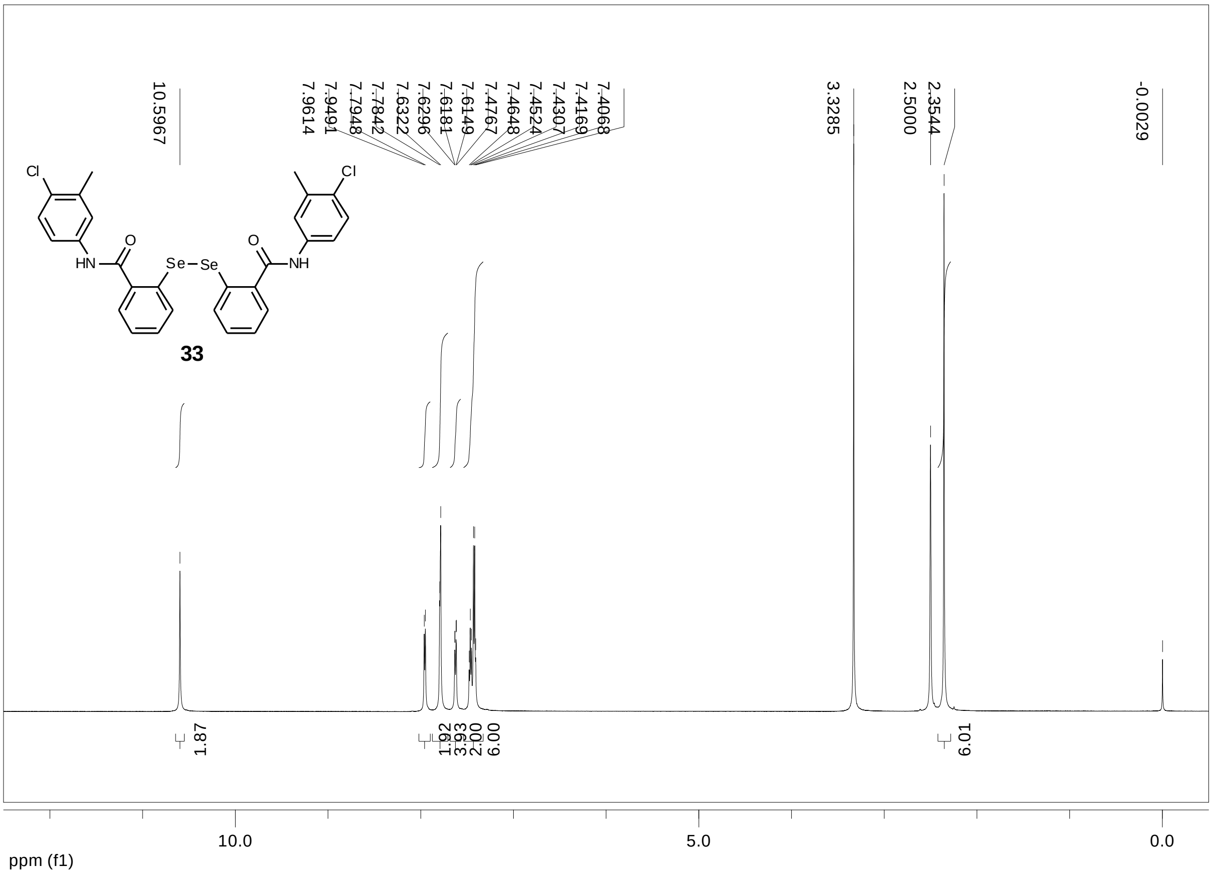

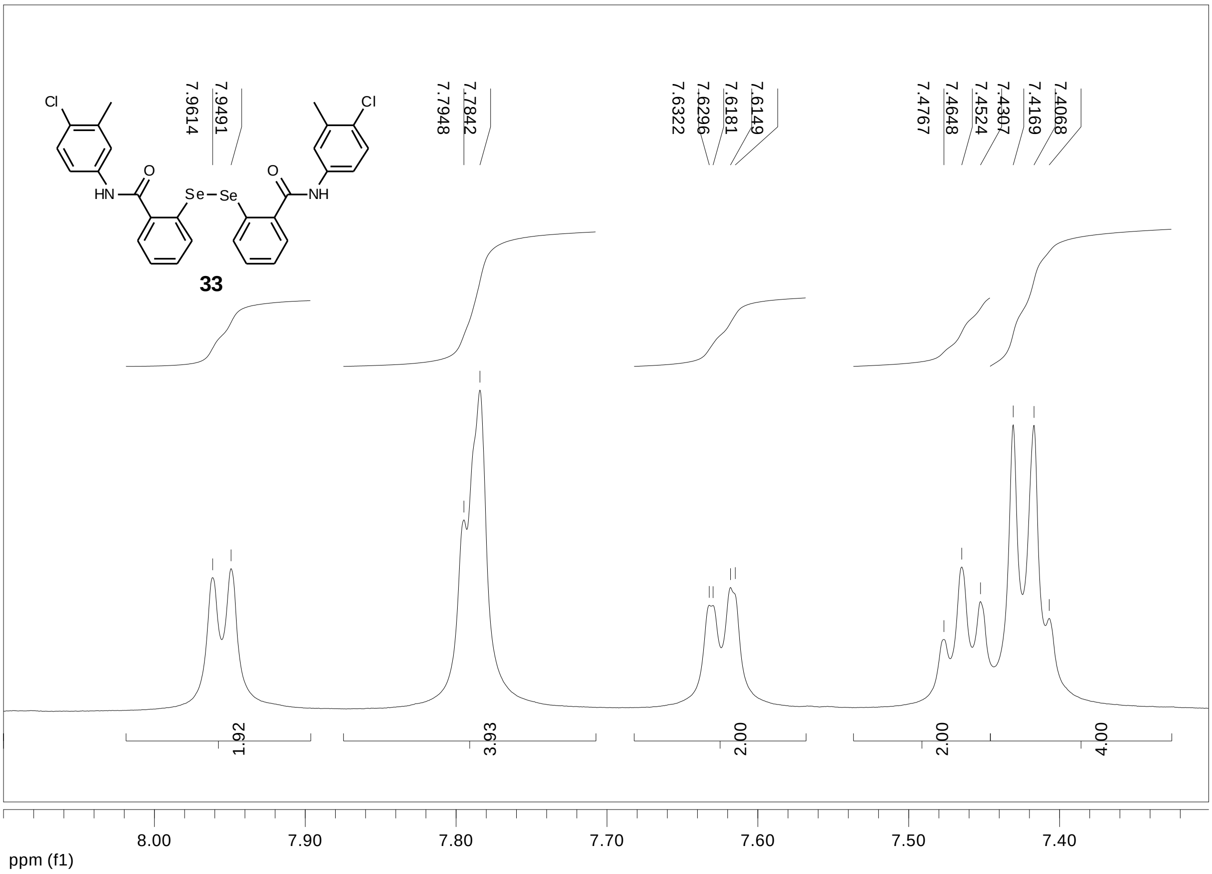

Fig. S159. ^{1}H-NMR (399.8 MHz, DMSO-d_6) spectrum of compound **33**

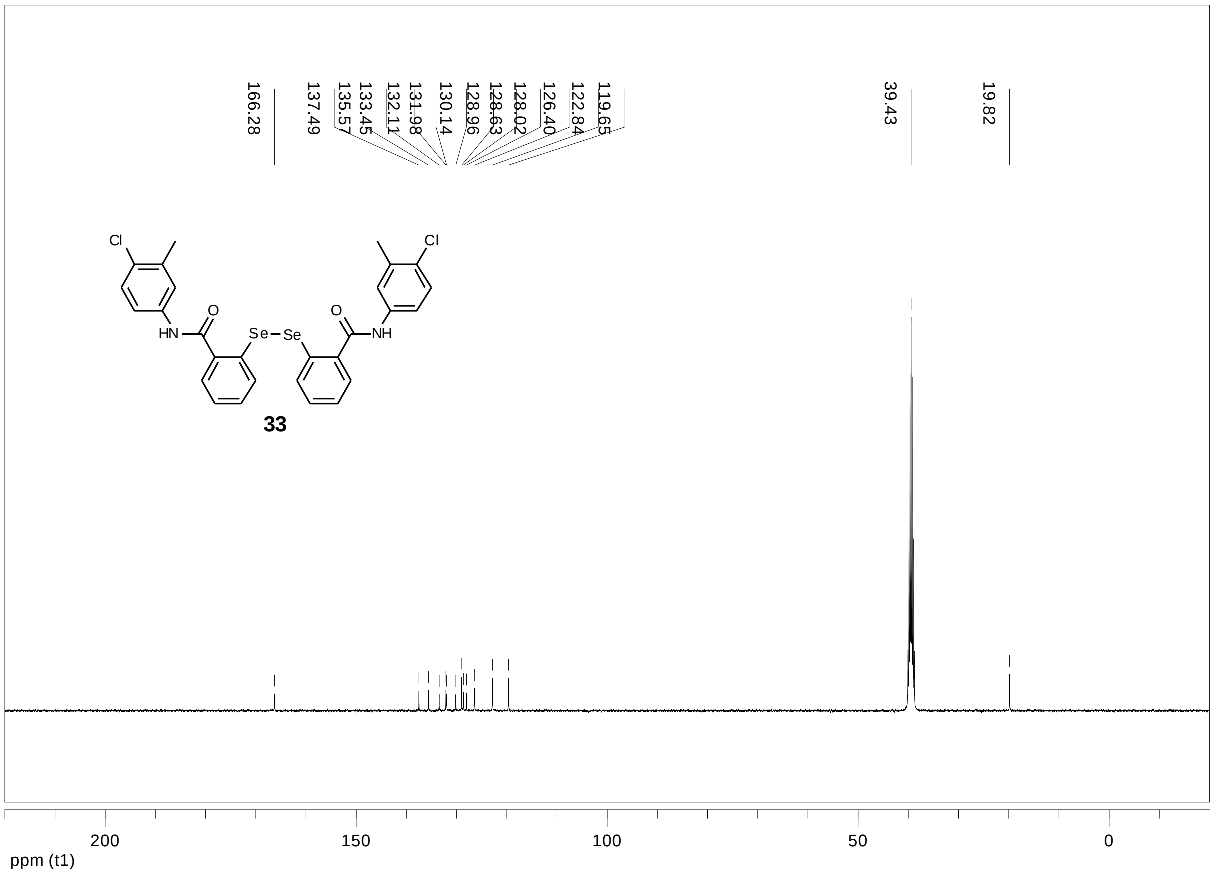

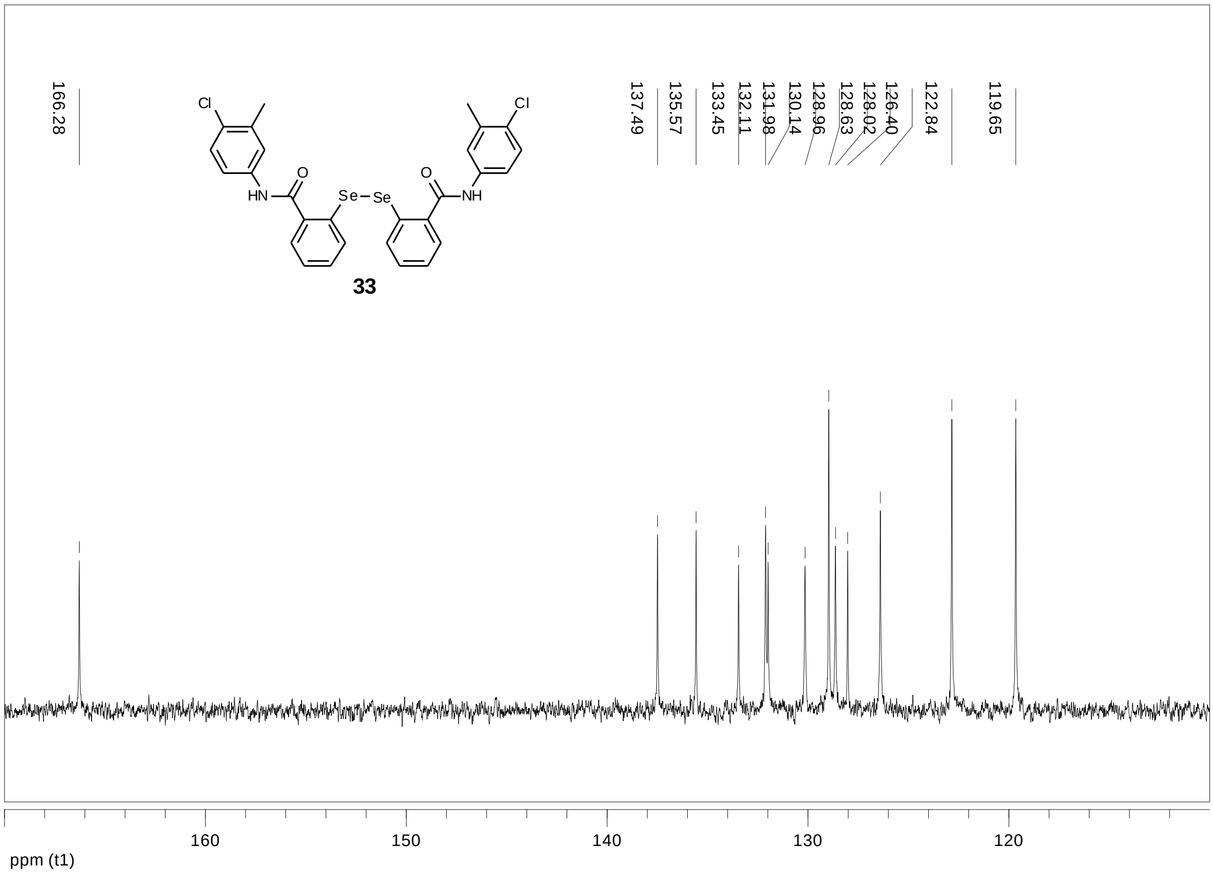

Fig. S160. ^{13}C-NMR (100.5 MHz, DMSO-d_6) spectrum of compound **33**

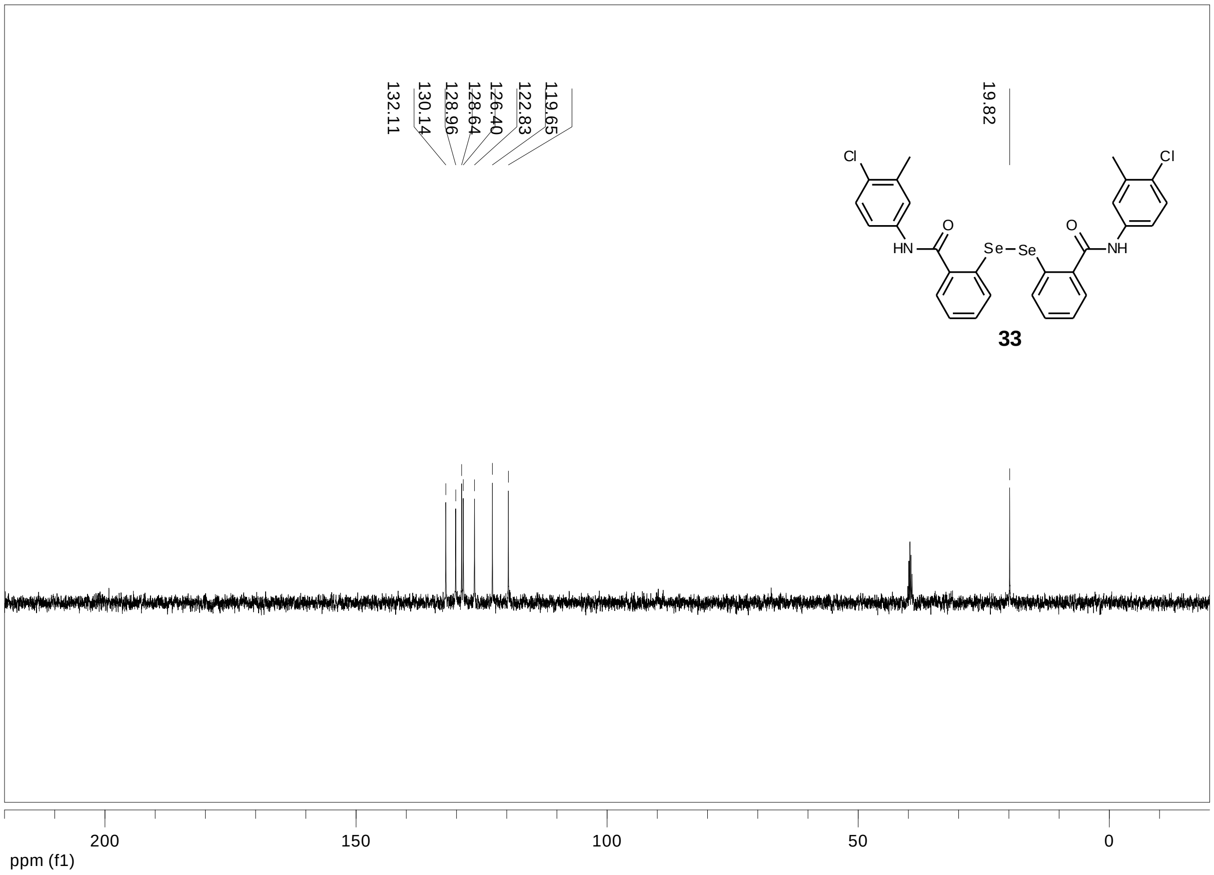

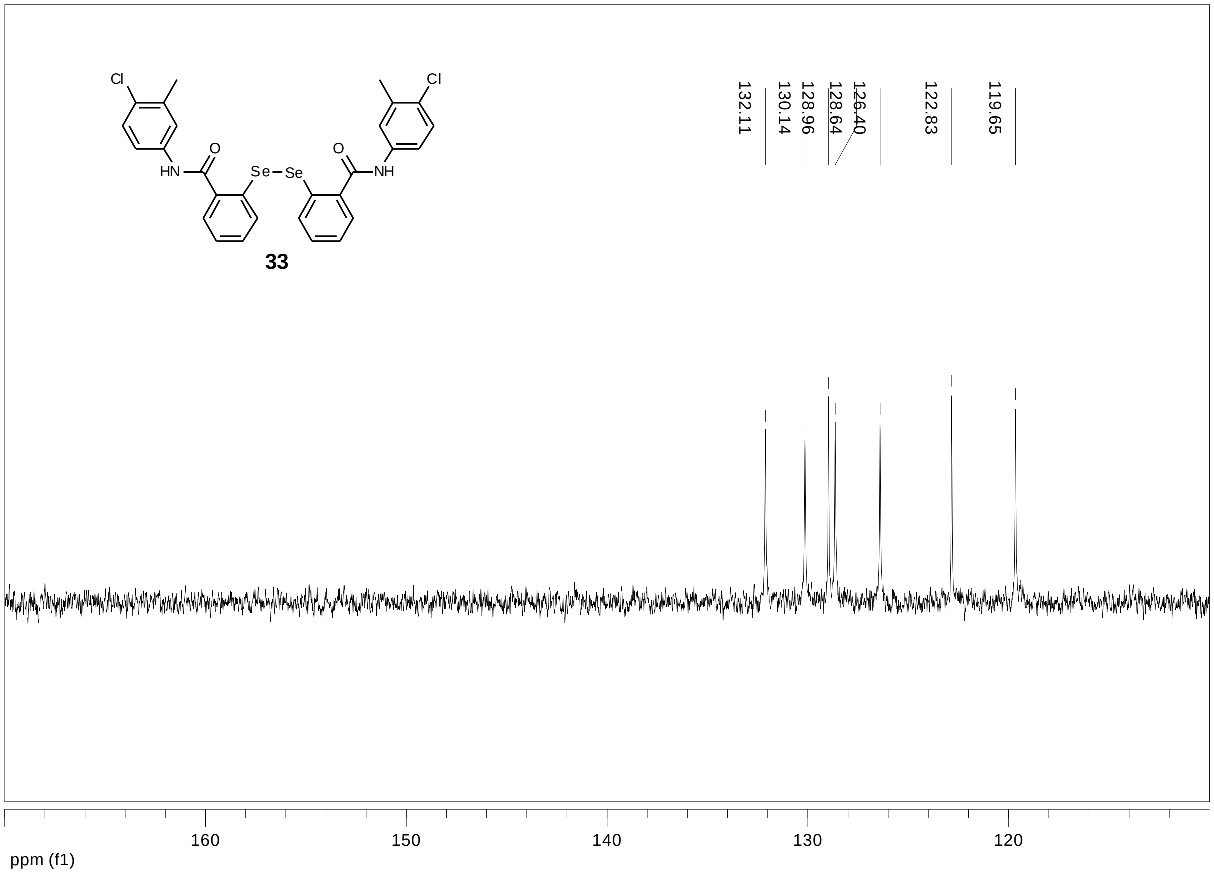

Fig. S161. ^{13}C-NMR (100.5 MHz, DMSO-d_6) dept-135 experiment of compound **33**

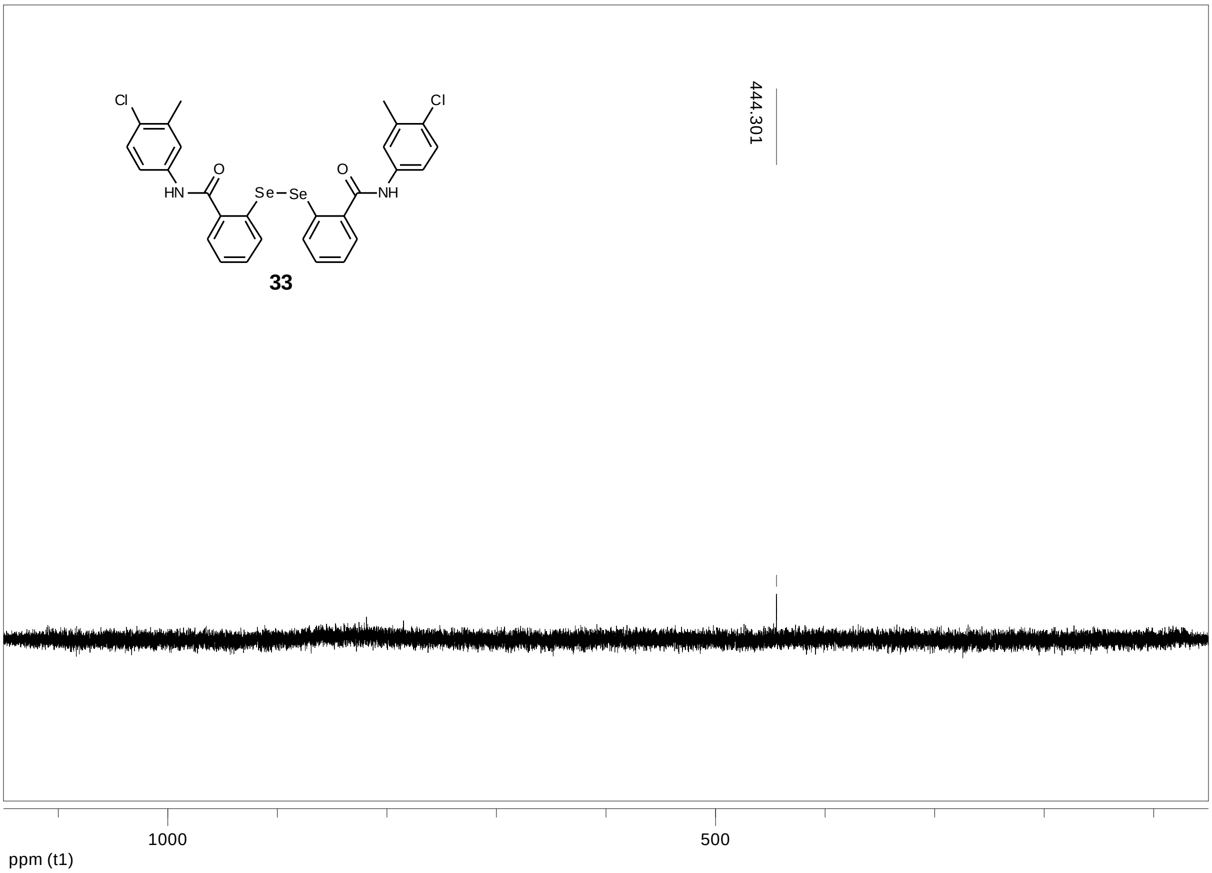

Fig. S162. ^{77}Se-NMR (76.24 MHz, DMSO-d_6) spectrum of compound **33**

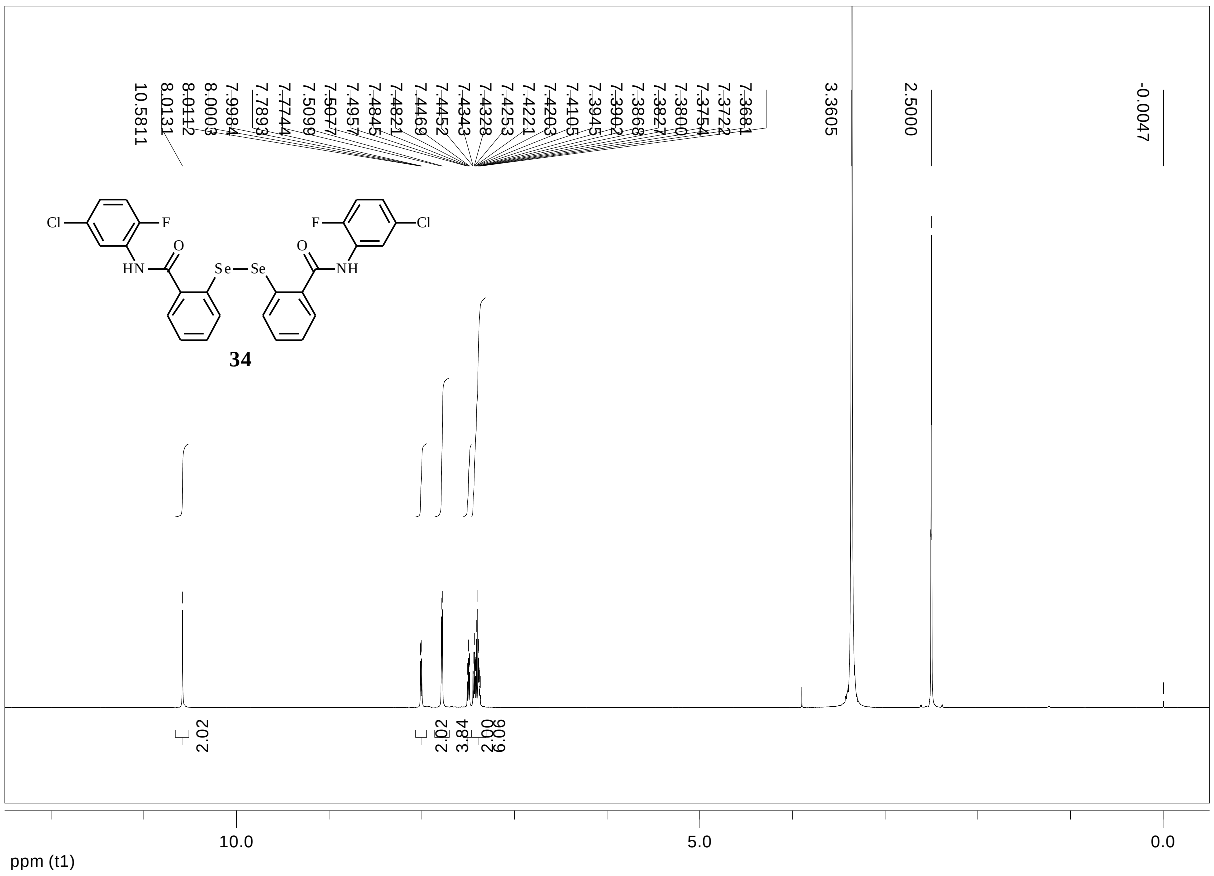

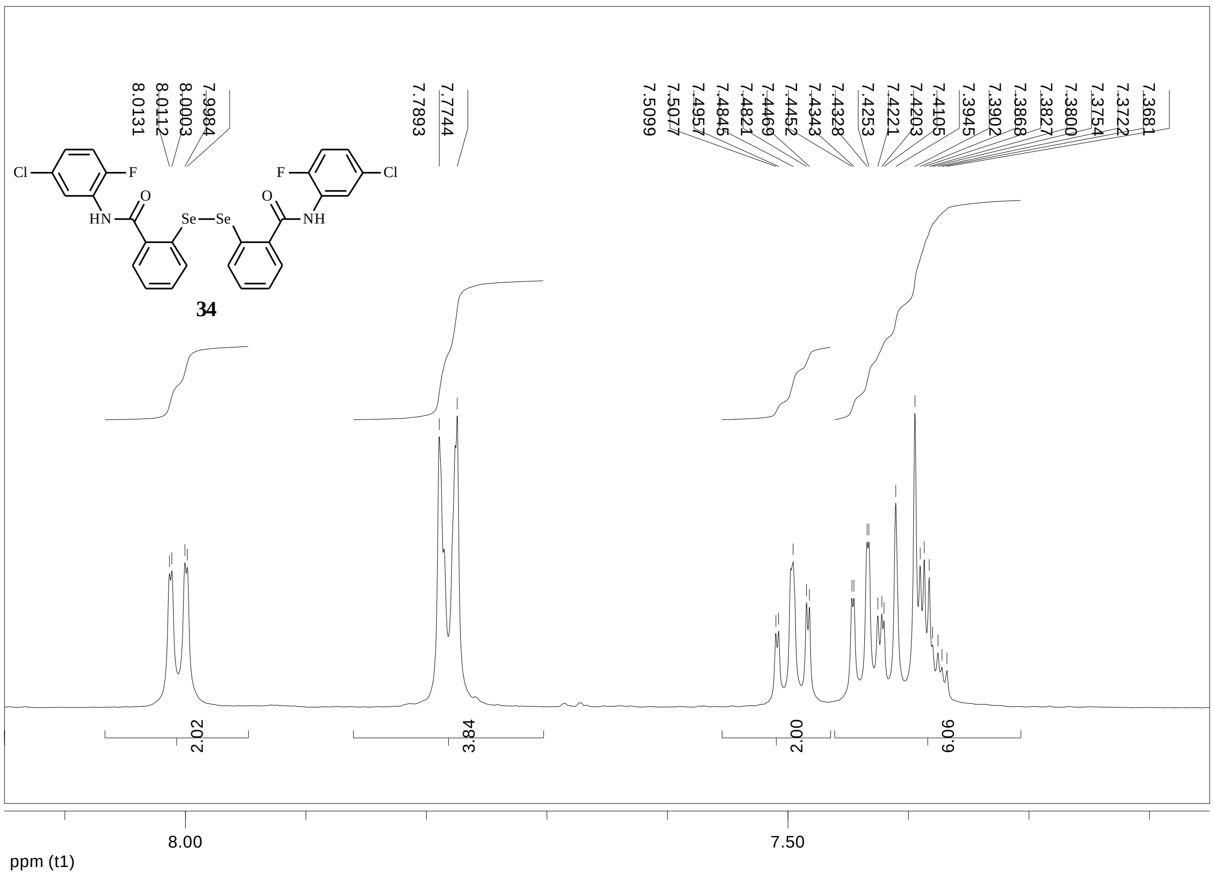

Fig. S163. ^{1}H-NMR (600.6 MHz, DMSO-d_6) spectrum of compound **34**

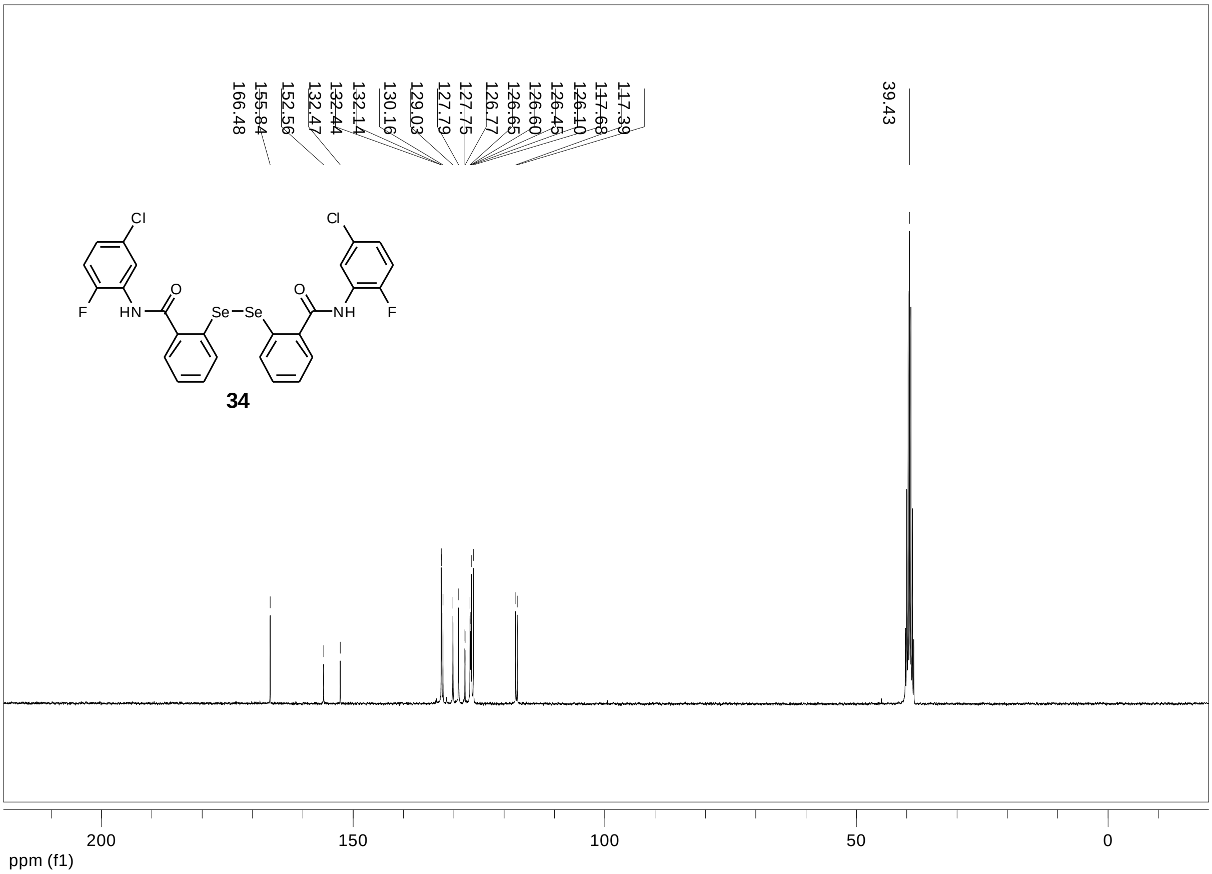

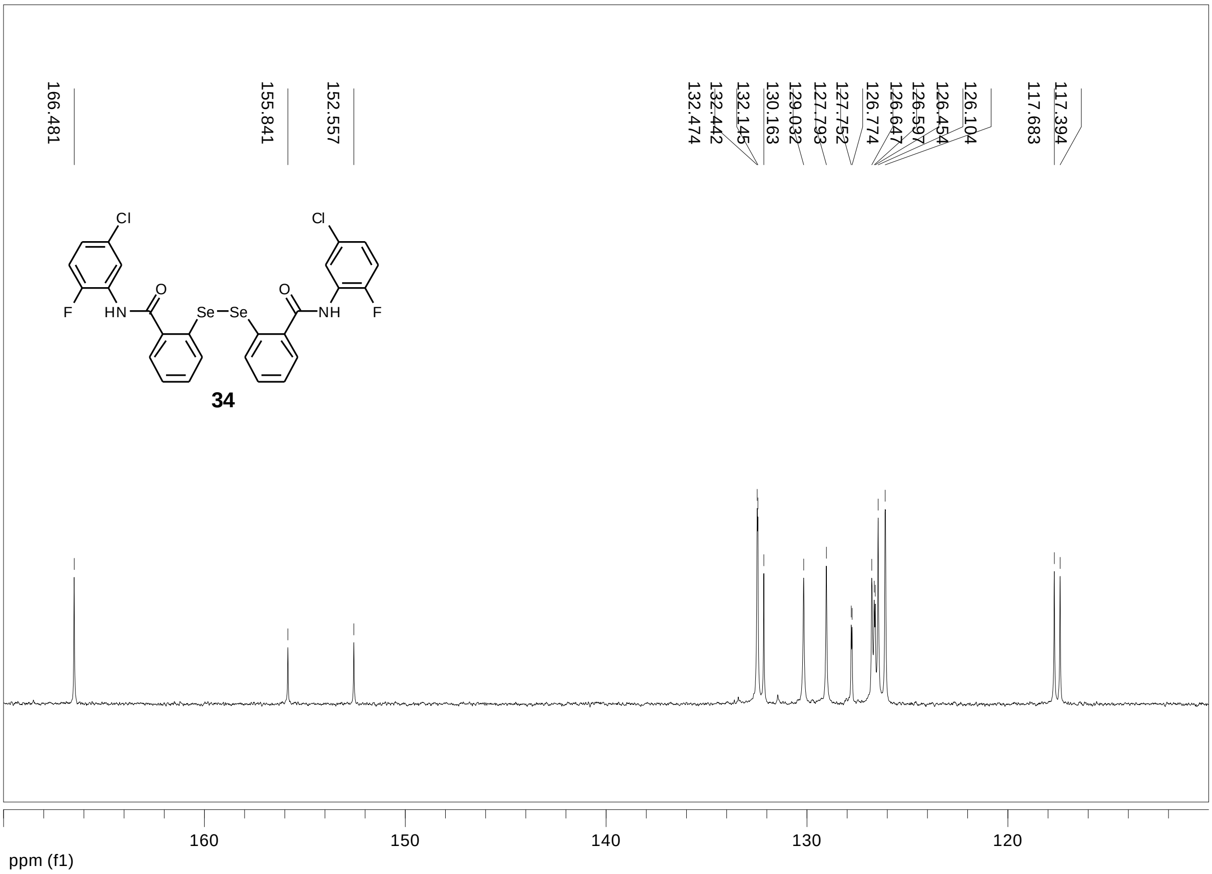

Fig. S164. ^{13}C-NMR (75.45 MHz, DMSO-d_6) spectrum of compound **34**

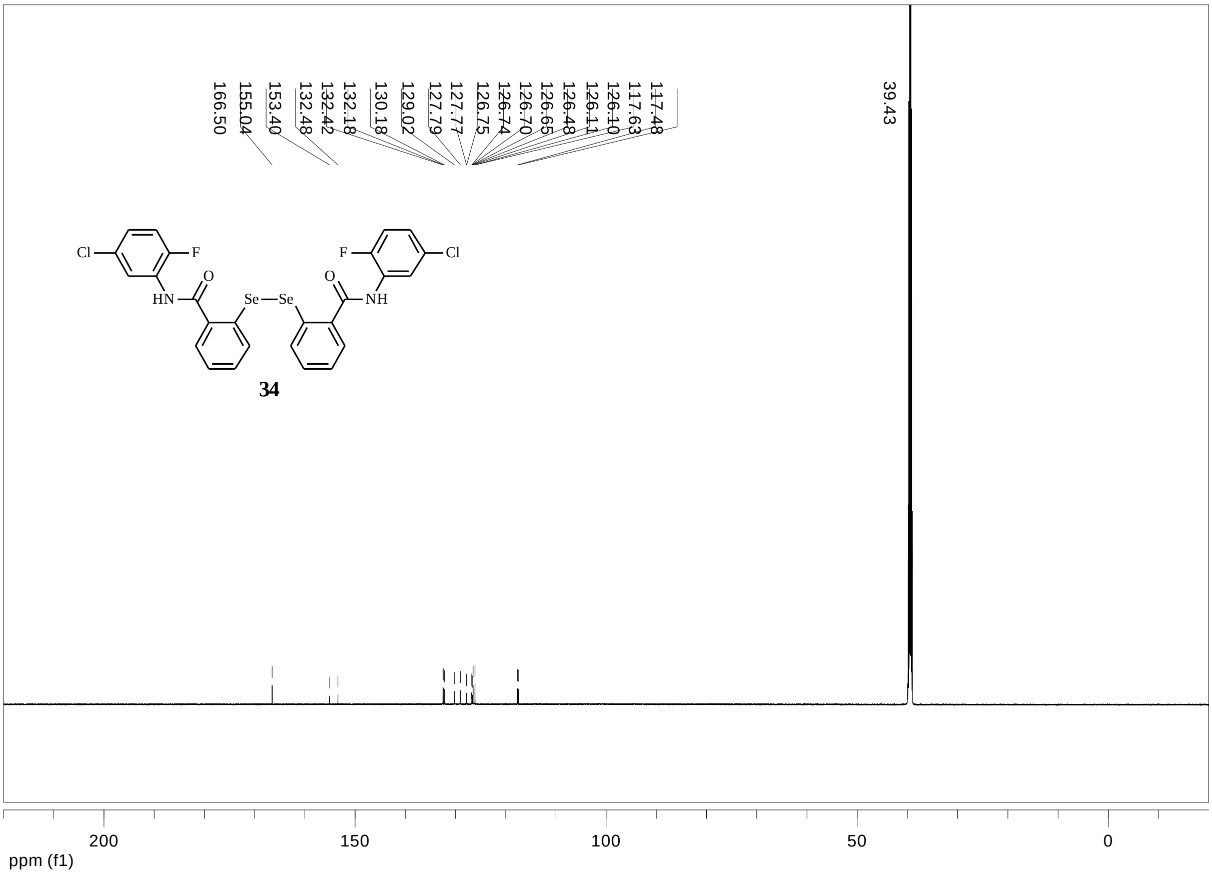

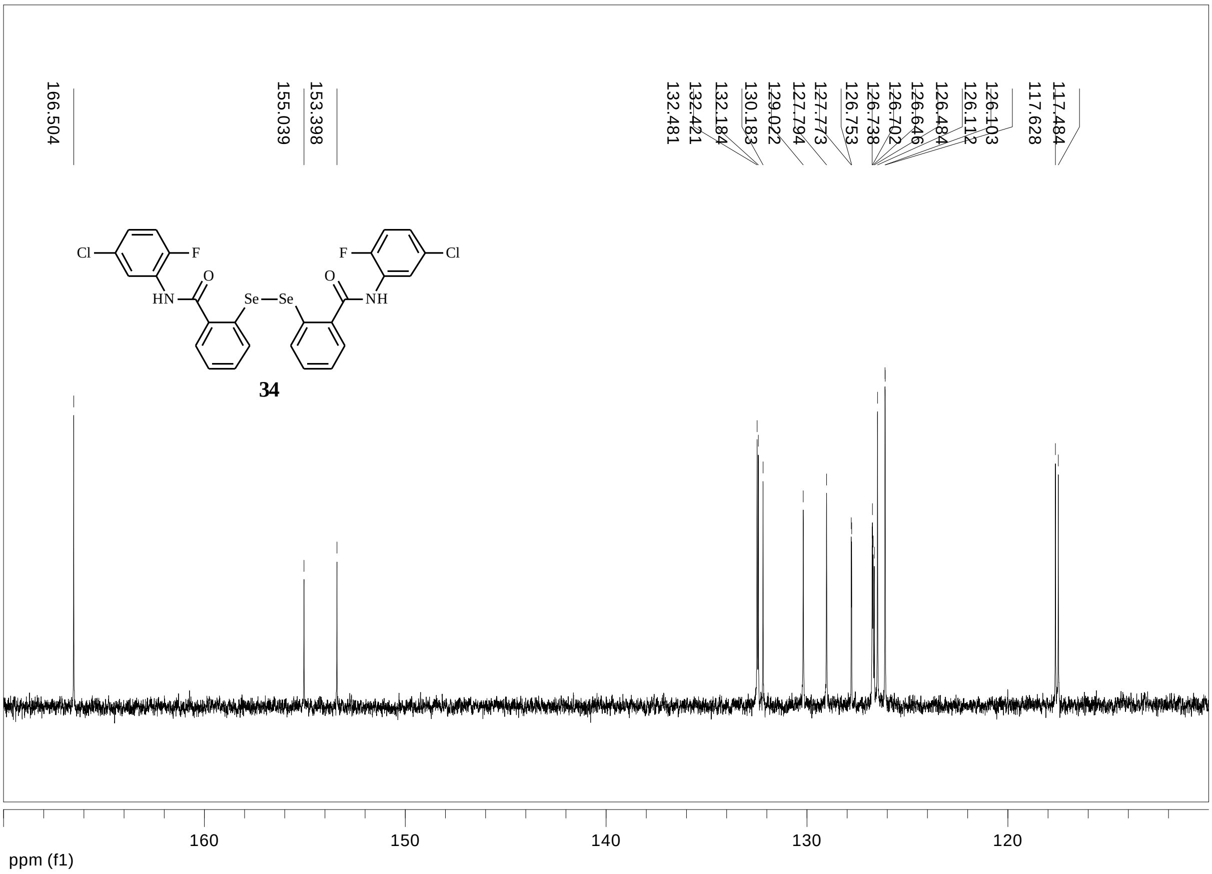

Fig. S165. ^{13}C-NMR (151.0 MHz, DMSO-d_6) spectrum of compound **34**

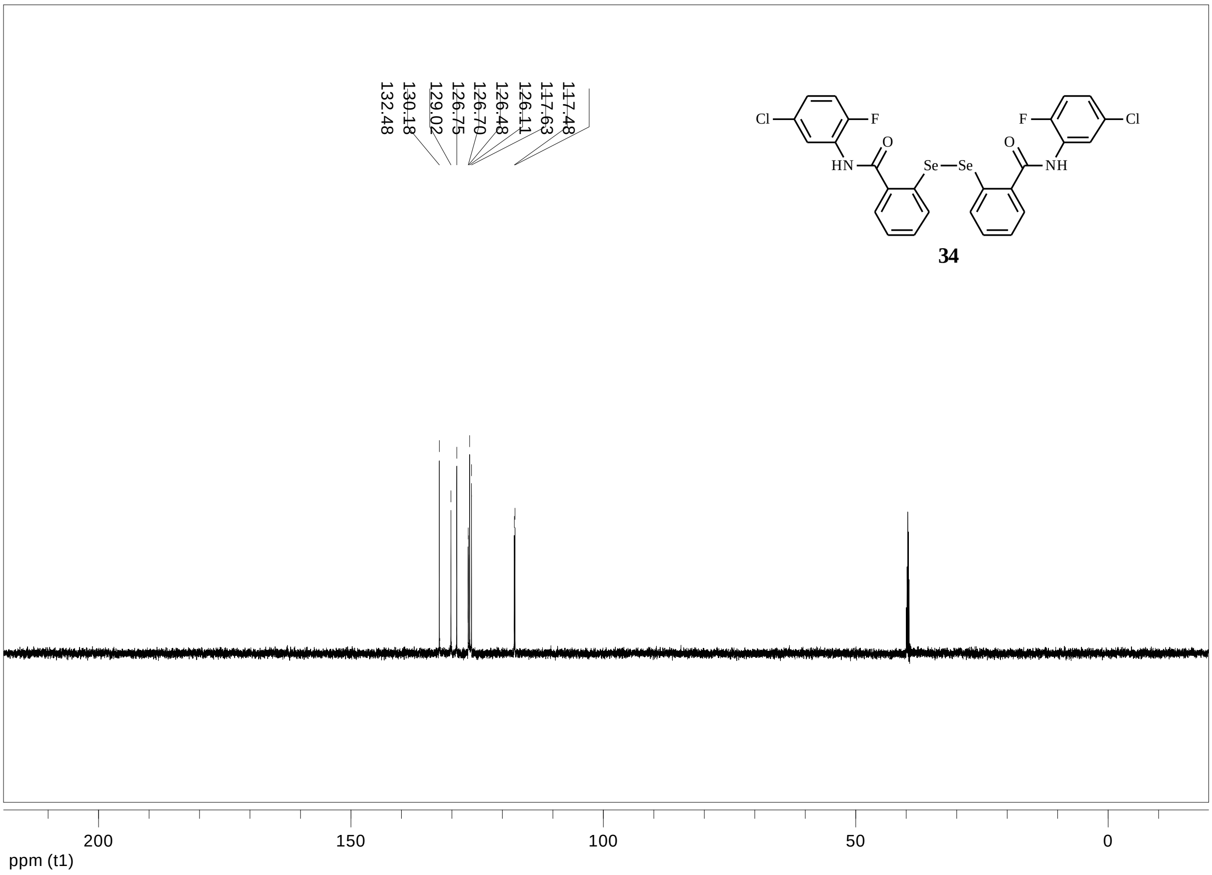

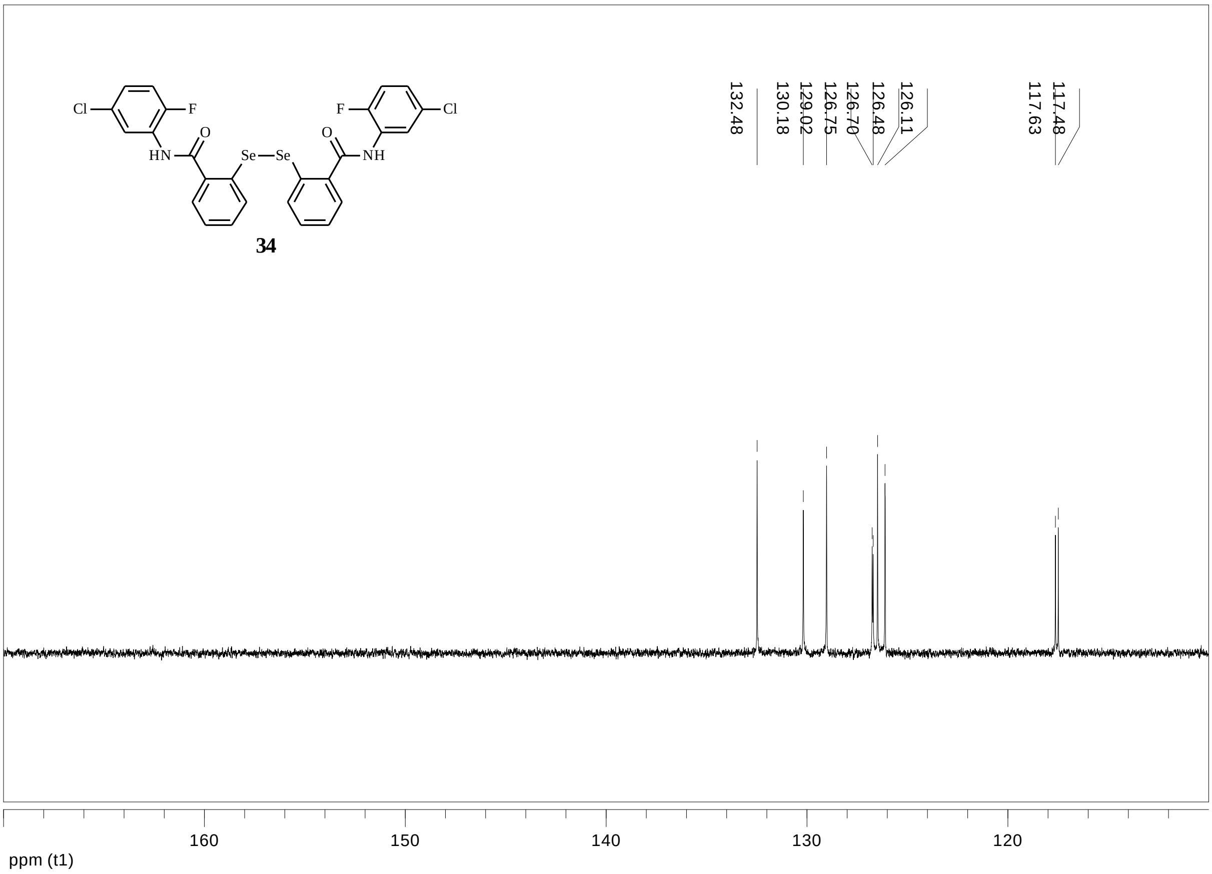

Fig. S166. ^{13}C-NMR (151.0 MHz, DMSO-d_6) dept-135 experiment of compound **34**

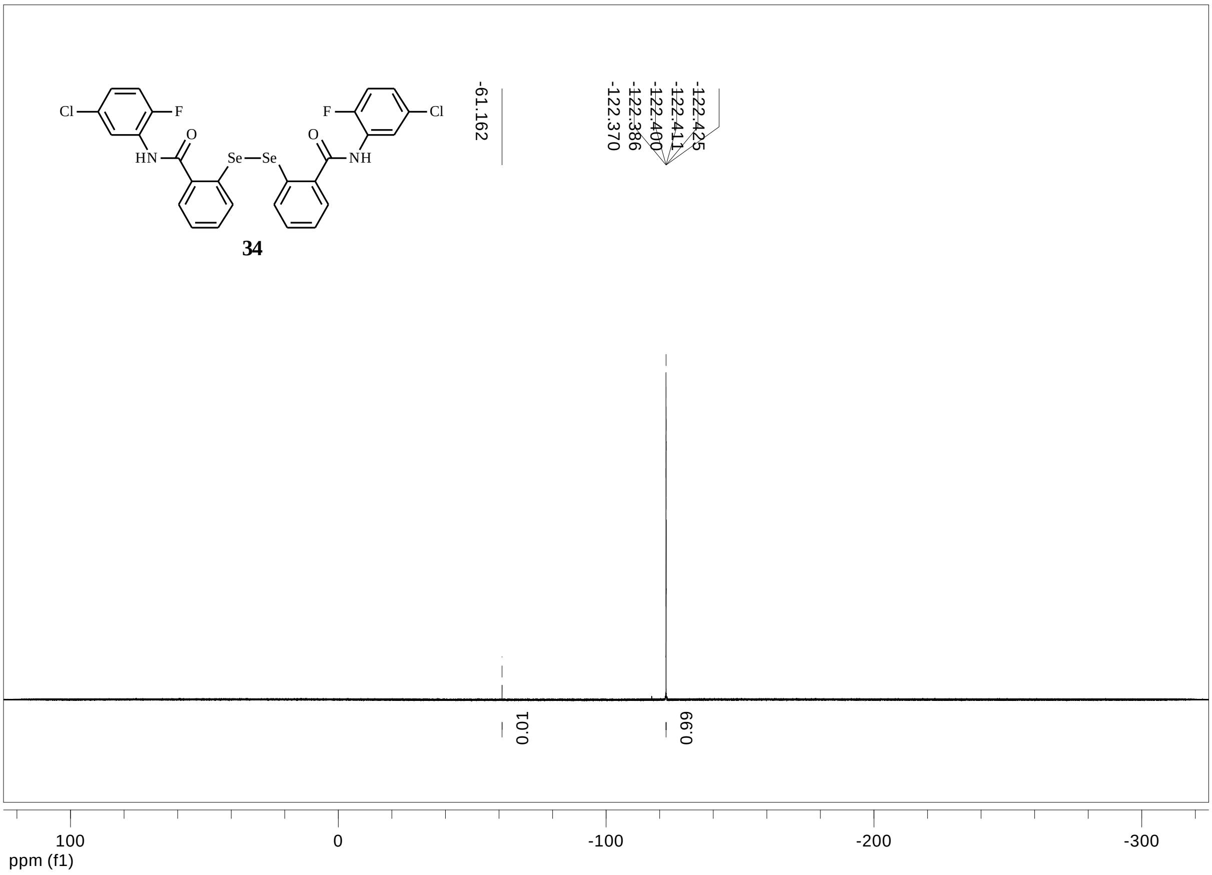

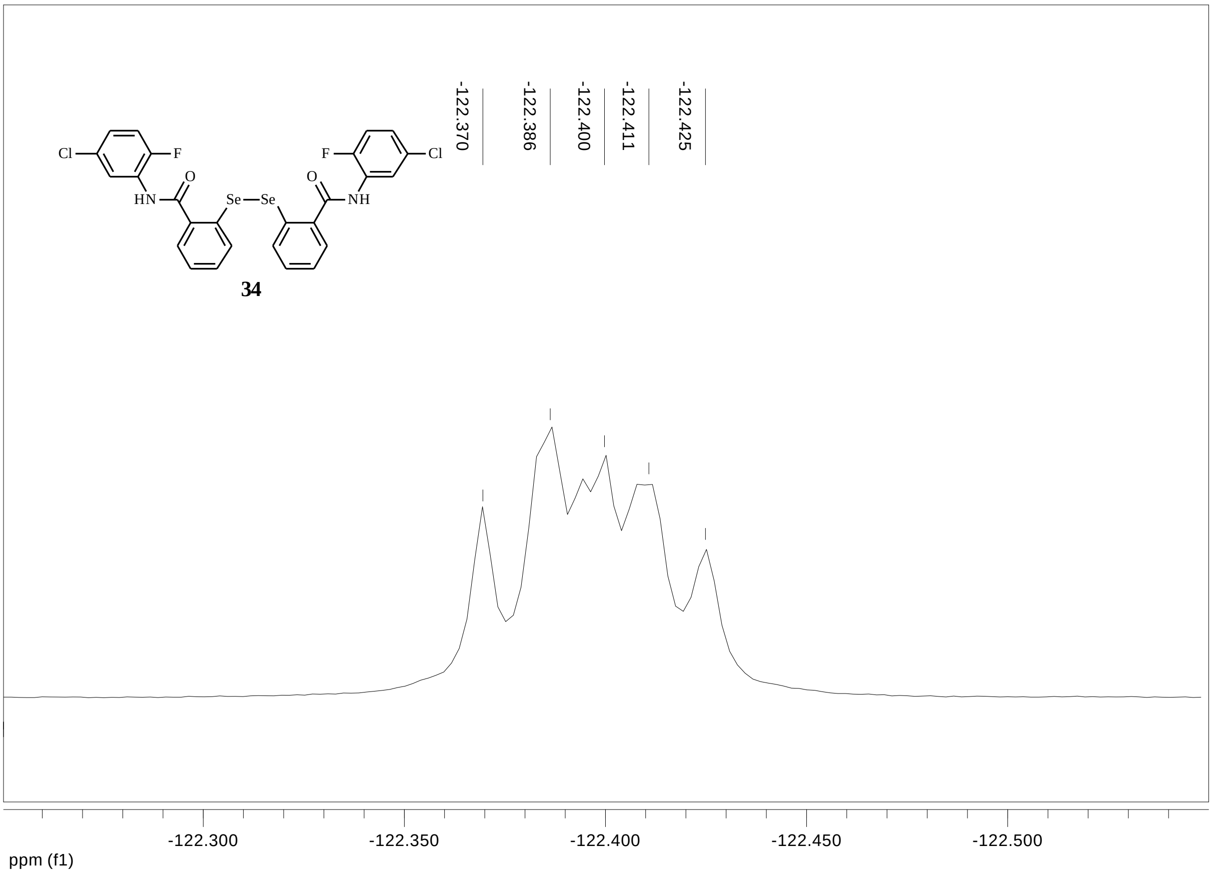

Fig. S167. ^{19}F-NMR (376.2 MHz, DMSO-d_6) spectrum of compound **34**

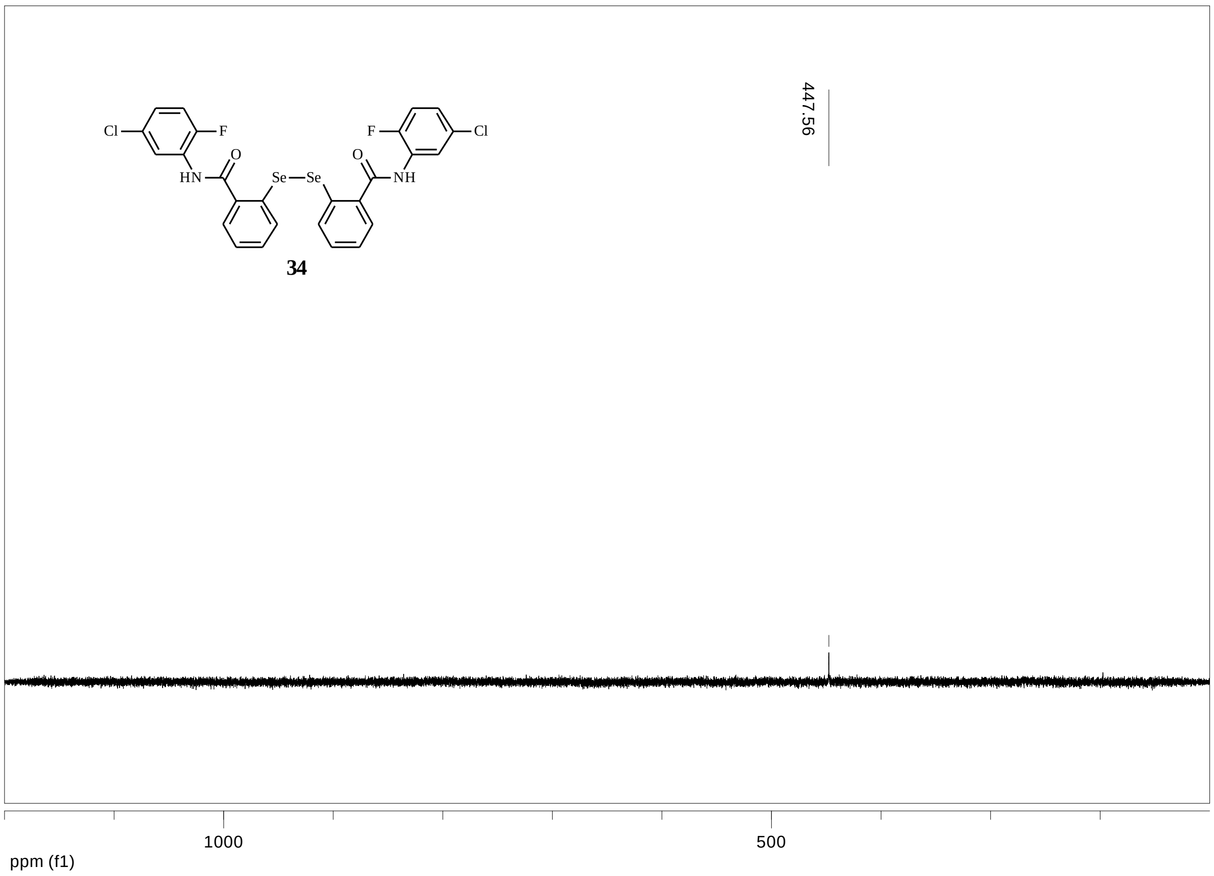

Fig. S168. ^{77}Se-NMR (76.24 MHz, DMSO-d_6) spectrum of compound **34**

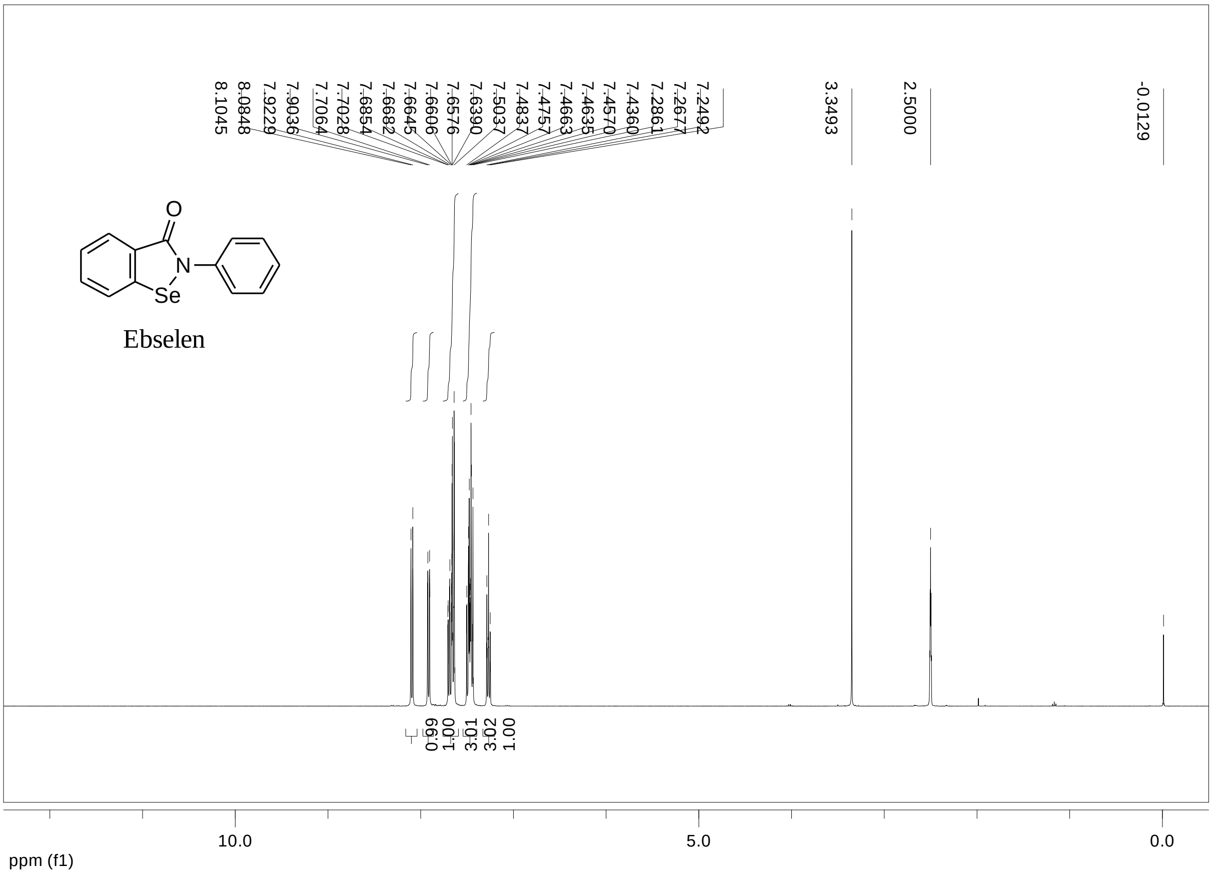

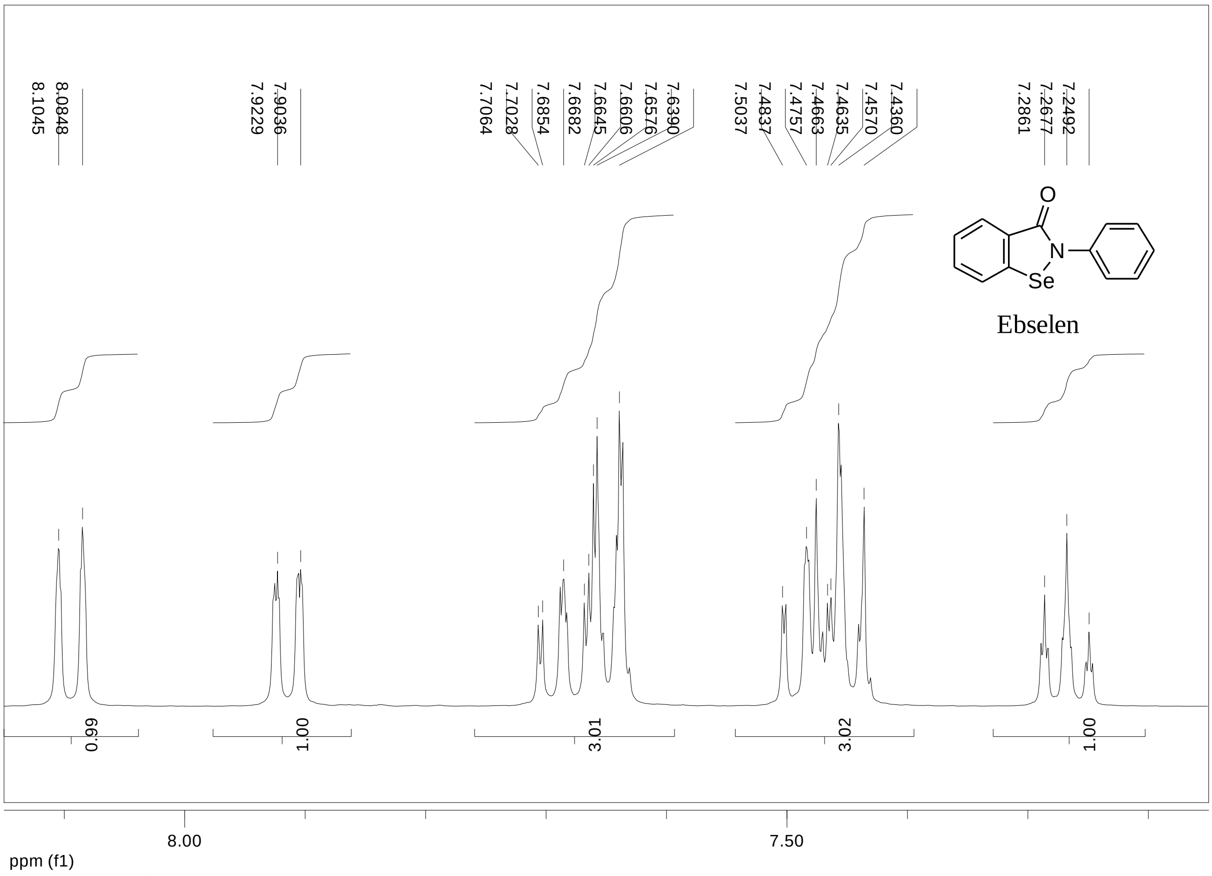

Fig. S169. ^{1}H-NMR (399.78 MHz, DMSO-d_6) spectrum of ebselen

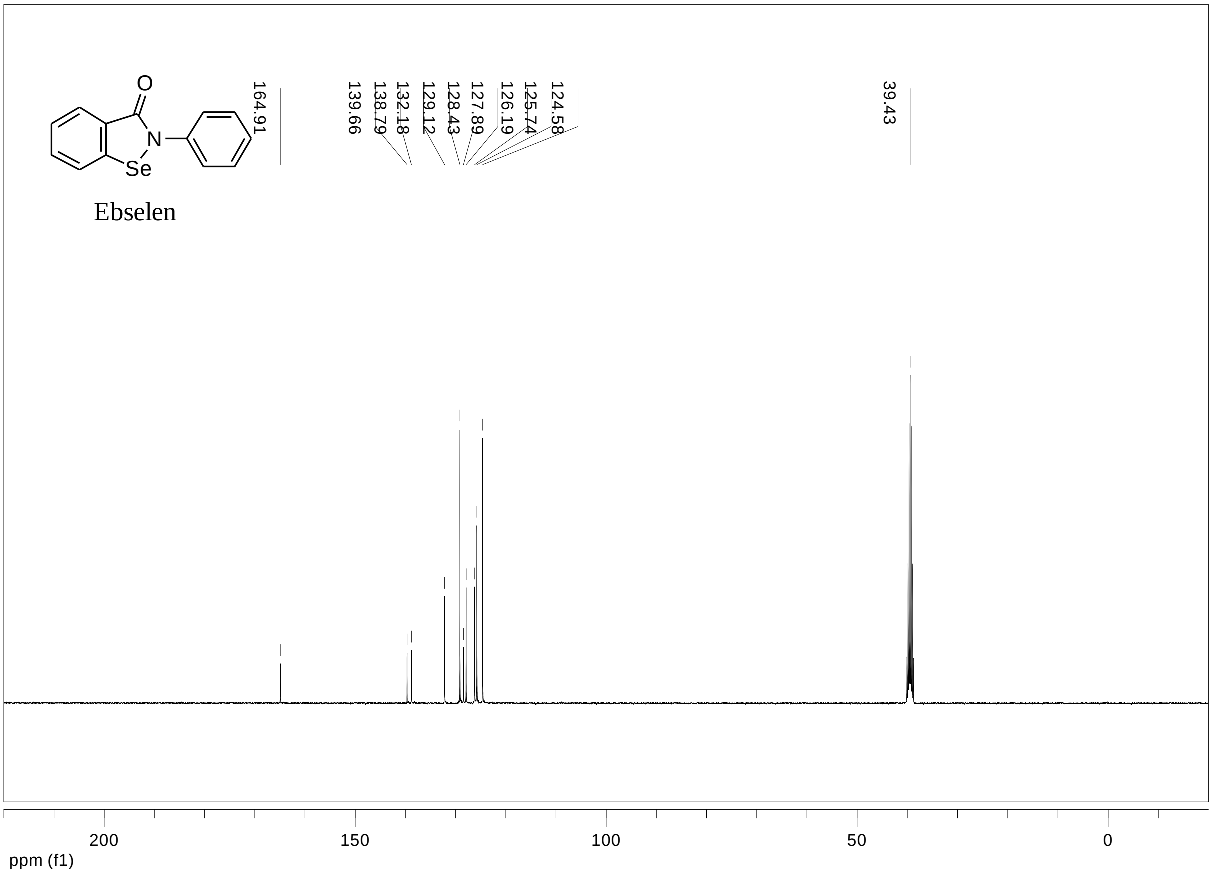

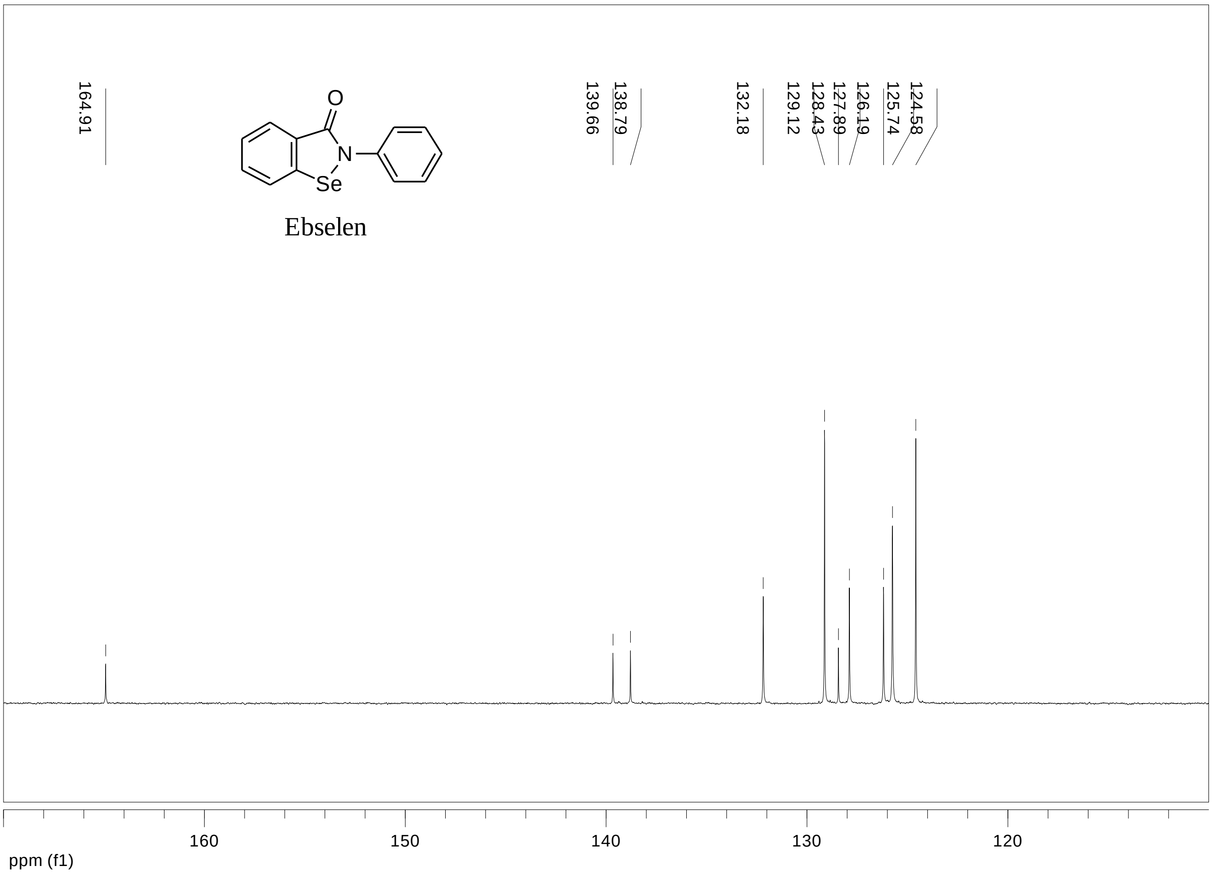

Fig. S170. ^{13}C-NMR (100.52 MHz, DMSO-d_6) spectrum of ebselen

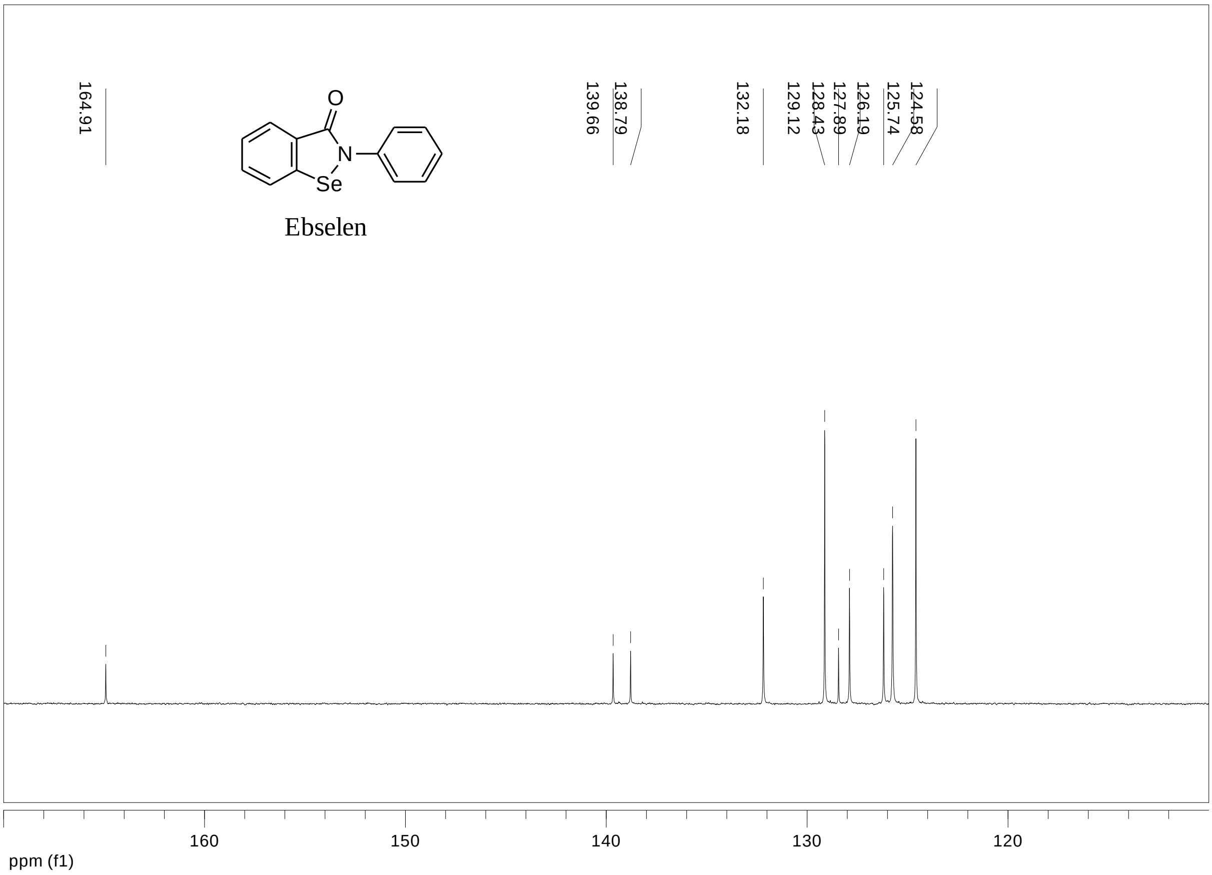

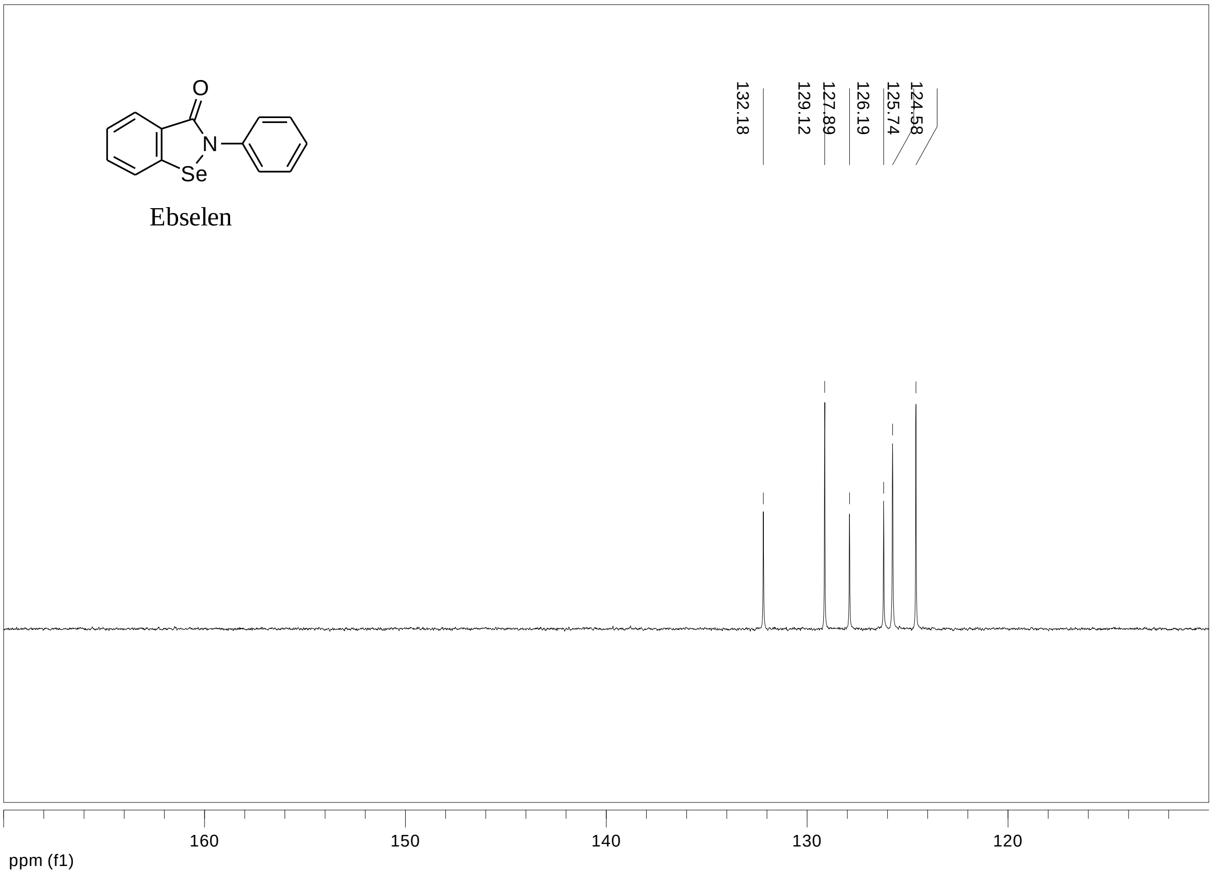

Fig. S171. ^{13}C-NMR (100.52 MHz, DMSO-d_6) dept-135 experiment of ebselen

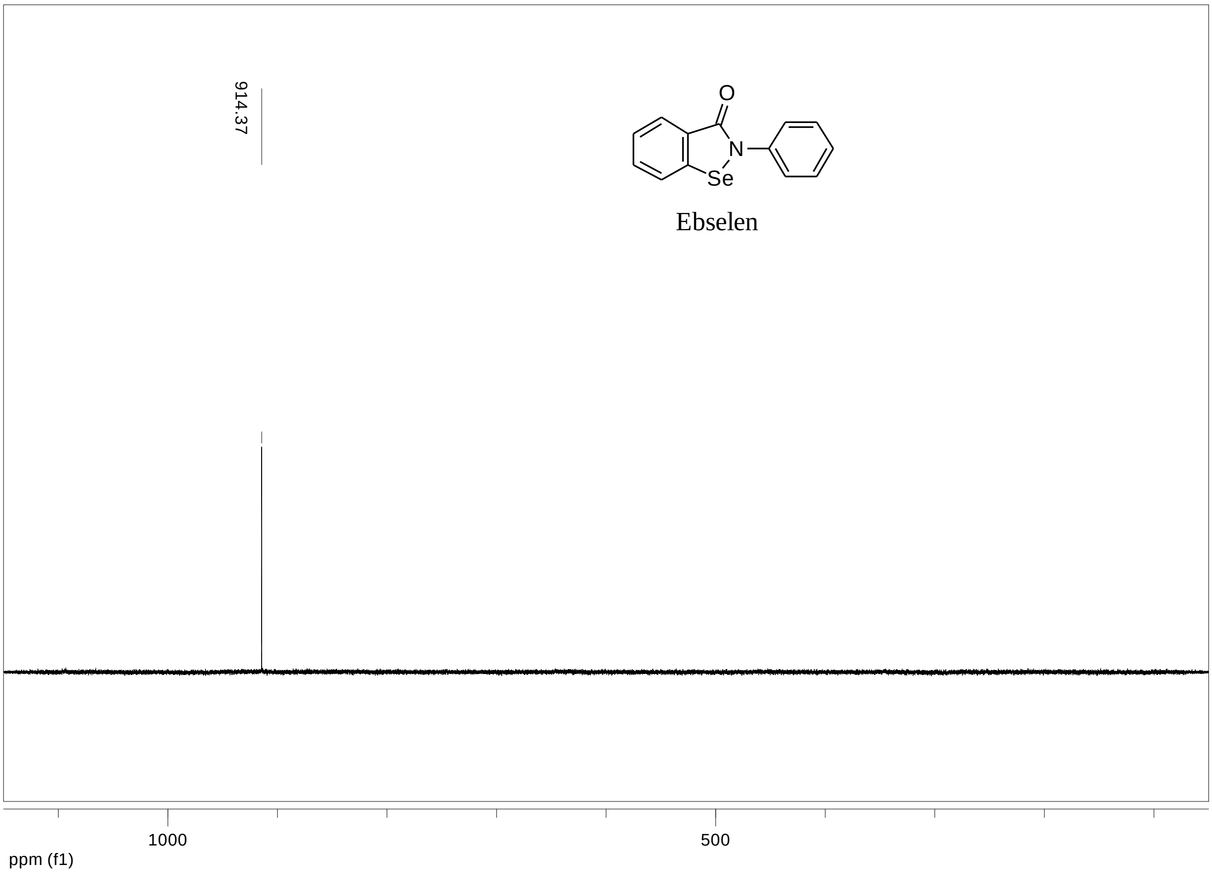

Fig. S172. ^{77}Se-NMR (76.24 MHz, DMSO-d_6) spectrum of ebselen

References:

1. Giurg, M., et al., *Reaction of bis[(2-chlorocarbonyl)phenyl] Diselenide with Phenols, Aminophenols, and Other Amines towards Diphenyl Diselenides with Antimicrobial and Antiviral Properties.* Molecules, 2017. **22**(6).
2. Balkrishna, S.J., B.S. Bhakuni, and S. Kumar, *Copper catalyzed/mediated synthetic methodology for ebselen and related isoselenazolones.* Tetrahedron, 2011. **67**(49): p. 9565-9575.
3. Balkrishna, S.J., et al., *Cu-catalyzed efficient synthetic methodology for ebselen and related Se-N heterocycles.* Org Lett, 2010. **12**(23): p. 5394-7.
4. Garland, M., et al., *Covalent Modifiers of Botulinum Neurotoxin Counteract Toxin Persistence.* ACS Chem Biol, 2019. **14**(1): p. 76-87.
5. Welter, A., et al., *Diselenobis-benzoic acid amides of primary and secondary amines and processes for the treatment of diseases in humans caused by a cell injury*. 1989.
6. Gustafsson, T.N., et al., *Ebselen and analogs as inhibitors of Bacillus anthracis thioredoxin reductase and bactericidal antibacterials targeting Bacillus species, Staphylococcus aureus and Mycobacterium tuberculosis.* Biochim Biophys Acta, 2016. **1860**(6): p. 1265-71.
7. Welter, A., L. Christiaens, and F. Wirtz-Peitz, *Benzisoselenazolones and processes for the treatment of rheumatic and arthritic diseases using them*. 1983.
8. Pacuła, A.J., J. Ścianowski, and K.B. Aleksandrzak, *Highly efficient synthesis and antioxidant capacity of N-substituted benzisoselenazol-3(2H)-ones.* RSC Adv., 2014. **4**(90): p. 48959-48962.
9. Młochowski, J., et al., *Aromatic and Azaaromatic Diselenides, Benzisoselenazolones and Related Compounds as Immunomodulators Active in Humans: Synthesis and Properties.* Liebigs Annalen der Chemie, 1993. **1993**(12): p. 1239-1244.
10. Wan, J., et al., *Benzoselenazole ketone compound and application thereof and bactericide*. 2021.
11. Bender, C.O., et al., *Use of small molecules for the treatment of clostridium difficile toxicity*. 2015.
12. Piętka-Ottlik, M., et al., *Synthesis of new alkylated and methoxylated analogues of ebselen with antiviral and antimicrobial properties.* Arkivoc, 2017. **2017**(2): p. 546-556.
13. Granda, J., et al., *Synthesis of 7- and 8-Functionalized 2-Aminophenoxazinones via Cyclocondensation of 2-Aminophenols.* Synthesis, 2015. **47**(21): p. 3321-3332.
14. Chang, T.C., et al., *Synthesis and biological evaluation of ebselen and its acyclic derivatives.* Chem Pharm Bull (Tokyo), 2003. **51**(12): p. 1413-6.
15. Daolio, A., et al., *Binding motif of ebselen in solution: chalcogen and hydrogen bonds team up.* New Journal of Chemistry, 2020. **44**(47): p. 20697-20703.
16. Huff, S., et al., *Discovery and Mechanism of SARS-CoV-2 Main Protease Inhibitors.* J Med Chem, 2022. **65**(4): p. 2866-2879.
17. Osajda, M. and J. Młochowski, *The reactions of 2-(chloroseleno)benzoyl chloride with nucleophiles.* Tetrahedron, 2002. **58**(37): p. 7531-7537.
18. Mukherjee, S., et al., *Ebselen inhibits hepatitis C virus NS3 helicase binding to nucleic acid and prevents viral replication.* ACS Chem Biol, 2014. **9**(10): p. 2393-403.
19. Simanjuntak, Y., et al., *Ebselen alleviates testicular pathology in mice with Zika virus infection and prevents its sexual transmission.* PLoS Pathog, 2018. **14**(2): p. e1006854.
